# Supplementary material for: Soy Protein Isolate Affects Blood and Brain Biomarker Expression in a Mouse Model of Fragile X
Source: Int J Mol Sci. 2025 Jun 26;26(13):6137. doi: 10.3390/ijms26136137 (PMC12250412; doi:10.3390/ijms26136137)
Supplement: Supplementary file 1 [file ijms-26-06137-s001.zip › Supplementary File S1.pdf]

Teklad Custom Diet

TD.180374

0.2% Na Control Diet (93G, R)

Formula

Formula	g/Kg
Casein	200.0
L-Cystine	3.0
Corn Starch	394.886
Maltodextrin	132.0
Sucrose	100.0
Soybean Oil	70.0
Cellulose	50.0
Mineral Mix, AIN-93G-MX (94046)	35.0
Sodium Chloride	2.5
Vitamin Mix, AIN-93-VX (94047)	10.0
Choline Bitartrate	2.5
TBHQ, antioxidant	0.014
Red Food Color	0.1

Footnote

Modification of AIN-93G (TD.94045) to increase sodium to 2 g/kg diet (0.2%). Red food dye added for visual differentiation.

Selected Nutrient Information[1]

	% by weight	% kcal from
**Protein**	17.7	18.9
**Carbohydrate**	59.8	63.8
**Fat**	7.2	17.3

Kcal/g 3.7

[1] Values are calculated from ingredient analysis or manufacturer data

Speak With A Nutritionist

+ **(800) 483·5523**
+ **askanutritionist@envigo.com**

Teklad diets are designed & manufactured for research purposes only.

Key Features

+ **Purified Diet**
+ **AIN-93G Modification**
+ **Sodium Chloride**
+ **Color Coded Red**

Key Planning Information

+ *Products are made fresh to order*
+ Store product at 4°C or lower
+ Use within 6 months (applicable to most diets)
+ Box labeled with product name, manufacturing date, and lot number
+ Replace diet at minimum once per week
  More frequent replacement may be advised
+ Lead time:
  · 2 weeks non-irradiated
  · 4 weeks irradiated

Product Specific Information

+ 1/2" Pellet or Powder (free flowing)
+ Minimum order 3 Kg
+ Irradiation not advised
  · Contact a nutritionist for recommendations

Options (fees will apply)

+ Rush order (pending availability)
+ Irradiation (see Product Specific Information)
+ Vacuum packaging (1 and 2 Kg)

Contact Us

Obtain pricing · Check order status

+ **teklad@envigo.com**
+ **(800) 483·5523**

International Inquiry *(outside USA or Canada)*

+ **askanutritionist@envigo.com**

Place Your Order (USA & Canada)

Please Choose One

+ **www.envigo.com/teklad-orders**
+ **tekladorders@envigo.com**
+ **(800) 483·5523**
+ **(608) 277·2066** *facsimile*

JBF

© 2015 Envigo

05/17/18

Teklad Custom Diet

TD.180375

Soy Protein Diet (93G, G)

Formula

Formula	g/Kg
Isolated Soy Protein	200.0
L-Cystine	1.3
L-Methionine	2.4
Corn Starch	405.726
Maltodextrin	132.0
Sucrose	100.0
Cellulose	50.0
Soybean Oil	70.0
Trace Mineral Mix, AIN-93G (06095)	5.0
Calcium Phosphate, dibasic	9.5
Calcium Carbonate	4.5
Potassium Chloride	6.3
Magnesium Oxide	0.66
Vitamin Mix, AIN-93-VX (94047)	10.0
Choline Bitartrate	2.5
TBHQ, antioxidant	0.014
Green Food Color	0.1

Footnote

Modified from AIN-93G to replace casein with soy protein isolate and to match macrominerals to control diet TD.180374 including: 0.5% Ca, 0.3% Avail. P, 0.2% Na, 0.36% K, 0.3% Cl and 0.05% Mg. Green food dye added for visual differentiation.

Selected Nutrient Information[1]

	% by weight	% kcal from
**Protein**	17.8	18.8
**Carbohydrate**	60.7	64.1
**Fat**	7.2	17.1

Kcal/g **3.8**

[1] Values are calculated from ingredient analysis or manufacturer data

Speak With A Nutritionist

+ **(800) 483·5523**
+ **askanutritionist@envigo.com**

Teklad diets are designed & manufactured for research purposes only.

Key Features

+ **Purified Diet**
+ **AIN-93G Modification (Reproduction)**
+ **Soy Protein Isolate**
+ **Matched to Casein Diets**

Key Planning Information

+ *Products are made fresh to order*
+ Store product at 4°C or lower
+ Use within 6 months (applicable to most diets)
+ Box labeled with product name, manufacturing date, and lot number
+ Replace diet at minimum once per week

  More frequent replacement may be advised
+ Lead time:
  · 2 weeks non-irradiated
  · 4 weeks irradiated

Product Specific Information

+ 1/2" Pellet or Powder (free flowing)
+ Minimum order 3 Kg
+ Irradiation not advised
  · Contact a nutritionist for recommendations

Options (fees will apply)

+ Rush order (pending availability)
+ Irradiation (see Product Specific Information)
+ Vacuum packaging (1 and 2 Kg)

Contact Us

Obtain pricing · Check order status

+ **teklad@envigo.com**
+ **(800) 483·5523**

International Inquiry *(outside USA or Canada)*

+ **askanutritionist@envigo.com**

Place Your Order (USA & Canada)

Please Choose One

+ **www.envigo.com/teklad-orders**
+ **tekladorders@envigo.com**
+ **(800) 483·5523**
+ **(608) 277·2066** *facsimile*

JBF

© 2015 Envigo

05/17/18
