# Supplementary material for: Soy Protein Isolate Affects Blood and Brain Biomarker Expression in a Mouse Model of Fragile X
Source: Int J Mol Sci. 2025 Jun 26;26(13):6137. doi: 10.3390/ijms26136137 (PMC12250412; doi:10.3390/ijms26136137)
Supplement: Supplementary file 1 [file ijms-26-06137-s001.zip › Supplementary File S10b Array 12 Graphs.pdf]

Supplementary File S10. Protein expression of Array 12 targets as function of *Fmr1* genotype and AIN-93G diets. Mice on AIN-93G/cas (colored pink) included n=5 $Fmr1^{HET}$ female, n=8 $Fmr1^{KO}$ female, n=4 WT male and n=9 $Fmr1^{KO}$ male. Mice on AIN-93G/soy (colored green) included n=9 $Fmr1^{HET}$ female, n=8 $Fmr1^{KO}$ female, n=11 WT male and n=8 $Fmr1^{KO}$ male. The average concentration in cortex, hippocampus, hypothalamus and plasma in pg/mL was plotted versus genotype. Statistics were determined by 2-way ANOVA and Tukey's multiple comparison tests denoted by $p < 0.05$ (*), $p < 0.01$ (**), $p < 0.001$ (***) and $p < 0.0001$ (****).

Cortex

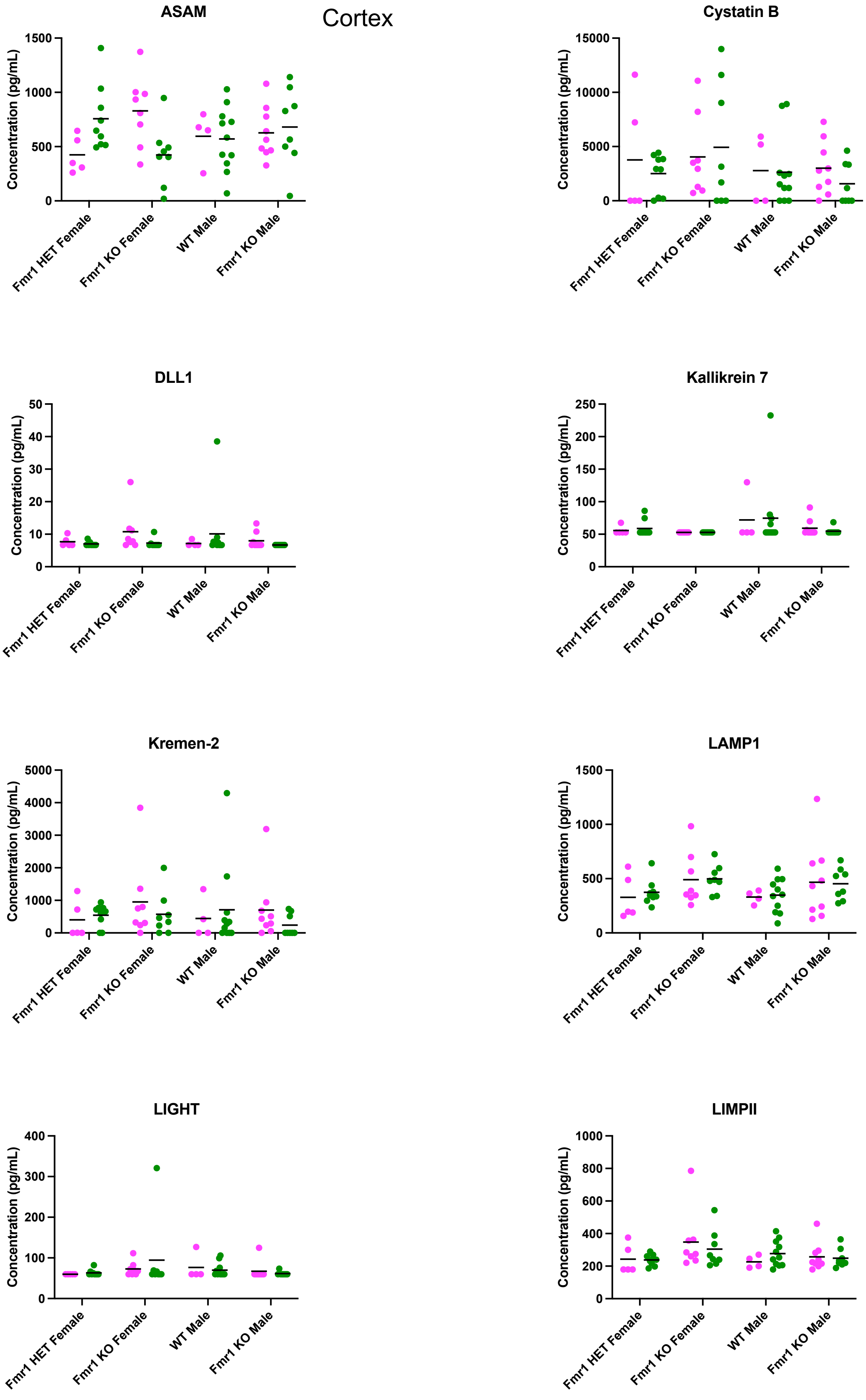

ASAM
Cystatin B
DLL1
Kallikrein 7
Kremen-2
LAMP1
LIGHT
LIMPII
Concentration (pg/mL)
Fmr1 HET Female
Fmr1 KO Female
WT Male
Fmr1 KO Male

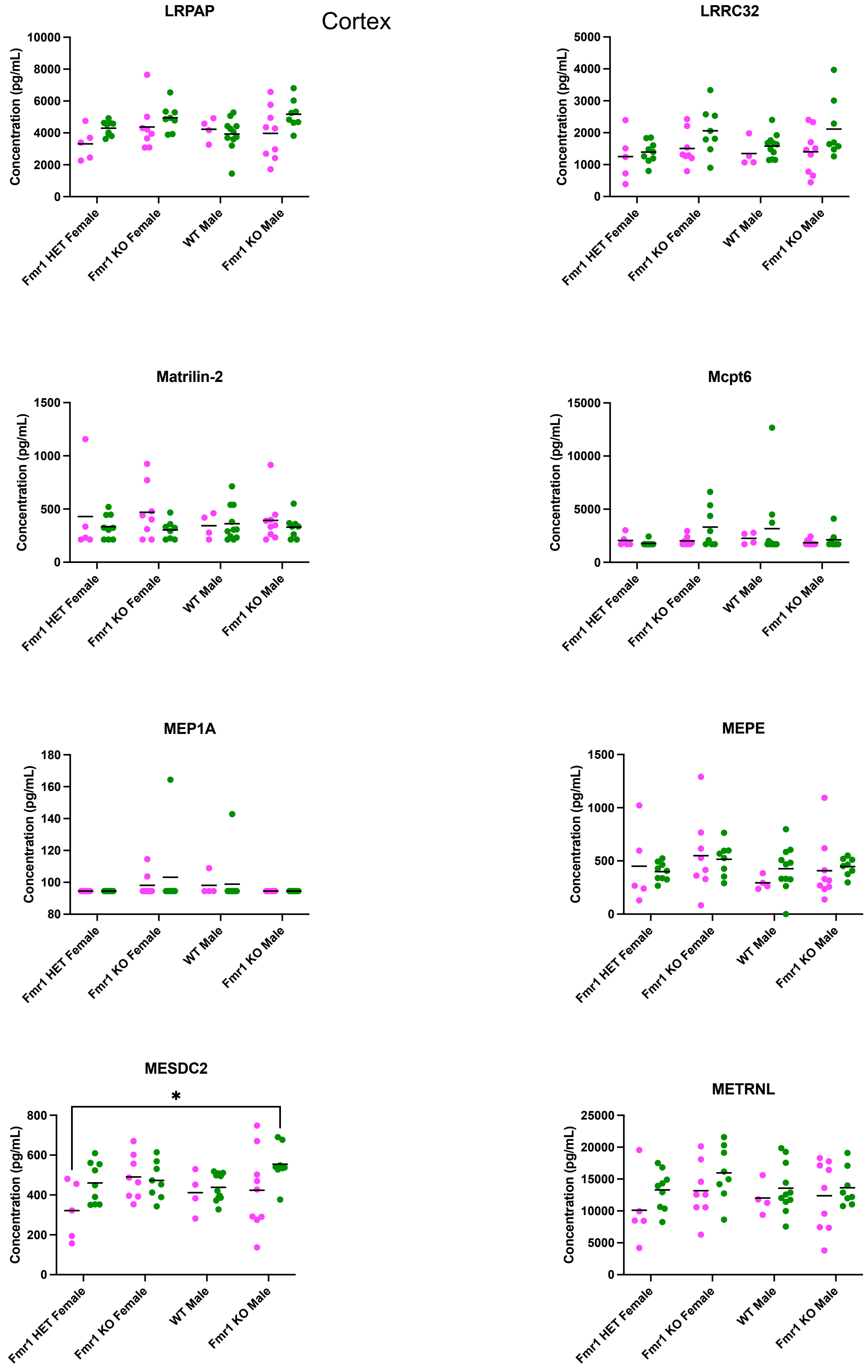
Cortex
LRPAP
LRRC32
Matrilin-2
Mcpt6
MEP1A
MEPE
MESDC2
METRNL
Concentration (pg/mL)
0
2000
4000
6000
8000
10000
1000
3000
5000
500
1500
15000
80
100
120
140
160
180
200
400
600
800
20000
25000
*
Fmr1 HET Female
Fmr1 KO Female
WT Male
Fmr1 KO Male

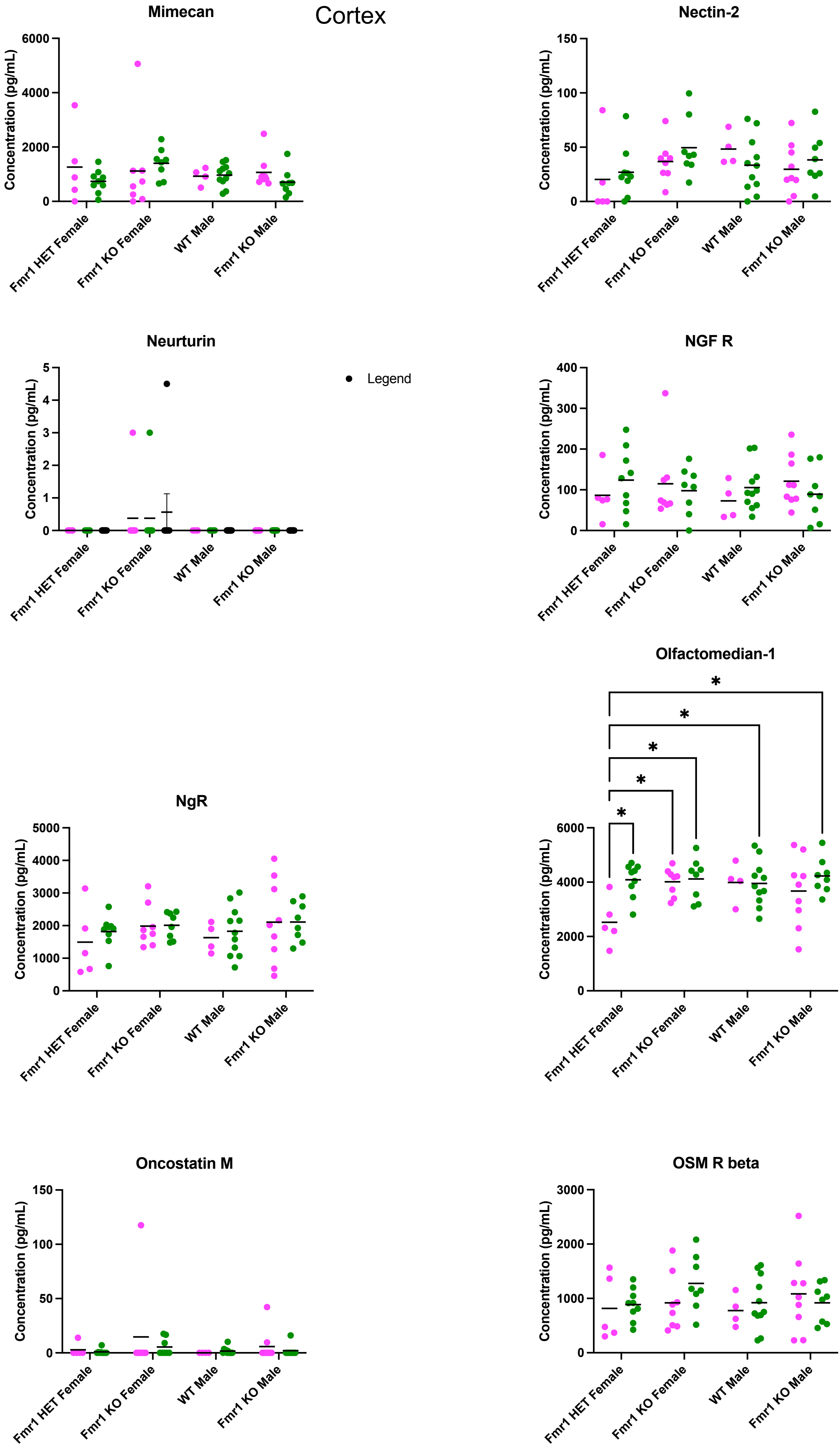
Cortex
Mimecan
Nectin-2
Neurturin
Legend
NGF R
Olfactomedian-1
NgR
Oncostatin M
OSM R beta
Concentration (pg/mL)
Fmr1 HET Female
Fmr1 KO Female
WT Male
Fmr1 KO Male
*
*
*
*
*

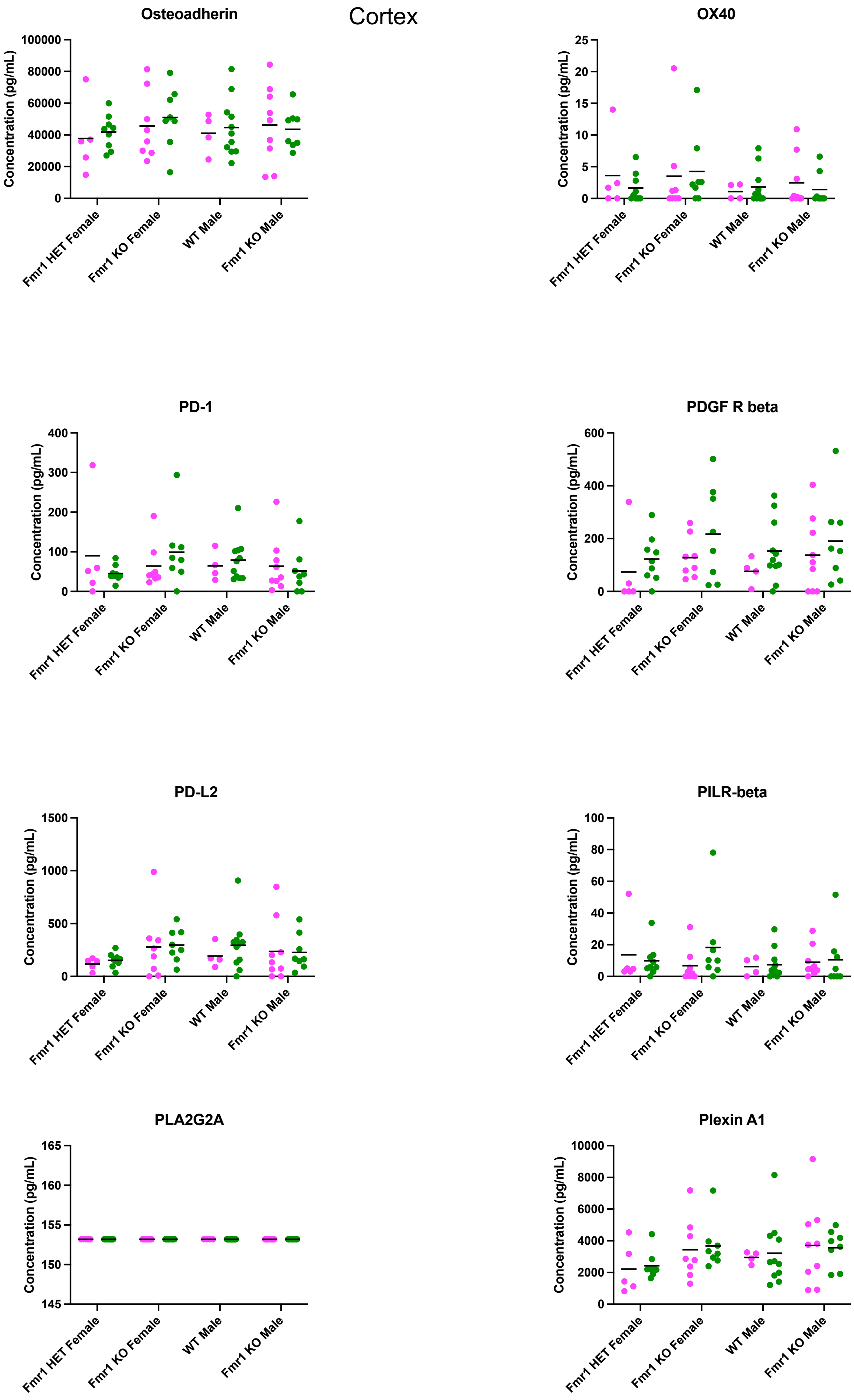
Cortex
Osteoadherin
OX40
PD-1
PDGF R beta
PD-L2
PILR-beta
PLA2G2A
Plexin A1
Concentration (pg/mL)
Fmr1 HET Female
Fmr1 KO Female
WT Male
Fmr1 KO Male

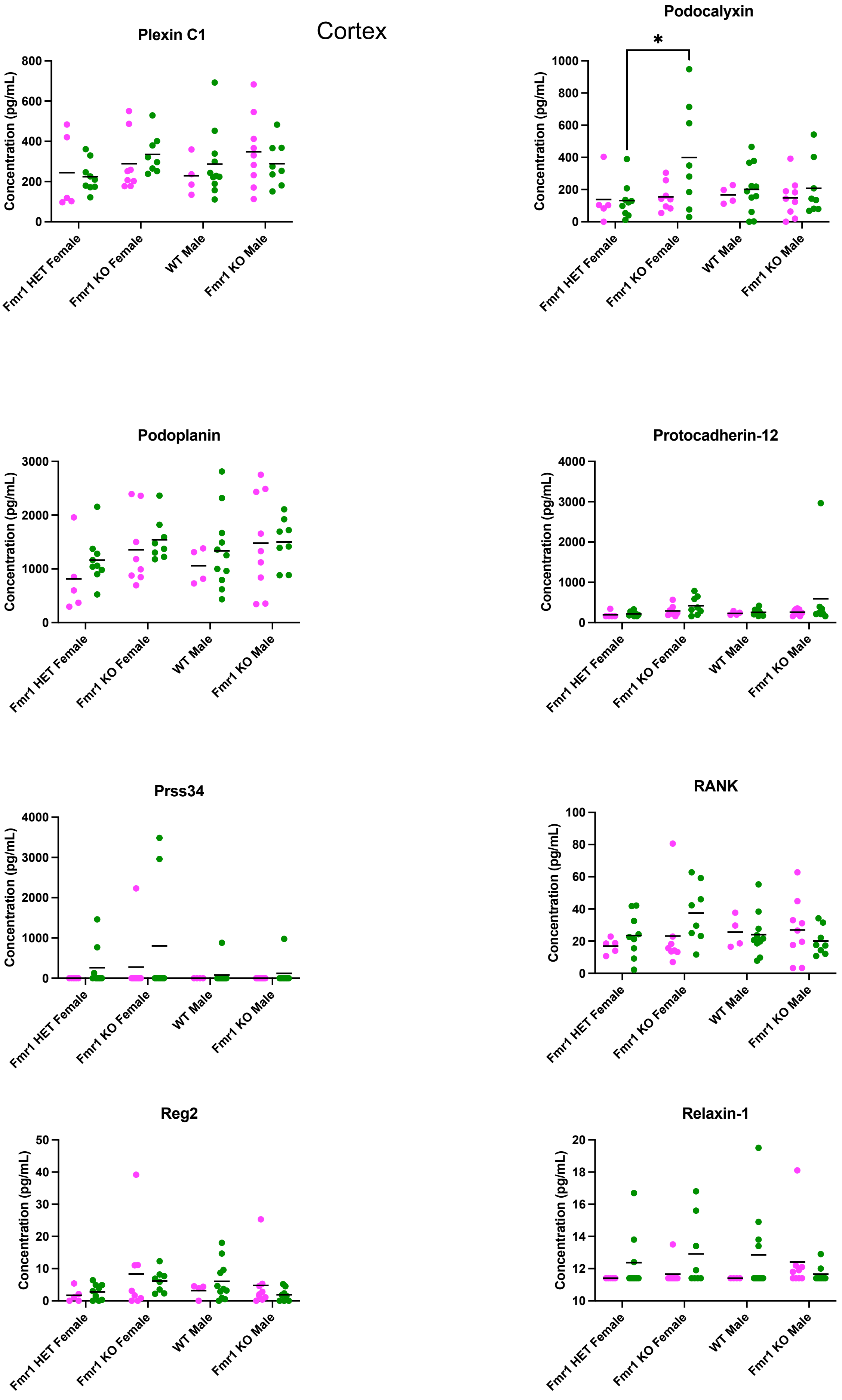

Cortex
Plexin C1
Podocalyxin
*
Podoplanin
Protocadherin-12
Prss34
RANK
Reg2
Relaxin-1
Concentration (pg/mL)
Fmr1 HET Female
Fmr1 KO Female
WT Male
Fmr1 KO Male

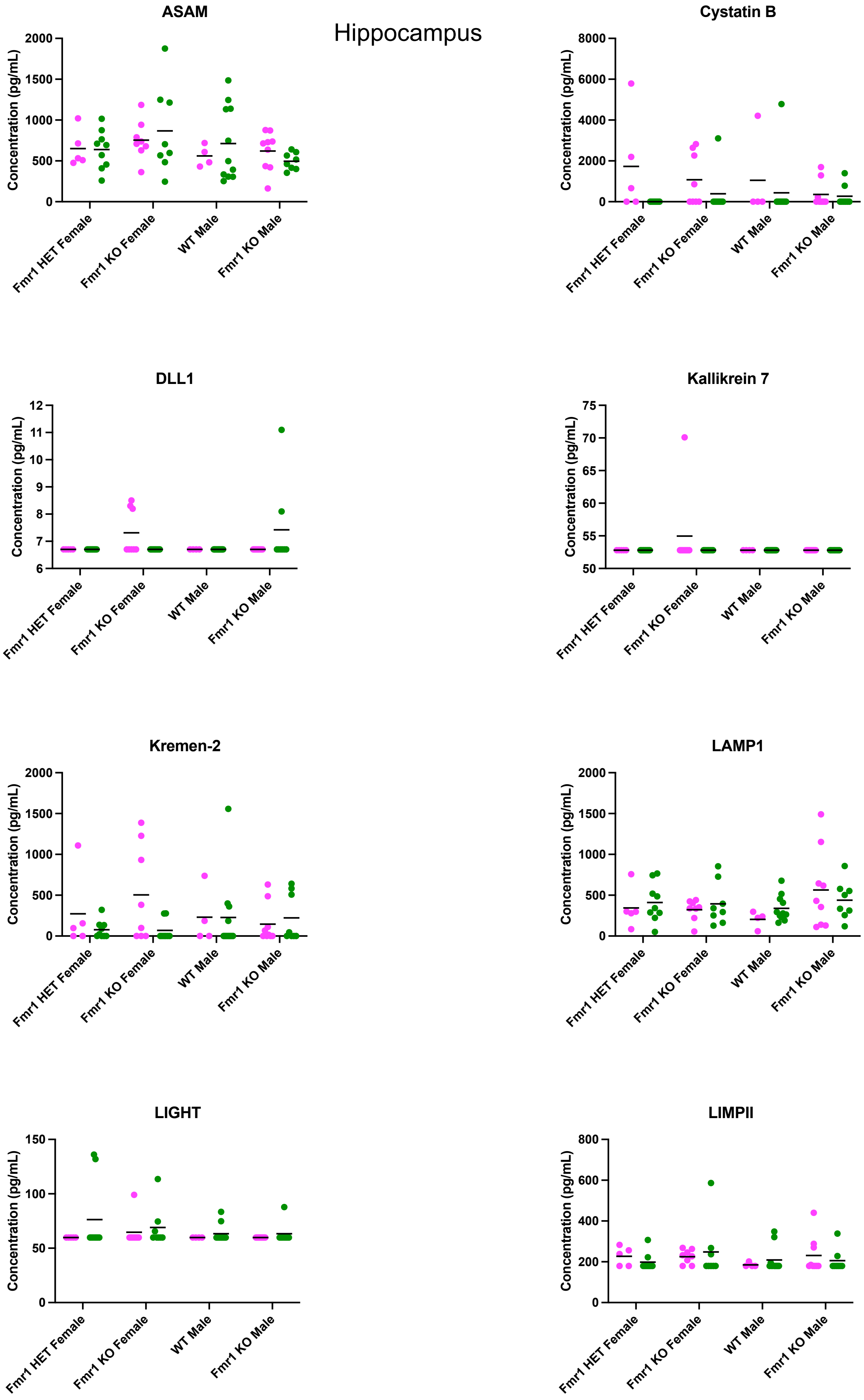
Hippocampus
ASAM
Cystatin B
DLL1
Kallikrein 7
Kremen-2
LAMP1
LIGHT
LIMPII
Concentration (pg/mL)
2000
1500
1000
500
0
8000
6000
4000
2000
12
11
10
9
8
7
6
75
70
65
60
55
50
150
100
50
800
600
400
200
Fmr1 HET Female
Fmr1 KO Female
WT Male
Fmr1 KO Male

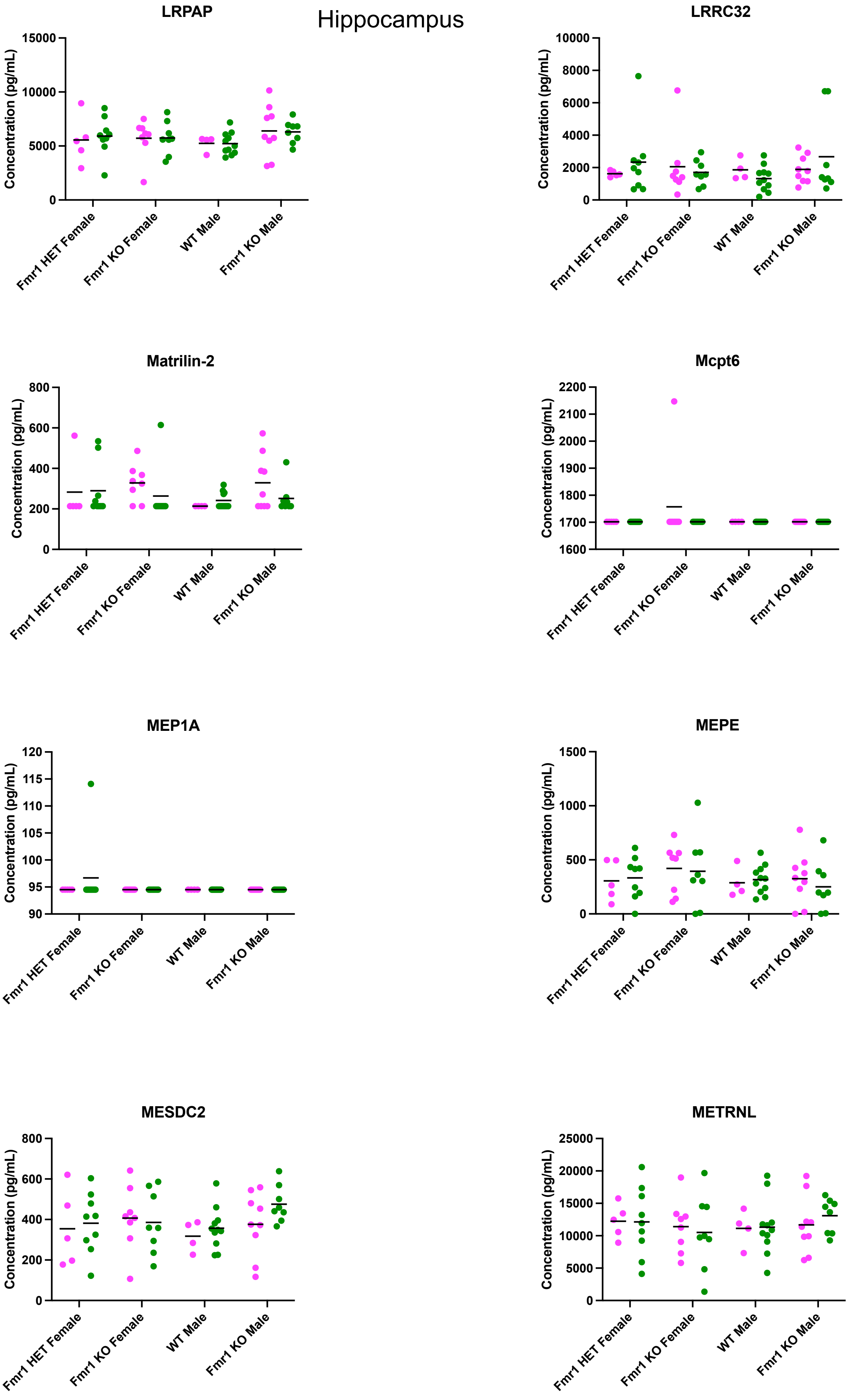
Hippocampus
LRPAP
LRRC32
Matrilin-2
Mcpt6
MEP1A
MEPE
MESDC2
METRNL
Concentration (pg/mL)
Fmr1 HET Female
Fmr1 KO Female
WT Male
Fmr1 KO Male

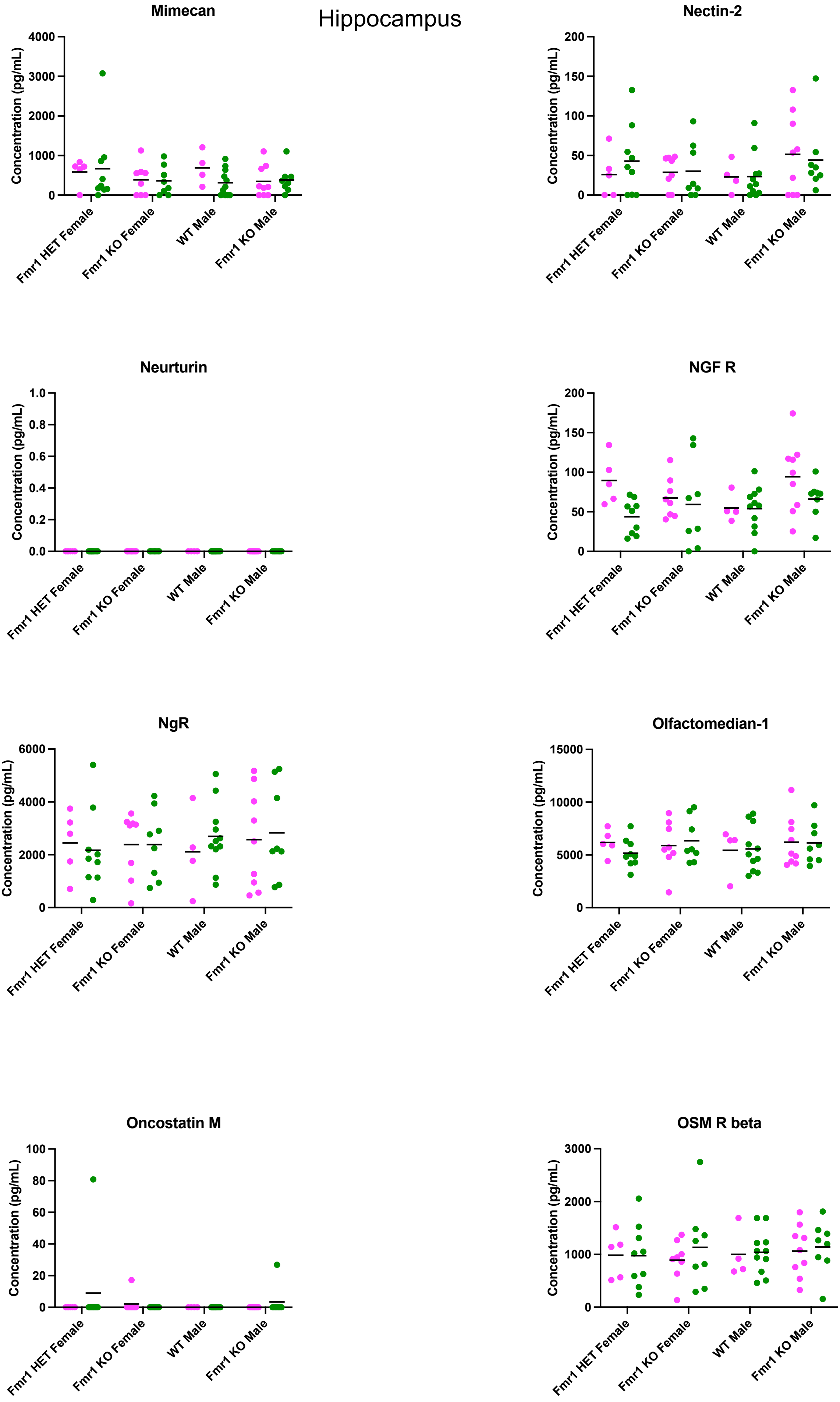
Hippocampus
Mimecan
Nectin-2
Neurturin
NGF R
NgR
Olfactomedian-1
Oncostatin M
OSM R beta
Concentration (pg/mL)
Fmr1 HET Female
Fmr1 KO Female
WT Male
Fmr1 KO Male

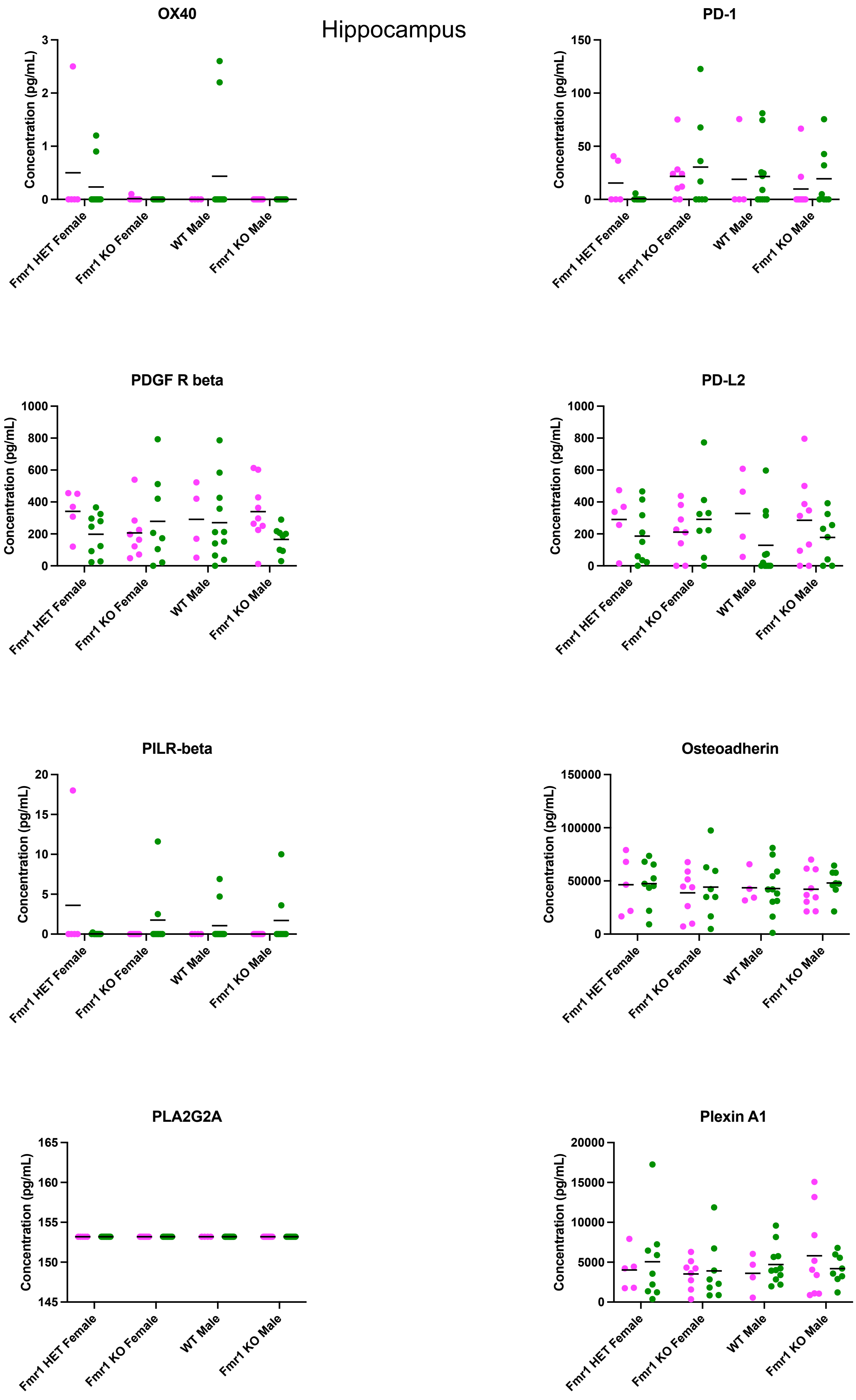

Hippocampus
OX40
PD-1
PDGF R beta
PD-L2
PILR-beta
Osteoadherin
PLA2G2A
Plexin A1
Concentration (pg/mL)
Fmr1 HET Female
Fmr1 KO Female
WT Male
Fmr1 KO Male

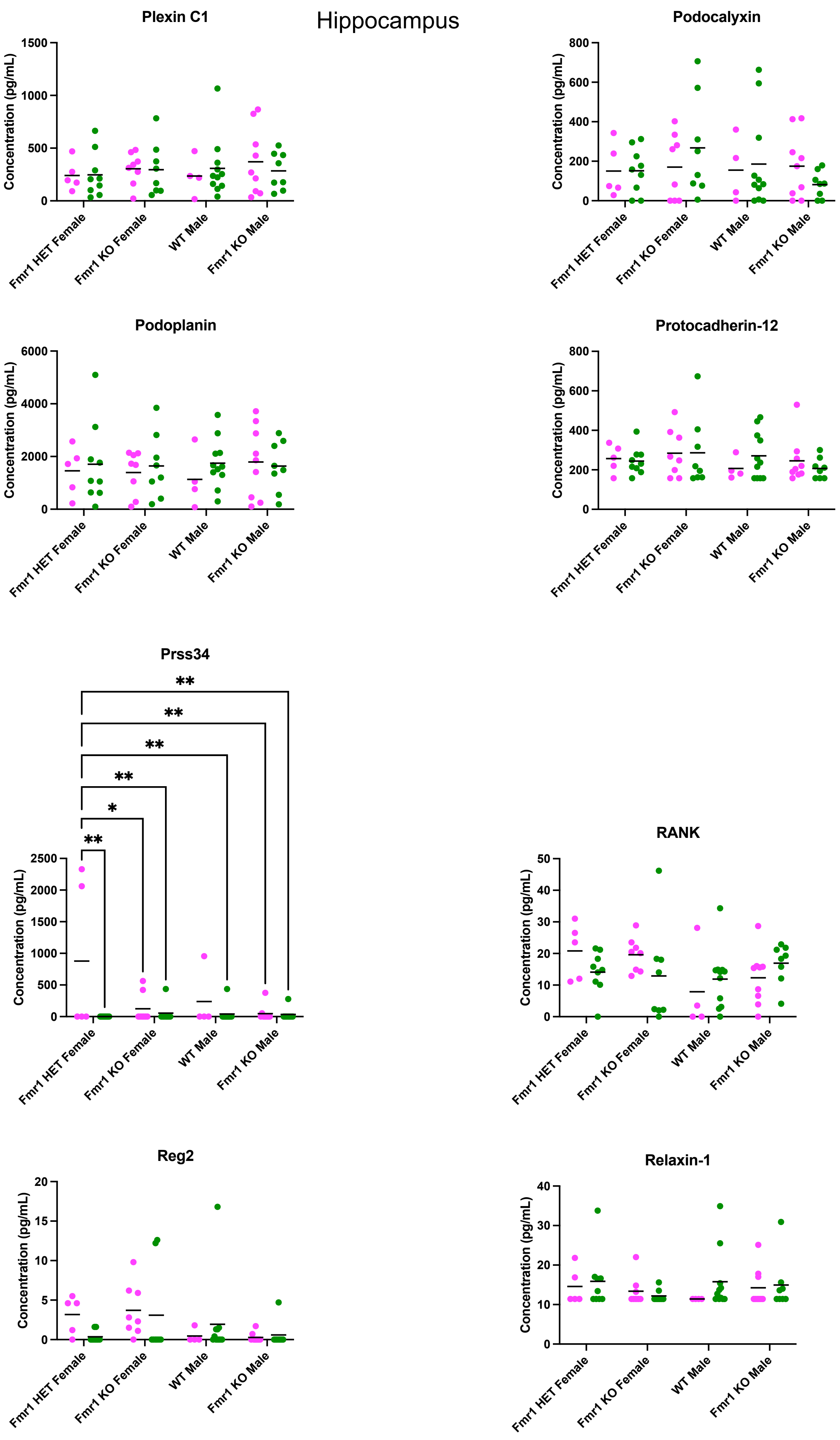
Hippocampus
Plexin C1
Podocalyxin
Podoplanin
Protocadherin-12
Prss34
RANK
Reg2
Relaxin-1
Concentration (pg/mL)
Fmr1 HET Female
Fmr1 KO Female
WT Male
Fmr1 KO Male
**
*

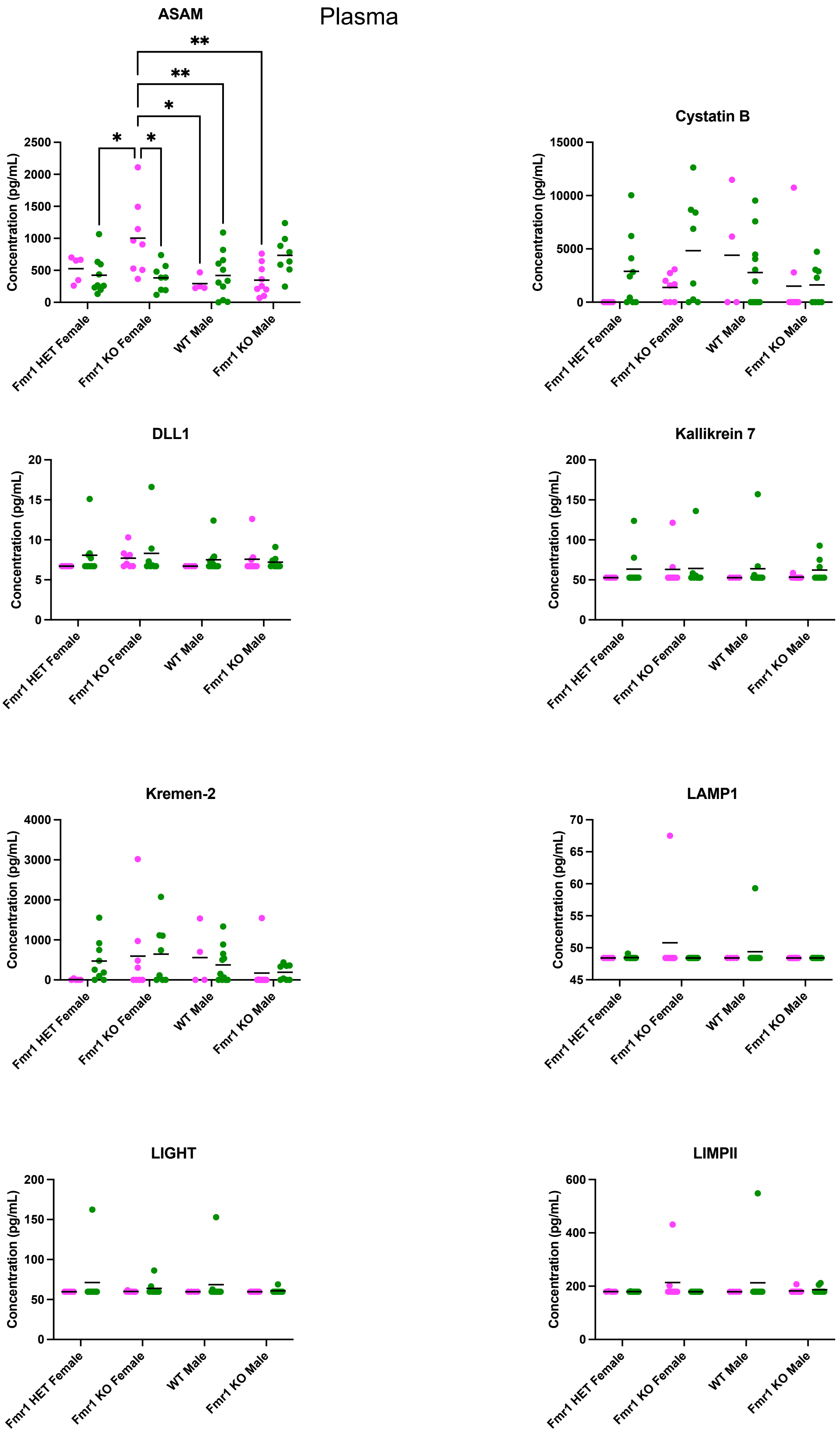
Plasma
ASAM
Cystatin B
DLL1
Kallikrein 7
Kremen-2
LAMP1
LIGHT
LIMPII
Concentration (pg/mL)
Fmr1 HET Female
Fmr1 KO Female
WT Male
Fmr1 KO Male
*
**

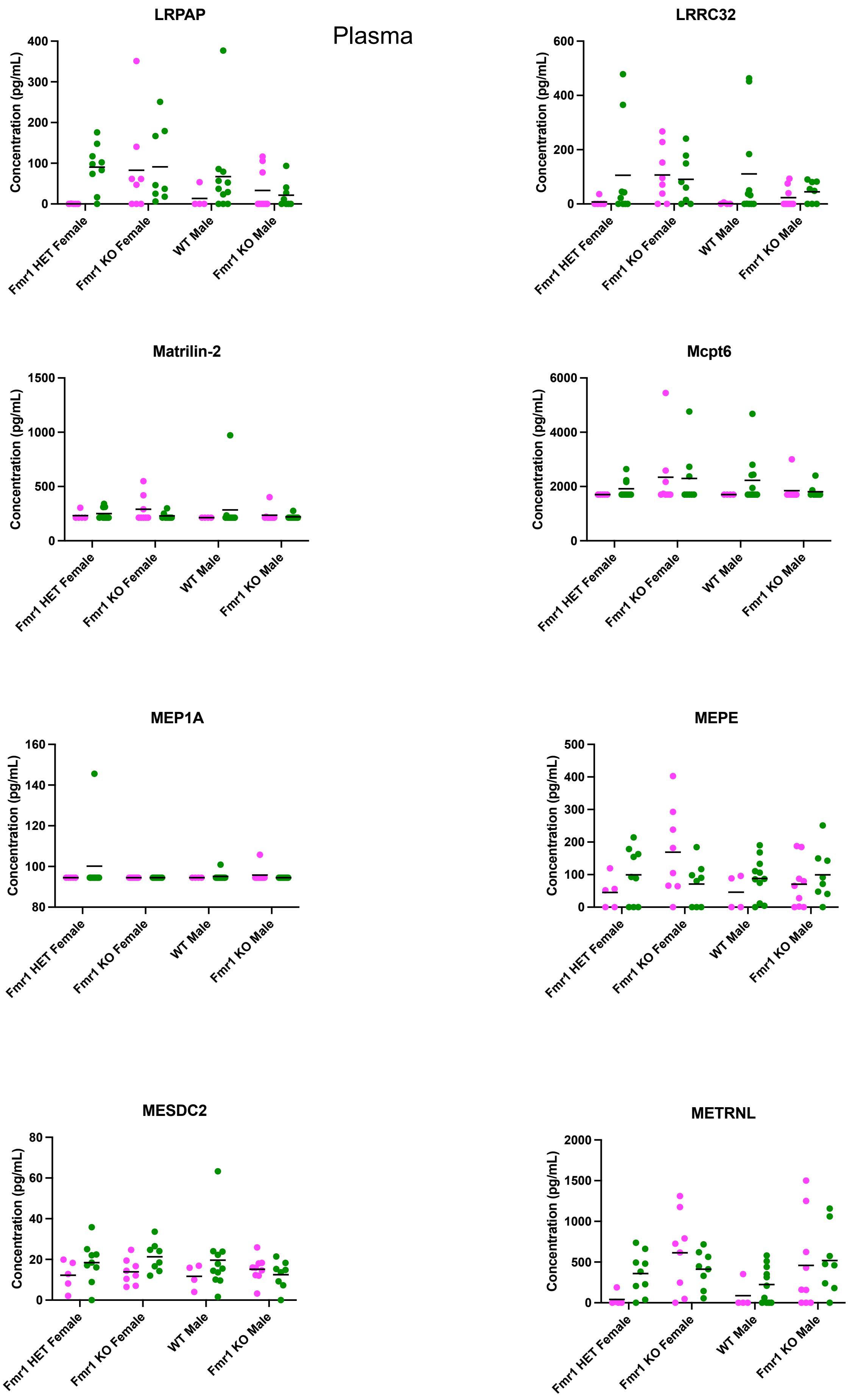

Plasma
LRPAP
LRRC32
Matrilin-2
Mcpt6
MEP1A
MEPE
MESDC2
METRNL
Concentration (pg/mL)
Fmr1 HET Female
Fmr1 KO Female
WT Male
Fmr1 KO Male

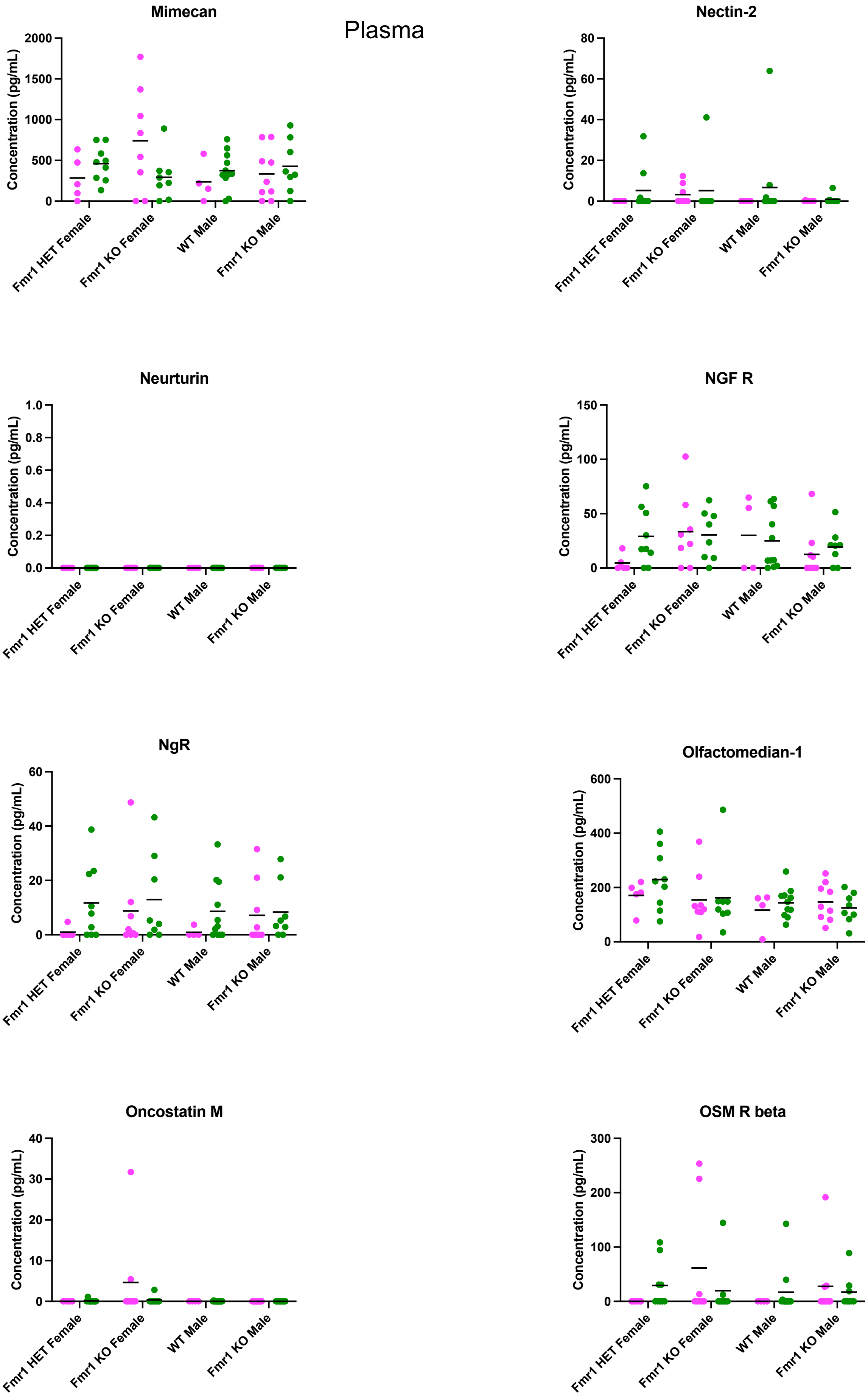
Plasma
Mimecan
Nectin-2
Neurturin
NGF R
NgR
Olfactomedian-1
Oncostatin M
OSM R beta
Concentration (pg/mL)
Fmr1 HET Female
Fmr1 KO Female
WT Male
Fmr1 KO Male

Plasma

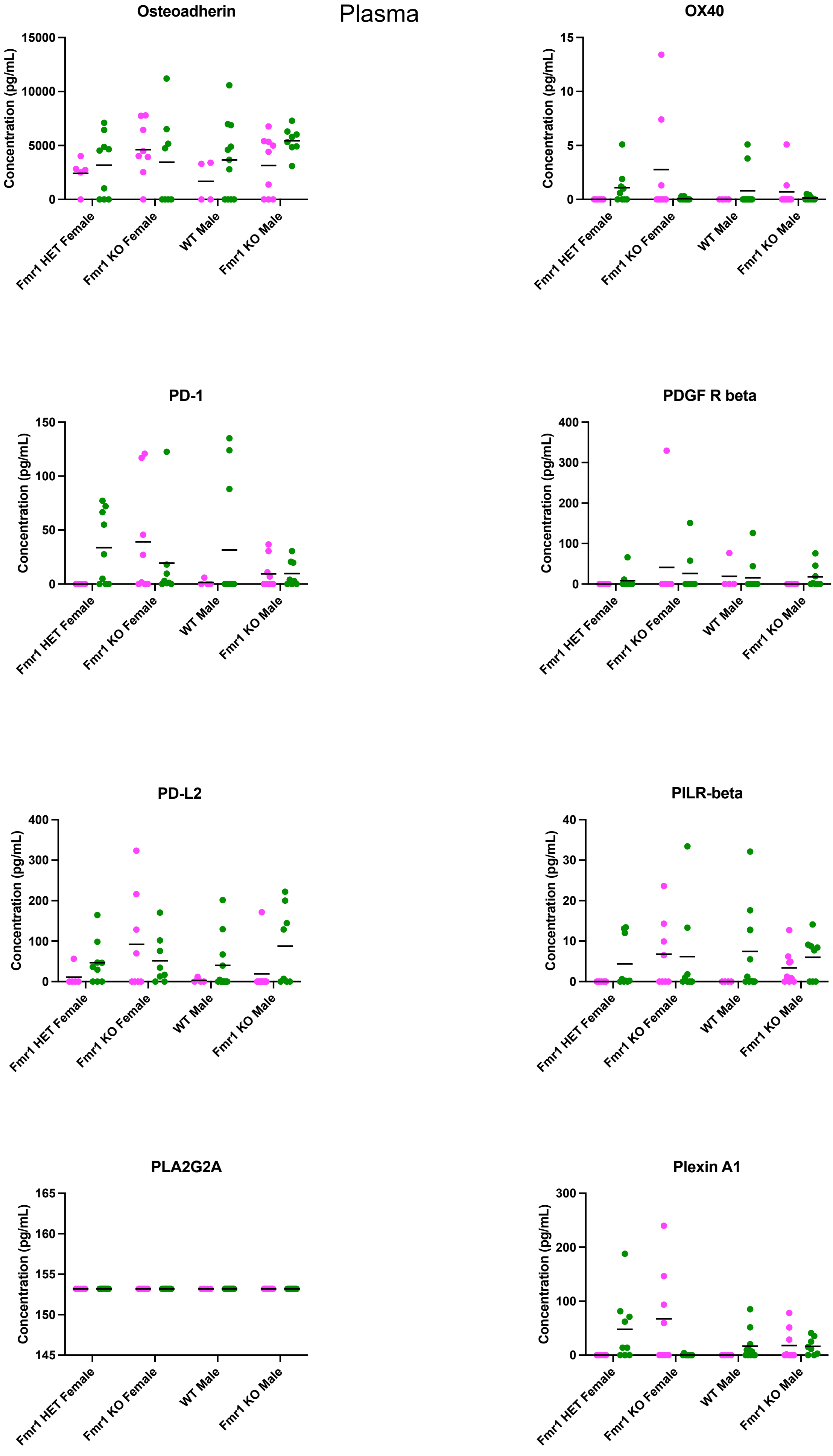
Osteoadherin
Concentration (pg/mL)
15000
10000
5000
0
Fmr1 HET Female
Fmr1 KO Female
WT Male
Fmr1 KO Male
OX40
15
10
5
0
PD-1
150
100
50
0
PDGF R beta
400
300
200
100
0
PD-L2
400
300
200
100
0
PILR-beta
40
30
20
10
0
PLA2G2A
165
160
155
150
145
Plexin A1
300
200
100
0

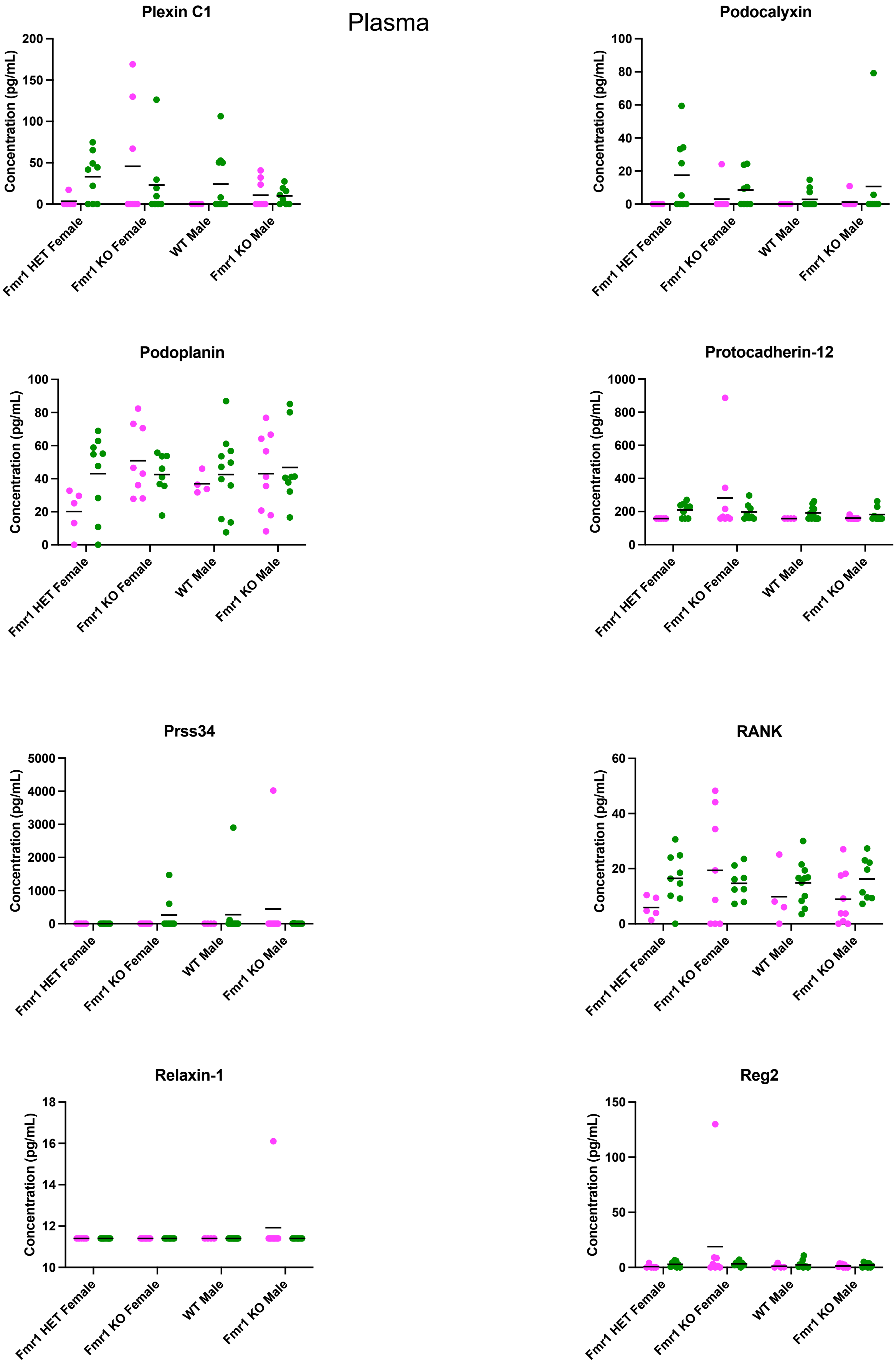
Plasma
Plexin C1
Podocalyxin
Podoplanin
Protocadherin-12
Prss34
RANK
Relaxin-1
Reg2
Concentration (pg/mL)
Fmr1 HET Female
Fmr1 KO Female
WT Male
Fmr1 KO Male
