# Supplementary material for: Soy Protein Isolate Affects Blood and Brain Biomarker Expression in a Mouse Model of Fragile X
Source: Int J Mol Sci. 2025 Jun 26;26(13):6137. doi: 10.3390/ijms26136137 (PMC12250412; doi:10.3390/ijms26136137)
Supplement: Supplementary file 1 [file ijms-26-06137-s001.zip › Supplementary File S11b Array 13 Graphs.pdf]

Supplementary File S11. Protein expression of Array 13 targets as function of *Fmr1* genotype and AIN-93G diets. Mice on AIN-93G/cas (colored pink) included n=5 *Fmr1*^{HET} female, n=8 *Fmr1*^{KO} female, n=4 WT male and n=9 *Fmr1*^{KO} male. Mice on AIN-93G/soy (colored green) included n=9 *Fmr1*^{HET} female, n=8 *Fmr1*^{KO} female, n=11 WT male and n=8 *Fmr1*^{KO} male. The average concentration in cortex, hippocampus, hypothalamus and plasma in pg/mL was plotted versus genotype. Statistics were determined by 2-way ANOVA and Tukey's multiple comparison tests denoted by $p < 0.05$ (*), $p < 0.01$ (**), $p < 0.001$ (***) and $p < 0.0001$ (****).

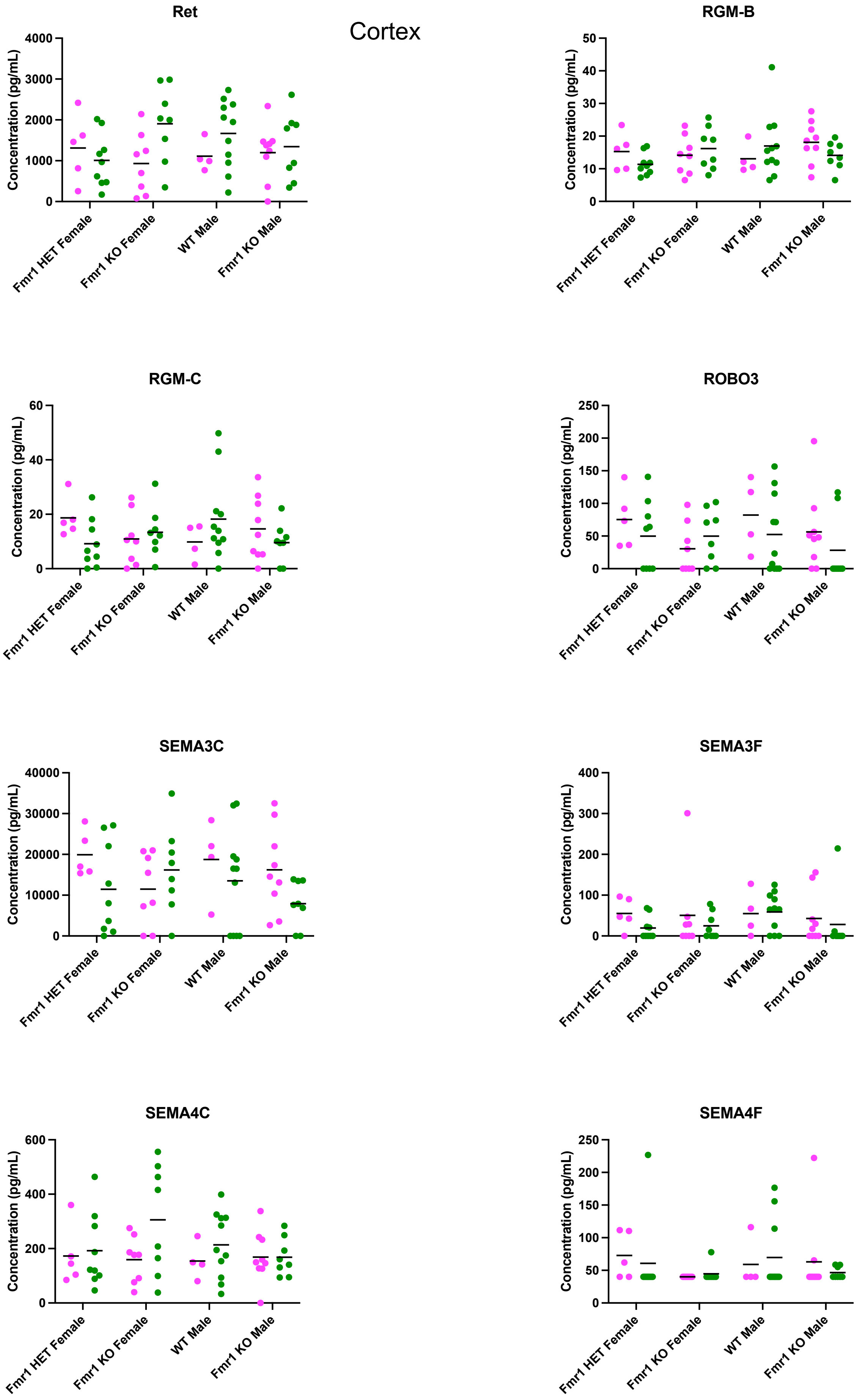

Cortex
Ret
RGM-B
RGM-C
ROBO3
SEMA3C
SEMA3F
SEMA4C
SEMA4F
Concentration (pg/mL)
Fmr1 HET Female
Fmr1 KO Female
WT Male
Fmr1 KO Male

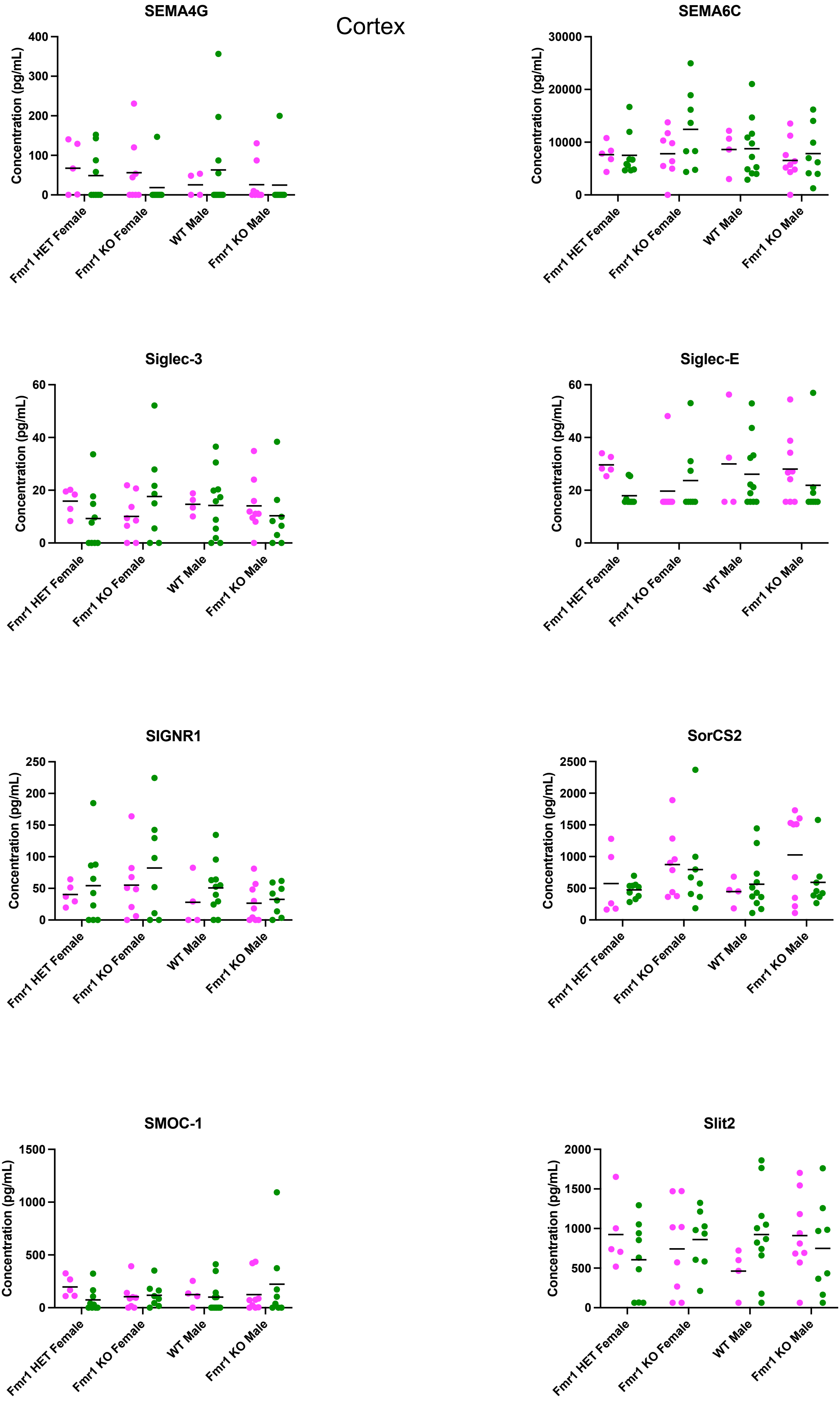
Cortex
SEMA4G
SEMA6C
Siglec-3
Siglec-E
SIGNR1
SorCS2
SMOC-1
Slit2
Concentration (pg/mL)
Fmr1 HET Female
Fmr1 KO Female
WT Male
Fmr1 KO Male
0
100
200
300
400
10000
20000
30000
20
40
60
50
150
250
500
1000
1500
2000
2500

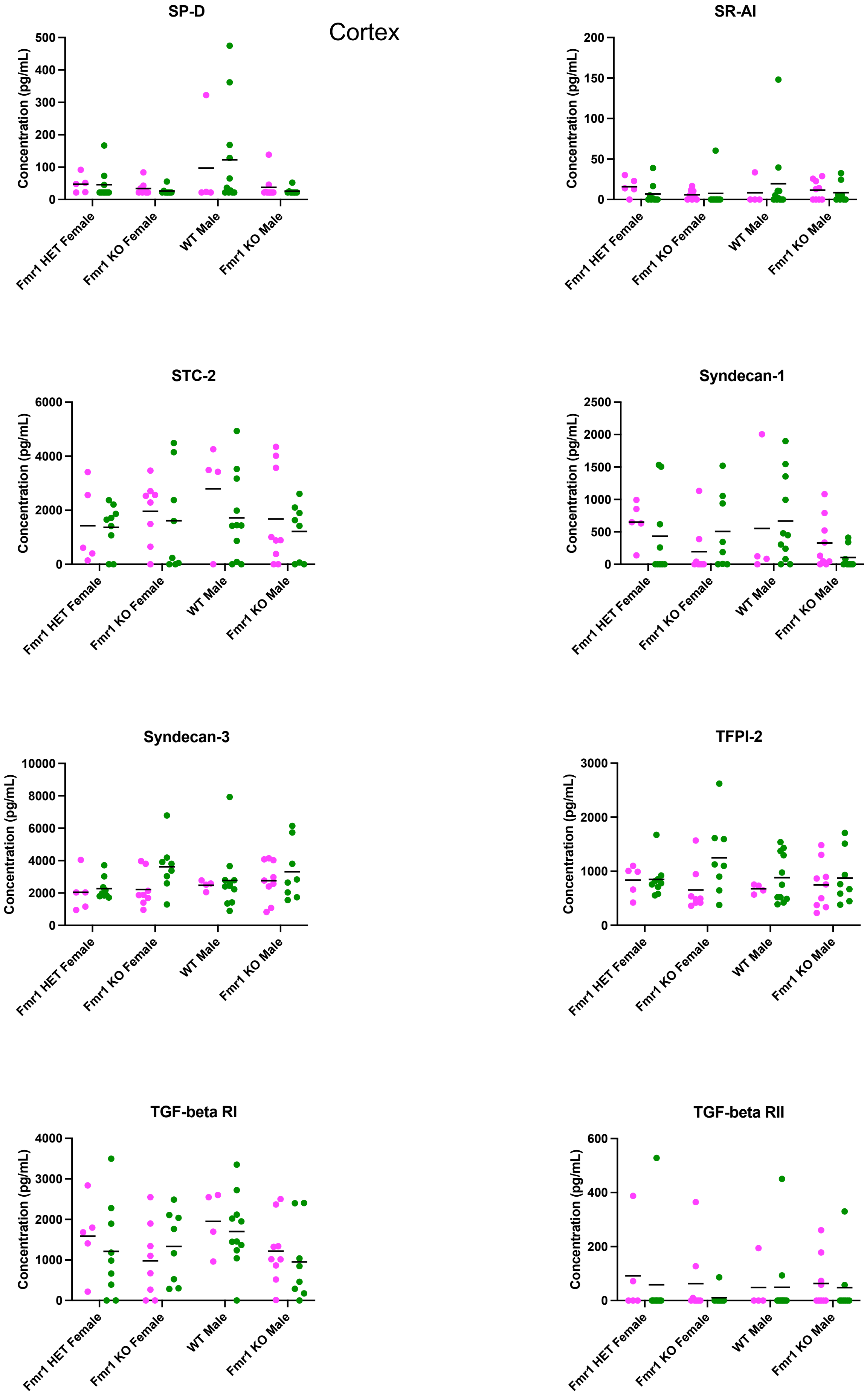
Cortex
SP-D
SR-AI
STC-2
Syndecan-1
Syndecan-3
TFPI-2
TGF-beta RI
TGF-beta RII
Concentration (pg/mL)
Fmr1 HET Female
Fmr1 KO Female
WT Male
Fmr1 KO Male
0
100
200
300
400
500
50
150
2000
4000
6000
500
1000
1500
2000
2500
8000
10000
3000
600

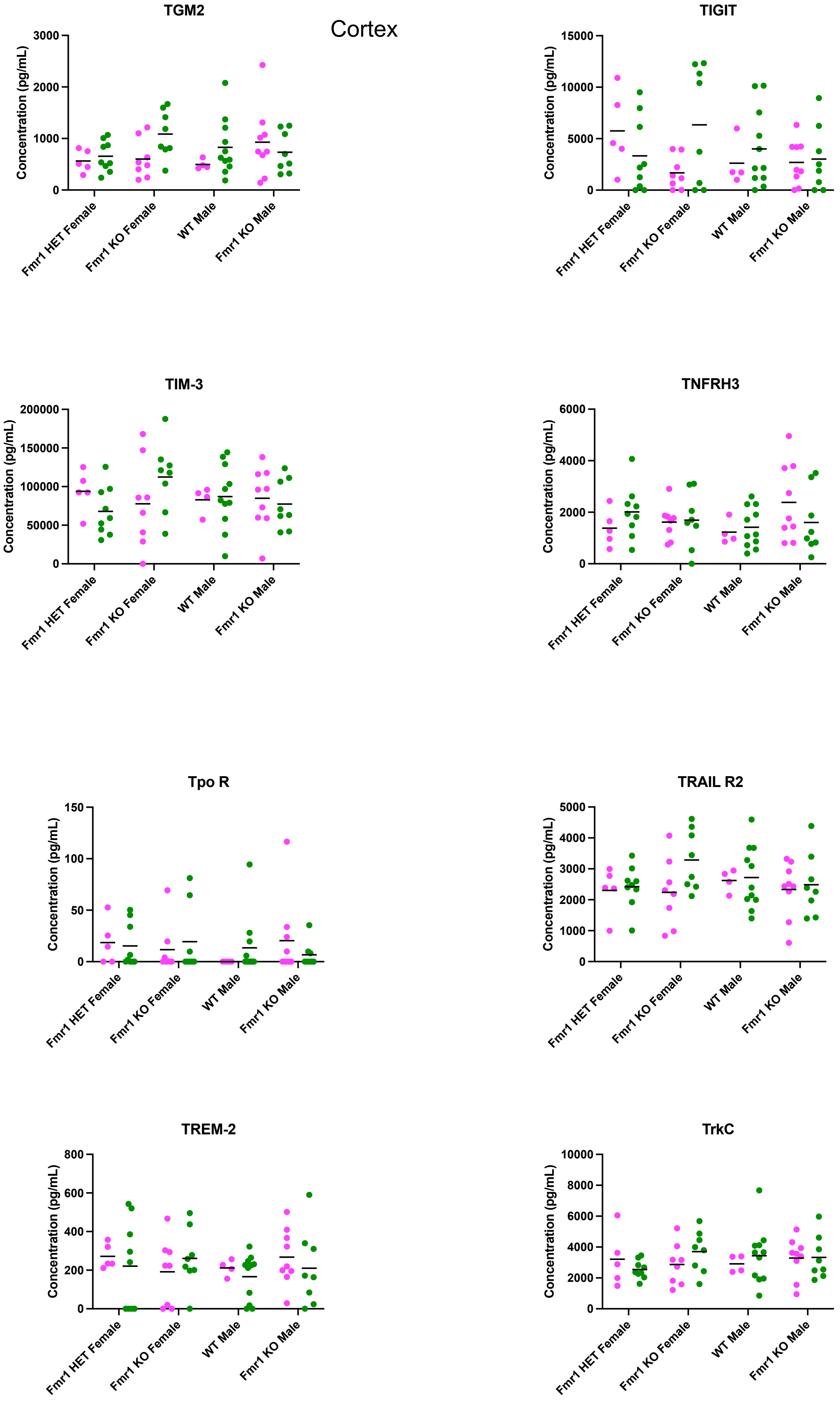

Cortex
TGM2
TIGIT
TIM-3
TNFRH3
Tpo R
TRAIL R2
TREM-2
TrkC
Concentration (pg/mL)
Fmr1 HET Female
Fmr1 KO Female
WT Male
Fmr1 KO Male
0
1000
2000
3000
5000
10000
15000
50000
100000
150000
200000
4000
6000
50
100
150
200
400
600
800
8000

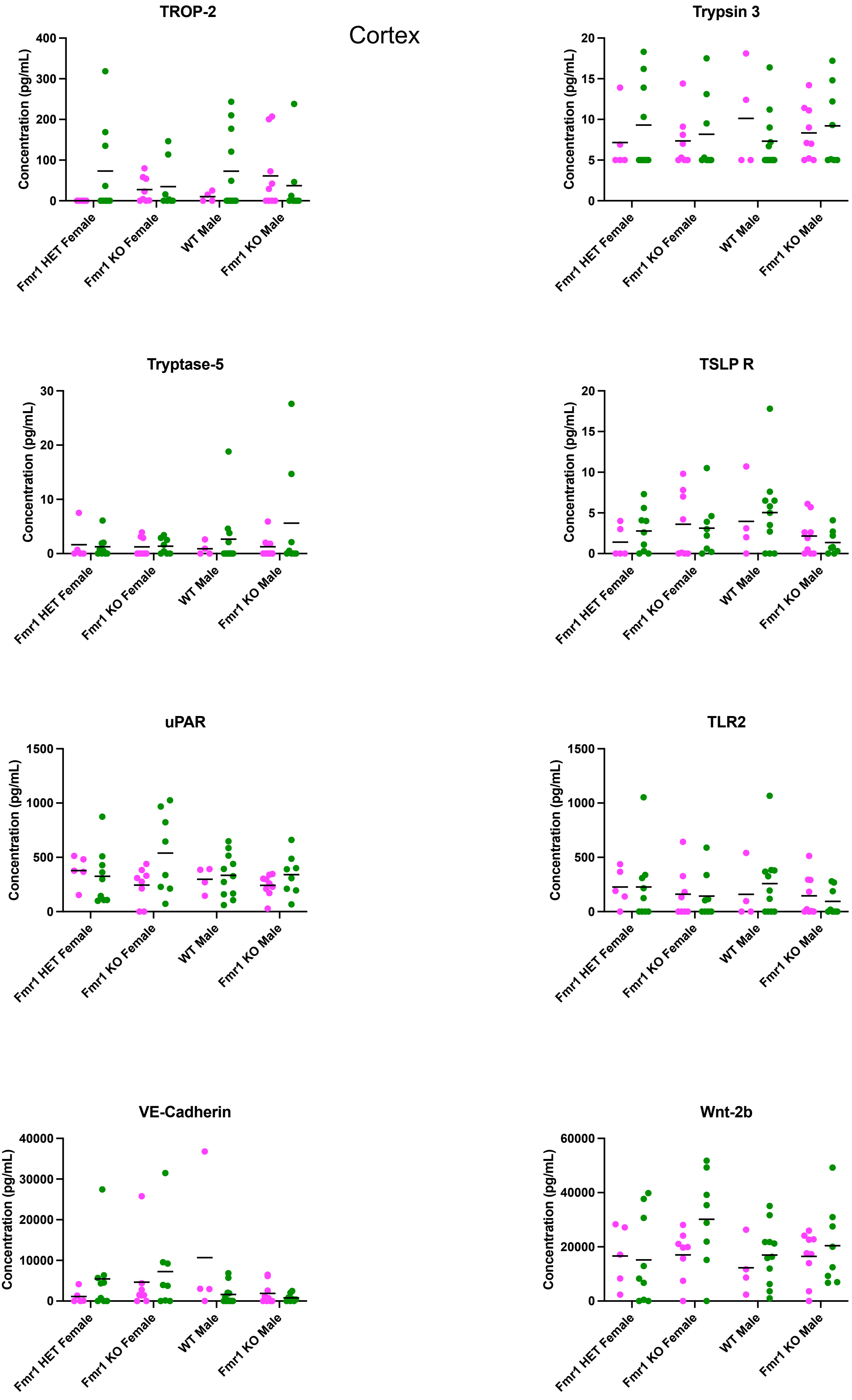

Cortex
TROP-2
Trypsin 3
Tryptase-5
TSLP R
uPAR
TLR2
VE-Cadherin
Wnt-2b
Concentration (pg/mL)
0
100
200
300
400
5
10
15
20
30
500
1000
1500
10000
20000
30000
40000
60000
Fmr1 HET Female
Fmr1 KO Female
WT Male
Fmr1 KO Male

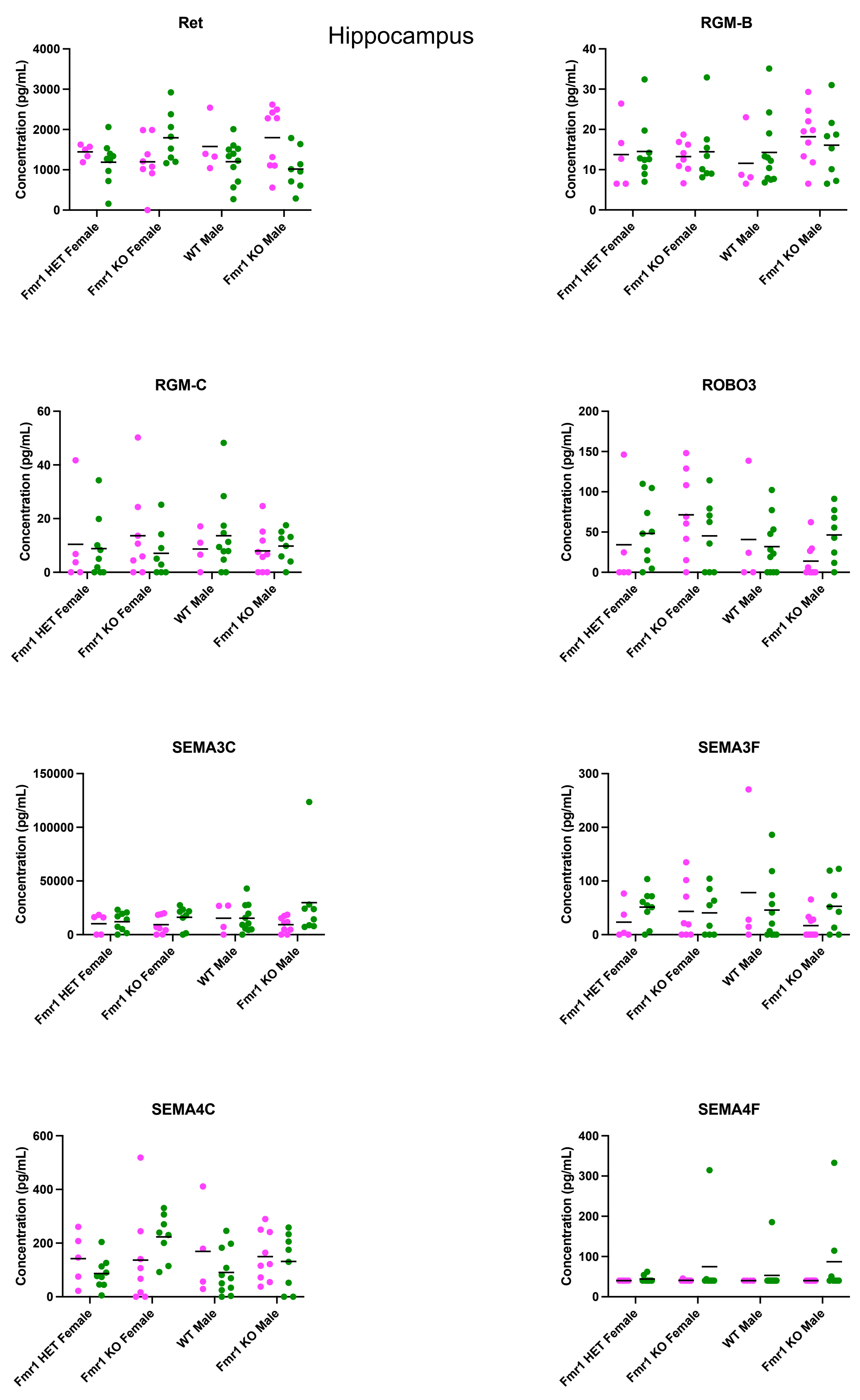
Hippocampus
Ret
Concentration (pg/mL)
0
1000
2000
3000
4000
Fmr1 HET Female
Fmr1 KO Female
WT Male
Fmr1 KO Male
RGM-B
Concentration (pg/mL)
0
10
20
30
40
Fmr1 HET Female
Fmr1 KO Female
WT Male
Fmr1 KO Male
RGM-C
Concentration (pg/mL)
0
20
40
60
Fmr1 HET Female
Fmr1 KO Female
WT Male
Fmr1 KO Male
ROBO3
Concentration (pg/mL)
0
50
100
150
200
Fmr1 HET Female
Fmr1 KO Female
WT Male
Fmr1 KO Male
SEMA3C
Concentration (pg/mL)
0
50000
100000
150000
Fmr1 HET Female
Fmr1 KO Female
WT Male
Fmr1 KO Male
SEMA3F
Concentration (pg/mL)
0
100
200
300
Fmr1 HET Female
Fmr1 KO Female
WT Male
Fmr1 KO Male
SEMA4C
Concentration (pg/mL)
0
200
400
600
Fmr1 HET Female
Fmr1 KO Female
WT Male
Fmr1 KO Male
SEMA4F
Concentration (pg/mL)
0
100
200
300
400
Fmr1 HET Female
Fmr1 KO Female
WT Male
Fmr1 KO Male

Hippocampus

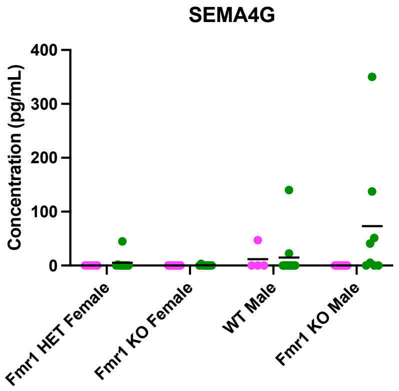

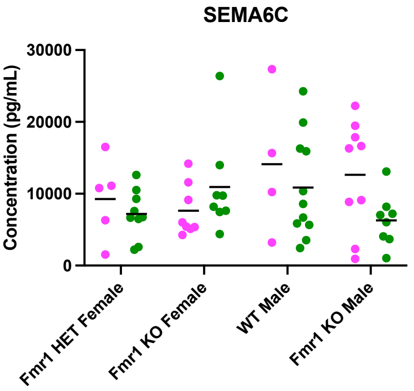

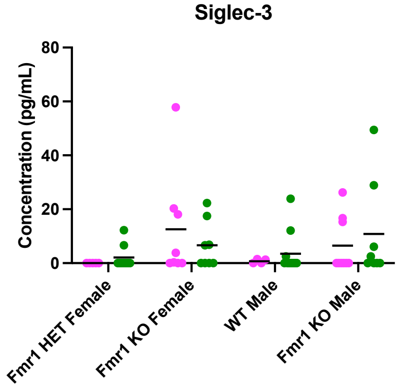

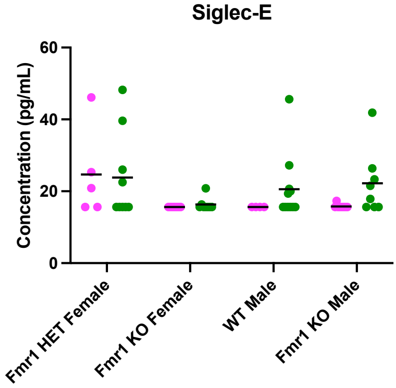

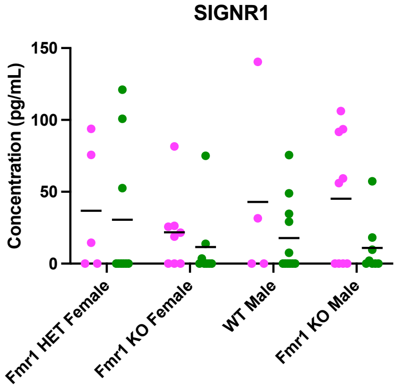

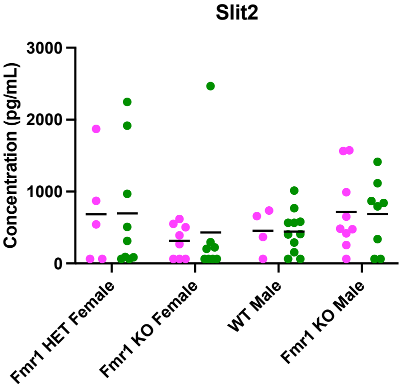

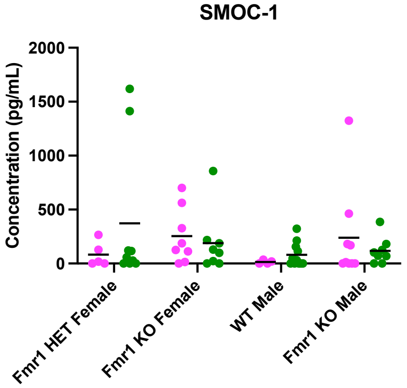

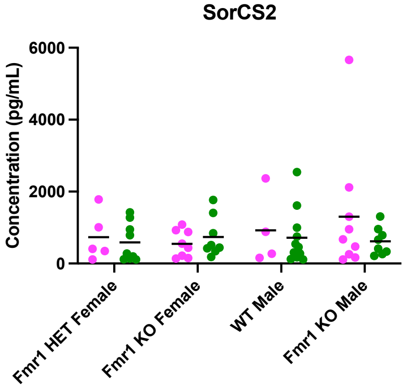

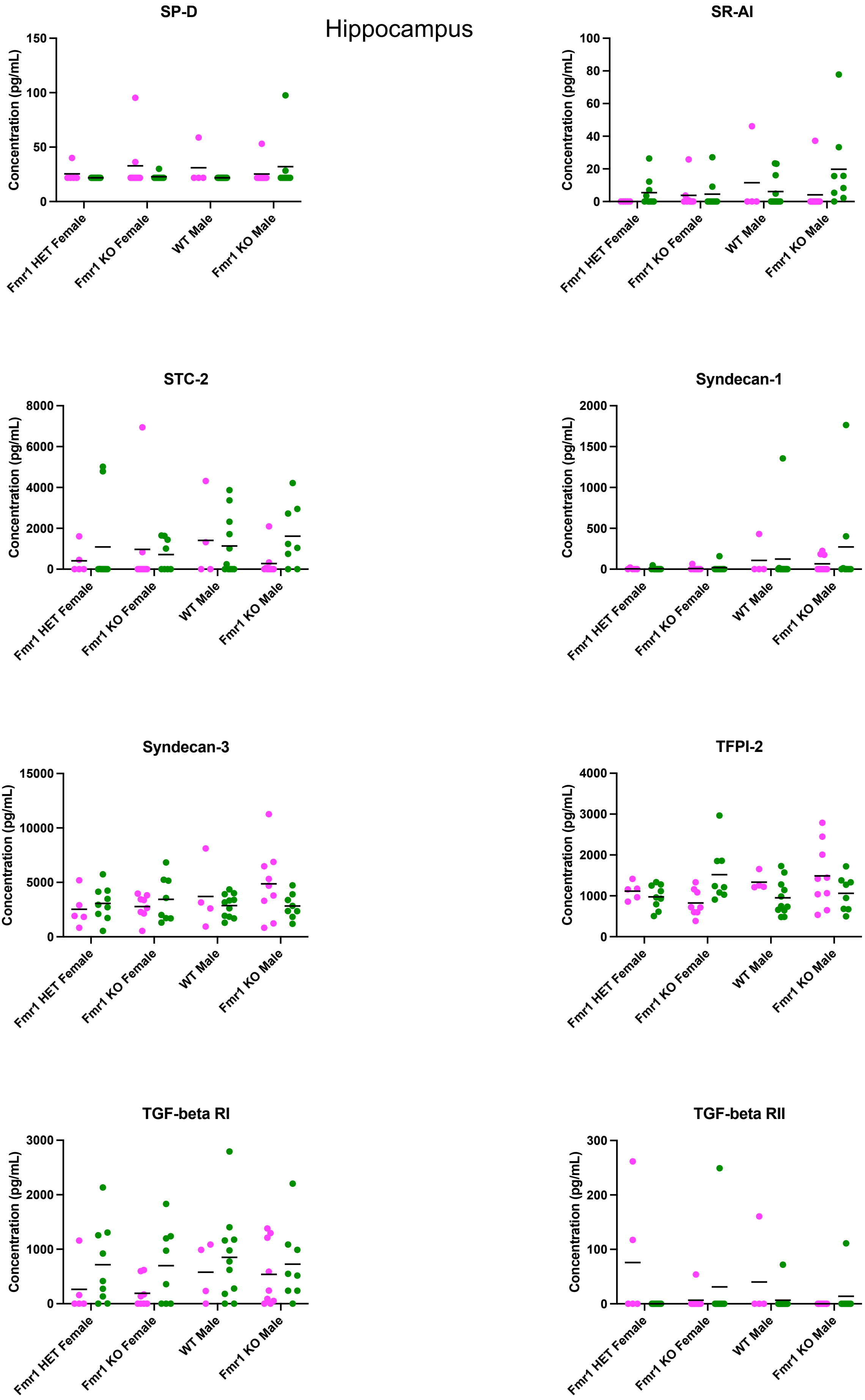

Hippocampus
SP-D
SR-AI
STC-2
Syndecan-1
Syndecan-3
TFPI-2
TGF-beta RI
TGF-beta RII
Concentration (pg/mL)
Fmr1 HET Female
Fmr1 KO Female
WT Male
Fmr1 KO Male

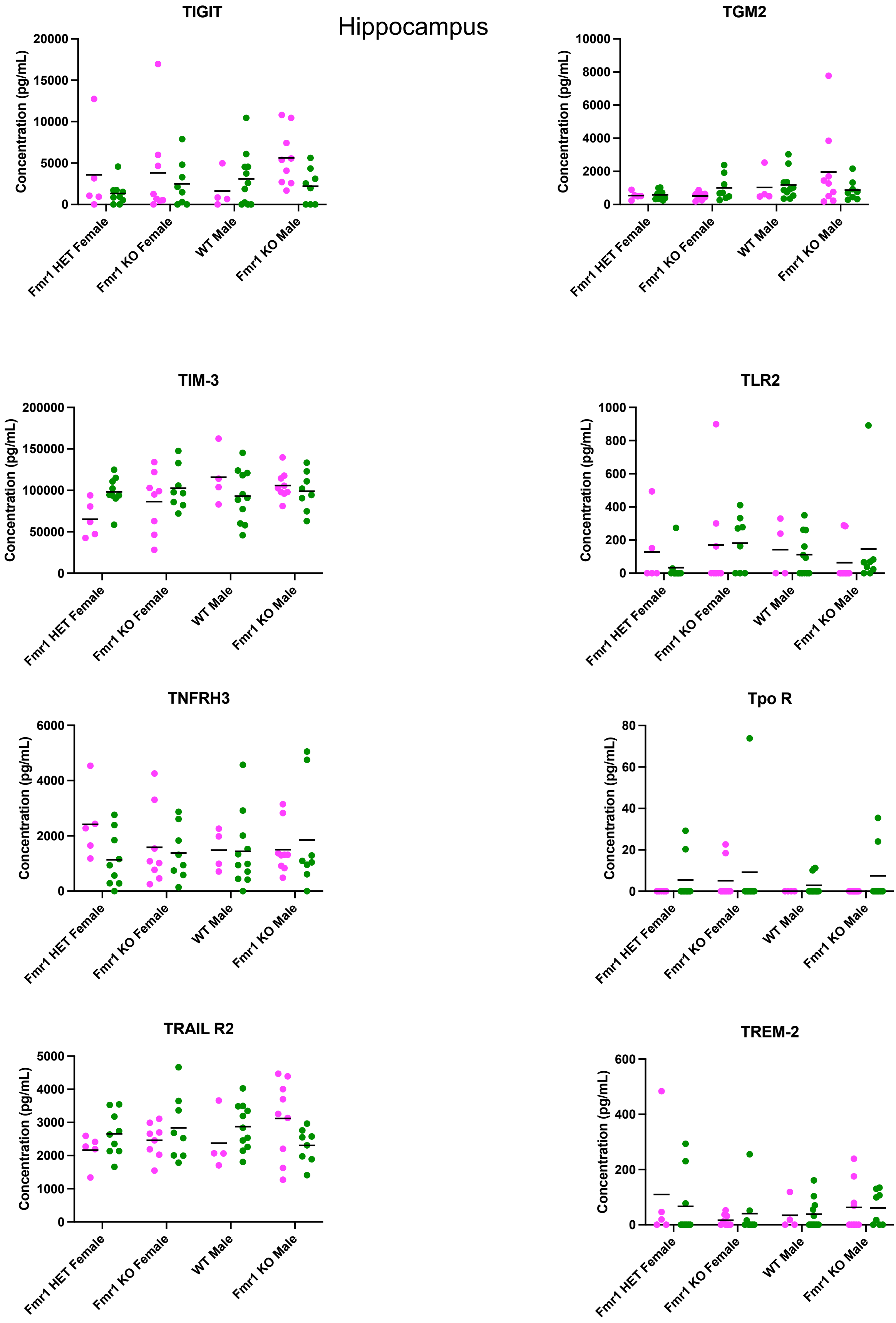

Hippocampus
TIGIT
TGM2
TIM-3
TLR2
TNFRH3
Tpo R
TRAIL R2
TREM-2
Concentration (pg/mL)
0
5000
10000
15000
20000
2000
4000
6000
8000
10000
50000
100000
150000
200000
200
400
600
800
1000
20
40
60
80
3000
5000
Fmr1 HET Female
Fmr1 KO Female
WT Male
Fmr1 KO Male

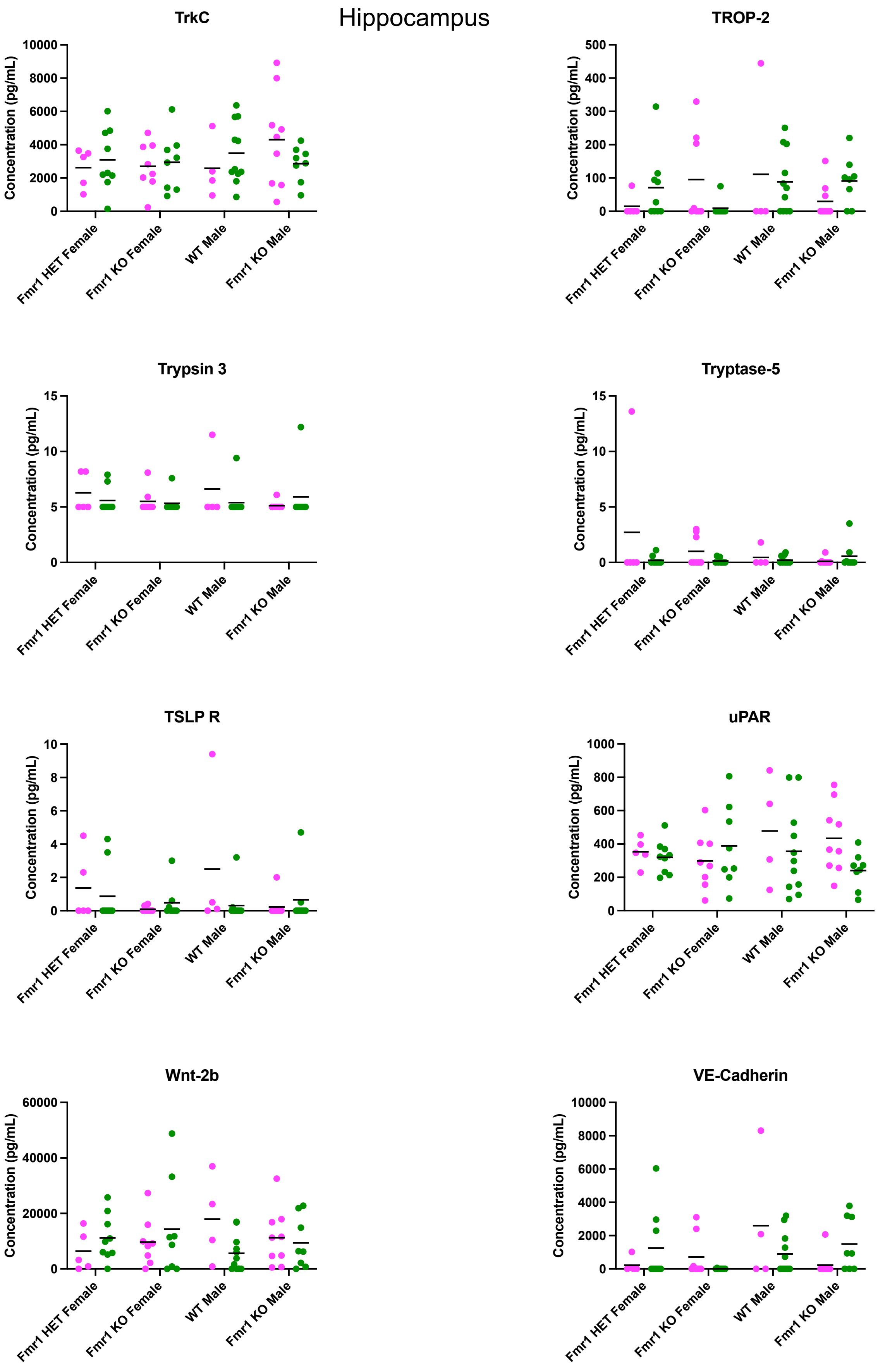

Hippocampus
TrkC
TROP-2
Trypsin 3
Tryptase-5
TSLP R
uPAR
Wnt-2b
VE-Cadherin
Concentration (pg/mL)
Fmr1 HET Female
Fmr1 KO Female
WT Male
Fmr1 KO Male

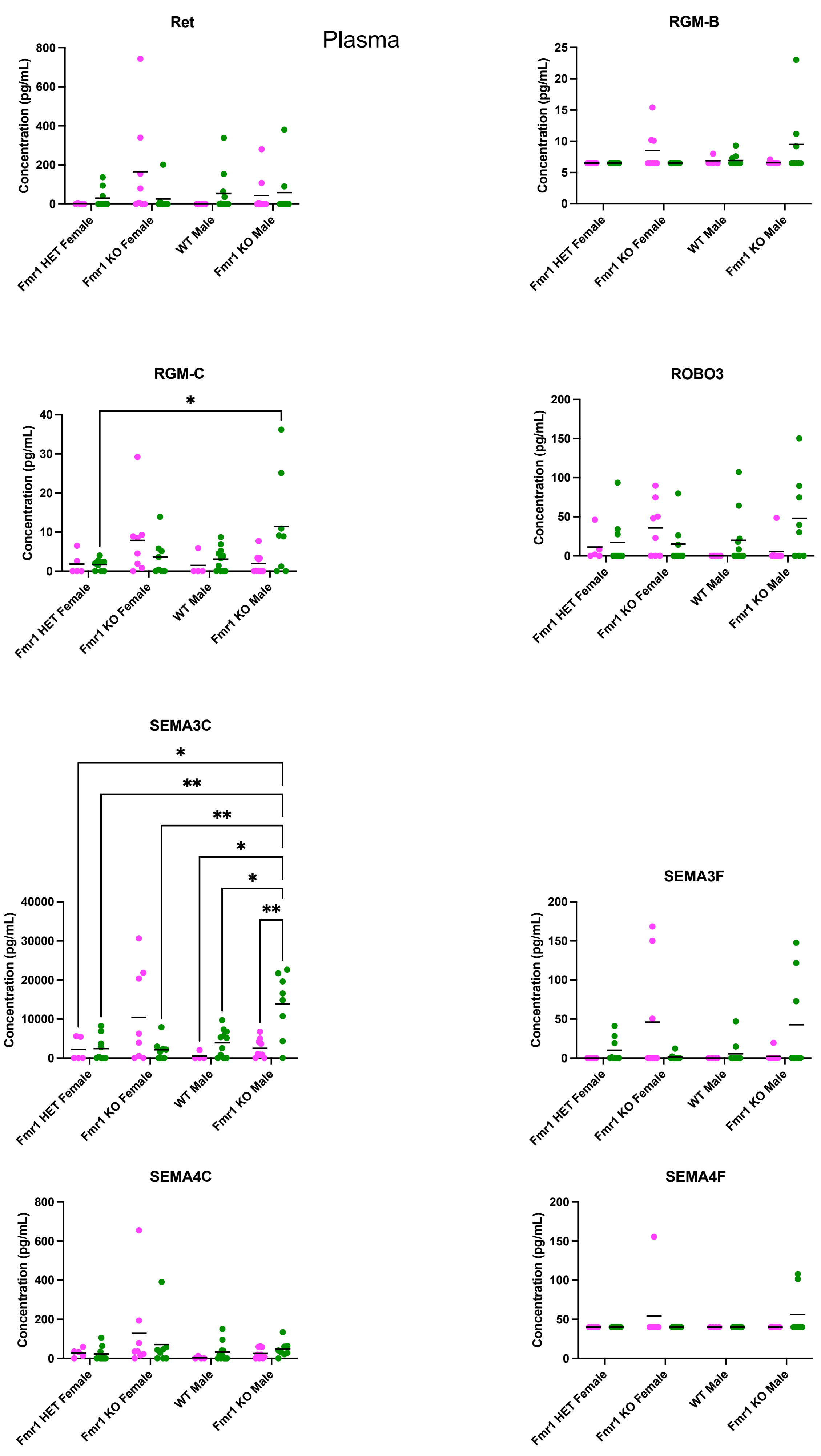
Plasma
Ret
RGM-B
RGM-C
ROBO3
SEMA3C
SEMA3F
SEMA4C
SEMA4F
Concentration (pg/mL)
Fmr1 HET Female
Fmr1 KO Female
WT Male
Fmr1 KO Male
*
**
0
5
10
15
20
25
30
40
50
100
150
200
400
600
800
10000
20000
30000
40000

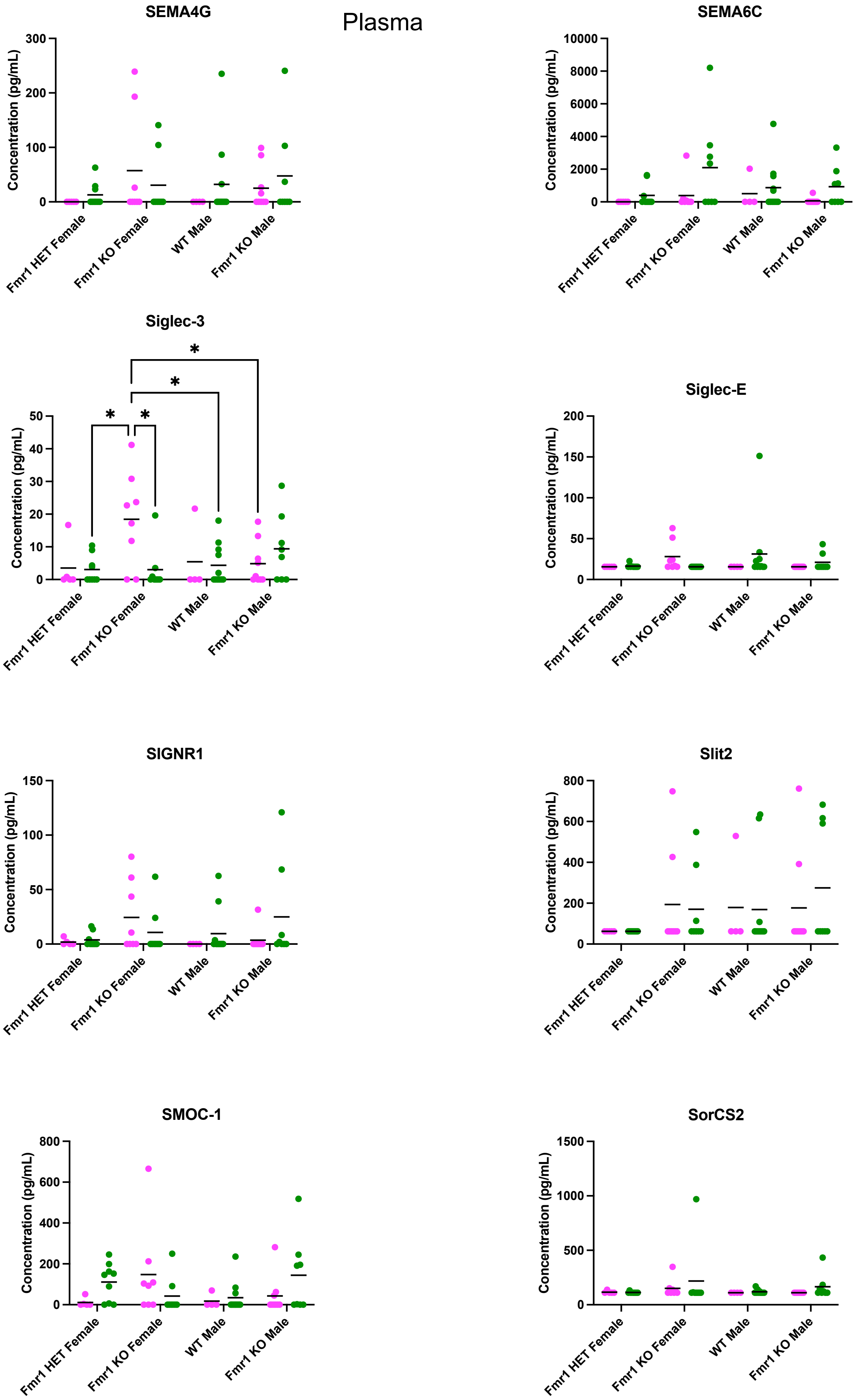
Plasma
SEMA4G
SEMA6C
Siglec-3
Siglec-E
SIGNR1
Slit2
SMOC-1
SorCS2
Concentration (pg/mL)
0
100
200
300
2000
4000
6000
8000
10000
10
20
30
40
50
150
400
600
800
500
1000
1500
*
Fmr1 HET Female
Fmr1 KO Female
WT Male
Fmr1 KO Male

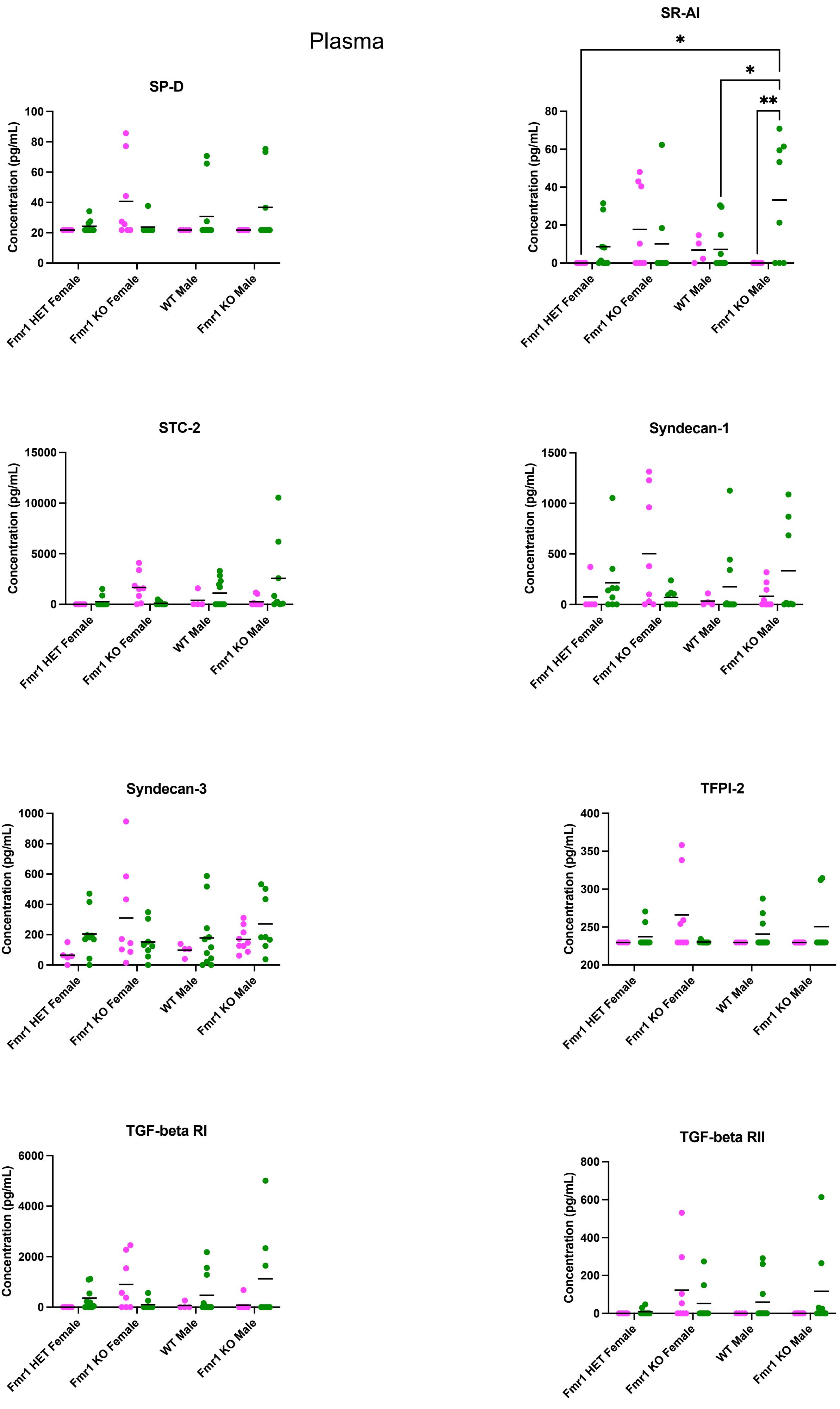

Plasma
SP-D
SR-AI
STC-2
Syndecan-1
Syndecan-3
TFPI-2
TGF-beta RI
TGF-beta RII
Concentration (pg/mL)
Fmr1 HET Female
Fmr1 KO Female
WT Male
Fmr1 KO Male
*
*
**

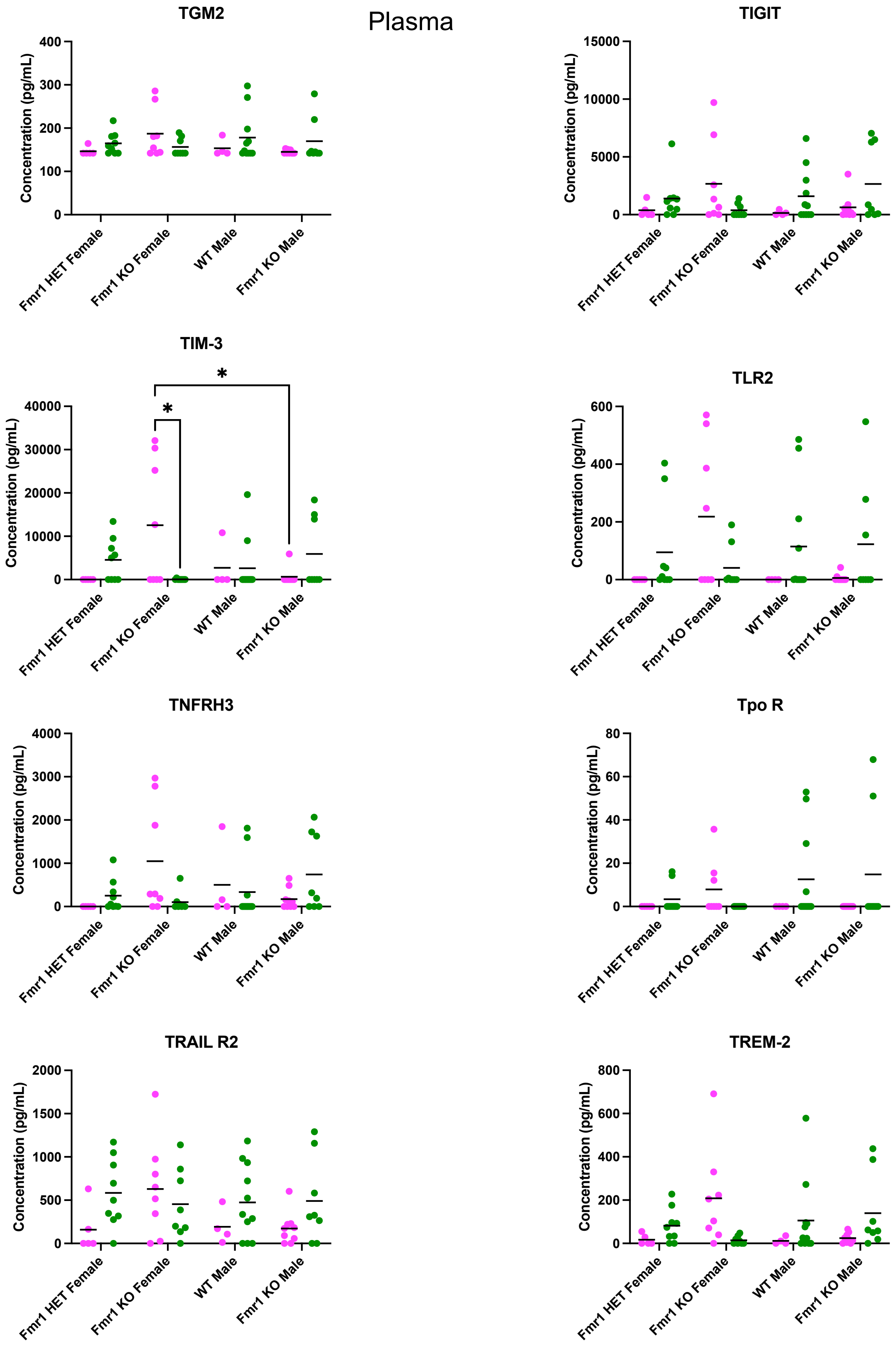
Plasma
TGM2
TIGIT
TIM-3
TLR2
TNFRH3
Tpo R
TRAIL R2
TREM-2
Concentration (pg/mL)
Fmr1 HET Female
Fmr1 KO Female
WT Male
Fmr1 KO Male
*
*

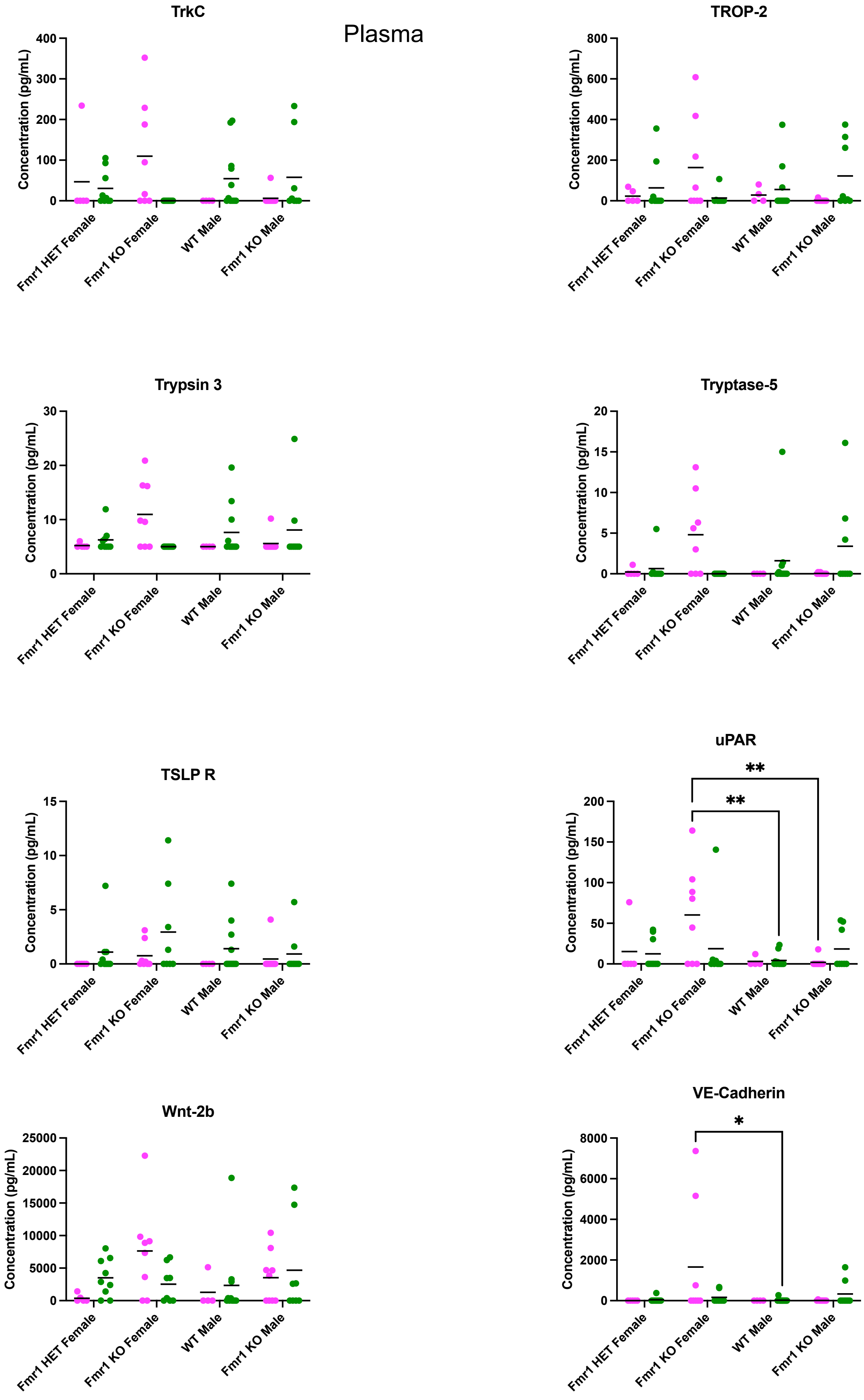
Plasma
TrkC
TROP-2
Trypsin 3
Tryptase-5
TSLP R
uPAR
Wnt-2b
VE-Cadherin
Concentration (pg/mL)
Fmr1 HET Female
Fmr1 KO Female
WT Male
Fmr1 KO Male
**
**
*
0
100
200
300
400
600
800
10
20
30
5
15
50
150
5000
10000
15000
20000
25000
2000
4000
6000
8000
