# Supplementary material for: Soy Protein Isolate Affects Blood and Brain Biomarker Expression in a Mouse Model of Fragile X
Source: Int J Mol Sci. 2025 Jun 26;26(13):6137. doi: 10.3390/ijms26136137 (PMC12250412; doi:10.3390/ijms26136137)
Supplement: Supplementary file 1 [file ijms-26-06137-s001.zip › Supplementary File S12b Array 14 Graphs.pdf]

Supplementary File S12. Protein expression of Array 14 targets as function of *Fmr1* genotype and AIN-93G diets. Mice on AIN-93G/cas (colored pink) included n=5 $Fmr1^{HET}$ female, n=8 $Fmr1^{KO}$ female, n=4 WT male and n=9 $Fmr1^{KO}$ male. Mice on AIN-93G/soy (colored green) included n=9 $Fmr1^{HET}$ female, n=8 $Fmr1^{KO}$ female, n=11 WT male and n=8 $Fmr1^{KO}$ male. The average concentration in cortex, hippocampus, hypothalamus and plasma in pg/mL was plotted versus genotype. Statistics were determined by 2-way ANOVA and Tukey's multiple comparison tests denoted by $p < 0.05$ (*), $p < 0.01$ (**), $p < 0.001$ (***) and $p < 0.0001$ (****).

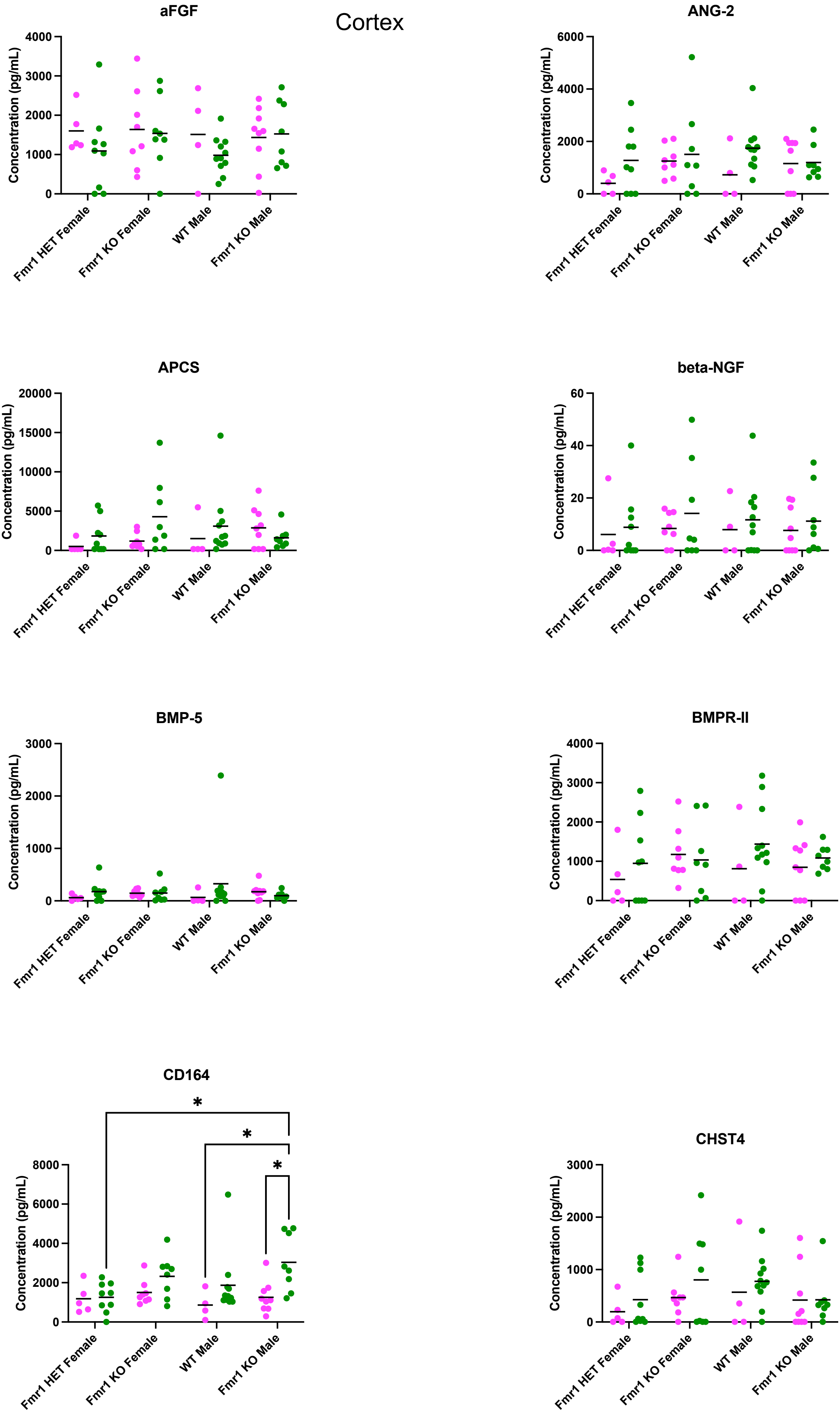
Cortex
aFGF
ANG-2
APCS
beta-NGF
BMP-5
BMPR-II
CD164
CHST4
Concentration (pg/mL)
Fmr1 HET Female
Fmr1 KO Female
WT Male
Fmr1 KO Male
*
*
*

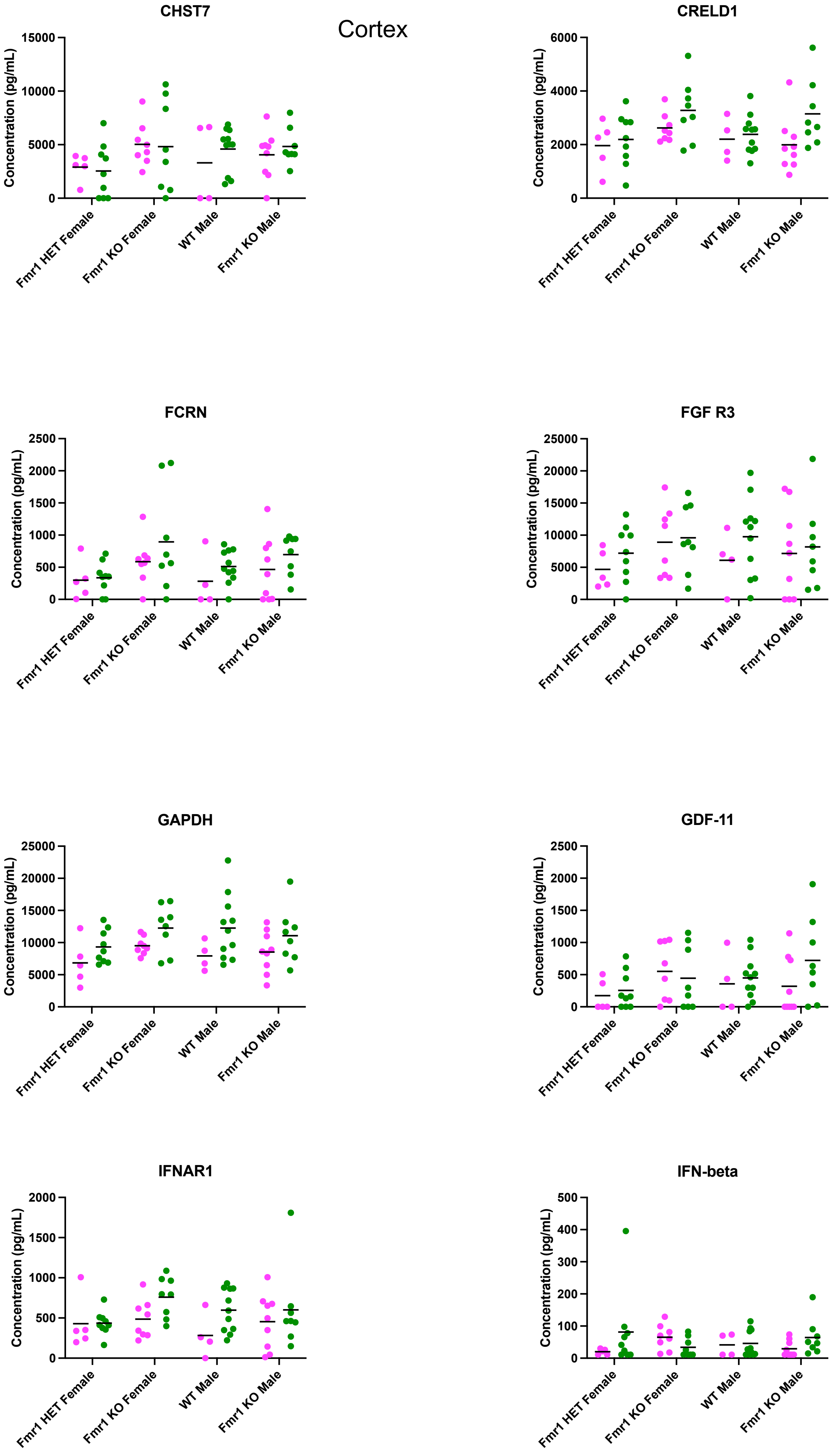
Cortex
CHST7
CRELD1
FCRN
FGF R3
GAPDH
GDF-11
IFNAR1
IFN-beta
Concentration (pg/mL)
0
5000
10000
15000
20000
25000
2000
4000
6000
500
1000
1500
2500
100
200
300
400
Fmr1 HET Female
Fmr1 KO Female
WT Male
Fmr1 KO Male

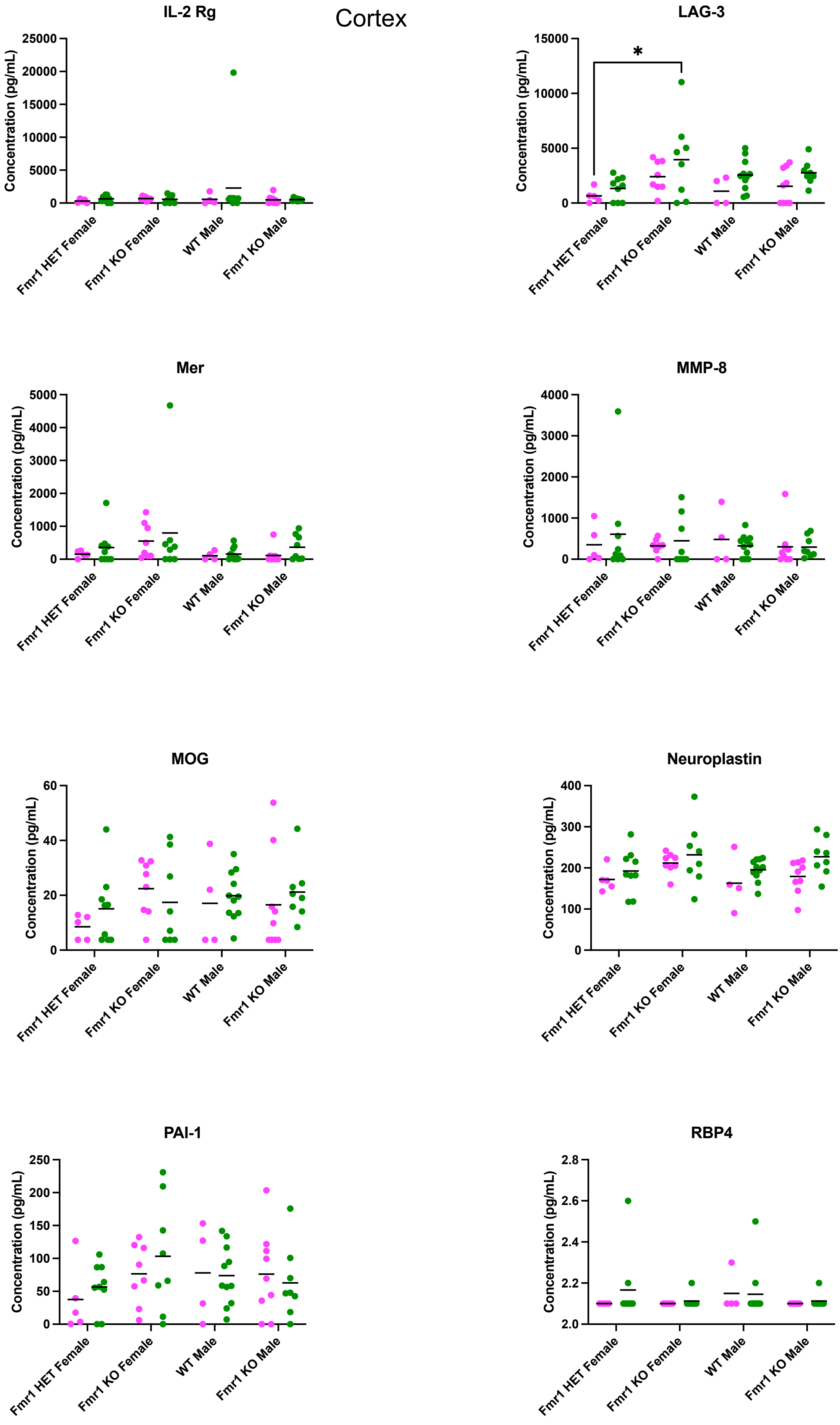

Cortex
IL-2 Rg
LAG-3
Mer
MMP-8
MOG
Neuroplastin
PAI-1
RBP4
Concentration (pg/mL)
Fmr1 HET Female
Fmr1 KO Female
WT Male
Fmr1 KO Male
*

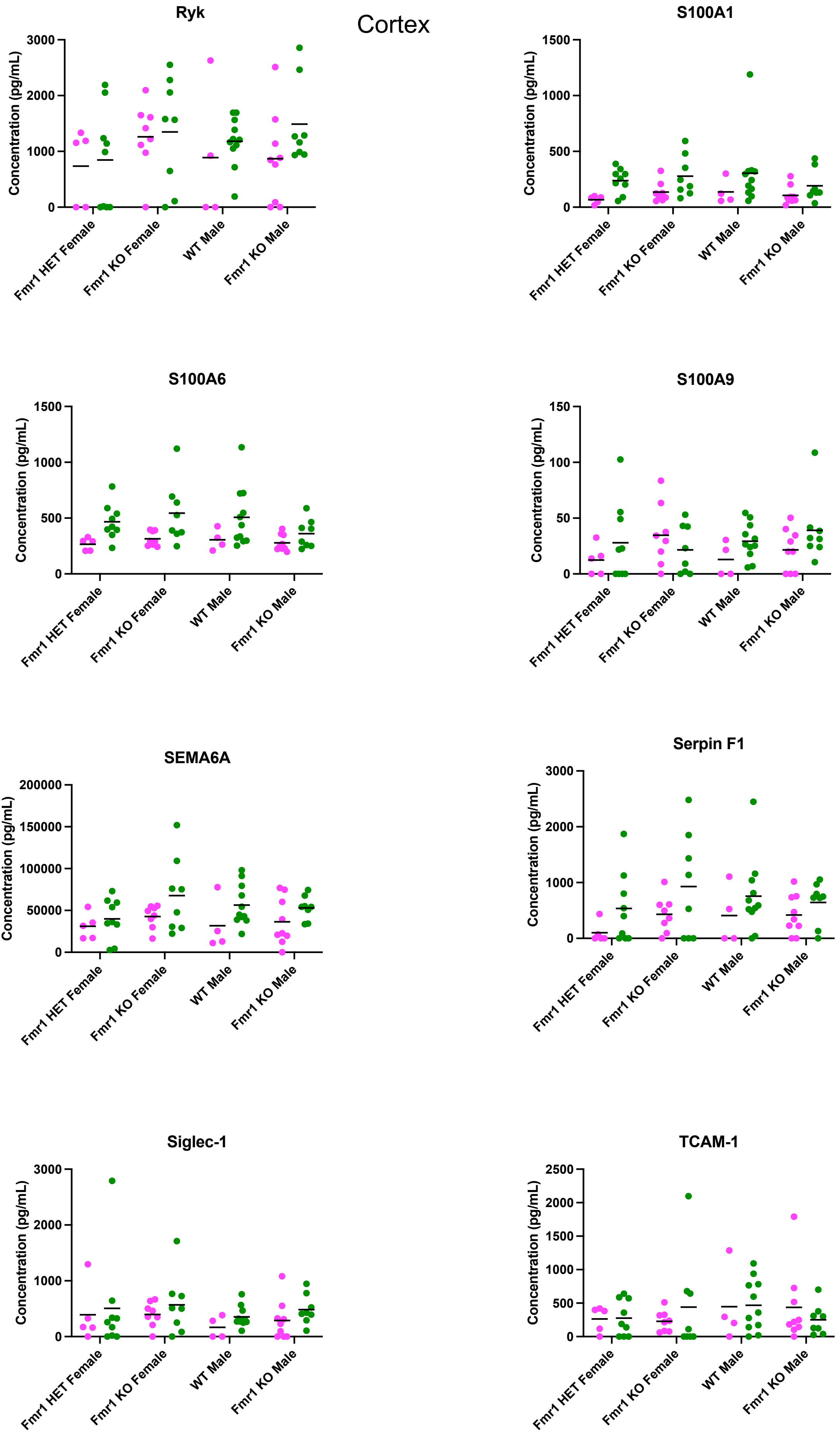
Cortex
Ryk
S100A1
S100A6
S100A9
SEMA6A
Serpin F1
Siglec-1
TCAM-1
Concentration (pg/mL)
Fmr1 HET Female
Fmr1 KO Female
WT Male
Fmr1 KO Male
0
1000
2000
3000
500
1500
50
100
150
50000
100000
150000
200000
2500

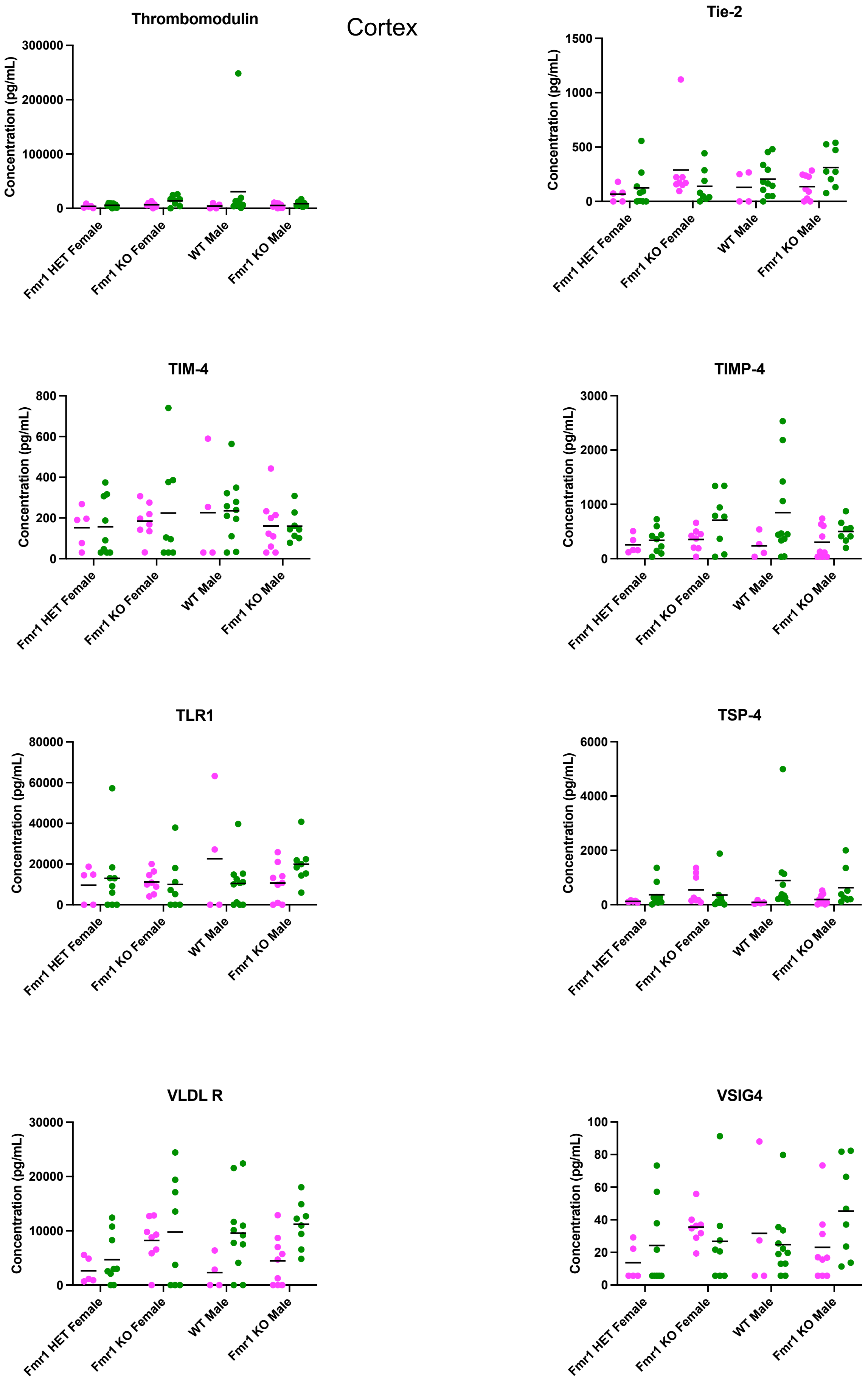
Cortex
Thrombomodulin
Concentration (pg/mL)
300000
200000
100000
0
Fmr1 HET Female
Fmr1 KO Female
WT Male
Fmr1 KO Male
Tie-2
Concentration (pg/mL)
1500
1000
500
0
Fmr1 HET Female
Fmr1 KO Female
WT Male
Fmr1 KO Male
TIM-4
Concentration (pg/mL)
800
600
400
200
0
Fmr1 HET Female
Fmr1 KO Female
WT Male
Fmr1 KO Male
TIMP-4
Concentration (pg/mL)
3000
2000
1000
0
Fmr1 HET Female
Fmr1 KO Female
WT Male
Fmr1 KO Male
TLR1
Concentration (pg/mL)
80000
60000
40000
20000
0
Fmr1 HET Female
Fmr1 KO Female
WT Male
Fmr1 KO Male
TSP-4
Concentration (pg/mL)
6000
4000
2000
0
Fmr1 HET Female
Fmr1 KO Female
WT Male
Fmr1 KO Male
VLDL R
Concentration (pg/mL)
30000
20000
10000
0
Fmr1 HET Female
Fmr1 KO Female
WT Male
Fmr1 KO Male
VSIG4
Concentration (pg/mL)
100
80
60
40
20
0
Fmr1 HET Female
Fmr1 KO Female
WT Male
Fmr1 KO Male

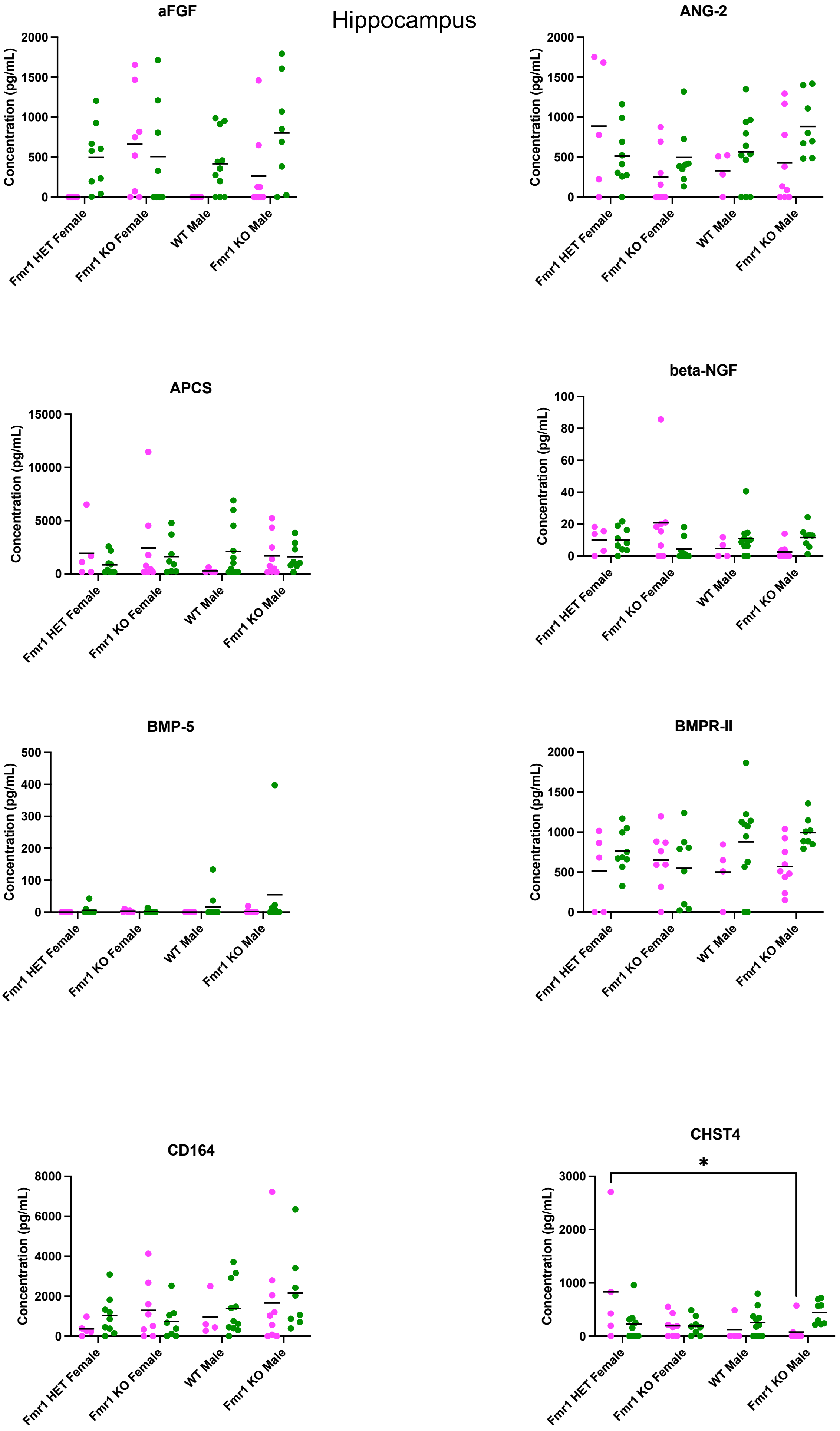
Hippocampus
aFGF
ANG-2
APCS
beta-NGF
BMP-5
BMPR-II
CD164
CHST4
*
Concentration (pg/mL)
Fmr1 HET Female
Fmr1 KO Female
WT Male
Fmr1 KO Male
0
500
1000
1500
2000
3000
4000
6000
8000
5000
10000
15000
20
40
60
80
100
200
300
400

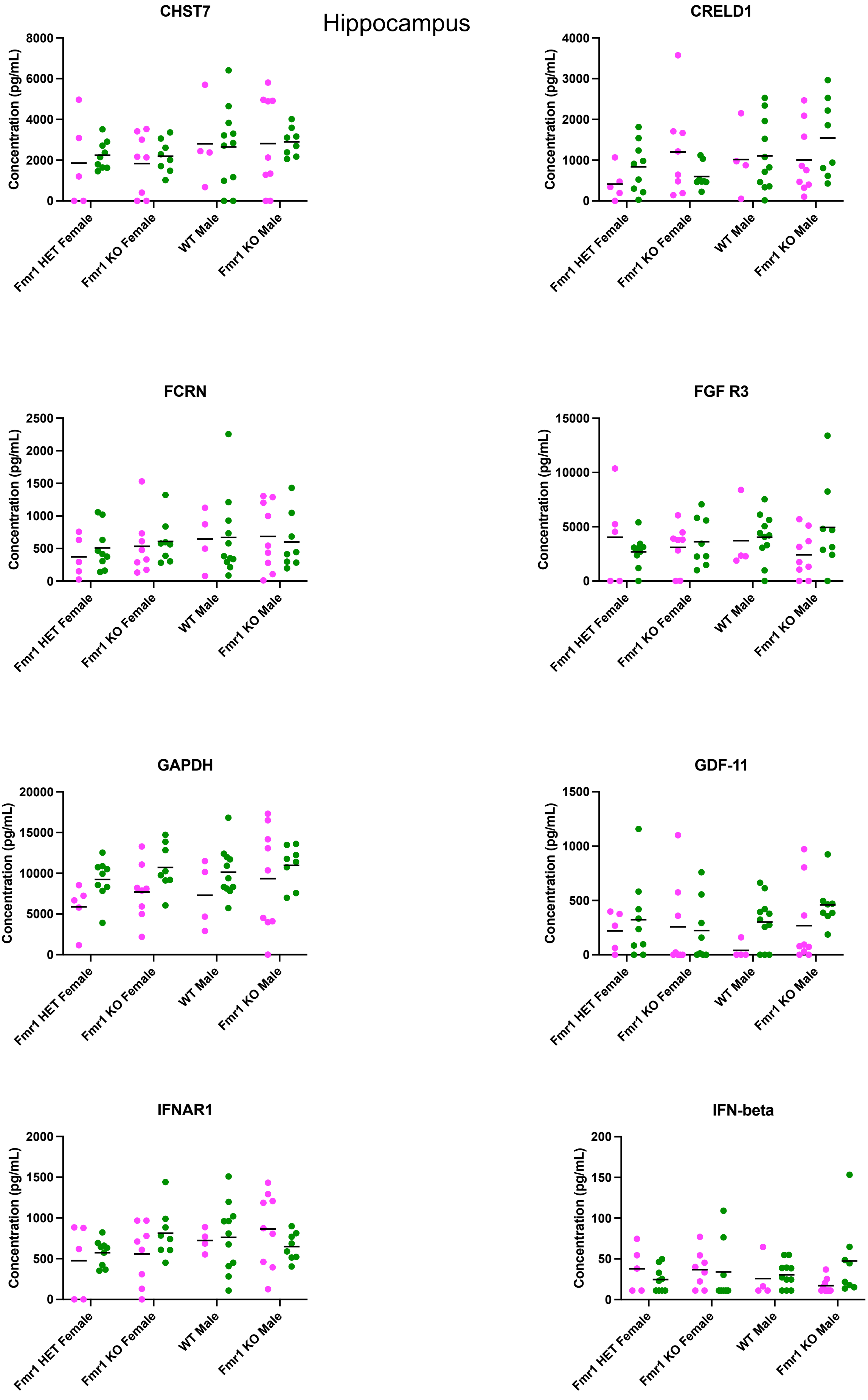
Hippocampus
CHST7
CRELD1
FCRN
FGF R3
GAPDH
GDF-11
IFNAR1
IFN-beta
Concentration (pg/mL)
Fmr1 HET Female
Fmr1 KO Female
WT Male
Fmr1 KO Male

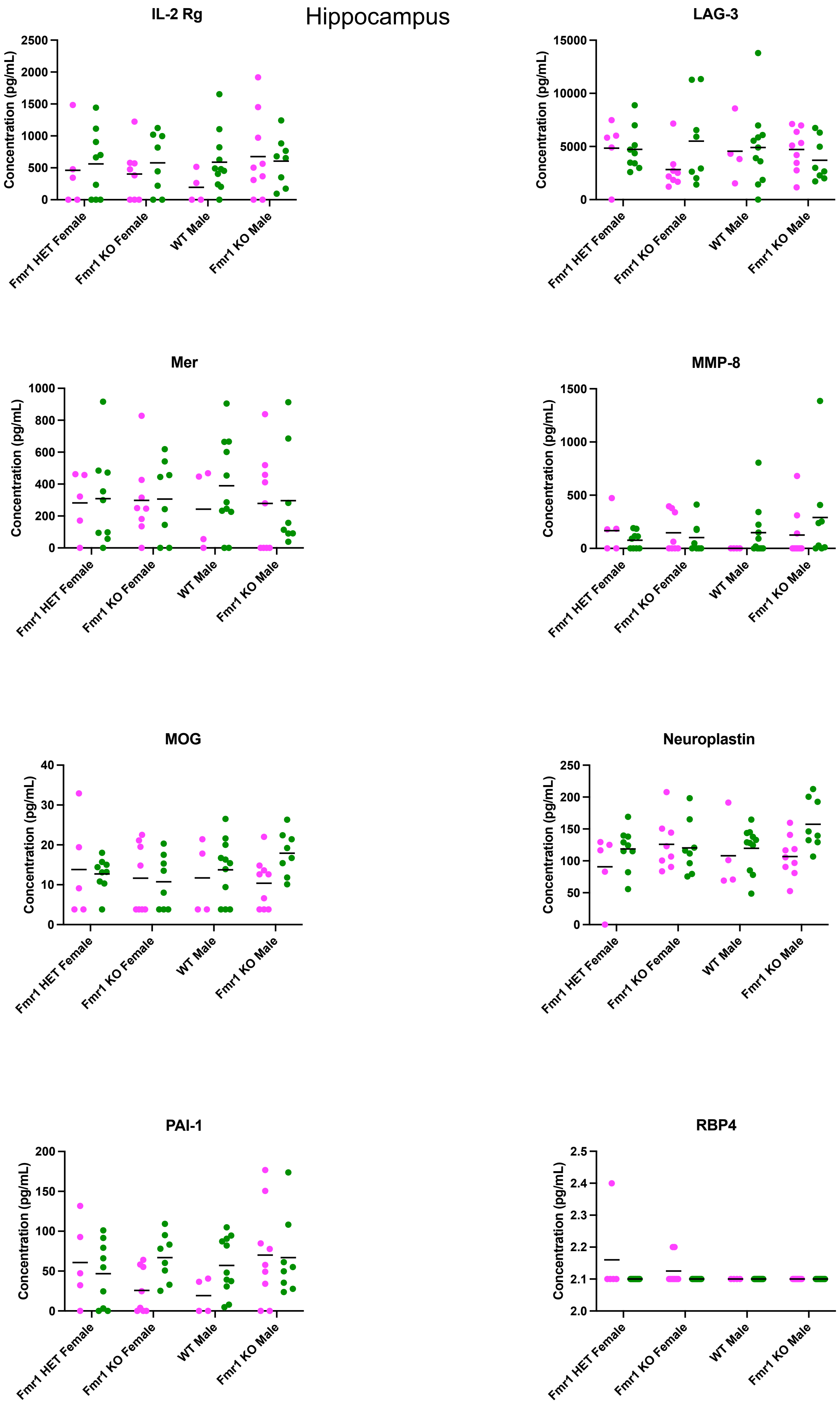
Hippocampus
IL-2 Rg
LAG-3
Mer
MMP-8
MOG
Neuroplastin
PAI-1
RBP4
Concentration (pg/mL)
Fmr1 HET Female
Fmr1 KO Female
WT Male
Fmr1 KO Male

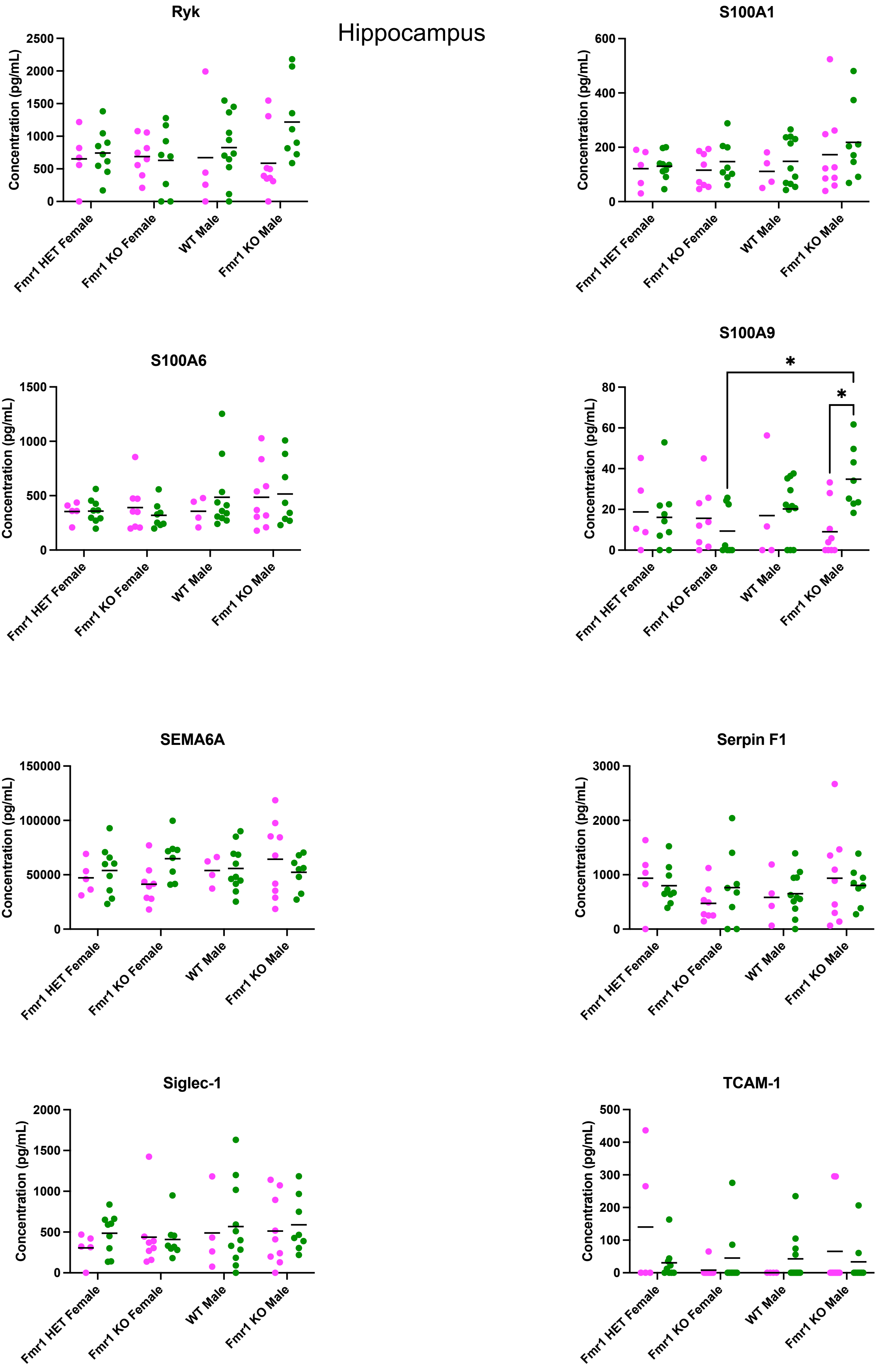

Hippocampus
Ryk
S100A1
S100A6
S100A9
SEMA6A
Serpin F1
Siglec-1
TCAM-1
Concentration (pg/mL)
0
500
1000
1500
2000
2500
200
400
600
20
40
60
80
50000
100000
150000
3000
100
300
Fmr1 HET Female
Fmr1 KO Female
WT Male
Fmr1 KO Male
*
*

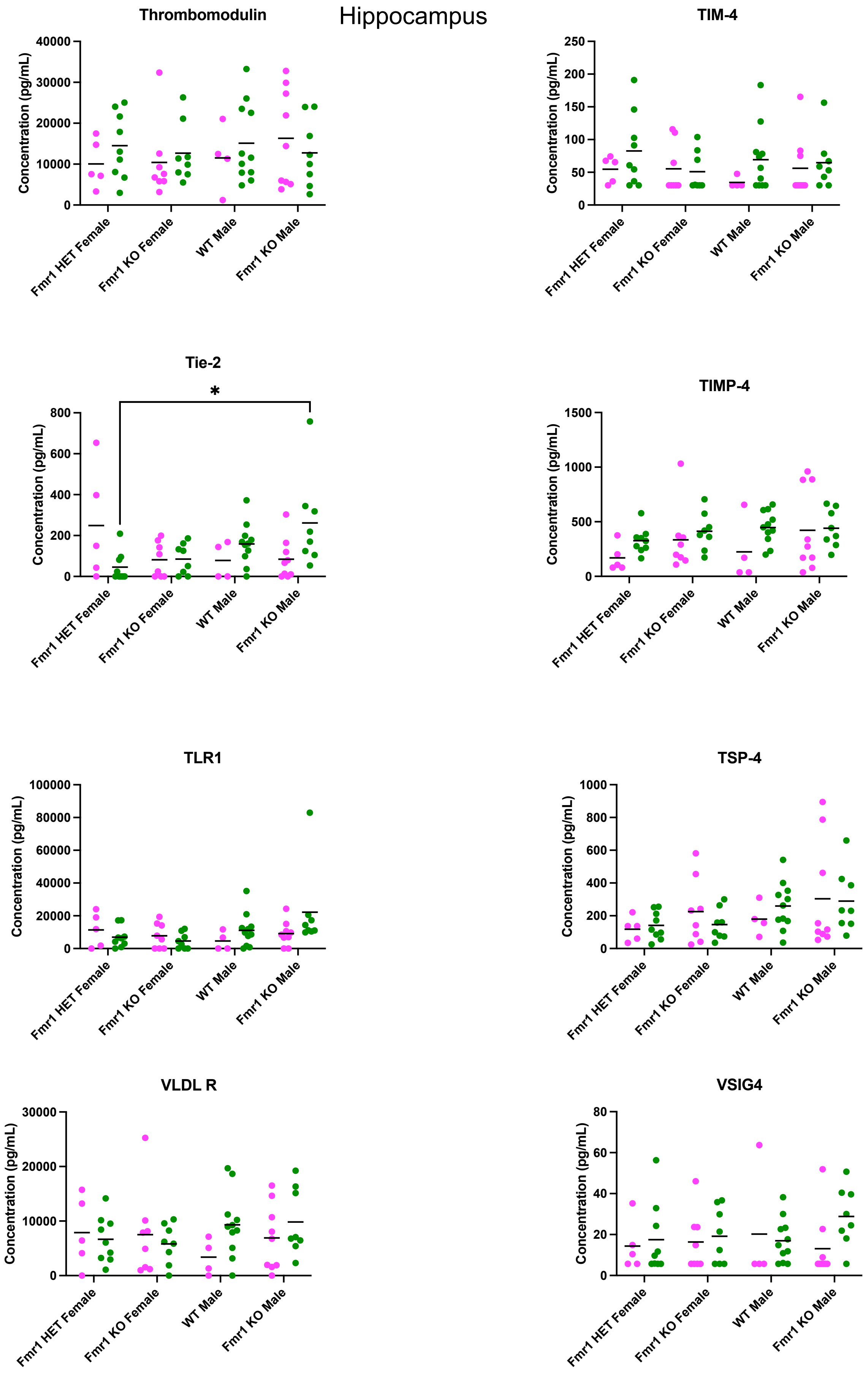
Hippocampus
Thrombomodulin
TIM-4
Tie-2
*
TIMP-4
TLR1
TSP-4
VLDL R
VSIG4
Concentration (pg/mL)
40000
30000
20000
10000
0
250
200
150
100
50
800
600
400
1500
1000
500
100000
80000
60000
40000
20000
1000
30000
80
60
40
20
Fmr1 HET Female
Fmr1 KO Female
WT Male
Fmr1 KO Male

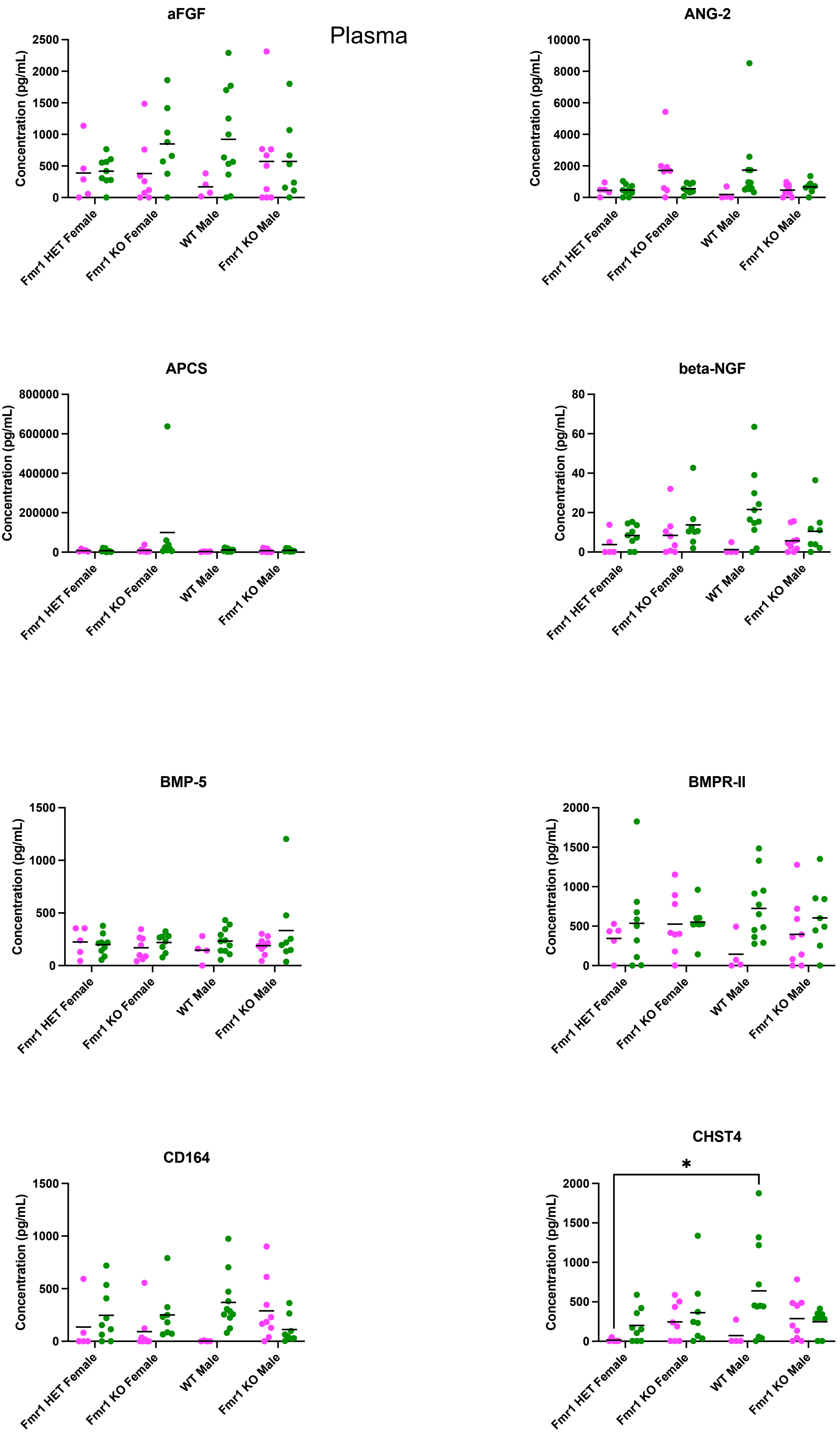
Plasma
aFGF
Concentration (pg/mL)
0
500
1000
1500
2000
2500
Fmr1 HET Female
Fmr1 KO Female
WT Male
Fmr1 KO Male
ANG-2
Concentration (pg/mL)
0
2000
4000
6000
8000
10000
Fmr1 HET Female
Fmr1 KO Female
WT Male
Fmr1 KO Male
APCS
Concentration (pg/mL)
0
200000
400000
600000
800000
Fmr1 HET Female
Fmr1 KO Female
WT Male
Fmr1 KO Male
beta-NGF
Concentration (pg/mL)
0
20
40
60
80
Fmr1 HET Female
Fmr1 KO Female
WT Male
Fmr1 KO Male
BMP-5
Concentration (pg/mL)
0
500
1000
1500
Fmr1 HET Female
Fmr1 KO Female
WT Male
Fmr1 KO Male
BMPR-II
Concentration (pg/mL)
0
500
1000
1500
2000
Fmr1 HET Female
Fmr1 KO Female
WT Male
Fmr1 KO Male
CD164
Concentration (pg/mL)
0
500
1000
1500
Fmr1 HET Female
Fmr1 KO Female
WT Male
Fmr1 KO Male
CHST4
*
Concentration (pg/mL)
0
500
1000
1500
2000
Fmr1 HET Female
Fmr1 KO Female
WT Male
Fmr1 KO Male

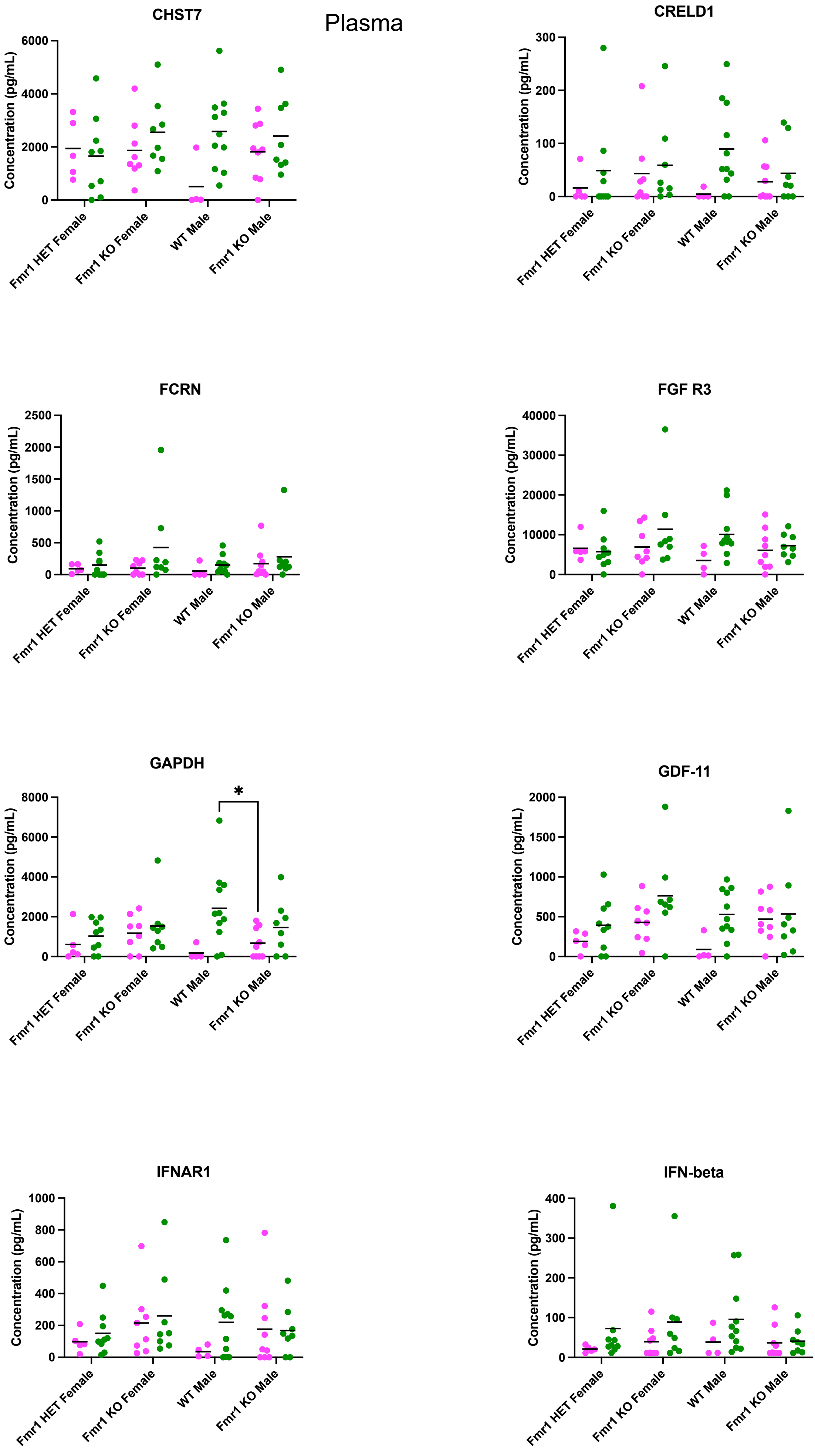
Plasma
CHST7
CRELD1
FCRN
FGF R3
GAPDH
GDF-11
IFNAR1
IFN-beta
Concentration (pg/mL)
Fmr1 HET Female
Fmr1 KO Female
WT Male
Fmr1 KO Male
*
0
2000
4000
6000
100
200
300
500
1000
1500
2000
2500
10000
20000
30000
40000
8000
400
600
800

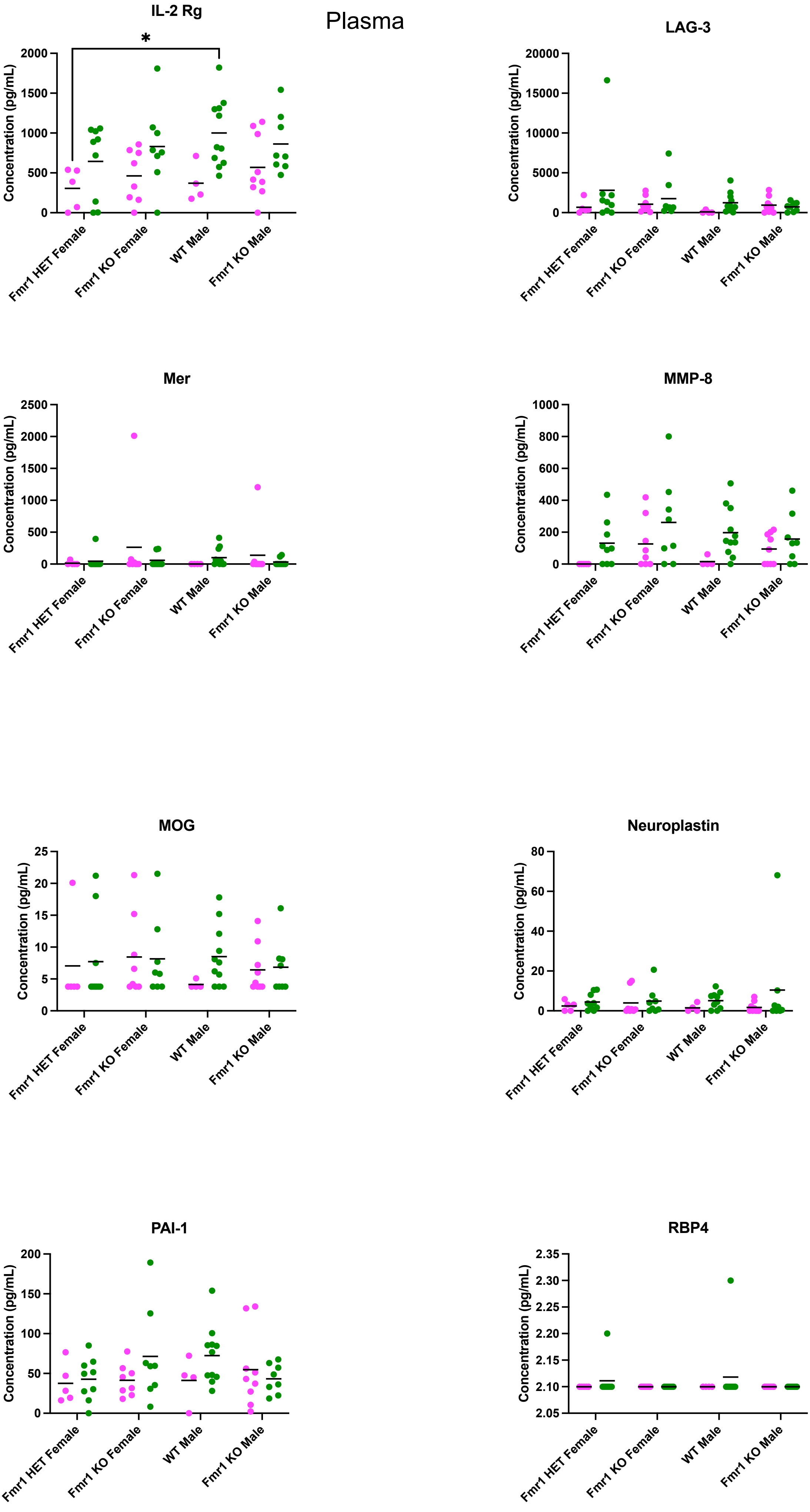

Plasma
IL-2 Rg
*
LAG-3
Mer
MMP-8
MOG
Neuroplastin
PAI-1
RBP4
Concentration (pg/mL)
Fmr1 HET Female
Fmr1 KO Female
WT Male
Fmr1 KO Male

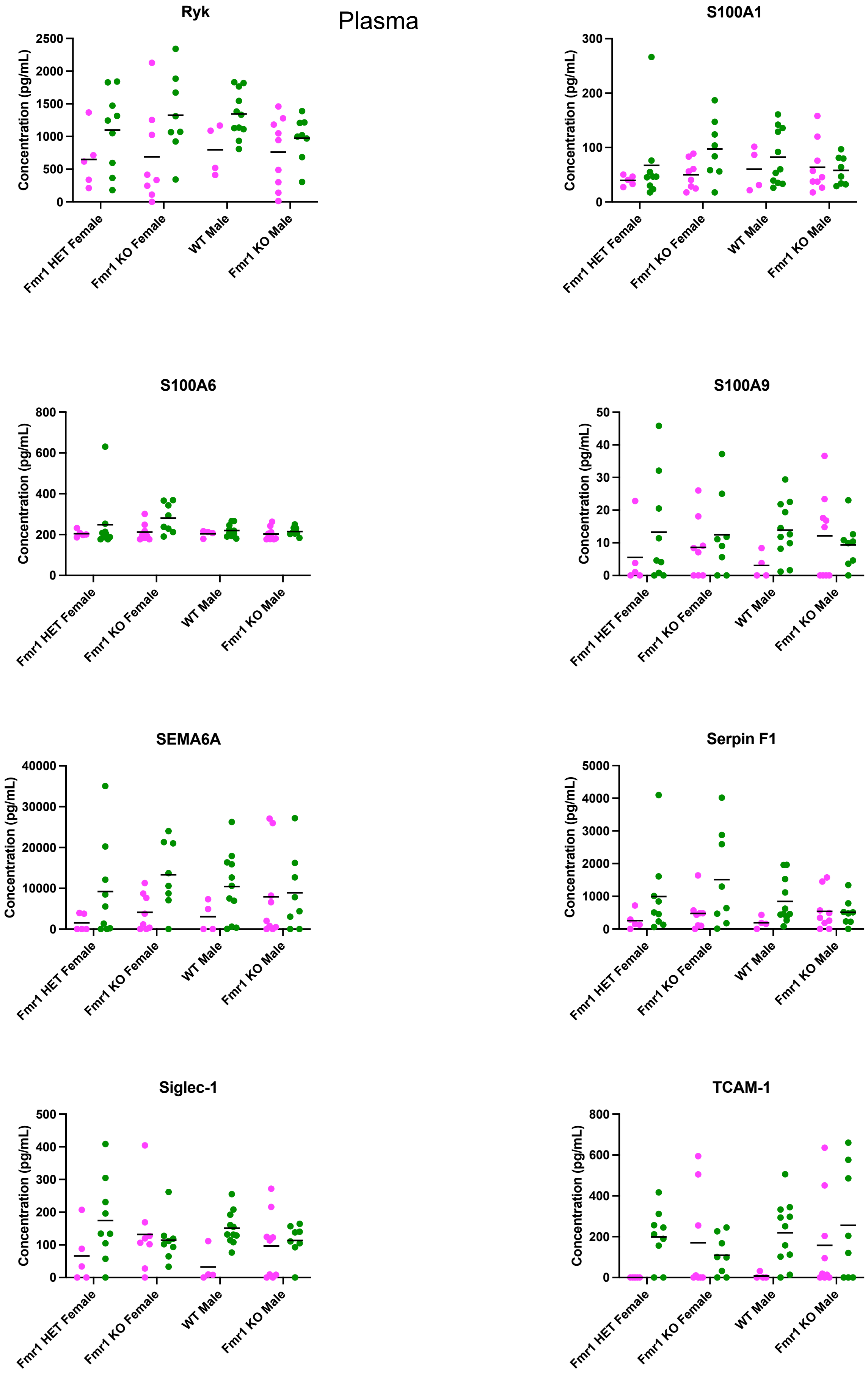
Plasma
Ryk
S100A1
S100A6
S100A9
SEMA6A
Serpin F1
Siglec-1
TCAM-1
Concentration (pg/mL)
Fmr1 HET Female
Fmr1 KO Female
WT Male
Fmr1 KO Male

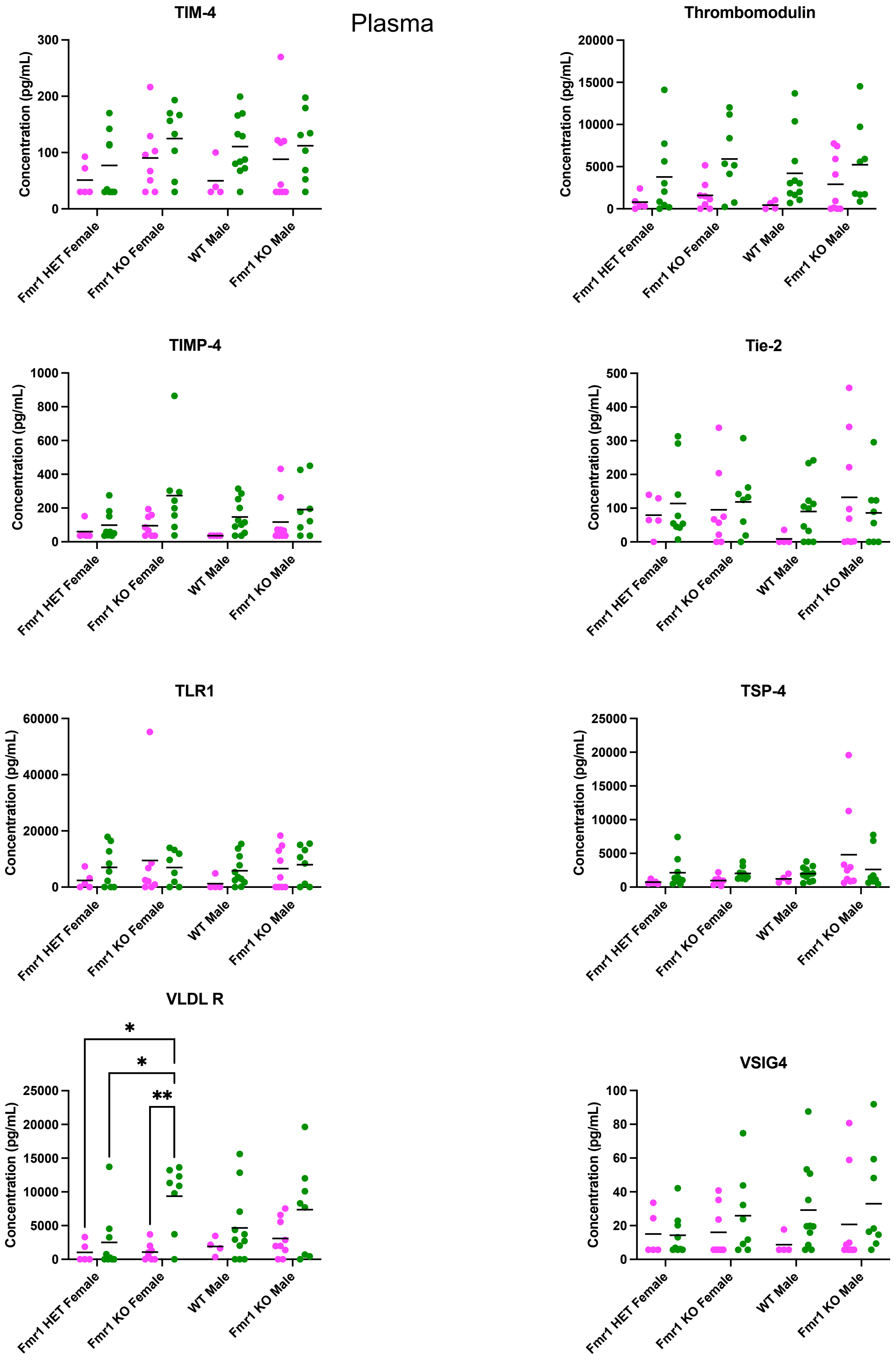
Plasma
TIM-4
Thrombomodulin
TIMP-4
Tie-2
TLR1
TSP-4
VLDL R
VSIG4
Concentration (pg/mL)
Fmr1 HET Female
Fmr1 KO Female
WT Male
Fmr1 KO Male
*
*
**
