# Supplementary material for: Soy Protein Isolate Affects Blood and Brain Biomarker Expression in a Mouse Model of Fragile X
Source: Int J Mol Sci. 2025 Jun 26;26(13):6137. doi: 10.3390/ijms26136137 (PMC12250412; doi:10.3390/ijms26136137)
Supplement: Supplementary file 1 [file ijms-26-06137-s001.zip › Supplementary File S13b Array 15 Graphs.pdf]

Supplementary File S13. Protein expression of Array 15 targets as function of *Fmr1* genotype and AIN-93G diets. Mice on AIN-93G/cas (colored pink) included n=5 $Fmr1^{HET}$ female, n=8 $Fmr1^{KO}$ female, n=4 WT male and n=9 $Fmr1^{KO}$ male. Mice on AIN-93G/soy (colored green) included n=9 $Fmr1^{HET}$ female, n=8 $Fmr1^{KO}$ female, n=11 WT male and n=8 $Fmr1^{KO}$ male. The average concentration in cortex, hippocampus, hypothalamus and plasma in pg/mL was plotted versus genotype. Statistics were determined by 2-way ANOVA and Tukey's multiple comparison tests denoted by $p < 0.05$ (*), $p < 0.01$ (**), $p < 0.001$ (***) and $p < 0.0001$ (****).

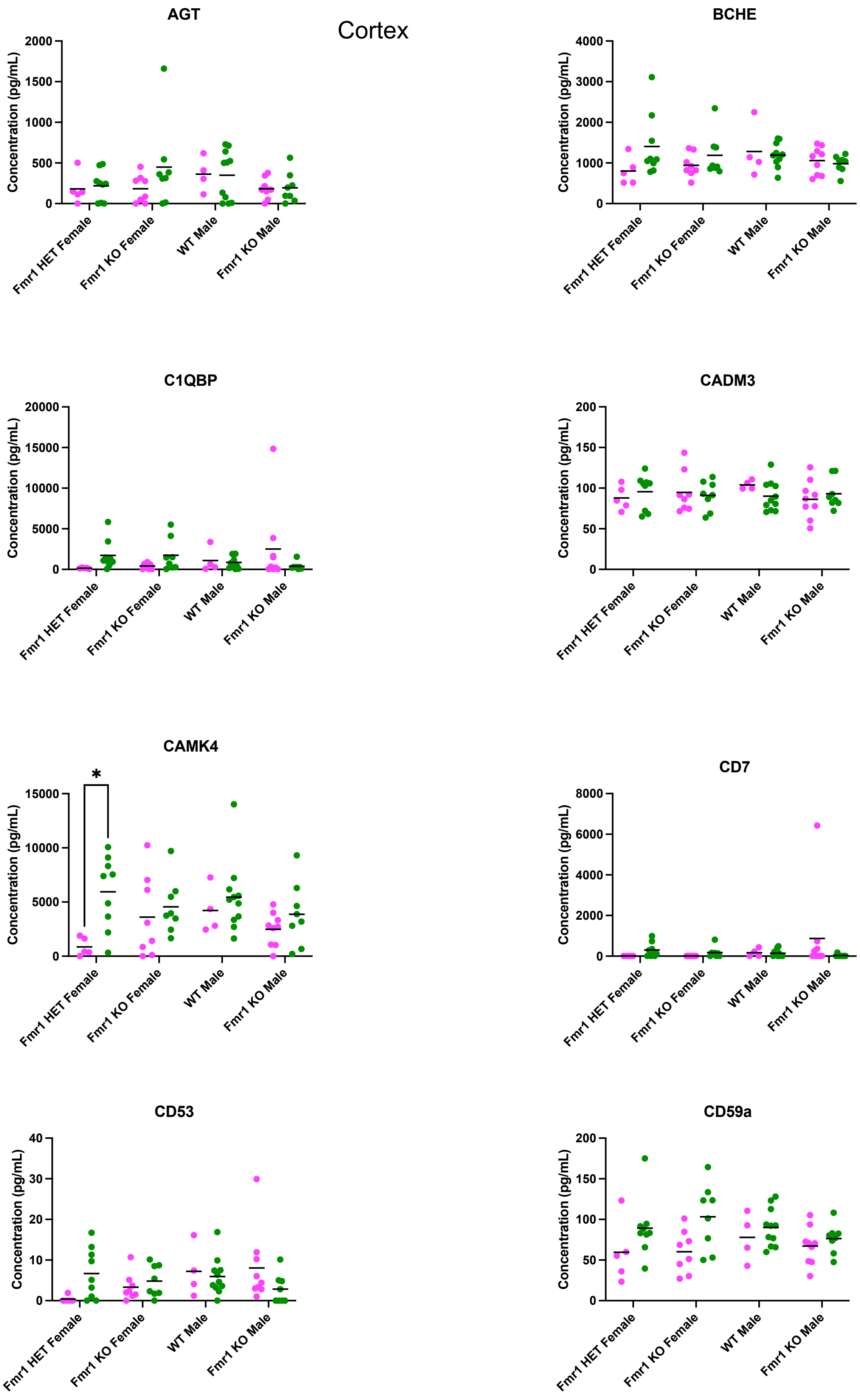
Cortex
AGT
BCHE
C1QBP
CADM3
CAMK4
CD7
CD53
CD59a
*
Concentration (pg/mL)
0
500
1000
1500
2000
3000
4000
5000
10000
15000
20000
50
100
150
200
6000
8000
10
20
30
40
Fmr1 HET Female
Fmr1 KO Female
WT Male
Fmr1 KO Male

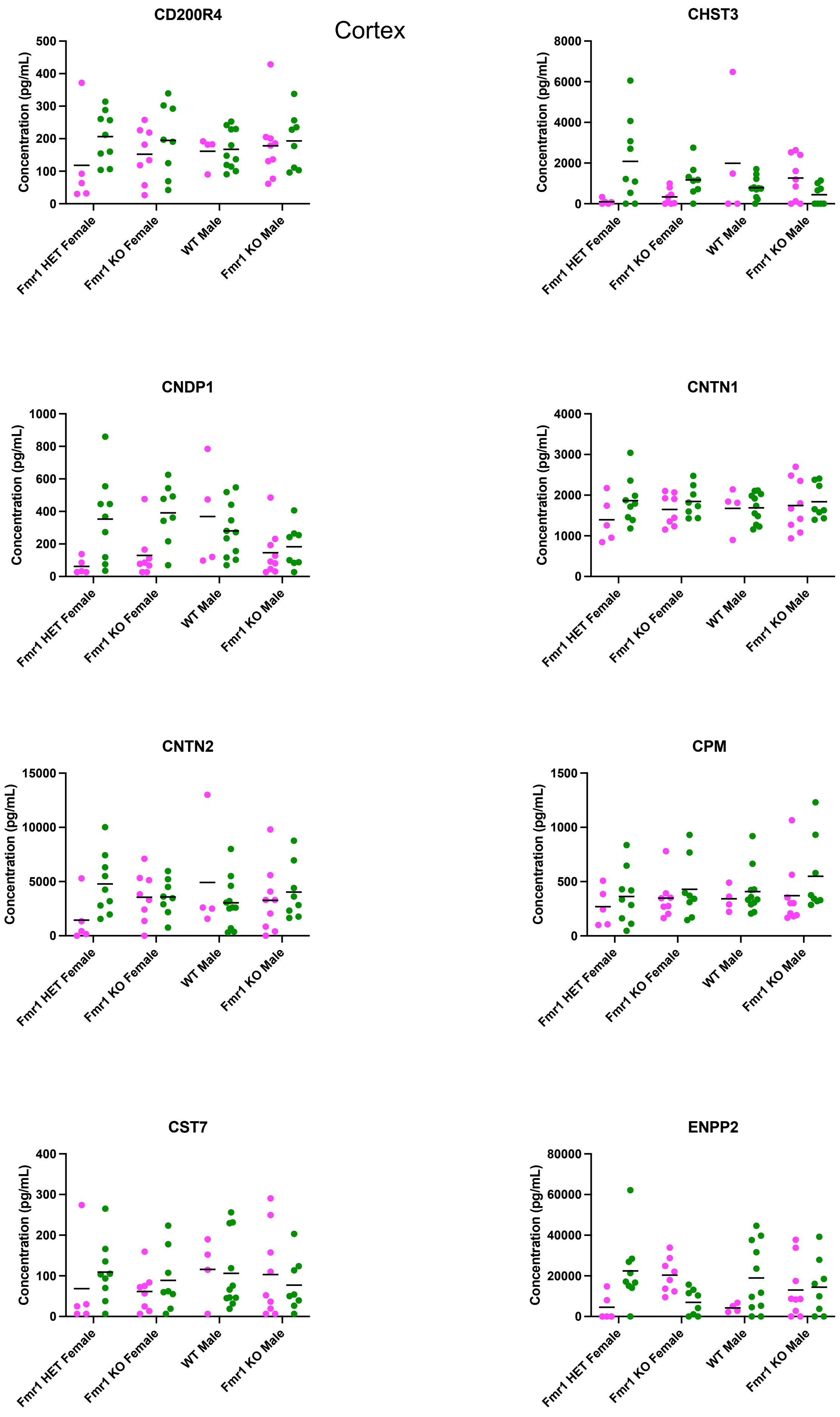
Cortex
CD200R4
CHST3
CNDP1
CNTN1
CNTN2
CPM
CST7
ENPP2
Concentration (pg/mL)
Fmr1 HET Female
Fmr1 KO Female
WT Male
Fmr1 KO Male

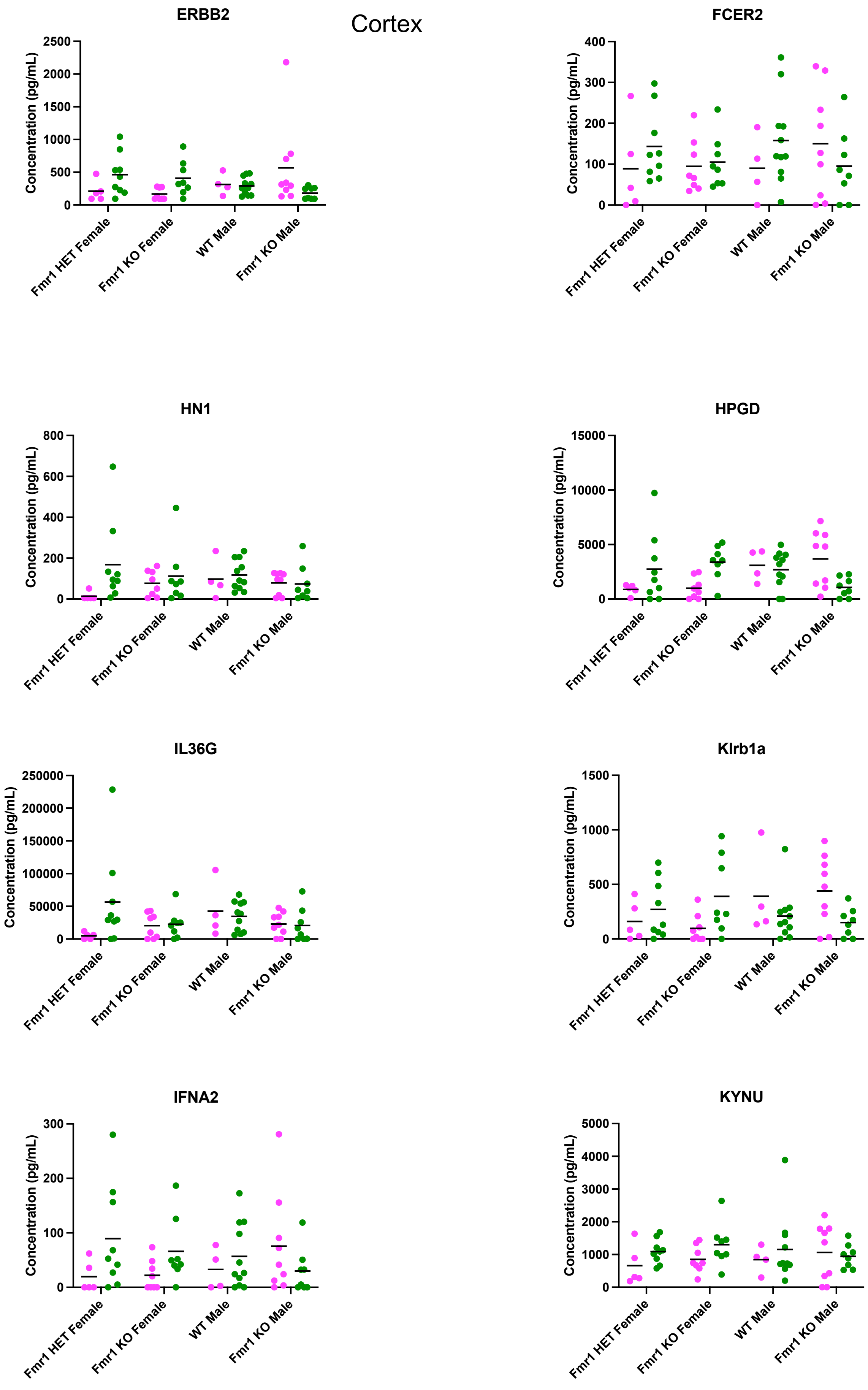
Cortex
ERBB2
FCER2
HN1
HPGD
IL36G
Klrb1a
IFNA2
KYNU
Concentration (pg/mL)
Fmr1 HET Female
Fmr1 KO Female
WT Male
Fmr1 KO Male

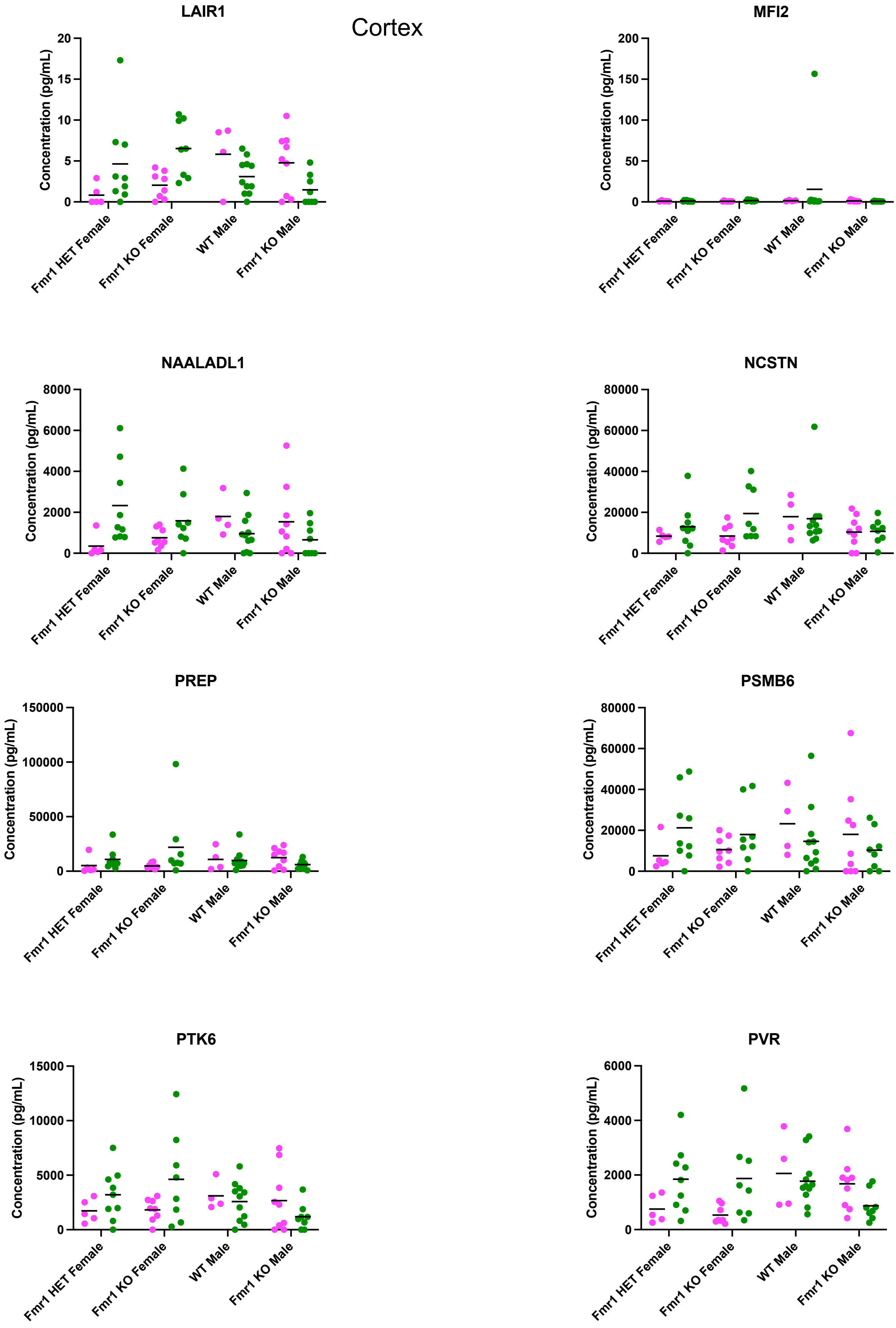
Cortex
LAIR1
MFI2
NAALADL1
NCSTN
PREP
PSMB6
PTK6
PVR
Concentration (pg/mL)
Fmr1 HET Female
Fmr1 KO Female
WT Male
Fmr1 KO Male
0
5
10
15
20
50
100
150
200
2000
4000
6000
8000
20000
40000
60000
80000
50000
100000
150000
5000
10000
15000

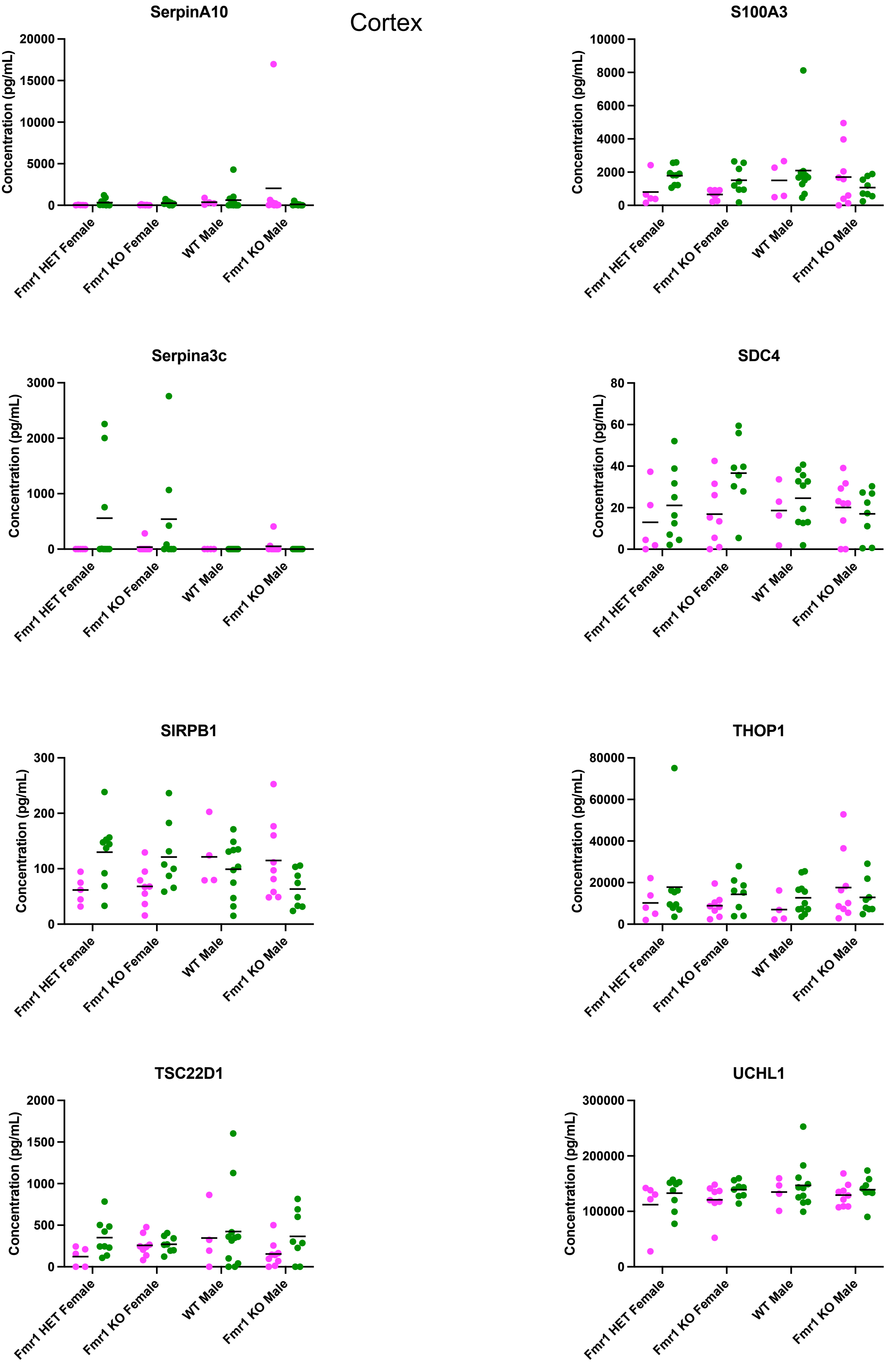
Cortex
SerpinA10
S100A3
Serpina3c
SDC4
SIRPB1
THOP1
TSC22D1
UCHL1
Concentration (pg/mL)
Fmr1 HET Female
Fmr1 KO Female
WT Male
Fmr1 KO Male
0
5000
10000
15000
20000
2000
4000
6000
8000
1000
3000
20
40
60
80
100
200
300
20000
40000
60000
80000
500
1500
100000
200000
300000

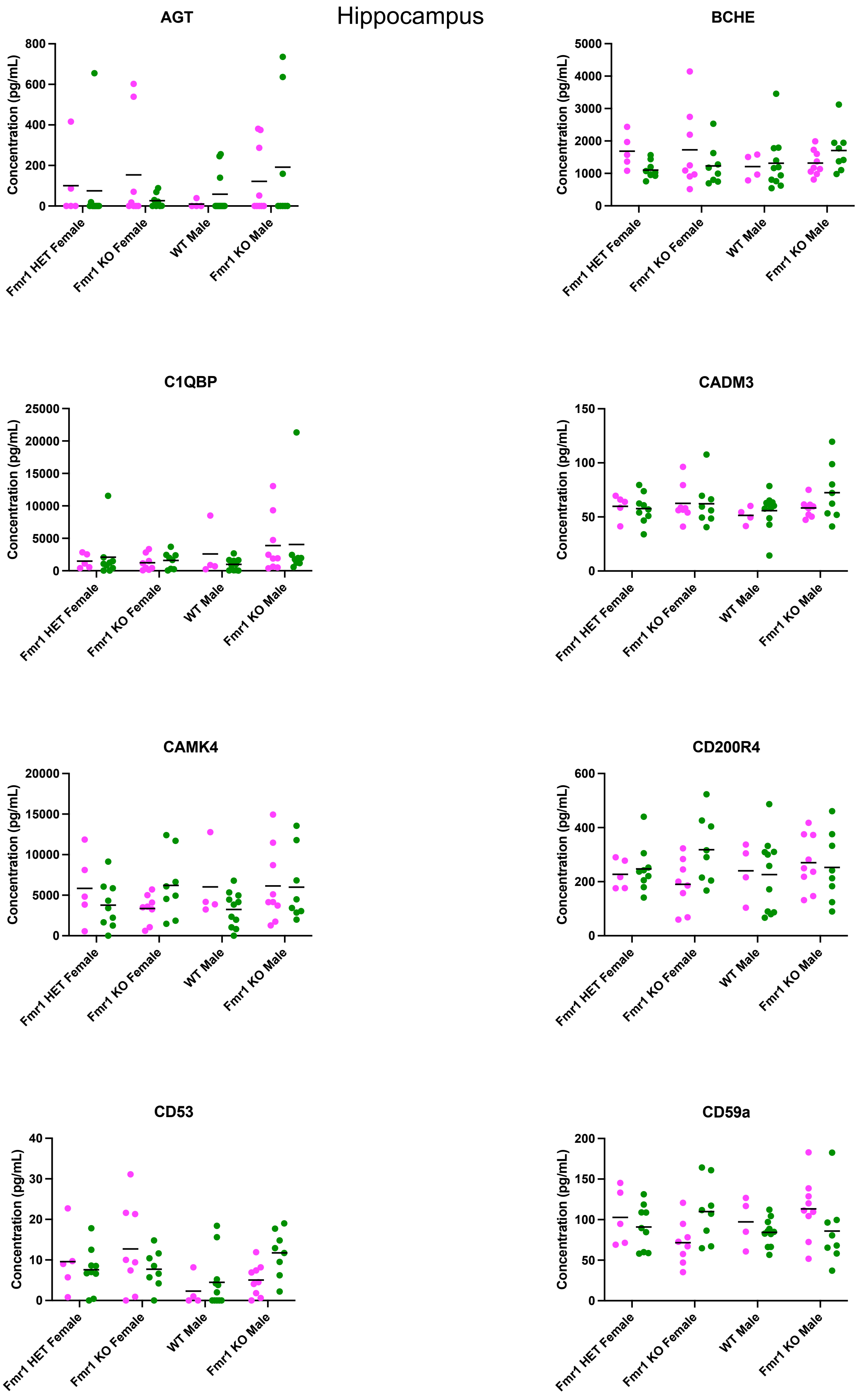
Hippocampus
AGT
BCHE
C1QBP
CADM3
CAMK4
CD200R4
CD53
CD59a
Concentration (pg/mL)
Fmr1 HET Female
Fmr1 KO Female
WT Male
Fmr1 KO Male
0
200
400
600
800
1000
2000
3000
4000
5000
10000
15000
20000
25000
50
100
150
30
40

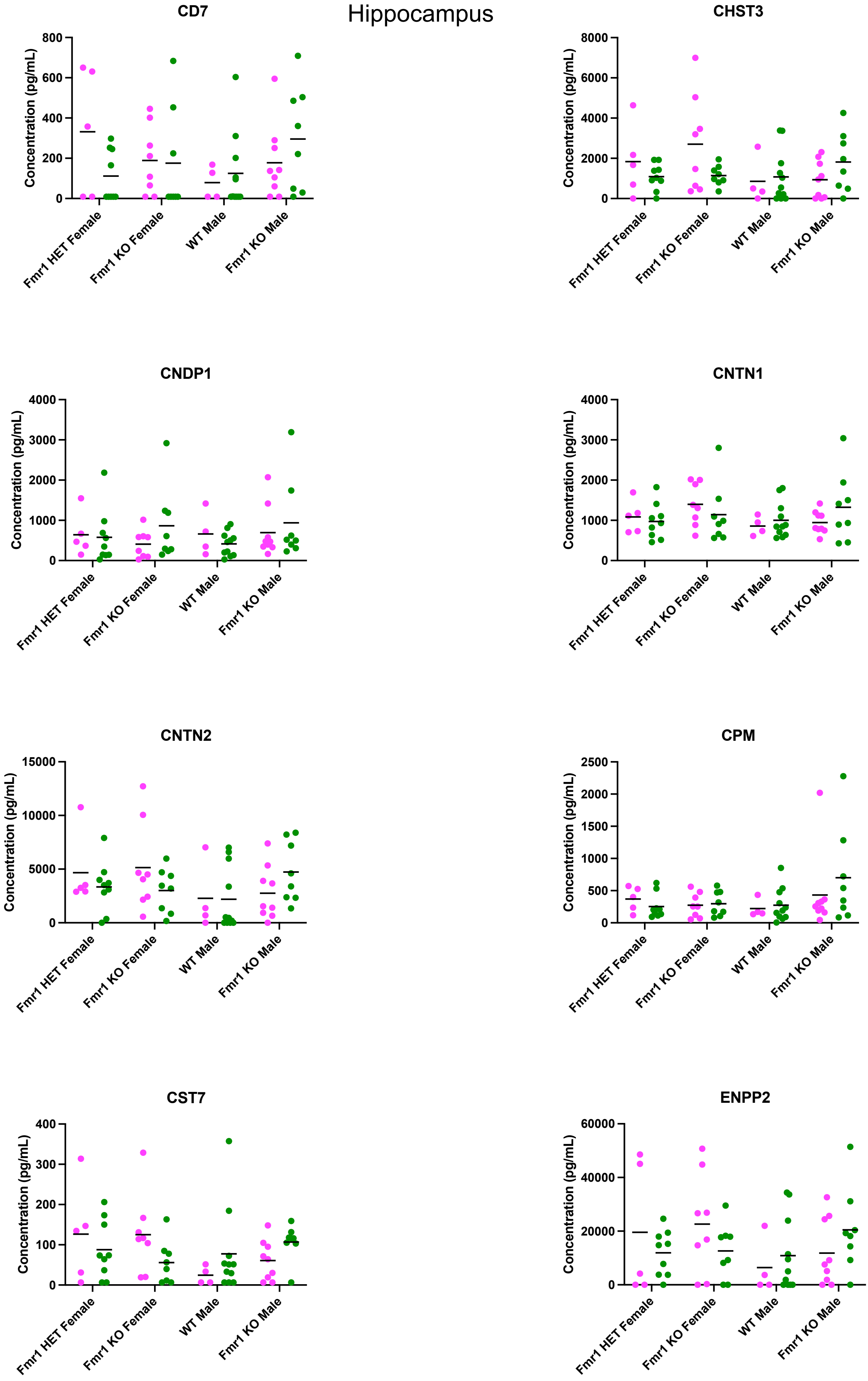
Hippocampus
CD7
CHST3
CNDP1
CNTN1
CNTN2
CPM
CST7
ENPP2
Concentration (pg/mL)
Fmr1 HET Female
Fmr1 KO Female
WT Male
Fmr1 KO Male
0
200
400
600
800
2000
4000
6000
8000
1000
3000
5000
10000
15000
500
1500
2500
100
300
20000
40000
60000

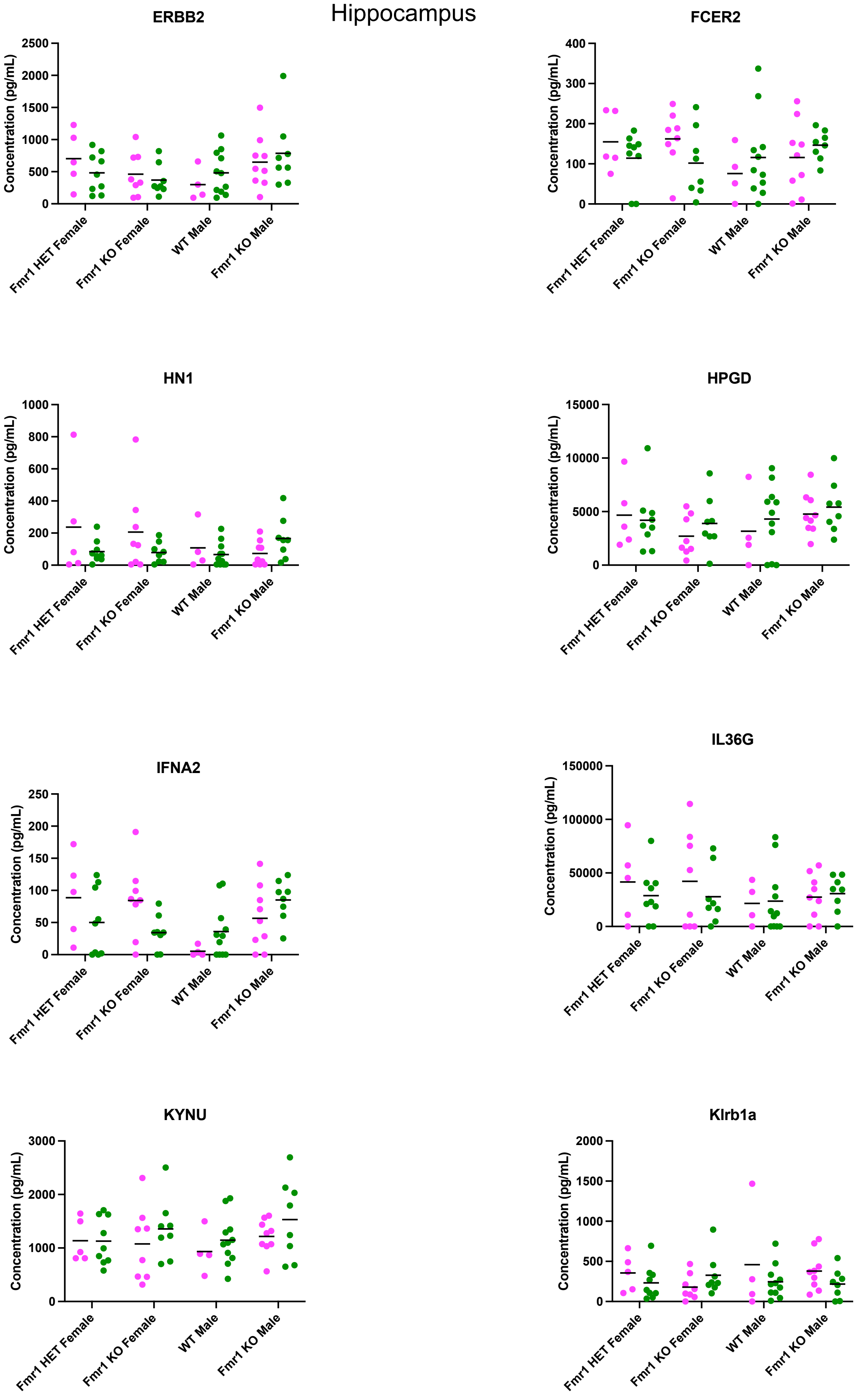
Hippocampus
ERBB2
FCER2
HN1
HPGD
IFNA2
IL36G
KYNU
Klrb1a
Concentration (pg/mL)
Fmr1 HET Female
Fmr1 KO Female
WT Male
Fmr1 KO Male
0
500
1000
1500
2000
2500
100
200
300
400
600
800
5000
10000
15000
50
150
250
50000
100000
150000
3000

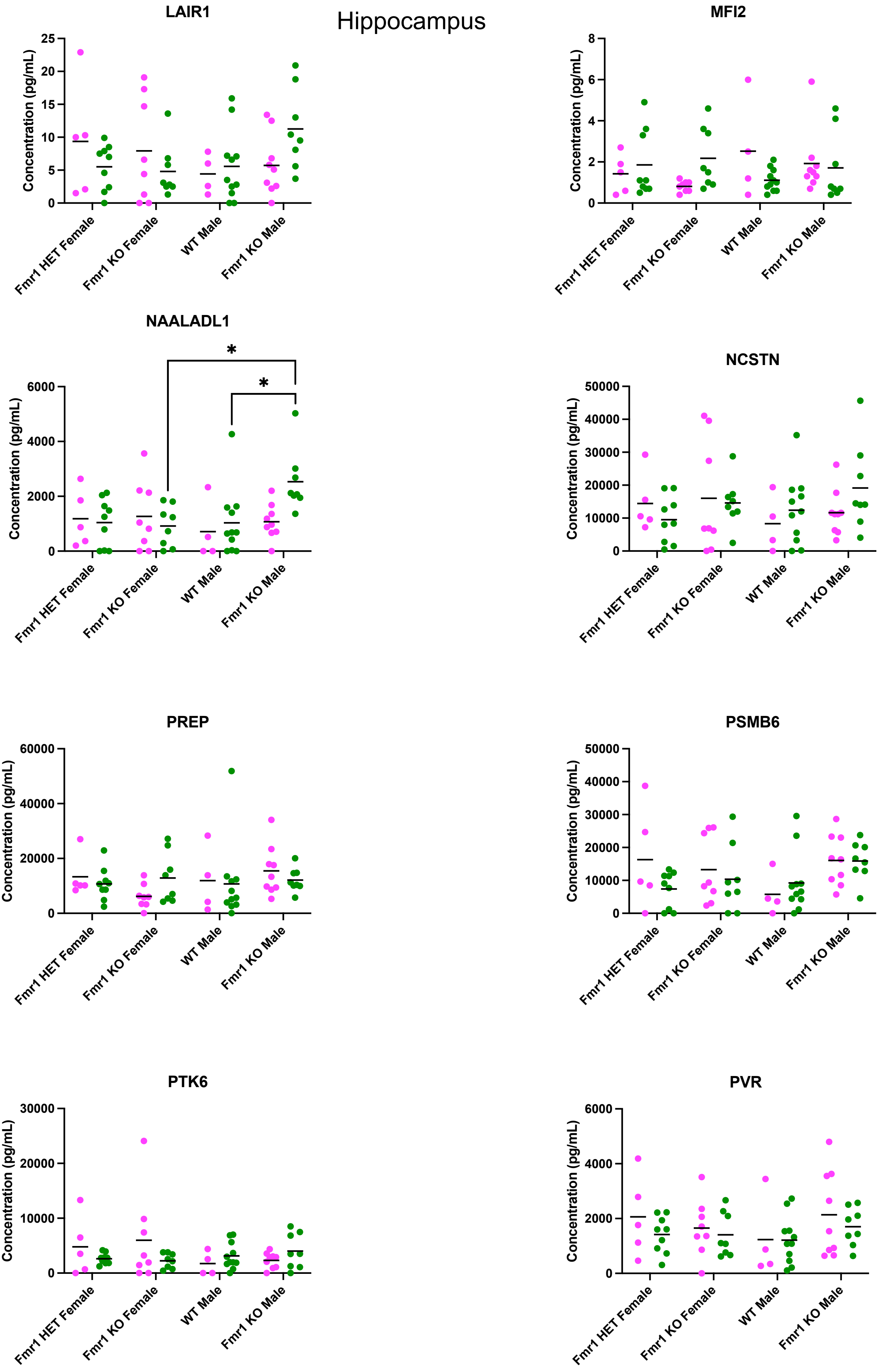

Hippocampus
LAIR1
MFI2
NAALADL1
NCSTN
PREP
PSMB6
PTK6
PVR
Concentration (pg/mL)
Fmr1 HET Female
Fmr1 KO Female
WT Male
Fmr1 KO Male
*
*

Hippocampus

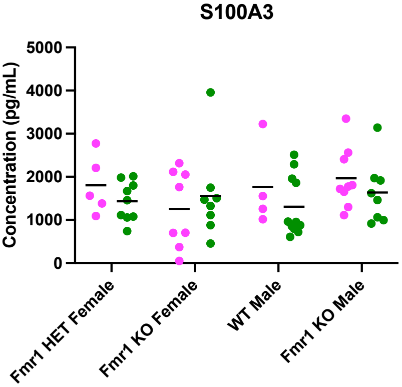

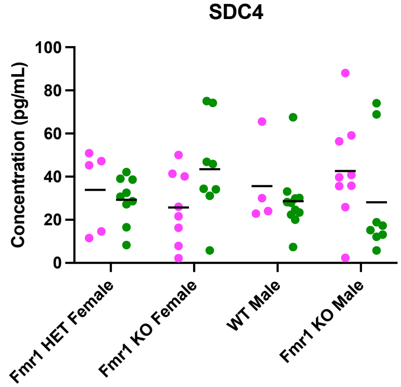

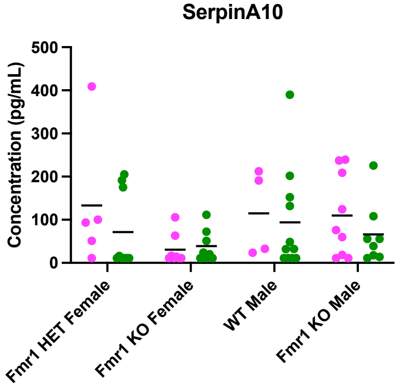

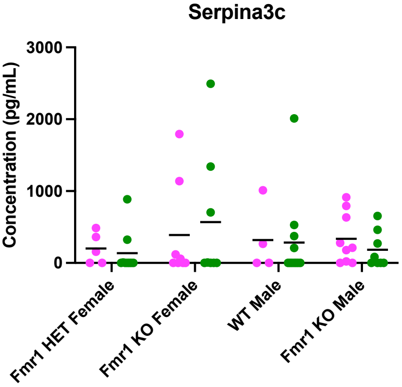

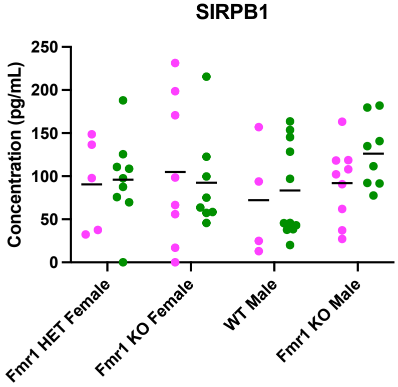

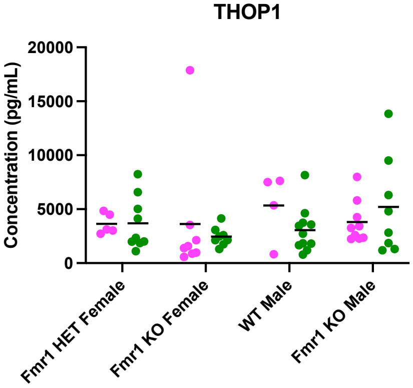

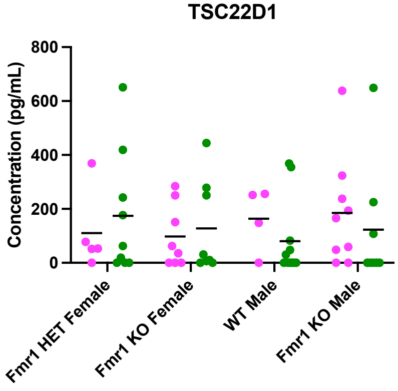

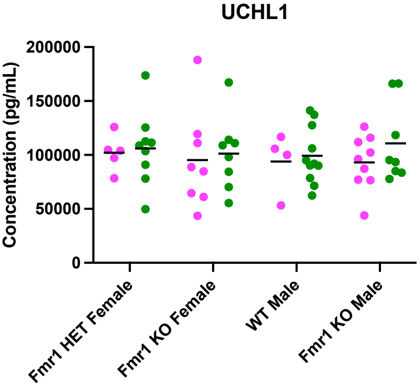

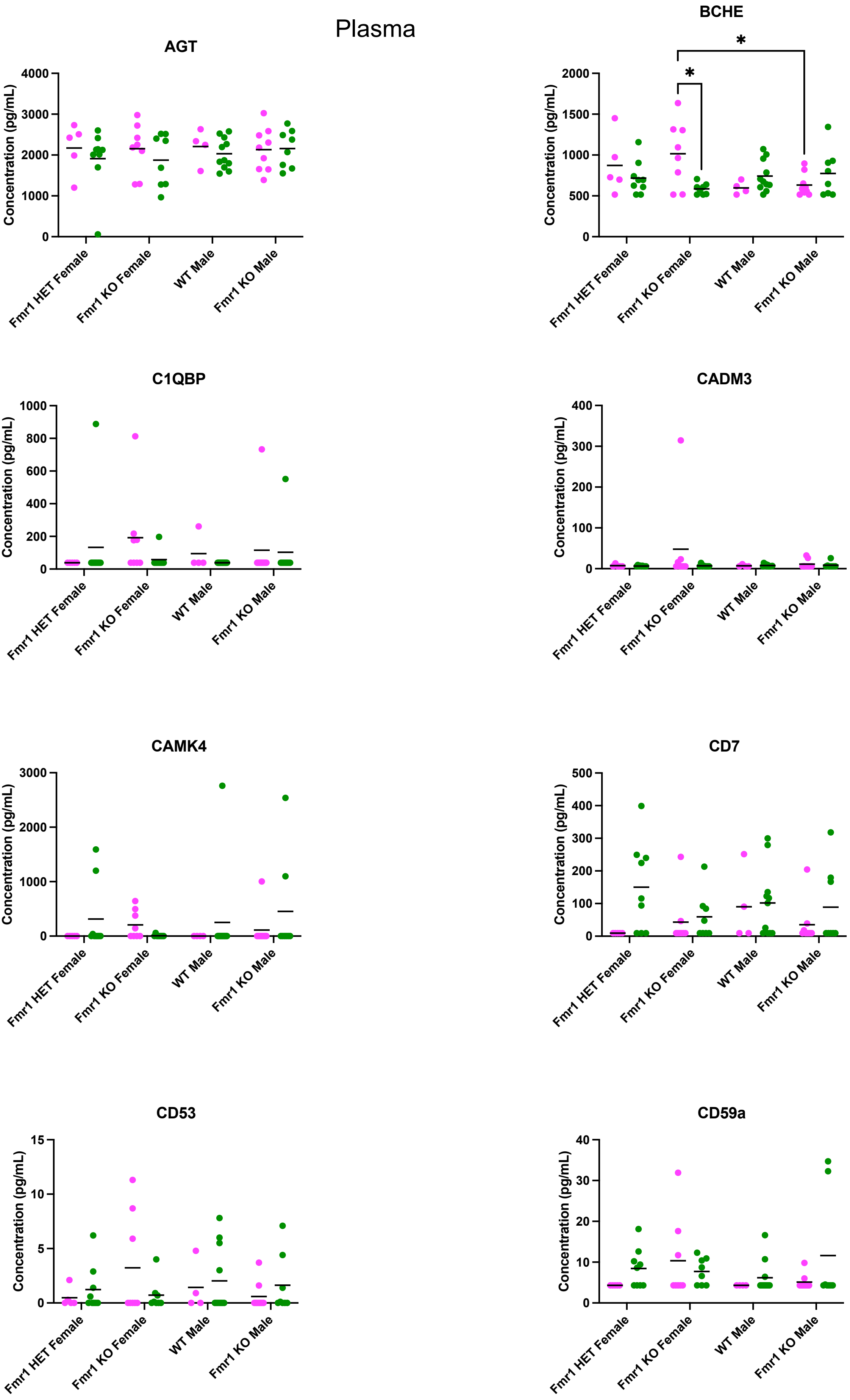
Plasma
AGT
BCHE
C1QBP
CADM3
CAMK4
CD7
CD53
CD59a
Concentration (pg/mL)
Fmr1 HET Female
Fmr1 KO Female
WT Male
Fmr1 KO Male
*
*

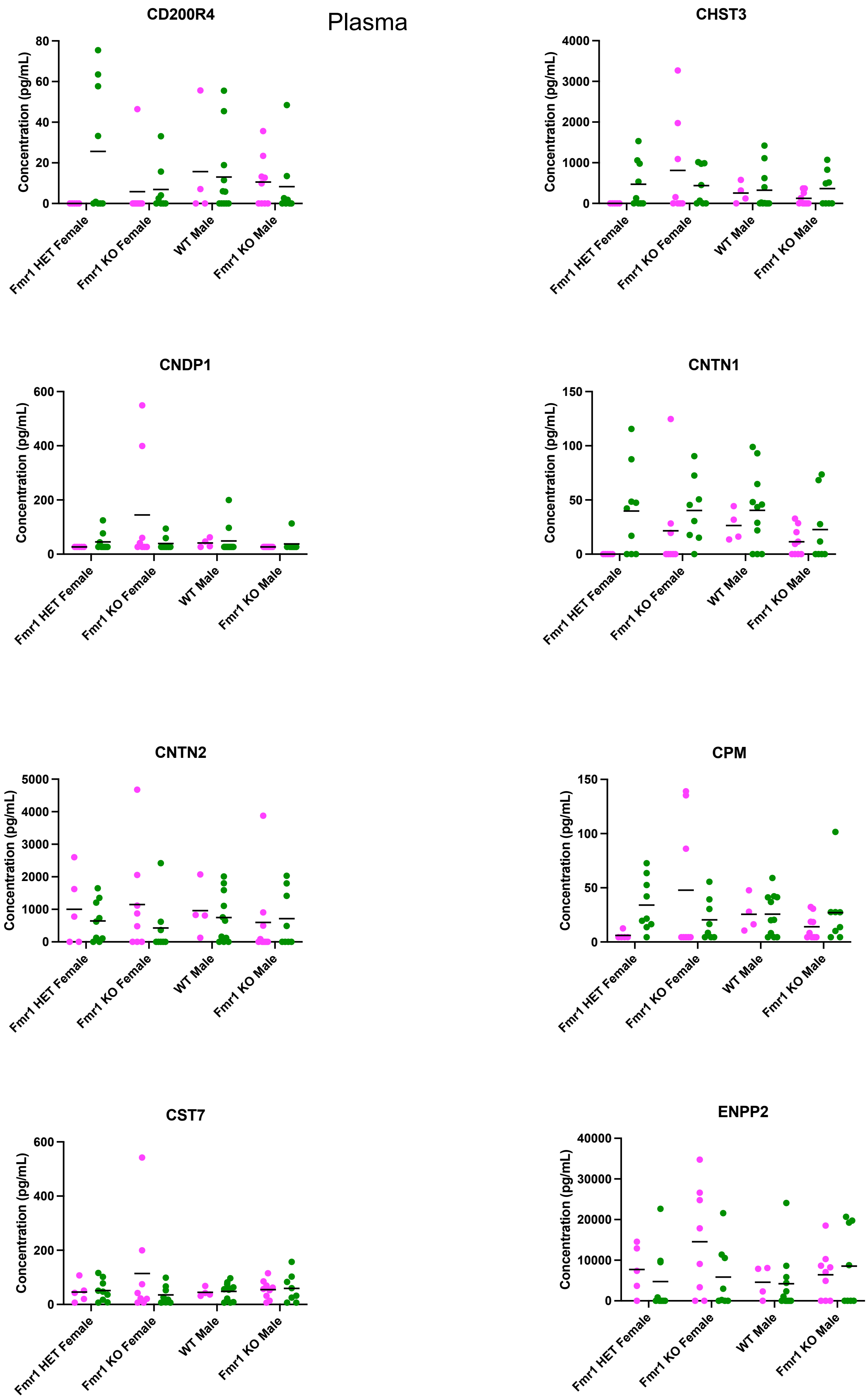
Plasma
CD200R4
CHST3
CNDP1
CNTN1
CNTN2
CPM
CST7
ENPP2
Concentration (pg/mL)
Fmr1 HET Female
Fmr1 KO Female
WT Male
Fmr1 KO Male

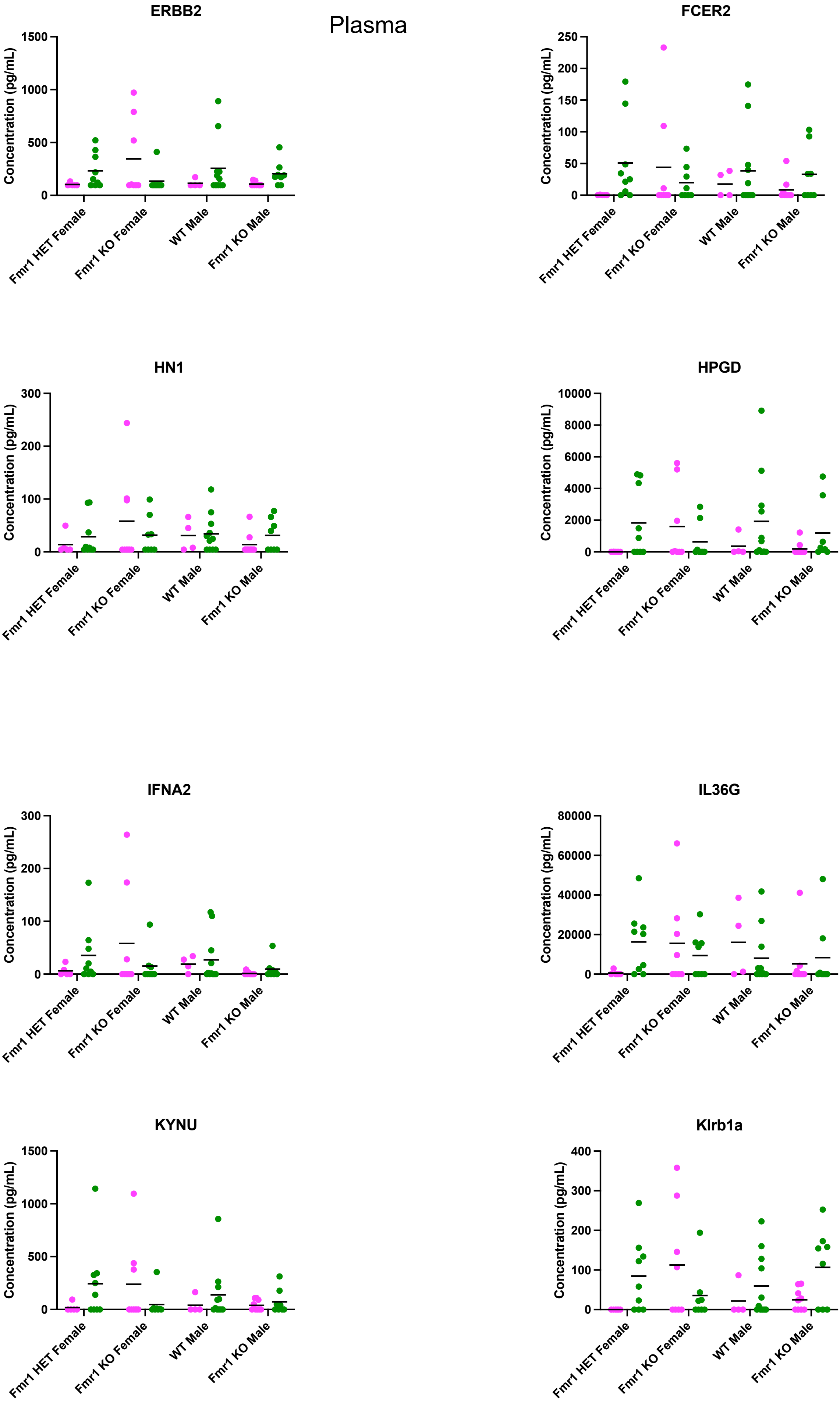
Plasma
ERBB2
FCER2
HN1
HPGD
IFNA2
IL36G
KYNU
Klrb1a
Concentration (pg/mL)
0
500
1000
1500
50
100
150
200
250
300
2000
4000
6000
8000
10000
20000
40000
60000
80000
400
Fmr1 HET Female
Fmr1 KO Female
WT Male
Fmr1 KO Male

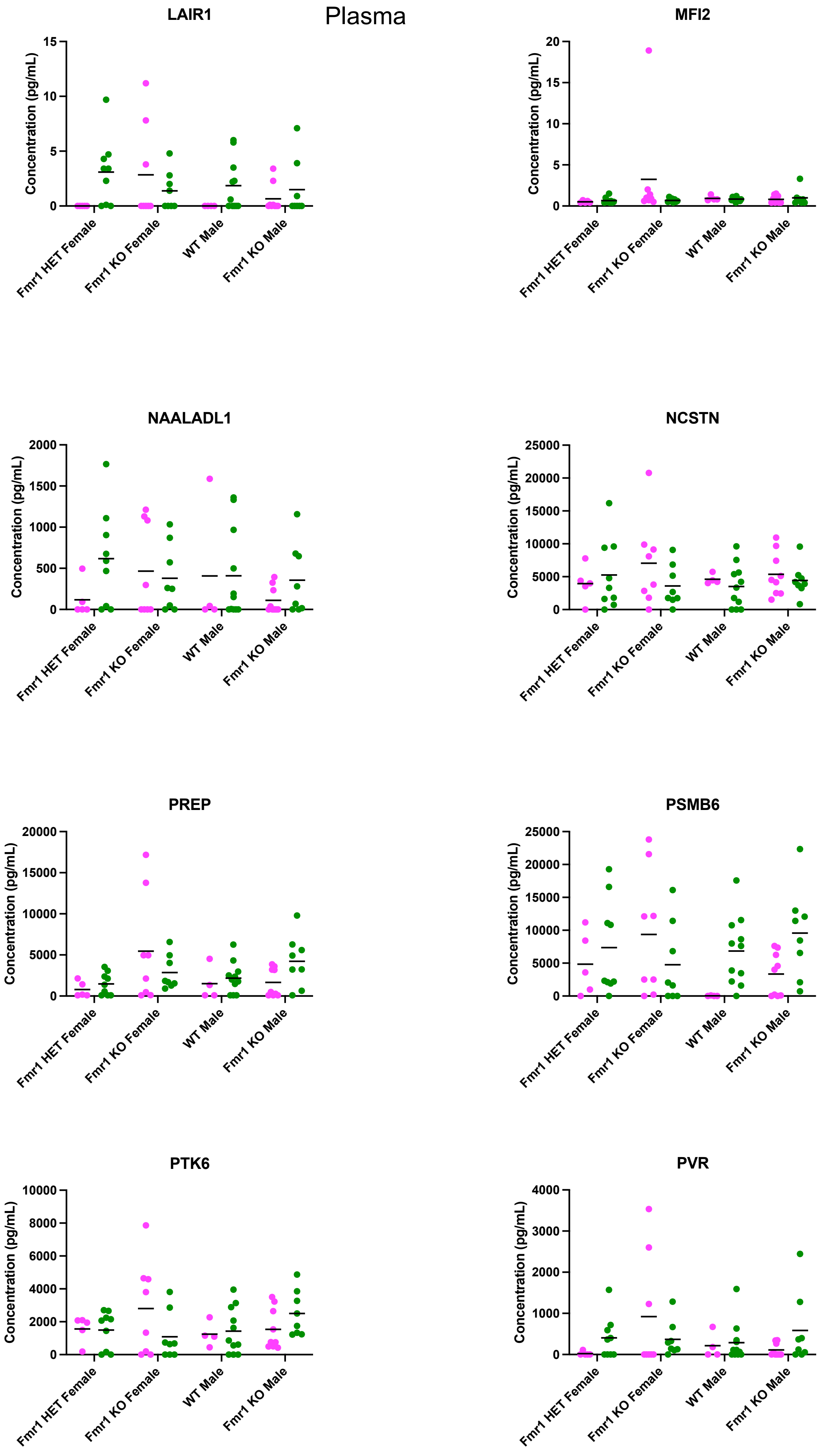
Plasma
LAIR1
MFI2
NAALADL1
NCSTN
PREP
PSMB6
PTK6
PVR
Concentration (pg/mL)
Fmr1 HET Female
Fmr1 KO Female
WT Male
Fmr1 KO Male

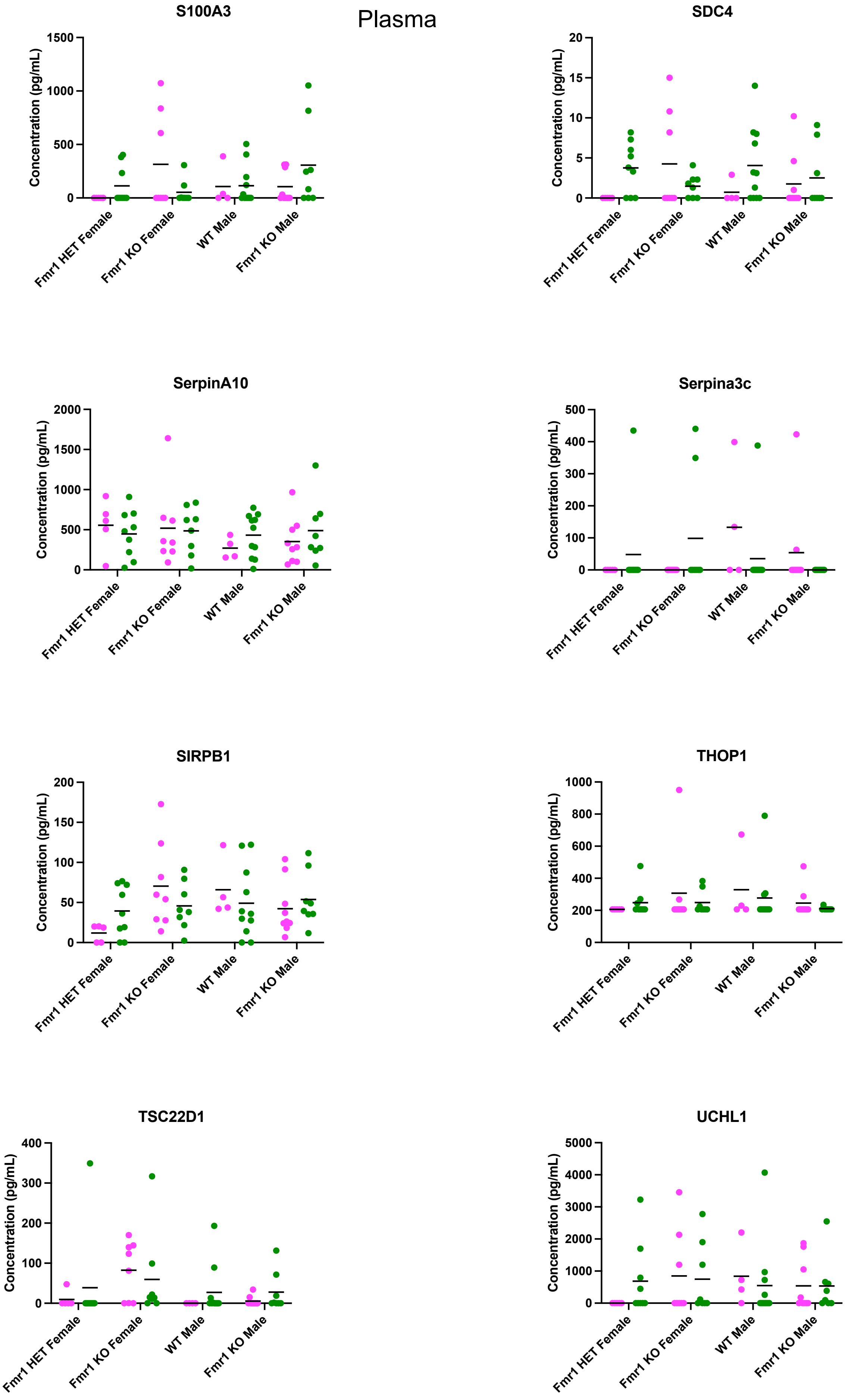
Plasma
S100A3
SDC4
SerpinA10
Serpina3c
SIRPB1
THOP1
TSC22D1
UCHL1
Concentration (pg/mL)
Fmr1 HET Female
Fmr1 KO Female
WT Male
Fmr1 KO Male
