# Supplementary material for: Soy Protein Isolate Affects Blood and Brain Biomarker Expression in a Mouse Model of Fragile X
Source: Int J Mol Sci. 2025 Jun 26;26(13):6137. doi: 10.3390/ijms26136137 (PMC12250412; doi:10.3390/ijms26136137)
Supplement: Supplementary file 1 [file ijms-26-06137-s001.zip › Supplementary File S14b Array 16 Graphs.pdf]

Supplementary File S14. Protein expression of Array 16 targets as function of *Fmr1* genotype and AIN-93G diets. Mice on AIN-93G/cas (colored pink) included n=5 *Fmr1*^{HET} female, n=8 *Fmr1*^{KO} female, n=4 WT male and n=9 *Fmr1*^{KO} male. Mice on AIN-93G/soy (colored green) included n=9 *Fmr1*^{HET} female, n=8 *Fmr1*^{KO} female, n=11 WT male and n=8 *Fmr1*^{KO} male. The average concentration in cortex, hippocampus, hypothalamus and plasma in pg/mL was plotted versus genotype. Statistics were determined by 2-way ANOVA and Tukey's multiple comparison tests denoted by $p < 0.05$ (*), $p < 0.01$ (**), $p < 0.001$ (***) and $p < 0.0001$ (****).

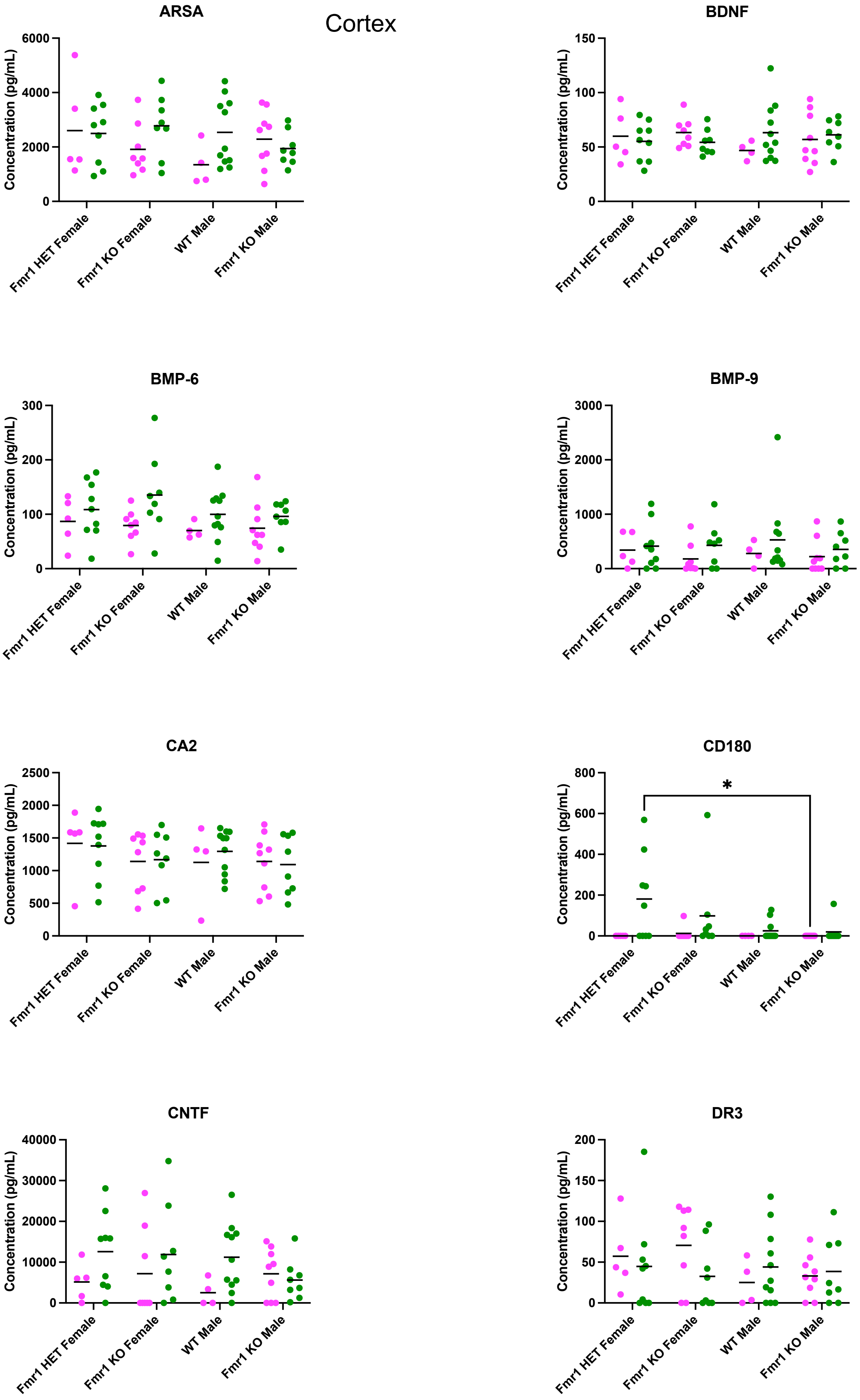
Cortex
ARSA
BDNF
BMP-6
BMP-9
CA2
CD180
CNTF
DR3
Concentration (pg/mL)
Fmr1 HET Female
Fmr1 KO Female
WT Male
Fmr1 KO Male
*

Cortex

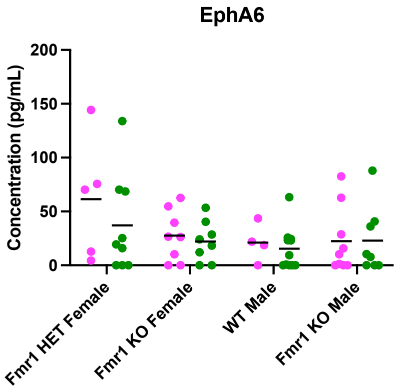
EphA6
Concentration (pg/mL)
0
50
100
150
200
Fmr1 HET Female
Fmr1 KO Female
WT Male
Fmr1 KO Male

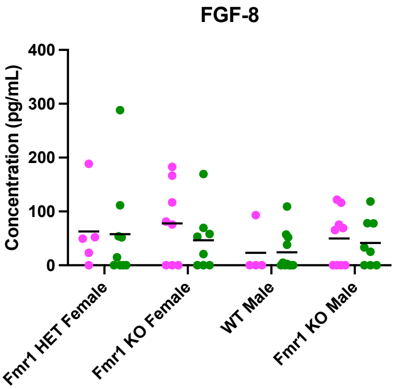
FGF-8
Concentration (pg/mL)
0
100
200
300
400
Fmr1 HET Female
Fmr1 KO Female
WT Male
Fmr1 KO Male

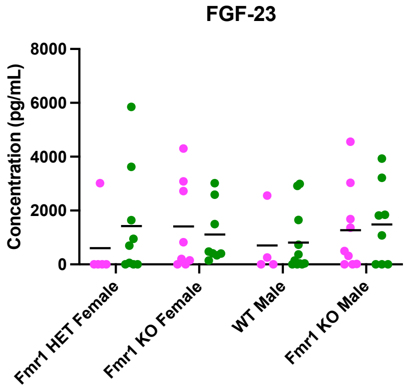
FGF-23
Concentration (pg/mL)
0
2000
4000
6000
8000
Fmr1 HET Female
Fmr1 KO Female
WT Male
Fmr1 KO Male

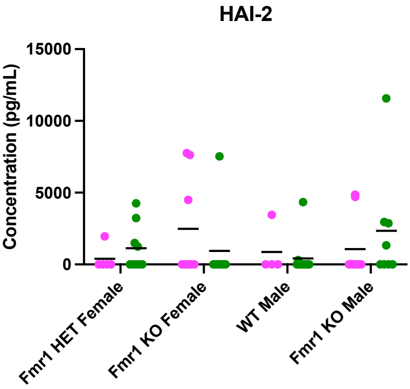
HAI-2
Concentration (pg/mL)
0
5000
10000
15000
Fmr1 HET Female
Fmr1 KO Female
WT Male
Fmr1 KO Male

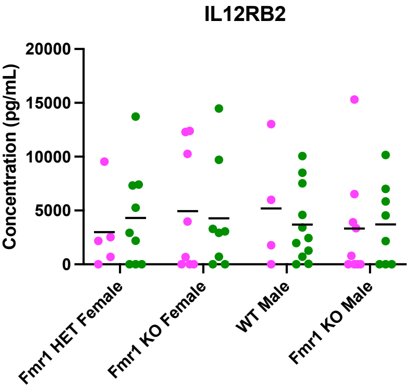
IL12RB2
Concentration (pg/mL)
0
5000
10000
15000
20000
Fmr1 HET Female
Fmr1 KO Female
WT Male
Fmr1 KO Male

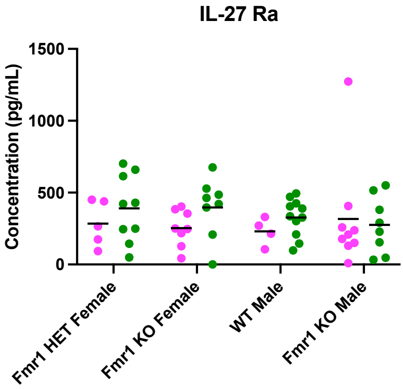
IL-27 Ra
Concentration (pg/mL)
0
500
1000
1500
Fmr1 HET Female
Fmr1 KO Female
WT Male
Fmr1 KO Male

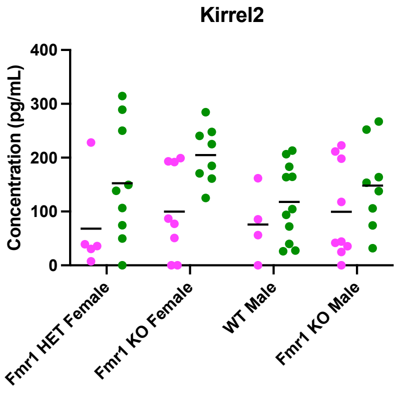
Kirrel2
Concentration (pg/mL)
0
100
200
300
400
Fmr1 HET Female
Fmr1 KO Female
WT Male
Fmr1 KO Male

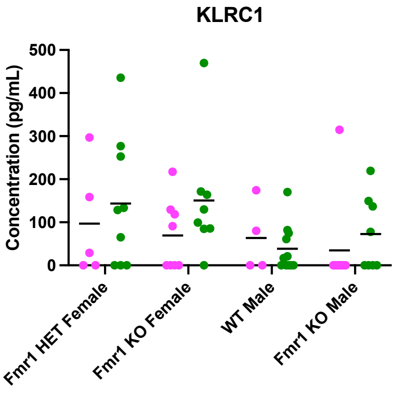
KLRC1
Concentration (pg/mL)
0
100
200
300
400
500
Fmr1 HET Female
Fmr1 KO Female
WT Male
Fmr1 KO Male

Cortex

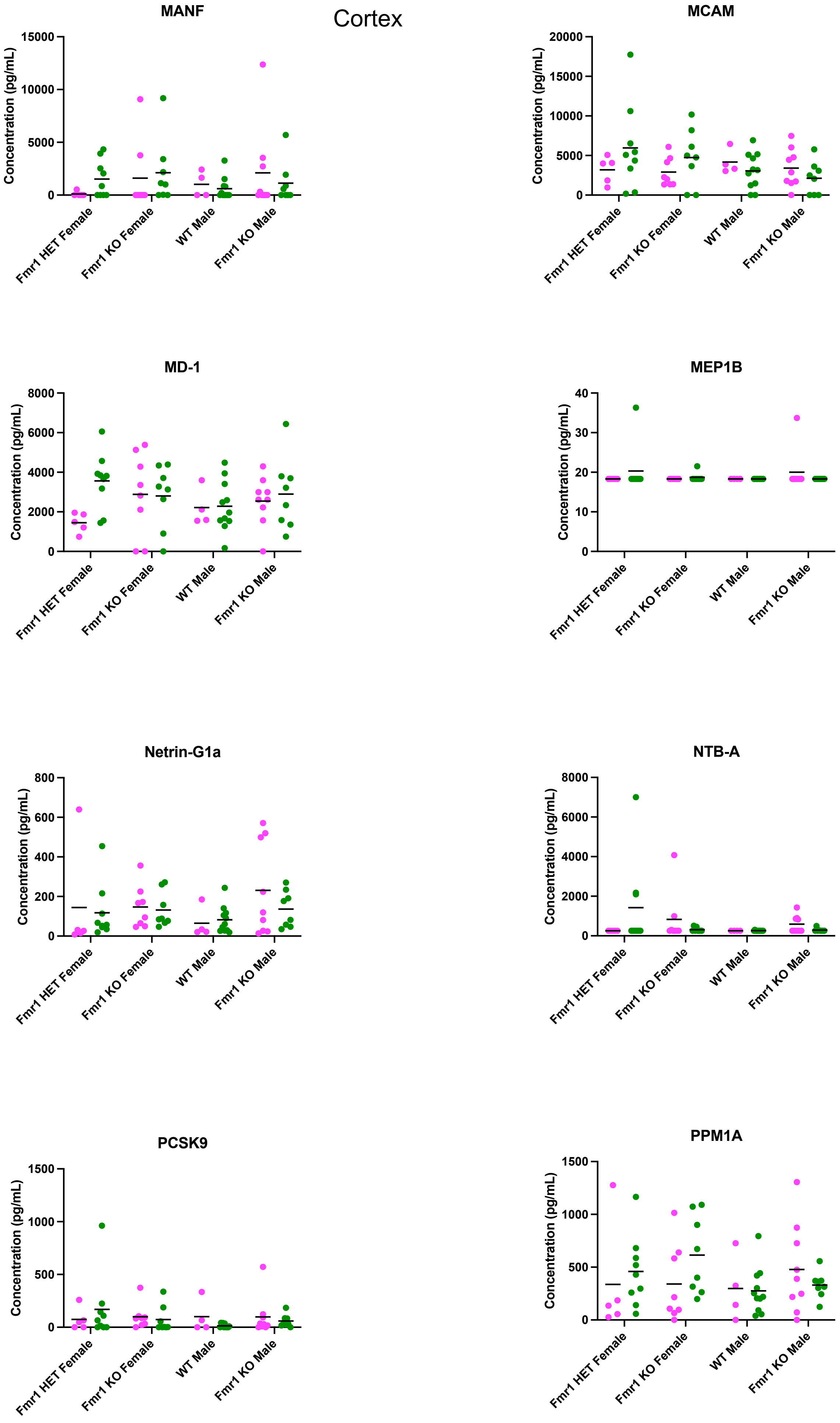
MANF
MCAM
MD-1
MEP1B
Netrin-G1a
NTB-A
PCSK9
PPM1A
Concentration (pg/mL)
Fmr1 HET Female
Fmr1 KO Female
WT Male
Fmr1 KO Male

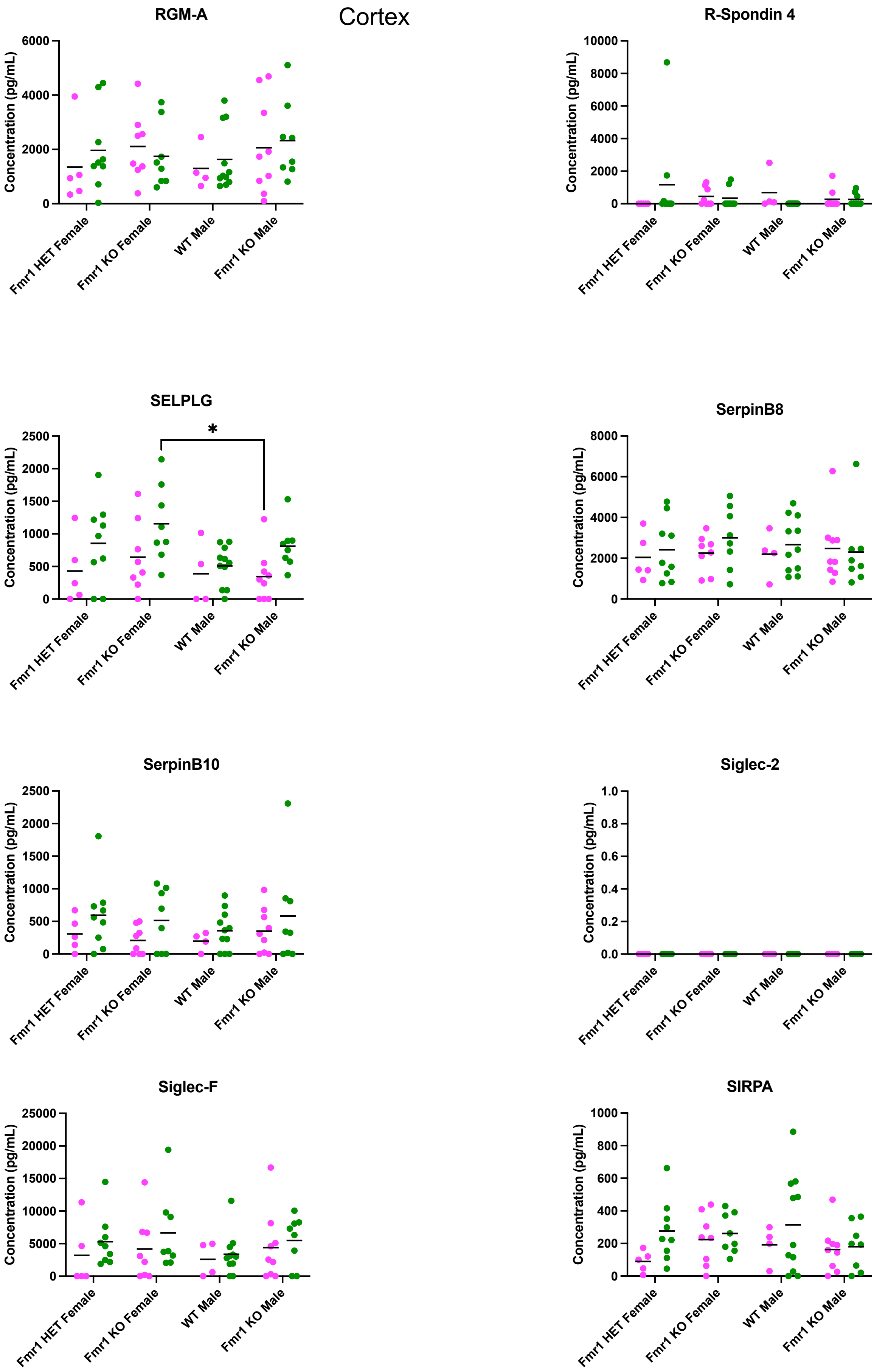

Cortex
RGM-A
R-Spondin 4
SELPLG
SerpinB8
SerpinB10
Siglec-2
Siglec-F
SIRPA
Concentration (pg/mL)
6000
4000
2000
0
10000
8000
6000
4000
2000
0
2500
2000
1500
1000
500
0
*
8000
6000
4000
2000
0
2500
2000
1500
1000
500
0
1.0
0.8
0.6
0.4
0.2
0.0
25000
20000
15000
10000
5000
0
1000
800
600
400
200
0
Fmr1 HET Female
Fmr1 KO Female
WT Male
Fmr1 KO Male

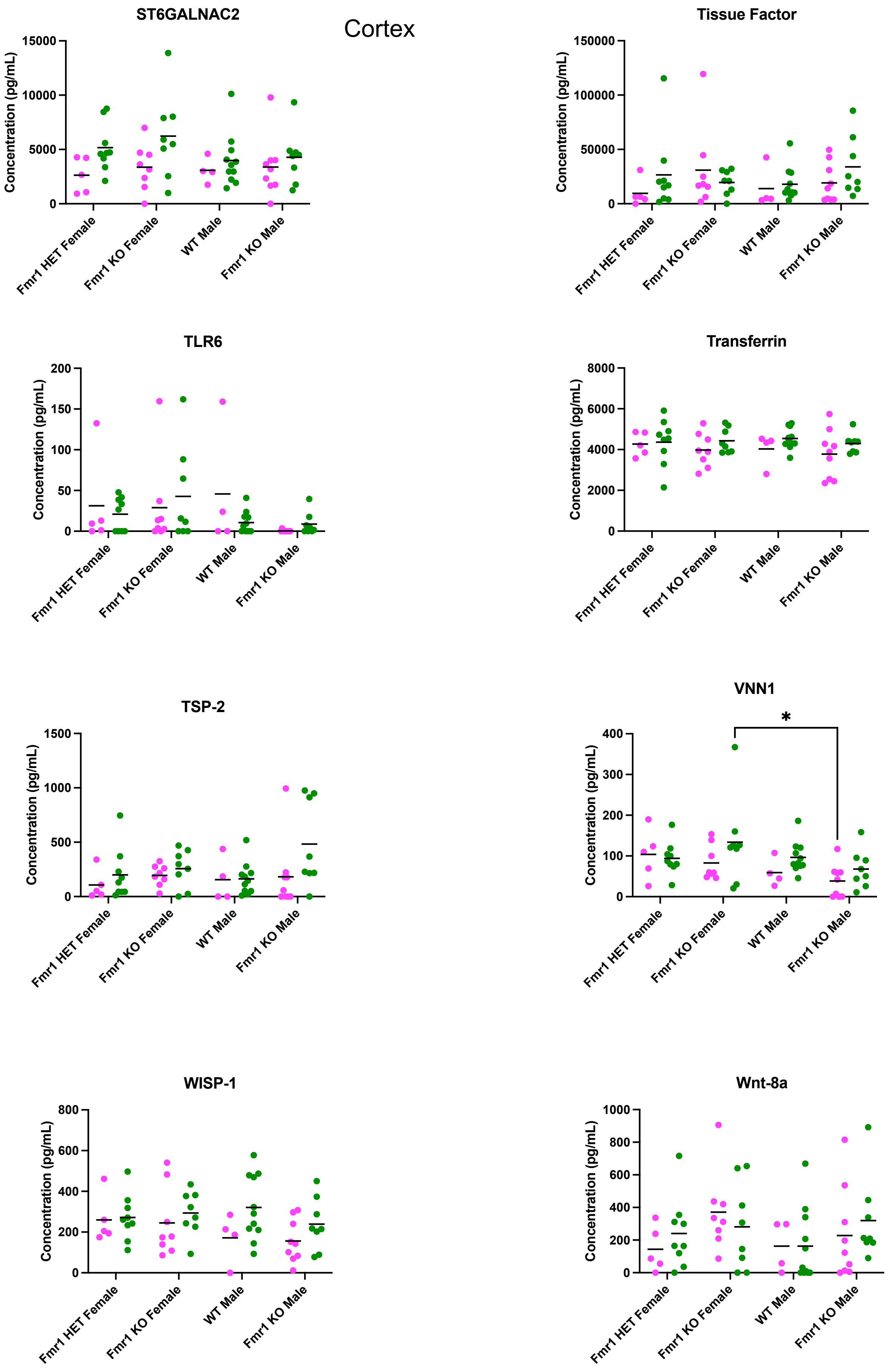

Cortex
ST6GALNAC2
Tissue Factor
TLR6
Transferrin
TSP-2
VNN1
*
WISP-1
Wnt-8a
Concentration (pg/mL)
Fmr1 HET Female
Fmr1 KO Female
WT Male
Fmr1 KO Male

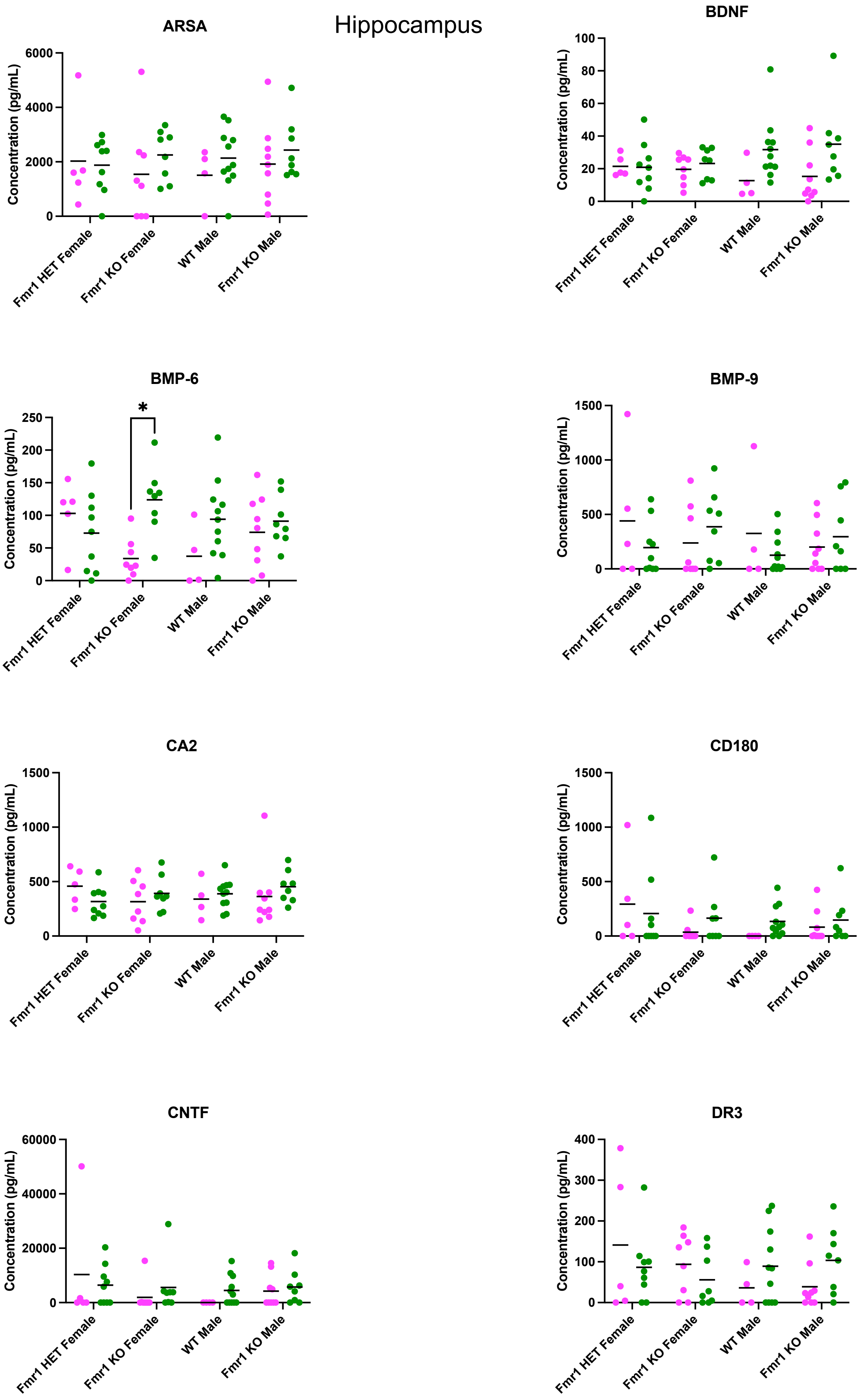
Hippocampus
ARSA
Concentration (pg/mL)
6000
4000
2000
0
Fmr1 HET Female
Fmr1 KO Female
WT Male
Fmr1 KO Male
BDNF
Concentration (pg/mL)
100
80
60
40
20
0
Fmr1 HET Female
Fmr1 KO Female
WT Male
Fmr1 KO Male
BMP-6
Concentration (pg/mL)
250
200
150
100
50
0
*
Fmr1 HET Female
Fmr1 KO Female
WT Male
Fmr1 KO Male
BMP-9
Concentration (pg/mL)
1500
1000
500
0
Fmr1 HET Female
Fmr1 KO Female
WT Male
Fmr1 KO Male
CA2
Concentration (pg/mL)
1500
1000
500
0
Fmr1 HET Female
Fmr1 KO Female
WT Male
Fmr1 KO Male
CD180
Concentration (pg/mL)
1500
1000
500
0
Fmr1 HET Female
Fmr1 KO Female
WT Male
Fmr1 KO Male
CNTF
Concentration (pg/mL)
60000
40000
20000
0
Fmr1 HET Female
Fmr1 KO Female
WT Male
Fmr1 KO Male
DR3
Concentration (pg/mL)
400
300
200
100
0
Fmr1 HET Female
Fmr1 KO Female
WT Male
Fmr1 KO Male

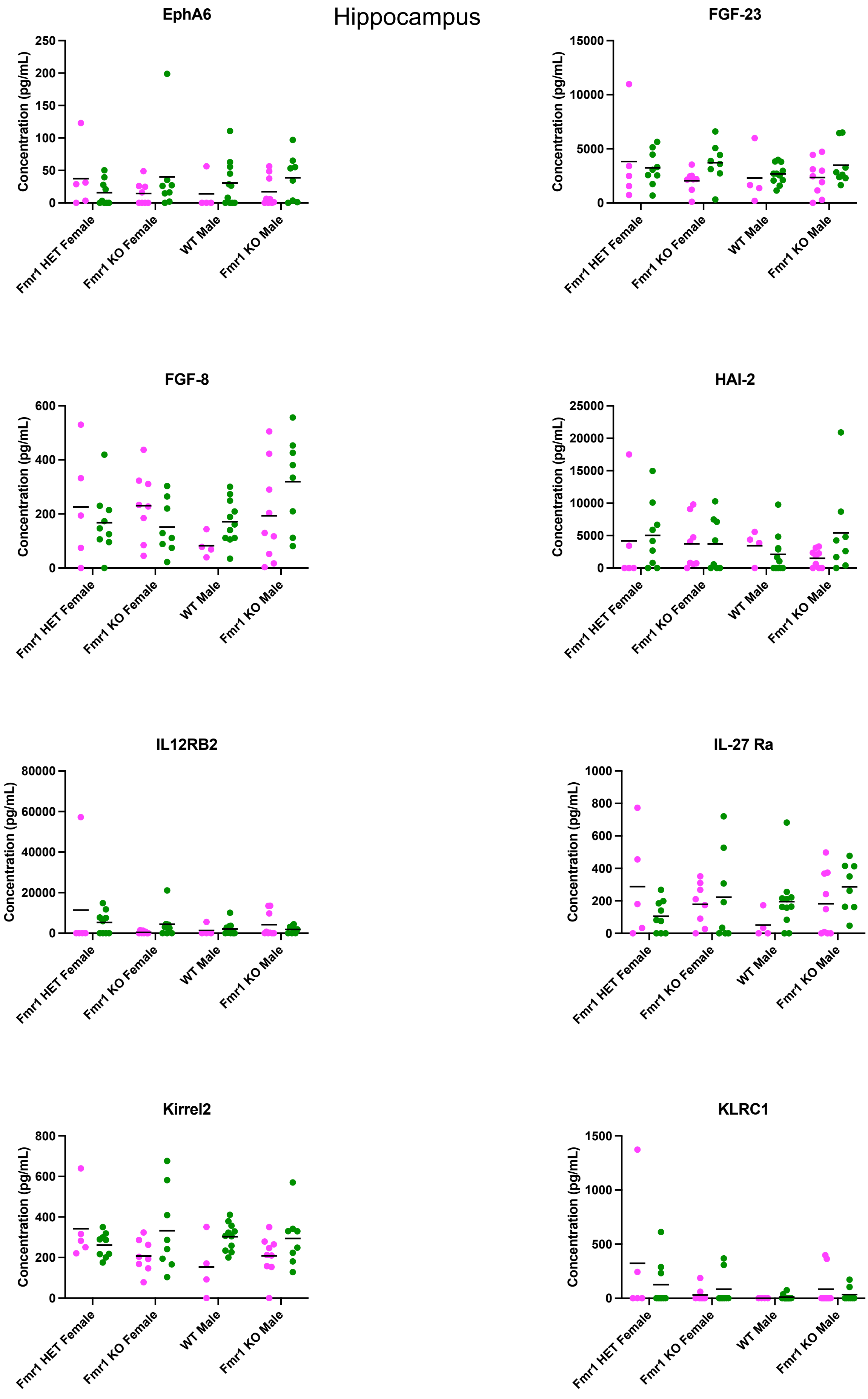
Hippocampus
EphA6
FGF-23
FGF-8
HAI-2
IL12RB2
IL-27 Ra
Kirrel2
KLRC1
Concentration (pg/mL)
Fmr1 HET Female
Fmr1 KO Female
WT Male
Fmr1 KO Male
0
50
100
150
200
250
5000
10000
15000
200
400
600
20000
25000
40000
60000
80000
800
1000
500
1500

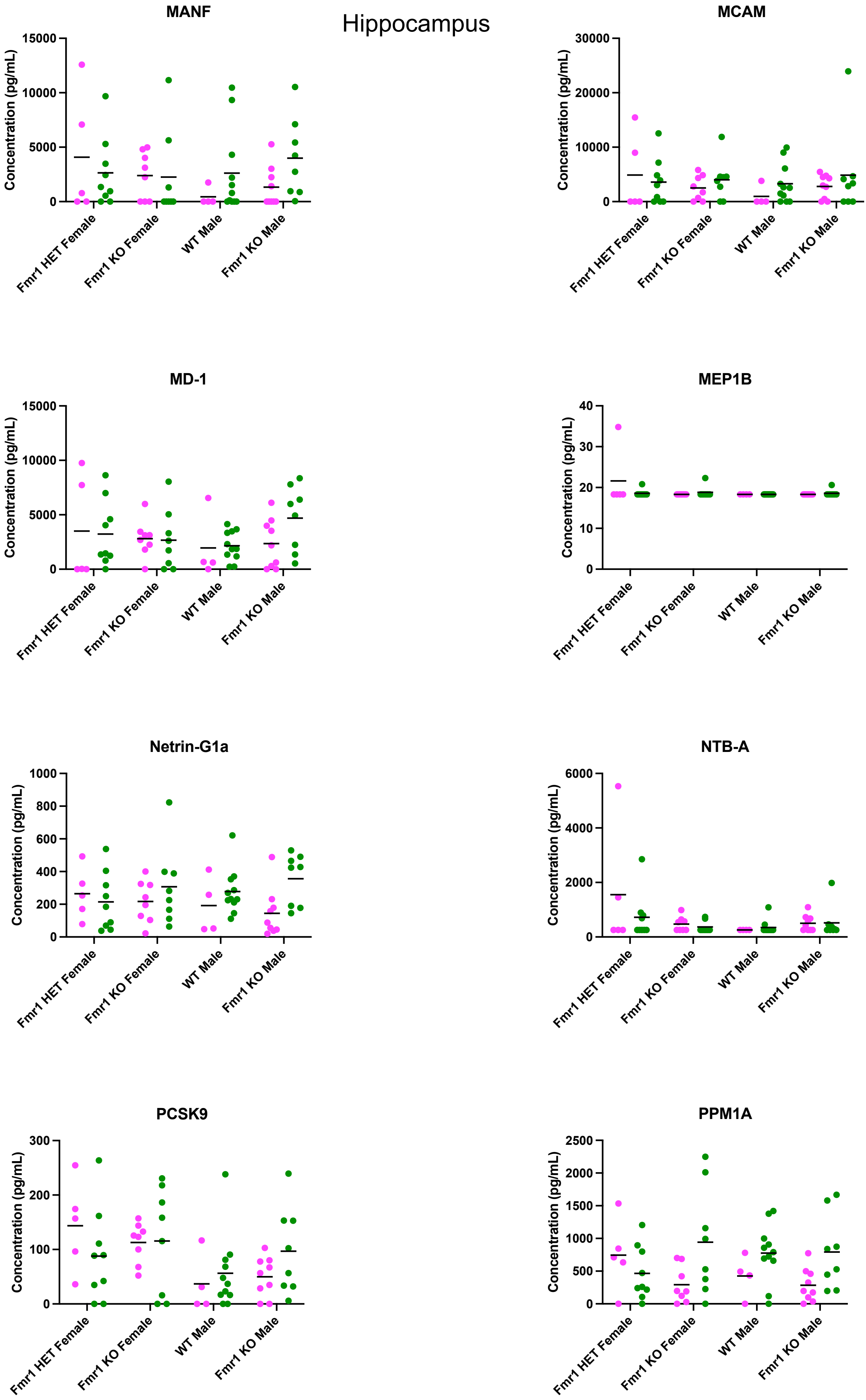
Hippocampus
MANF
MCAM
MD-1
MEP1B
Netrin-G1a
NTB-A
PCSK9
PPM1A
Concentration (pg/mL)
Fmr1 HET Female
Fmr1 KO Female
WT Male
Fmr1 KO Male
0
5000
10000
15000
20000
30000
10
20
30
40
200
400
600
800
1000
2000
4000
6000
100
300
500
1500
2500

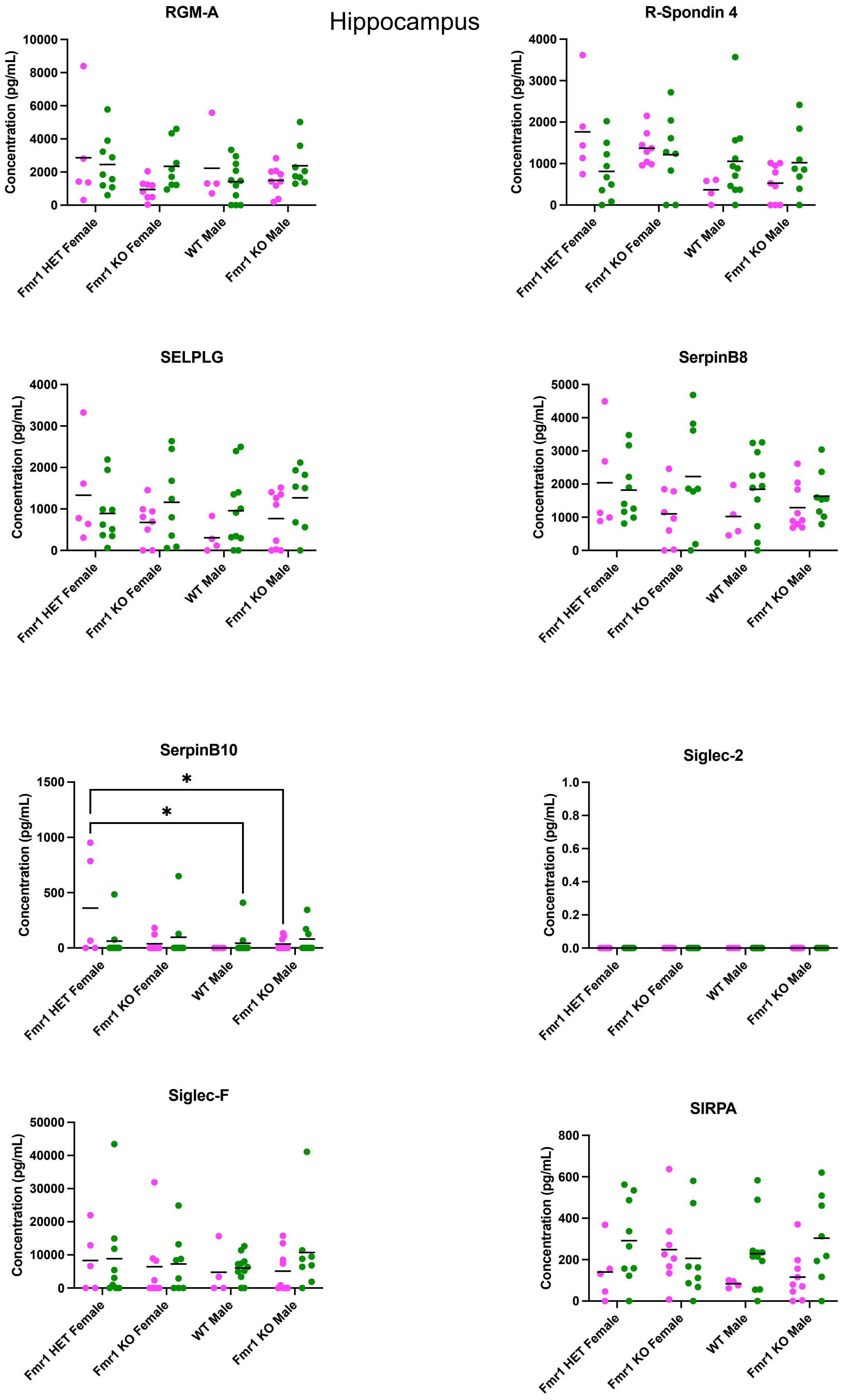
Hippocampus
RGM-A
R-Spondin 4
SELPLG
SerpinB8
SerpinB10
Siglec-2
Siglec-F
SIRPA
Concentration (pg/mL)
Fmr1 HET Female
Fmr1 KO Female
WT Male
Fmr1 KO Male
*
*

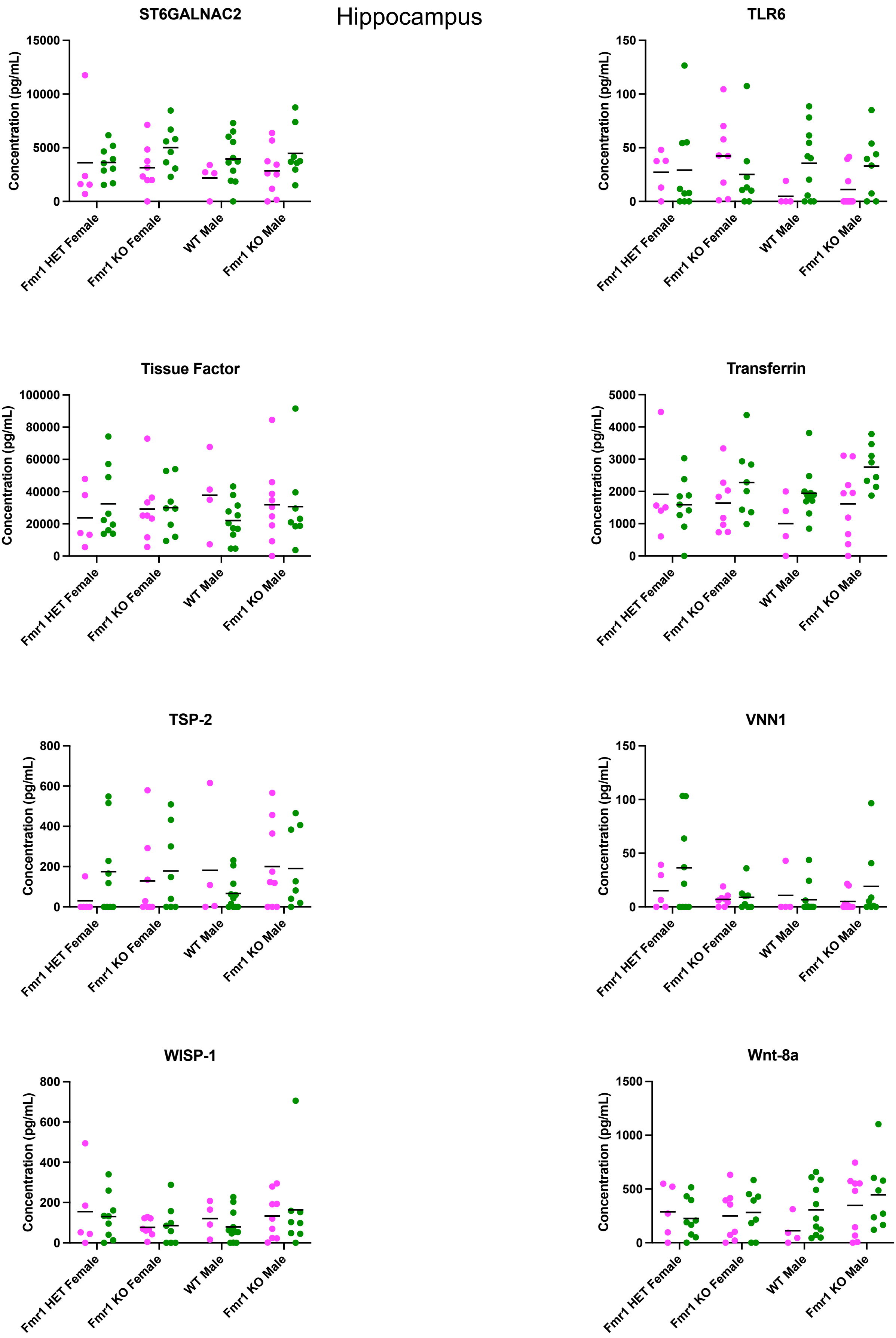
Hippocampus
ST6GALNAC2
TLR6
Tissue Factor
Transferrin
TSP-2
VNN1
WISP-1
Wnt-8a
Concentration (pg/mL)
Fmr1 HET Female
Fmr1 KO Female
WT Male
Fmr1 KO Male

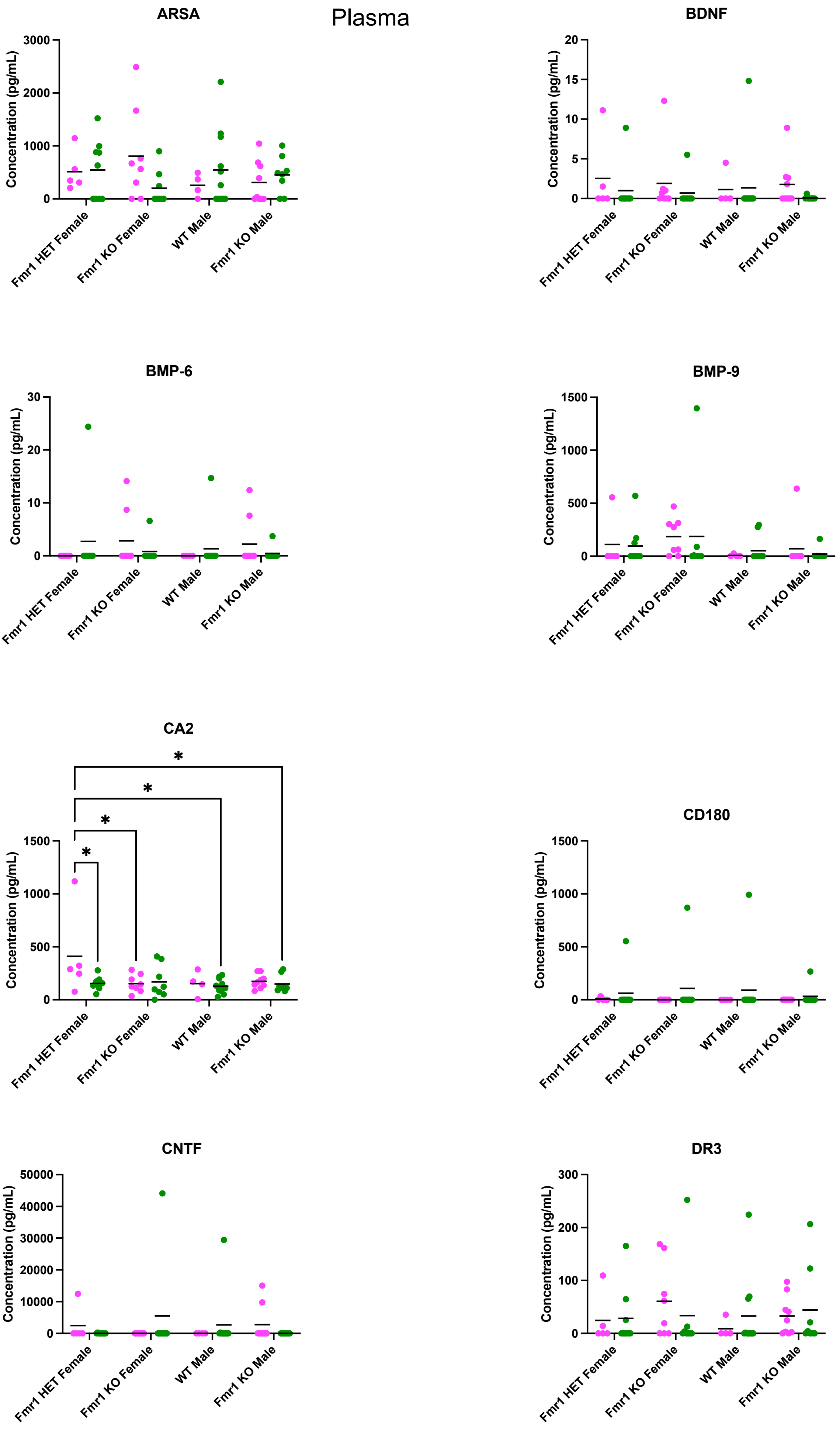
Plasma
ARSA
BDNF
BMP-6
BMP-9
CA2
CD180
CNTF
DR3
Concentration (pg/mL)
Fmr1 HET Female
Fmr1 KO Female
WT Male
Fmr1 KO Male
*
*
*
*

Plasma

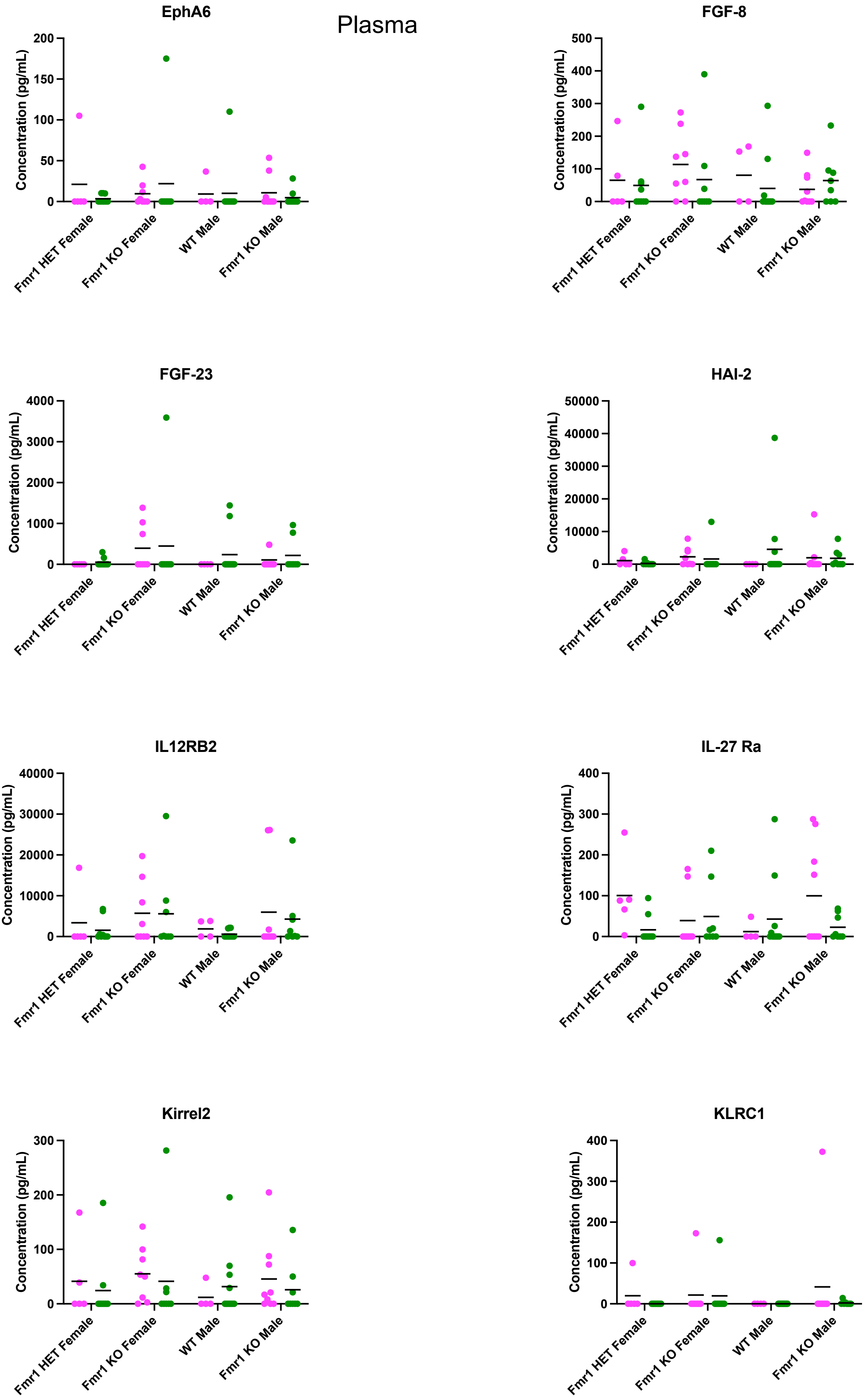
EphA6
FGF-8
FGF-23
HAI-2
IL12RB2
IL-27 Ra
Kirrel2
KLRC1
Concentration (pg/mL)
0
50
100
150
200
300
400
500
1000
2000
3000
4000
10000
20000
30000
40000
50000
Fmr1 HET Female
Fmr1 KO Female
WT Male
Fmr1 KO Male

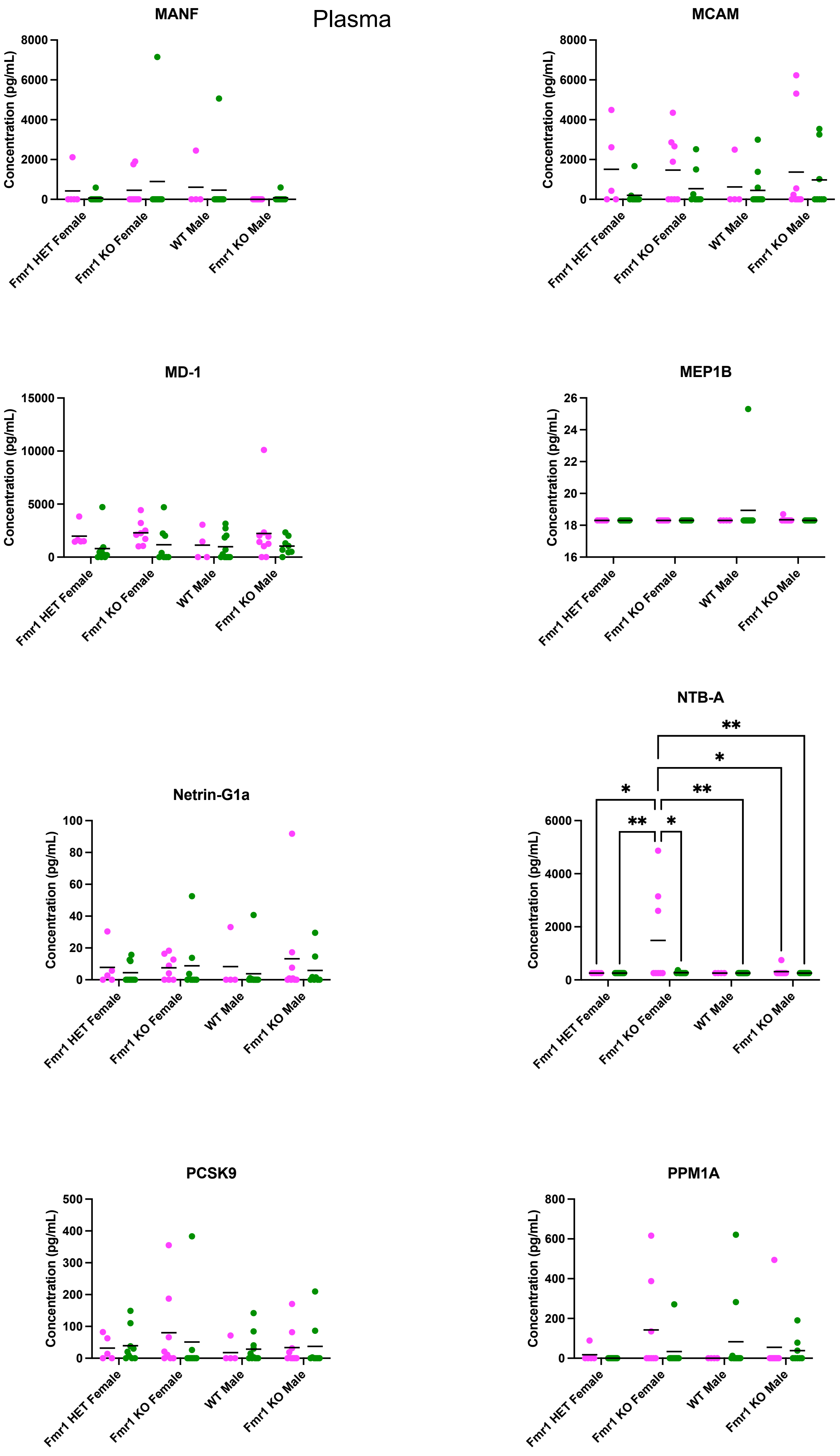
Plasma
MANF
MCAM
MD-1
MEP1B
Netrin-G1a
NTB-A
PCSK9
PPM1A
Concentration (pg/mL)
Fmr1 HET Female
Fmr1 KO Female
WT Male
Fmr1 KO Male
*
**

Plasma

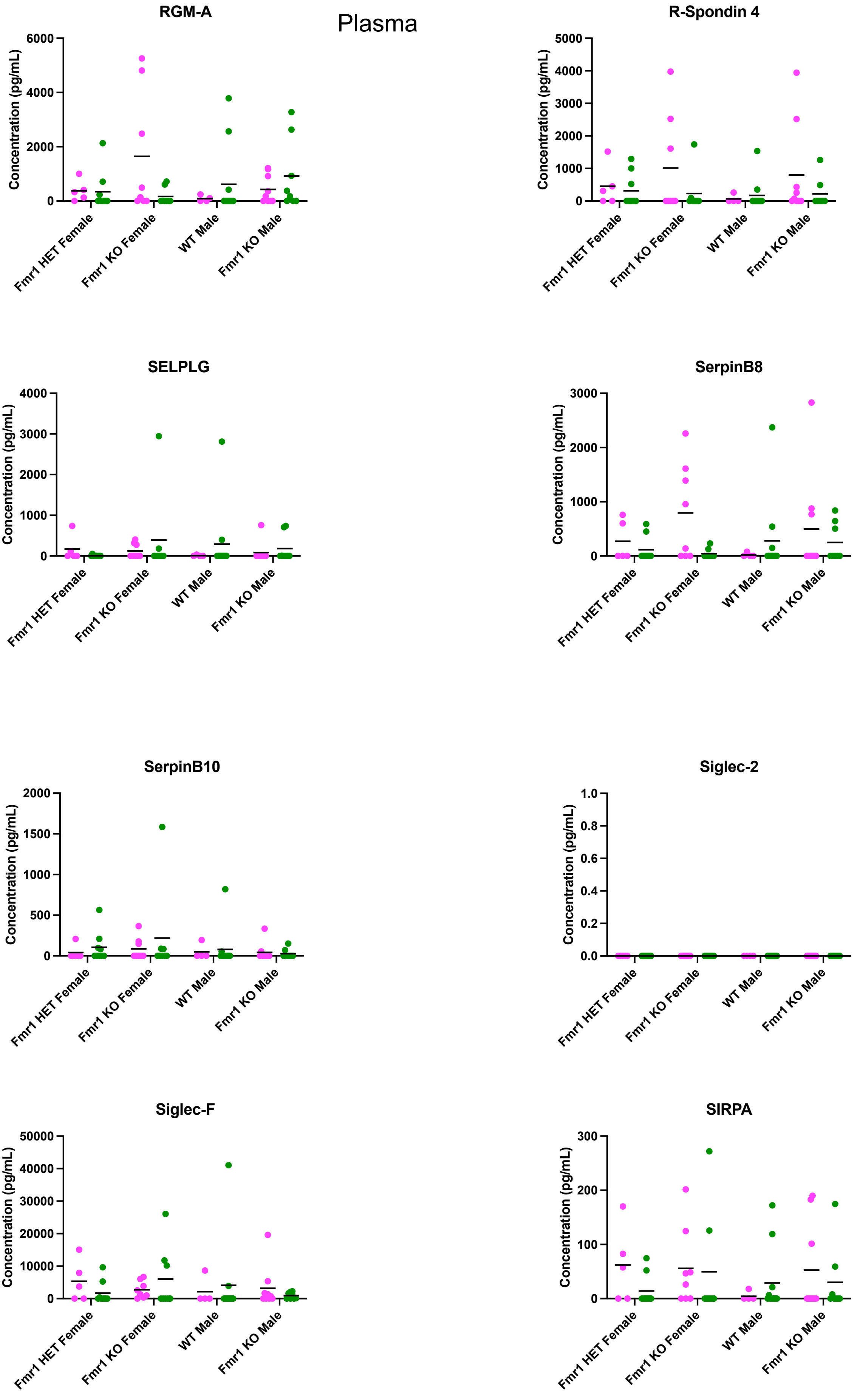
RGM-A
R-Spondin 4
SELPLG
SerpinB8
SerpinB10
Siglec-2
Siglec-F
SIRPA
Concentration (pg/mL)
Fmr1 HET Female
Fmr1 KO Female
WT Male
Fmr1 KO Male

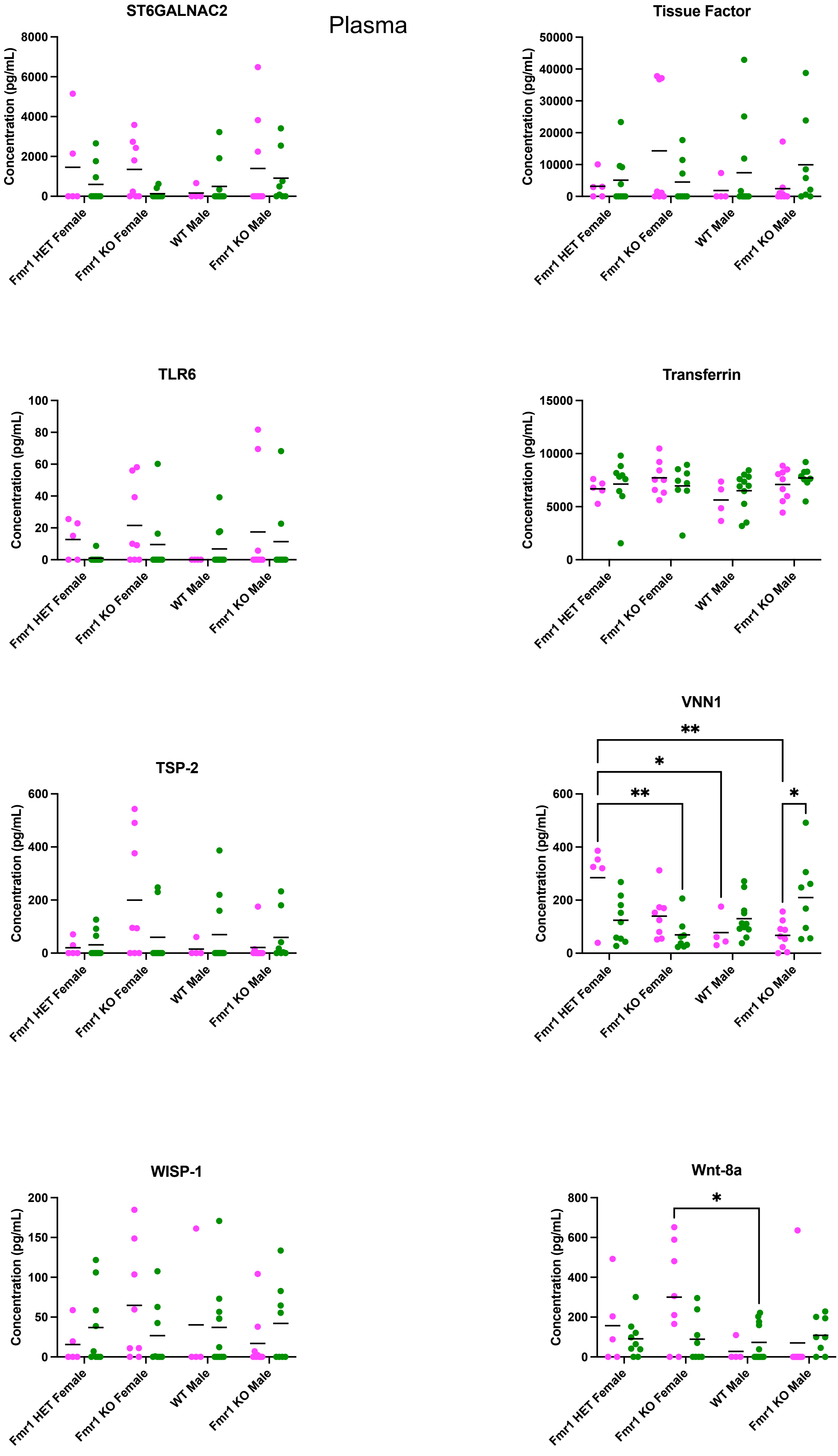
Plasma
ST6GALNAC2
Tissue Factor
TLR6
Transferrin
TSP-2
VNN1
WISP-1
Wnt-8a
Concentration (pg/mL)
Fmr1 HET Female
Fmr1 KO Female
WT Male
Fmr1 KO Male
8000
6000
4000
2000
0
50000
40000
30000
20000
10000
100
80
60
40
20
15000
10000
5000
600
400
200
150
50
800
**
*
