# Supplementary material for: Soy Protein Isolate Affects Blood and Brain Biomarker Expression in a Mouse Model of Fragile X
Source: Int J Mol Sci. 2025 Jun 26;26(13):6137. doi: 10.3390/ijms26136137 (PMC12250412; doi:10.3390/ijms26136137)
Supplement: Supplementary file 1 [file ijms-26-06137-s001.zip › Supplementary File S15b Array 17 Graphs.pdf]

Supplementary File S15. Protein expression of Array 17 targets as function of *Fmr1* genotype and AIN-93G diets. Mice on AIN-93G/cas (colored pink) included n=5 $Fmr1^{HET}$ female, n=8 $Fmr1^{KO}$ female, n=4 WT male and n=9 $Fmr1^{KO}$ male. Mice on AIN-93G/soy (colored green) included n=9 $Fmr1^{HET}$ female, n=8 $Fmr1^{KO}$ female, n=11 WT male and n=8 $Fmr1^{KO}$ male. The average concentration in cortex, hippocampus, hypothalamus and plasma in pg/mL was plotted versus genotype. Statistics were determined by 2-way ANOVA and Tukey's multiple comparison tests denoted by $p < 0.05$ (*), $p < 0.01$ (**), $p < 0.001$ (***) and $p < 0.0001$ (****).

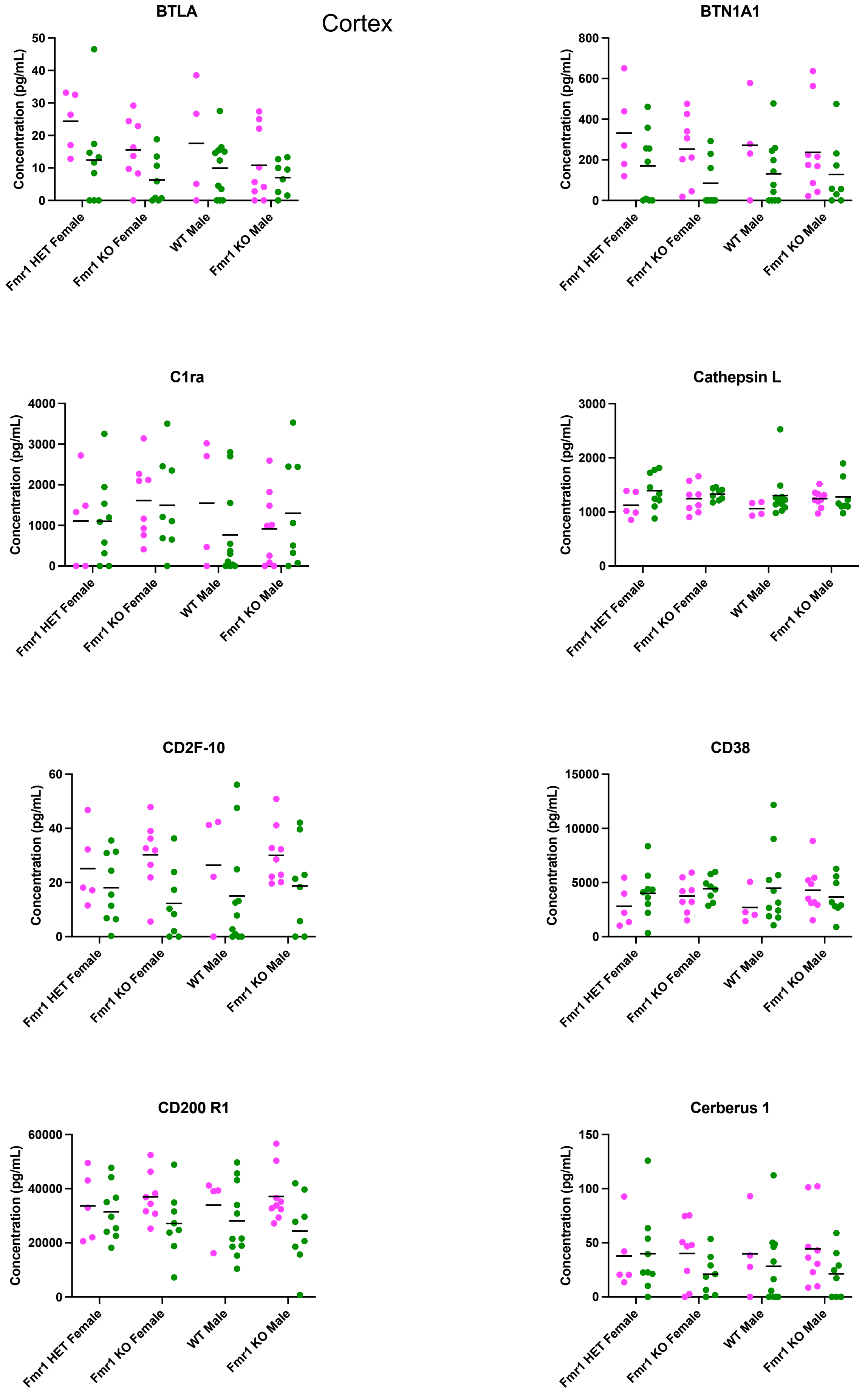
Cortex
BTLA
BTN1A1
C1ra
Cathepsin L
CD2F-10
CD38
CD200 R1
Cerberus 1
Concentration (pg/mL)
Fmr1 HET Female
Fmr1 KO Female
WT Male
Fmr1 KO Male

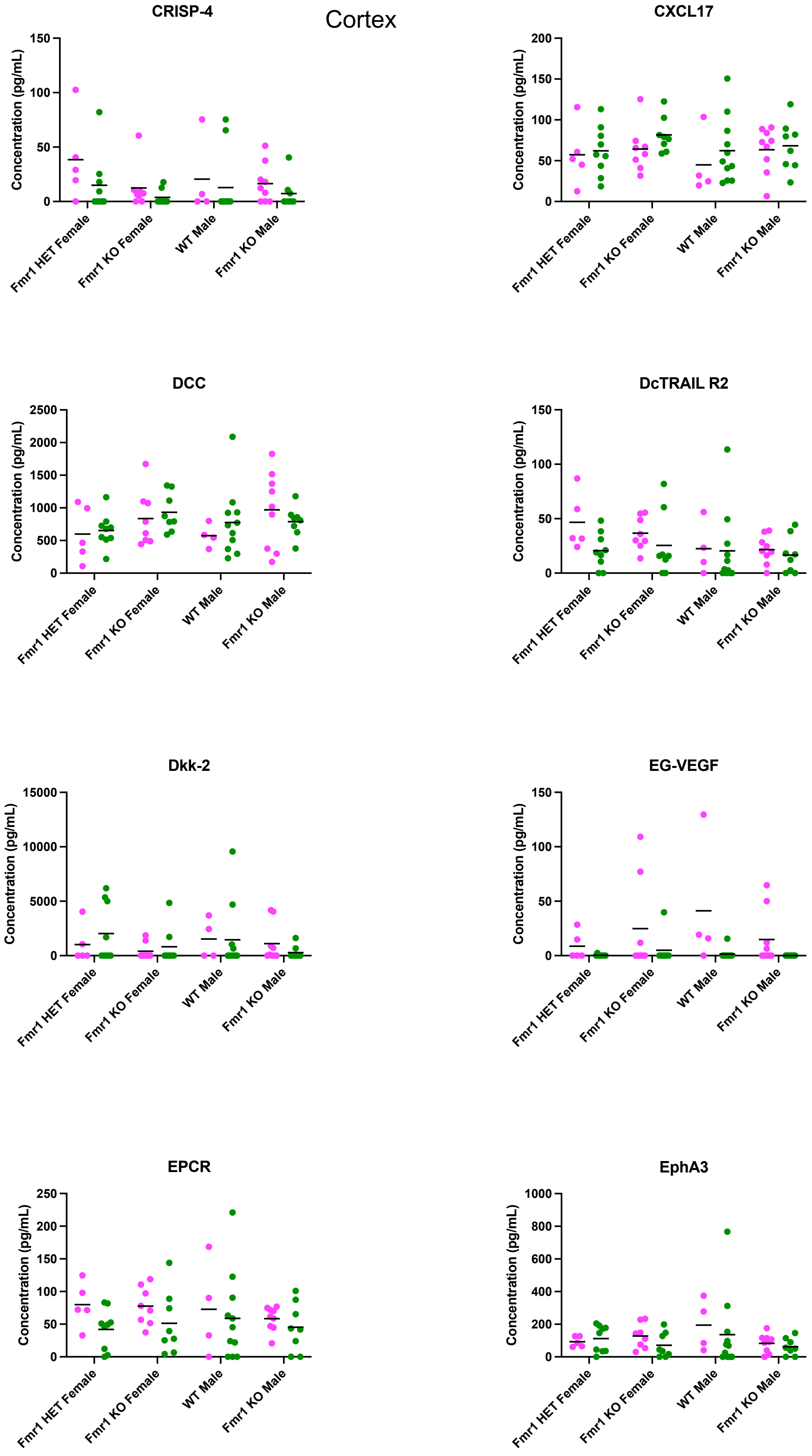
Cortex
CRISP-4
CXCL17
DCC
DcTRAIL R2
Dkk-2
EG-VEGF
EPCR
EphA3
Concentration (pg/mL)
Fmr1 HET Female
Fmr1 KO Female
WT Male
Fmr1 KO Male
0
50
100
150
200
250
400
500
600
800
1000
1500
2000
2500
5000
10000
15000

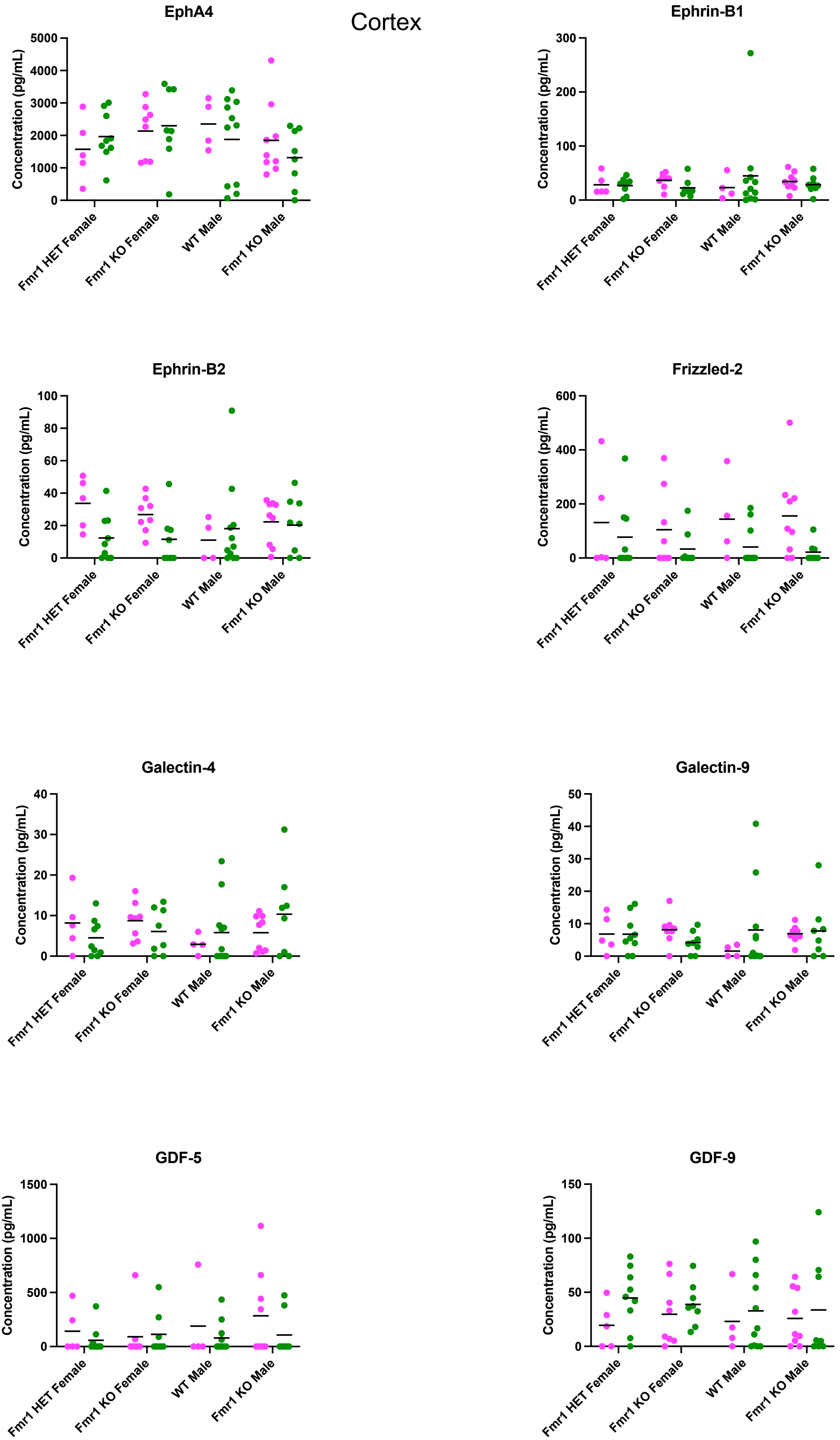
Cortex
EphA4
Ephrin-B1
Ephrin-B2
Frizzled-2
Galectin-4
Galectin-9
GDF-5
GDF-9
Concentration (pg/mL)
Fmr1 HET Female
Fmr1 KO Female
WT Male
Fmr1 KO Male
5000
4000
3000
2000
1000
0
300
200
100
0
100
80
60
40
20
0
600
400
200
0
40
30
20
10
0
50
40
30
20
10
0
1500
1000
500
0
150
100
50
0

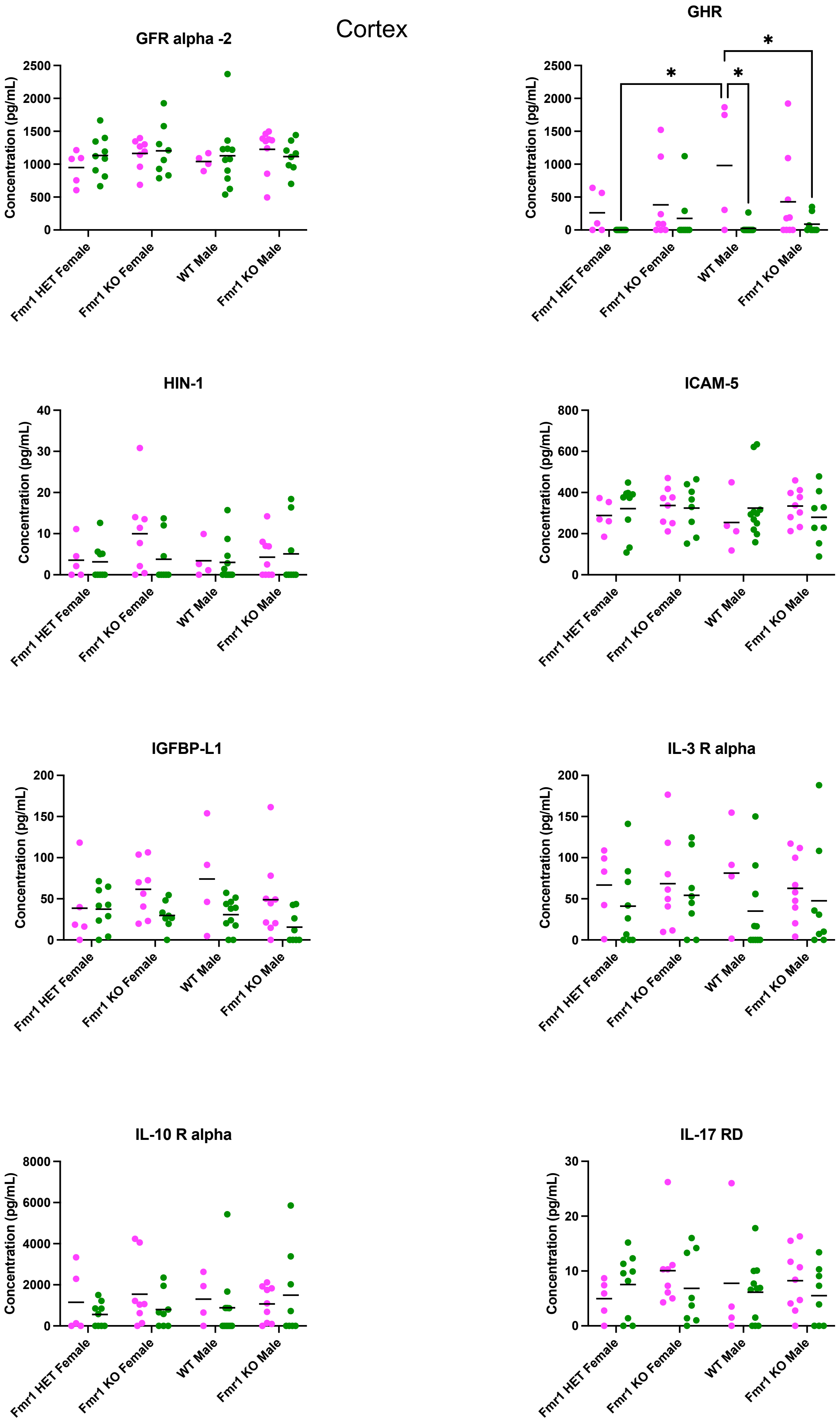

Cortex
GFR alpha -2
GHR
HIN-1
ICAM-5
IGFBP-L1
IL-3 R alpha
IL-10 R alpha
IL-17 RD
Concentration (pg/mL)
Fmr1 HET Female
Fmr1 KO Female
WT Male
Fmr1 KO Male
*

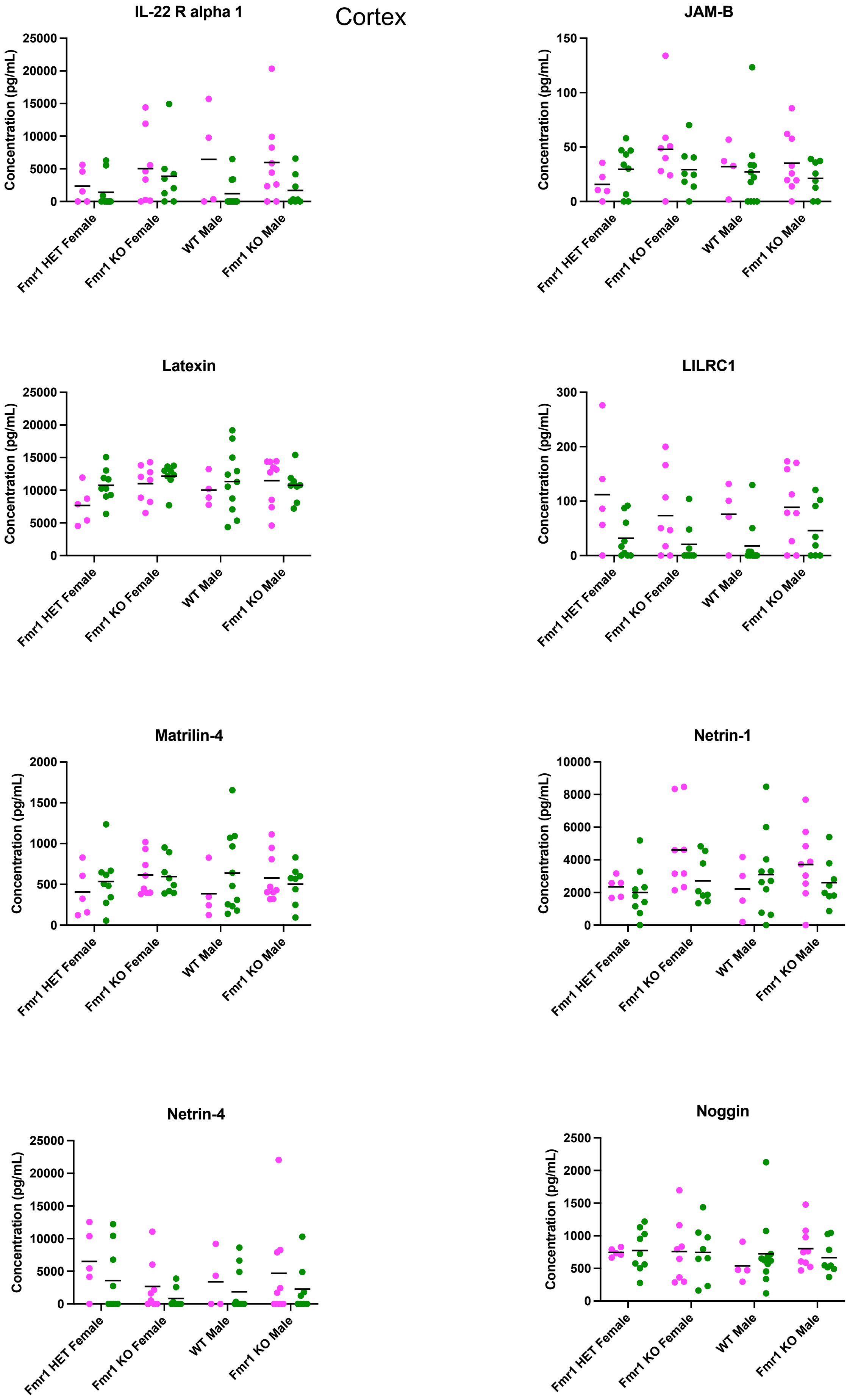
Cortex
IL-22 R alpha 1
JAM-B
Latexin
LILRC1
Matrilin-4
Netrin-1
Netrin-4
Noggin
Concentration (pg/mL)
Fmr1 HET Female
Fmr1 KO Female
WT Male
Fmr1 KO Male

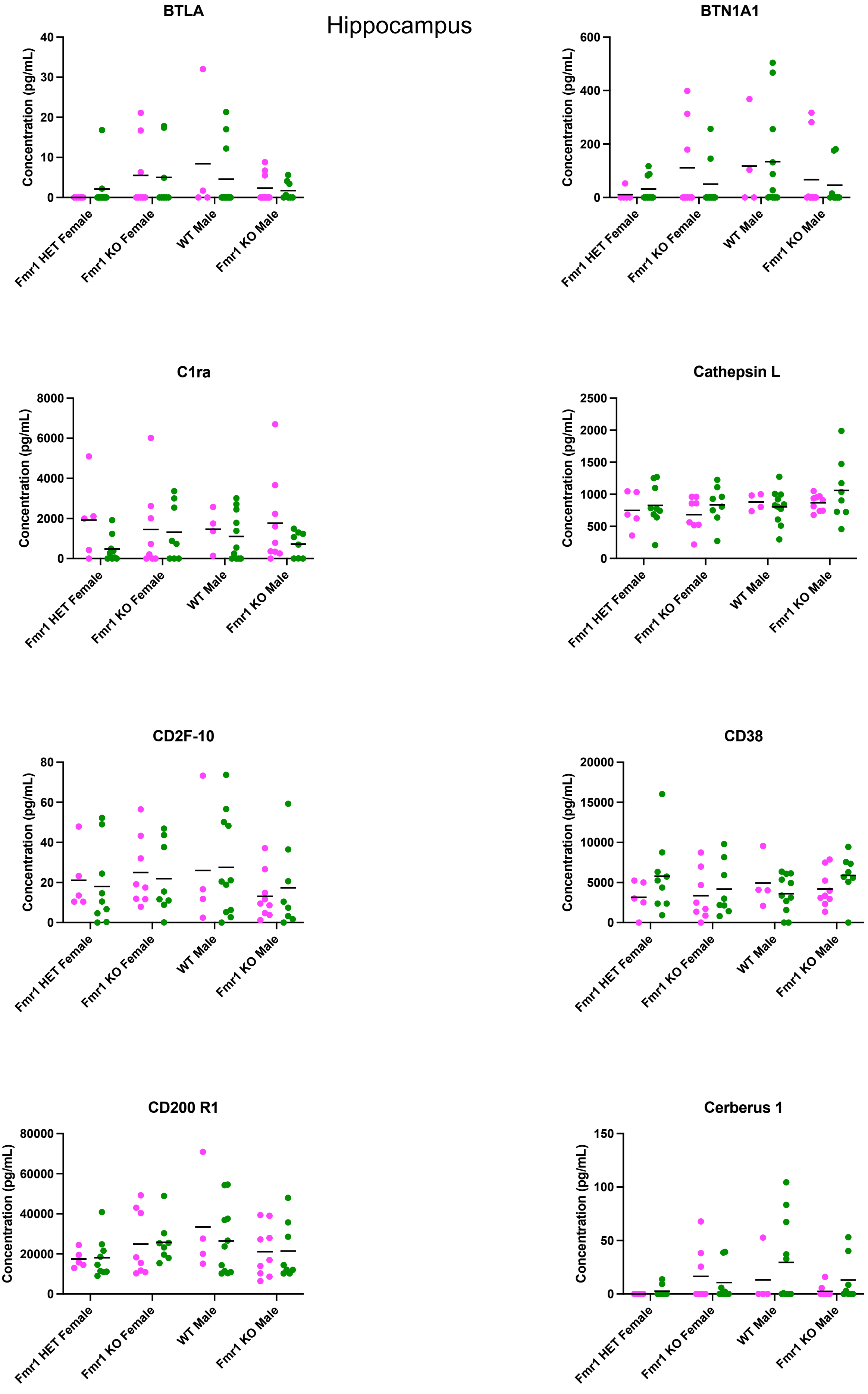

Hippocampus
BTLA
Concentration (pg/mL)
0
10
20
30
40
BTN1A1
Concentration (pg/mL)
0
200
400
600
C1ra
Concentration (pg/mL)
0
2000
4000
6000
8000
Cathepsin L
Concentration (pg/mL)
0
500
1000
1500
2000
2500
CD2F-10
Concentration (pg/mL)
0
20
40
60
80
CD38
Concentration (pg/mL)
0
5000
10000
15000
20000
CD200 R1
Concentration (pg/mL)
0
20000
40000
60000
80000
Cerberus 1
Concentration (pg/mL)
0
50
100
150
Fmr1 HET Female
Fmr1 KO Female
WT Male
Fmr1 KO Male

Hippocampus

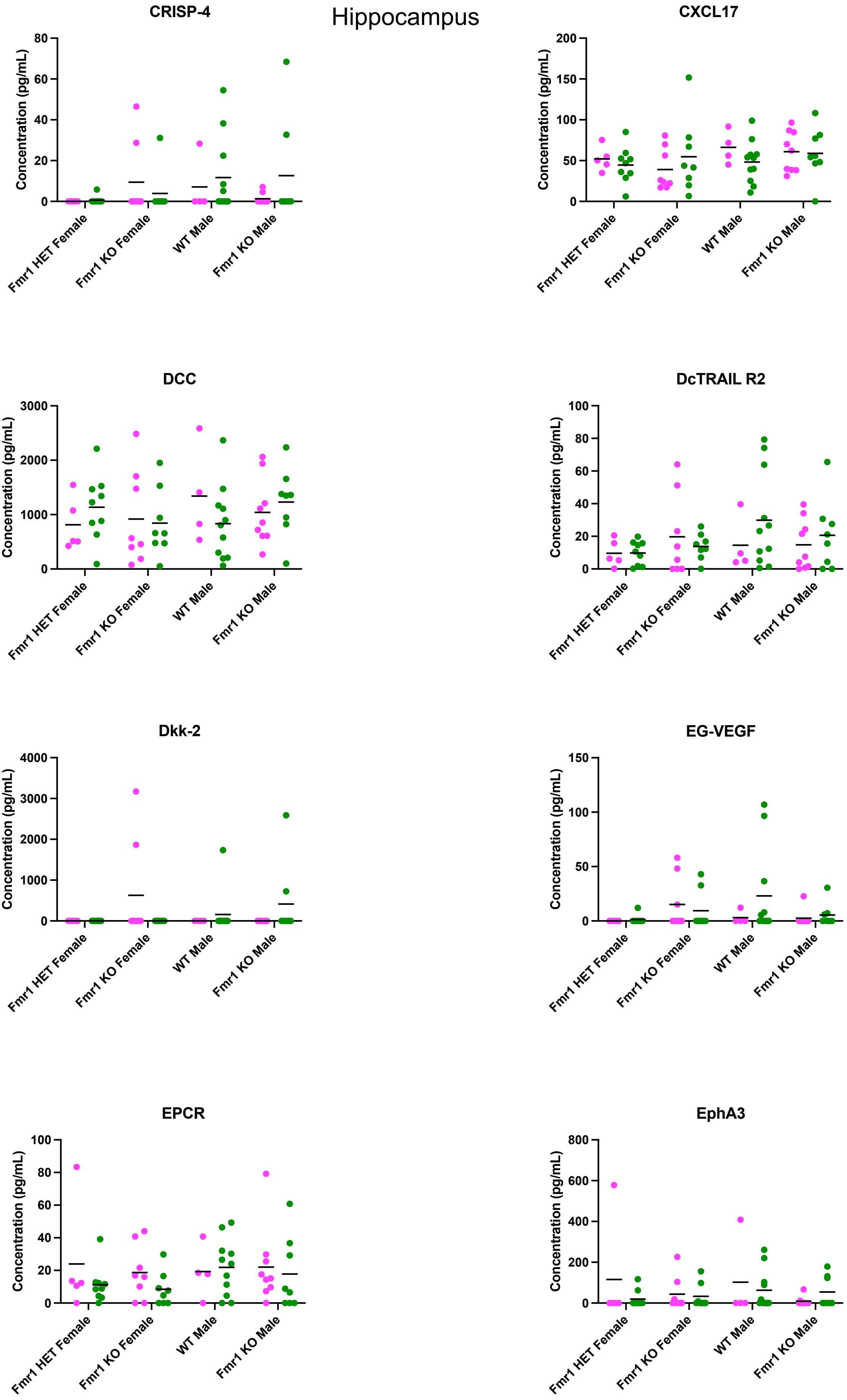

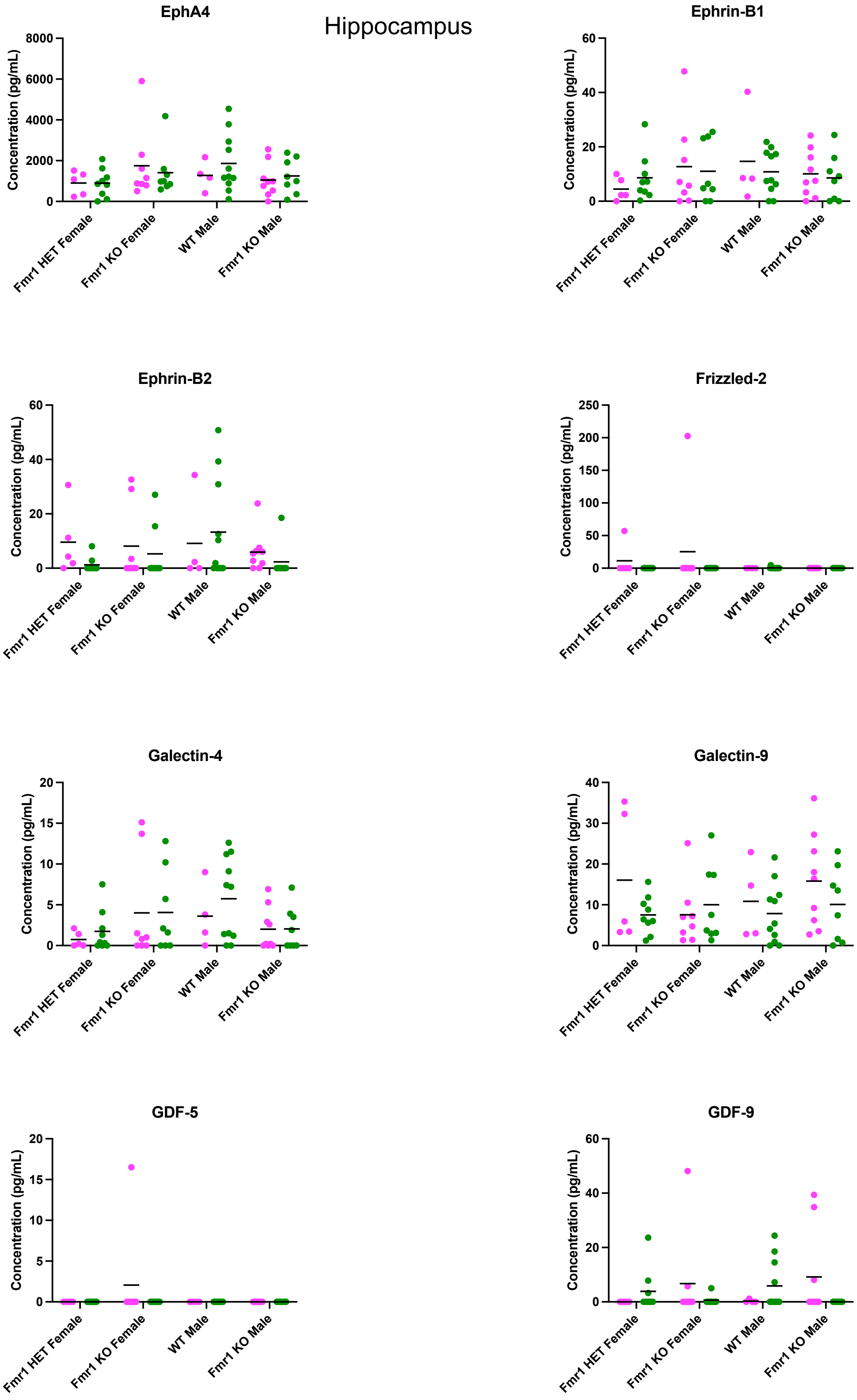

Hippocampus
EphA4
Ephrin-B1
Ephrin-B2
Frizzled-2
Galectin-4
Galectin-9
GDF-5
GDF-9
Concentration (pg/mL)
Fmr1 HET Female
Fmr1 KO Female
WT Male
Fmr1 KO Male

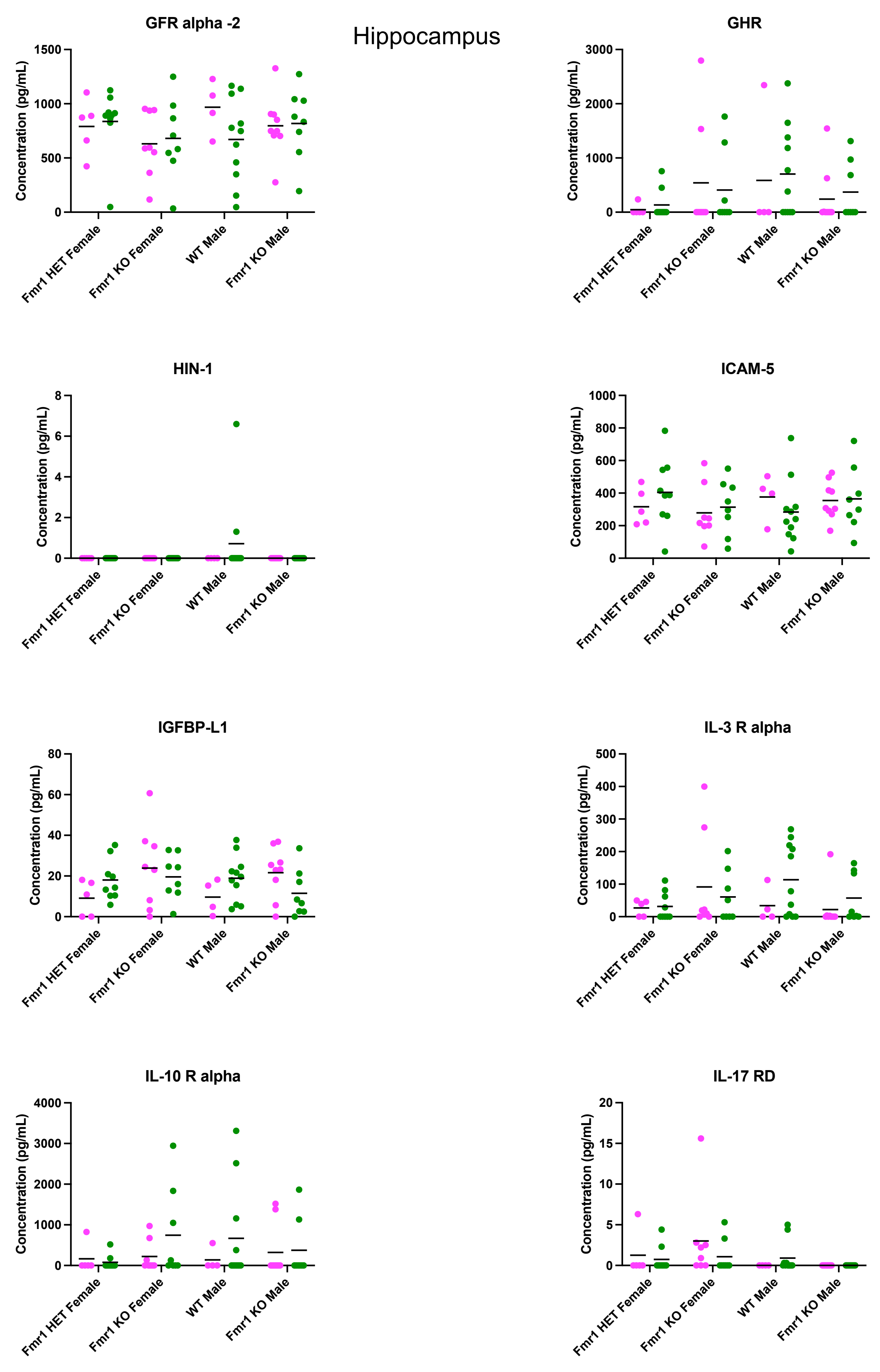

Hippocampus
GFR alpha -2
GHR
HIN-1
ICAM-5
IGFBP-L1
IL-3 R alpha
IL-10 R alpha
IL-17 RD
Concentration (pg/mL)
Fmr1 HET Female
Fmr1 KO Female
WT Male
Fmr1 KO Male
0
500
1000
1500
2000
3000
4000
2
4
6
8
20
40
60
80
100
200
300
400
500
600
800
5
10
15

Hippocampus

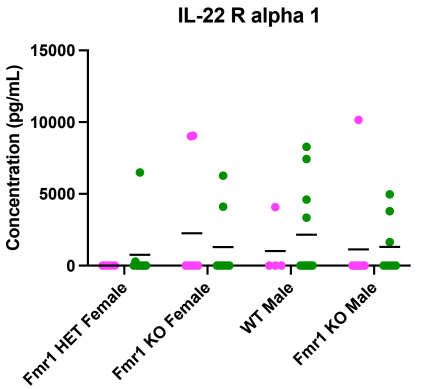

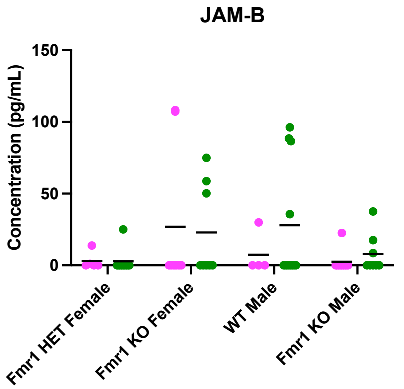

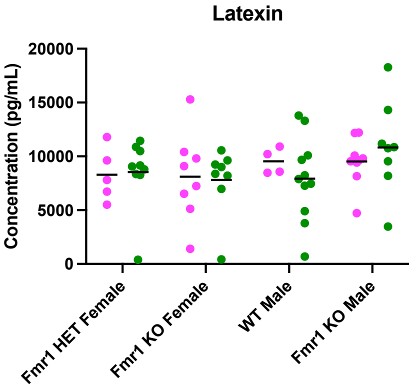

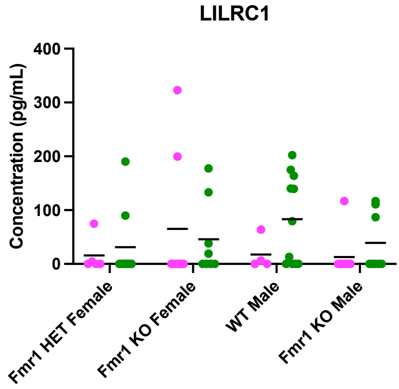

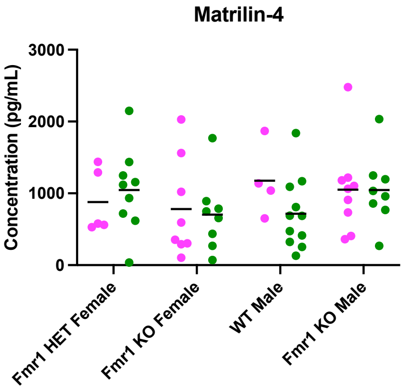

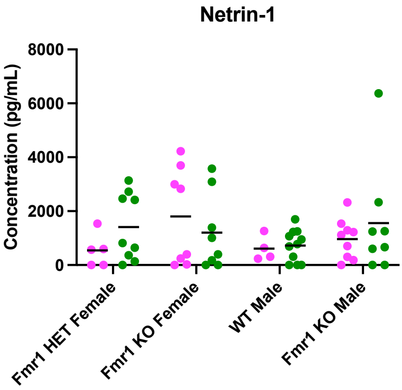

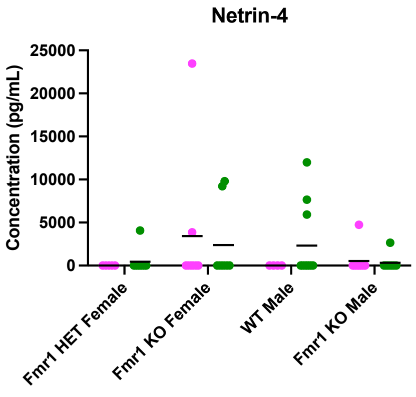

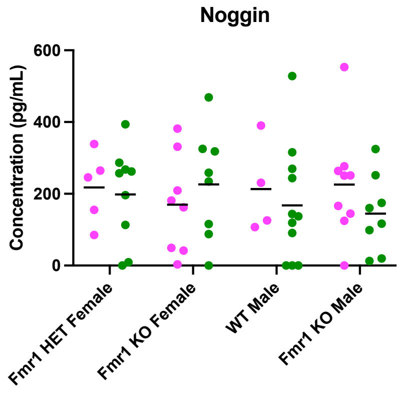

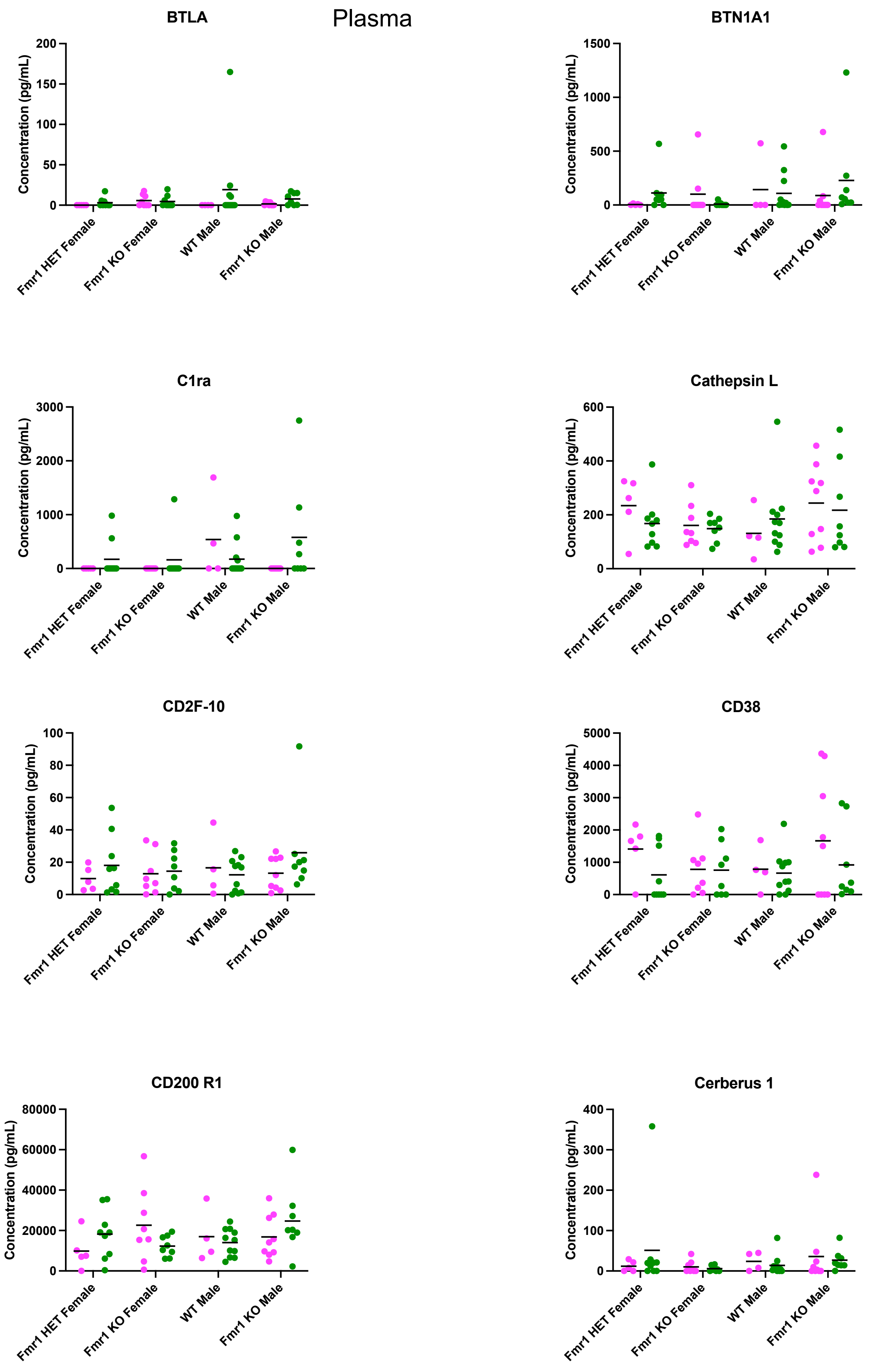
Plasma
BTLA
Concentration (pg/mL)
0
50
100
150
200
Fmr1 HET Female
Fmr1 KO Female
WT Male
Fmr1 KO Male
BTN1A1
Concentration (pg/mL)
0
500
1000
1500
Fmr1 HET Female
Fmr1 KO Female
WT Male
Fmr1 KO Male
C1ra
Concentration (pg/mL)
0
1000
2000
3000
Fmr1 HET Female
Fmr1 KO Female
WT Male
Fmr1 KO Male
Cathepsin L
Concentration (pg/mL)
0
200
400
600
Fmr1 HET Female
Fmr1 KO Female
WT Male
Fmr1 KO Male
CD2F-10
Concentration (pg/mL)
0
20
40
60
80
100
Fmr1 HET Female
Fmr1 KO Female
WT Male
Fmr1 KO Male
CD38
Concentration (pg/mL)
0
1000
2000
3000
4000
5000
Fmr1 HET Female
Fmr1 KO Female
WT Male
Fmr1 KO Male
CD200 R1
Concentration (pg/mL)
0
20000
40000
60000
80000
Fmr1 HET Female
Fmr1 KO Female
WT Male
Fmr1 KO Male
Cerberus 1
Concentration (pg/mL)
0
100
200
300
400
Fmr1 HET Female
Fmr1 KO Female
WT Male
Fmr1 KO Male

Plasma

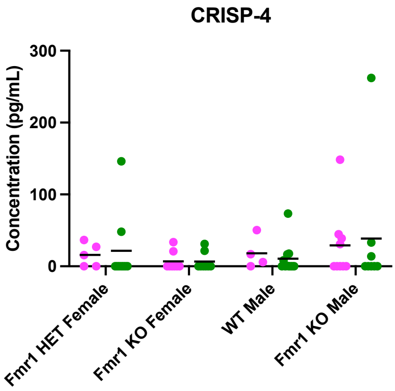
CRISP-4
Concentration (pg/mL)
300
200
100
0
Fmr1 HET Female
Fmr1 KO Female
WT Male
Fmr1 KO Male

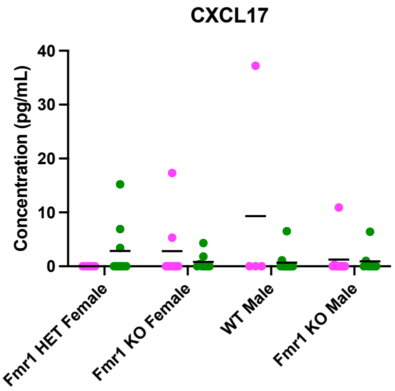
CXCL17
Concentration (pg/mL)
40
30
20
10
0
Fmr1 HET Female
Fmr1 KO Female
WT Male
Fmr1 KO Male

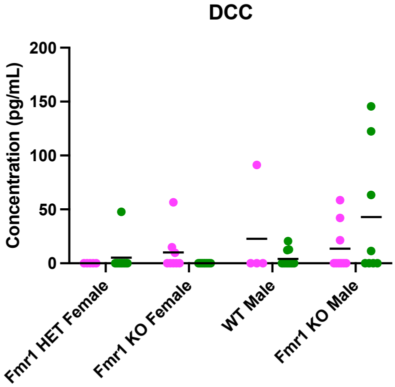
DCC
Concentration (pg/mL)
200
150
100
50
0
Fmr1 HET Female
Fmr1 KO Female
WT Male
Fmr1 KO Male

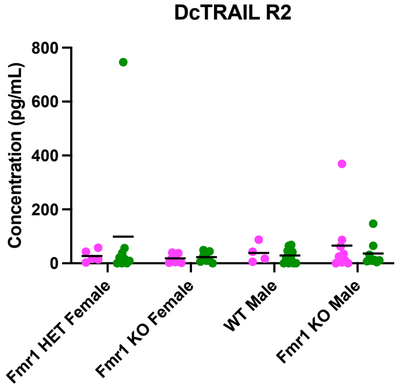
DcTRAIL R2
Concentration (pg/mL)
800
600
400
200
0
Fmr1 HET Female
Fmr1 KO Female
WT Male
Fmr1 KO Male

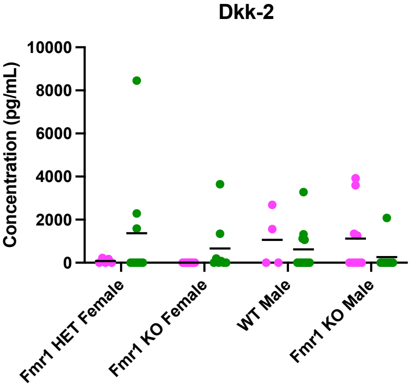
Dkk-2
Concentration (pg/mL)
10000
8000
6000
4000
2000
0
Fmr1 HET Female
Fmr1 KO Female
WT Male
Fmr1 KO Male

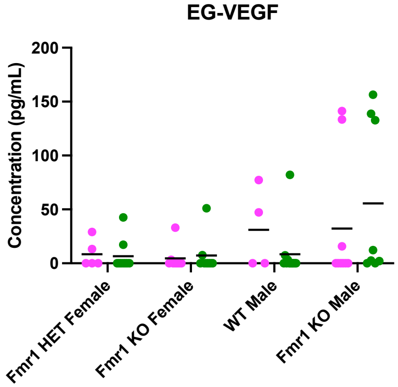
EG-VEGF
Concentration (pg/mL)
200
150
100
50
0
Fmr1 HET Female
Fmr1 KO Female
WT Male
Fmr1 KO Male

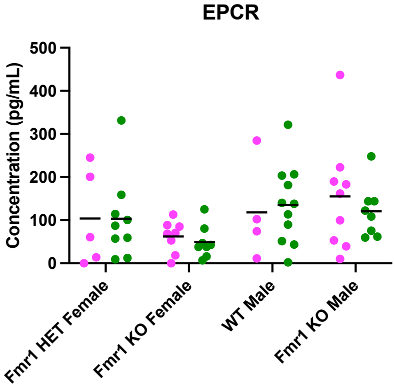
EPCR
Concentration (pg/mL)
500
400
300
200
100
0
Fmr1 HET Female
Fmr1 KO Female
WT Male
Fmr1 KO Male

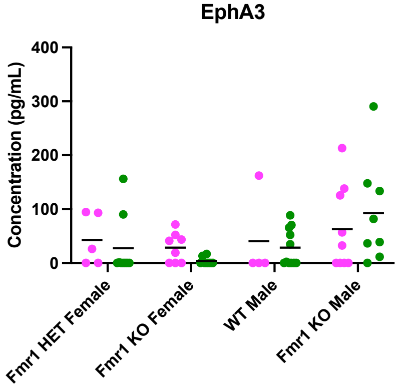
EphA3
Concentration (pg/mL)
400
300
200
100
0
Fmr1 HET Female
Fmr1 KO Female
WT Male
Fmr1 KO Male

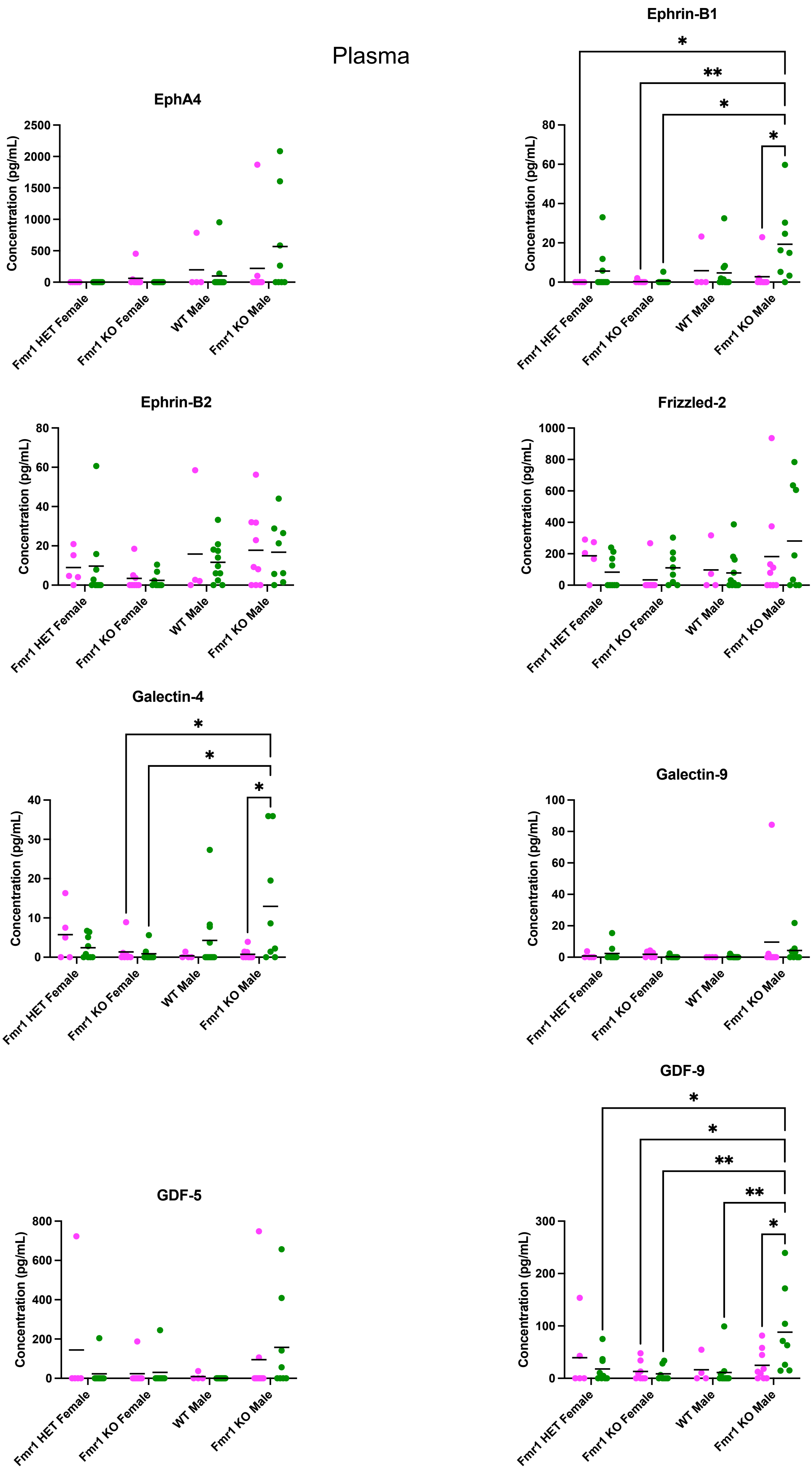

Plasma
EphA4
Concentration (pg/mL)
2500
2000
1500
1000
500
0
Fmr1 HET Female
Fmr1 KO Female
WT Male
Fmr1 KO Male
Ephrin-B1
*
**
*
*
Concentration (pg/mL)
80
60
40
20
0
Fmr1 HET Female
Fmr1 KO Female
WT Male
Fmr1 KO Male
Ephrin-B2
Concentration (pg/mL)
80
60
40
20
0
Fmr1 HET Female
Fmr1 KO Female
WT Male
Fmr1 KO Male
Frizzled-2
Concentration (pg/mL)
1000
800
600
400
200
0
Fmr1 HET Female
Fmr1 KO Female
WT Male
Fmr1 KO Male
Galectin-4
*
*
*
Concentration (pg/mL)
40
30
20
10
0
Fmr1 HET Female
Fmr1 KO Female
WT Male
Fmr1 KO Male
Galectin-9
Concentration (pg/mL)
100
80
60
40
20
0
Fmr1 HET Female
Fmr1 KO Female
WT Male
Fmr1 KO Male
GDF-5
Concentration (pg/mL)
800
600
400
200
0
Fmr1 HET Female
Fmr1 KO Female
WT Male
Fmr1 KO Male
GDF-9
*
*
**
**
*
Concentration (pg/mL)
300
200
100
0
Fmr1 HET Female
Fmr1 KO Female
WT Male
Fmr1 KO Male

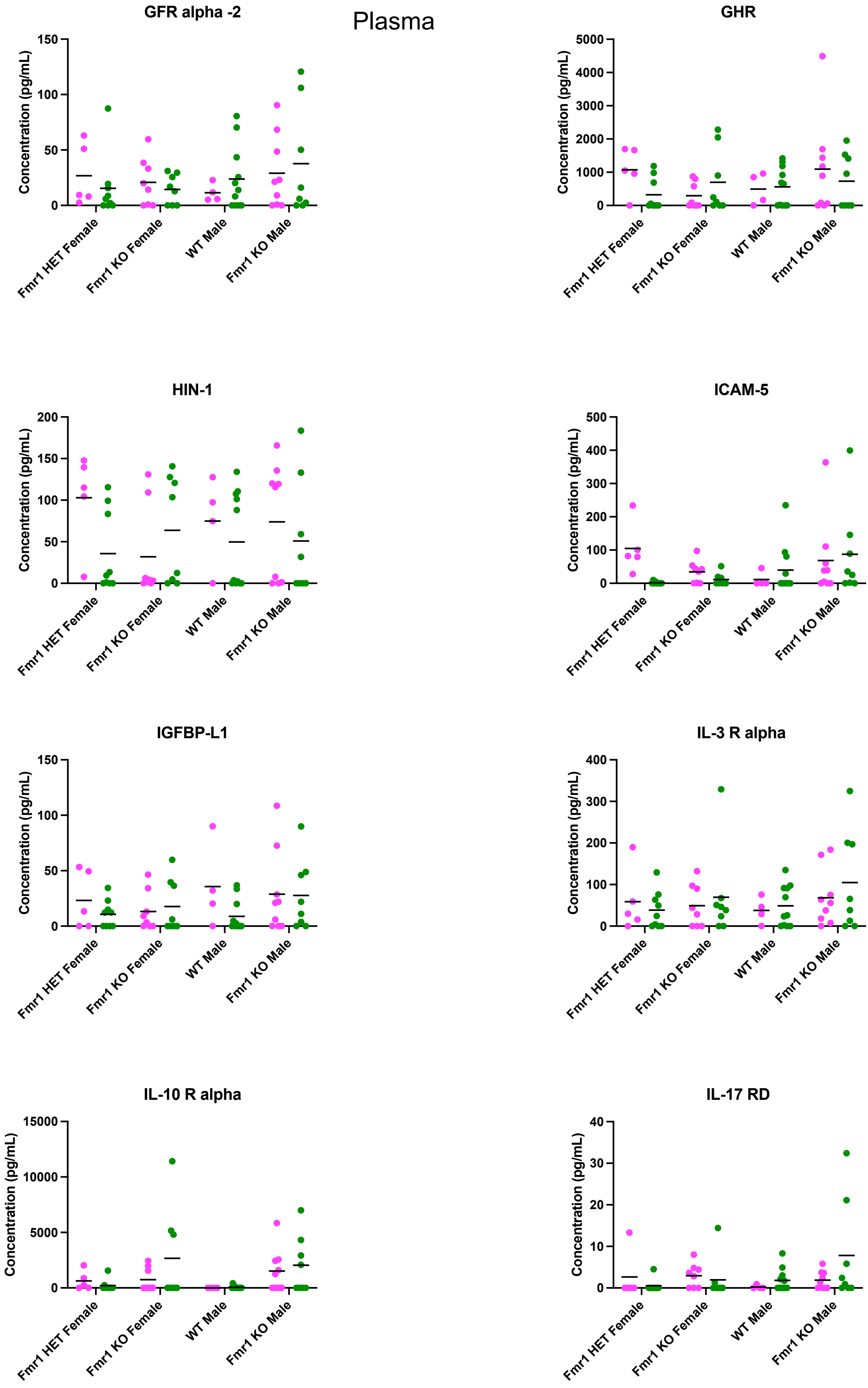
Plasma
GFR alpha -2
GHR
HIN-1
ICAM-5
IGFBP-L1
IL-3 R alpha
IL-10 R alpha
IL-17 RD
Concentration (pg/mL)
Fmr1 HET Female
Fmr1 KO Female
WT Male
Fmr1 KO Male
0
50
100
150
200
300
400
500
1000
2000
3000
4000
5000
10000
15000
10
20
30
40

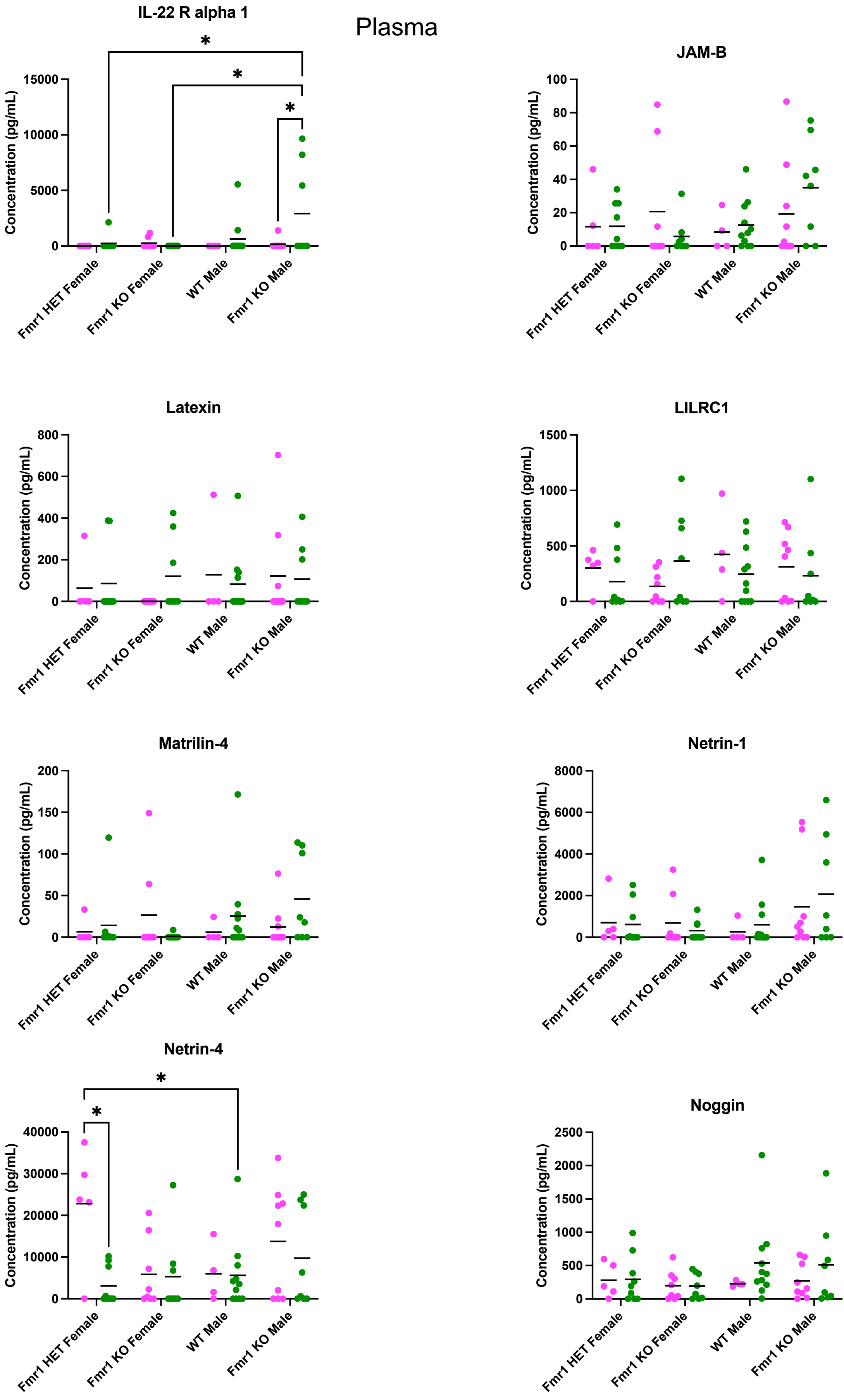
Plasma
IL-22 R alpha 1
*
*
*
15000
10000
5000
0
Concentration (pg/mL)
Fmr1 HET Female
Fmr1 KO Female
WT Male
Fmr1 KO Male
JAM-B
100
80
60
40
20
0
Concentration (pg/mL)
Fmr1 HET Female
Fmr1 KO Female
WT Male
Fmr1 KO Male
Latexin
800
600
400
200
0
Concentration (pg/mL)
Fmr1 HET Female
Fmr1 KO Female
WT Male
Fmr1 KO Male
LILRC1
1500
1000
500
0
Concentration (pg/mL)
Fmr1 HET Female
Fmr1 KO Female
WT Male
Fmr1 KO Male
Matrilin-4
200
150
100
50
0
Concentration (pg/mL)
Fmr1 HET Female
Fmr1 KO Female
WT Male
Fmr1 KO Male
Netrin-1
8000
6000
4000
2000
0
Concentration (pg/mL)
Fmr1 HET Female
Fmr1 KO Female
WT Male
Fmr1 KO Male
Netrin-4
*
*
40000
30000
20000
10000
0
Concentration (pg/mL)
Fmr1 HET Female
Fmr1 KO Female
WT Male
Fmr1 KO Male
Noggin
2500
2000
1500
1000
500
0
Concentration (pg/mL)
Fmr1 HET Female
Fmr1 KO Female
WT Male
Fmr1 KO Male
