# Supplementary material for: Soy Protein Isolate Affects Blood and Brain Biomarker Expression in a Mouse Model of Fragile X
Source: Int J Mol Sci. 2025 Jun 26;26(13):6137. doi: 10.3390/ijms26136137 (PMC12250412; doi:10.3390/ijms26136137)
Supplement: Supplementary file 1 [file ijms-26-06137-s001.zip › Supplementary File S16b Array 18 Graphs.pdf]

Supplementary File S16. Protein expression of Array 18 targets as function of *Fmr1* genotype and AIN-93G diets. Mice on AIN-93G/cas (colored pink) included n=5 $Fmr1^{HET}$ female, n=8 $Fmr1^{KO}$ female, n=4 WT male and n=9 $Fmr1^{KO}$ male. Mice on AIN-93G/soy (colored green) included n=9 $Fmr1^{HET}$ female, n=8 $Fmr1^{KO}$ female, n=11 WT male and n=8 $Fmr1^{KO}$ male. The average concentration in cortex, hippocampus, hypothalamus and plasma in pg/mL was plotted versus genotype. Statistics were determined by 2-way ANOVA and Tukey's multiple comparison tests denoted by $p < 0.05$ (*), $p < 0.01$ (**), $p < 0.001$ (***) and $p < 0.0001$ (****).

Cortex

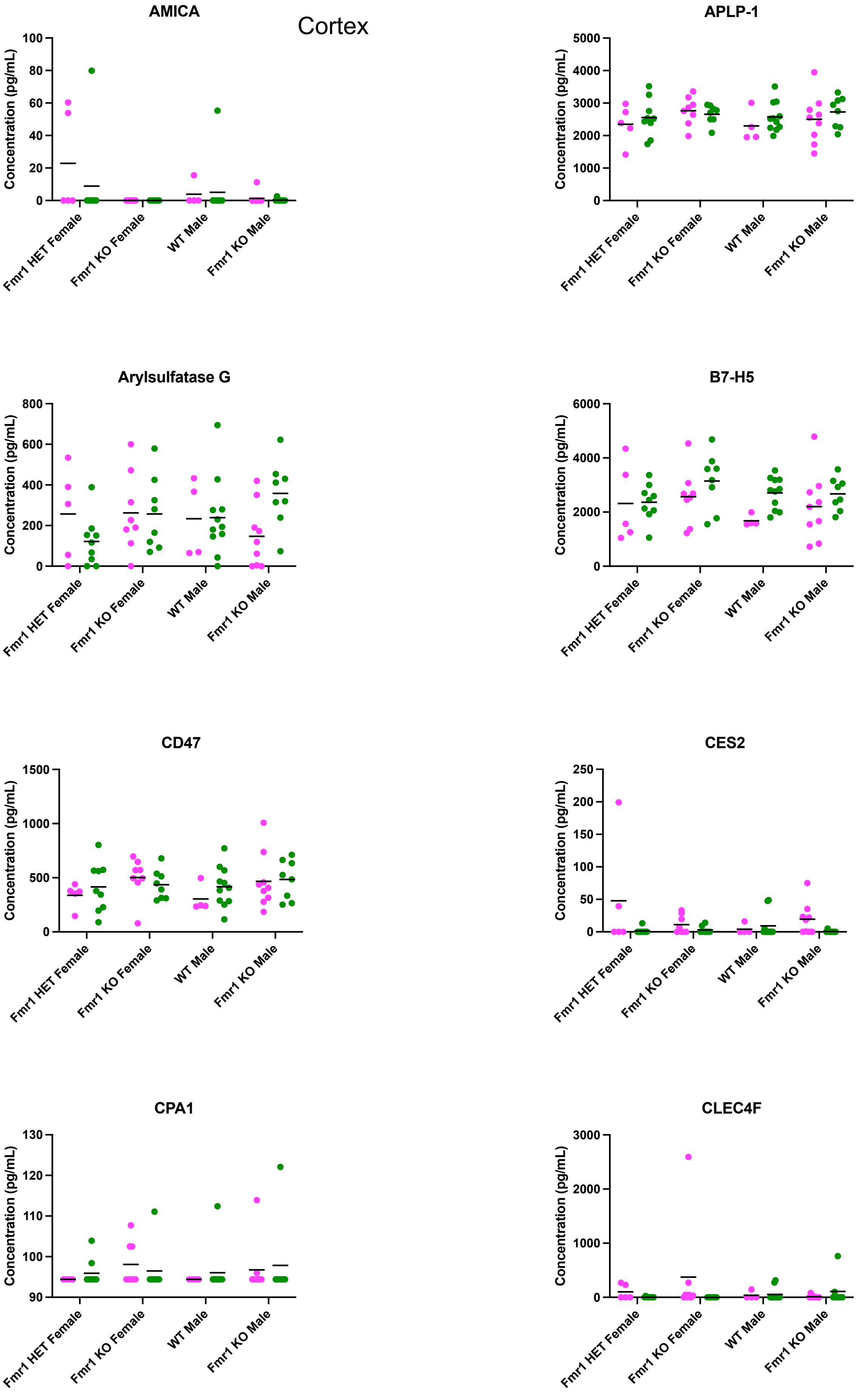
AMICA
APLP-1
Arylsulfatase G
B7-H5
CD47
CES2
CPA1
CLEC4F
Concentration (pg/mL)
Fmr1 HET Female
Fmr1 KO Female
WT Male
Fmr1 KO Male
0
20
40
60
80
100
1000
2000
3000
4000
5000
200
400
600
800
6000
500
1500
50
150
250
90
110
120
130

Cortex

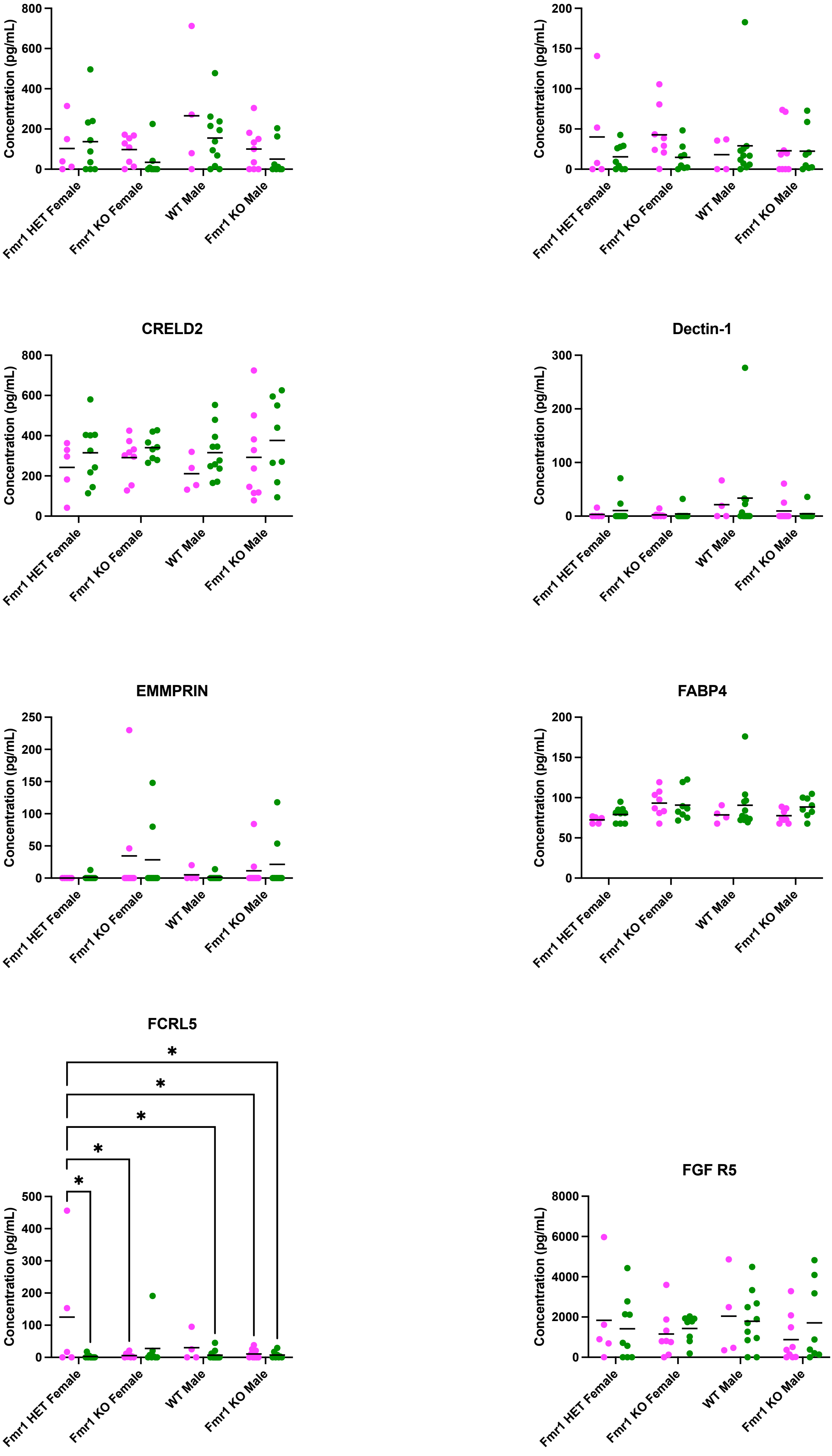
CPB1
CREG
CRELD2
Dectin-1
EMMPRIN
FABP4
FCRL5
FGF R5
Concentration (pg/mL)
Fmr1 HET Female
Fmr1 KO Female
WT Male
Fmr1 KO Male
*
*
*
*
*

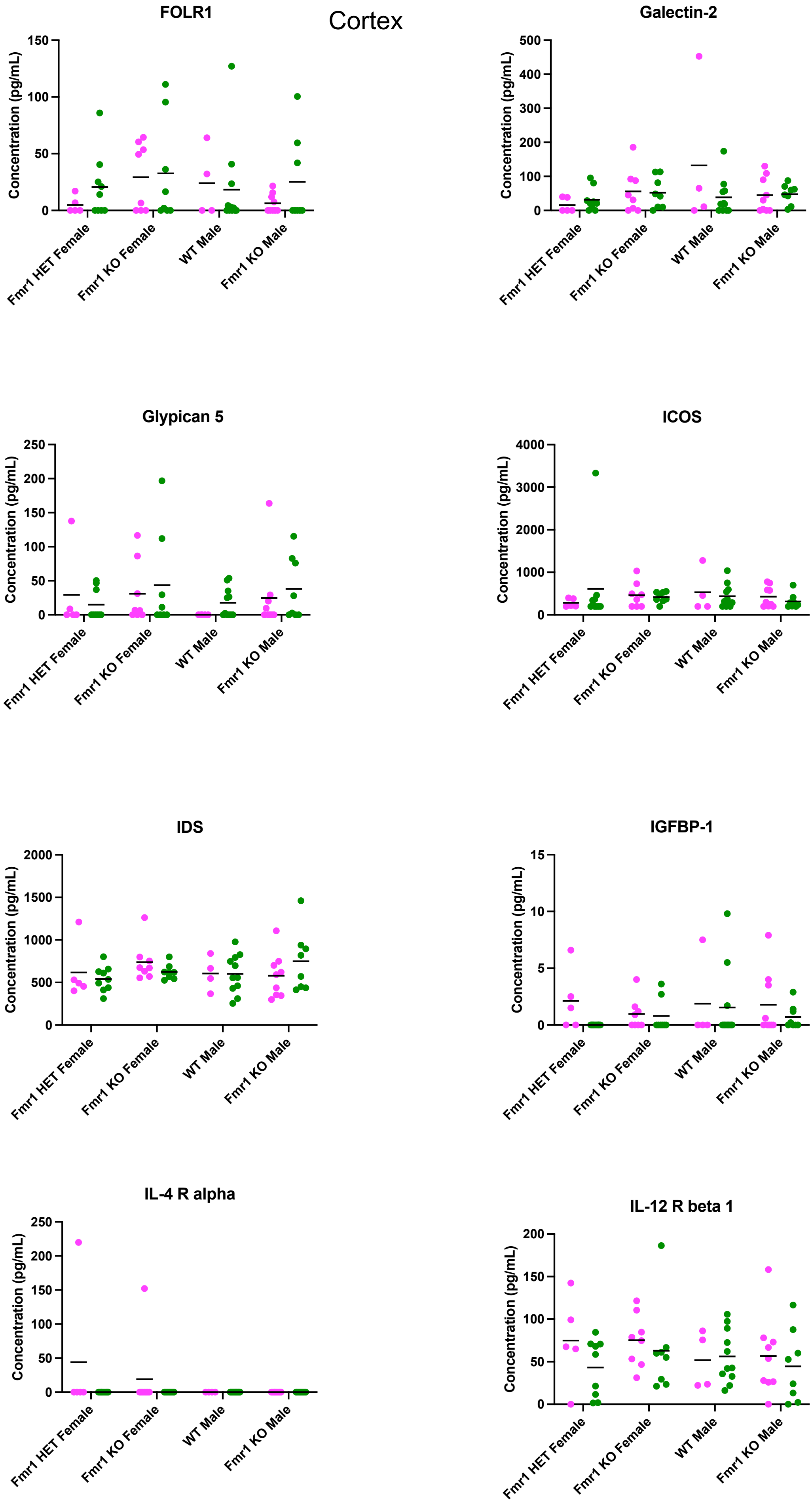
Cortex
FOLR1
Galectin-2
Glypican 5
ICOS
IDS
IGFBP-1
IL-4 R alpha
IL-12 R beta 1
Concentration (pg/mL)
Fmr1 HET Female
Fmr1 KO Female
WT Male
Fmr1 KO Male

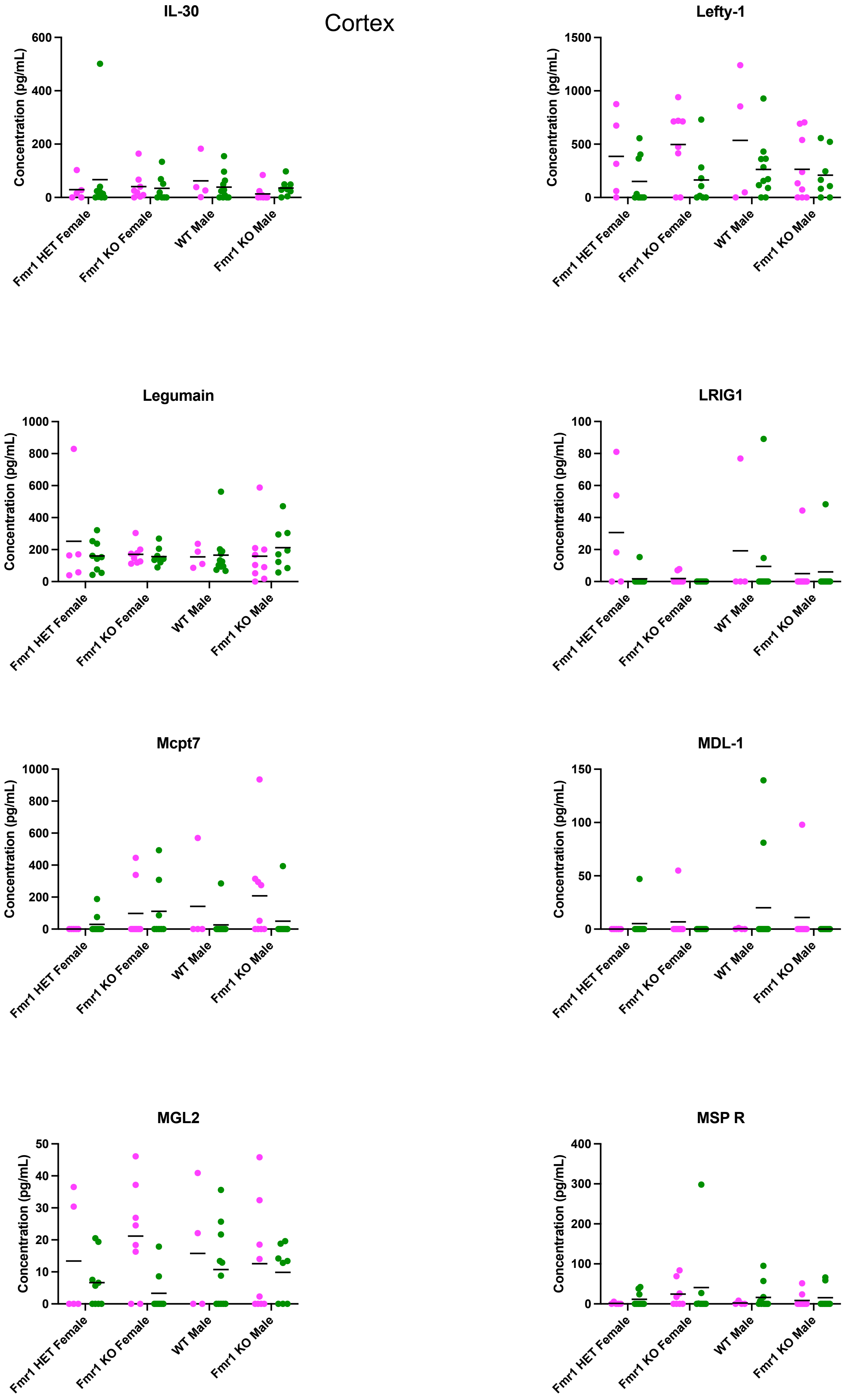
Cortex
IL-30
Lefty-1
Legumain
LRIG1
Mcpt7
MDL-1
MGL2
MSP R
Concentration (pg/mL)
Fmr1 HET Female
Fmr1 KO Female
WT Male
Fmr1 KO Male
0
200
400
600
500
1000
1500
800
20
40
60
80
100
50
150
10
30
300

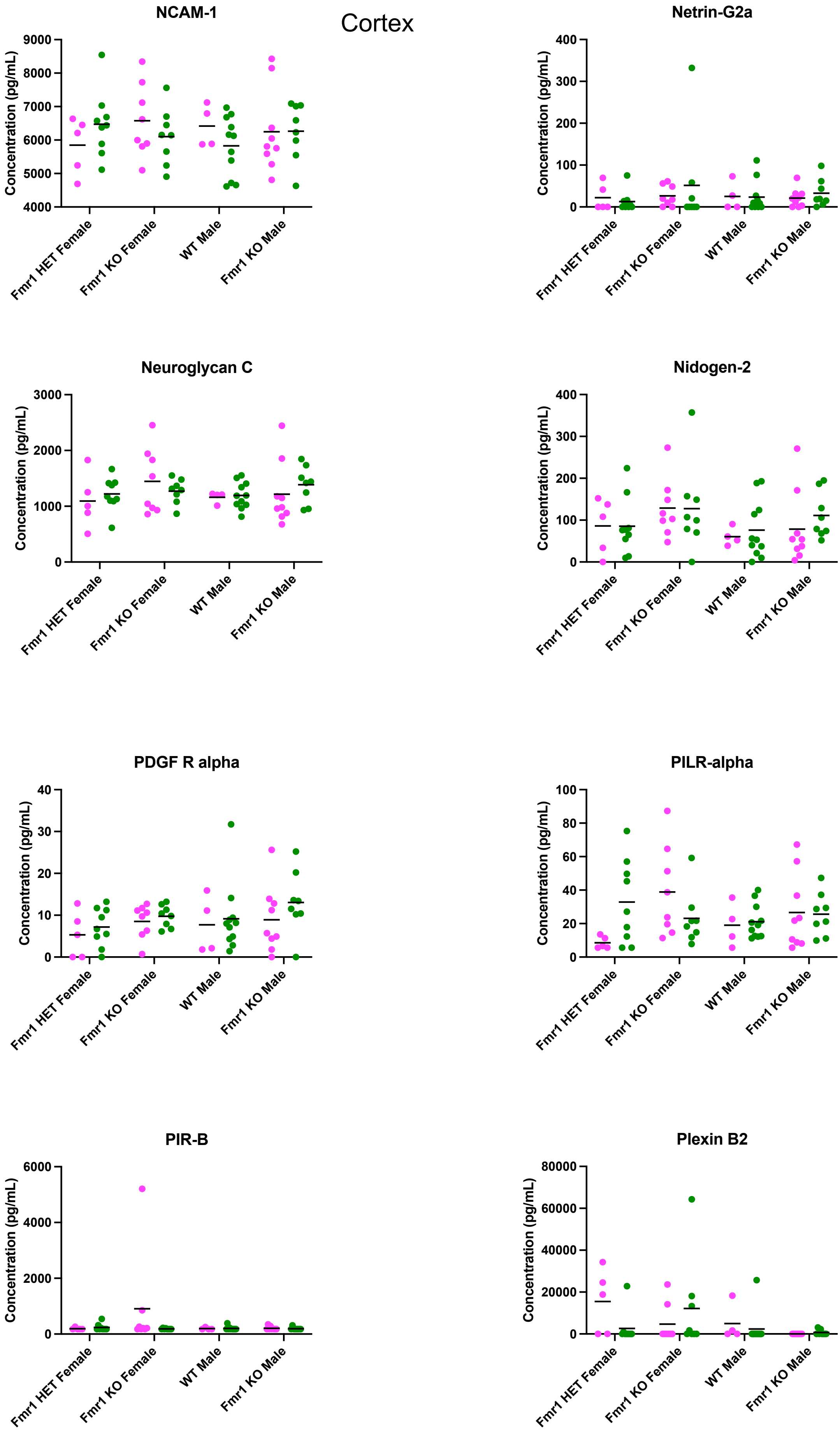
Cortex
NCAM-1
Netrin-G2a
Neuroglycan C
Nidogen-2
PDGF R alpha
PILR-alpha
PIR-B
Plexin B2
Concentration (pg/mL)
9000
8000
7000
6000
5000
4000
400
300
200
100
0
3000
2000
1000
40
30
20
10
80
60
6000
4000
2000
80000
60000
40000
20000
Fmr1 HET Female
Fmr1 KO Female
WT Male
Fmr1 KO Male

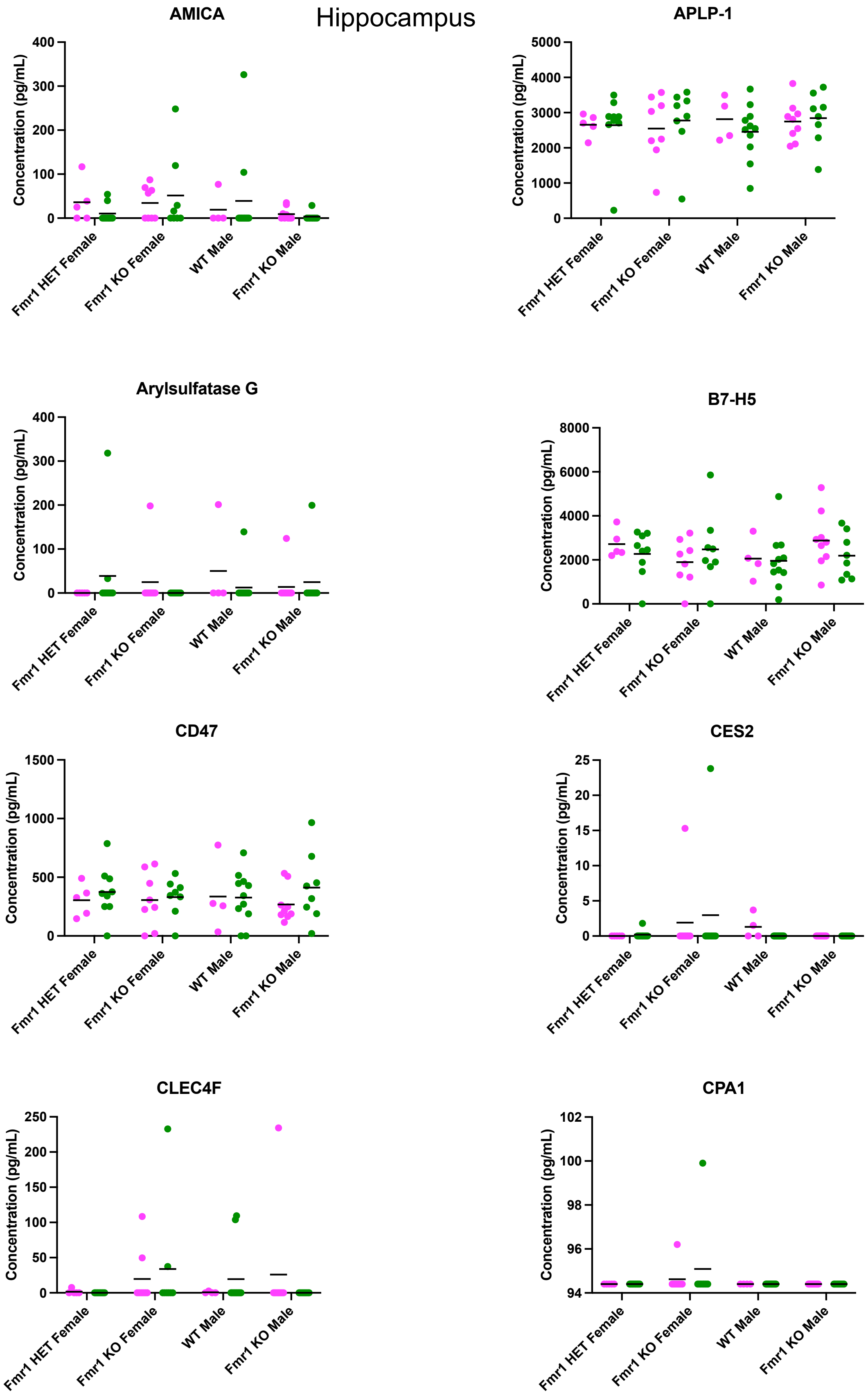
Hippocampus
AMICA
APLP-1
Arylsulfatase G
B7-H5
CD47
CES2
CLEC4F
CPA1
Concentration (pg/mL)
Fmr1 HET Female
Fmr1 KO Female
WT Male
Fmr1 KO Male

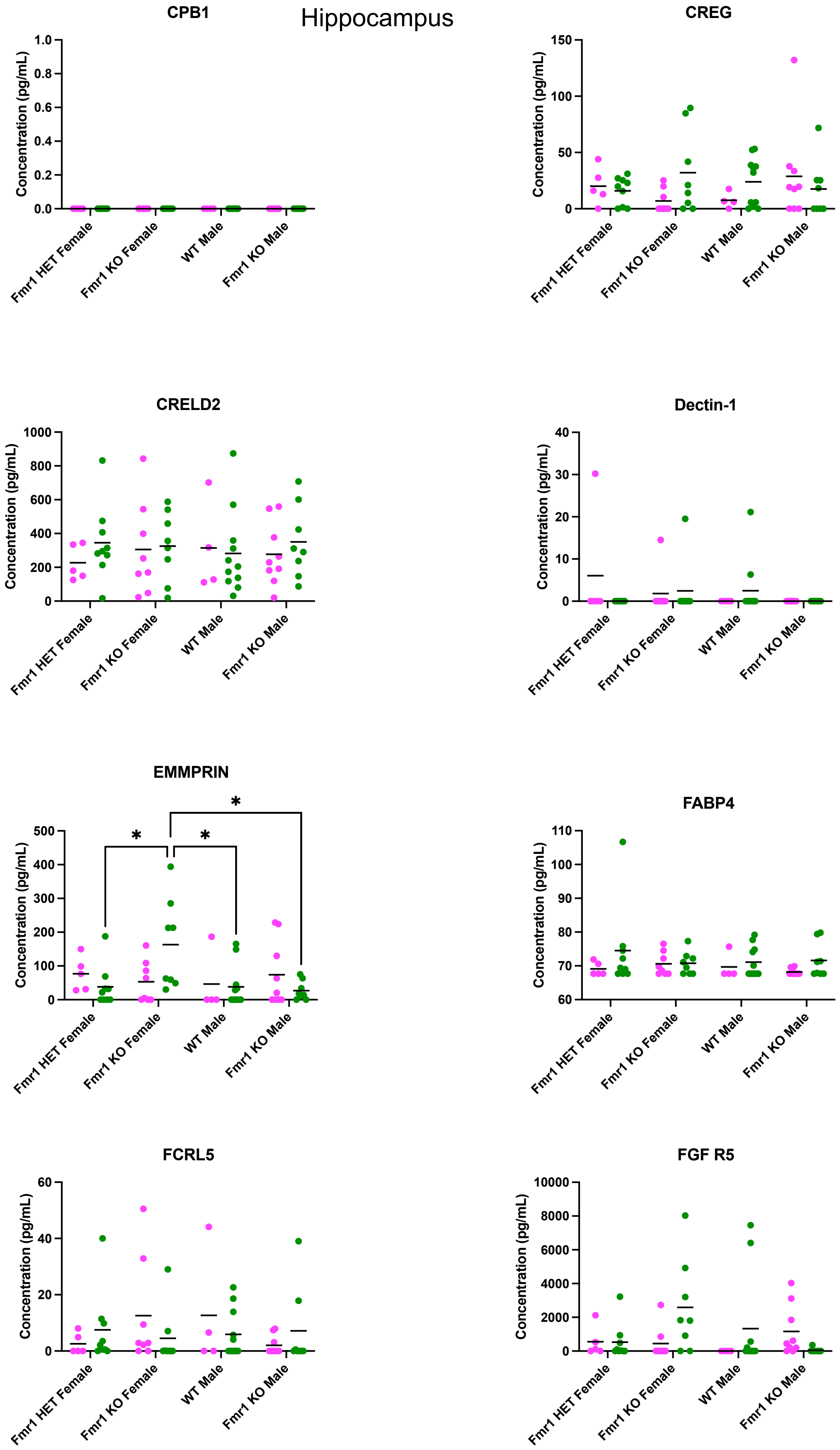
Hippocampus
CPB1
CREG
CRELD2
Dectin-1
EMMPRIN
FABP4
FCRL5
FGF R5
Concentration (pg/mL)
Fmr1 HET Female
Fmr1 KO Female
WT Male
Fmr1 KO Male
*
*
*

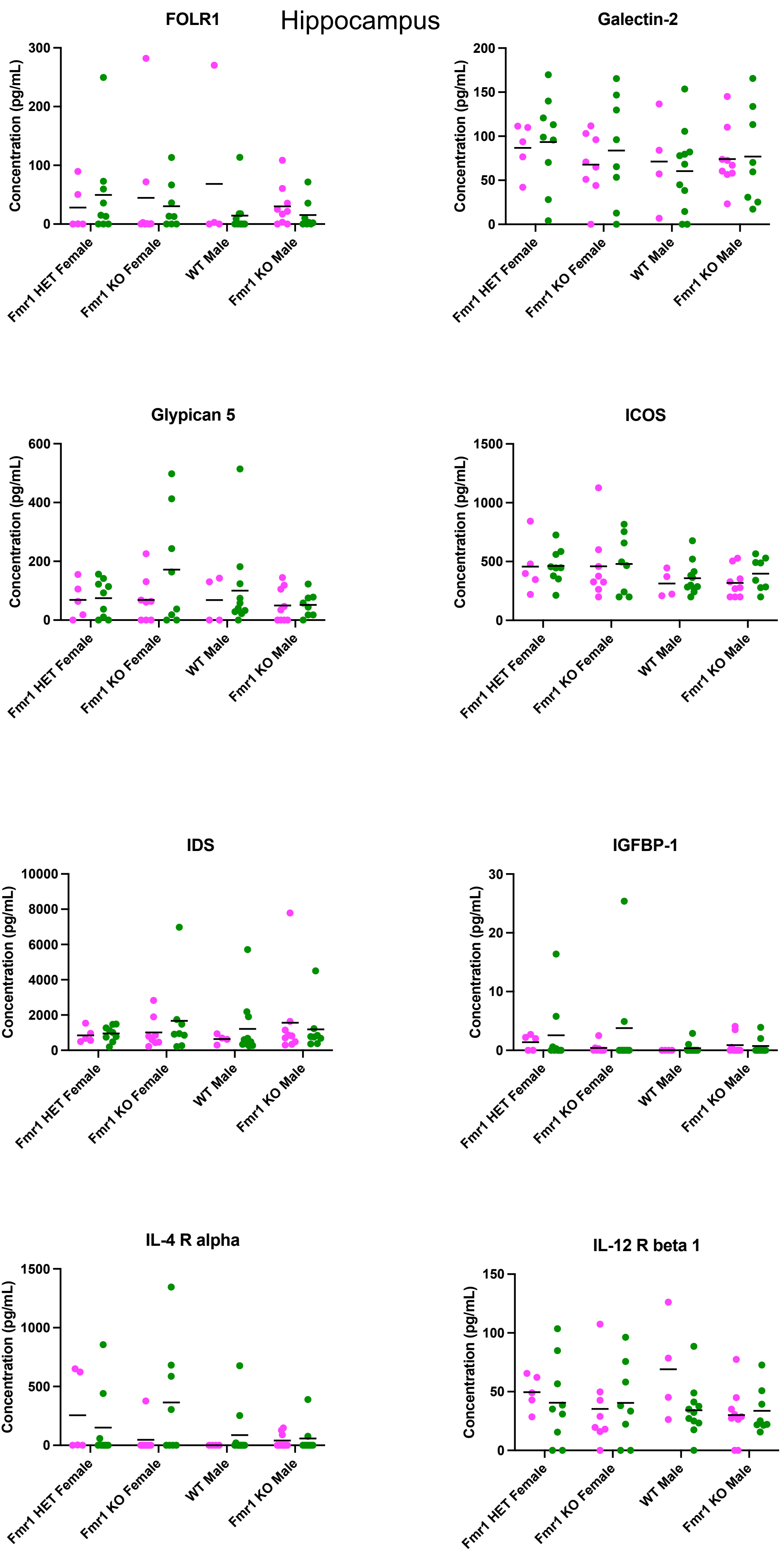

Hippocampus
FOLR1
Galectin-2
Glypican 5
ICOS
IDS
IGFBP-1
IL-4 R alpha
IL-12 R beta 1
Concentration (pg/mL)
Fmr1 HET Female
Fmr1 KO Female
WT Male
Fmr1 KO Male

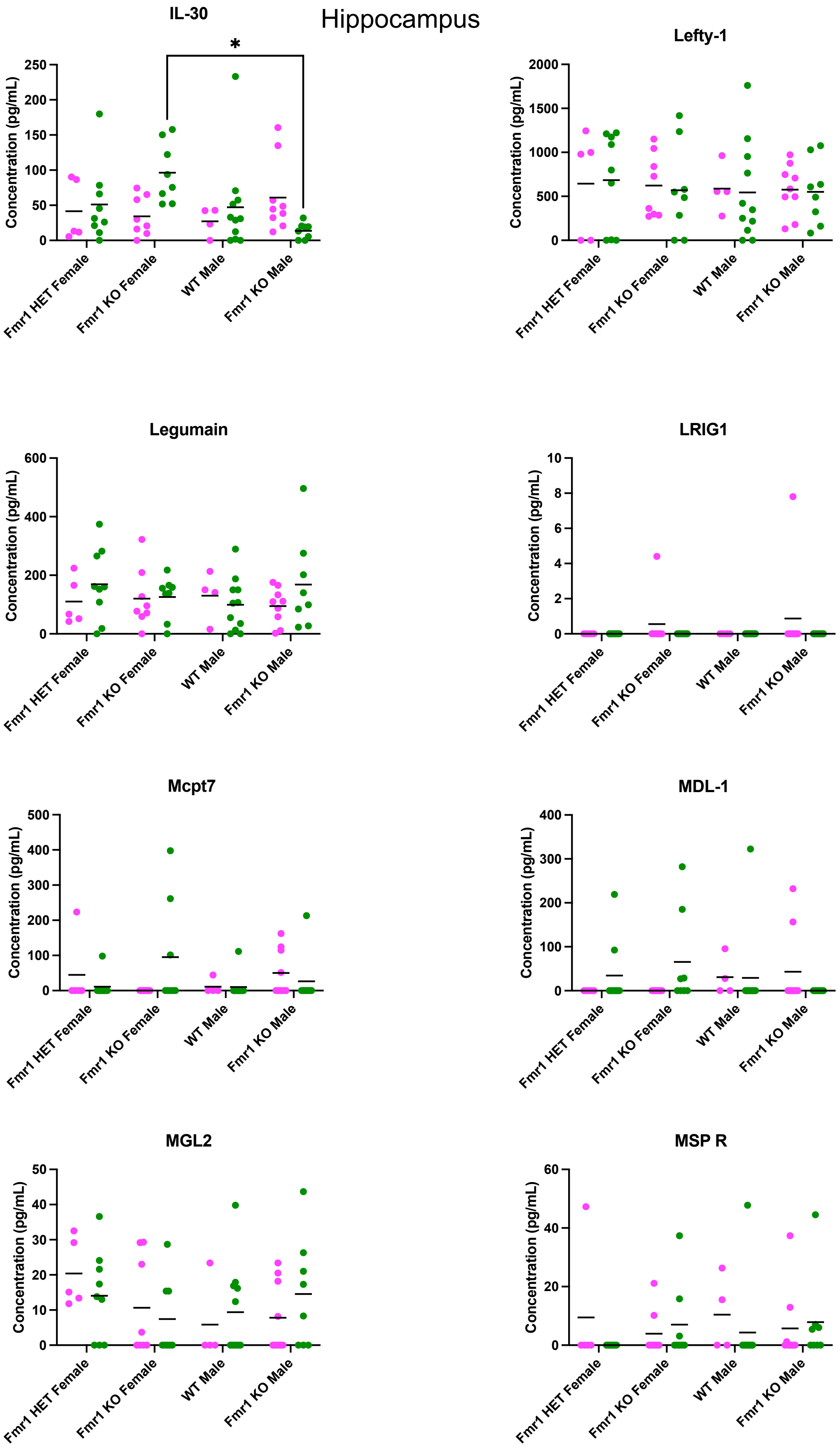
Hippocampus
IL-30
*
Lefty-1
Legumain
LRIG1
Mcpt7
MDL-1
MGL2
MSP R
Concentration (pg/mL)
Fmr1 HET Female
Fmr1 KO Female
WT Male
Fmr1 KO Male

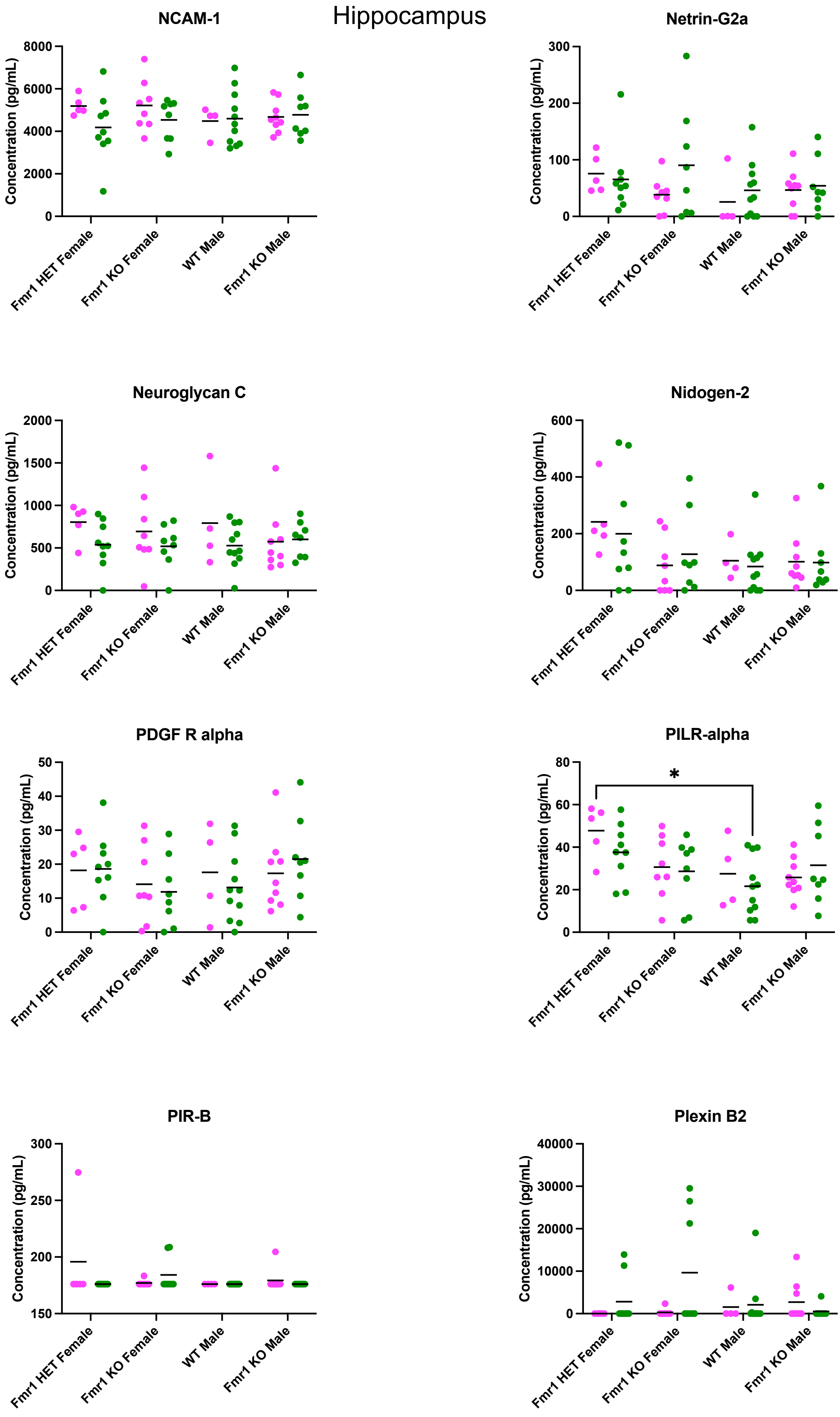
Hippocampus
NCAM-1
Netrin-G2a
Neuroglycan C
Nidogen-2
PDGF R alpha
PILR-alpha
*
PIR-B
Plexin B2
Concentration (pg/mL)
0
2000
4000
6000
8000
100
200
300
500
1000
1500
2000
400
600
10
20
30
40
50
60
80
150
250
10000
20000
30000
40000
Fmr1 HET Female
Fmr1 KO Female
WT Male
Fmr1 KO Male

Plasma

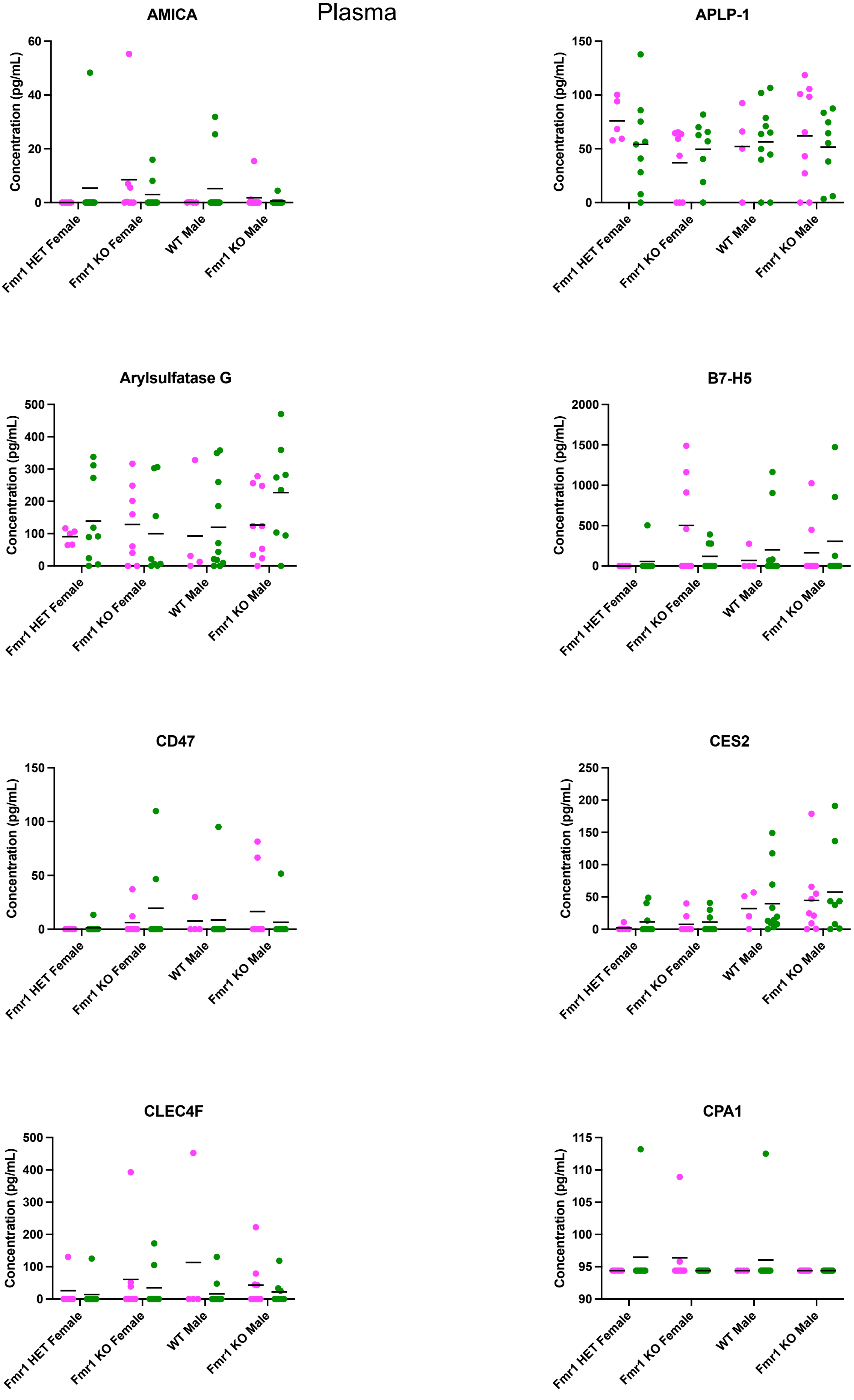
AMICA
APLP-1
Arylsulfatase G
B7-H5
CD47
CES2
CLEC4F
CPA1
Concentration (pg/mL)
Fmr1 HET Female
Fmr1 KO Female
WT Male
Fmr1 KO Male

Plasma

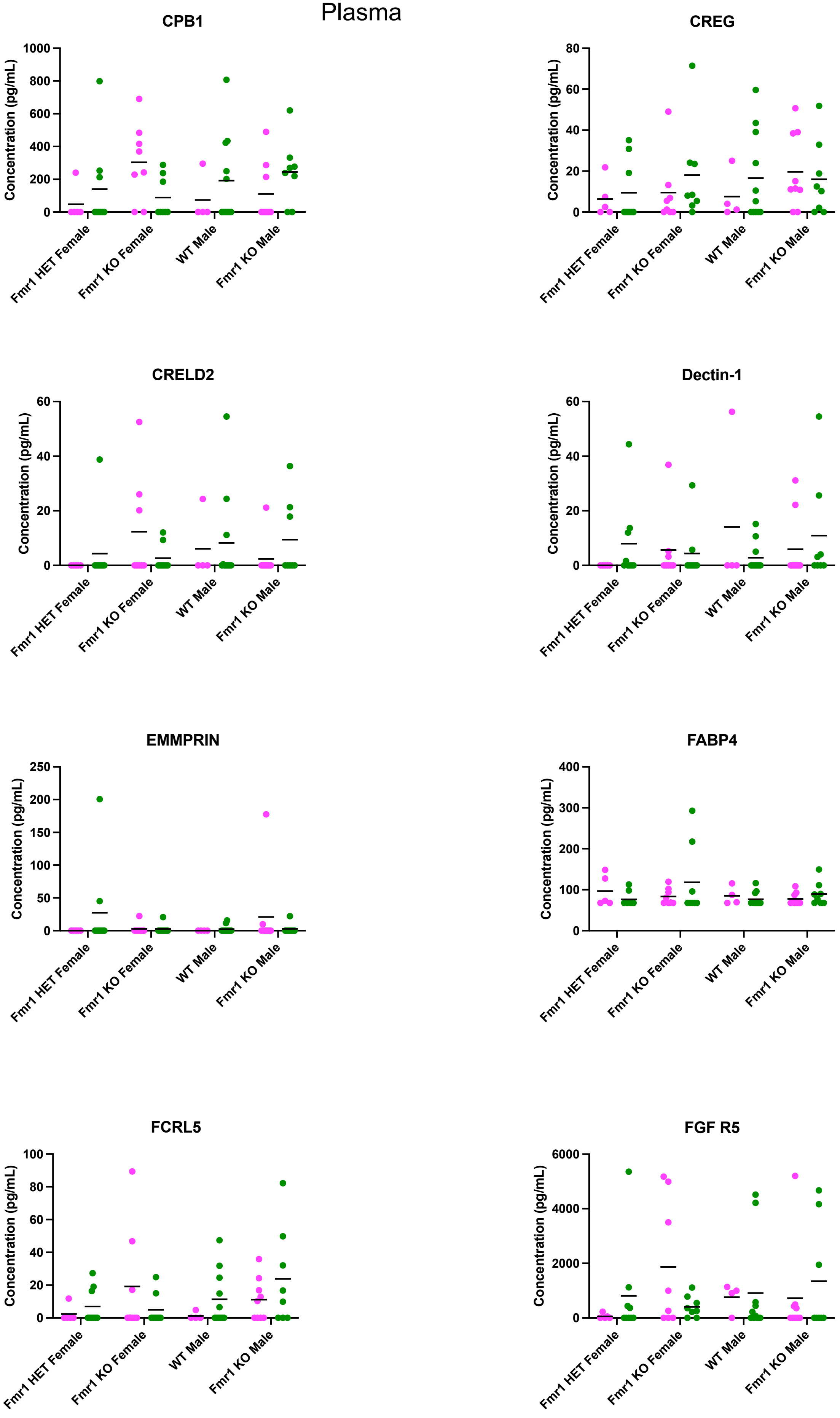
CPB1
CREG
CRELD2
Dectin-1
EMMPRIN
FABP4
FCRL5
FGF R5
Concentration (pg/mL)
0
200
400
600
800
1000
20
40
60
80
50
100
150
250
300
2000
4000
6000
Fmr1 HET Female
Fmr1 KO Female
WT Male
Fmr1 KO Male

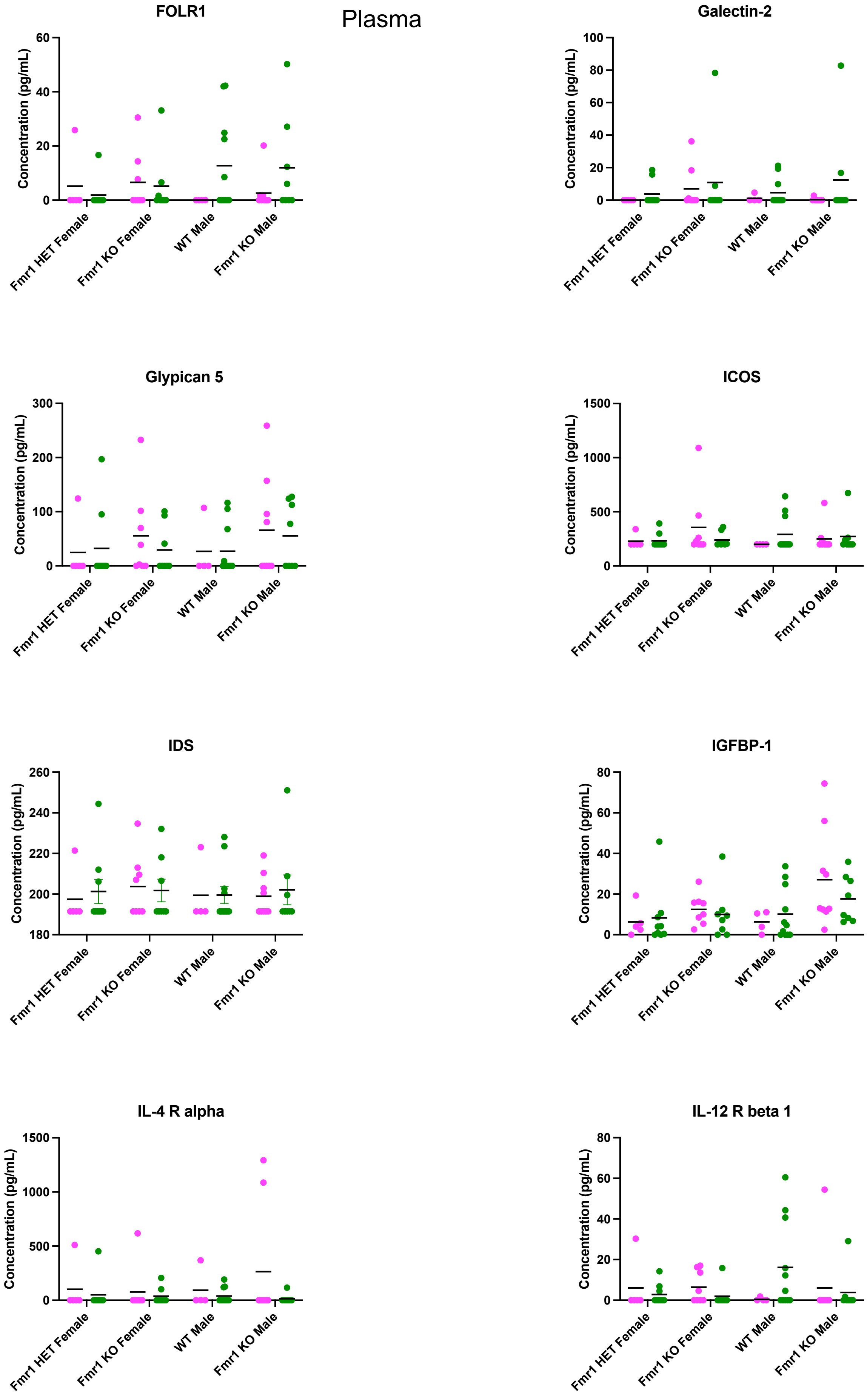
Plasma
FOLR1
Galectin-2
Glypican 5
ICOS
IDS
IGFBP-1
IL-4 R alpha
IL-12 R beta 1
Concentration (pg/mL)
Fmr1 HET Female
Fmr1 KO Female
WT Male
Fmr1 KO Male

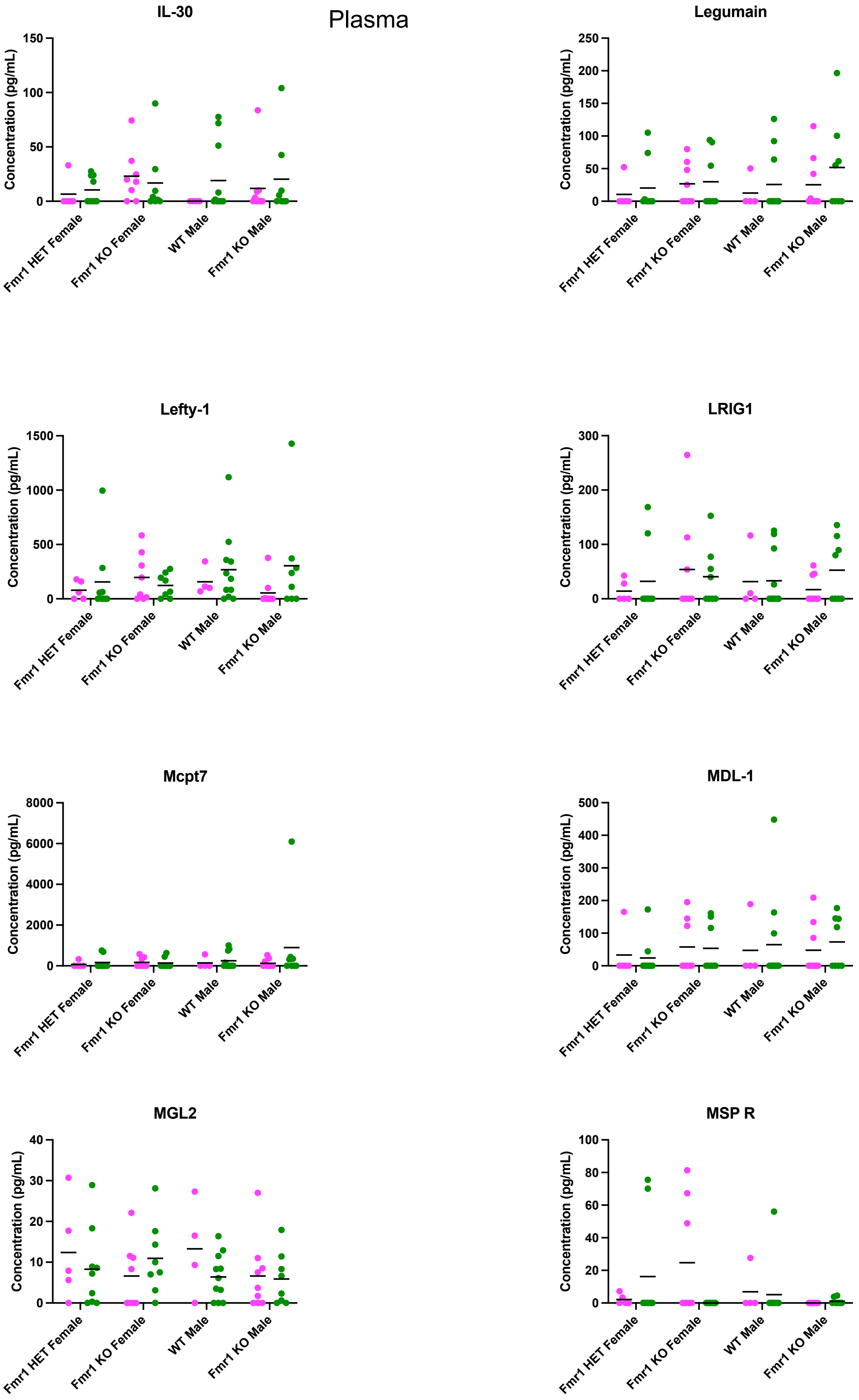
Plasma
IL-30
Legumain
Lefty-1
LRIG1
Mcpt7
MDL-1
MGL2
MSP R
Concentration (pg/mL)
Fmr1 HET Female
Fmr1 KO Female
WT Male
Fmr1 KO Male

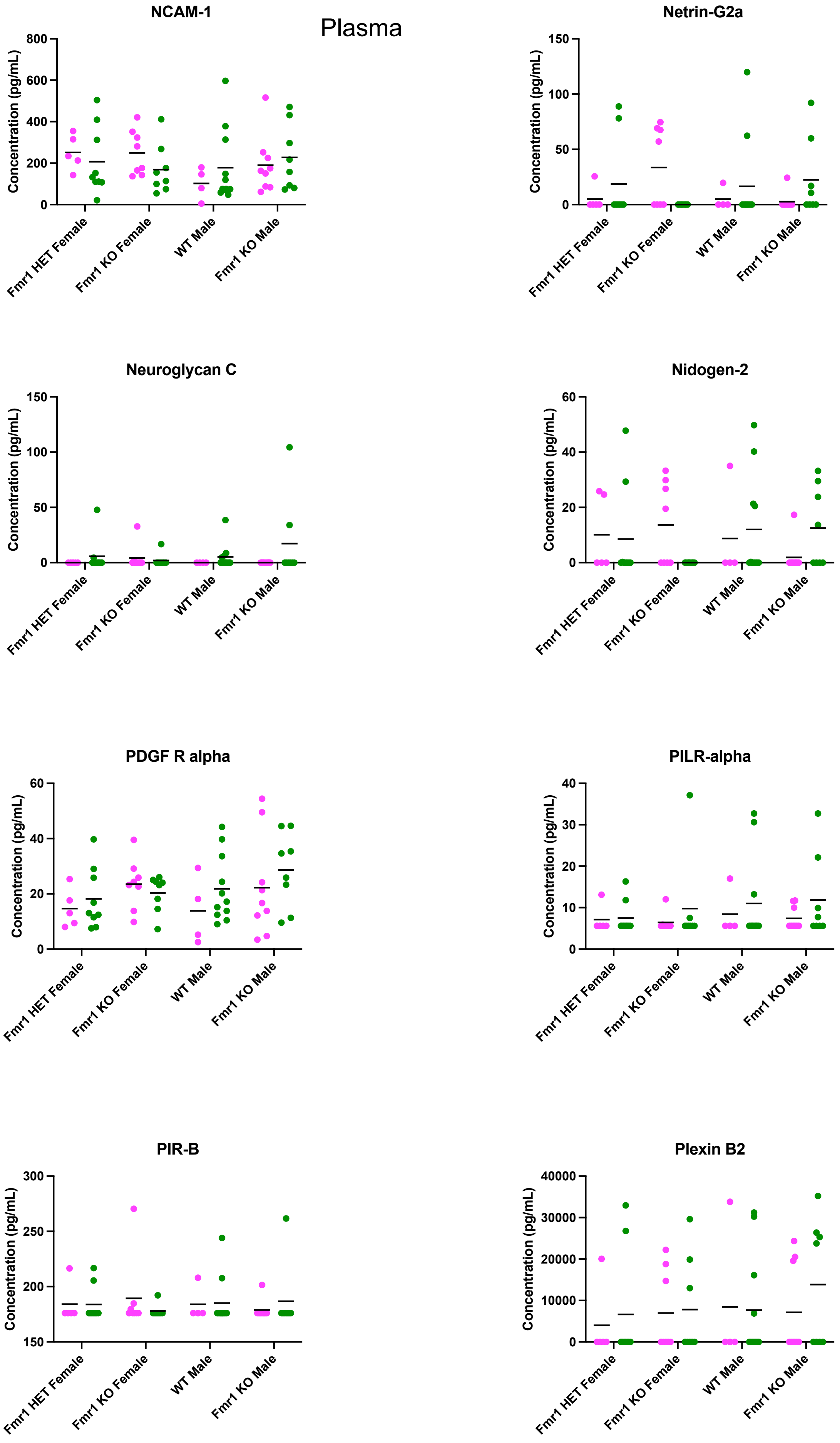
Plasma
NCAM-1
Netrin-G2a
Neuroglycan C
Nidogen-2
PDGF R alpha
PILR-alpha
PIR-B
Plexin B2
Concentration (pg/mL)
Fmr1 HET Female
Fmr1 KO Female
WT Male
Fmr1 KO Male
0
200
400
600
800
50
100
150
10
20
30
40
60
250
300
10000
20000
30000
40000
