# Supplementary material for: Soy Protein Isolate Affects Blood and Brain Biomarker Expression in a Mouse Model of Fragile X
Source: Int J Mol Sci. 2025 Jun 26;26(13):6137. doi: 10.3390/ijms26136137 (PMC12250412; doi:10.3390/ijms26136137)
Supplement: Supplementary file 1 [file ijms-26-06137-s001.zip › Supplementary File S17b Array 19 Graphs.pdf]

Supplementary File S17. Protein expression of Array 19 targets as function of *Fmr1* genotype and AIN-93G diets. Mice on AIN-93G/cas (colored pink) included n=5 *Fmr1*^{HET} female, n=8 *Fmr1*^{KO} female, n=4 WT male and n=9 *Fmr1*^{KO} male. Mice on AIN-93G/soy (colored green) included n=9 *Fmr1*^{HET} female, n=8 *Fmr1*^{KO} female, n=11 WT male and n=8 *Fmr1*^{KO} male. The average concentration in cortex, hippocampus, hypothalamus and plasma in pg/mL was plotted versus genotype. Statistics were determined by 2-way ANOVA and Tukey's multiple comparison tests denoted by $p < 0.05$ (*), $p < 0.01$ (**), $p < 0.001$ (***) and $p < 0.0001$ (****).

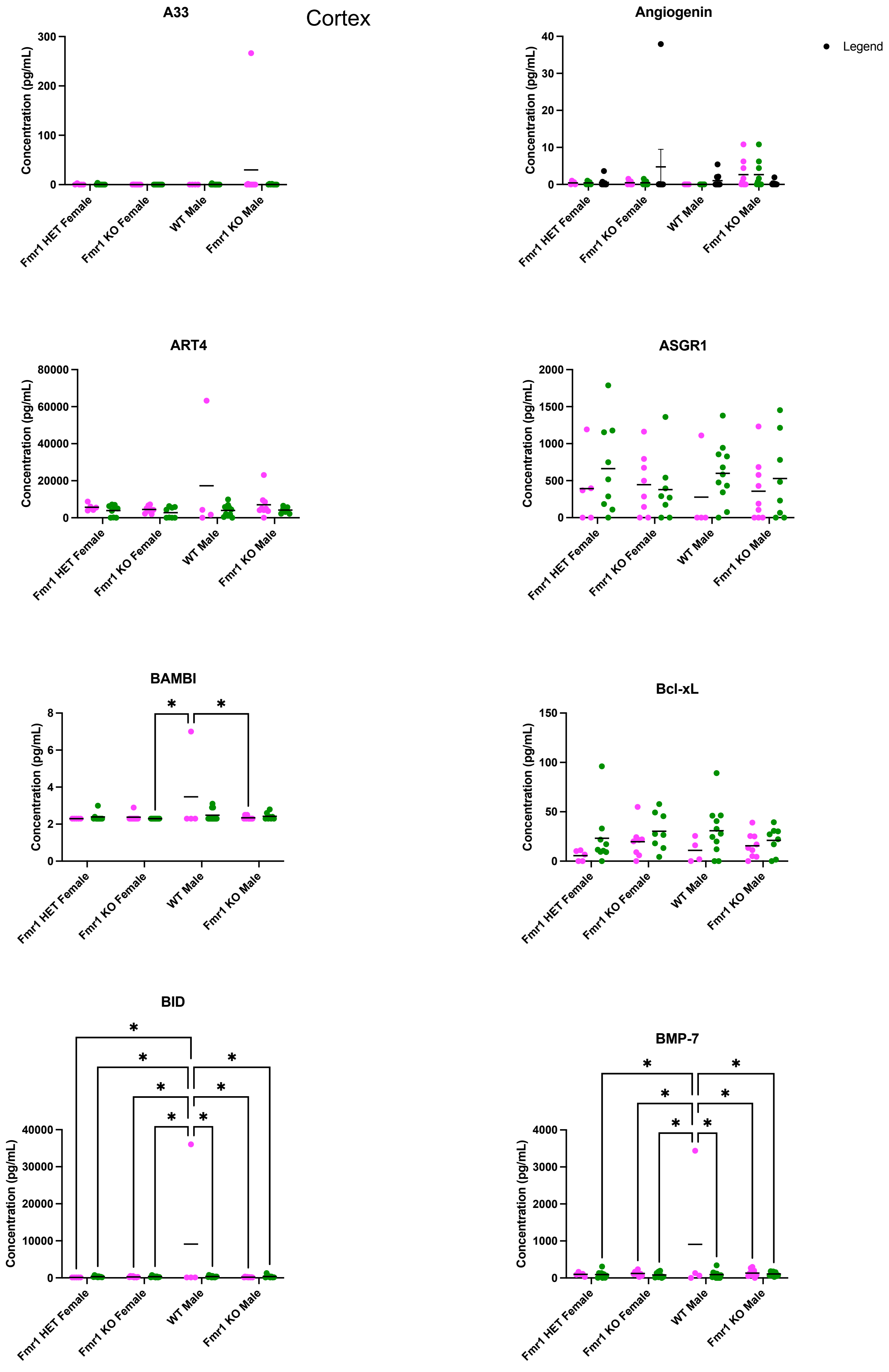

Cortex
A33
Angiogenin
Legend
ART4
ASGR1
BAMBI
Bcl-xL
BID
BMP-7
Concentration (pg/mL)
Fmr1 HET Female
Fmr1 KO Female
WT Male
Fmr1 KO Male
*

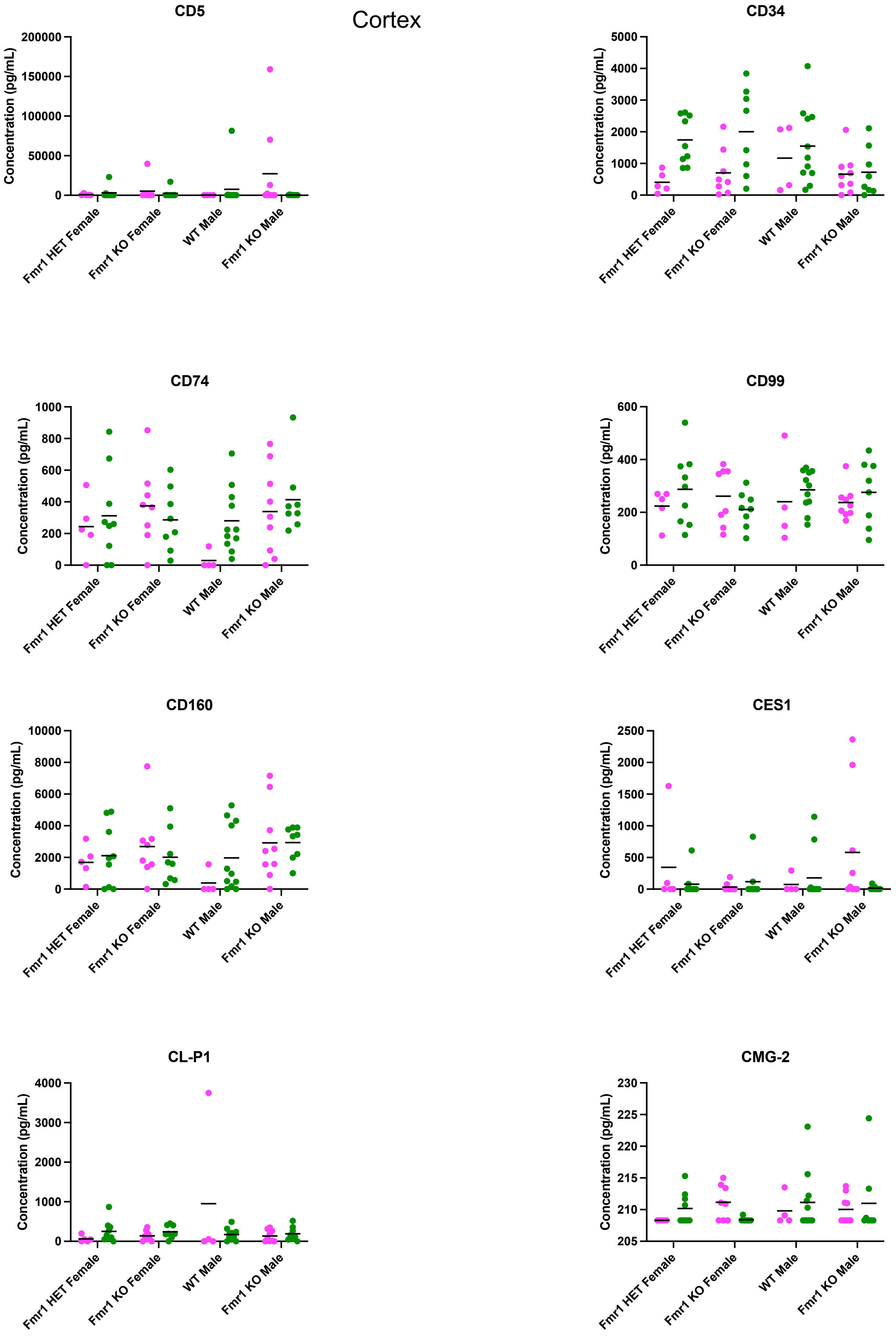
Cortex
CD5
CD34
CD74
CD99
CD160
CES1
CL-P1
CMG-2
Concentration (pg/mL)
0
50000
100000
150000
200000
1000
2000
3000
4000
5000
200
400
600
800
1000
6000
8000
10000
500
1500
2000
2500
205
210
215
220
225
230
Fmr1 HET Female
Fmr1 KO Female
WT Male
Fmr1 KO Male

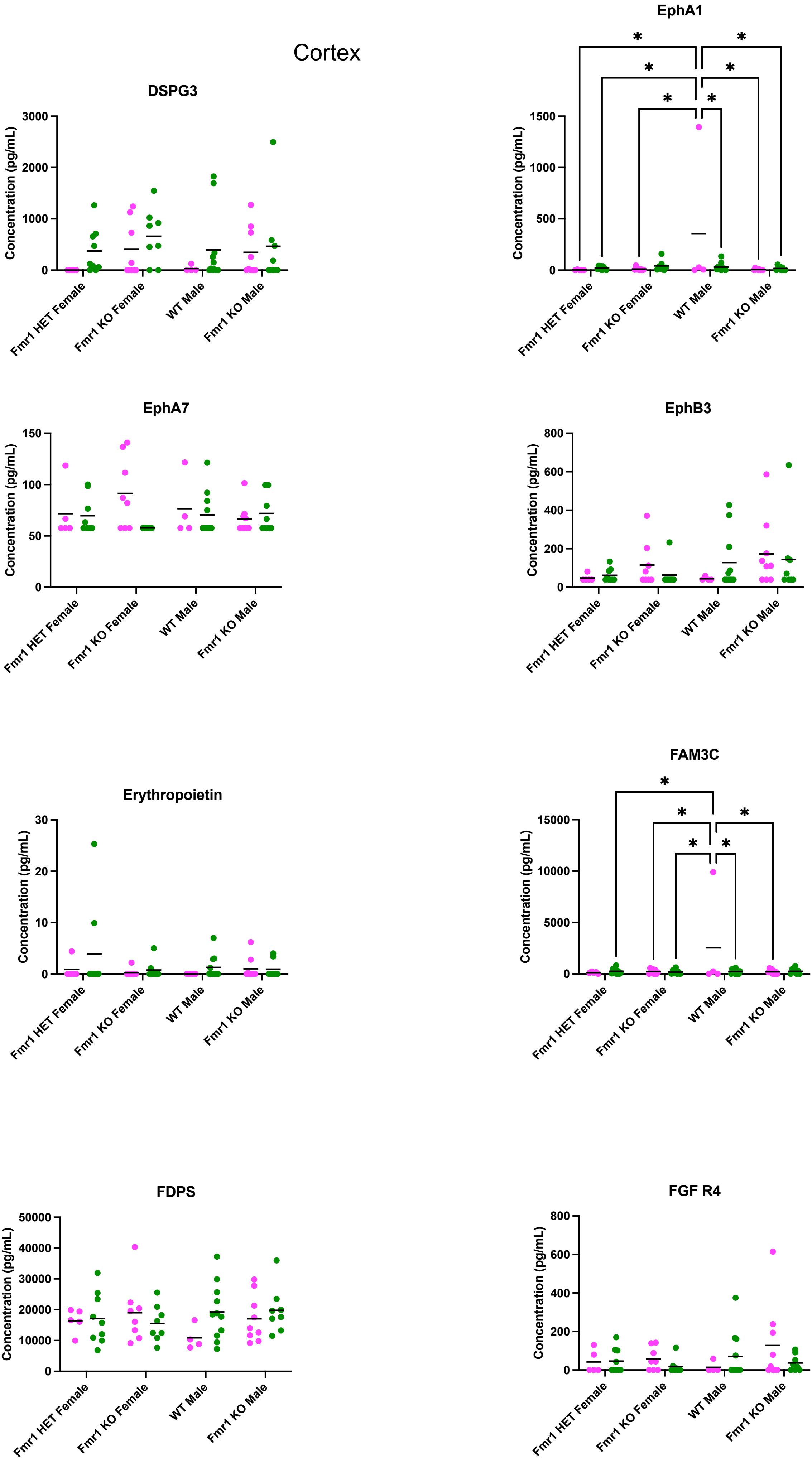

Cortex
DSPG3
EphA1
EphA7
EphB3
Erythropoietin
FAM3C
FDPS
FGF R4
Concentration (pg/mL)
Fmr1 HET Female
Fmr1 KO Female
WT Male
Fmr1 KO Male
0
1000
2000
3000
500
1500
50
100
150
200
400
600
800
10
20
30
5000
10000
15000
20000
30000
40000
50000
*

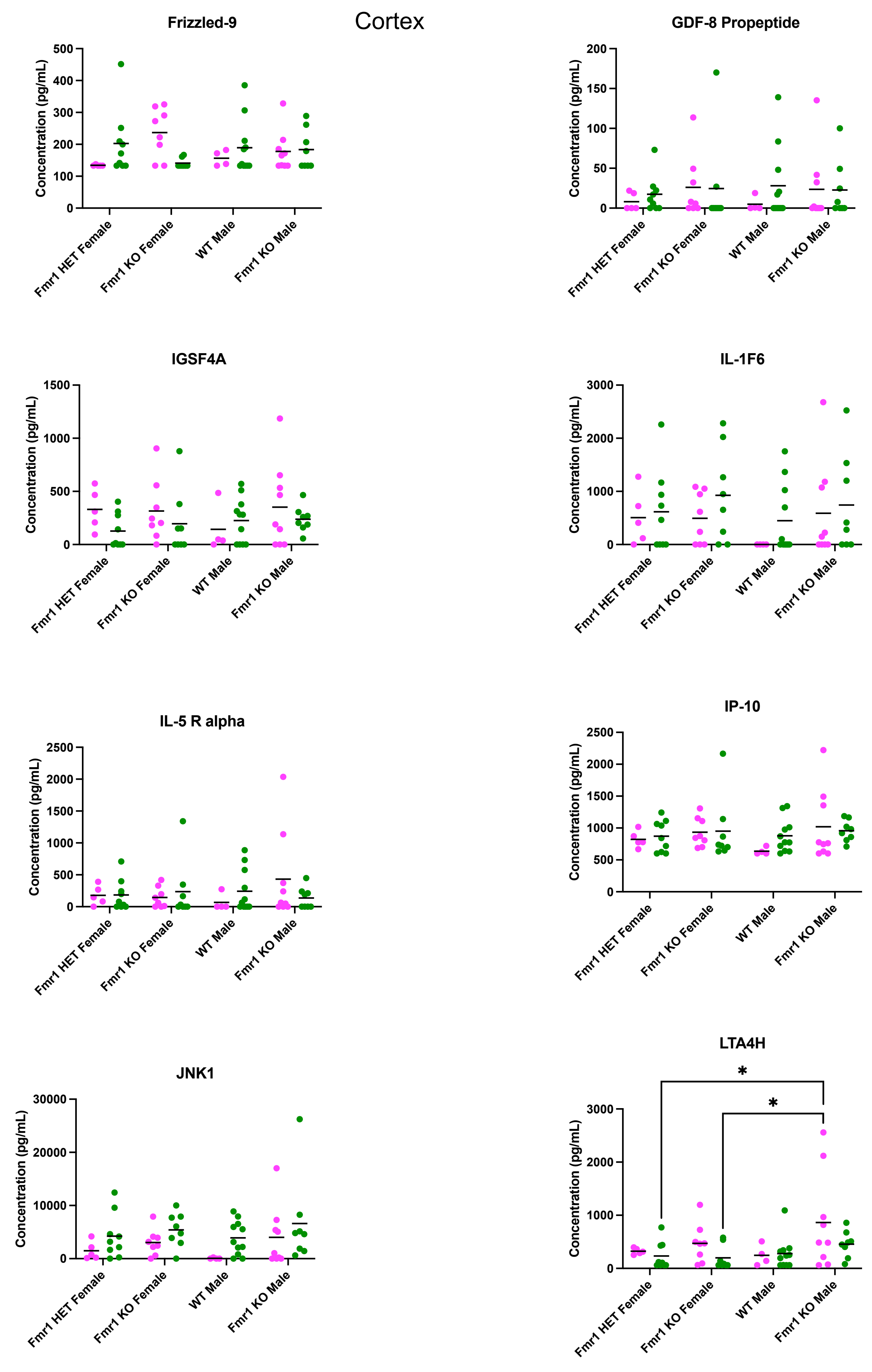

Cortex
Frizzled-9
GDF-8 Propeptide
IGSF4A
IL-1F6
IL-5 R alpha
IP-10
JNK1
LTA4H
Concentration (pg/mL)
0
100
200
300
400
500
50
150
1000
1500
2000
2500
3000
10000
20000
30000
Fmr1 HET Female
Fmr1 KO Female
WT Male
Fmr1 KO Male
*
*

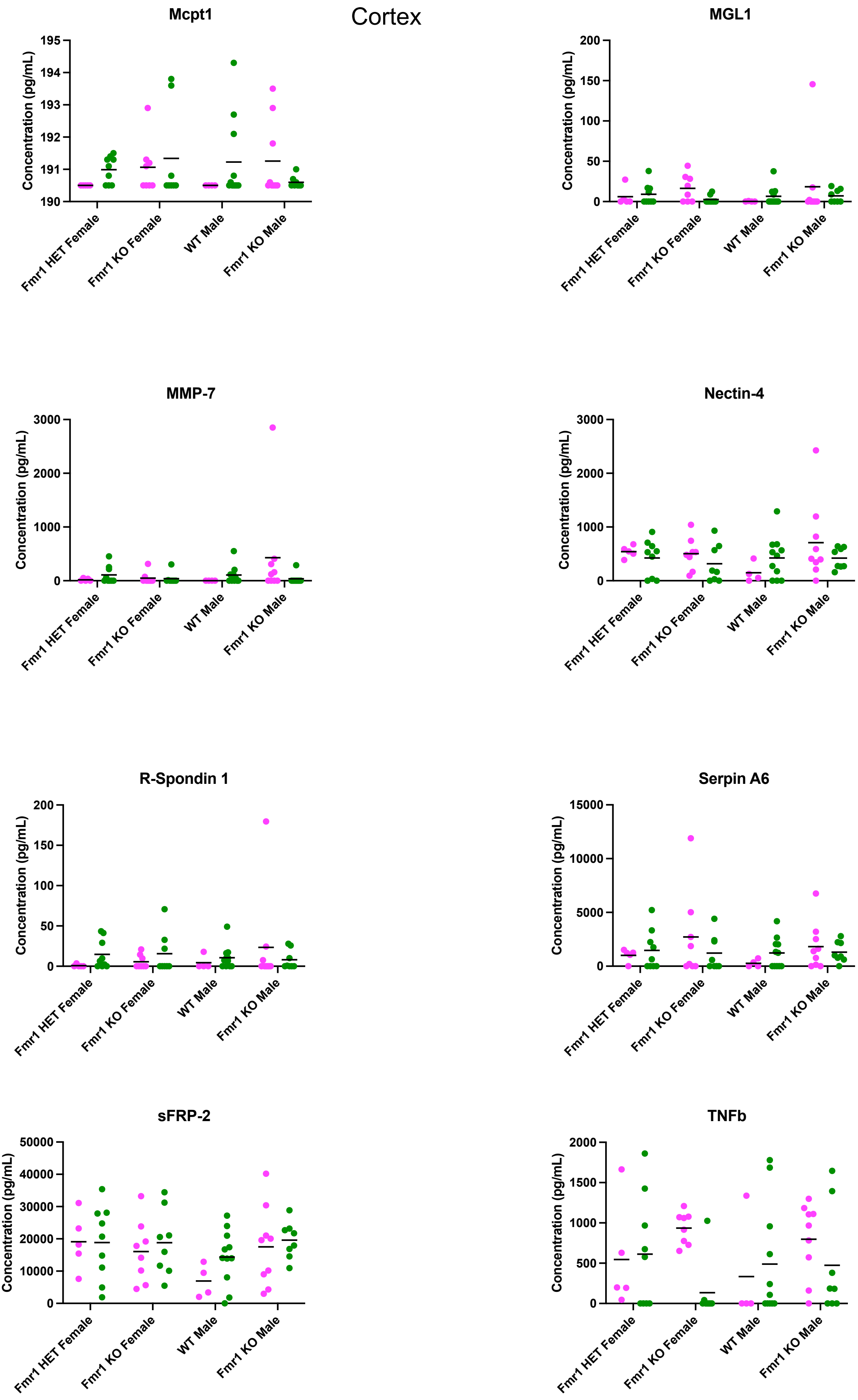
Cortex
Mcpt1
MGL1
MMP-7
Nectin-4
R-Spondin 1
Serpin A6
sFRP-2
TNFb
Concentration (pg/mL)
Fmr1 HET Female
Fmr1 KO Female
WT Male
Fmr1 KO Male
190
191
192
193
194
195
0
50
100
150
200
1000
2000
3000
5000
10000
15000
10000
20000
30000
40000
50000
500
1000
1500
2000

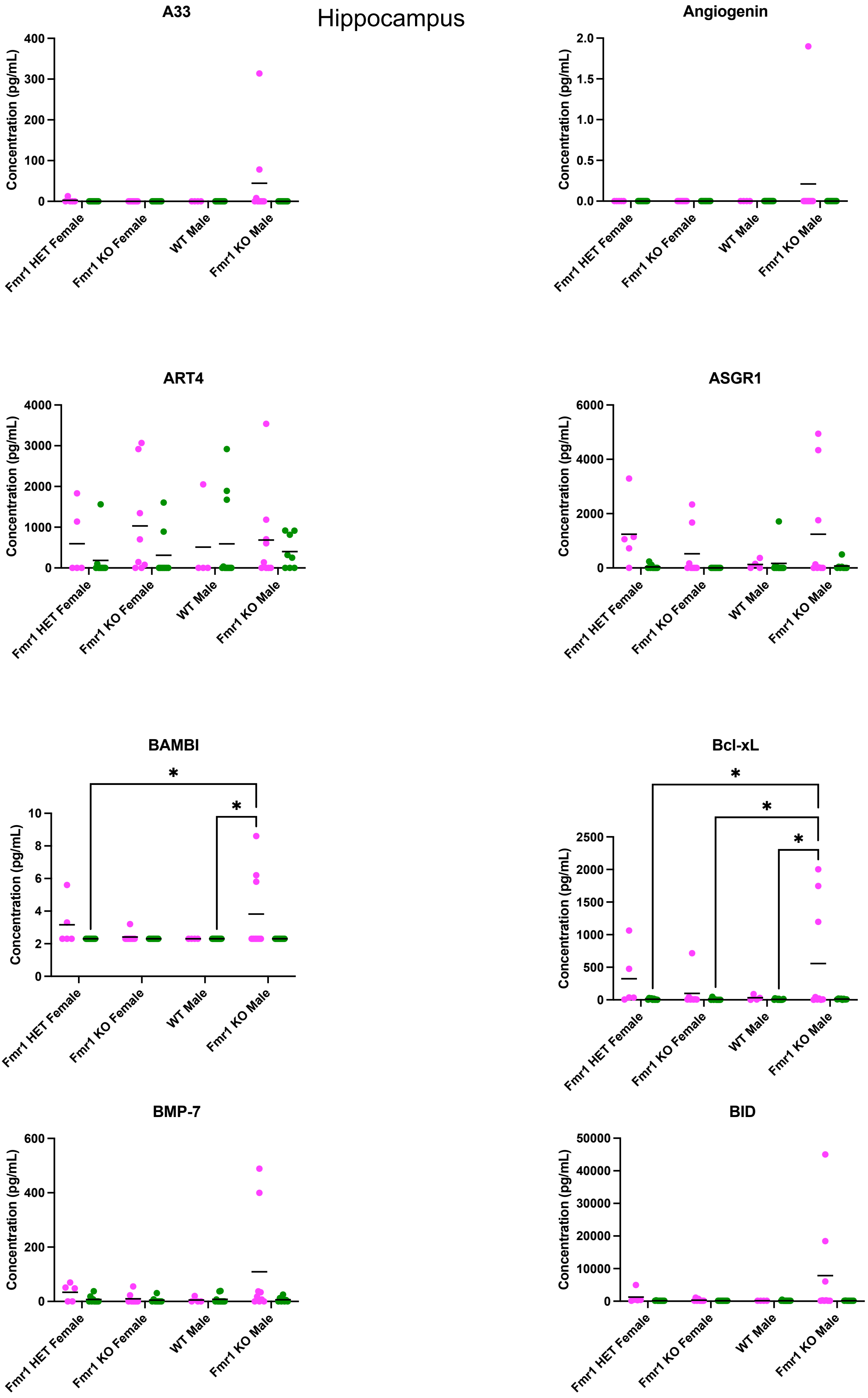

Hippocampus
A33
Angiogenin
ART4
ASGR1
BAMBI
Bcl-xL
BMP-7
BID
Concentration (pg/mL)
Fmr1 HET Female
Fmr1 KO Female
WT Male
Fmr1 KO Male
*

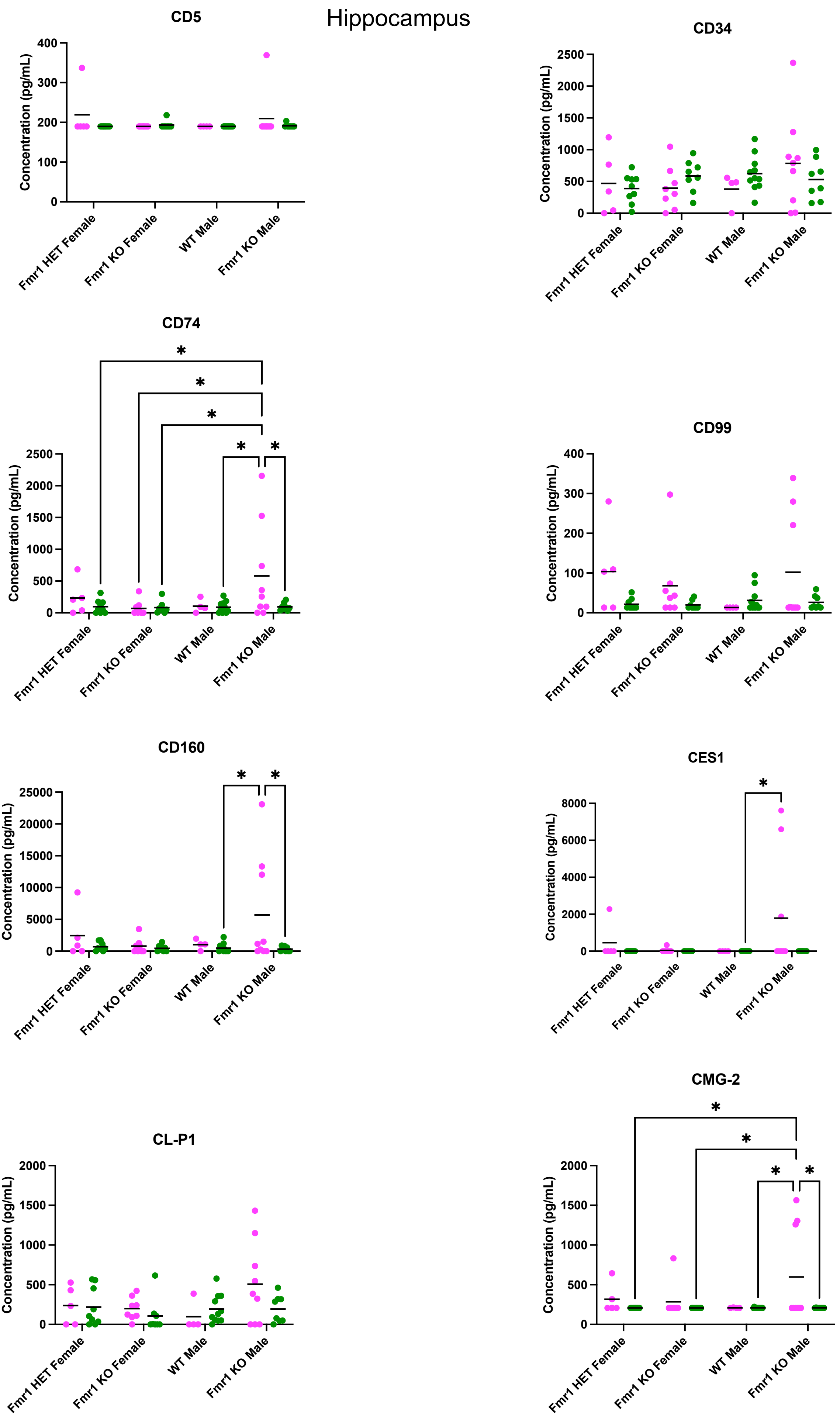
Hippocampus
CD5
CD34
CD74
CD99
CD160
CES1
CL-P1
CMG-2
Concentration (pg/mL)
Fmr1 HET Female
Fmr1 KO Female
WT Male
Fmr1 KO Male
*

Hippocampus

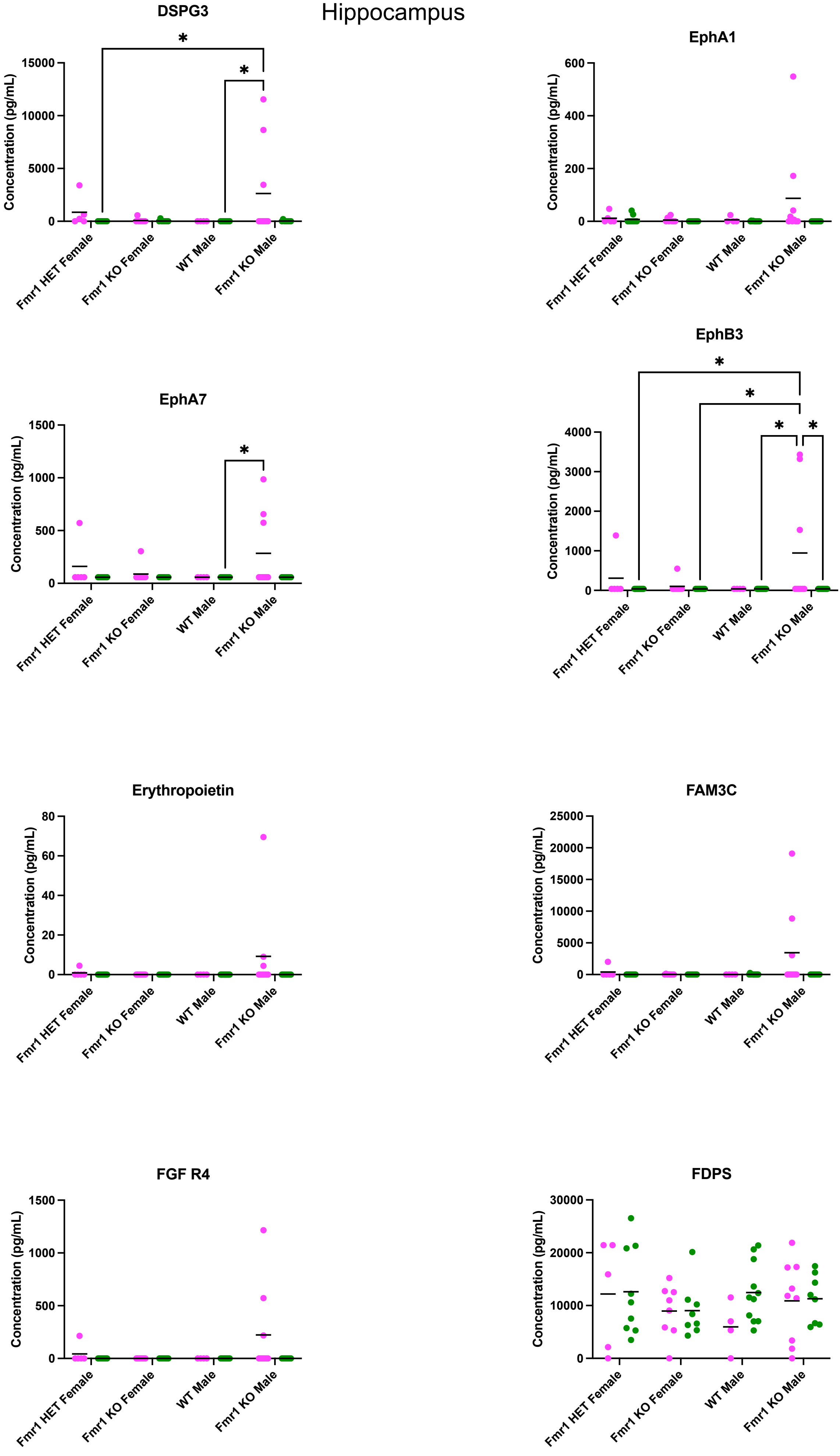

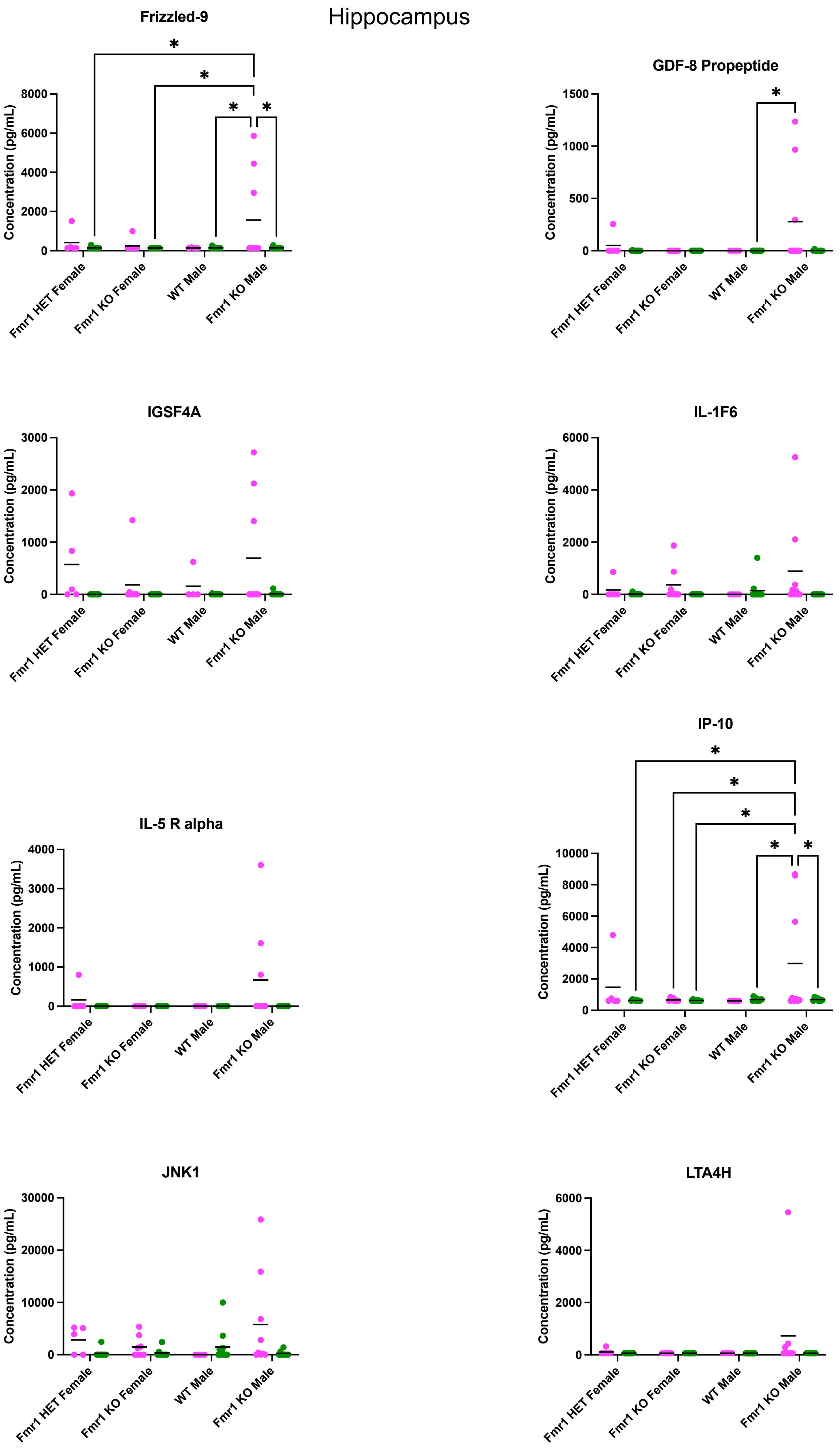
Hippocampus
Frizzled-9
GDF-8 Propeptide
IGSF4A
IL-1F6
IL-5 R alpha
IP-10
JNK1
LTA4H
Concentration (pg/mL)
Fmr1 HET Female
Fmr1 KO Female
WT Male
Fmr1 KO Male
*

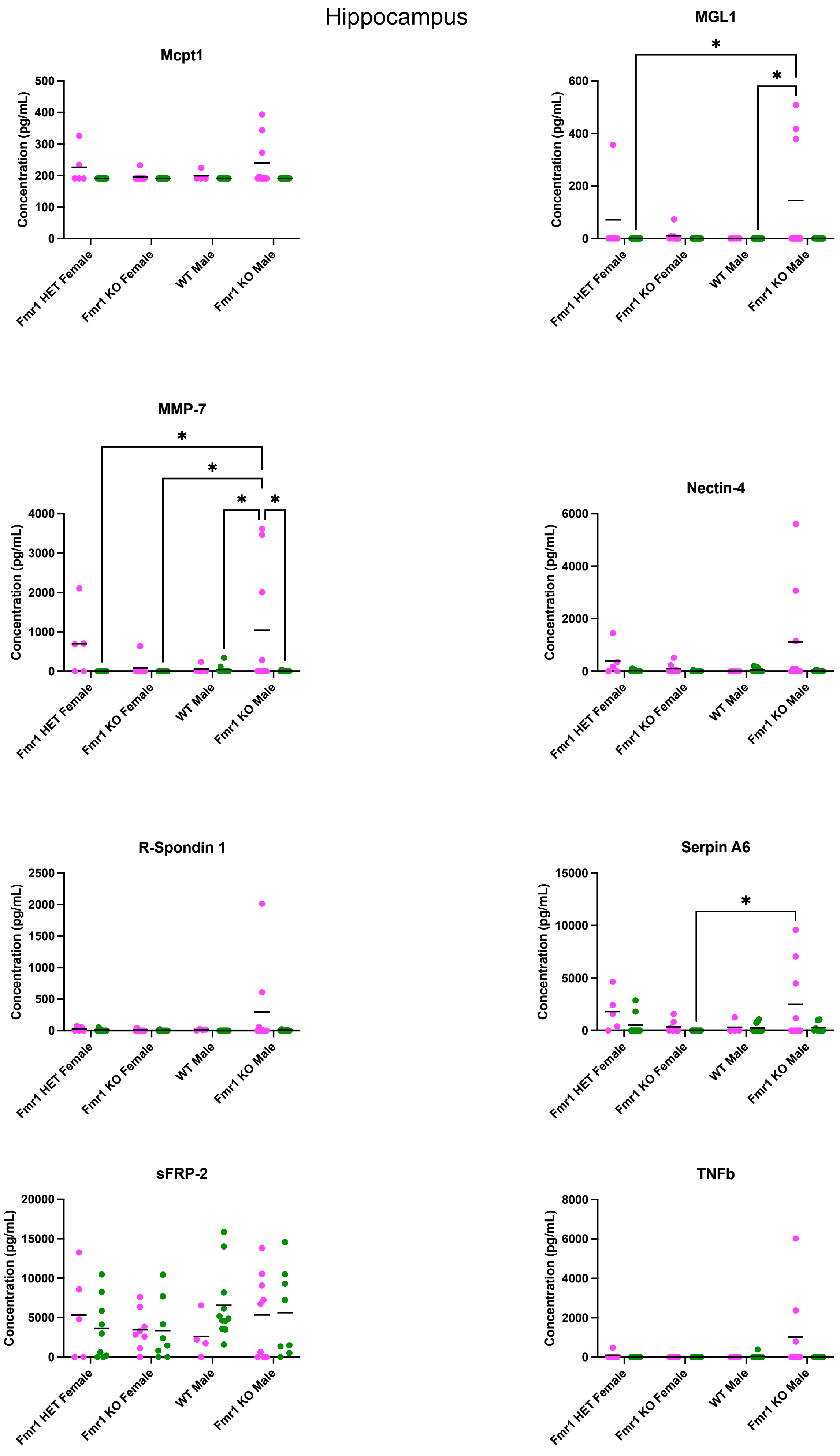

Hippocampus
Mcpt1
MGL1
MMP-7
Nectin-4
R-Spondin 1
Serpin A6
sFRP-2
TNFb
Concentration (pg/mL)
Fmr1 HET Female
Fmr1 KO Female
WT Male
Fmr1 KO Male
*

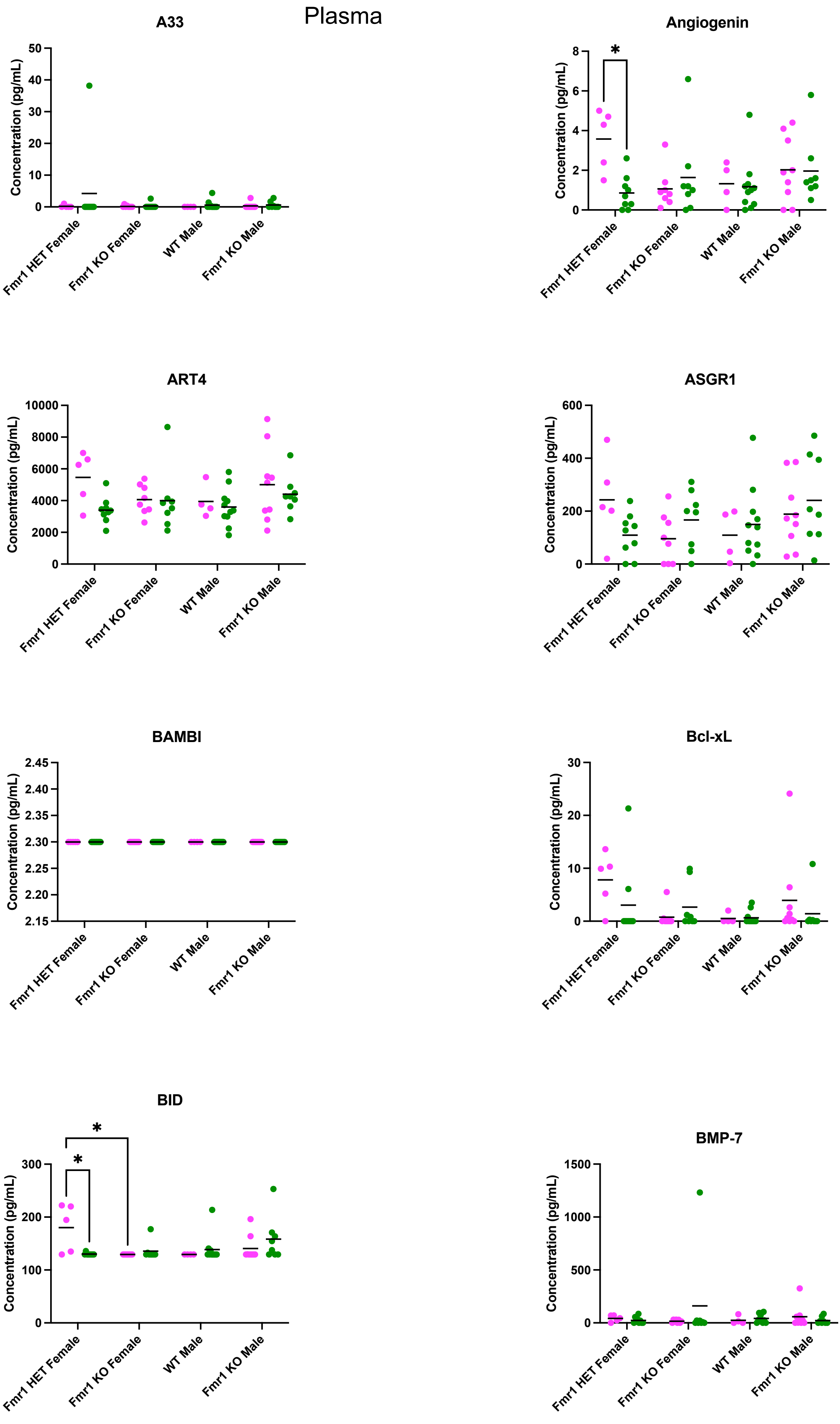

Plasma
A33
Angiogenin
ART4
ASGR1
BAMBI
Bcl-xL
BID
BMP-7
Concentration (pg/mL)
*
Fmr1 HET Female
Fmr1 KO Female
WT Male
Fmr1 KO Male

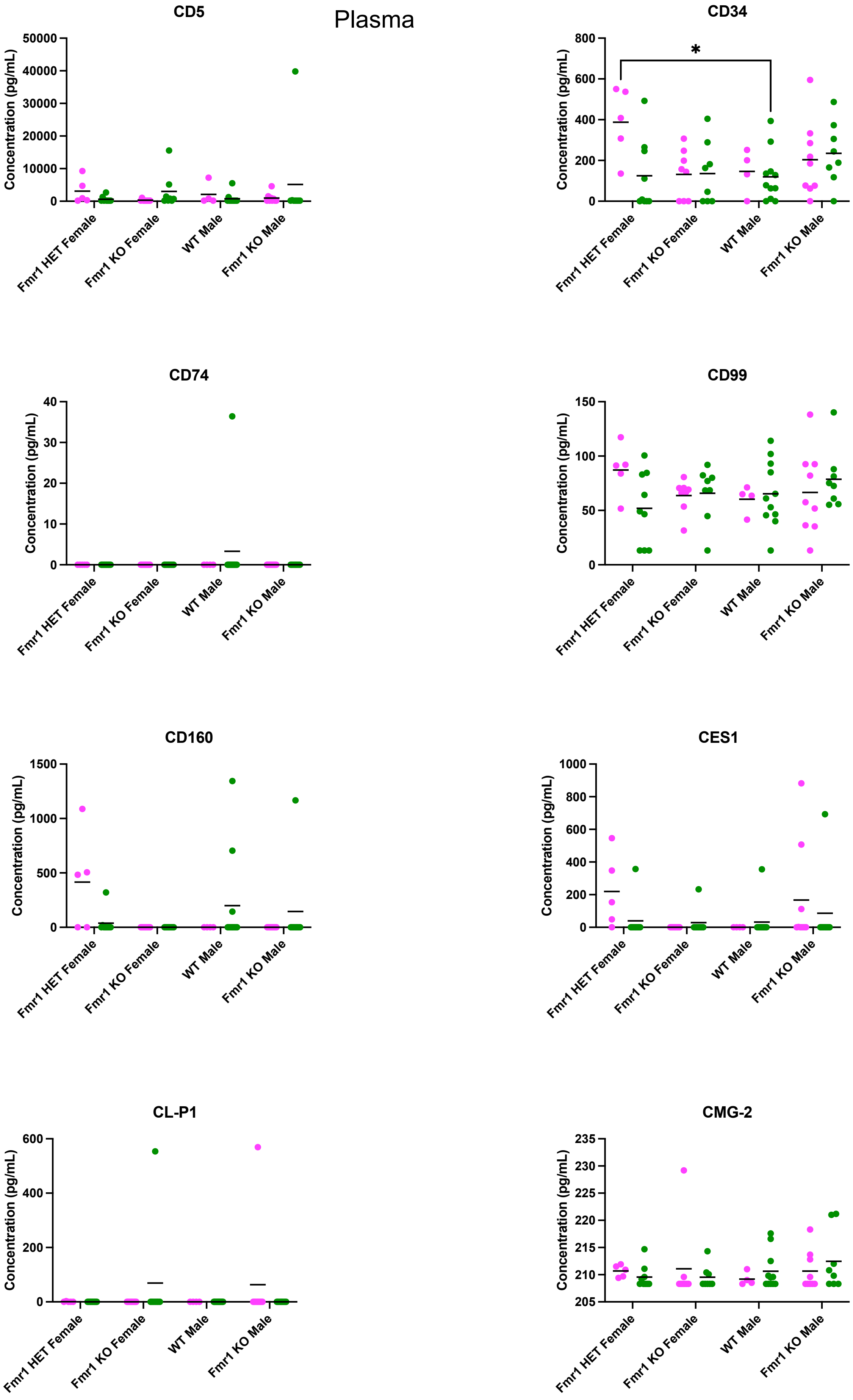
Plasma
CD5
CD34
CD74
CD99
CD160
CES1
CL-P1
CMG-2
Concentration (pg/mL)
*
Fmr1 HET Female
Fmr1 KO Female
WT Male
Fmr1 KO Male
0
10000
20000
30000
40000
50000
200
400
600
800
10
20
30
40
50
100
150
500
1000
1500
205
210
215
220
225
230
235

Plasma

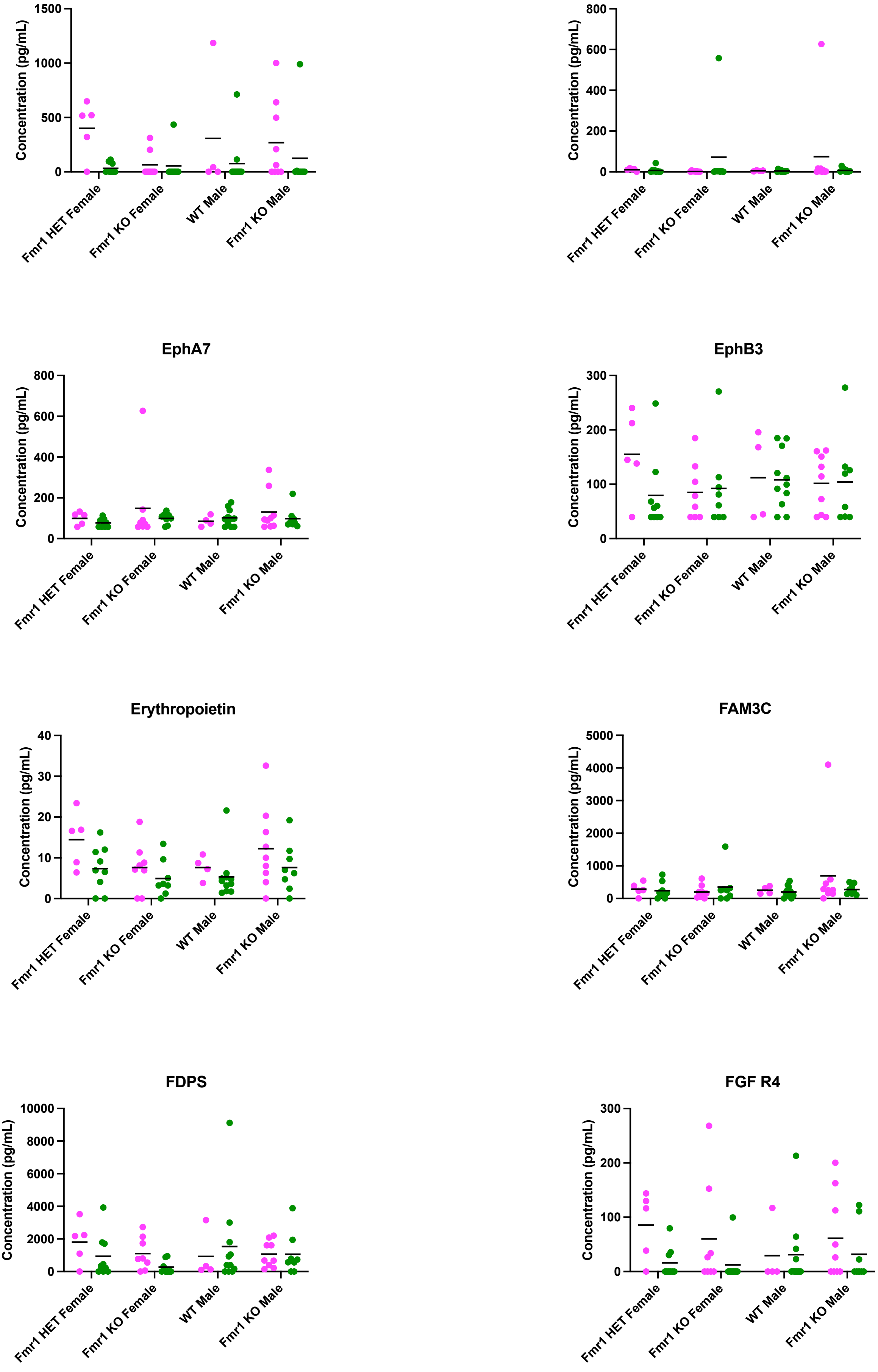

DSPG3
EphA1
EphA7
EphB3
Erythropoietin
FAM3C
FDPS
FGF R4
Concentration (pg/mL)
Fmr1 HET Female
Fmr1 KO Female
WT Male
Fmr1 KO Male

Plasma

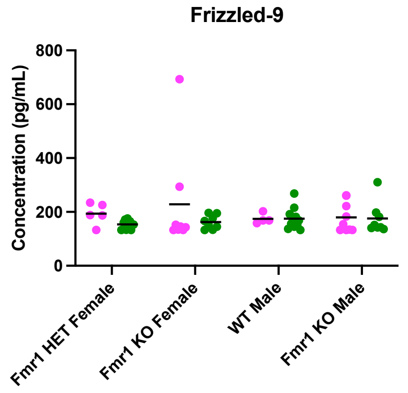
Frizzled-9
Concentration (pg/mL)
800
600
400
200
0
Fmr1 HET Female
Fmr1 KO Female
WT Male
Fmr1 KO Male

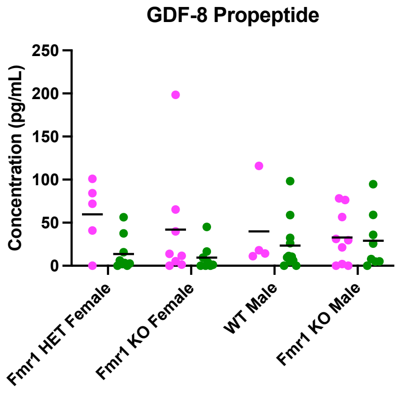
GDF-8 Propeptide
Concentration (pg/mL)
250
200
150
100
50
0
Fmr1 HET Female
Fmr1 KO Female
WT Male
Fmr1 KO Male

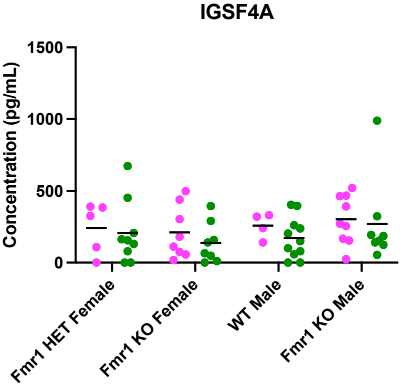
IGSF4A
Concentration (pg/mL)
1500
1000
500
0
Fmr1 HET Female
Fmr1 KO Female
WT Male
Fmr1 KO Male

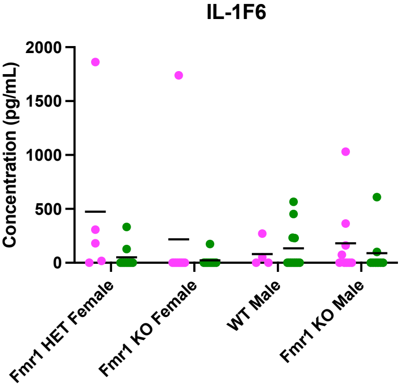
IL-1F6
Concentration (pg/mL)
2000
1500
1000
500
0
Fmr1 HET Female
Fmr1 KO Female
WT Male
Fmr1 KO Male

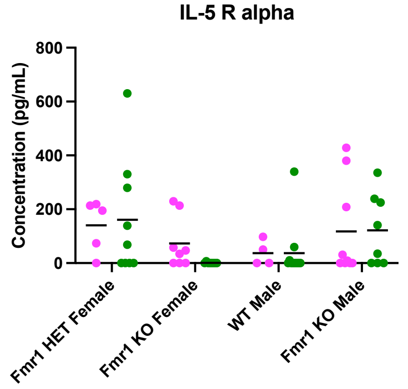
IL-5 R alpha
Concentration (pg/mL)
800
600
400
200
0
Fmr1 HET Female
Fmr1 KO Female
WT Male
Fmr1 KO Male

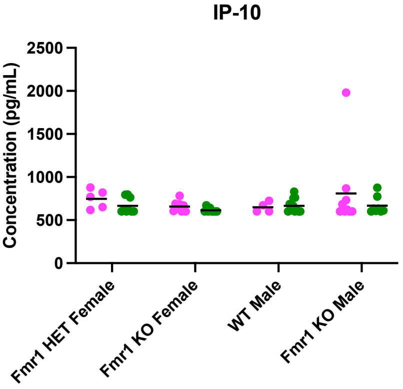
IP-10
Concentration (pg/mL)
2500
2000
1500
1000
500
0
Fmr1 HET Female
Fmr1 KO Female
WT Male
Fmr1 KO Male

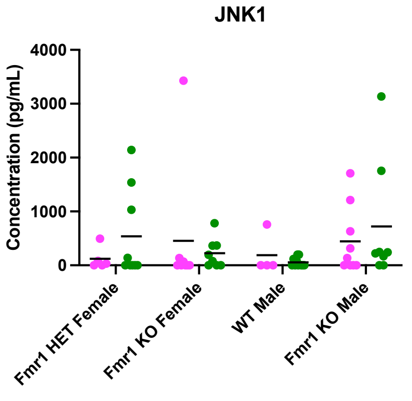
JNK1
Concentration (pg/mL)
4000
3000
2000
1000
0
Fmr1 HET Female
Fmr1 KO Female
WT Male
Fmr1 KO Male

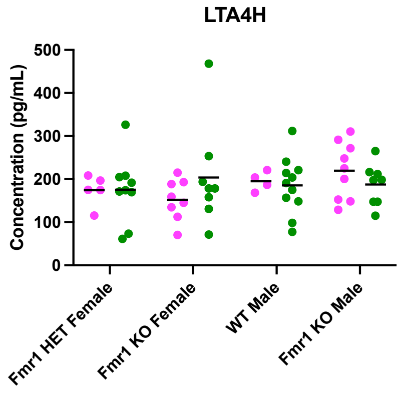
LTA4H
Concentration (pg/mL)
500
400
300
200
100
0
Fmr1 HET Female
Fmr1 KO Female
WT Male
Fmr1 KO Male

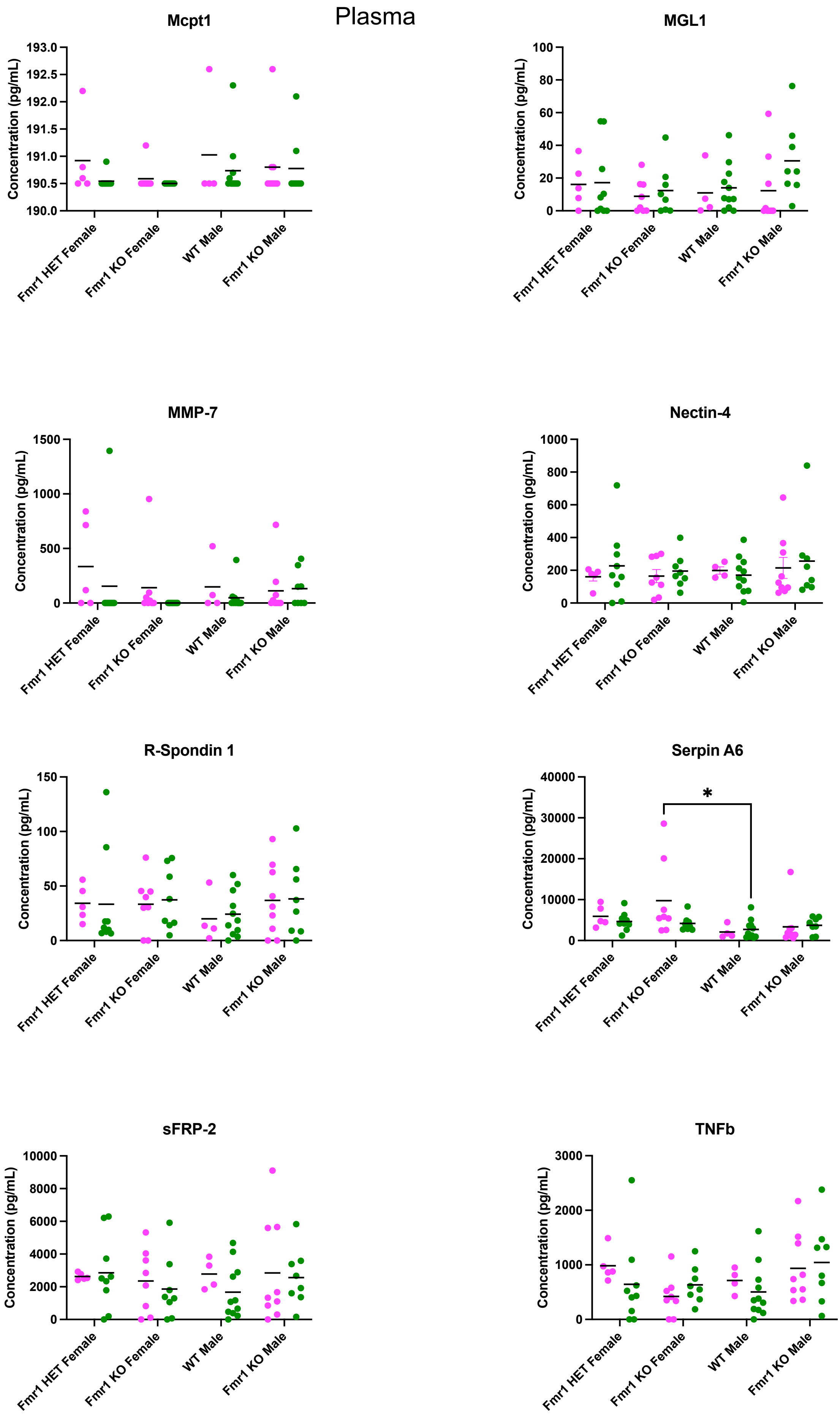

Plasma
Mcpt1
Concentration (pg/mL)
193.0
192.5
192.0
191.5
191.0
190.5
190.0
Fmr1 HET Female
Fmr1 KO Female
WT Male
Fmr1 KO Male
MGL1
Concentration (pg/mL)
100
80
60
40
20
0
Fmr1 HET Female
Fmr1 KO Female
WT Male
Fmr1 KO Male
MMP-7
Concentration (pg/mL)
1500
1000
500
0
Fmr1 HET Female
Fmr1 KO Female
WT Male
Fmr1 KO Male
Nectin-4
Concentration (pg/mL)
1000
800
600
400
200
0
Fmr1 HET Female
Fmr1 KO Female
WT Male
Fmr1 KO Male
R-Spondin 1
Concentration (pg/mL)
150
100
50
0
Fmr1 HET Female
Fmr1 KO Female
WT Male
Fmr1 KO Male
Serpin A6
Concentration (pg/mL)
40000
30000
20000
10000
0
*
Fmr1 HET Female
Fmr1 KO Female
WT Male
Fmr1 KO Male
sFRP-2
Concentration (pg/mL)
10000
8000
6000
4000
2000
0
Fmr1 HET Female
Fmr1 KO Female
WT Male
Fmr1 KO Male
TNFb
Concentration (pg/mL)
3000
2000
1000
0
Fmr1 HET Female
Fmr1 KO Female
WT Male
Fmr1 KO Male
