# Supplementary material for: Soy Protein Isolate Affects Blood and Brain Biomarker Expression in a Mouse Model of Fragile X
Source: Int J Mol Sci. 2025 Jun 26;26(13):6137. doi: 10.3390/ijms26136137 (PMC12250412; doi:10.3390/ijms26136137)
Supplement: Supplementary file 1 [file ijms-26-06137-s001.zip › Supplementary File S18.pdf]

Supplementary File S18. Top differentially expressed Quantibody proteins. Statistically significant comparisons (*p* values) are provided for 202 significant differences for targets from cortex (n = 47), hippocampus (n= 54), hypothalamus (n = 9), and plasma (n=92). Statistics were determined by 2-way ANOVA and Tukey's multiple comparison tests.

array	tissue	gene	genotype	P
4	ctx	IGFBP2	KOf v KOm cas	0.0401
4	ctx	OPG	KOf cas v soy	0.0483
4	ctx	OPG	KOf v KOm cas	0.035
4	ctx	SCF	KOf cas v soy	0.0271
4	ctx	SCF	KOf v KOm cas	0.0169
4	hipp	EGF	KOf v WT cas	0.0442
4	hypo	EGF	KOf v KOm soy	0.0449
4	hypo	EGF	KOm cas v soy	0.041
4	pls	CXCL16	HET v KOf soy	0.0085
4	pls	E-selectin	HET v WT soy	0.0429
4	pls	E-selectin	KOf v WT soy	0.0247
4	pls	IGFBP5	KOf v KOm cas	0.0493
4	pls	IL-17F	KOf cas v soy	0.0148
4	pls	IL-20	KOf cas v soy	0.0412
4	pls	IL-20	KOf v KOm cas	0.0251
4	pls	Prolactin	HET v KOf cas	0.0462
4	pls	Prolactin	KOf v WT cas	0.017
4	pls	Prolactin	KOf v KOm cas	0.0007
4	pls	Resistin	HET v WT soy	0.004
4	pls	Resistin	HET v KOm soy	0.0045
4	pls	Resistin	KOf v WT soy	0.0173
4	pls	Resistin	KOf v KOm soy	0.0167
5	ctx	Eotaxin	KOf cas v soy	0.0487
5	ctx	Eotaxin	KOf v KOm cas	0.0406
5	ctx	IL-1b	HET v WT soy	0.0107
5	ctx	KC	KOf v KOm cas	0.0459
5	ctx	Leptin	KOf cas v soy	0.0396
5	ctx	Leptin	KOf v KOm cas	0.0326
5	ctx	MIG	KOf cas v soy	0.0295
5	ctx	MIG	KOf v KOm cas	0.026
5	hypo	IL-3	KOf v WT soy	0.0294
5	pls	bFGF	HET v KOm soy	0.0396
5	pls	bFGF	KOf v KOm soy	0.0353
5	pls	bFGF	KOm cas v soy	0.0154
5	pls	IL-12p40	HET v KOf cas	0.0111
5	pls	IL-12p40	HET v KOm cas	0.0087
5	pls	TNF R1	HET v KOf cas	0.0094
5	pls	TNF R1	KOf v WT cas	0.021
5	pls	TNF R1	KOf cas v soy	0.0415
6	ctx	CT-1	KOf cas v soy	0.0313
6	ctx	CT-1	KOf v KOm cas	0.0228
6	ctx	IL-3 Rb	KOf cas v soy	0.0333
6	hypo	TWEAK R	KOf cas v soy	0.0475
6	hypo	TWEAK R	KOf v KOm cas	0.0374

6	pls	GAL-3	HET v WT soy	0.0197
6	pls	GAL-3	HET v KOm soy	0.0039
6	pls	GAL-3	KOf v WT soy	0.0396
6	pls	GAL-3	KOf v KOm soy	0.0083
6	pls	JAM-A	HET v KOm soy	0.0173
6	pls	JAM-A	KOf v KOm soy	0.0016
6	pls	JAM-A	WT v KOm soy	0.0329
6	pls	JAM-A	KOm cas v soy	0.0368
7	ctx	CCL6	HET v KOm cas	0.022
7	ctx	CysC	HET v KOm soy	0.0106
7	ctx	CysC	WT v KOm soy	0.02
7	hypo	NOV	HET v WT soy	0.0046
7	pls	DAN	KOf v WT cas	0.0467
7	pls	DAN	KOf v KOm cas	0.0202
7	pls	Lipocalin-2	HET v WT soy	0.001
7	pls	Lipocalin-2	KOf v WT soy	0.0147
7	pls	Marapsin	HET v WT cas	0.0235
7	pls	MBL-2	HET v WT cas	0.0052
7	pls	MBL-2	HET v KOm cas	0.0221
7	pls	NOV	KOf v KOm cas	0.0485
8	hipp	MMP-10	HET v KOm cas	0.047
8	hipp	TRANCE	HET cas v soy	0.0346
8	hipp	TRANCE	HET v KOf cas	0.0342
8	hipp	TRANCE	HET v KOm cas	0.031
8	hypo	MMP-3	KOm cas v soy	0.0198
8	hypo	Persephin	KOm cas v soy	0.045
8	hypo	TCK-1	KOf cas v soy	0.0204
8	pls	Epiregulin	HET cas v soy	0.0037
8	pls	Epiregulin	HET v KOf cas	0.0183
8	pls	Epiregulin	HET v KOm cas	0.0022
8	pls	Persephin	HET cas v soy	0.0485
8	pls	Persephin	HET v KOm cas	0.0376
8	pls	SLAM	HET cas v soy	0.0137
8	pls	SLAM	HET v KOf cas	0.0061
8	pls	SLAM	HET v WT cas	0.0367
8	pls	SLAM	HET v KOm cas	0.0181
8	pls	TRANCE	HET cas v soy	0.0453
8	pls	TRANCE	HET v KOm cas	0.0409
9	ctx	B7-2	HET cas v soy	0.0364
9	ctx	CD99-L2	WT v KOm cas	0.0311
9	hipp	CD45	KOf v WT cas	0.0331
9	hipp	CD45	WT cas v soy	0.0252
9	pls	BAFF	KOf cas v soy	0.0168
10	ctx	CD14	HET cas v soy	0.0037
10	hipp	Follistatin	HET v KOm soy	0.0229

10	hipp	Follistatin	KOf v KOm soy	0.0425
10	hipp	Follistatin	WT v KOm soy	0.012
10	hipp	IL-1F8	KOf cas v soy	0.0083
10	hipp	IL-15 R alpha	KOf cas v soy	0.003
10	hipp	IL-15 R alpha	KOf v WT cas	0.0262
10	hipp	IL-15 R alpha	KOf v KOm cas	0.0039
10	hipp	IL-20 R beta	KOf v KOm cas	0.0313
10	hipp	Klotho beta	KOf cas v soy	0.002
10	hipp	Klotho beta	KOf v WT cas	0.016
10	hipp	Klotho beta	KOf v KOm cas	0.0015
10	hipp	LIF	KOf v KOm cas	0.0207
11	hipp	CD4	KOf v KOm soy	0.0339
11	hipp	CD4	KOm cas v soy	0.0039
11	hipp	EpCAM	HET v KOm soy	0.0129
11	hipp	EpCAM	WT v KOm soy	0.0106
11	hipp	EpCAM	KOm cas v soy	0.0079
11	hipp	EphA5	KOm cas v soy	0.0318
11	hipp	FGF4	HET v KOm soy	0.0414
11	hipp	FGF4	KOm cas v soy	0.0159
11	hipp	FGF6	WT v KOm soy	0.0389
11	hipp	FGF6	KOm cas v soy	0.0163
11	hipp	FGF10	WT v KOm soy	0.0409
12	ctx	Olfactomedian-1	HET cas v soy	0.0252
12	ctx	Olfactomedian-1	HET v KOf cas	0.0458
12	ctx	Podocalyxin	HET v KOf soy	0.0406
12	hipp	Prss34	HET cas v soy	0.002
12	hipp	Prss34	HET v KOf cas	0.016
12	hipp	Prss34	HET v KOm cas	0.0042
12	pls	ASAM	KOf cas v soy	0.0106
12	pls	ASAM	KOf v WT cas	0.0209
12	pls	ASAM	KOf v KOm cas	0.0039
13	pls	RGM-C	HET v KOm soy	0.0489
13	pls	SEMA3C	HET v KOm soy	0.0041
13	pls	SEMA3C	KOf v KOm soy	0.0042
13	pls	SEMA3C	WT v KOm soy	0.0127
13	pls	SEMA3C	KOm cas v soy	0.0044
13	pls	Siglec-3	KOf cas v soy	0.0147
13	pls	Siglec-3	KOf v KOm cas	0.0371
13	pls	SR-A1	WT v KOm soy	0.0428
13	pls	SR-A1	KOm cas v soy	0.0057
13	pls	TIM-3	KOf cas v soy	0.017
13	pls	TIM-3	KOf v KOm cas	0.0211
13	pls	uPAR	KOf v KOm cas	0.0097
14	ctx	CD164	HET v KOm soy	0.0285
14	ctx	CD164	KOm cas v soy	0.0292

14	hipp	CHST4	HET v KOm cas	0.0132
14	hipp	S100A9	KOf v KOm soy	0.0395
14	hipp	S100A9	KOm cas v soy	0.0275
14	hipp	Tie-2	HET v KOm soy	0.0357
14	pls	VLDL R	HET v KOf soy	0.0389
14	pls	VLDL R	KOf cas v soy	0.0078
15	ctx	CAMK4	HET cas v soy	0.0445
15	hipp	NAALADL1	KOf v KOm soy	0.0491
15	hipp	NAALADL1	WT v KOm soy	0.0496
15	pls	BCHE	KOf cas v soy	0.0152
15	pls	BCHE	KOf v KOm cas	0.0344
16	hipp	BMP-6	KOf cas v soy	0.021
16	hipp	SerpinB10	HET v KOm cas	0.0422
16	pls	CA2	HET cas v soy	0.0369
16	pls	CA2	HET v KOf cas	0.0422
16	pls	NTB-A	HET v KOf cas	0.0347
16	pls	NTB-A	KOf cas v soy	0.0107
16	pls	NTB-A	KOf v KOm cas	0.0113
16	pls	VNN1	HET v WT cas	0.03
16	pls	VNN1	HET v KOm cas	0.0022
16	pls	VNN1	KOm cas v soy	0.0459
17	ctx	GHR	WT cas v soy	0.0106
17	pls	Ephrin-B1	KOf v KOm soy	0.0109
17	pls	Ephrin-B1	KOm cas v soy	0.0275
17	pls	Galectin-4	KOf v KOm soy	0.0271
17	pls	Galectin-4	KOm cas v soy	0.0184
17	pls	GDF-9	HET v KOm soy	0.0176
17	pls	GDF-9	KOf v KOm soy	0.0062
17	pls	GDF-9	WT v KOm soy	0.0038
17	pls	GDF-9	KOm cas v soy	0.0468
17	pls	IL-22 R alpha 1	HET v KOm soy	0.0419
17	pls	IL-22 R alpha 1	KOf v KOm soy	0.0256
17	pls	IL-22 R alpha 1	KOm cas v soy	0.0325
17	pls	Netrin-4	HET cas v soy	0.0158
18	ctx	FCRL5	HET cas v soy	0.0127
18	ctx	FCRL5	HET v KOf cas	0.0204
18	ctx	FCRL5	HET v KOm cas	0.0248
18	hipp	EMMPRIN	HET v KOf soy	0.0367
18	hipp	EMMPRIN	KOf v WT soy	0.0246
18	hipp	EMMPRIN	KOf v KOm soy	0.0219
18	hipp	IL-30	KOf v KOm soy	0.0167
19	ctx	BAMBI	WT v KOm cas	0.0437
19	ctx	BID	HET v WT cas	0.0493
19	ctx	BID	KOf v WT cas	0.0255
19	ctx	BID	WT cas v soy	0.0177

19	ctx	BID	WT v KOm cas	0.0196
19	ctx	BMP-7	KOf v WT cas	0.0478
19	ctx	BMP-7	WT cas v soy	0.0228
19	ctx	BMP-7	WT v KOm cas	0.0457
19	ctx	EphA1	HET v WT cas	0.0448
19	ctx	EphA1	KOf v WT cas	0.025
19	ctx	EphA1	WT cas v soy	0.0284
19	ctx	EphA1	WT v KOm cas	0.0183
19	ctx	FAM3C	KOf v WT cas	0.0456
19	ctx	FAM3C	WT cas v soy	0.0299
19	ctx	FAM3C	WT v KOm cas	0.0355
19	hipp	CD74	KOf v KOm cas	0.0318
19	hipp	CD74	KOm cas v soy	0.0474
19	hipp	CD160	KOm cas v soy	0.0456
19	hipp	CMG-2	KOm cas v soy	0.0385
19	hipp	EphB3	KOm cas v soy	0.0484
19	hipp	Frizzled-9	KOm cas v soy	0.0392
19	hipp	IP-10	KOf v KOm cas	0.0399
19	hipp	IP-10	KOm cas v soy	0.0428
19	hipp	MMP-7	KOm cas v soy	0.0362
19	pls	Angiongenin	HET cas v soy	0.0312
19	pls	BID	HET cas v soy	0.0138
19	pls	BID	HET v KOf cas	0.0149
