# Supplementary material for: Soy Protein Isolate Affects Blood and Brain Biomarker Expression in a Mouse Model of Fragile X
Source: Int J Mol Sci. 2025 Jun 26;26(13):6137. doi: 10.3390/ijms26136137 (PMC12250412; doi:10.3390/ijms26136137)
Supplement: Supplementary file 1 [file ijms-26-06137-s001.zip › Supplementary File S19.pdf]

Supplementary File S19. Differentially expressed proteins in hypothalamus and hippocampus comparing mass spectrometry with Quantibody arrays. Data is displayed as a function of *Fmr1* genotype and diet. Mass spectrometry data included 3 biological and 3 technical replicates for each cohort of WT and $Fmr1^{KO}$ male mice on AIN-93-G/casein and AIN-93G/soy diets with average Intensity, Log2 Normalized plotted versus genotype/diet cohort. Quantibody array data included n=5 $Fmr1^{HET}$ female, n=8 $Fmr1^{KO}$ female, n=4 WT male and n=9 $Fmr1^{KO}$ male mice maintained on AIN-93G/casein and n=9 $Fmr1^{HET}$ female, n=8 $Fmr1^{KO}$ female, n=11 WT male and n=8 $Fmr1^{KO}$ male mice maintained on AIN-03G/soy with average concentration in pg/mL plotted versus genotype. AIN-93G/casein data is colored pink. AIN-93G/soy data is colored green. Merging the shotgun proteomics and Quantibody datasets identified overlap in expression of 33 proteins. Statistics were determined by 2-way ANOVA and Tukey's multiple comparison tests where significant differences are denoted by $p < 0.05$ (*), $p < 0.01$ (**), $p < 0.001$ (***) and $p < 0.0001$ (****).

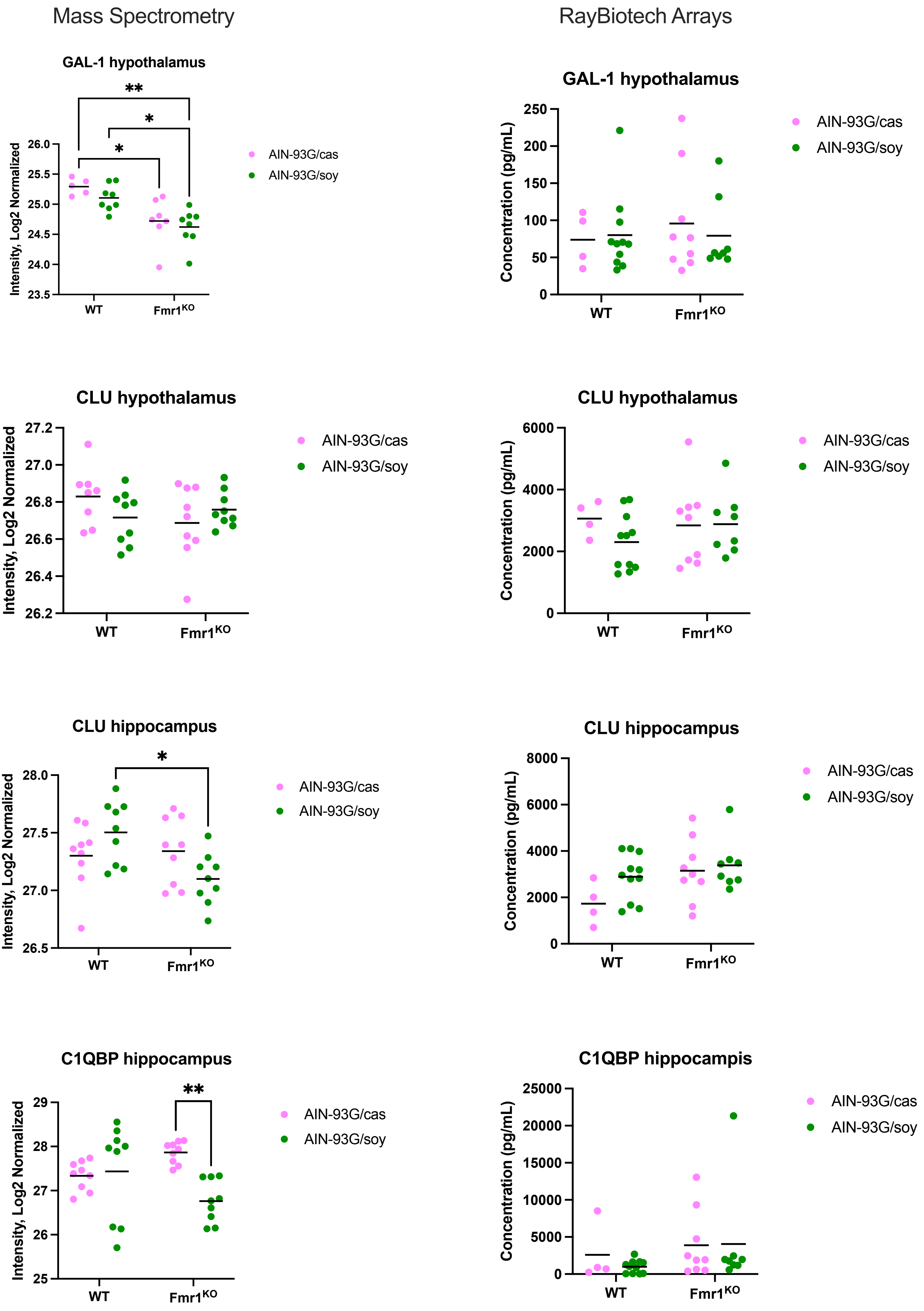

Mass Spectrometry
RayBiotech Arrays
GAL-1 hypothalamus
Intensity, Log2 Normalized
WT
Fmr1KO
AIN-93G/cas
AIN-93G/soy
**
*
*
GAL-1 hypothalamus
Concentration (pg/mL)
WT
Fmr1KO
AIN-93G/cas
AIN-93G/soy
CLU hypothalamus
Intensity, Log2 Normalized
WT
Fmr1KO
AIN-93G/cas
AIN-93G/soy
CLU hypothalamus
Concentration (pg/mL)
WT
Fmr1KO
AIN-93G/cas
AIN-93G/soy
CLU hippocampus
Intensity, Log2 Normalized
*
WT
Fmr1KO
AIN-93G/cas
AIN-93G/soy
CLU hippocampus
Concentration (pg/mL)
WT
Fmr1KO
AIN-93G/cas
AIN-93G/soy
C1QBP hippocampus
Intensity, Log2 Normalized
**
WT
Fmr1KO
AIN-93G/cas
AIN-93G/soy
C1QBP hippocampis
Concentration (pg/mL)
WT
Fmr1KO
AIN-93G/cas
AIN-93G/soy

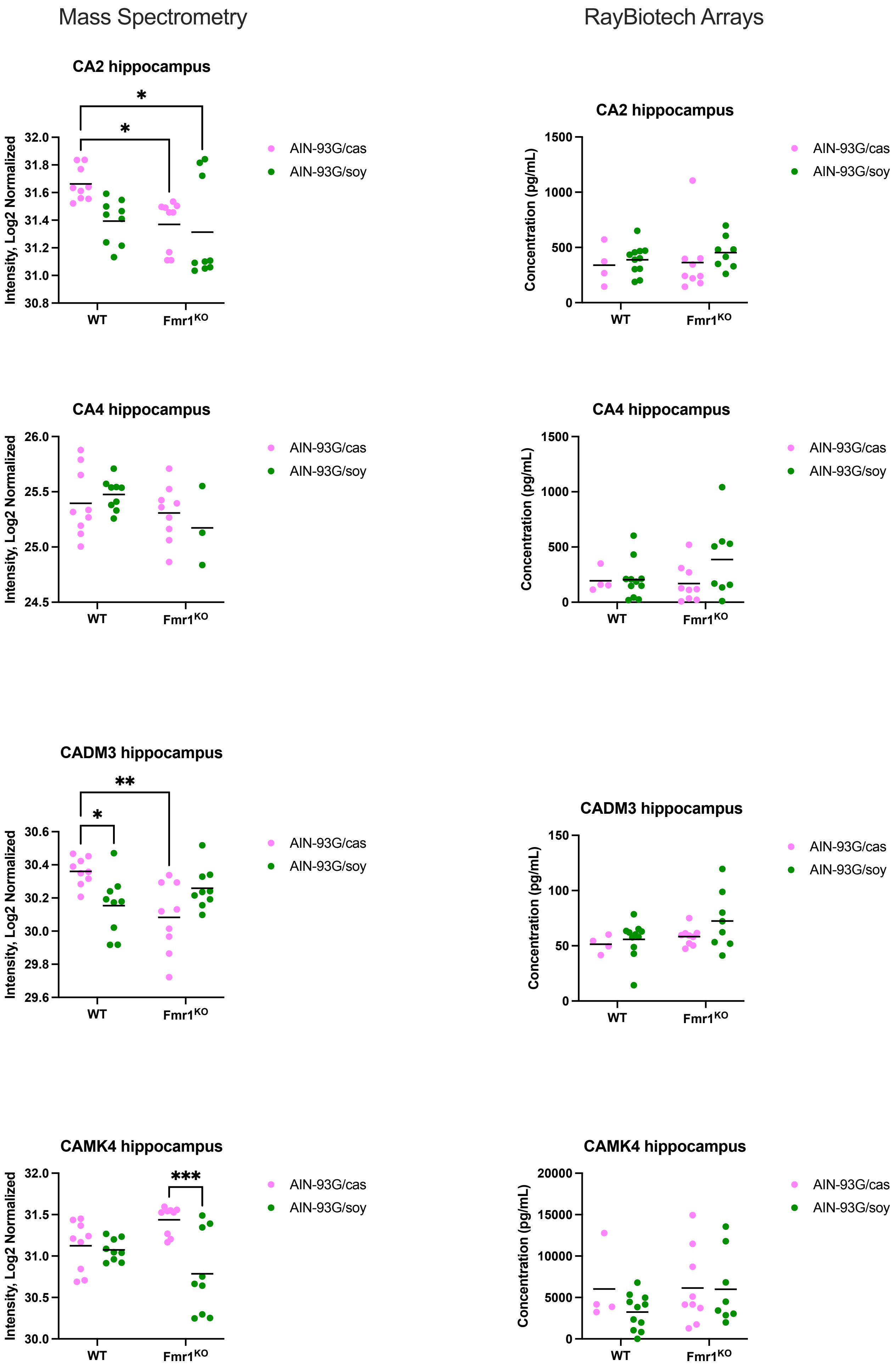

Mass Spectrometry
RayBiotech Arrays
CA2 hippocampus
CA4 hippocampus
CADM3 hippocampus
CAMK4 hippocampus
Intensity, Log2 Normalized
Concentration (pg/mL)
AIN-93G/cas
AIN-93G/soy
WT
Fmr1KO
*
**
***

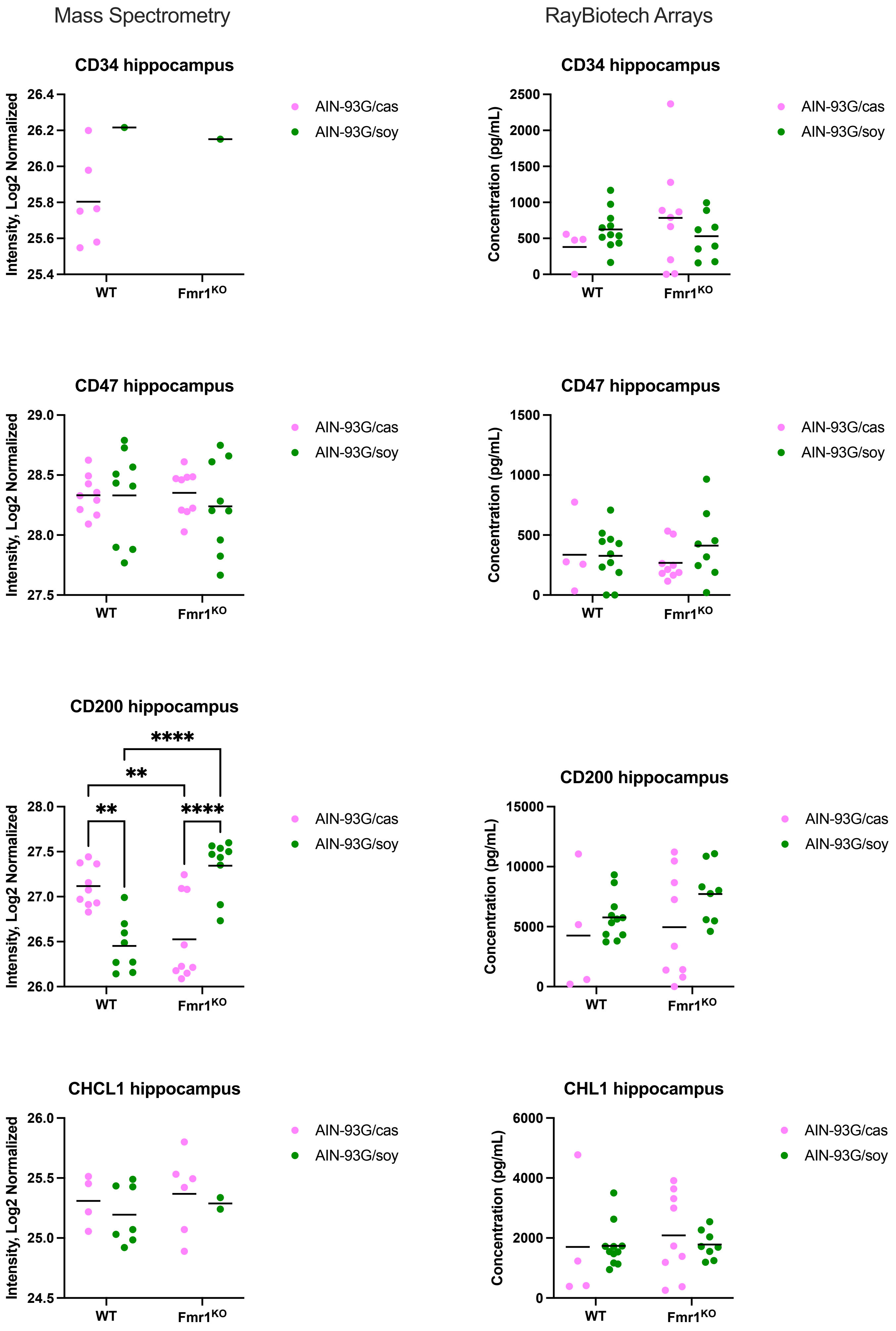
Mass Spectrometry
RayBiotech Arrays
CD34 hippocampus
CD47 hippocampus
CD200 hippocampus
CHCL1 hippocampus
CHL1 hippocampus
Intensity, Log2 Normalized
Concentration (pg/mL)
WT
Fmr1KO
AIN-93G/cas
AIN-93G/soy
**
****
26.4
26.2
26.0
25.8
25.6
25.4
29.0
28.5
28.0
27.5
27.0
26.5
25.5
25.0
24.5
2500
2000
1500
1000
500
0
15000
10000
5000
6000
4000

Mass Spectrometry

RayBiotech Arrays

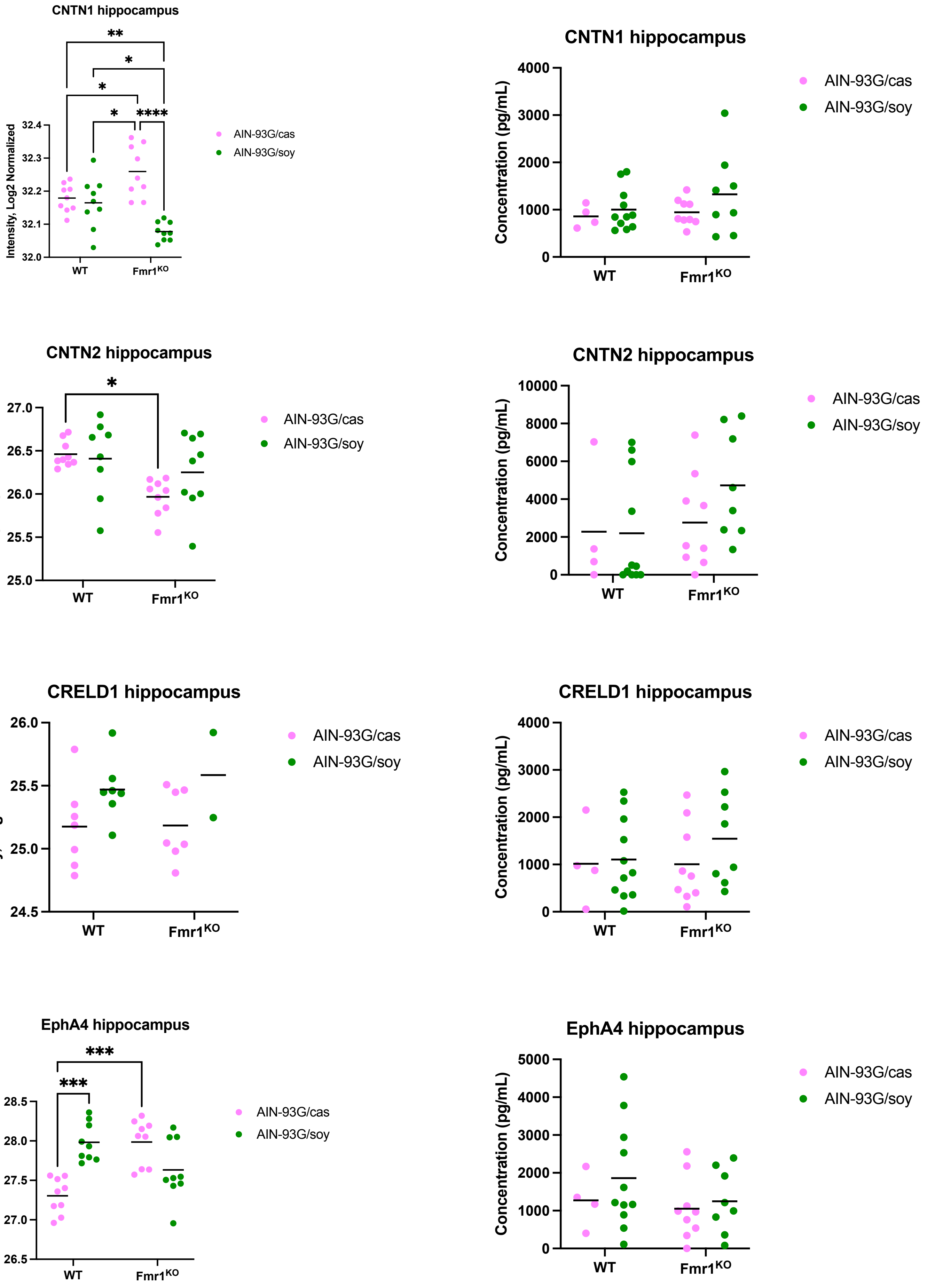

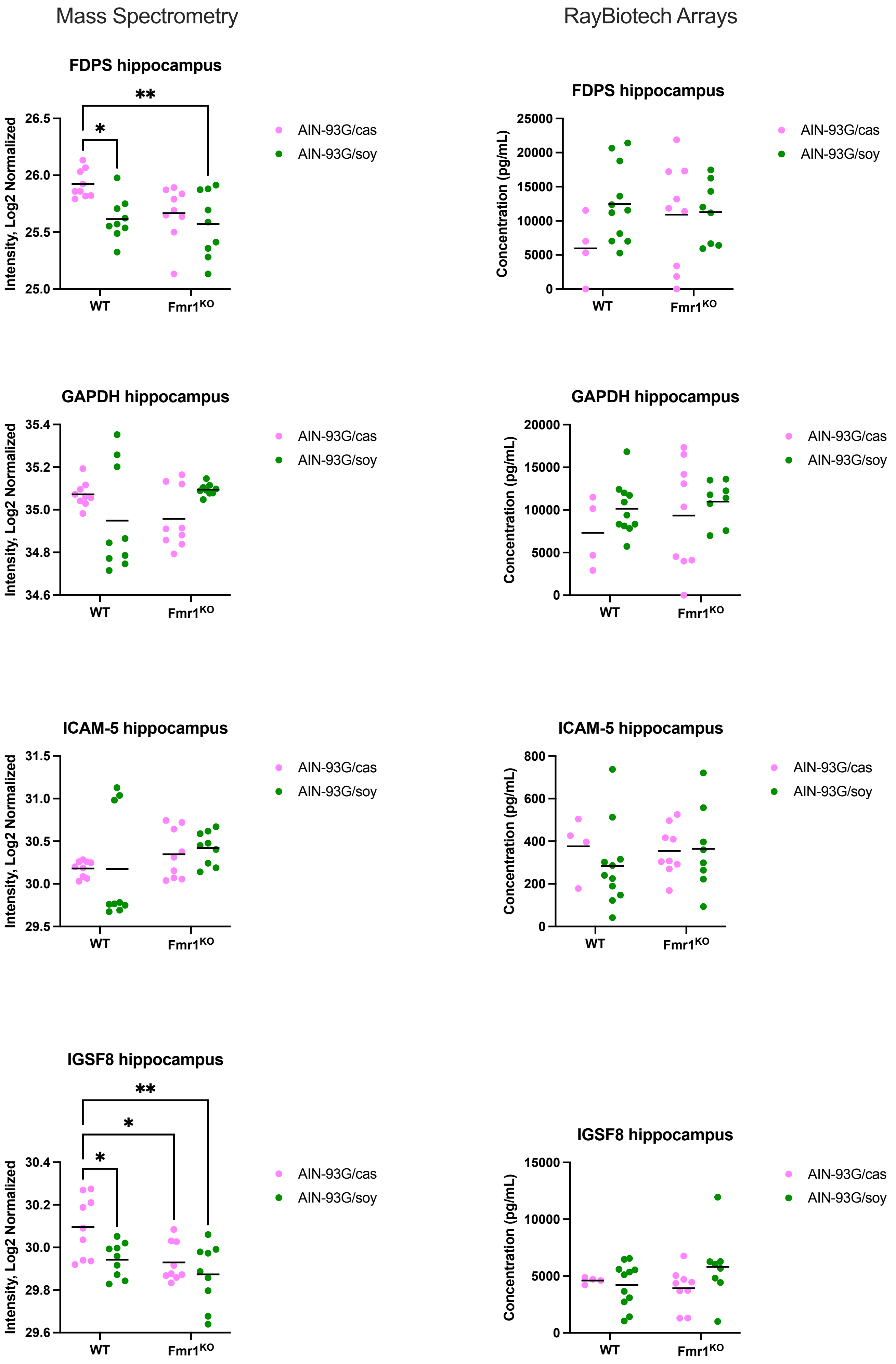
Mass Spectrometry
RayBiotech Arrays
FDPS hippocampus
**
*
Intensity, Log2 Normalized
26.5
26.0
25.5
25.0
WT
Fmr1KO
AIN-93G/cas
AIN-93G/soy
FDPS hippocampus
Concentration (pg/mL)
25000
20000
15000
10000
5000
0
WT
Fmr1KO
AIN-93G/cas
AIN-93G/soy
GAPDH hippocampus
Intensity, Log2 Normalized
35.4
35.2
35.0
34.8
34.6
WT
Fmr1KO
AIN-93G/cas
AIN-93G/soy
GAPDH hippocampus
Concentration (pg/mL)
20000
15000
10000
5000
0
WT
Fmr1KO
AIN-93G/cas
AIN-93G/soy
ICAM-5 hippocampus
Intensity, Log2 Normalized
31.5
31.0
30.5
30.0
29.5
WT
Fmr1KO
AIN-93G/cas
AIN-93G/soy
ICAM-5 hippocampus
Concentration (pg/mL)
800
600
400
200
0
WT
Fmr1KO
AIN-93G/cas
AIN-93G/soy
IGSF8 hippocampus
**
*
*
Intensity, Log2 Normalized
30.4
30.2
30.0
29.8
29.6
WT
Fmr1KO
AIN-93G/cas
AIN-93G/soy
IGSF8 hippocampus
Concentration (pg/mL)
15000
10000
5000
0
WT
Fmr1KO
AIN-93G/cas
AIN-93G/soy

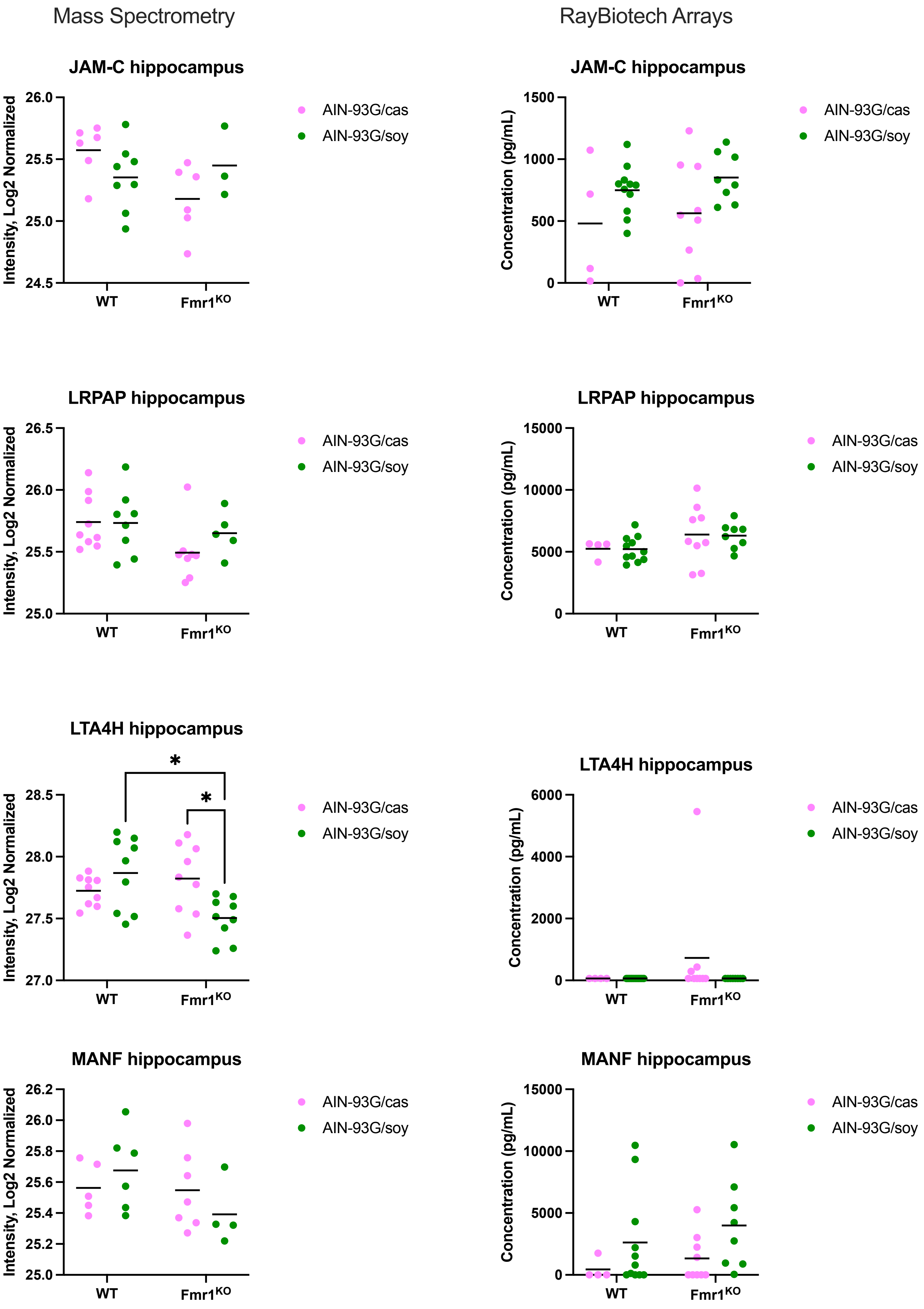

Mass Spectrometry
RayBiotech Arrays
JAM-C hippocampus
Intensity, Log2 Normalized
26.0
25.5
25.0
24.5
WT
Fmr1KO
AIN-93G/cas
AIN-93G/soy
JAM-C hippocampus
Concentration (pg/mL)
1500
1000
500
0
WT
Fmr1KO
AIN-93G/cas
AIN-93G/soy
LRPAP hippocampus
Intensity, Log2 Normalized
26.5
26.0
25.5
25.0
WT
Fmr1KO
AIN-93G/cas
AIN-93G/soy
LRPAP hippocampus
Concentration (pg/mL)
15000
10000
5000
0
WT
Fmr1KO
AIN-93G/cas
AIN-93G/soy
LTA4H hippocampus
*
*
Intensity, Log2 Normalized
28.5
28.0
27.5
27.0
WT
Fmr1KO
AIN-93G/cas
AIN-93G/soy
LTA4H hippocampus
Concentration (pg/mL)
6000
4000
2000
0
WT
Fmr1KO
AIN-93G/cas
AIN-93G/soy
MANF hippocampus
Intensity, Log2 Normalized
26.2
26.0
25.8
25.6
25.4
25.2
25.0
WT
Fmr1KO
AIN-93G/cas
AIN-93G/soy
MANF hippocampus
Concentration (pg/mL)
15000
10000
5000
0
WT
Fmr1KO
AIN-93G/cas
AIN-93G/soy

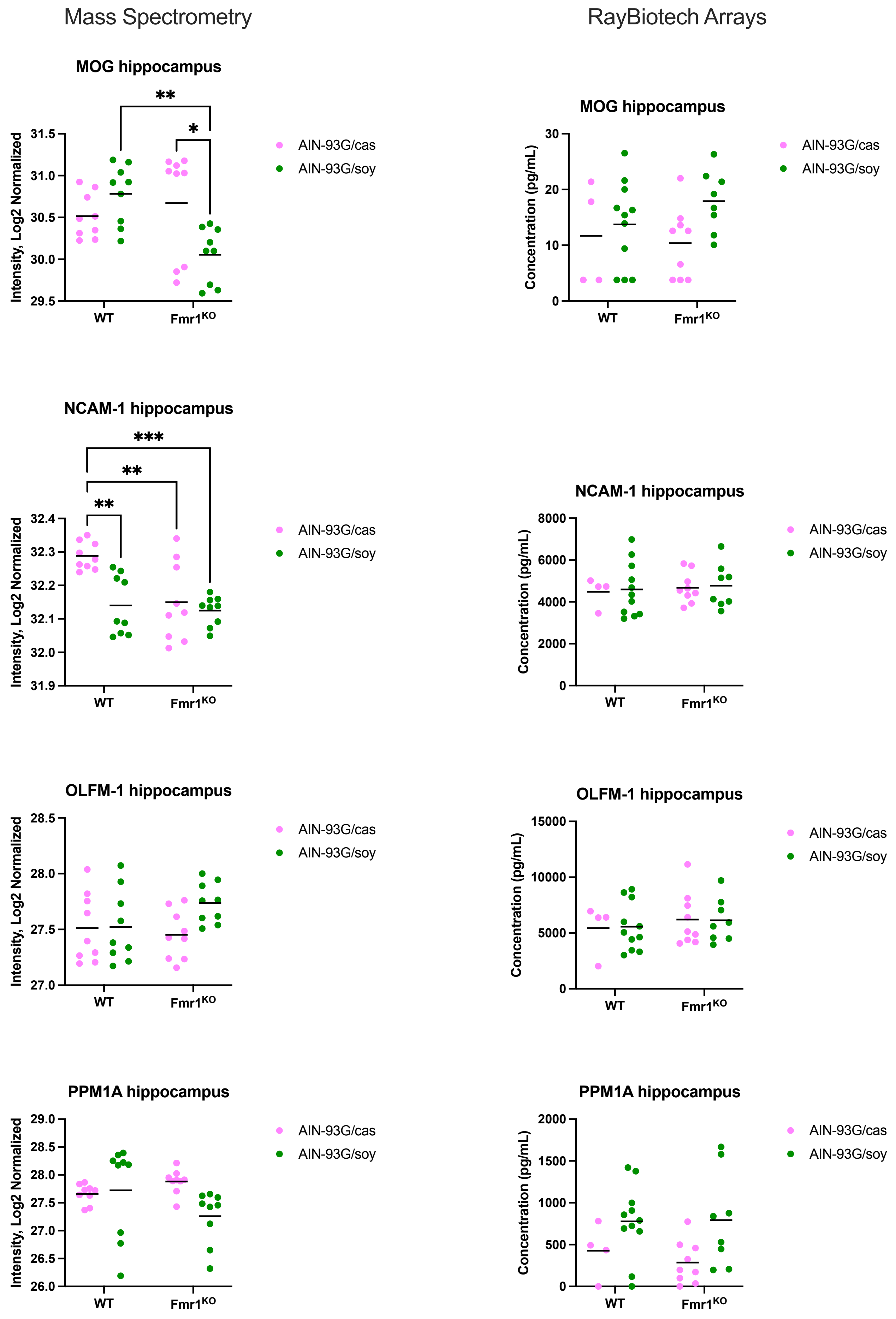
Mass Spectrometry
RayBiotech Arrays
MOG hippocampus
Intensity, Log2 Normalized
31.5
31.0
30.5
30.0
29.5
**
*
WT
Fmr1KO
AIN-93G/cas
AIN-93G/soy
MOG hippocampus
Concentration (pg/mL)
30
20
10
0
WT
Fmr1KO
AIN-93G/cas
AIN-93G/soy
NCAM-1 hippocampus
Intensity, Log2 Normalized
32.4
32.3
32.2
32.1
32.0
31.9
***
**
**
WT
Fmr1KO
AIN-93G/cas
AIN-93G/soy
NCAM-1 hippocampus
Concentration (pg/mL)
8000
6000
4000
2000
0
WT
Fmr1KO
AIN-93G/cas
AIN-93G/soy
OLFM-1 hippocampus
Intensity, Log2 Normalized
28.5
28.0
27.5
27.0
WT
Fmr1KO
AIN-93G/cas
AIN-93G/soy
OLFM-1 hippocampus
Concentration (pg/mL)
15000
10000
5000
0
WT
Fmr1KO
AIN-93G/cas
AIN-93G/soy
PPM1A hippocampus
Intensity, Log2 Normalized
29.0
28.5
28.0
27.5
27.0
26.5
26.0
WT
Fmr1KO
AIN-93G/cas
AIN-93G/soy
PPM1A hippocampus
Concentration (pg/mL)
2000
1500
1000
500
0
WT
Fmr1KO
AIN-93G/cas
AIN-93G/soy

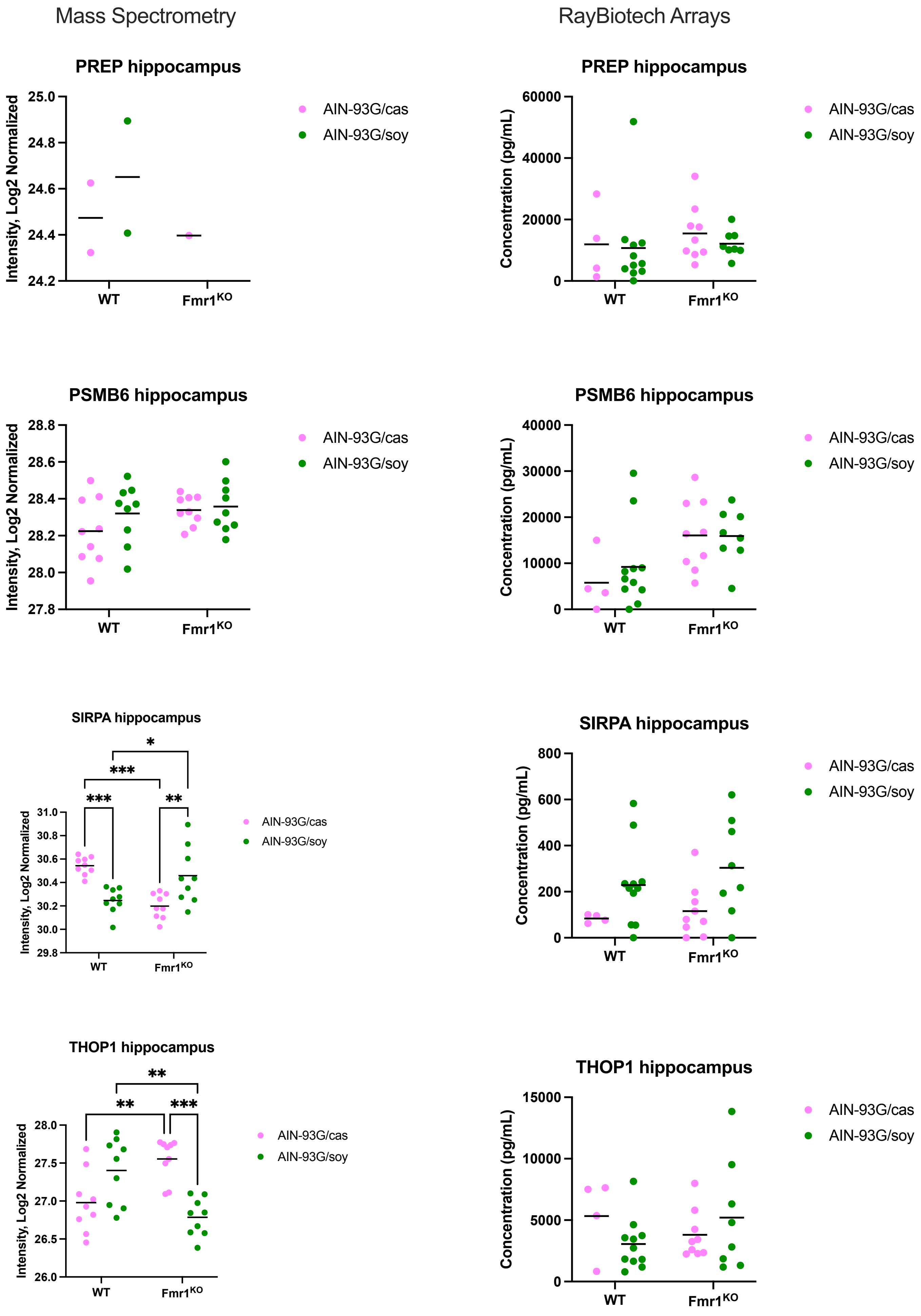

Mass Spectrometry
RayBiotech Arrays
PREP hippocampus
Intensity, Log2 Normalized
25.0
24.8
24.6
24.4
24.2
WT
Fmr1KO
AIN-93G/cas
AIN-93G/soy
PREP hippocampus
Concentration (pg/mL)
60000
40000
20000
0
WT
Fmr1KO
AIN-93G/cas
AIN-93G/soy
PSMB6 hippocampus
Intensity, Log2 Normalized
28.8
28.6
28.4
28.2
28.0
27.8
WT
Fmr1KO
AIN-93G/cas
AIN-93G/soy
PSMB6 hippocampus
Concentration (pg/mL)
40000
30000
20000
10000
0
WT
Fmr1KO
AIN-93G/cas
AIN-93G/soy
SIRPA hippocampus
*
***
***
**
Intensity, Log2 Normalized
31.0
30.8
30.6
30.4
30.2
30.0
29.8
WT
Fmr1KO
AIN-93G/cas
AIN-93G/soy
SIRPA hippocampus
Concentration (pg/mL)
800
600
400
200
0
WT
Fmr1KO
AIN-93G/cas
AIN-93G/soy
THOP1 hippocampus
**
**
***
Intensity, Log2 Normalized
28.0
27.5
27.0
26.5
26.0
WT
Fmr1KO
AIN-93G/cas
AIN-93G/soy
THOP1 hippocampus
Concentration (pg/mL)
15000
10000
5000
0
WT
Fmr1KO
AIN-93G/cas
AIN-93G/soy

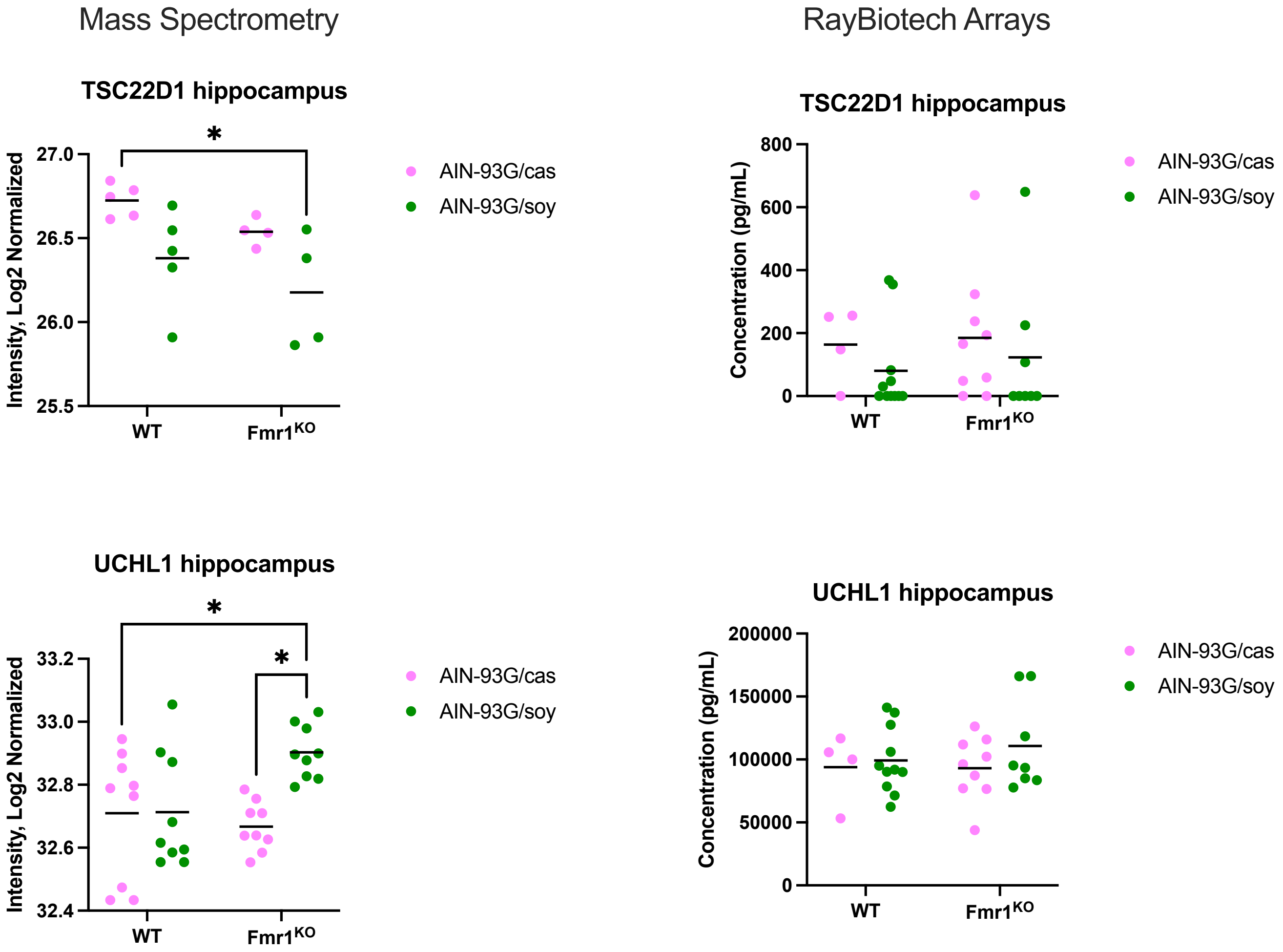
Mass Spectrometry
RayBiotech Arrays
TSC22D1 hippocampus
Intensity, Log2 Normalized
27.0
26.5
26.0
25.5
*
WT
Fmr1KO
AIN-93G/cas
AIN-93G/soy
TSC22D1 hippocampus
Concentration (pg/mL)
800
600
400
200
0
WT
Fmr1KO
AIN-93G/cas
AIN-93G/soy
UCHL1 hippocampus
Intensity, Log2 Normalized
33.2
33.0
32.8
32.6
32.4
*
*
WT
Fmr1KO
AIN-93G/cas
AIN-93G/soy
UCHL1 hippocampus
Concentration (pg/mL)
200000
150000
100000
50000
0
WT
Fmr1KO
AIN-93G/cas
AIN-93G/soy
