# Supplementary material for: Soy Protein Isolate Affects Blood and Brain Biomarker Expression in a Mouse Model of Fragile X
Source: Int J Mol Sci. 2025 Jun 26;26(13):6137. doi: 10.3390/ijms26136137 (PMC12250412; doi:10.3390/ijms26136137)
Supplement: Supplementary file 1 [file ijms-26-06137-s001.zip › Supplementary File S2b Array 4 Graphs.pdf]

Supplementary File S2. Protein expression of Array 4 targets as function of *Fmr1* genotype and AIN-93G diets. Mice on AIN-93G/cas (colored pink) included n=5 $Fmr1^{HET}$ female, n=8 $Fmr1^{KO}$ female, n=4 WT male and n=9 $Fmr1^{KO}$ male. Mice on AIN-93G/soy (colored green) included n=9 $Fmr1^{HET}$ female, n=8 $Fmr1^{KO}$ female, n=11 WT male and n=8 $Fmr1^{KO}$ male. The average concentration in cortex, hippocampus, hypothalamus and plasma in pg/mL was plotted versus genotype. Statistics were determined by 2-way ANOVA and Tukey's multiple comparison tests denoted by $p < 0.05$ (*), $p < 0.01$ (**), $p < 0.001$ (***) and $p < 0.0001$ (****).

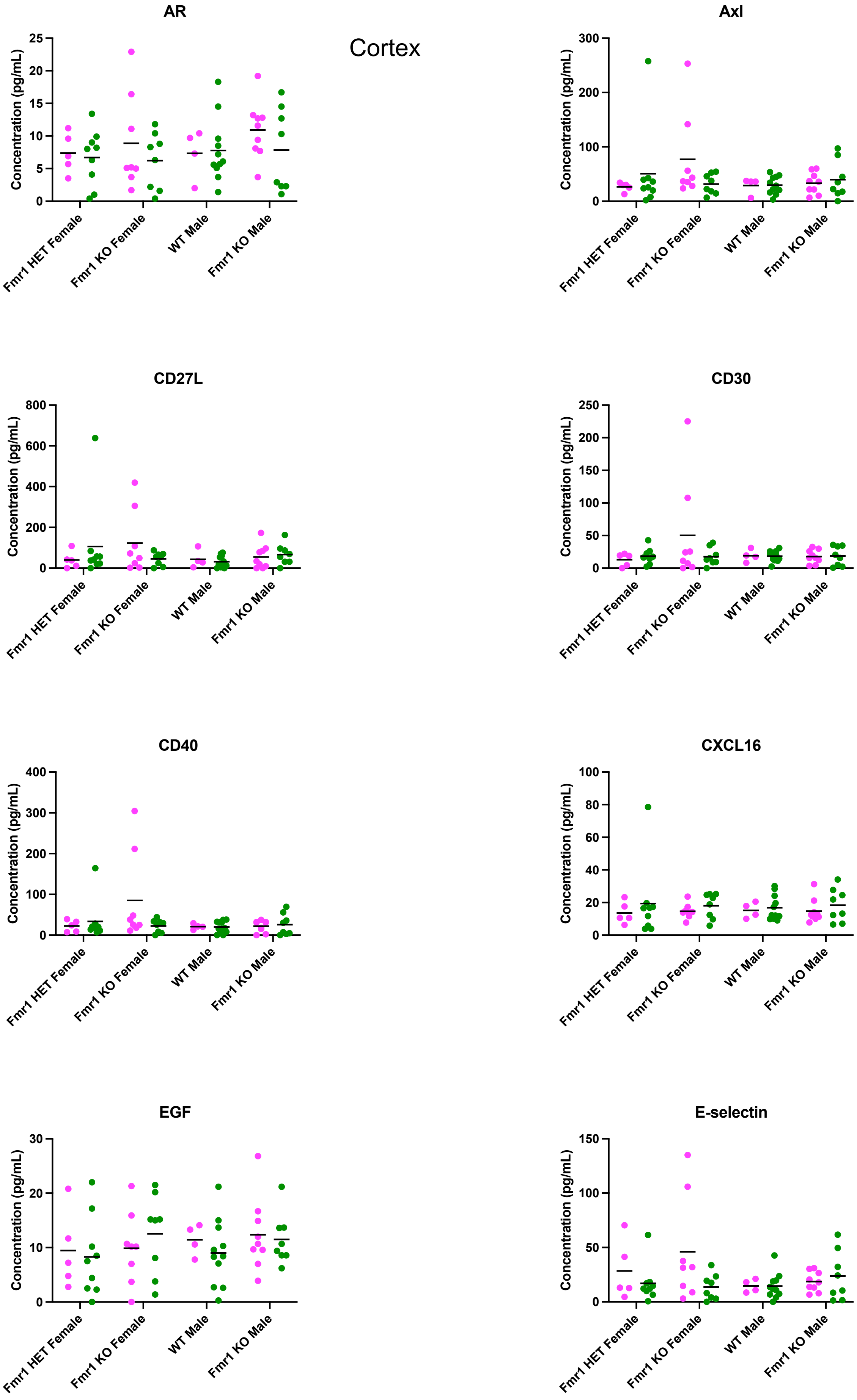
Cortex
AR
Axl
CD27L
CD30
CD40
CXCL16
EGF
E-selectin
Concentration (pg/mL)
Fmr1 HET Female
Fmr1 KO Female
WT Male
Fmr1 KO Male
0
5
10
15
20
25
30
40
50
60
80
100
150
200
250
300
400
600
800

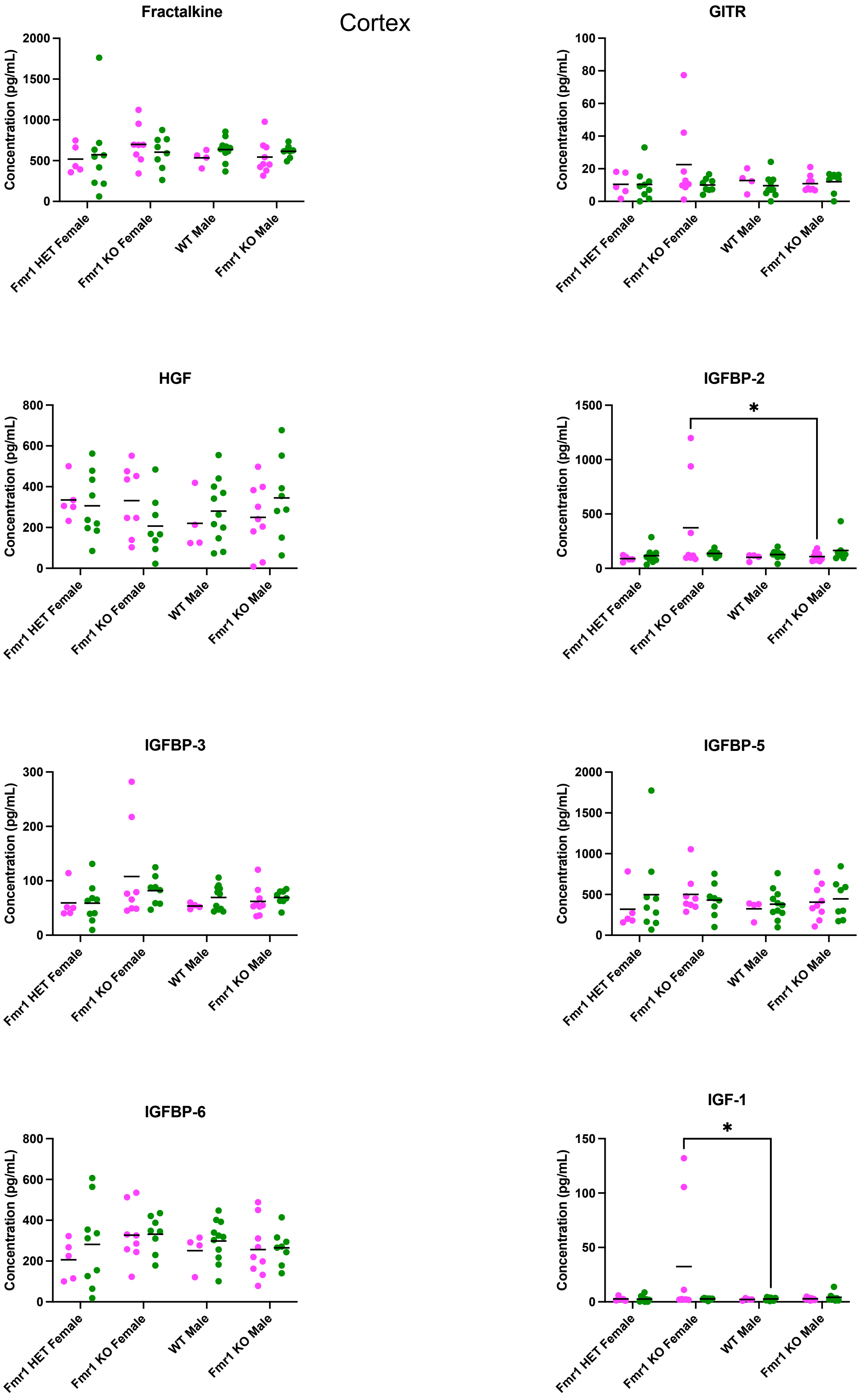
Cortex
Fractalkine
GITR
HGF
IGFBP-2
IGFBP-3
IGFBP-5
IGFBP-6
IGF-1
Concentration (pg/mL)
Fmr1 HET Female
Fmr1 KO Female
WT Male
Fmr1 KO Male
*

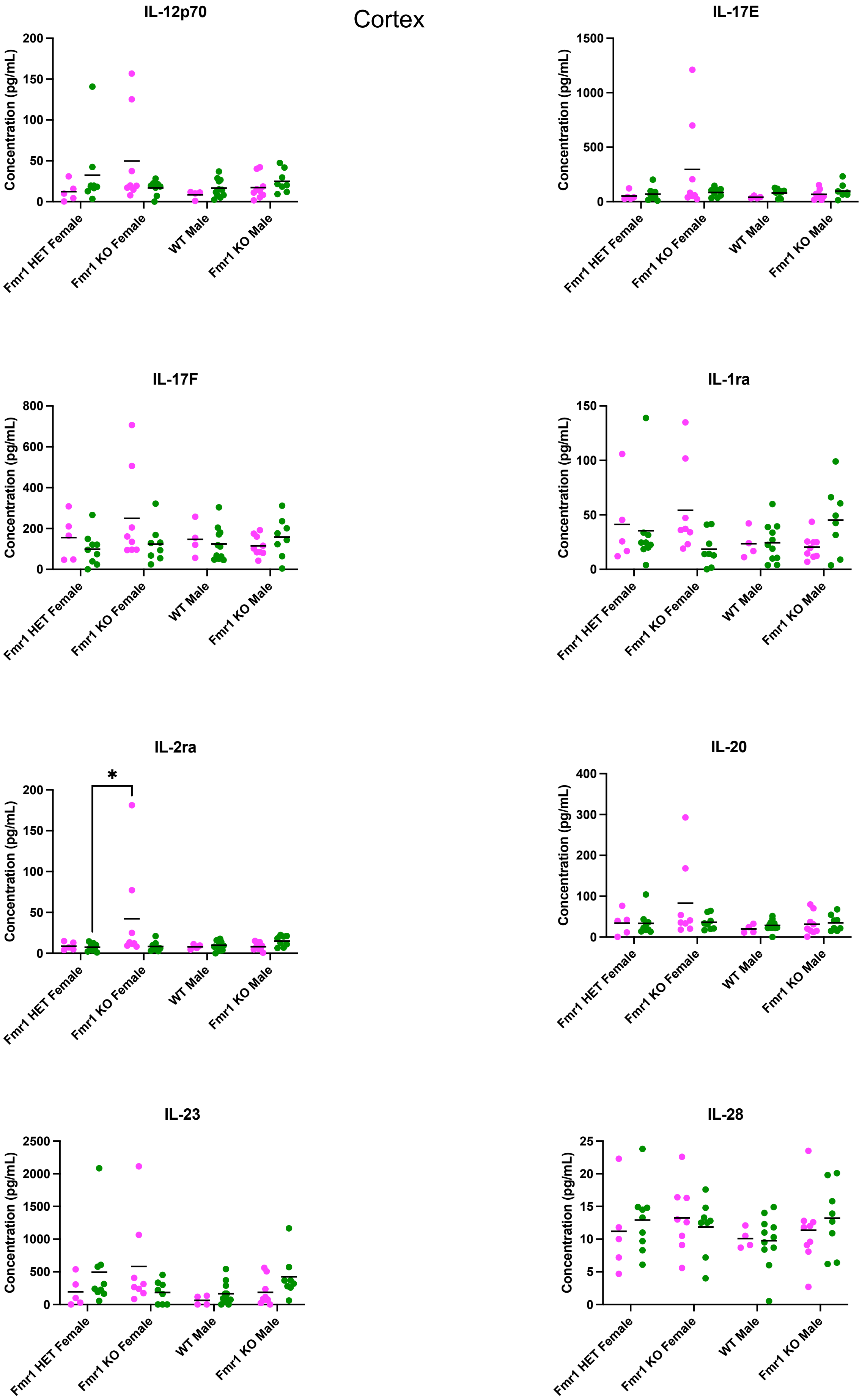
Cortex
IL-12p70
IL-17E
IL-17F
IL-1ra
IL-2ra
*
IL-20
IL-23
IL-28
Concentration (pg/mL)
Fmr1 HET Female
Fmr1 KO Female
WT Male
Fmr1 KO Male
0
50
100
150
200
500
1000
1500
400
600
800
300
2000
2500
5
10
15
20
25

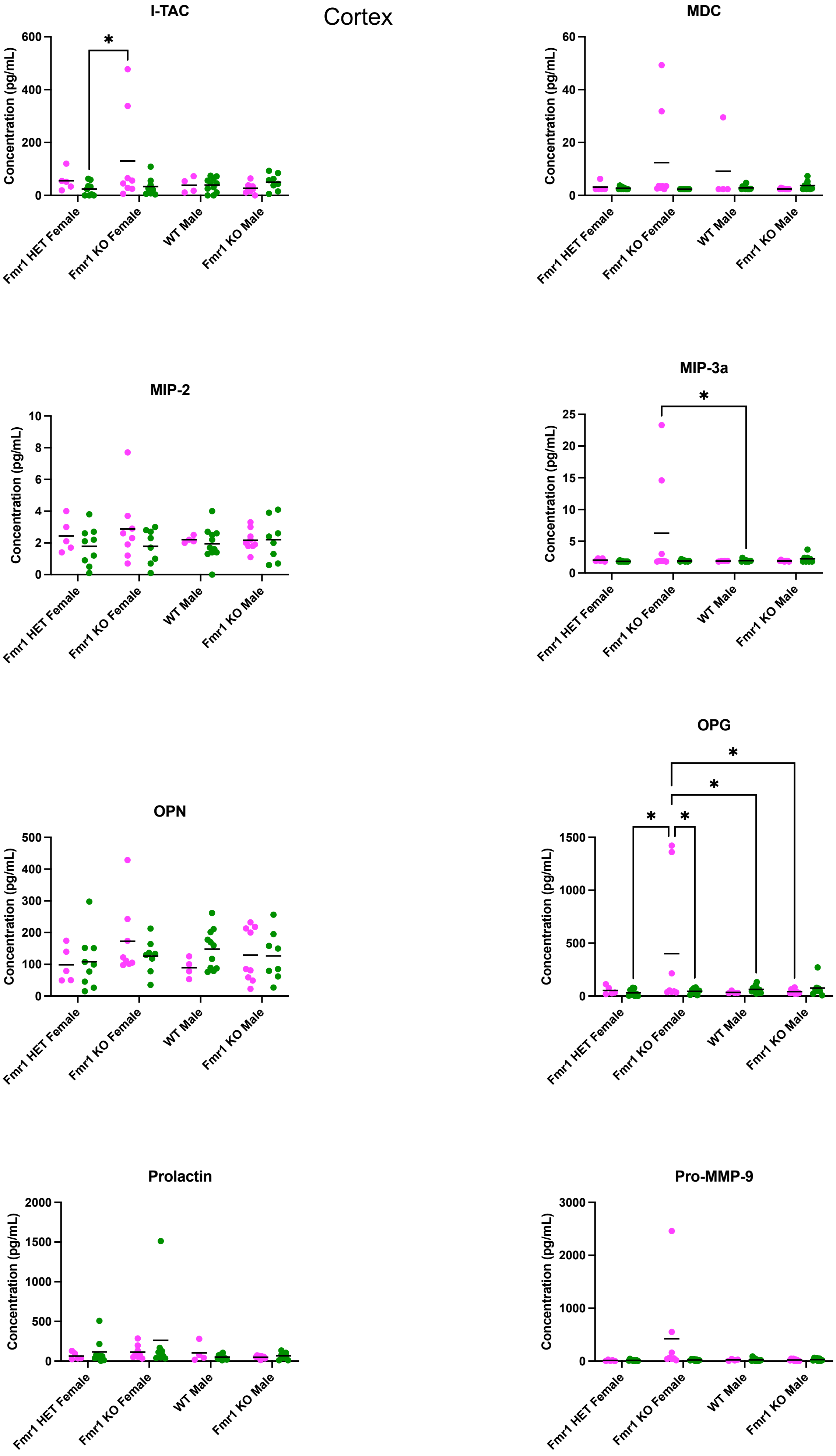
Cortex
I-TAC
MDC
MIP-2
MIP-3a
OPN
OPG
Prolactin
Pro-MMP-9
Concentration (pg/mL)
Fmr1 HET Female
Fmr1 KO Female
WT Male
Fmr1 KO Male
*

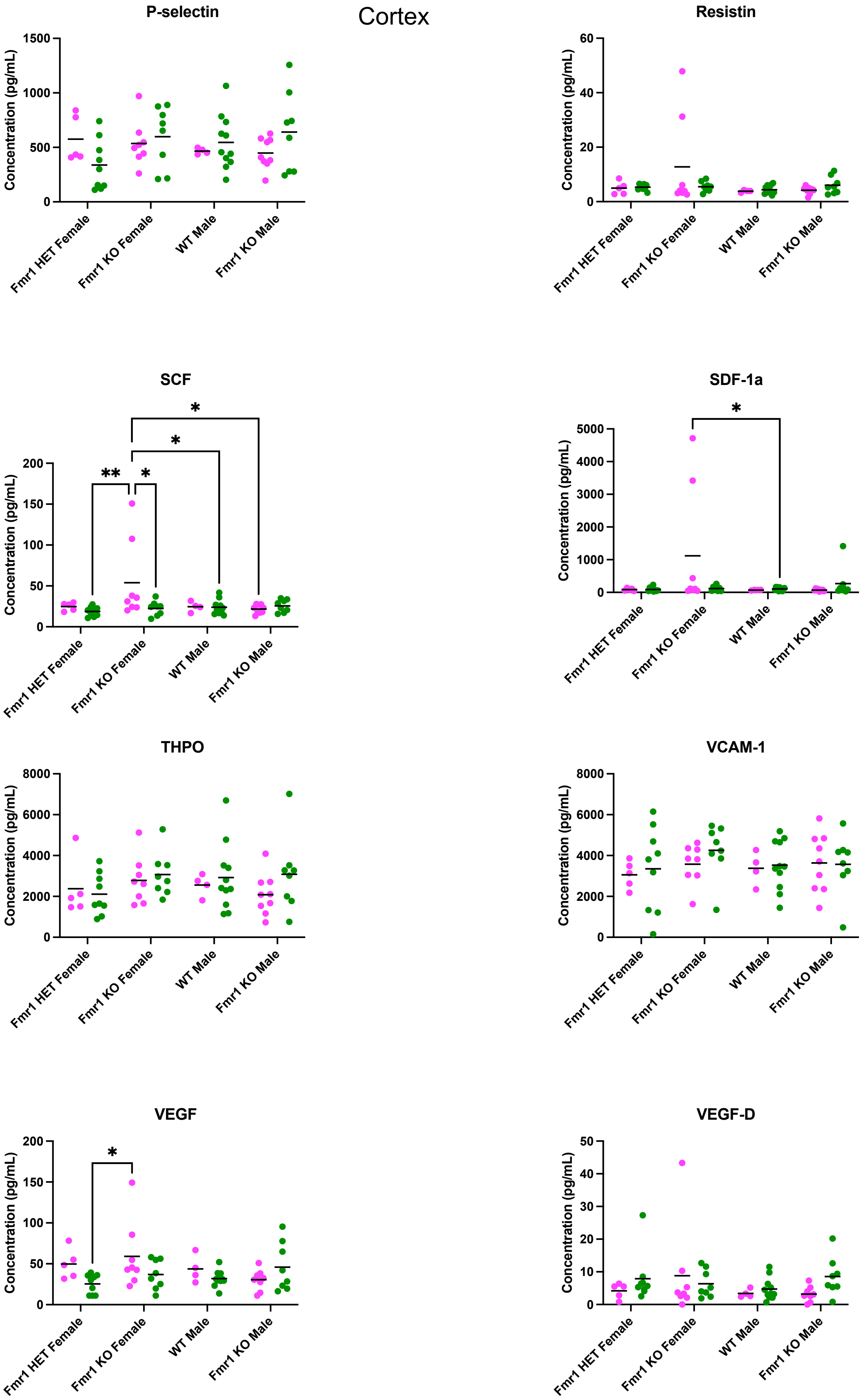
Cortex
P-selectin
Resistin
SCF
SDF-1a
THPO
VCAM-1
VEGF
VEGF-D
Concentration (pg/mL)
Fmr1 HET Female
Fmr1 KO Female
WT Male
Fmr1 KO Male
**
*

Hippocampus

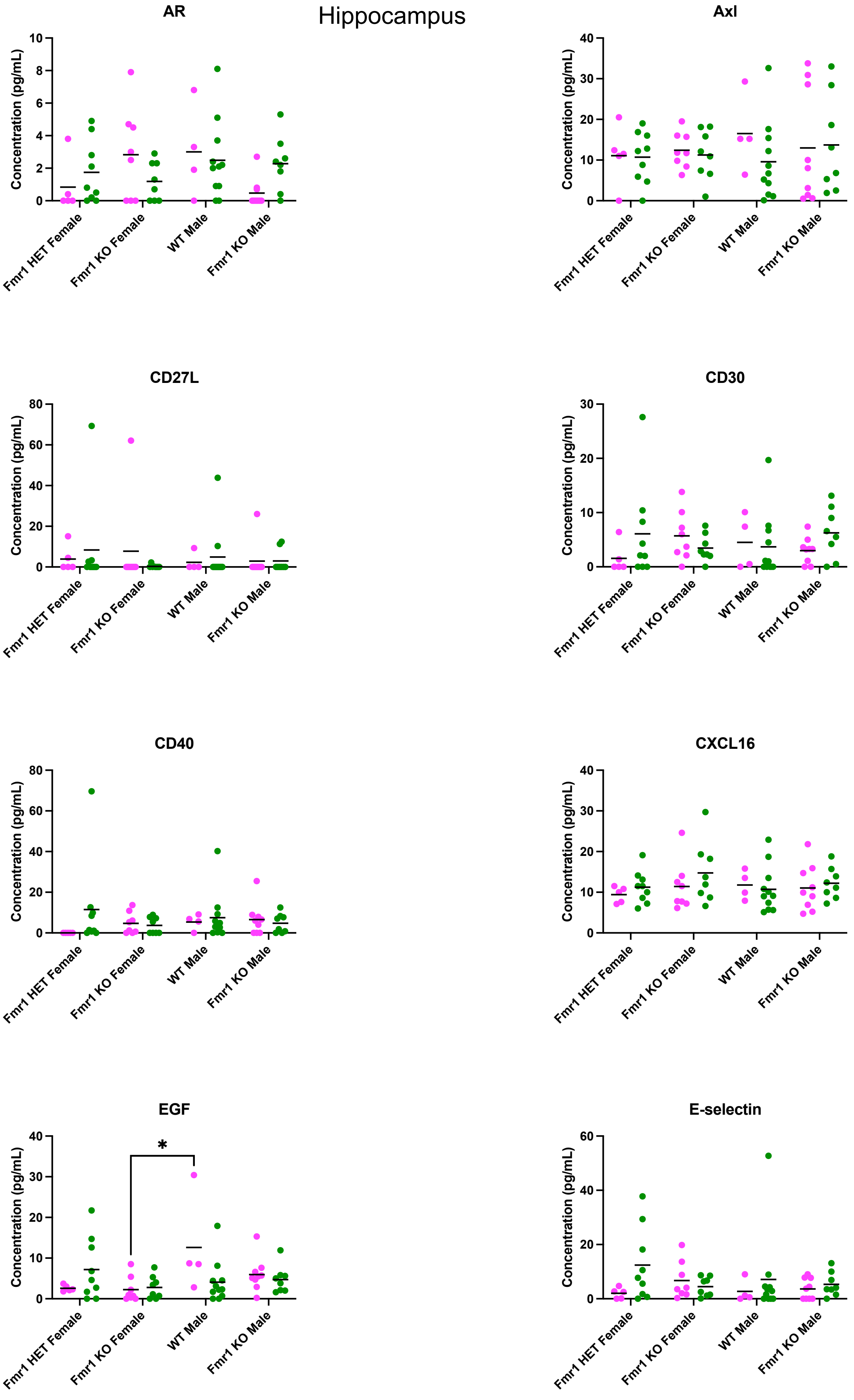

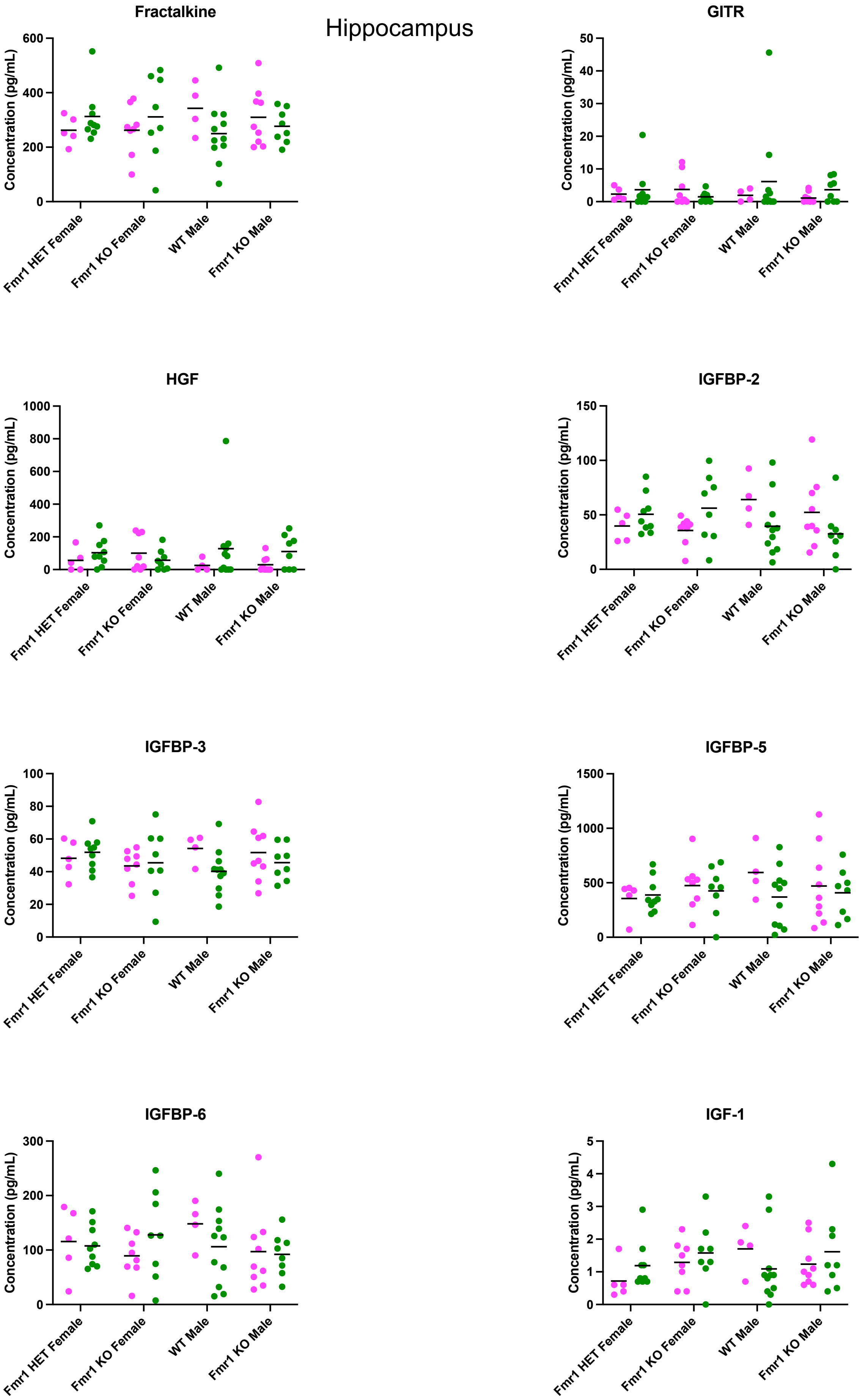
Hippocampus
Fractalkine
GITR
HGF
IGFBP-2
IGFBP-3
IGFBP-5
IGFBP-6
IGF-1
Concentration (pg/mL)
Fmr1 HET Female
Fmr1 KO Female
WT Male
Fmr1 KO Male

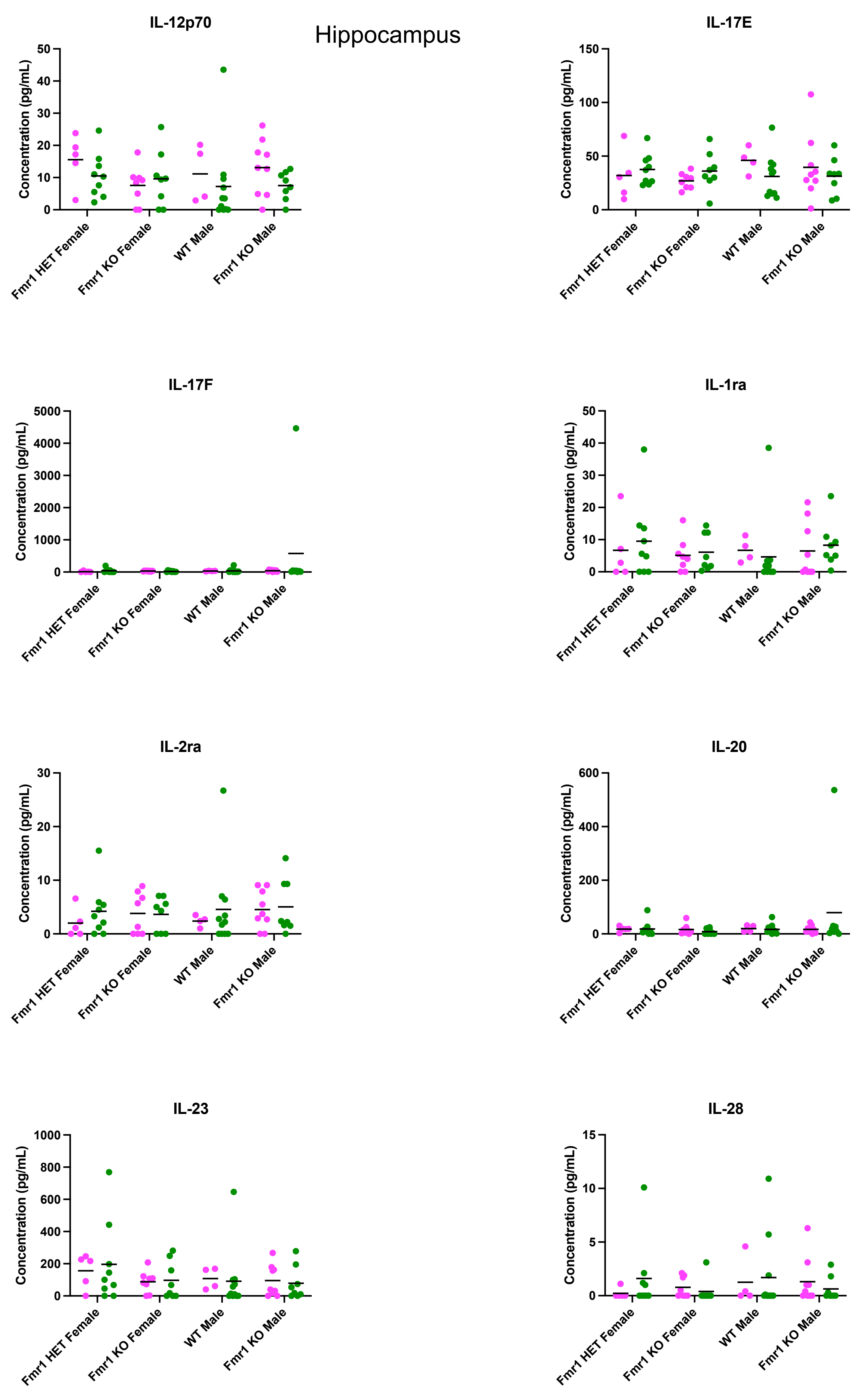

Hippocampus
IL-12p70
IL-17E
IL-17F
IL-1ra
IL-2ra
IL-20
IL-23
IL-28
Concentration (pg/mL)
0
10
20
30
40
50
100
150
1000
2000
3000
4000
5000
200
400
600
800
5
15
Fmr1 HET Female
Fmr1 KO Female
WT Male
Fmr1 KO Male

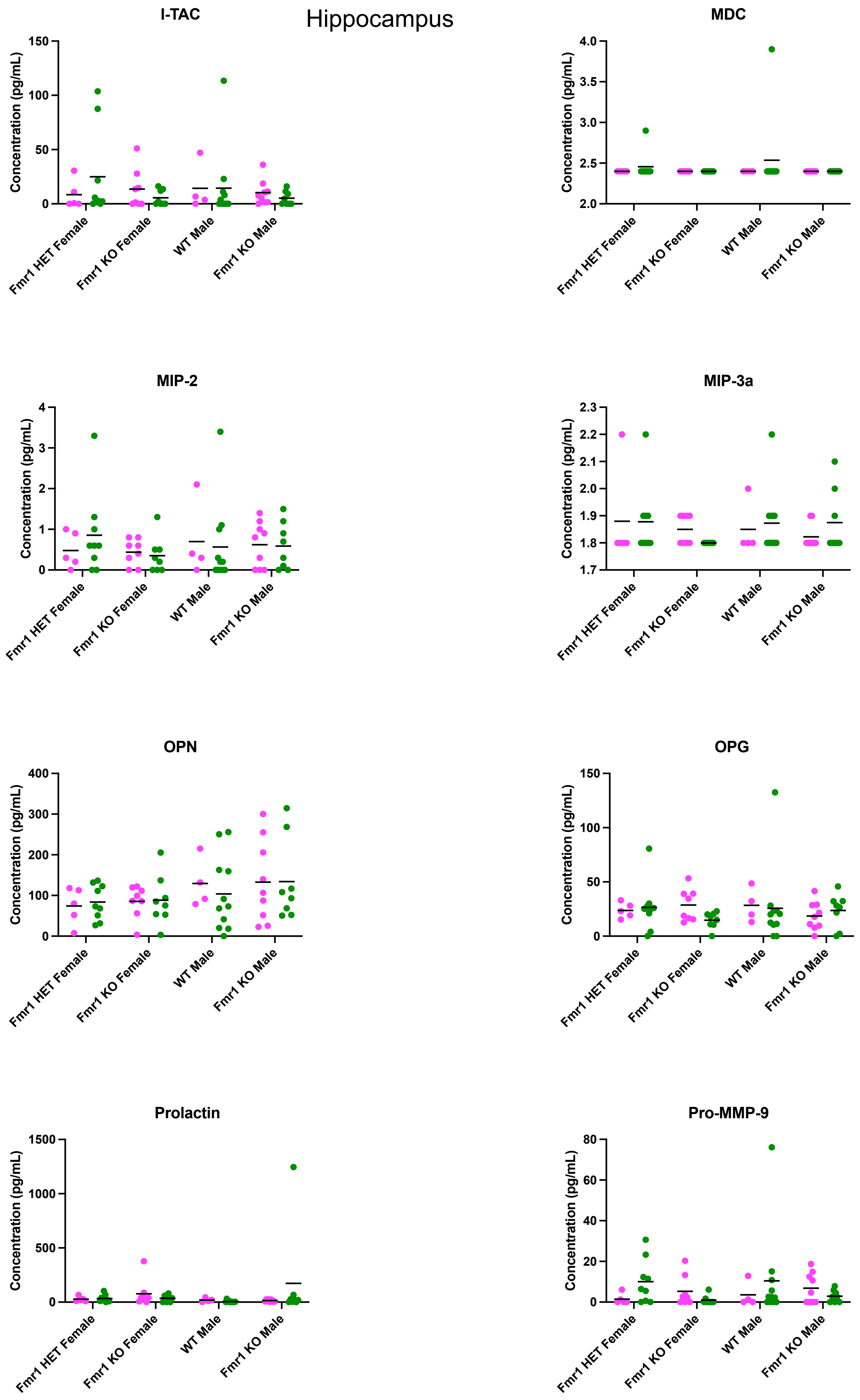
Hippocampus
I-TAC
MDC
MIP-2
MIP-3a
OPN
OPG
Prolactin
Pro-MMP-9
Concentration (pg/mL)
Fmr1 HET Female
Fmr1 KO Female
WT Male
Fmr1 KO Male

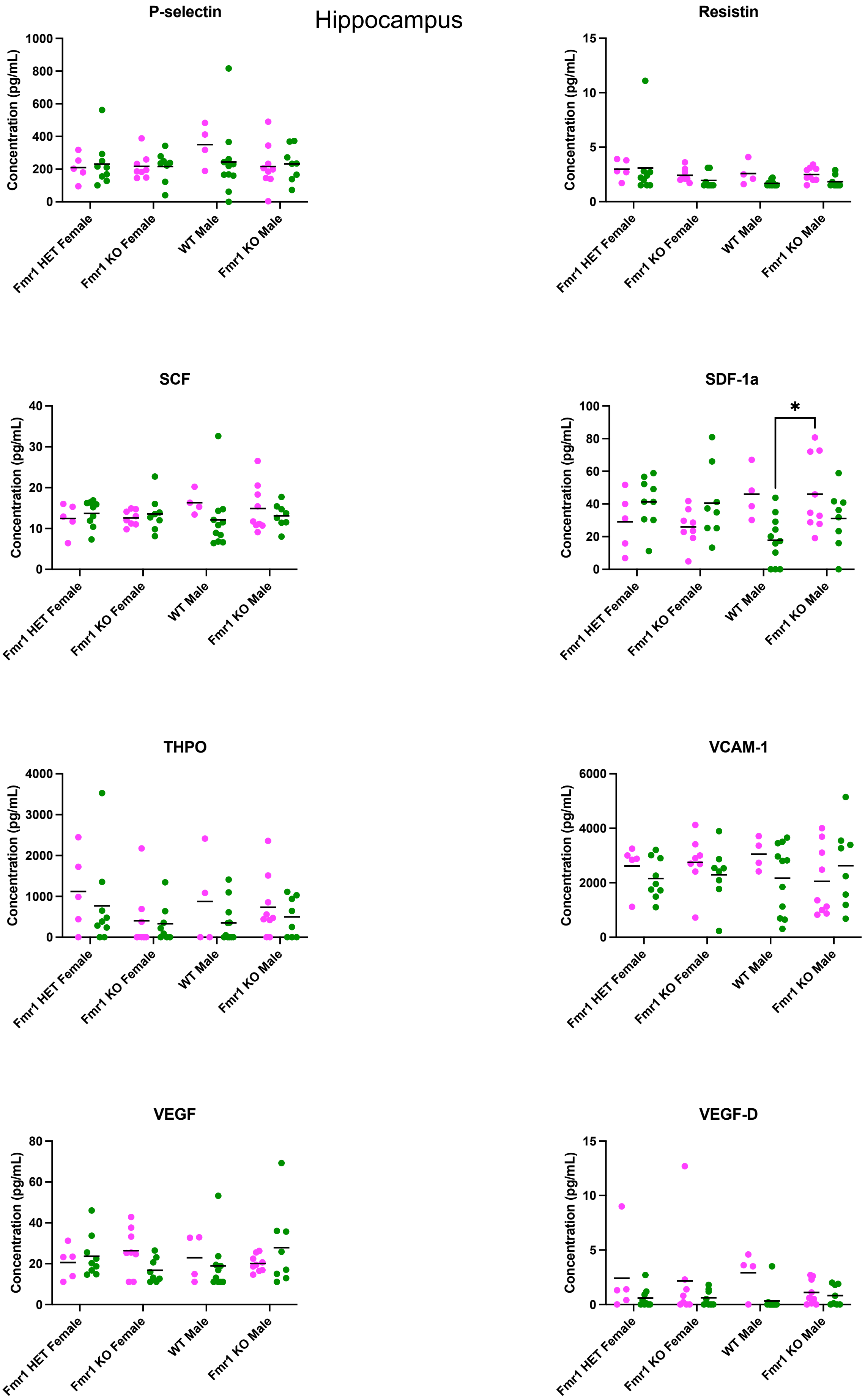

Hippocampus
P-selectin
Resistin
SCF
SDF-1a
*
THPO
VCAM-1
VEGF
VEGF-D
Concentration (pg/mL)
Fmr1 HET Female
Fmr1 KO Female
WT Male
Fmr1 KO Male
0
200
400
600
800
1000
5
10
15
20
30
40
60
80
100
1000
2000
3000
4000
6000

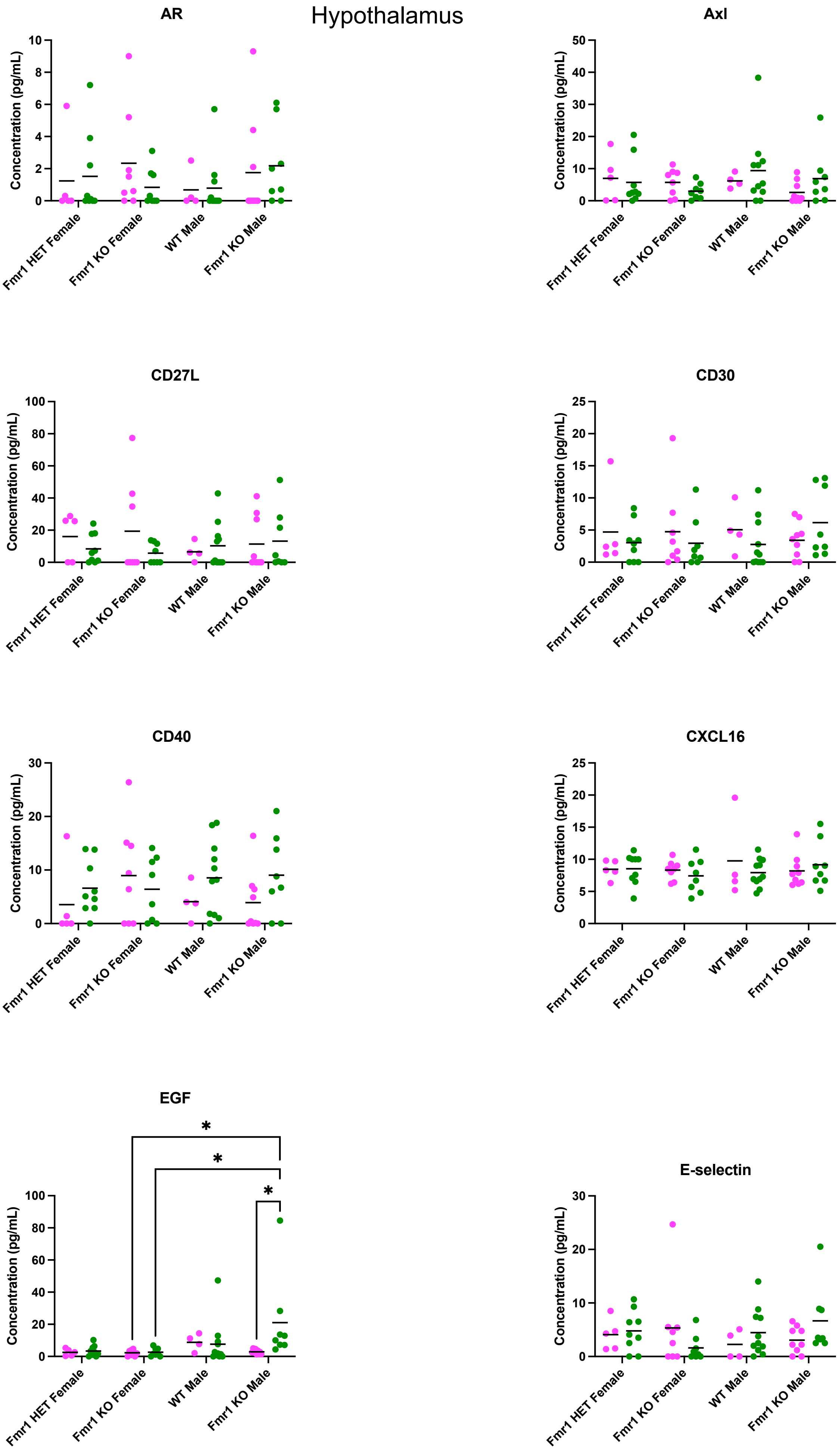
Hypothalamus
AR
Axl
CD27L
CD30
CD40
CXCL16
EGF
E-selectin
Concentration (pg/mL)
Fmr1 HET Female
Fmr1 KO Female
WT Male
Fmr1 KO Male
*
*
*

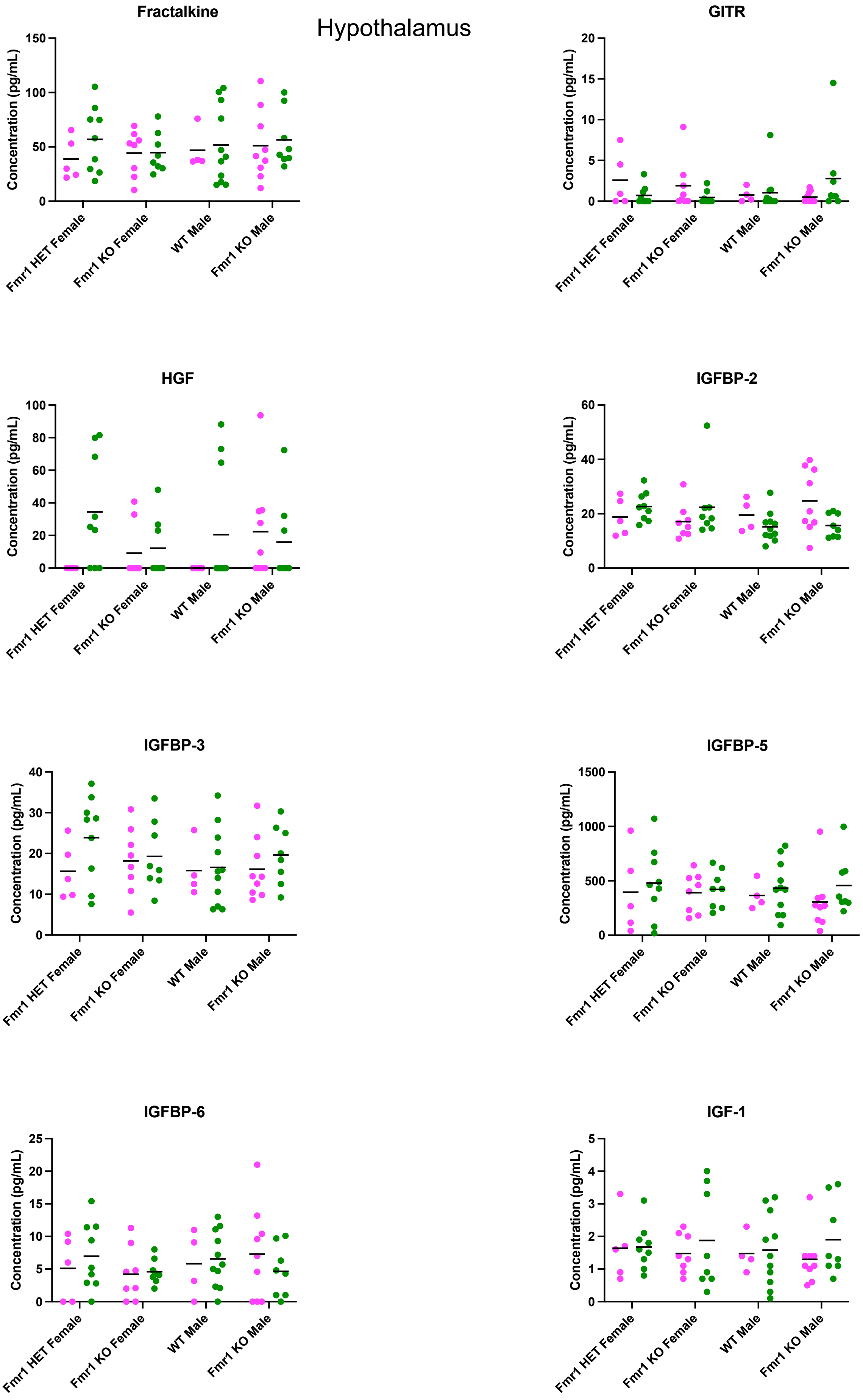
Hypothalamus
Fractalkine
GITR
HGF
IGFBP-2
IGFBP-3
IGFBP-5
IGFBP-6
IGF-1
Concentration (pg/mL)
Fmr1 HET Female
Fmr1 KO Female
WT Male
Fmr1 KO Male
0
50
100
150
5
10
15
20
20
40
60
80
25
30
500
1000
1500
1
2
3
4

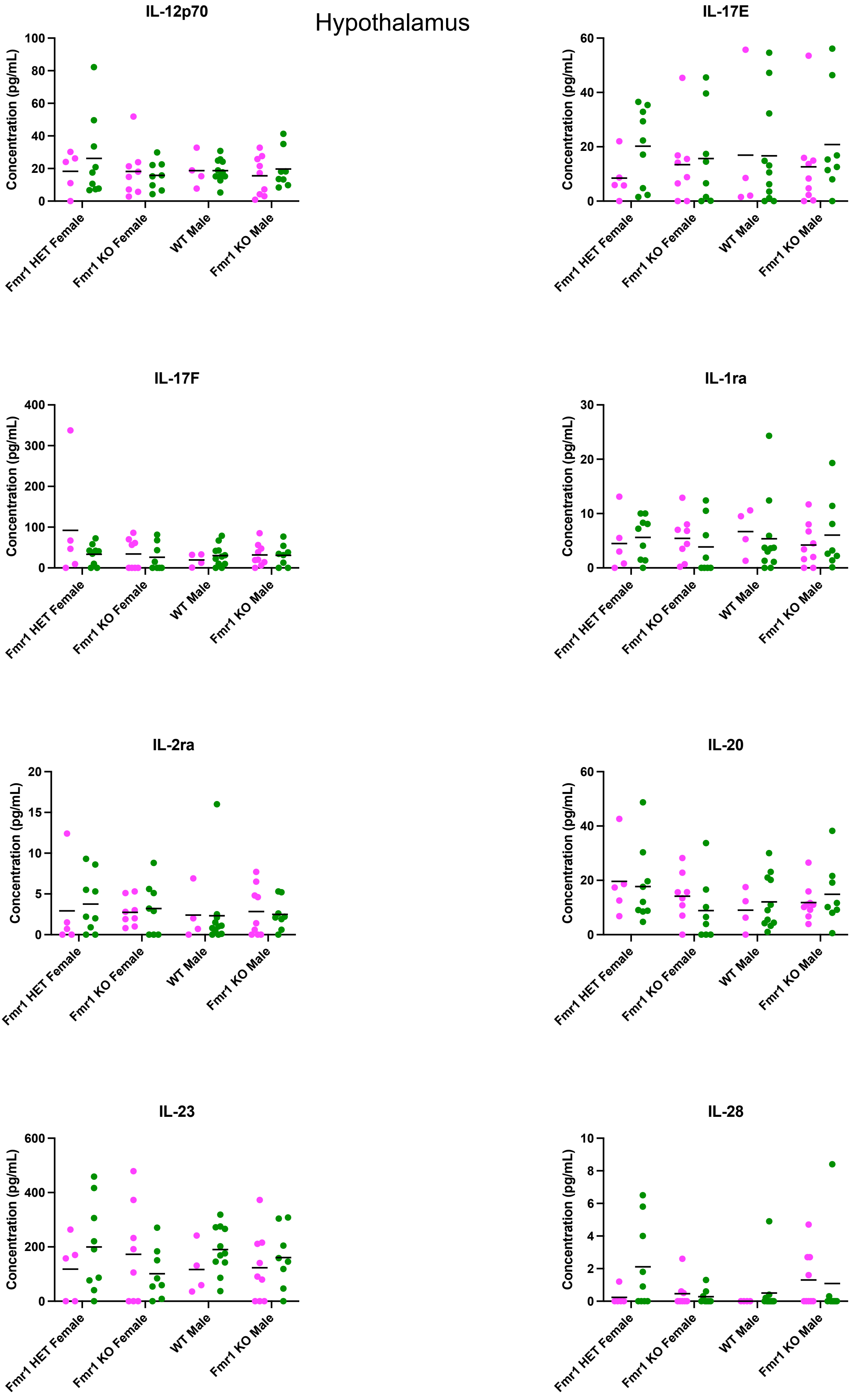
Hypothalamus
IL-12p70
IL-17E
IL-17F
IL-1ra
IL-2ra
IL-20
IL-23
IL-28
Concentration (pg/mL)
Fmr1 HET Female
Fmr1 KO Female
WT Male
Fmr1 KO Male

Hypothalamus

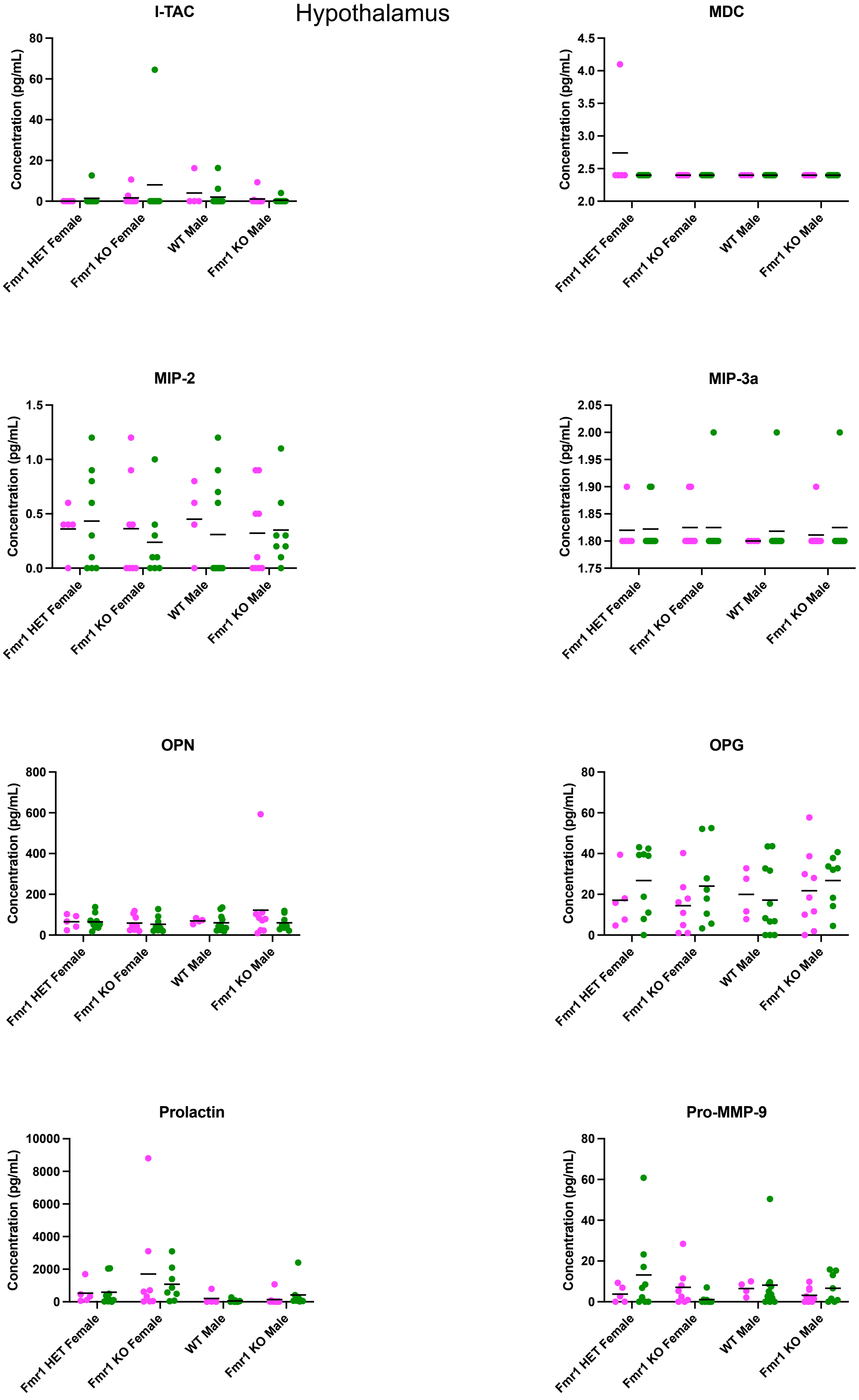

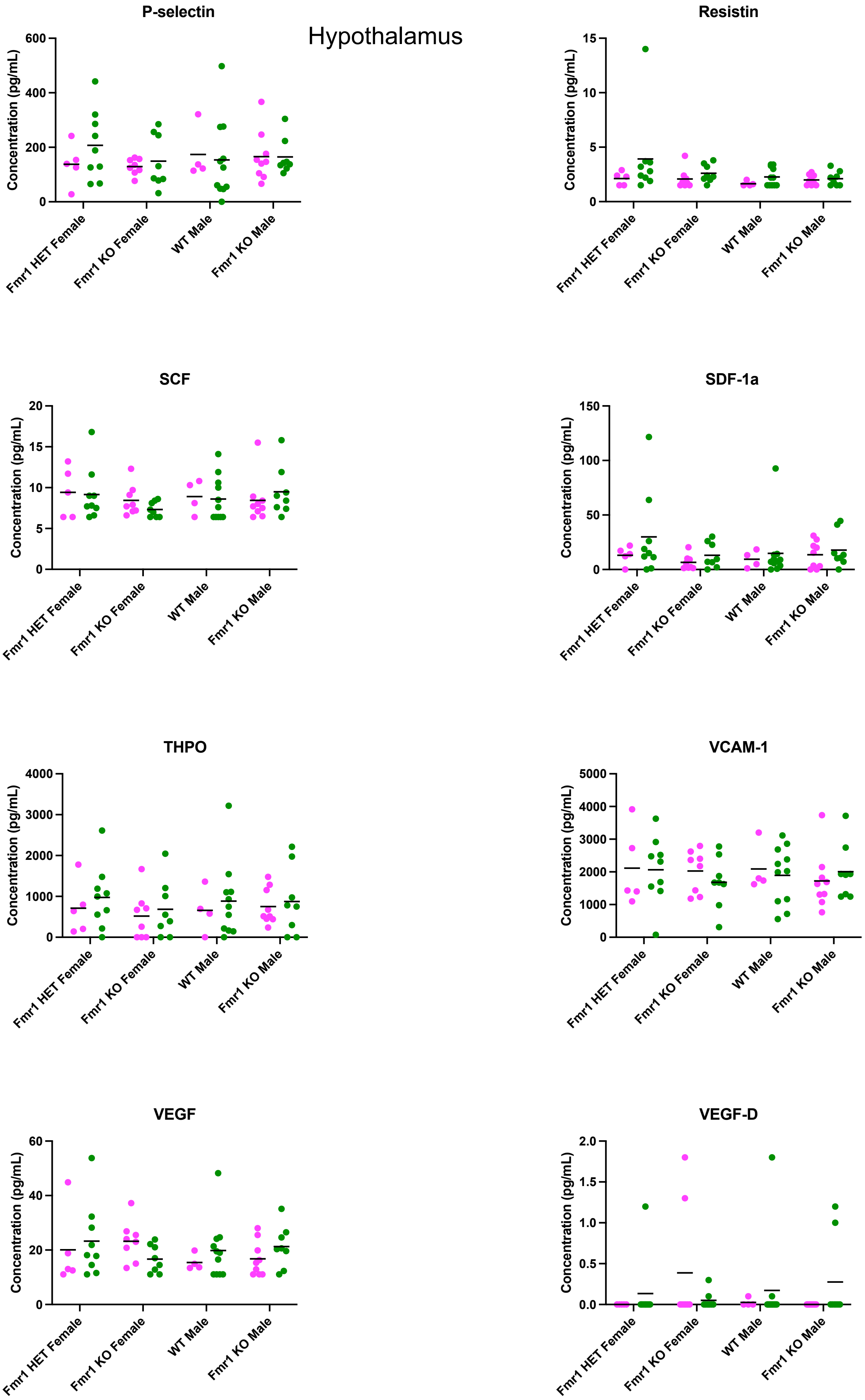
Hypothalamus
P-selectin
Resistin
SCF
SDF-1a
THPO
VCAM-1
VEGF
VEGF-D
Concentration (pg/mL)
Fmr1 HET Female
Fmr1 KO Female
WT Male
Fmr1 KO Male

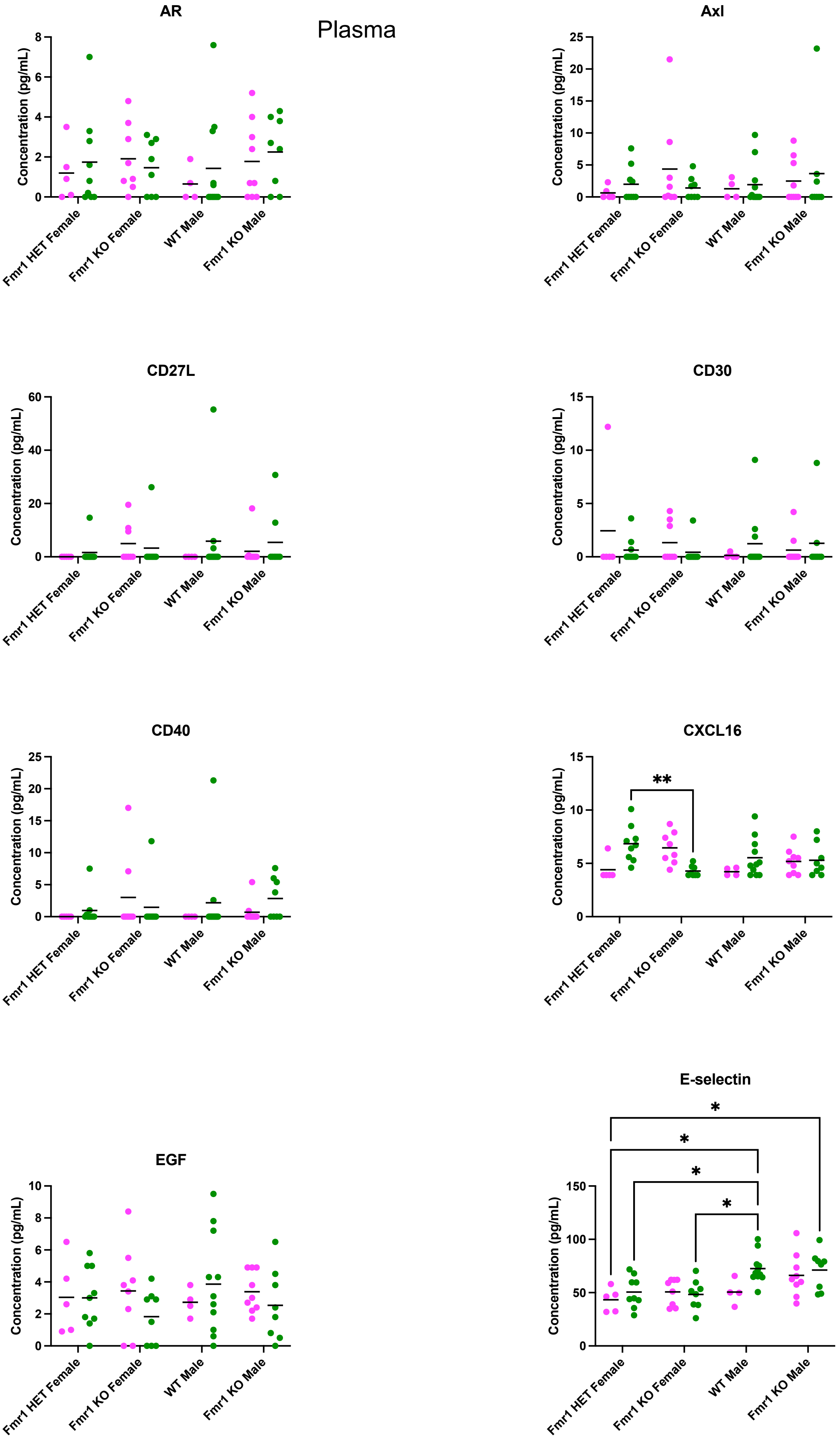
Plasma
AR
Axl
CD27L
CD30
CD40
CXCL16
EGF
E-selectin
Concentration (pg/mL)
Fmr1 HET Female
Fmr1 KO Female
WT Male
Fmr1 KO Male
**
*

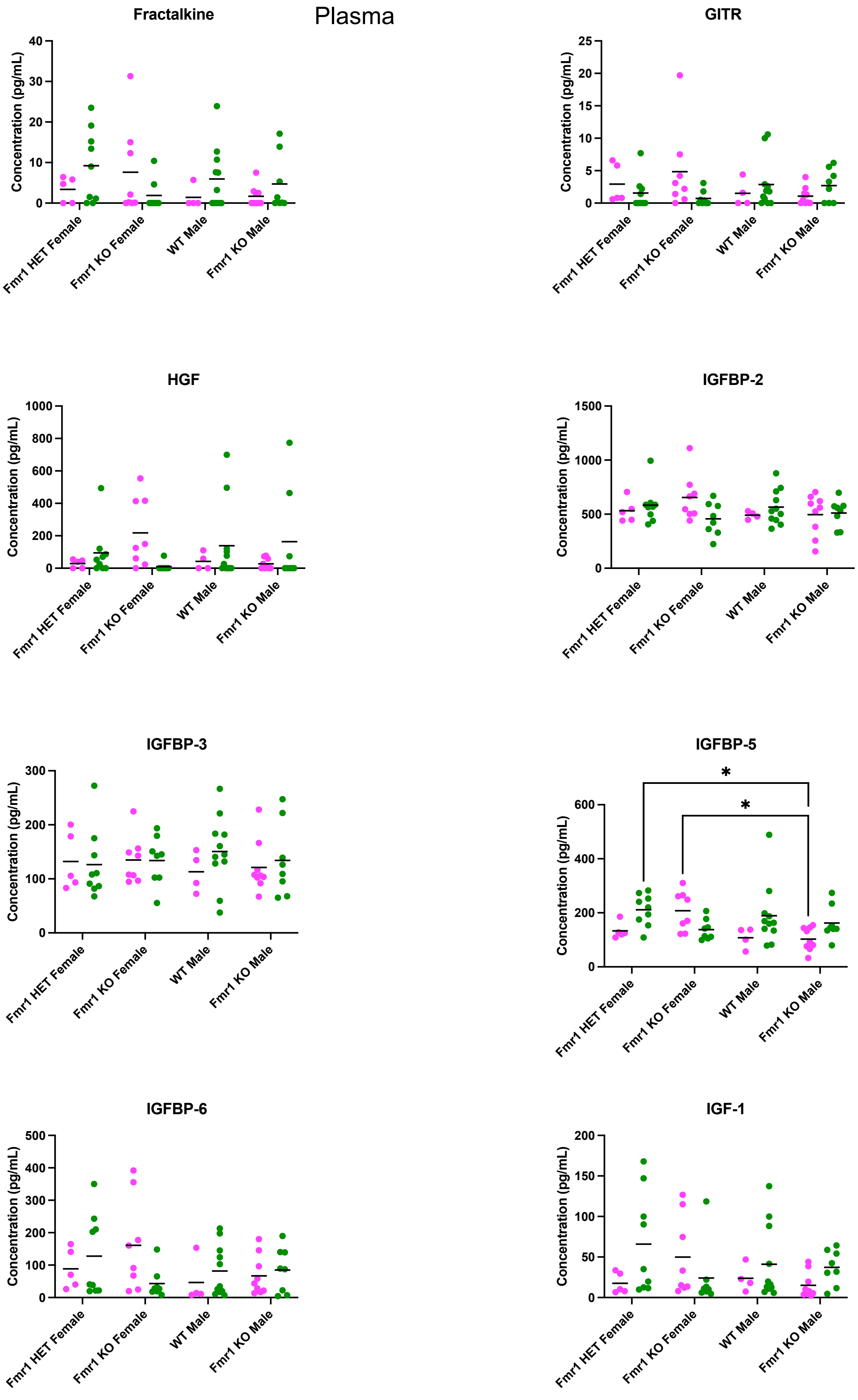
Plasma
Fractalkine
GITR
HGF
IGFBP-2
IGFBP-3
IGFBP-5
IGFBP-6
IGF-1
Concentration (pg/mL)
0
10
20
30
40
5
15
25
200
400
600
800
1000
500
1500
100
300
50
150
*
*
Fmr1 HET Female
Fmr1 KO Female
WT Male
Fmr1 KO Male

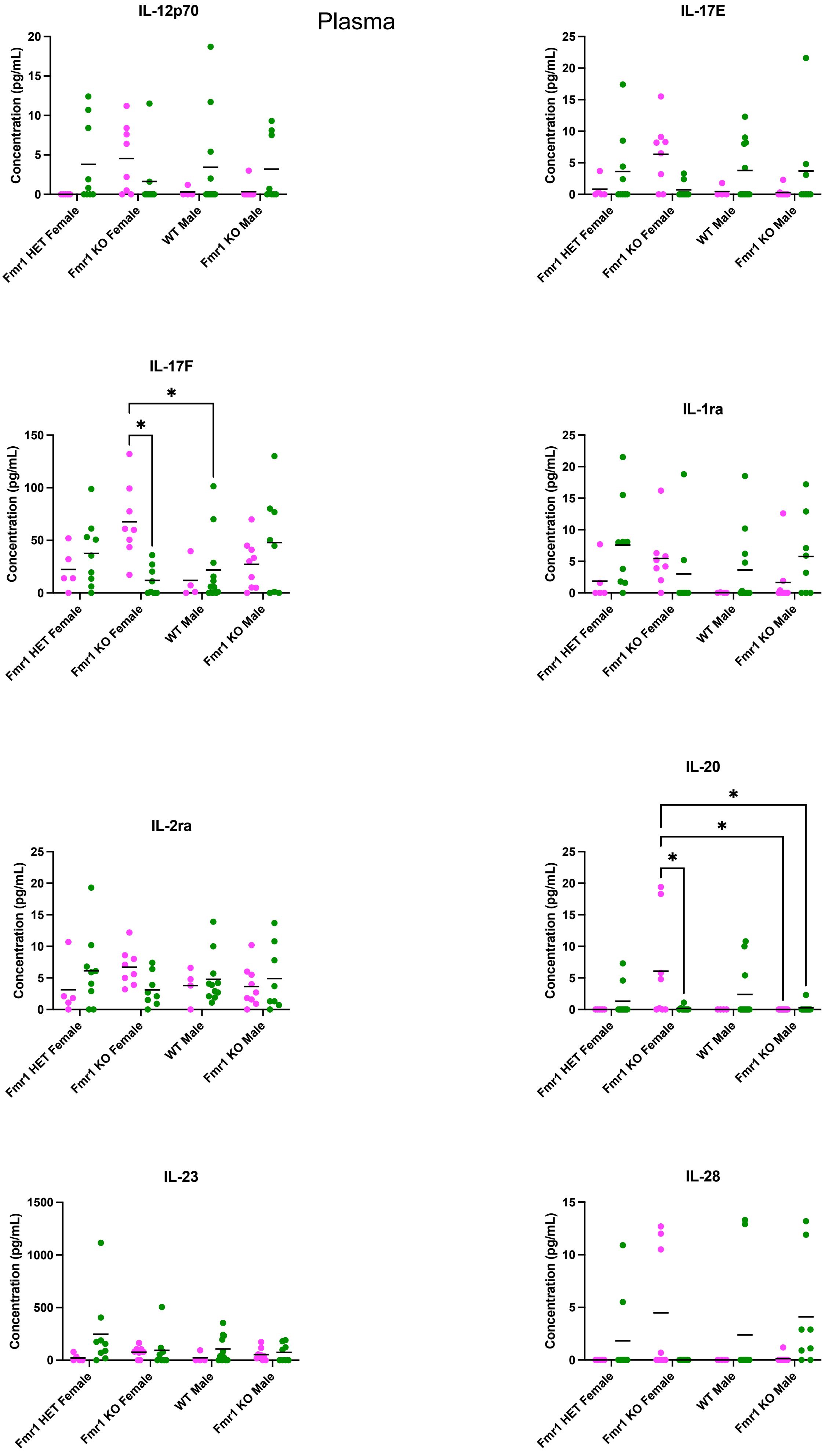
Plasma
IL-12p70
IL-17E
IL-17F
IL-1ra
IL-2ra
IL-20
IL-23
IL-28
Concentration (pg/mL)
Fmr1 HET Female
Fmr1 KO Female
WT Male
Fmr1 KO Male
*

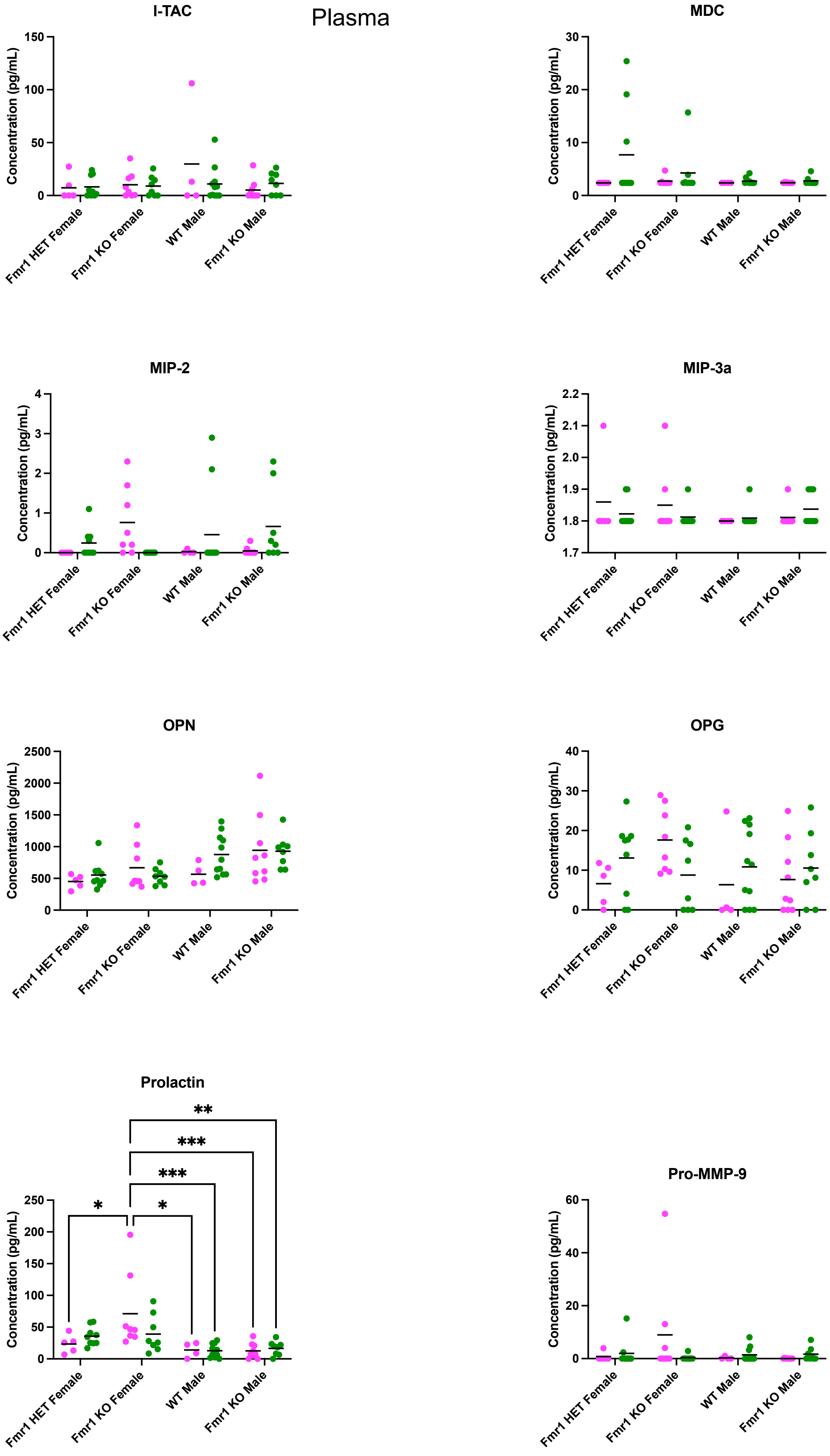

Plasma
I-TAC
MDC
MIP-2
MIP-3a
OPN
OPG
Prolactin
Pro-MMP-9
Concentration (pg/mL)
Fmr1 HET Female
Fmr1 KO Female
WT Male
Fmr1 KO Male
*
*
***
***
**

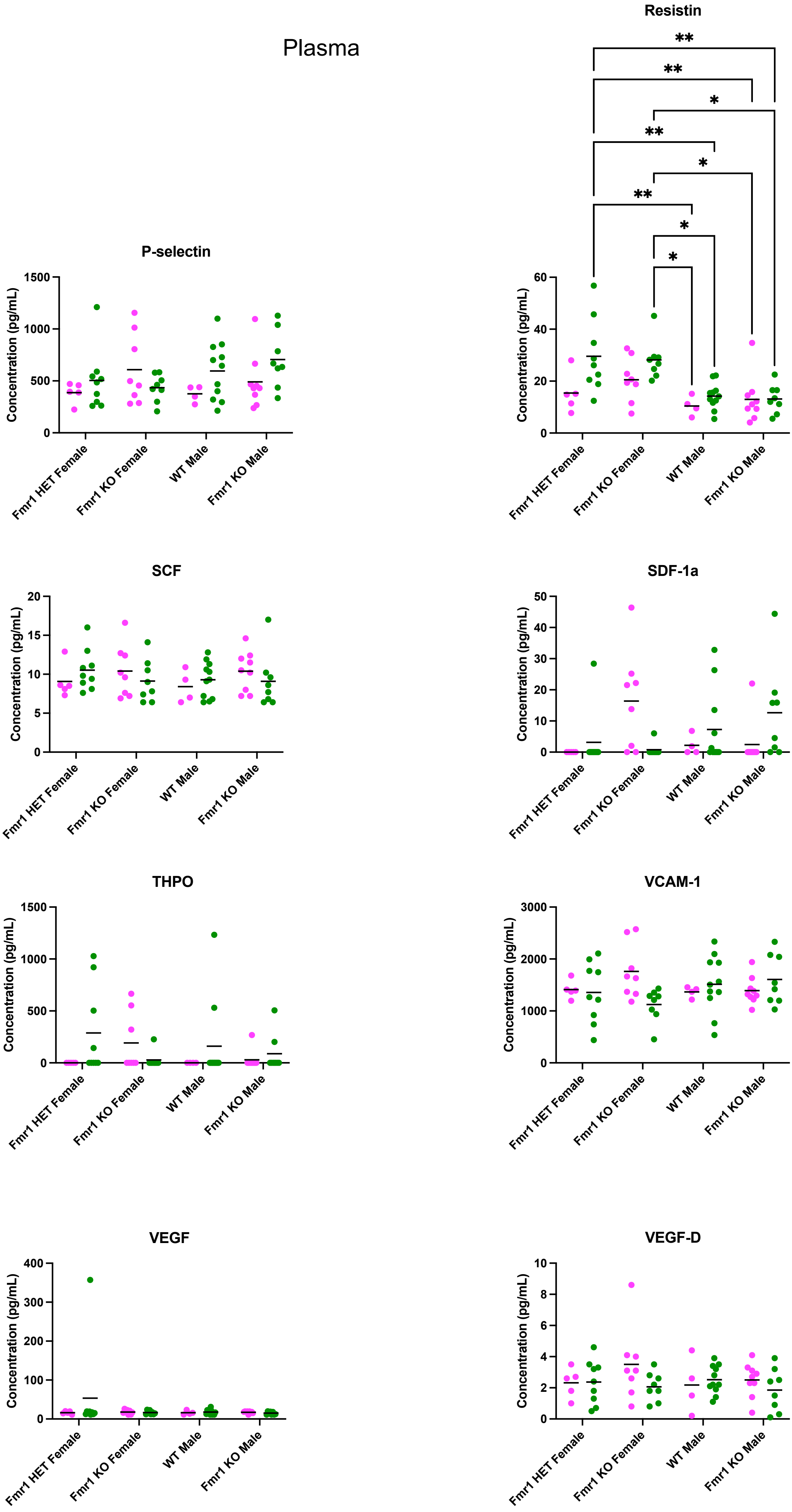
Plasma
P-selectin
Resistin
SCF
SDF-1a
THPO
VCAM-1
VEGF
VEGF-D
Concentration (pg/mL)
Fmr1 HET Female
Fmr1 KO Female
WT Male
Fmr1 KO Male
**
*
0
500
1000
1500
20
40
60
5
10
15
30
50
2000
3000
100
200
300
400
2
4
6
8
