# Supplementary material for: Soy Protein Isolate Affects Blood and Brain Biomarker Expression in a Mouse Model of Fragile X
Source: Int J Mol Sci. 2025 Jun 26;26(13):6137. doi: 10.3390/ijms26136137 (PMC12250412; doi:10.3390/ijms26136137)
Supplement: Supplementary file 1 [file ijms-26-06137-s001.zip › Supplementary File S3b Array 5 Graphs.pdf]

Supplementary File S3. Protein expression of Array 5 targets as function of *Fmr1* genotype and AIN-93G diets. Mice on AIN-93G/cas (colored pink) included n=5 *Fmr1*^{HET} female, n=8 *Fmr1*^{KO} female, n=4 WT male and n=9 *Fmr1*^{KO} male. Mice on AIN-93G/soy (colored green) included n=9 *Fmr1*^{HET} female, n=8 *Fmr1*^{KO} female, n=11 WT male and n=8 *Fmr1*^{KO} male. The average concentration in cortex, hippocampus, hypothalamus and plasma in pg/mL was plotted versus genotype. Statistics were determined by 2-way ANOVA and Tukey's multiple comparison tests denoted by $p < 0.05$ (*), $p < 0.01$ (**), $p < 0.001$ (***) and $p < 0.0001$ (****).

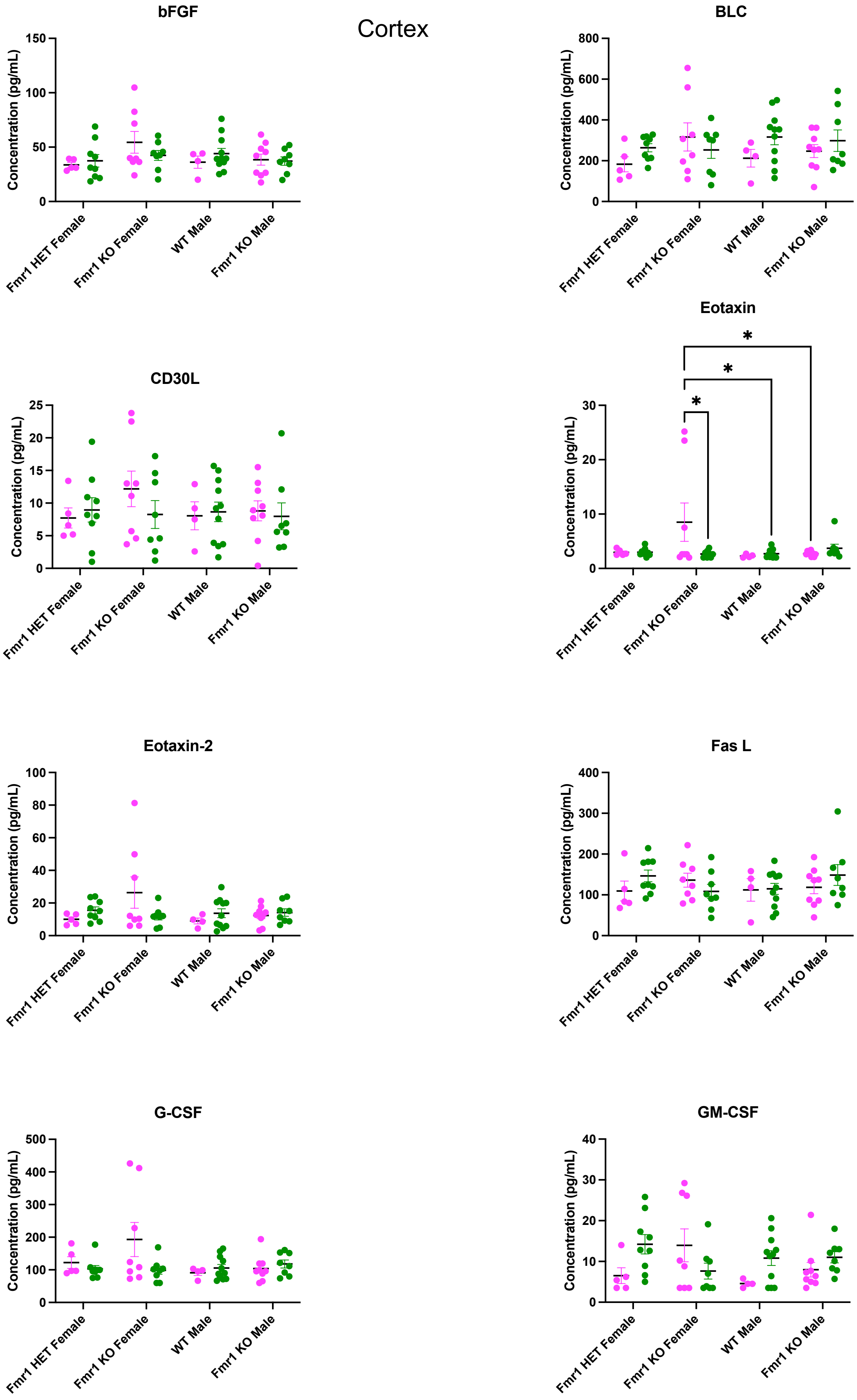
Cortex
bFGF
BLC
CD30L
Eotaxin
Eotaxin-2
Fas L
G-CSF
GM-CSF
Concentration (pg/mL)
Fmr1 HET Female
Fmr1 KO Female
WT Male
Fmr1 KO Male
*

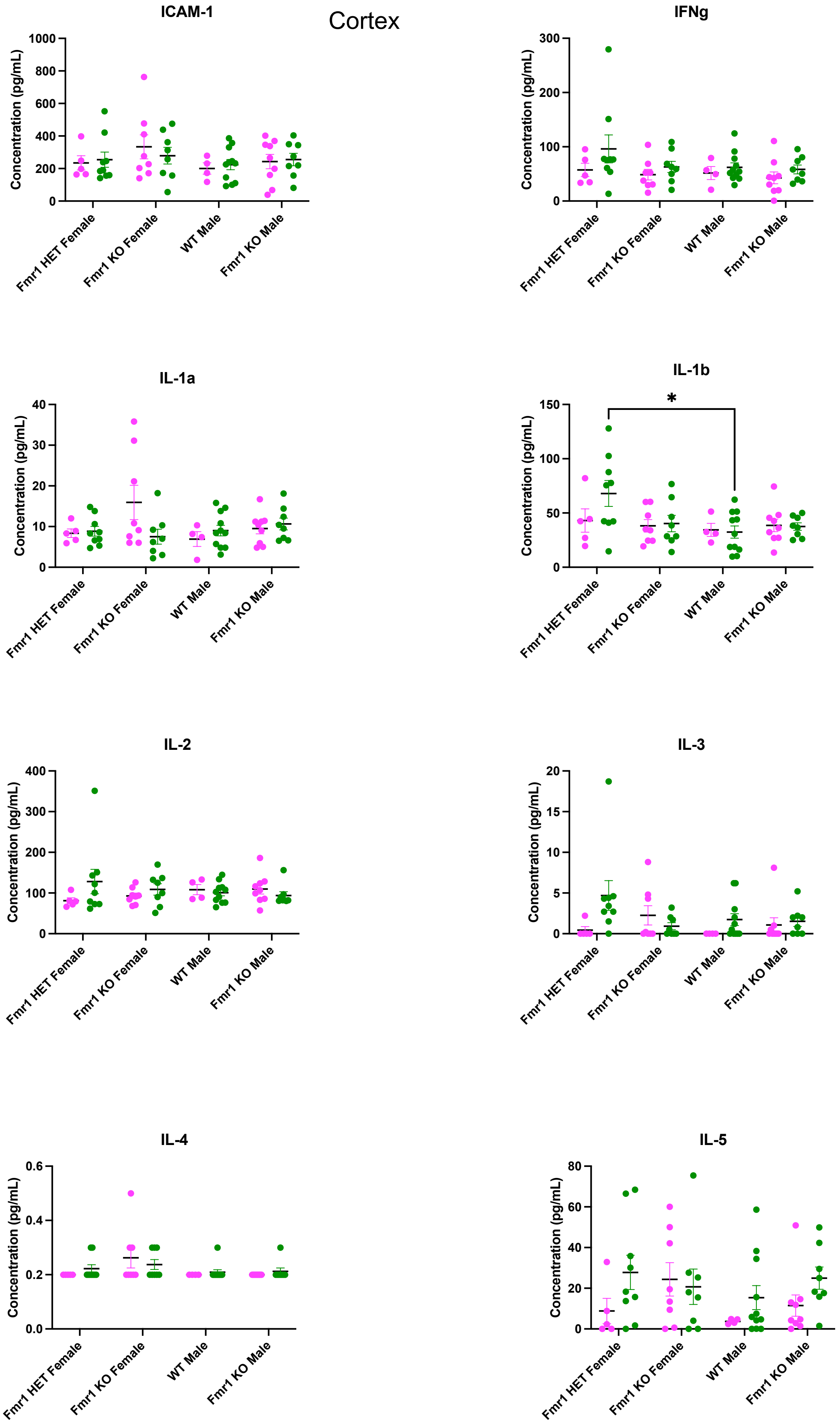
Cortex
ICAM-1
IFNg
IL-1a
IL-1b
*
IL-2
IL-3
IL-4
IL-5
Concentration (pg/mL)
Fmr1 HET Female
Fmr1 KO Female
WT Male
Fmr1 KO Male
0
200
400
600
800
1000
100
300
10
20
30
40
50
150
5
15
0.0
0.2
0.4
0.6
60
80

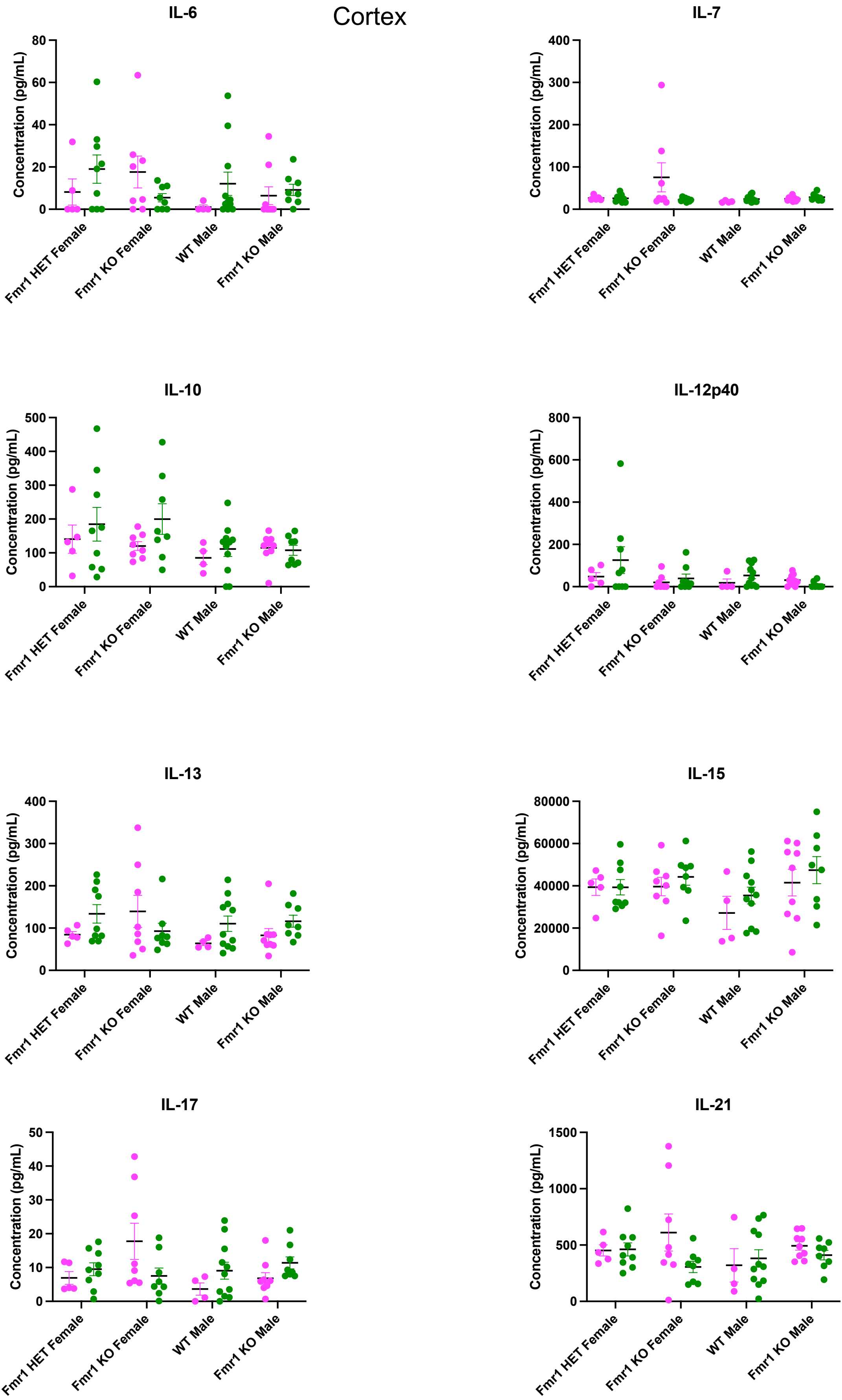
Cortex
IL-6
IL-7
IL-10
IL-12p40
IL-13
IL-15
IL-17
IL-21
Concentration (pg/mL)
Fmr1 HET Female
Fmr1 KO Female
WT Male
Fmr1 KO Male
0
20
40
60
80
100
200
300
400
500
600
800
1000
1500
20000
40000
60000
80000
10
30
50

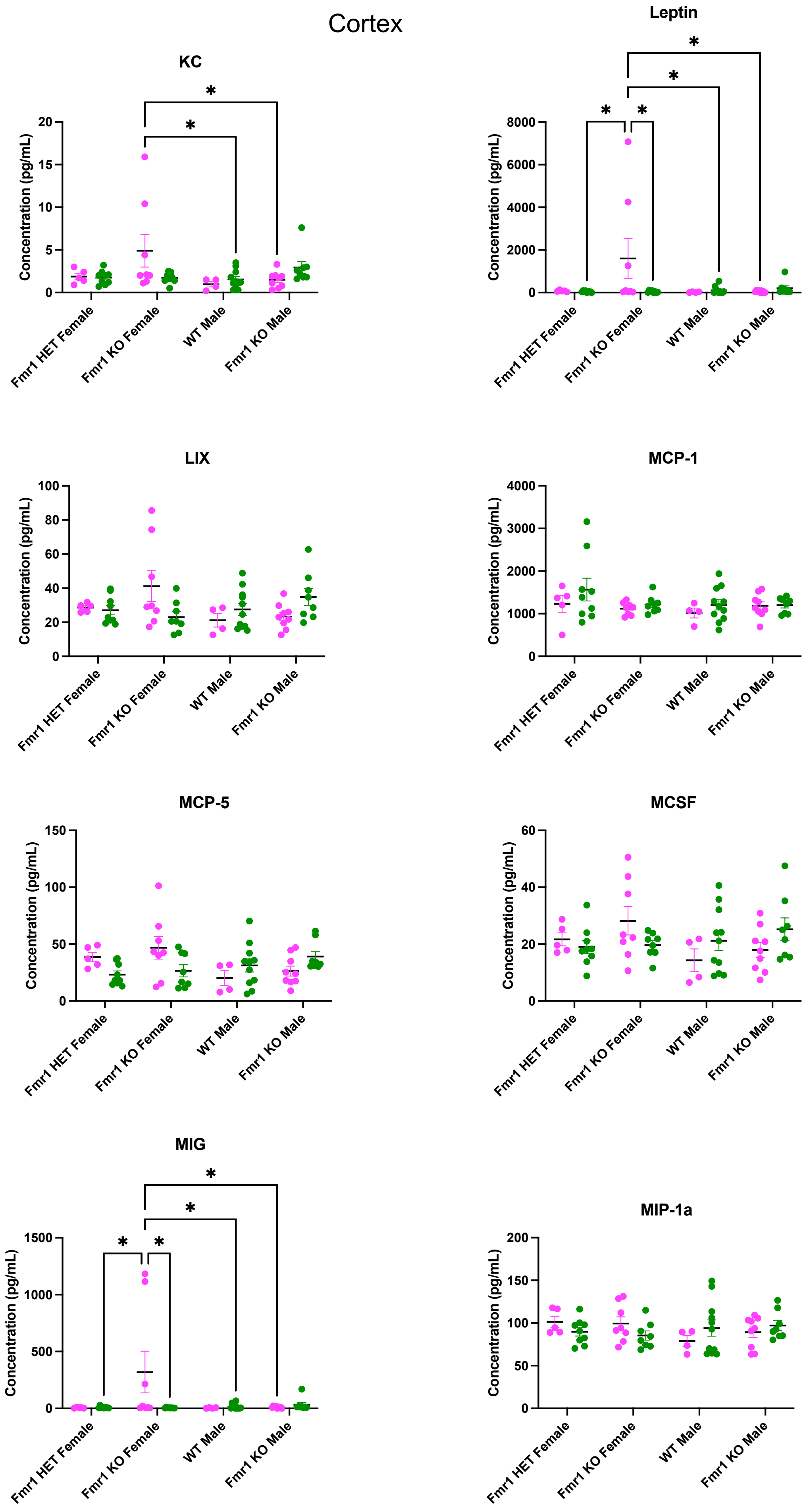

Cortex
KC
Leptin
LIX
MCP-1
MCP-5
MCSF
MIG
MIP-1a
Concentration (pg/mL)
Fmr1 HET Female
Fmr1 KO Female
WT Male
Fmr1 KO Male
*
0
5
10
15
20
2000
4000
6000
8000
40
60
80
100
1000
3000
50
100
150
500
1500
200

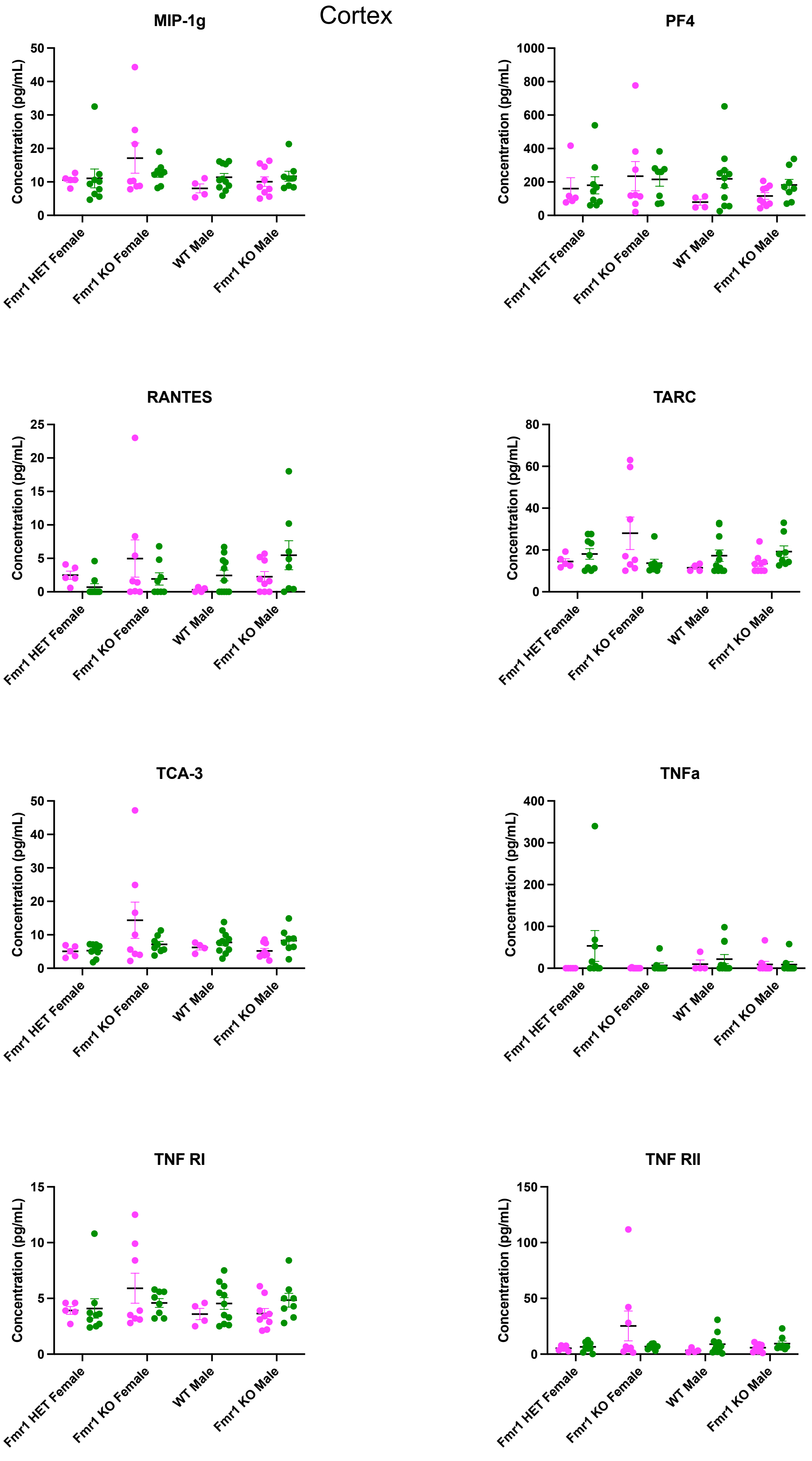
Cortex
MIP-1g
PF4
RANTES
TARC
TCA-3
TNFa
TNF RI
TNF RII
Concentration (pg/mL)
Fmr1 HET Female
Fmr1 KO Female
WT Male
Fmr1 KO Male
0
10
20
30
40
50
200
400
600
800
1000
5
15
25
60
80
100
300
150

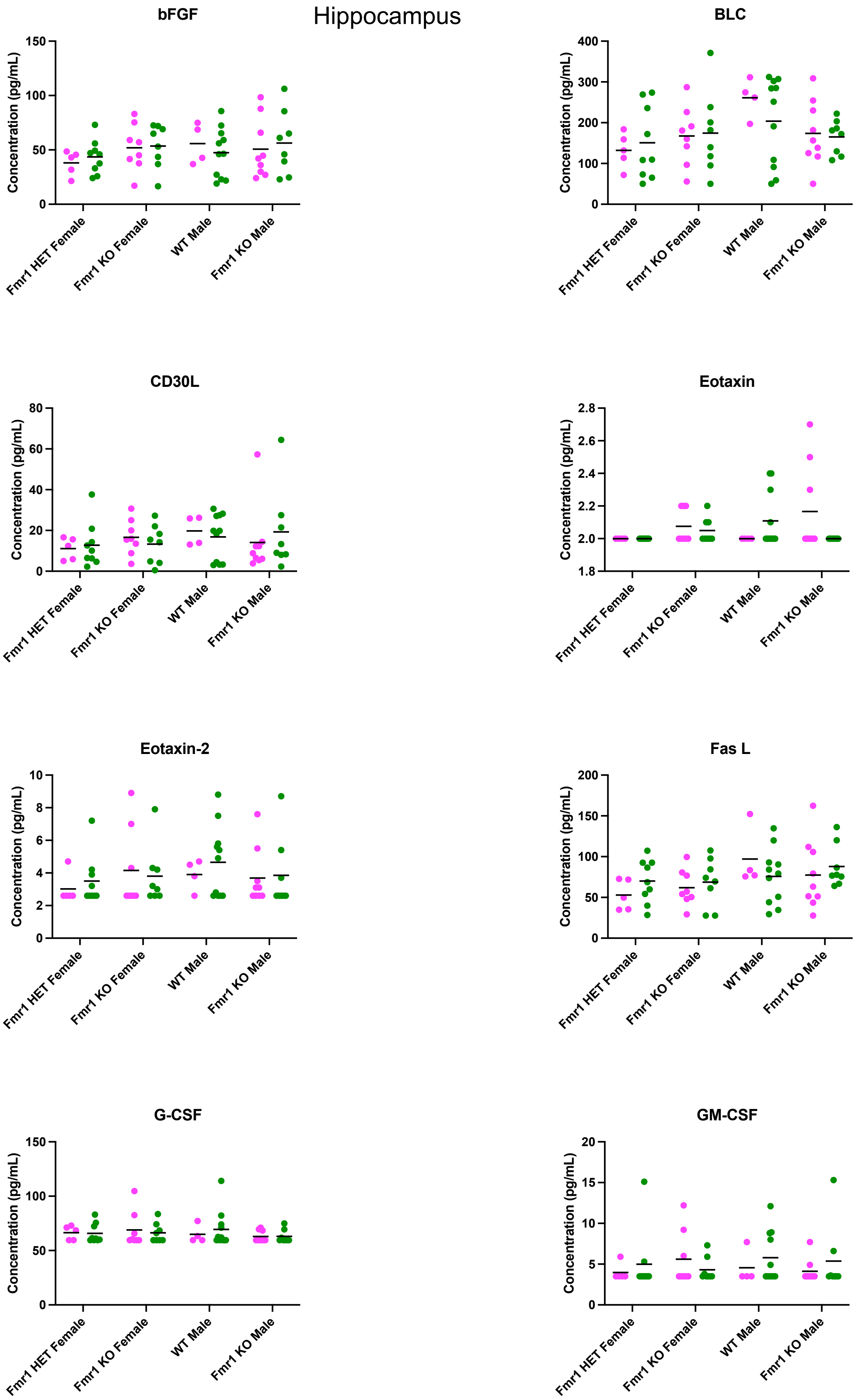
Hippocampus
bFGF
BLC
CD30L
Eotaxin
Eotaxin-2
Fas L
G-CSF
GM-CSF
Concentration (pg/mL)
Fmr1 HET Female
Fmr1 KO Female
WT Male
Fmr1 KO Male

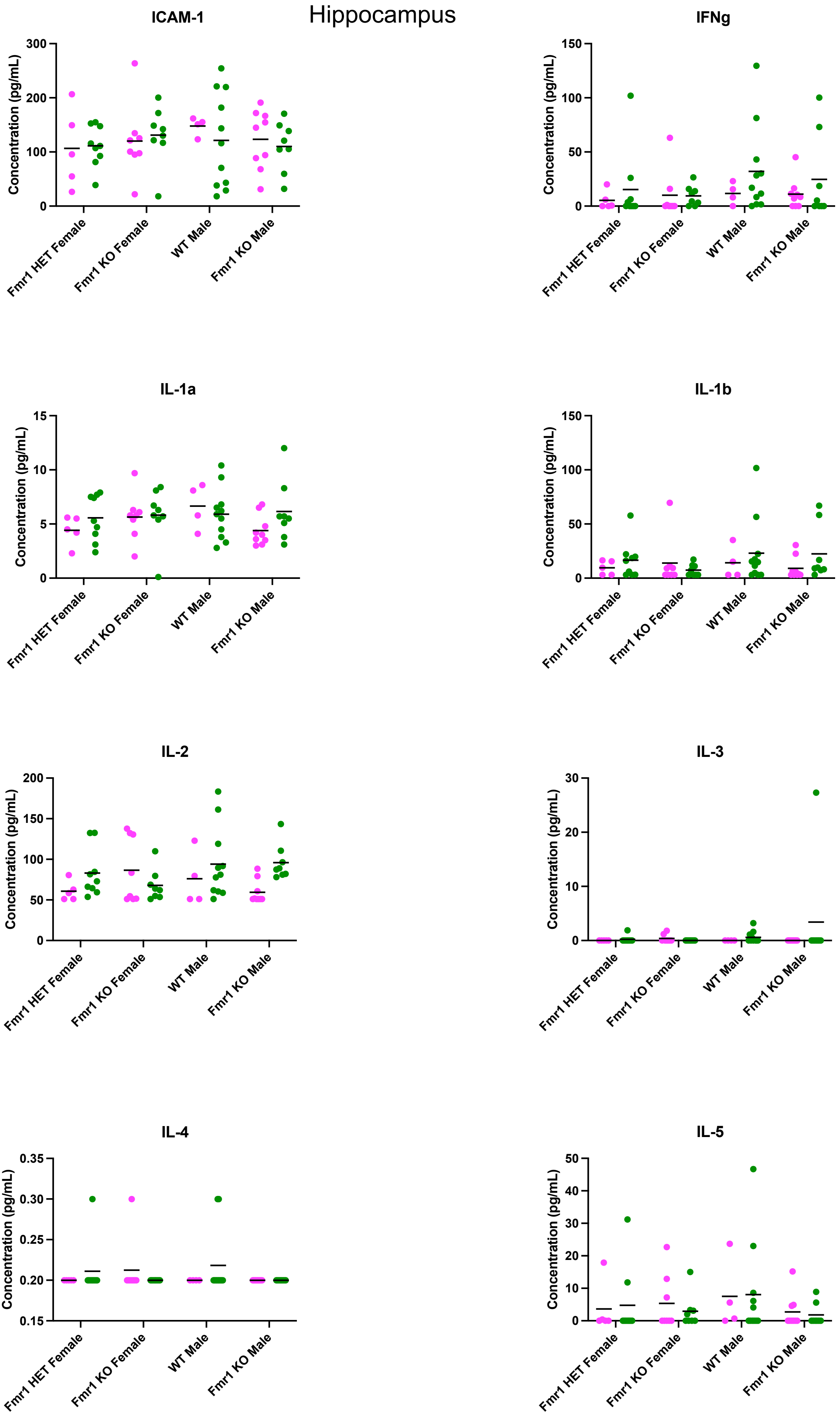
Hippocampus
ICAM-1
IFNg
IL-1a
IL-1b
IL-2
IL-3
IL-4
IL-5
Concentration (pg/mL)
Fmr1 HET Female
Fmr1 KO Female
WT Male
Fmr1 KO Male
0
100
200
300
50
150
5
10
15
20
30
0.15
0.20
0.25
0.30
0.35
40

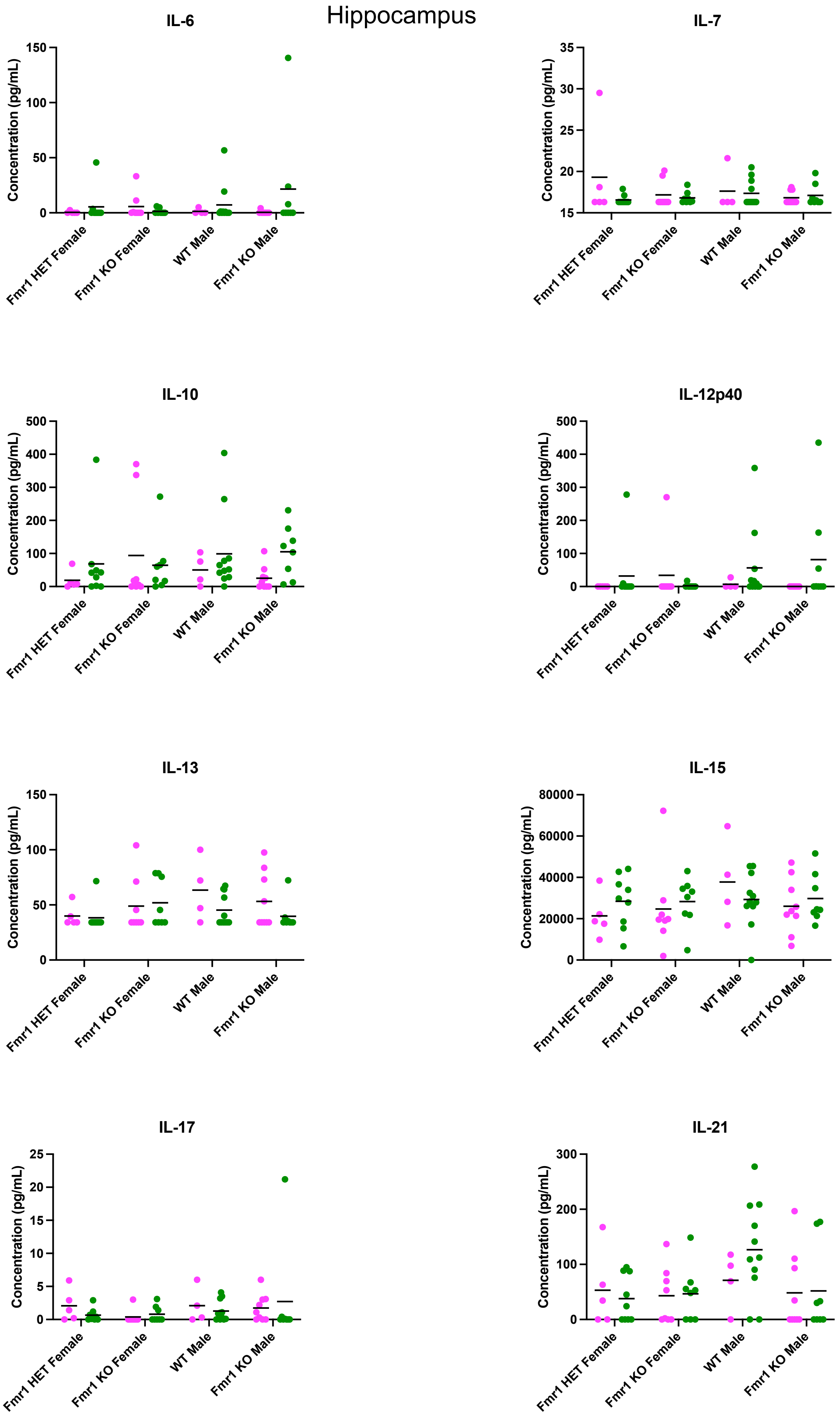
Hippocampus
IL-6
IL-7
IL-10
IL-12p40
IL-13
IL-15
IL-17
IL-21
Concentration (pg/mL)
0
50
100
150
15
20
25
30
35
200
300
400
500
20000
40000
60000
80000
5
10
Fmr1 HET Female
Fmr1 KO Female
WT Male
Fmr1 KO Male

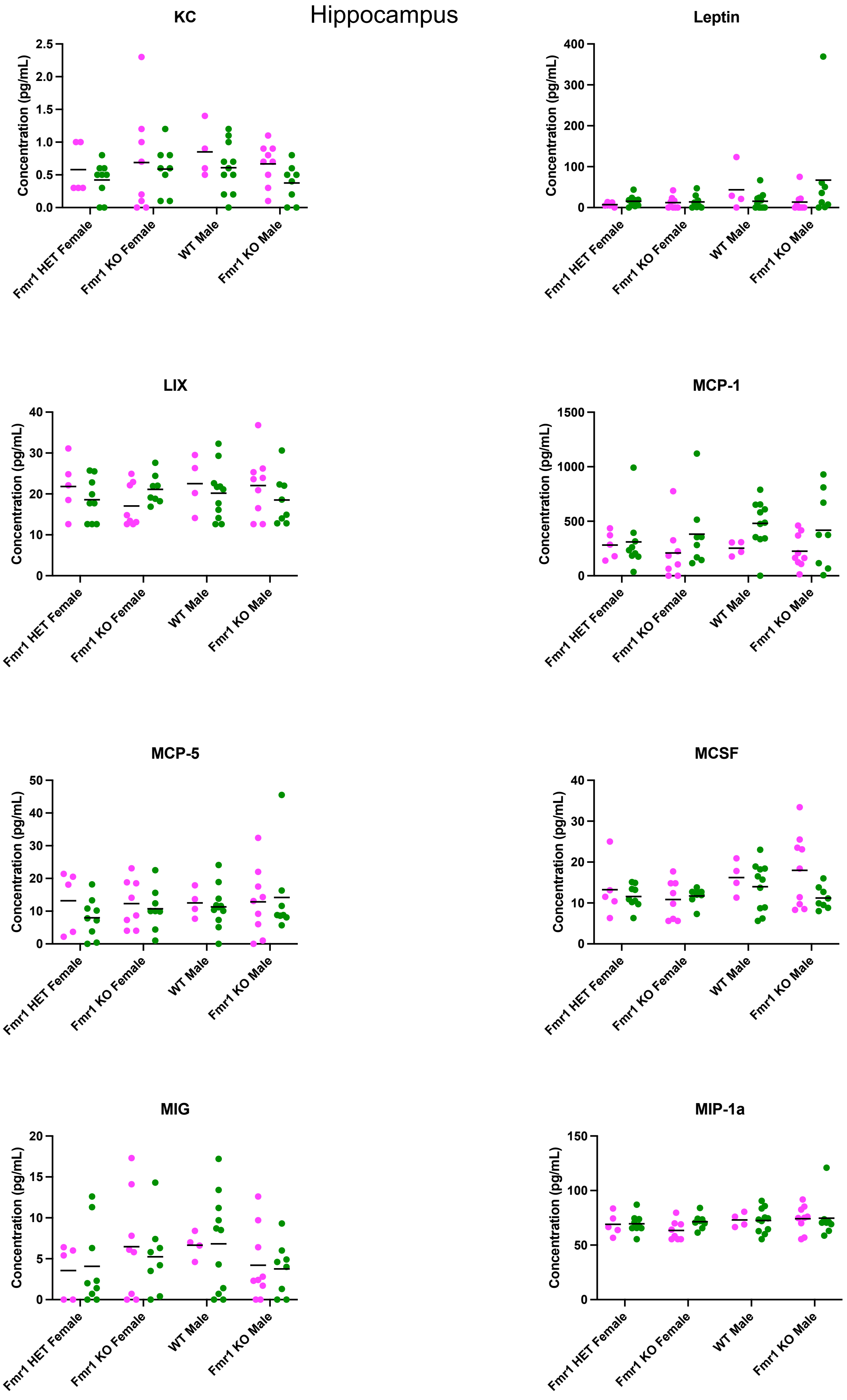

Hippocampus
KC
Leptin
LIX
MCP-1
MCP-5
MCSF
MIG
MIP-1a
Concentration (pg/mL)
0.0
0.5
1.0
1.5
2.0
2.5
0
100
200
300
400
10
20
30
40
500
1000
1500
50
5
15
150
Fmr1 HET Female
Fmr1 KO Female
WT Male
Fmr1 KO Male

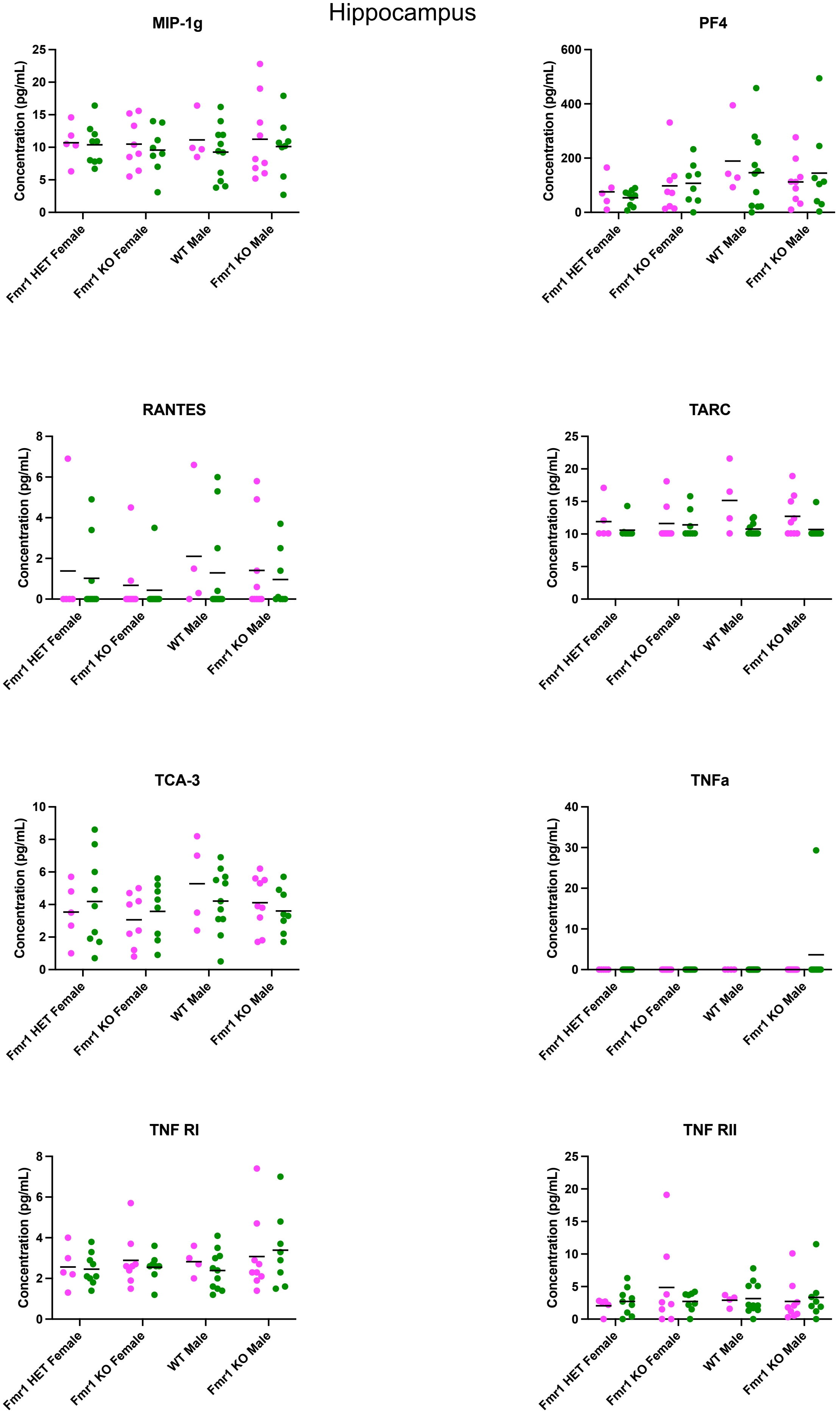
Hippocampus
MIP-1g
PF4
RANTES
TARC
TCA-3
TNFa
TNF RI
TNF RII
Concentration (pg/mL)
Fmr1 HET Female
Fmr1 KO Female
WT Male
Fmr1 KO Male

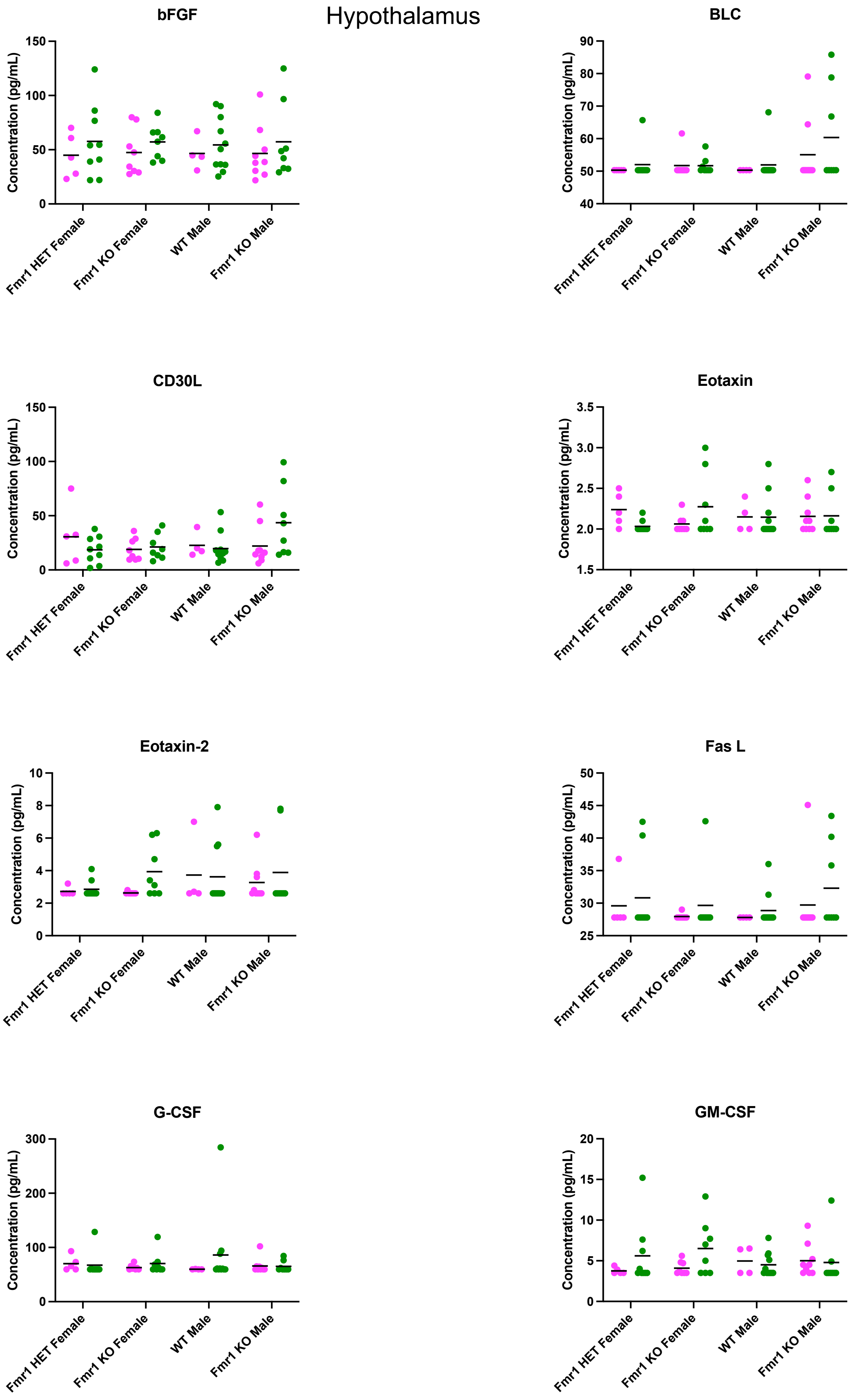
Hypothalamus
bFGF
BLC
CD30L
Eotaxin
Eotaxin-2
Fas L
G-CSF
GM-CSF
Concentration (pg/mL)
Fmr1 HET Female
Fmr1 KO Female
WT Male
Fmr1 KO Male

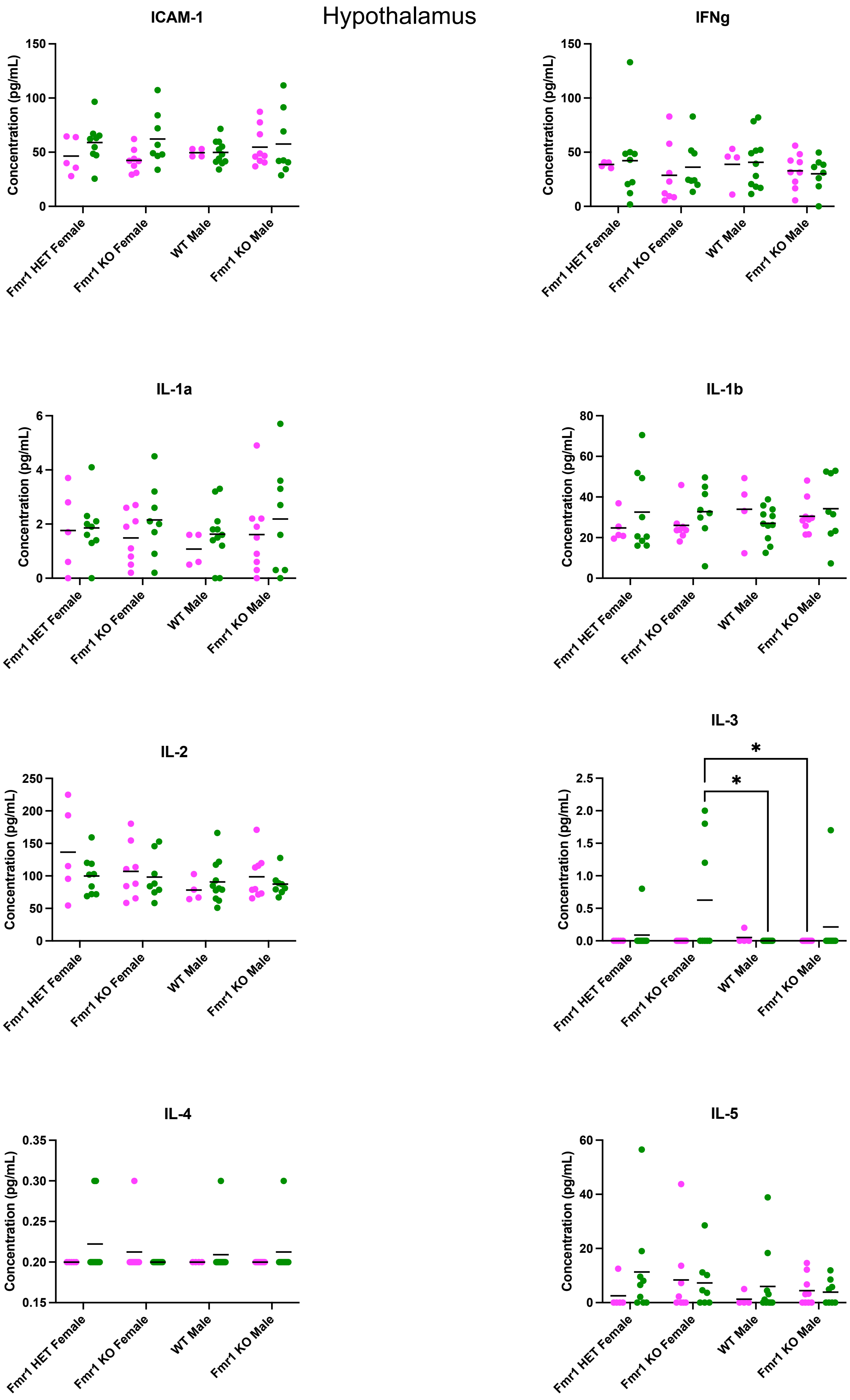
Hypothalamus
ICAM-1
IFNg
IL-1a
IL-1b
IL-2
IL-3
IL-4
IL-5
Concentration (pg/mL)
Fmr1 HET Female
Fmr1 KO Female
WT Male
Fmr1 KO Male
*
*
0
50
100
150
2
4
6
20
40
60
80
200
250
0.0
0.5
1.0
1.5
2.0
2.5
0.15
0.20
0.25
0.30
0.35

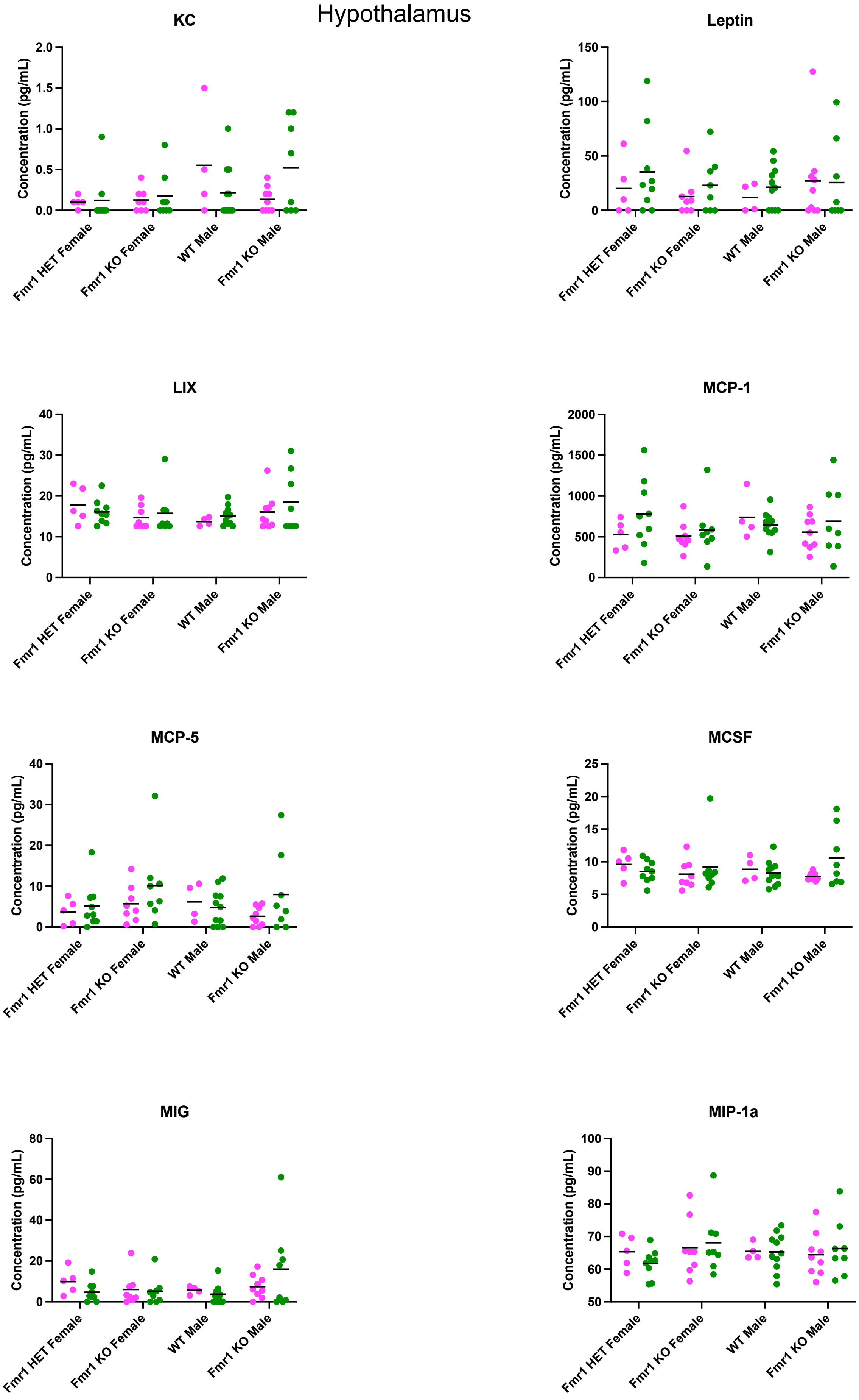
Hypothalamus
KC
Leptin
LIX
MCP-1
MCP-5
MCSF
MIG
MIP-1a
Concentration (pg/mL)
Fmr1 HET Female
Fmr1 KO Female
WT Male
Fmr1 KO Male

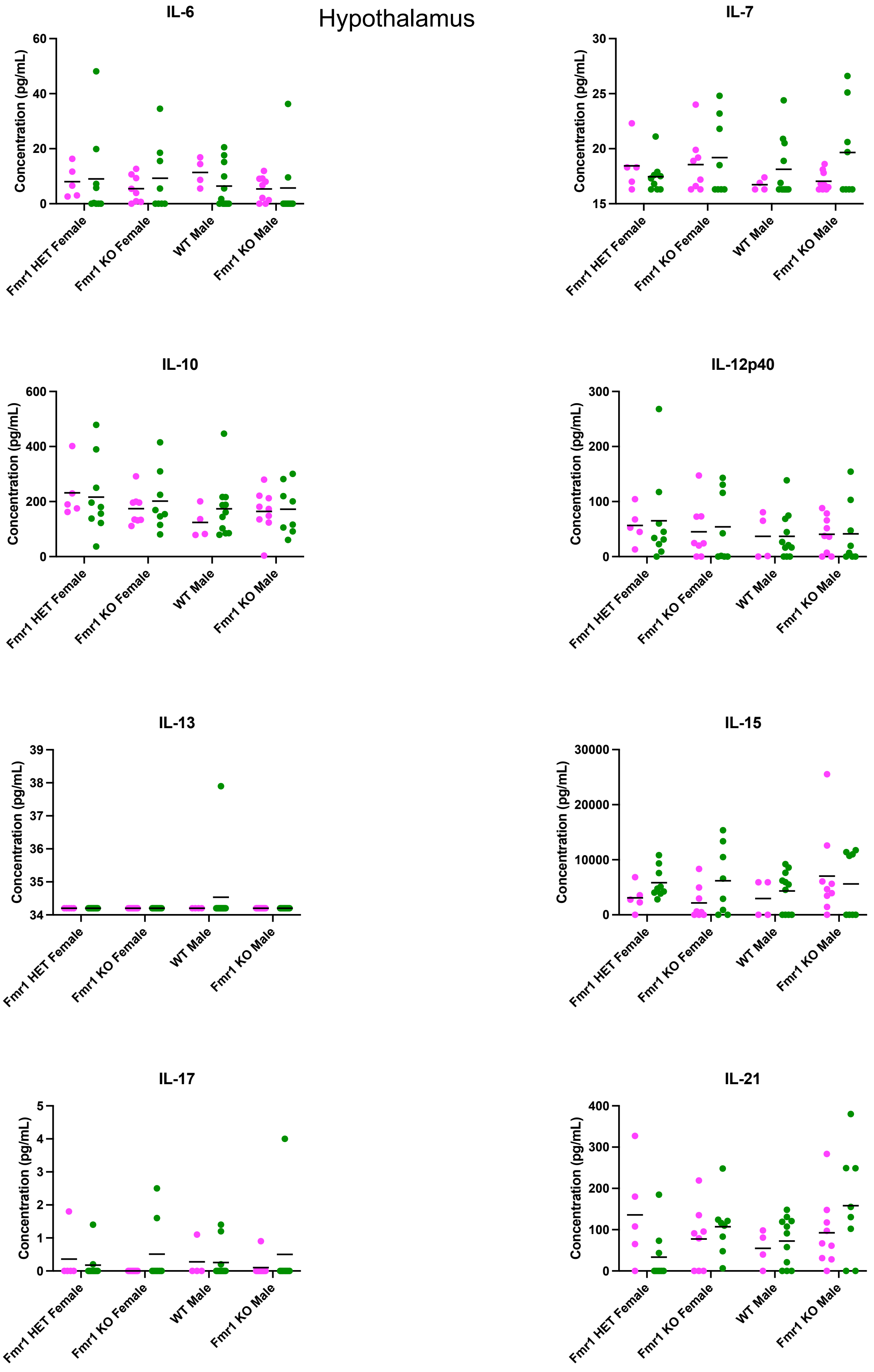
Hypothalamus
IL-6
IL-7
IL-10
IL-12p40
IL-13
IL-15
IL-17
IL-21
Concentration (pg/mL)
Fmr1 HET Female
Fmr1 KO Female
WT Male
Fmr1 KO Male

Hypothalamus

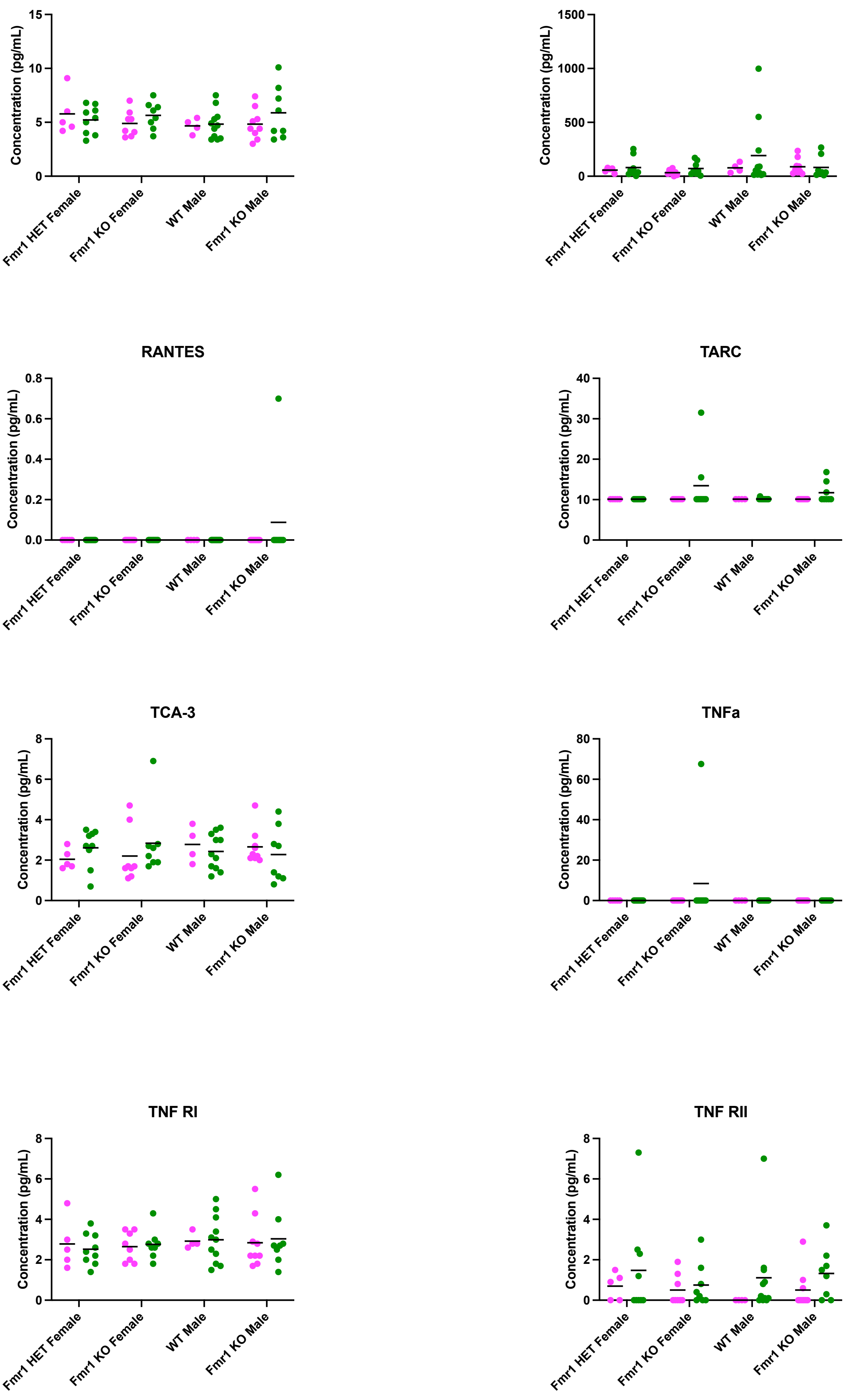

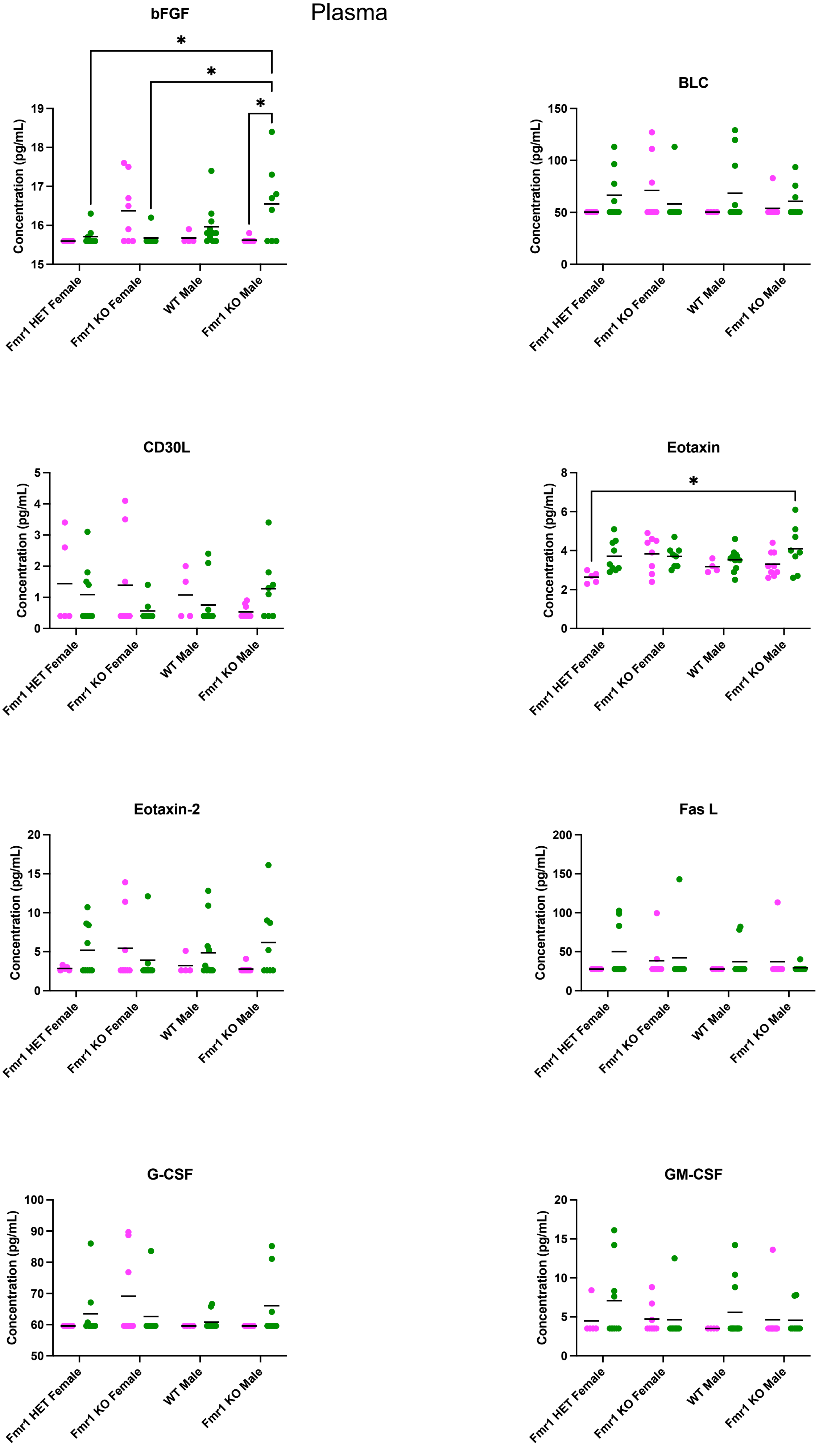
Plasma
bFGF
*
*
*
Concentration (pg/mL)
19
18
17
16
15
Fmr1 HET Female
Fmr1 KO Female
WT Male
Fmr1 KO Male
BLC
150
100
50
0
CD30L
5
4
3
2
1
0
Eotaxin
8
6
4
2
0
Eotaxin-2
20
15
10
5
0
Fas L
200
150
100
50
0
G-CSF
100
90
80
70
60
50
GM-CSF
20
15
10
5
0

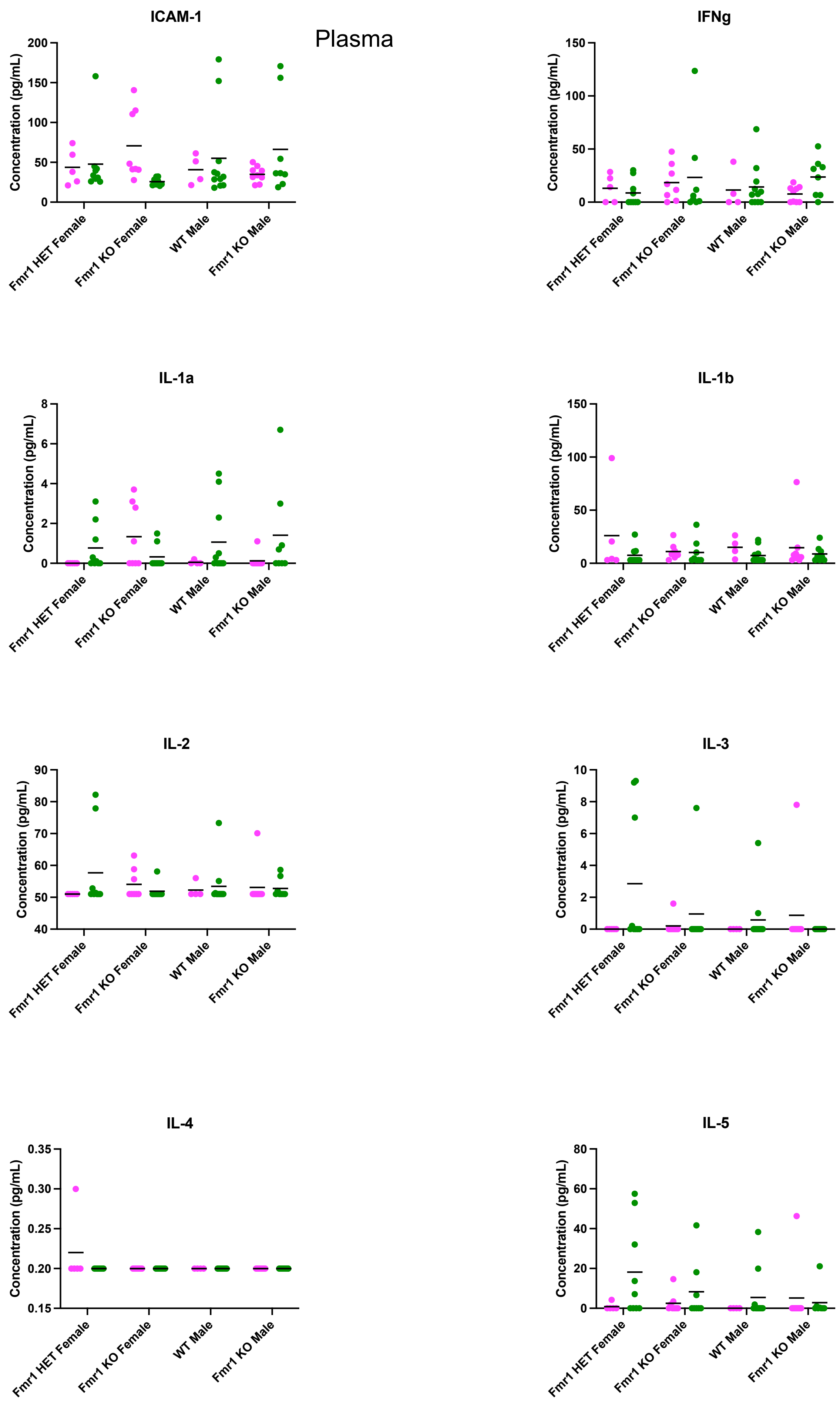
Plasma
ICAM-1
IFNg
IL-1a
IL-1b
IL-2
IL-3
IL-4
IL-5
Concentration (pg/mL)
Fmr1 HET Female
Fmr1 KO Female
WT Male
Fmr1 KO Male

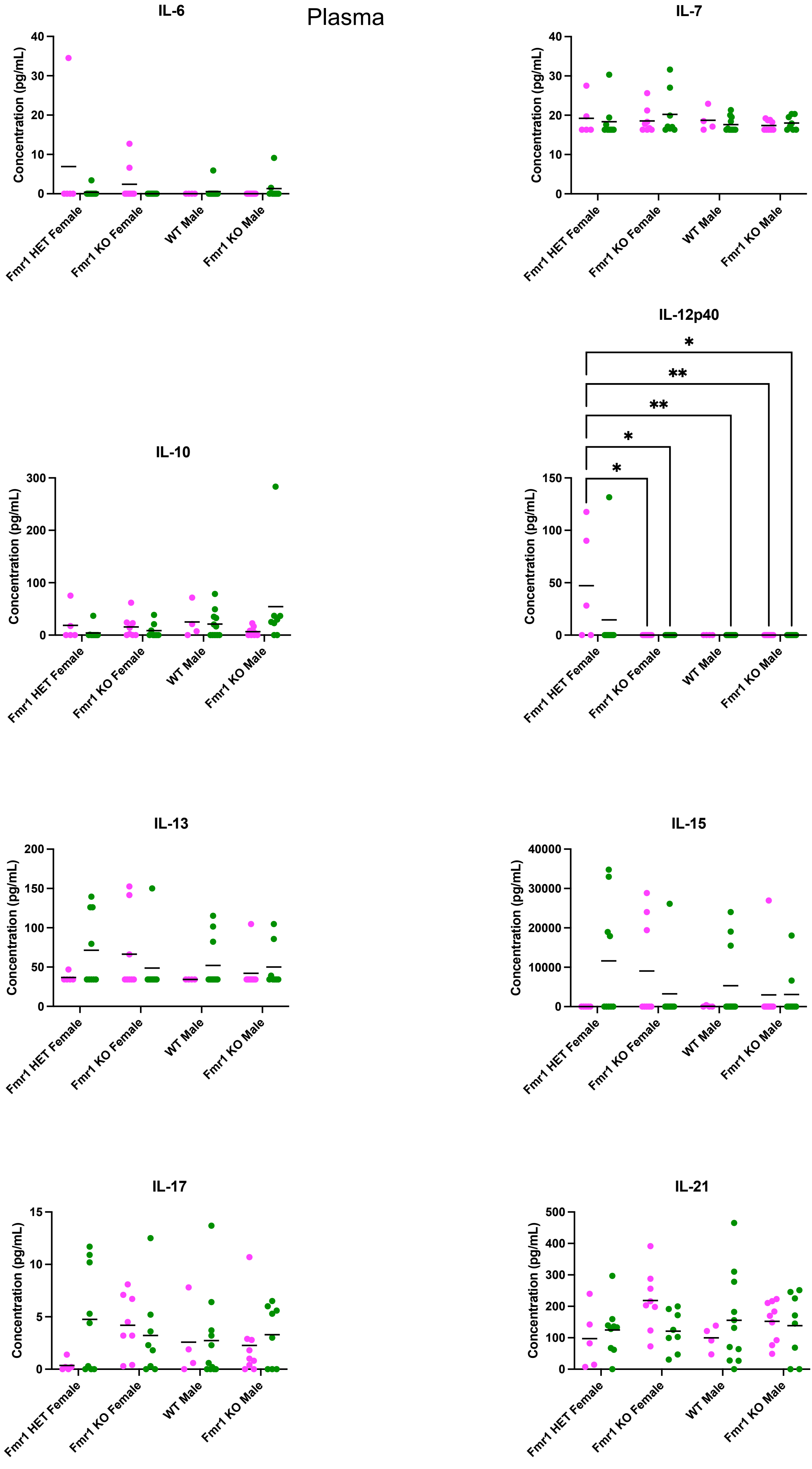
Plasma
IL-6
IL-7
IL-10
IL-12p40
IL-13
IL-15
IL-17
IL-21
Concentration (pg/mL)
Fmr1 HET Female
Fmr1 KO Female
WT Male
Fmr1 KO Male
*
**
**
*
*

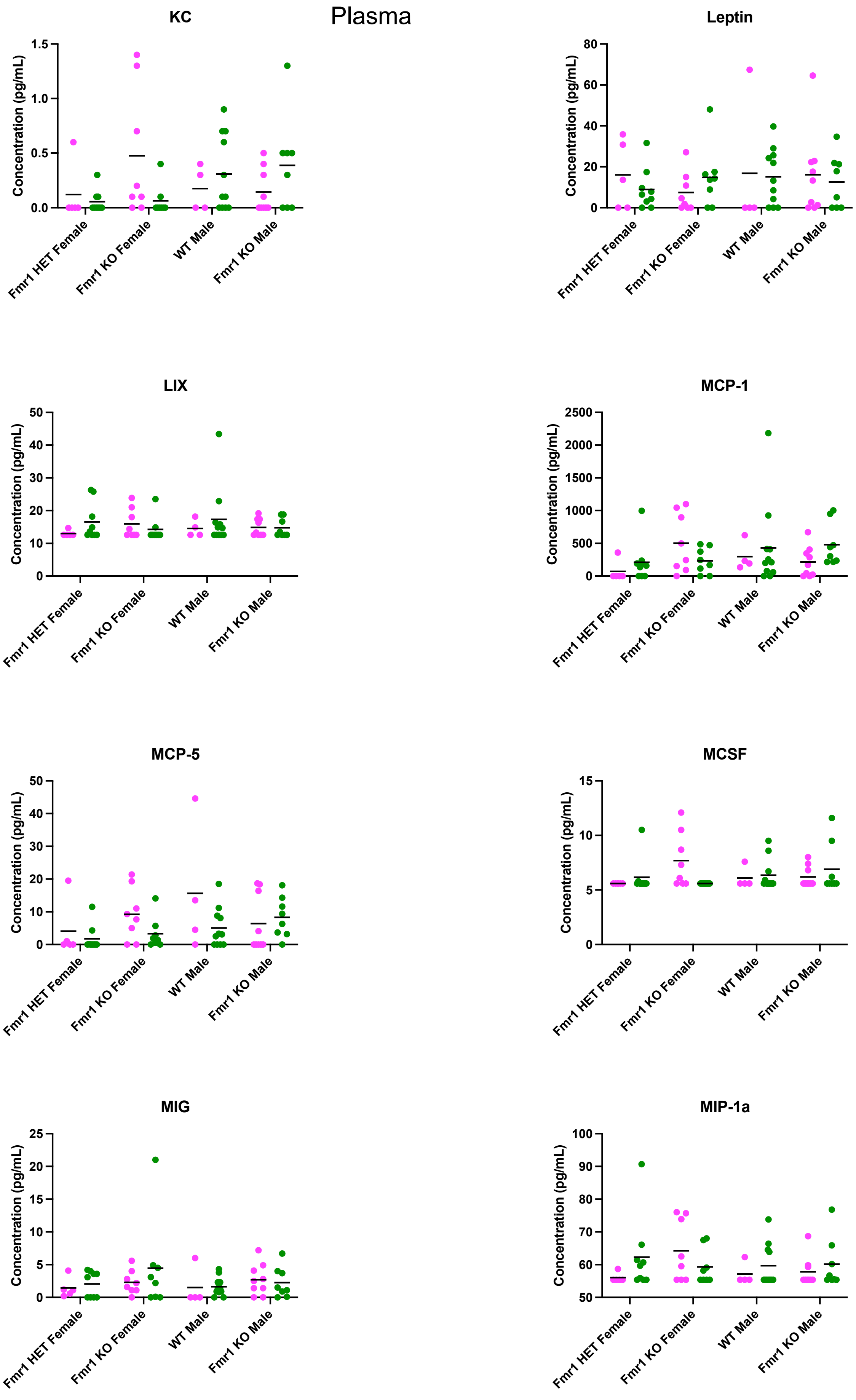
Plasma
KC
Concentration (pg/mL)
0.0
0.5
1.0
1.5
Leptin
Concentration (pg/mL)
0
20
40
60
80
LIX
Concentration (pg/mL)
0
10
20
30
40
50
MCP-1
Concentration (pg/mL)
0
500
1000
1500
2000
2500
MCP-5
Concentration (pg/mL)
0
10
20
30
40
50
MCSF
Concentration (pg/mL)
0
5
10
15
MIG
Concentration (pg/mL)
0
5
10
15
20
25
MIP-1a
Concentration (pg/mL)
50
60
70
80
90
100
Fmr1 HET Female
Fmr1 KO Female
WT Male
Fmr1 KO Male

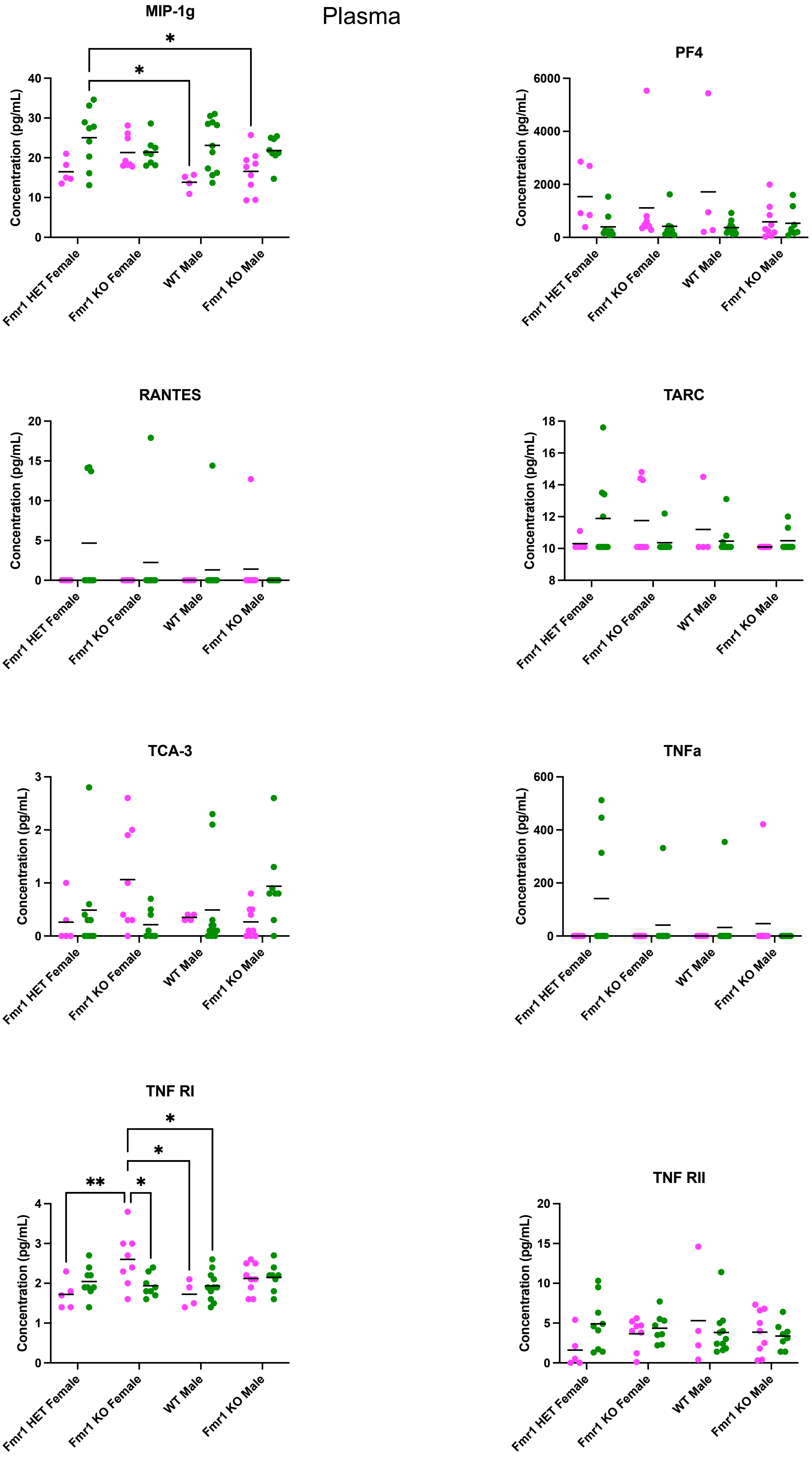
Plasma
MIP-1g
PF4
RANTES
TARC
TCA-3
TNFa
TNF RI
TNF RII
Concentration (pg/mL)
Fmr1 HET Female
Fmr1 KO Female
WT Male
Fmr1 KO Male
*
**
