# Supplementary material for: Soy Protein Isolate Affects Blood and Brain Biomarker Expression in a Mouse Model of Fragile X
Source: Int J Mol Sci. 2025 Jun 26;26(13):6137. doi: 10.3390/ijms26136137 (PMC12250412; doi:10.3390/ijms26136137)
Supplement: Supplementary file 1 [file ijms-26-06137-s001.zip › Supplementary File S4b Array 6 Graphs.pdf]

Supplementary File S4. Protein expression of Array 6 targets as function of *Fmr1* genotype and AIN-93G diets. Mice on AIN-93G/cas (colored pink) included n=5 $Fmr1^{HET}$ female, n=8 $Fmr1^{KO}$ female, n=4 WT male and n=9 $Fmr1^{KO}$ male. Mice on AIN-93G/soy (colored green) included n=9 $Fmr1^{HET}$ female, n=8 $Fmr1^{KO}$ female, n=11 WT male and n=8 $Fmr1^{KO}$ male. The average concentration in cortex, hippocampus, hypothalamus and plasma in pg/mL was plotted versus genotype. Statistics were determined by 2-way ANOVA and Tukey's multiple comparison tests denoted by $p < 0.05$ (*), $p < 0.01$ (**), $p < 0.001$ (***) and $p < 0.0001$ (****).

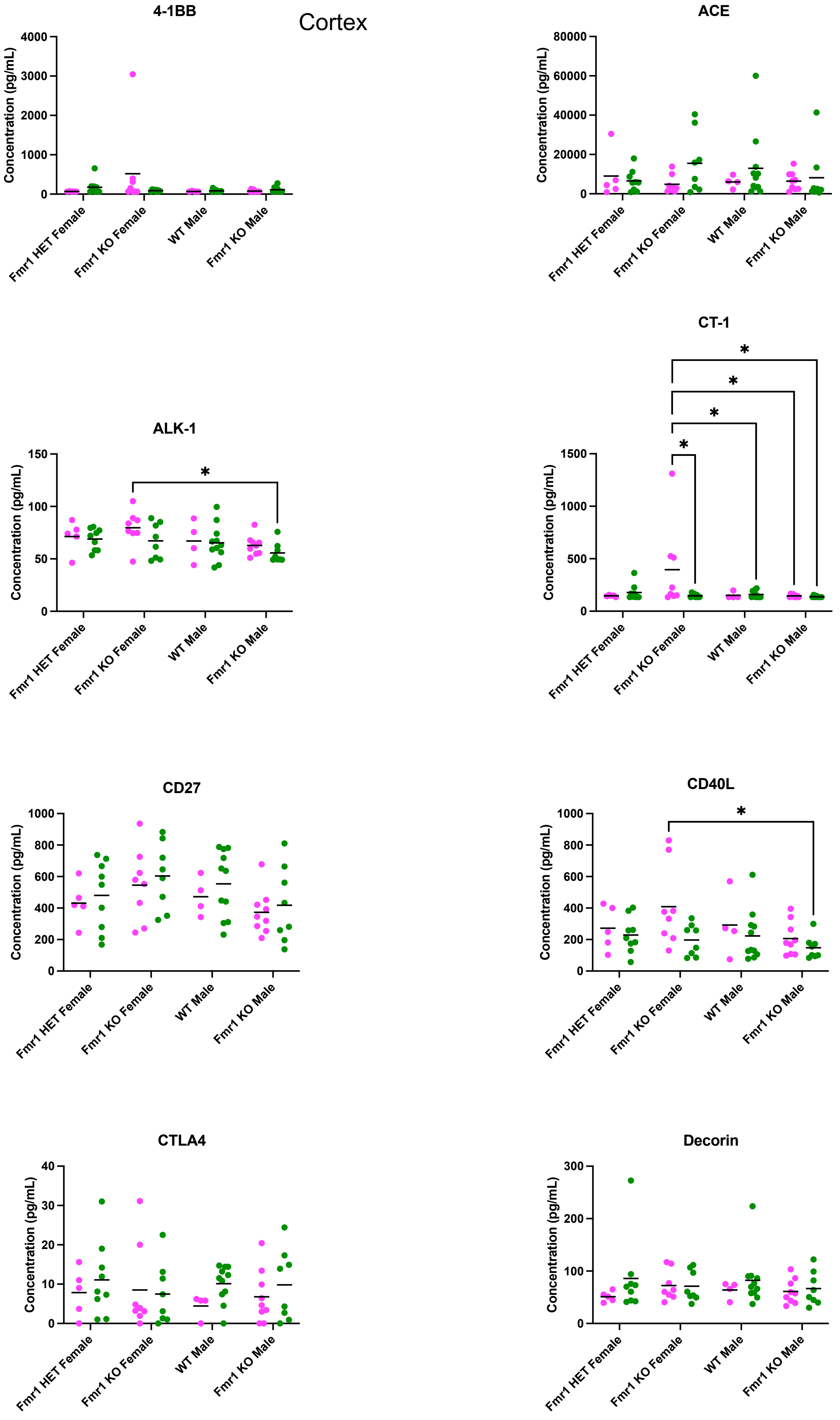
Cortex
4-1BB
ACE
ALK-1
CT-1
CD27
CD40L
CTLA4
Decorin
Concentration (pg/mL)
Fmr1 HET Female
Fmr1 KO Female
WT Male
Fmr1 KO Male
*

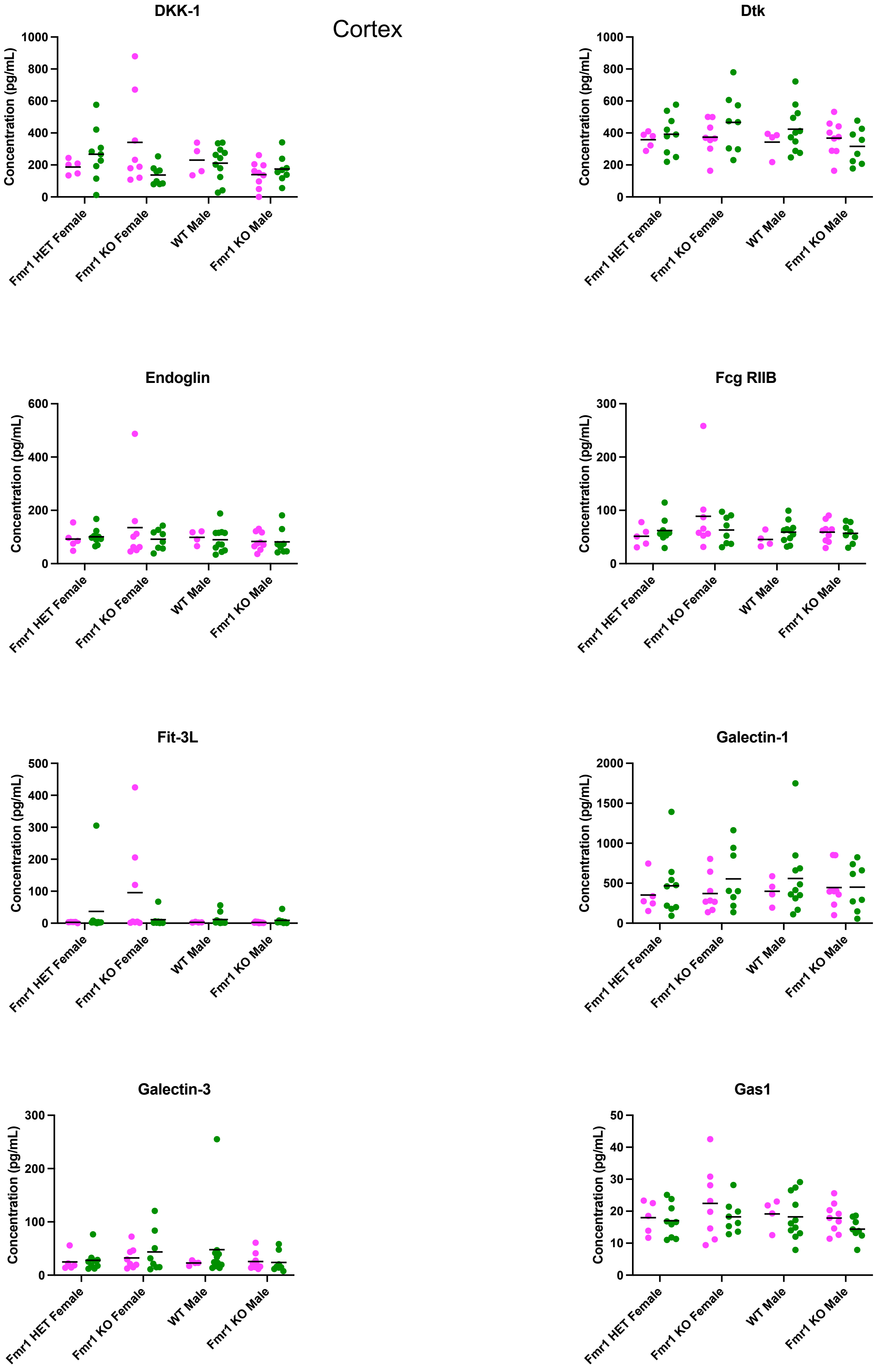
Cortex
DKK-1
Dtk
Endoglin
Fcg RIIB
Fit-3L
Galectin-1
Galectin-3
Gas1
Concentration (pg/mL)
Fmr1 HET Female
Fmr1 KO Female
WT Male
Fmr1 KO Male

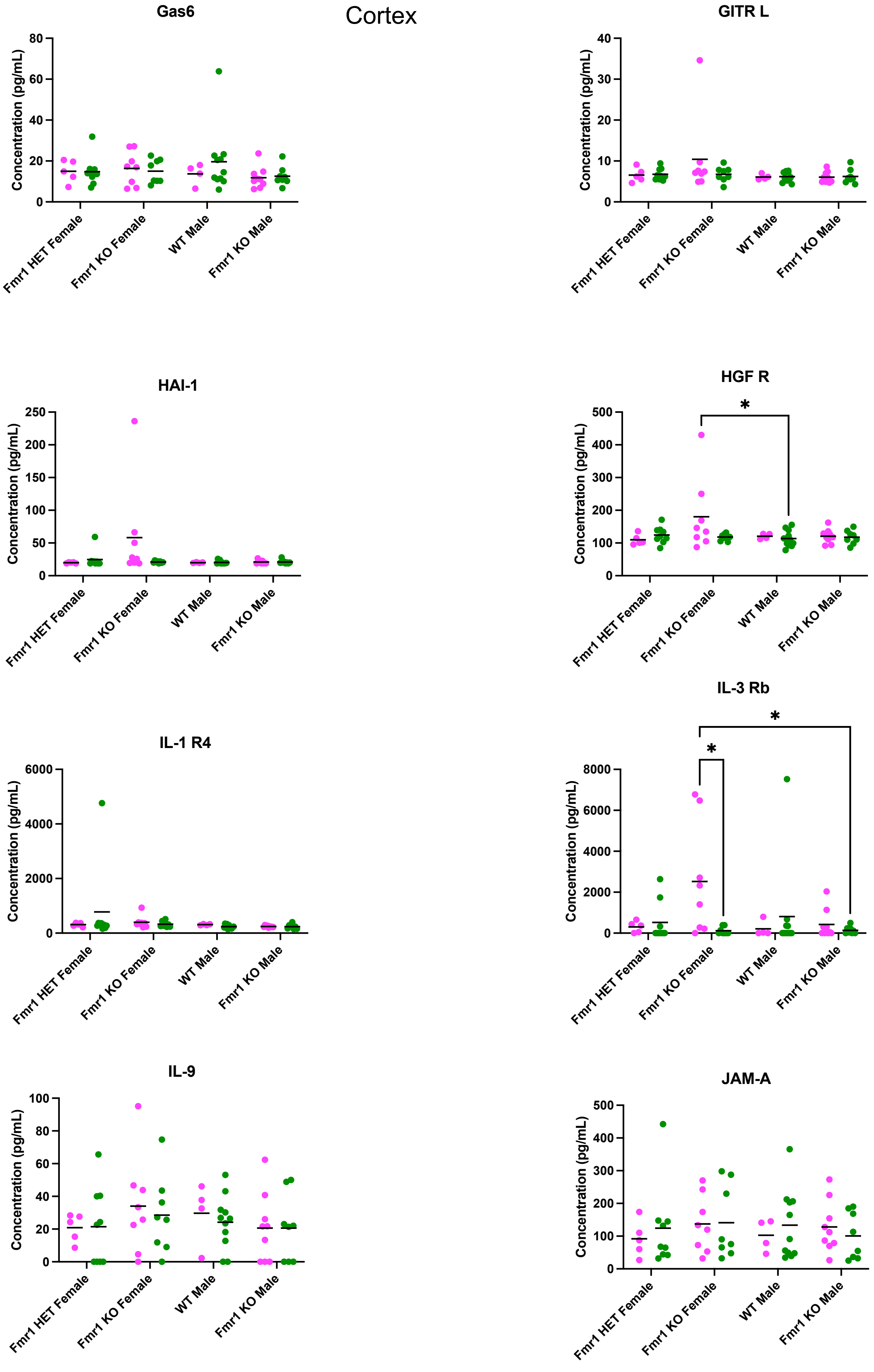
Cortex
Gas6
GITR L
HAI-1
HGF R
IL-1 R4
IL-3 Rb
IL-9
JAM-A
Concentration (pg/mL)
Fmr1 HET Female
Fmr1 KO Female
WT Male
Fmr1 KO Male
*

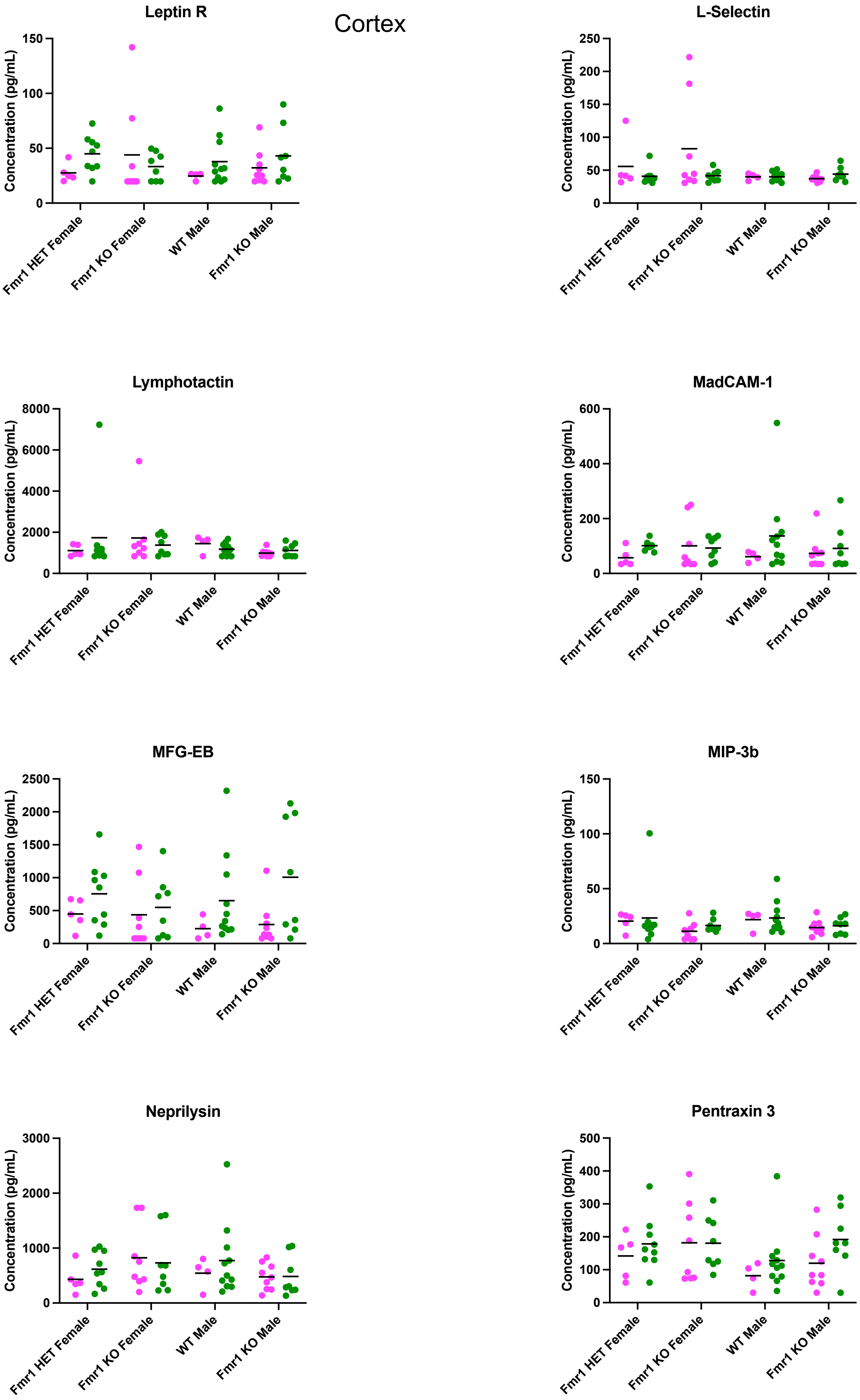

Cortex
Leptin R
L-Selectin
Lymphotactin
MadCAM-1
MFG-EB
MIP-3b
Neprilysin
Pentraxin 3
Concentration (pg/mL)
Fmr1 HET Female
Fmr1 KO Female
WT Male
Fmr1 KO Male

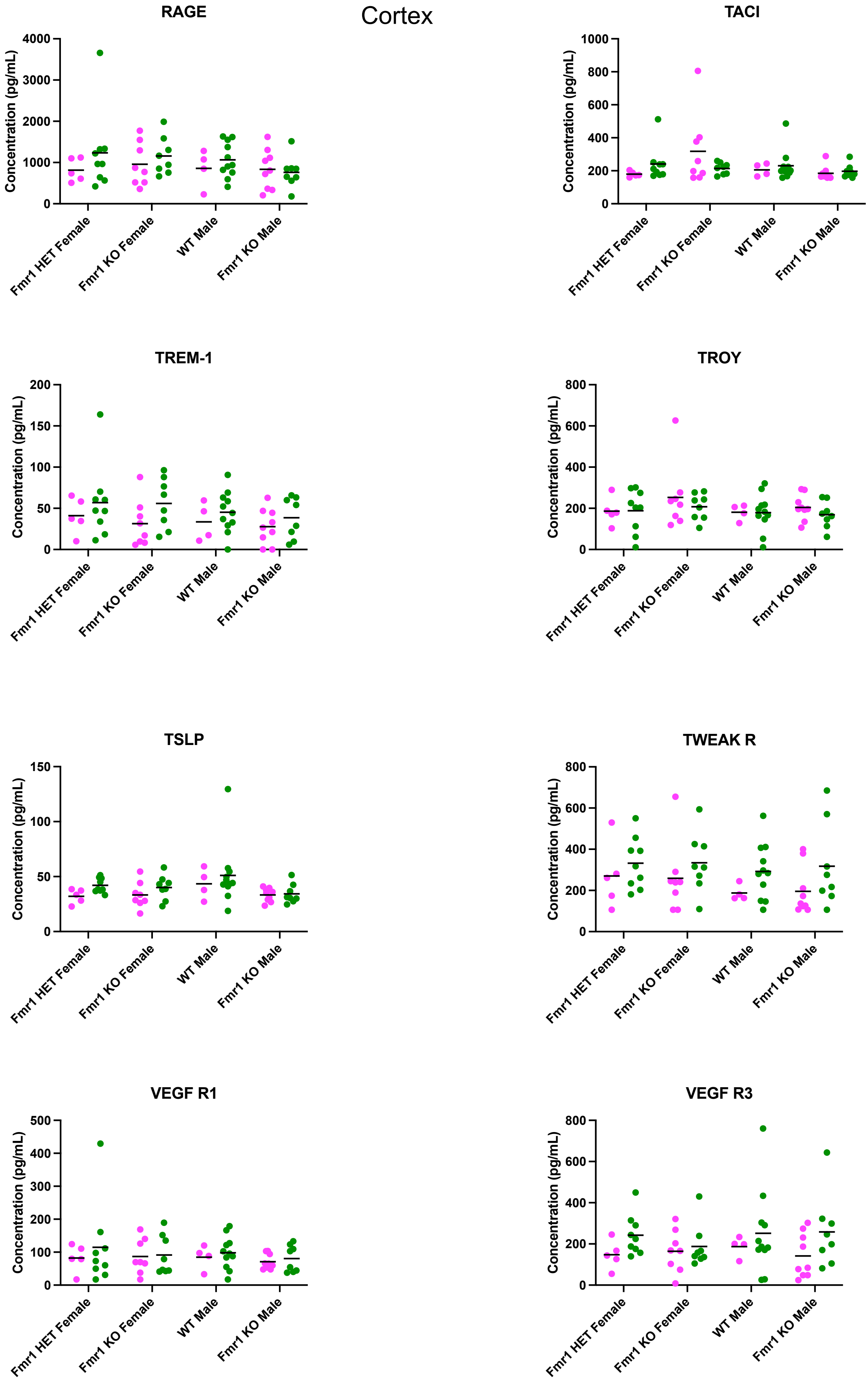
Cortex
RAGE
TACI
TREM-1
TROY
TSLP
TWEAK R
VEGF R1
VEGF R3
Concentration (pg/mL)
Fmr1 HET Female
Fmr1 KO Female
WT Male
Fmr1 KO Male

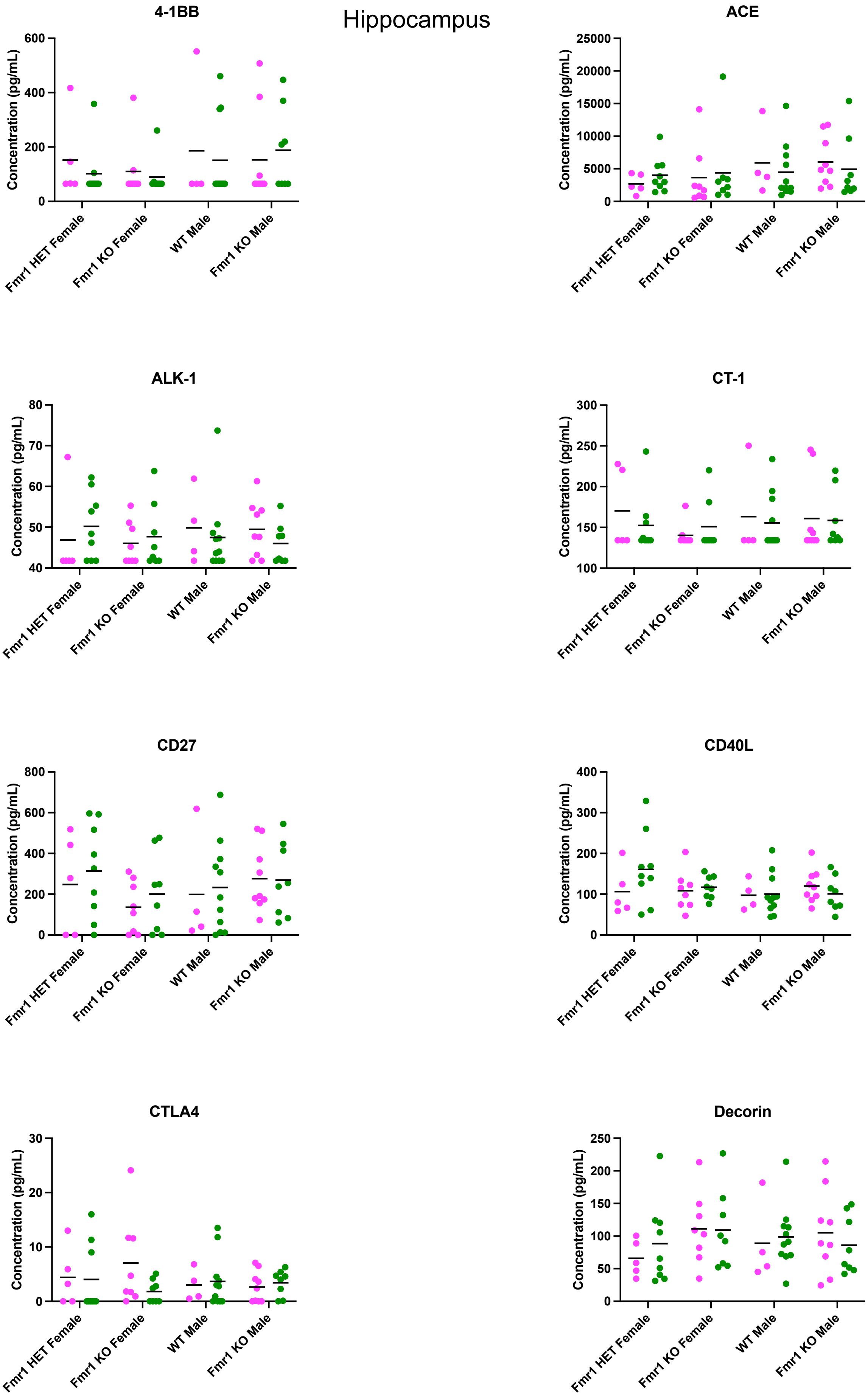
Hippocampus
4-1BB
ACE
ALK-1
CT-1
CD27
CD40L
CTLA4
Decorin
Concentration (pg/mL)
Fmr1 HET Female
Fmr1 KO Female
WT Male
Fmr1 KO Male

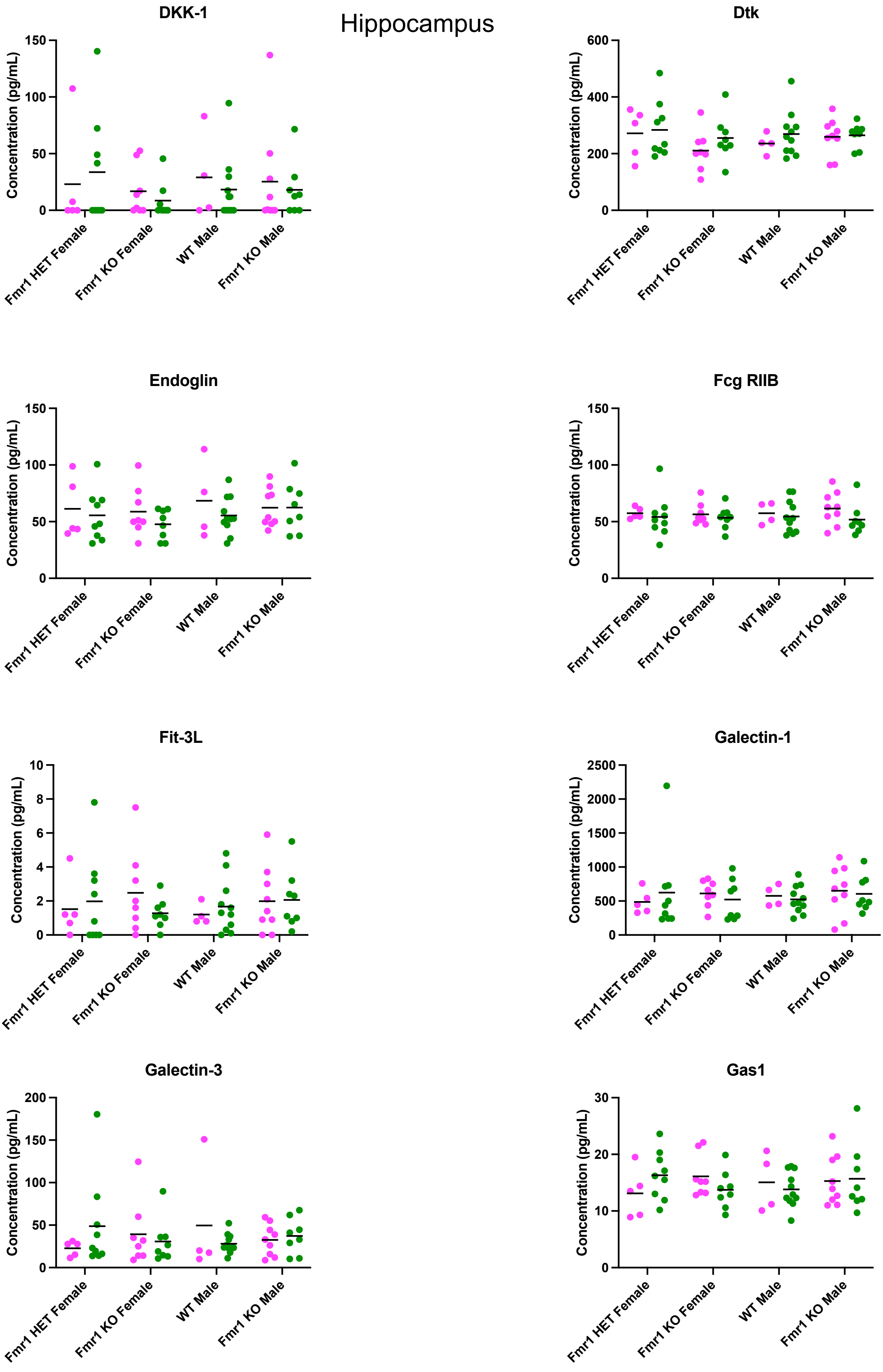
Hippocampus
DKK-1
Dtk
Endoglin
Fcg RIIB
Fit-3L
Galectin-1
Galectin-3
Gas1
Concentration (pg/mL)
0
50
100
150
200
400
600
2
4
6
8
10
500
1000
1500
2000
2500
200
10
20
30
Fmr1 HET Female
Fmr1 KO Female
WT Male
Fmr1 KO Male

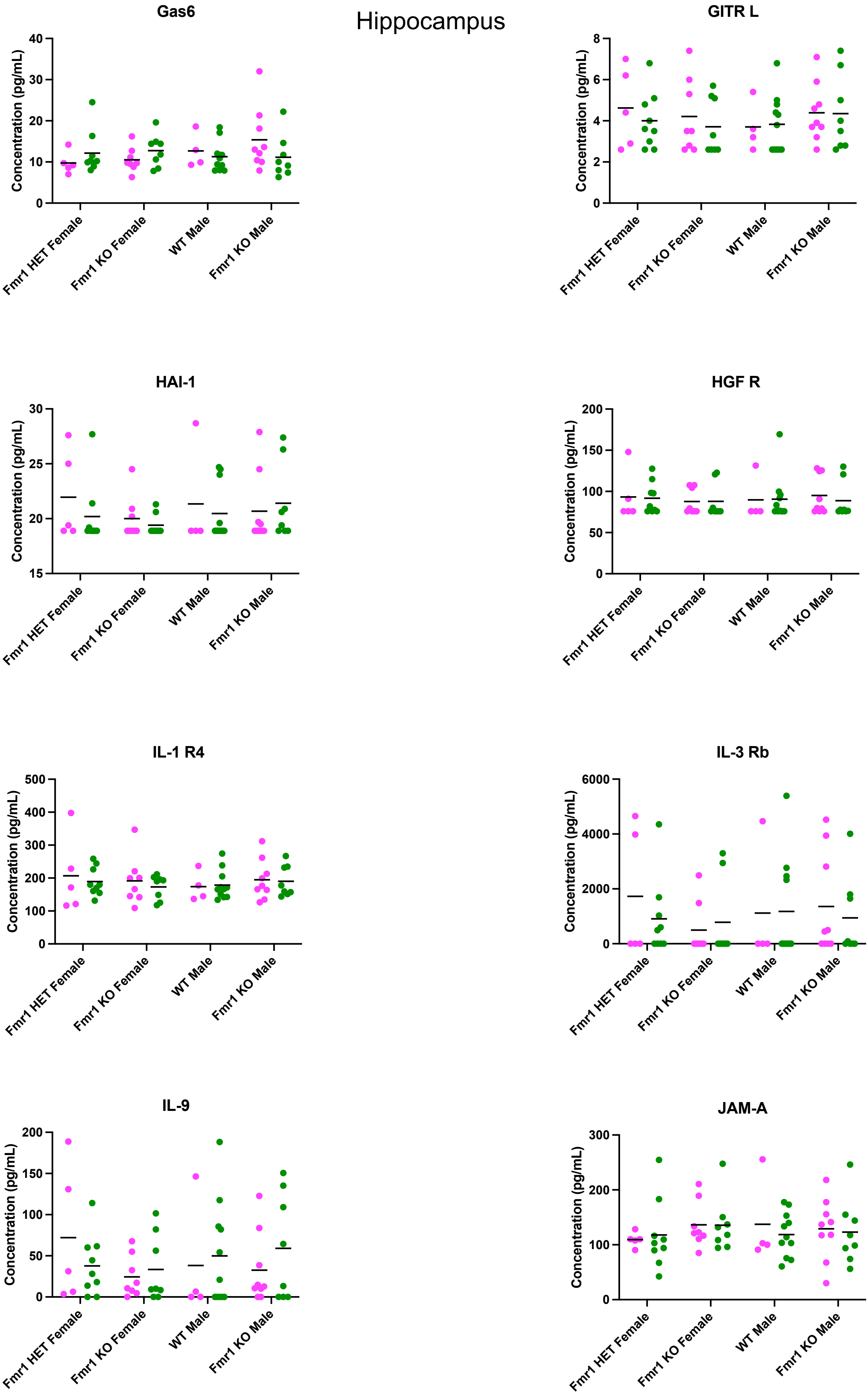
Hippocampus
Gas6
GITR L
HAI-1
HGF R
IL-1 R4
IL-3 Rb
IL-9
JAM-A
Concentration (pg/mL)
Fmr1 HET Female
Fmr1 KO Female
WT Male
Fmr1 KO Male

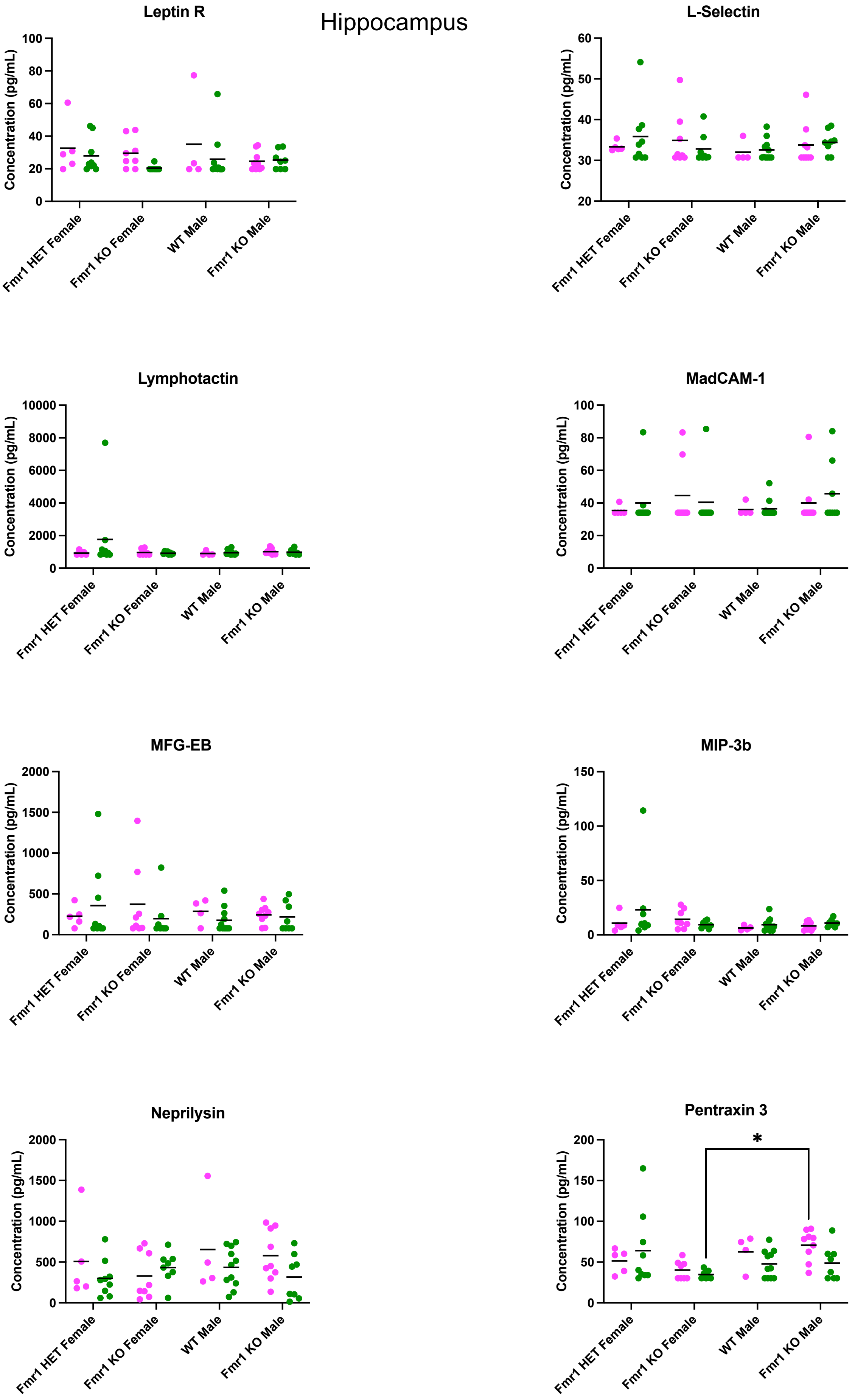
Hippocampus
Leptin R
L-Selectin
Lymphotactin
MadCAM-1
MFG-EB
MIP-3b
Neprilysin
Pentraxin 3
*
Concentration (pg/mL)
Fmr1 HET Female
Fmr1 KO Female
WT Male
Fmr1 KO Male

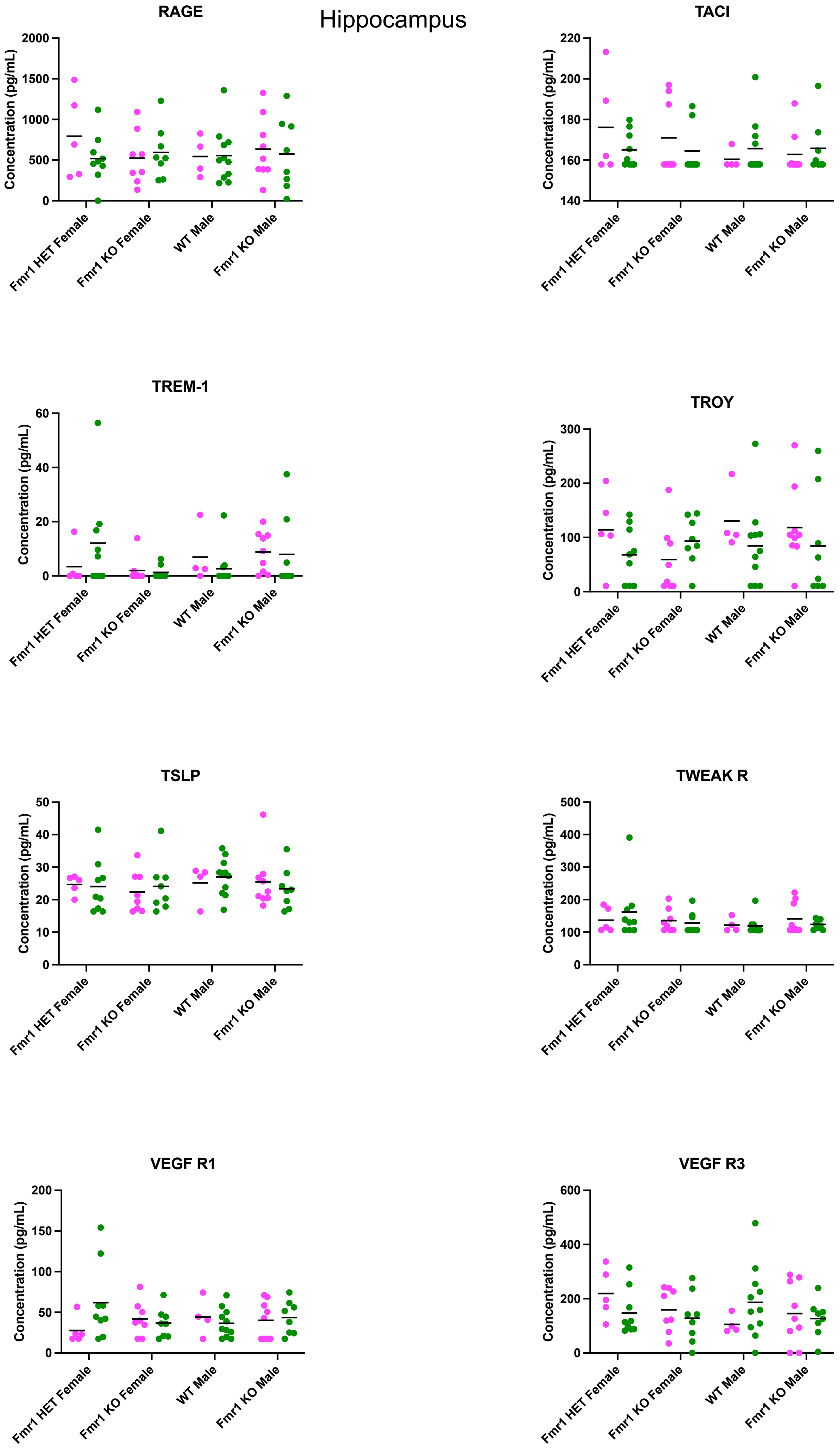
Hippocampus
RAGE
TACI
TREM-1
TROY
TSLP
TWEAK R
VEGF R1
VEGF R3
Concentration (pg/mL)
Fmr1 HET Female
Fmr1 KO Female
WT Male
Fmr1 KO Male

Hypothalamus

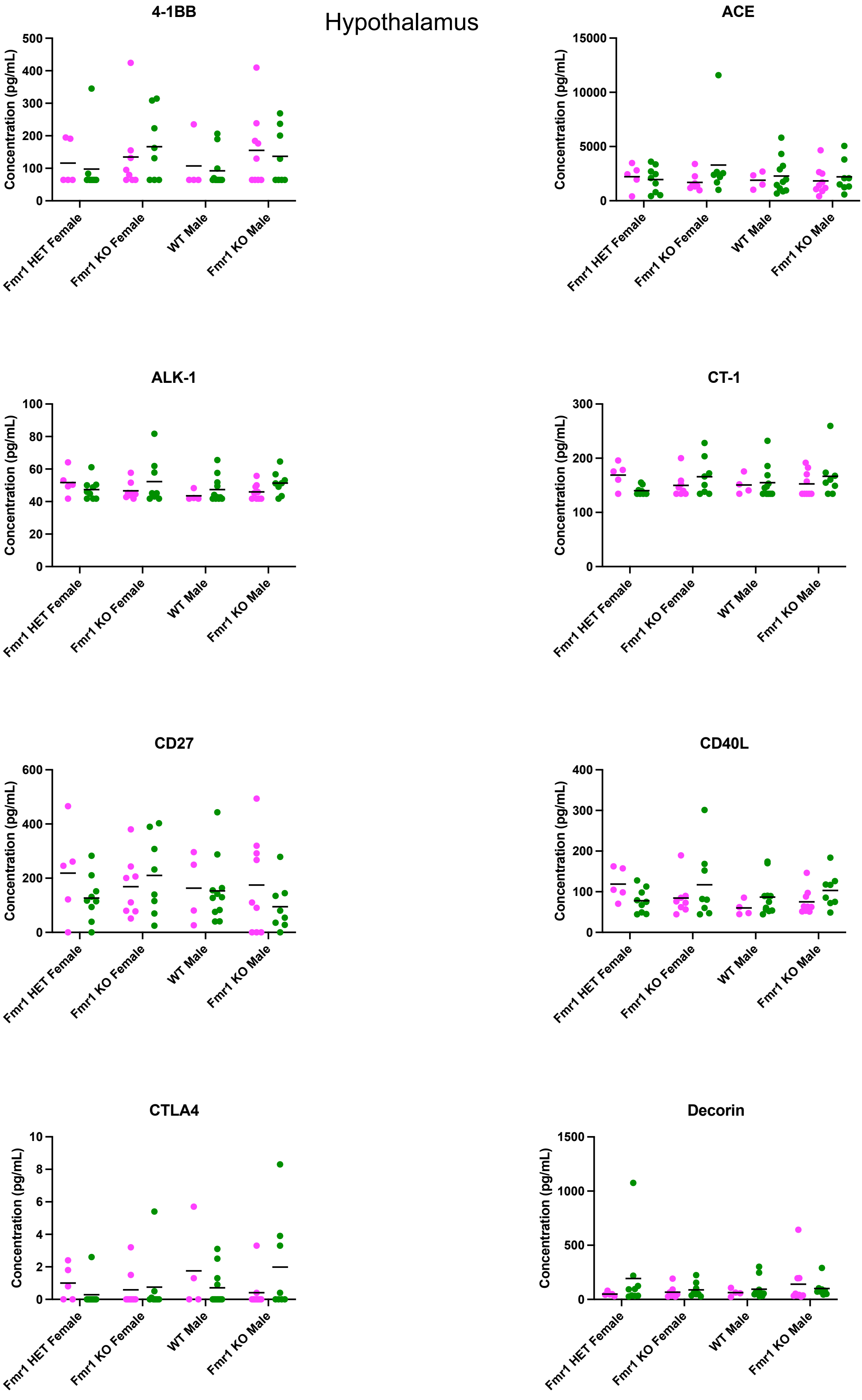

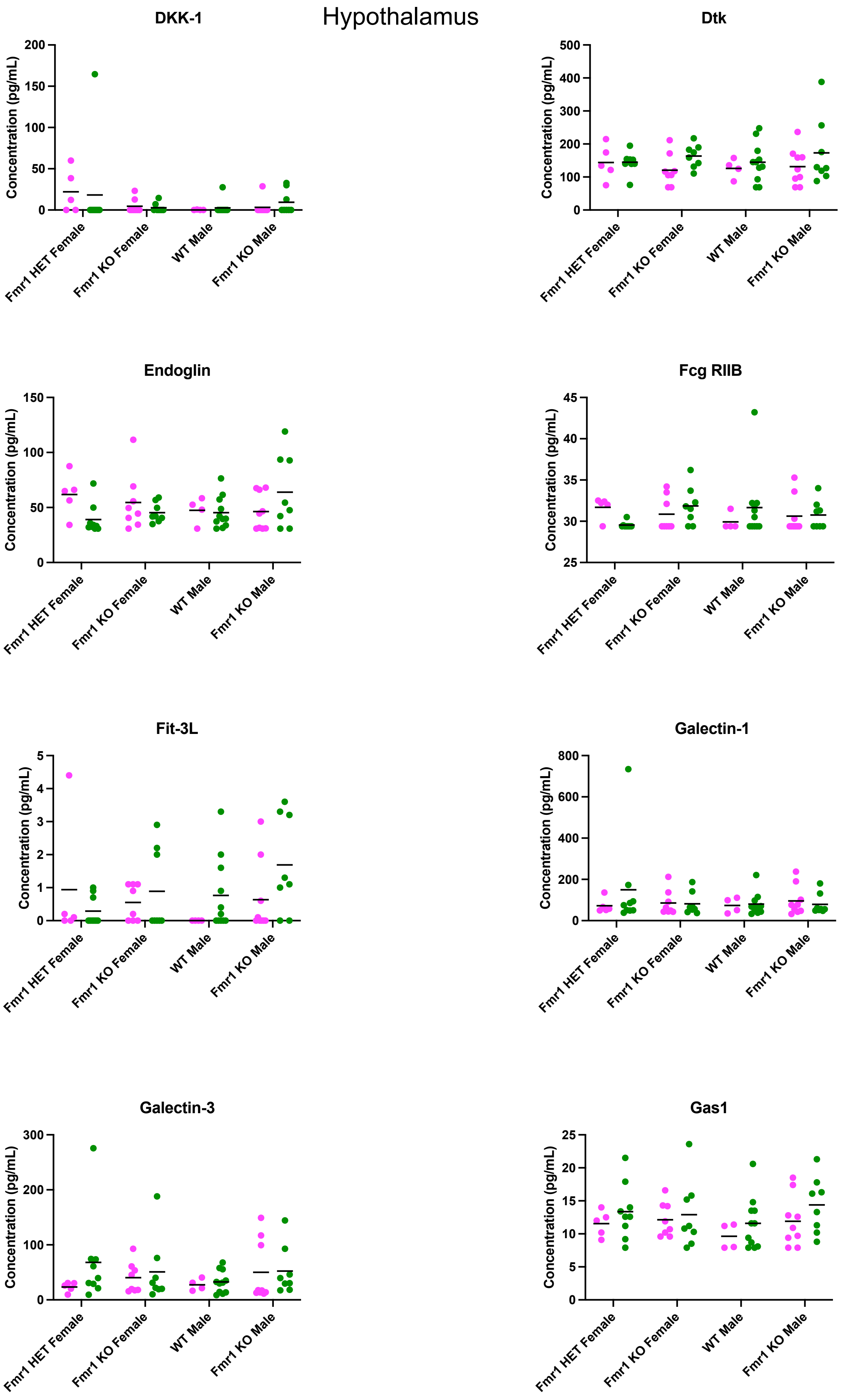

Hypothalamus
DKK-1
Dtk
Endoglin
Fcg RIIB
Fit-3L
Galectin-1
Galectin-3
Gas1
Concentration (pg/mL)
Fmr1 HET Female
Fmr1 KO Female
WT Male
Fmr1 KO Male

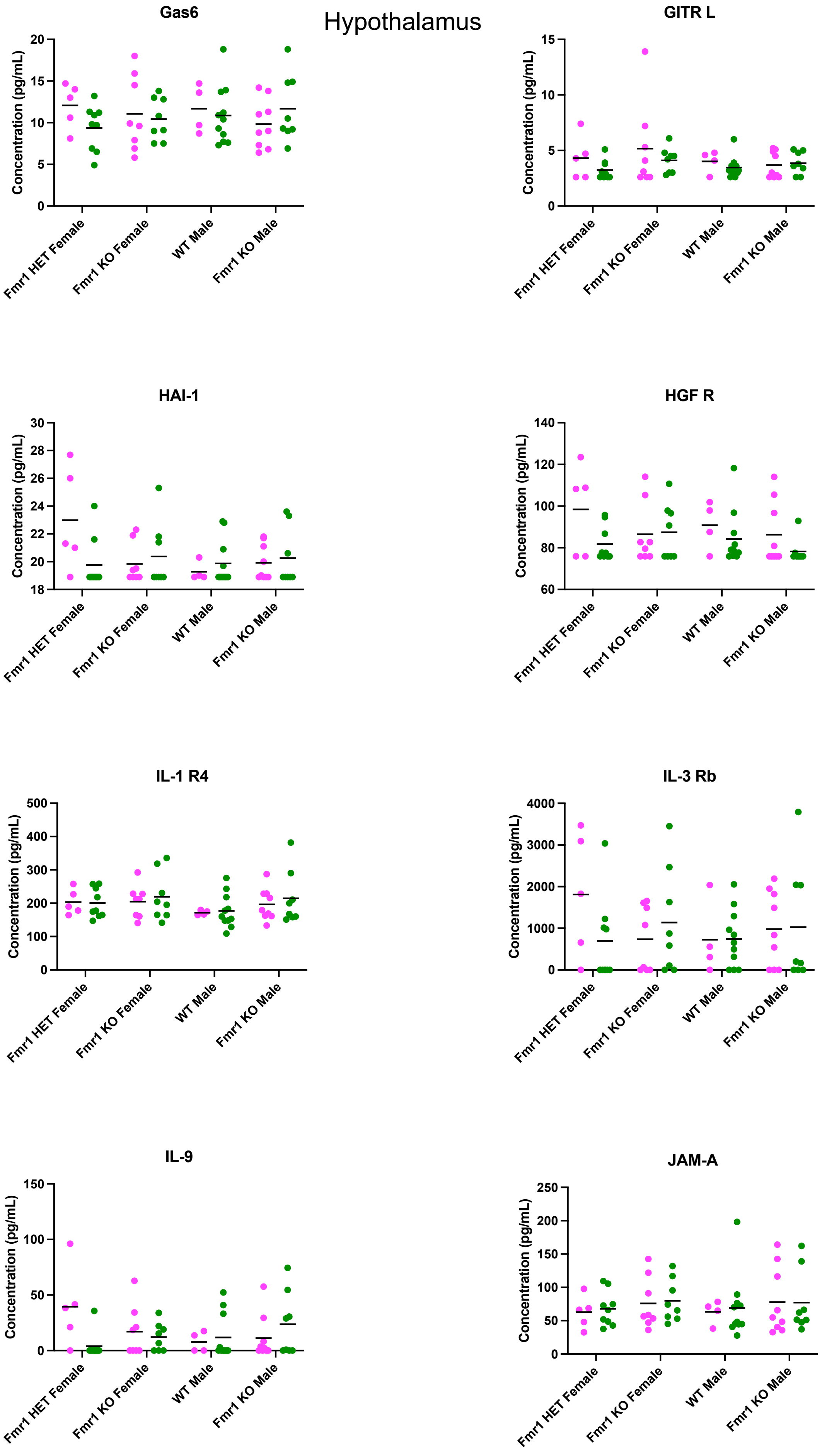
Hypothalamus
Gas6
GITR L
HAI-1
HGF R
IL-1 R4
IL-3 Rb
IL-9
JAM-A
Concentration (pg/mL)
0
5
10
15
20
18
22
24
26
28
30
60
80
100
120
140
200
300
400
500
1000
2000
3000
4000
50
150
250
Fmr1 HET Female
Fmr1 KO Female
WT Male
Fmr1 KO Male

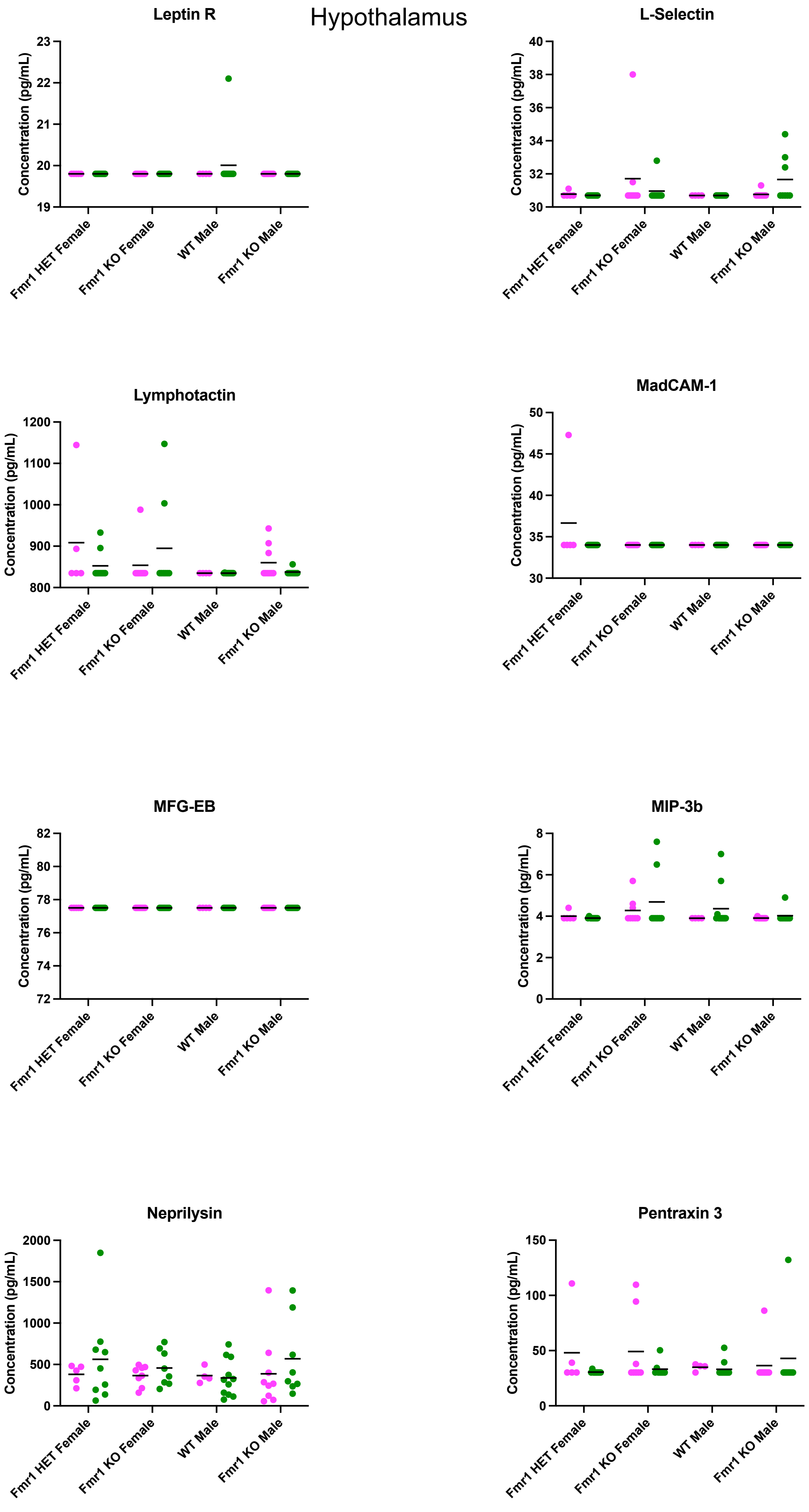
Hypothalamus
Leptin R
L-Selectin
Lymphotactin
MadCAM-1
MFG-EB
MIP-3b
Neprilysin
Pentraxin 3
Concentration (pg/mL)
Fmr1 HET Female
Fmr1 KO Female
WT Male
Fmr1 KO Male

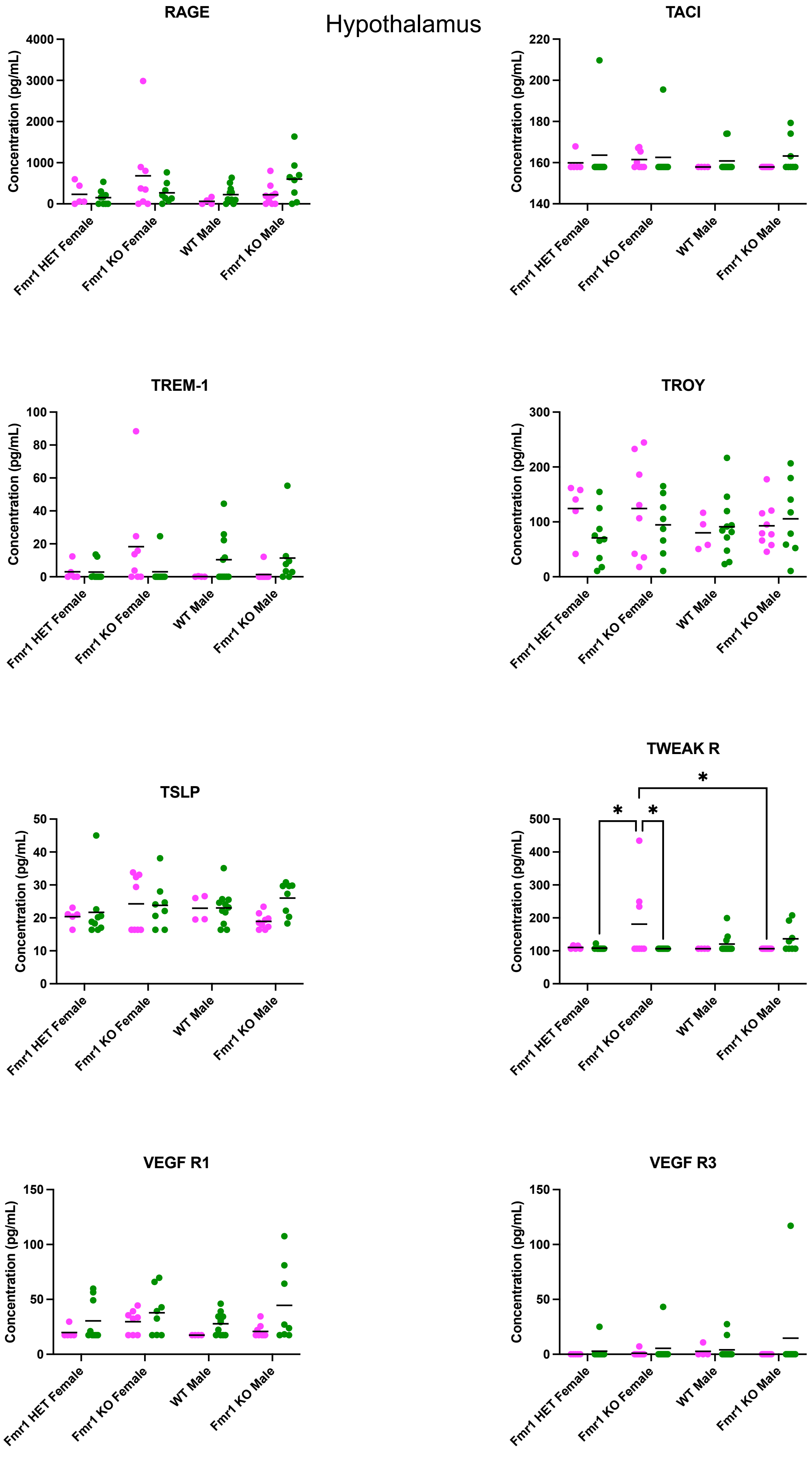
Hypothalamus
RAGE
TACI
TREM-1
TROY
TSLP
TWEAK R
VEGF R1
VEGF R3
Concentration (pg/mL)
Fmr1 HET Female
Fmr1 KO Female
WT Male
Fmr1 KO Male
*
*
*
0
1000
2000
3000
4000
140
160
180
200
220
20
40
60
80
100
300
10
30
50
400
500
150

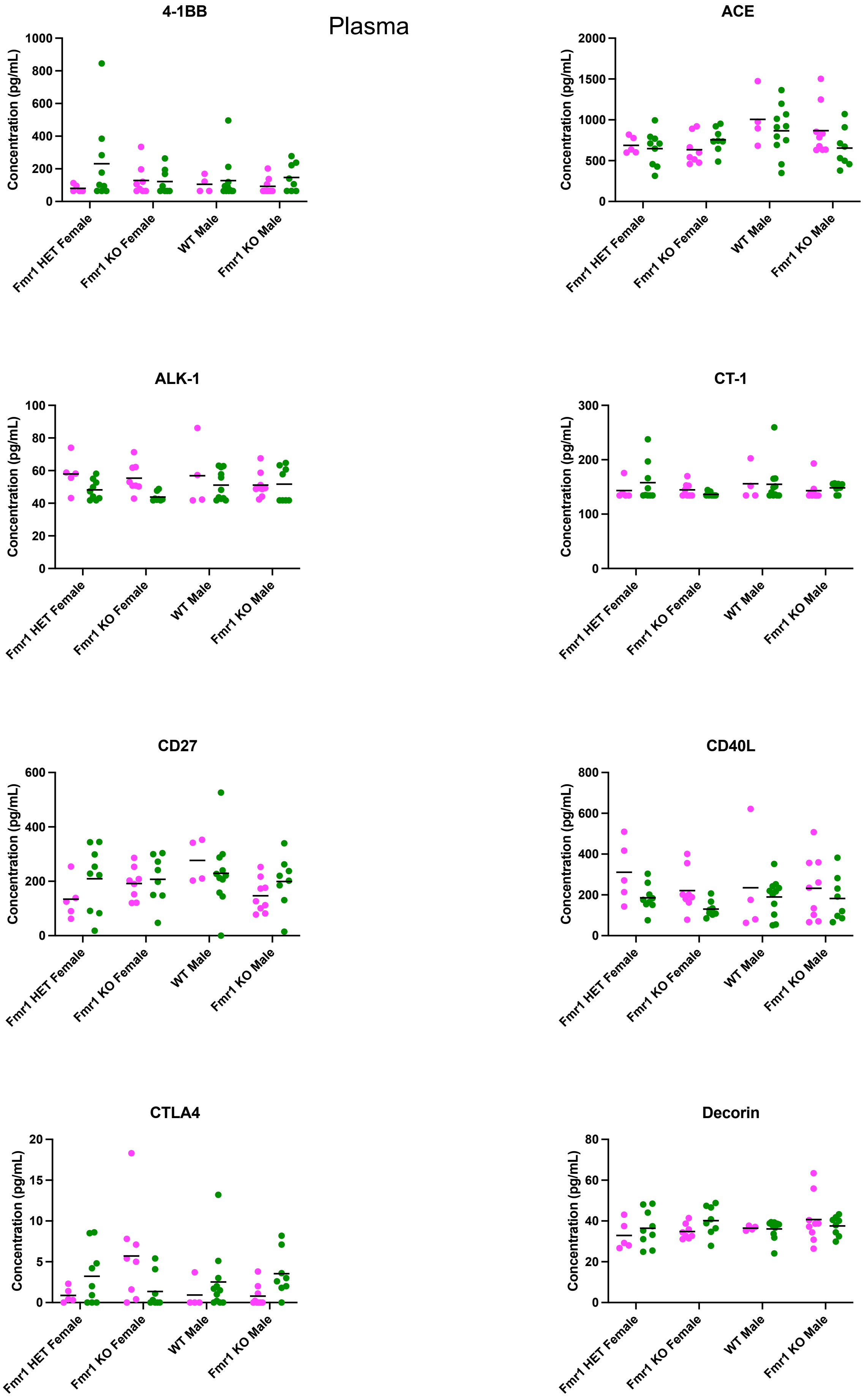
Plasma
4-1BB
ACE
ALK-1
CT-1
CD27
CD40L
CTLA4
Decorin
Concentration (pg/mL)
Fmr1 HET Female
Fmr1 KO Female
WT Male
Fmr1 KO Male
0
200
400
600
800
1000
500
1500
2000
20
40
60
80
100
300
5
10
15

Plasma

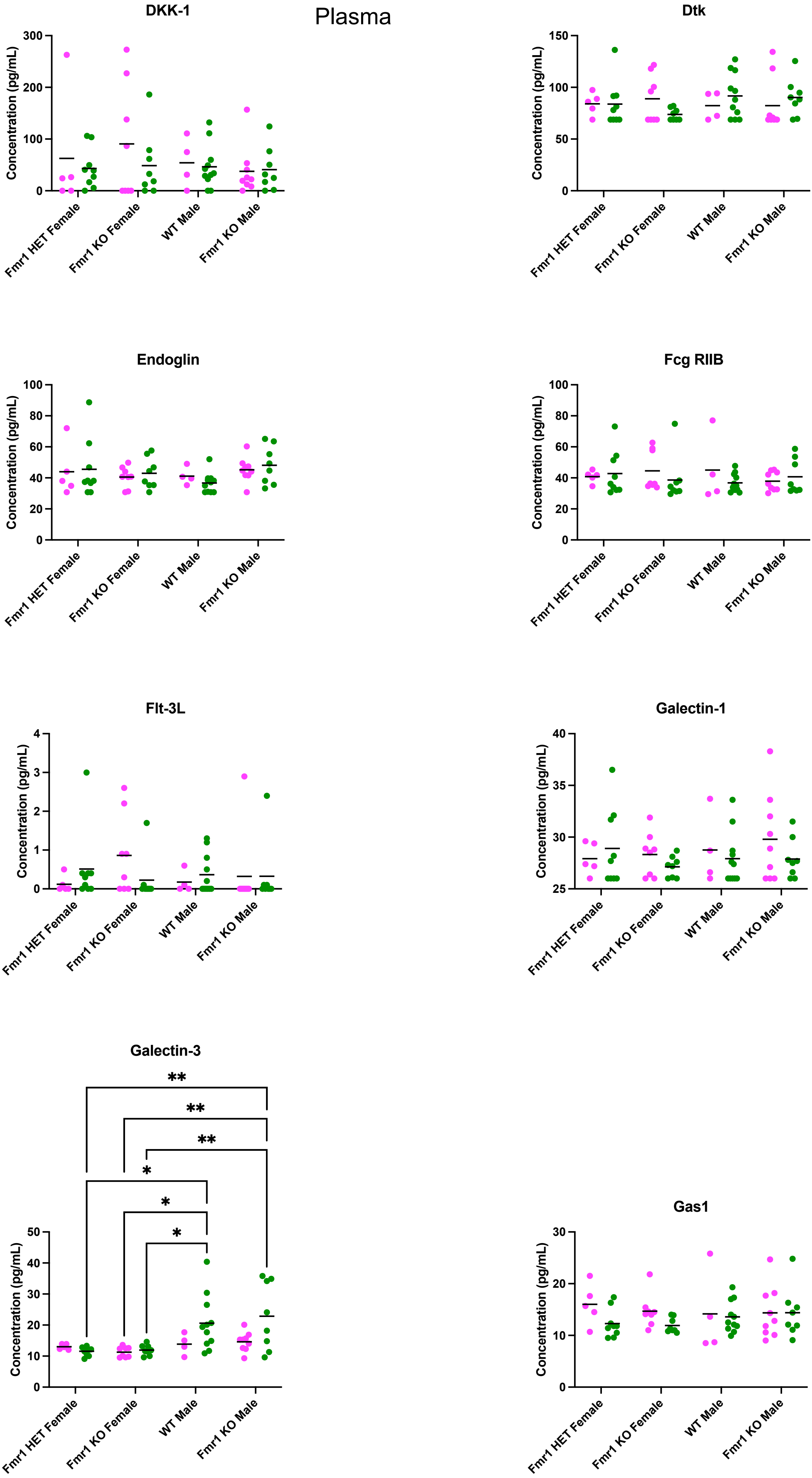
DKK-1
Dtk
Endoglin
Fcg RIIB
Flt-3L
Galectin-1
Galectin-3
Gas1
Concentration (pg/mL)
Fmr1 HET Female
Fmr1 KO Female
WT Male
Fmr1 KO Male
**
**
**
*
*
*

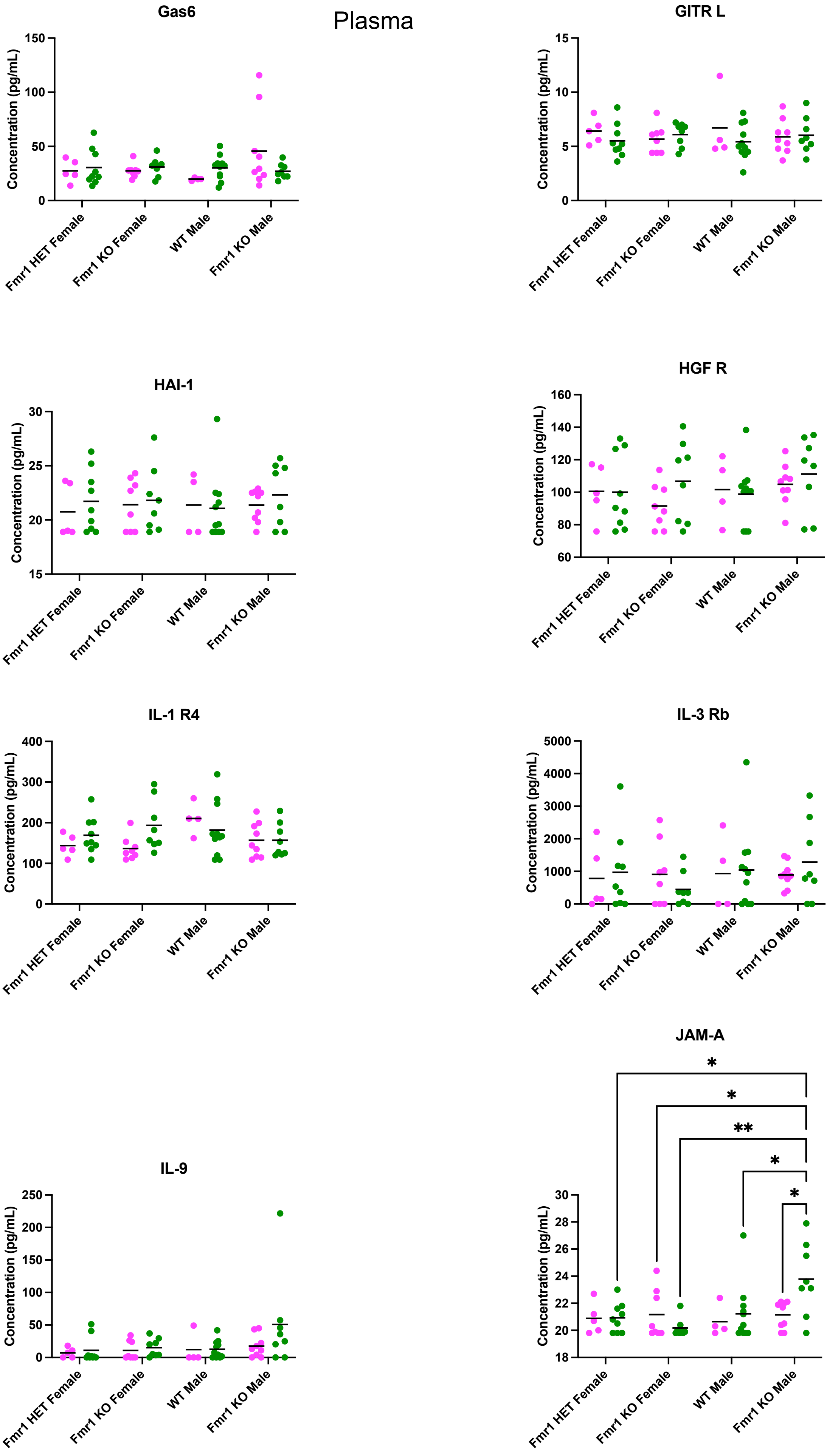

Plasma
Gas6
GITR L
HAI-1
HGF R
IL-1 R4
IL-3 Rb
IL-9
JAM-A
Concentration (pg/mL)
Fmr1 HET Female
Fmr1 KO Female
WT Male
Fmr1 KO Male
*
*
**
*
*

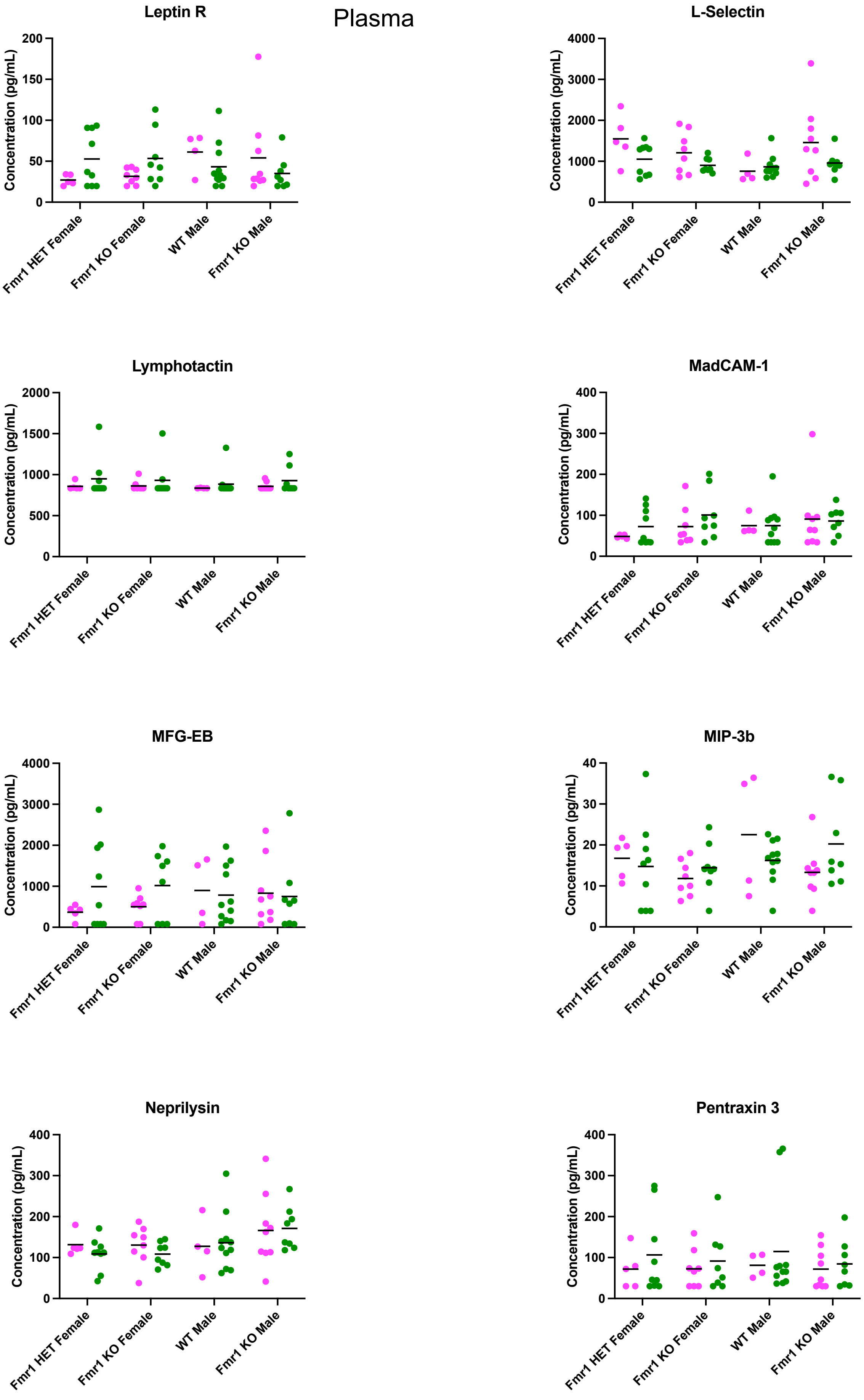
Plasma
Leptin R
L-Selectin
Lymphotactin
MadCAM-1
MFG-EB
MIP-3b
Neprilysin
Pentraxin 3
Concentration (pg/mL)
Fmr1 HET Female
Fmr1 KO Female
WT Male
Fmr1 KO Male

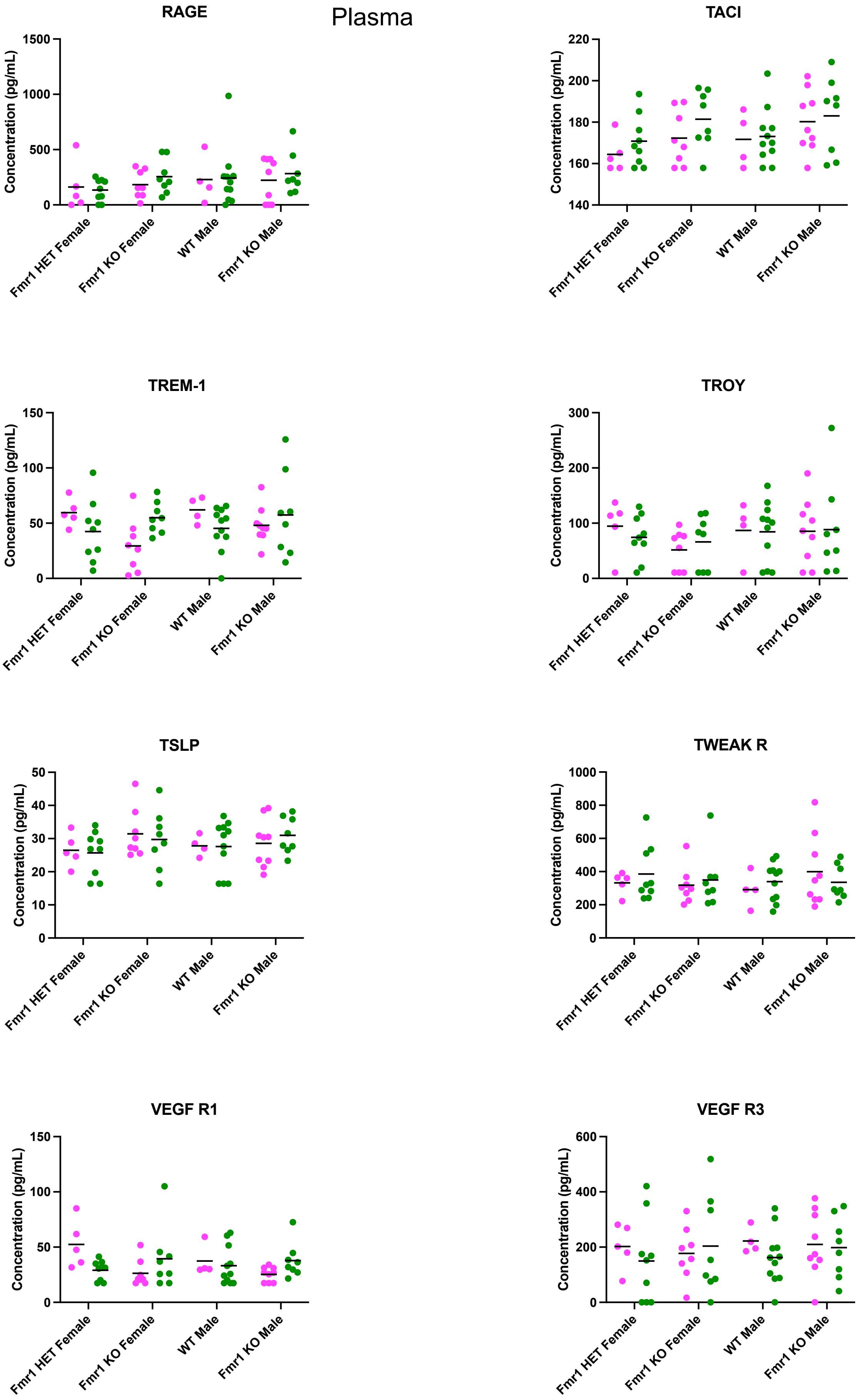
Plasma
RAGE
TACI
TREM-1
TROY
TSLP
TWEAK R
VEGF R1
VEGF R3
Concentration (pg/mL)
Fmr1 HET Female
Fmr1 KO Female
WT Male
Fmr1 KO Male
0
500
1000
1500
140
160
180
200
220
50
100
150
300
10
20
30
40
200
400
600
800
