# Supplementary material for: Soy Protein Isolate Affects Blood and Brain Biomarker Expression in a Mouse Model of Fragile X
Source: Int J Mol Sci. 2025 Jun 26;26(13):6137. doi: 10.3390/ijms26136137 (PMC12250412; doi:10.3390/ijms26136137)
Supplement: Supplementary file 1 [file ijms-26-06137-s001.zip › Supplementary File S5b Array 7 Graphs.pdf]

Supplementary File S5. Protein expression of Array 7 targets as function of *Fmr1* genotype and AIN-93G diets. Mice on AIN-93G/cas (colored pink) included n=5 *Fmr1*^{HET} female, n=8 *Fmr1*^{KO} female, n=4 WT male and n=9 *Fmr1*^{KO} male. Mice on AIN-93G/soy (colored green) included n=9 *Fmr1*^{HET} female, n=8 *Fmr1*^{KO} female, n=11 WT male and n=8 *Fmr1*^{KO} male. The average concentration in cortex, hippocampus, hypothalamus and plasma in pg/mL was plotted versus genotype. Statistics were determined by 2-way ANOVA and Tukey's multiple comparison tests denoted by $p < 0.05$ (*), $p < 0.01$ (**), $p < 0.001$ (***) and $p < 0.0001$ (****).

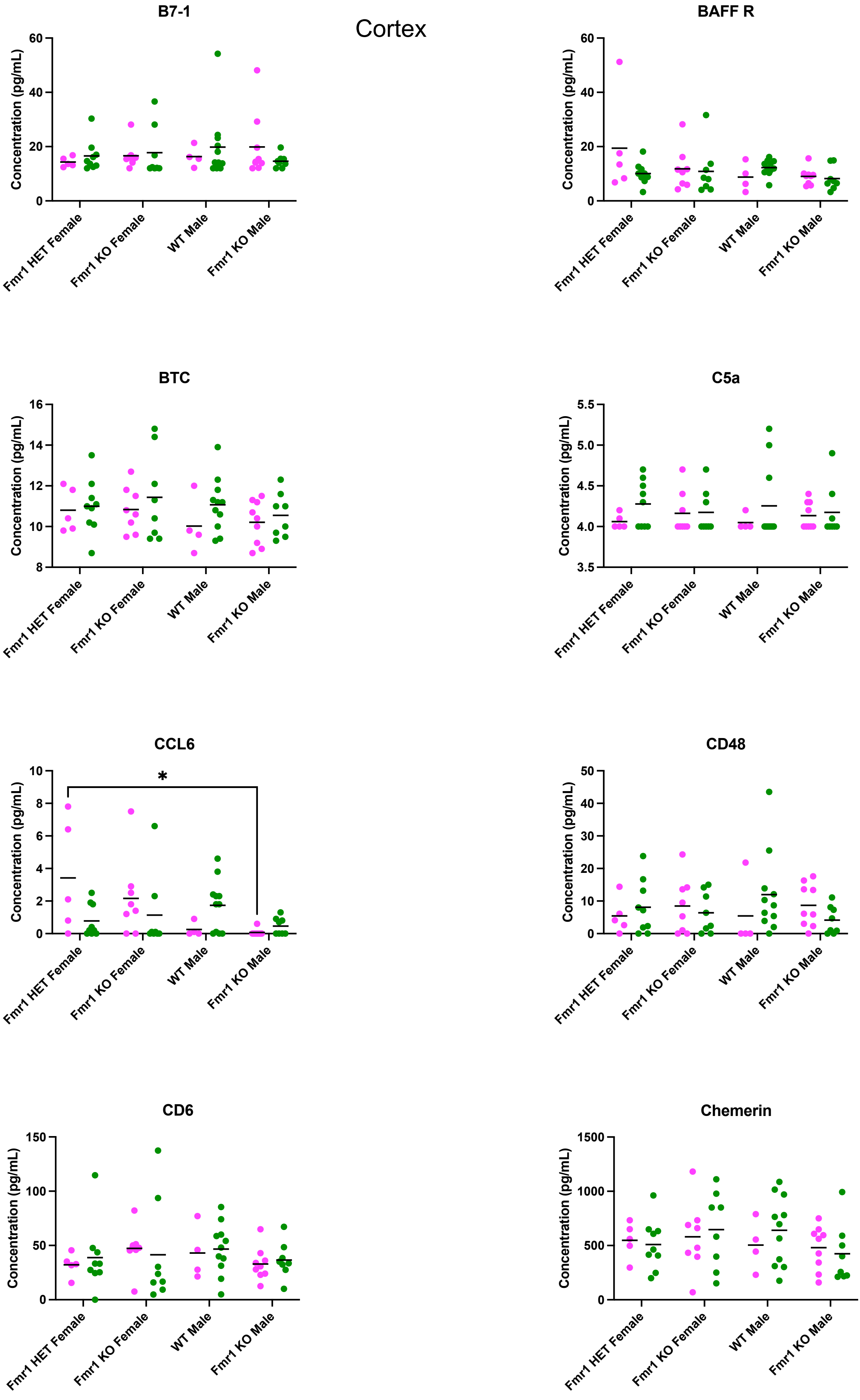
Cortex
B7-1
BAFF R
BTC
C5a
CCL6
*
CD48
CD6
Chemerin
Concentration (pg/mL)
Fmr1 HET Female
Fmr1 KO Female
WT Male
Fmr1 KO Male

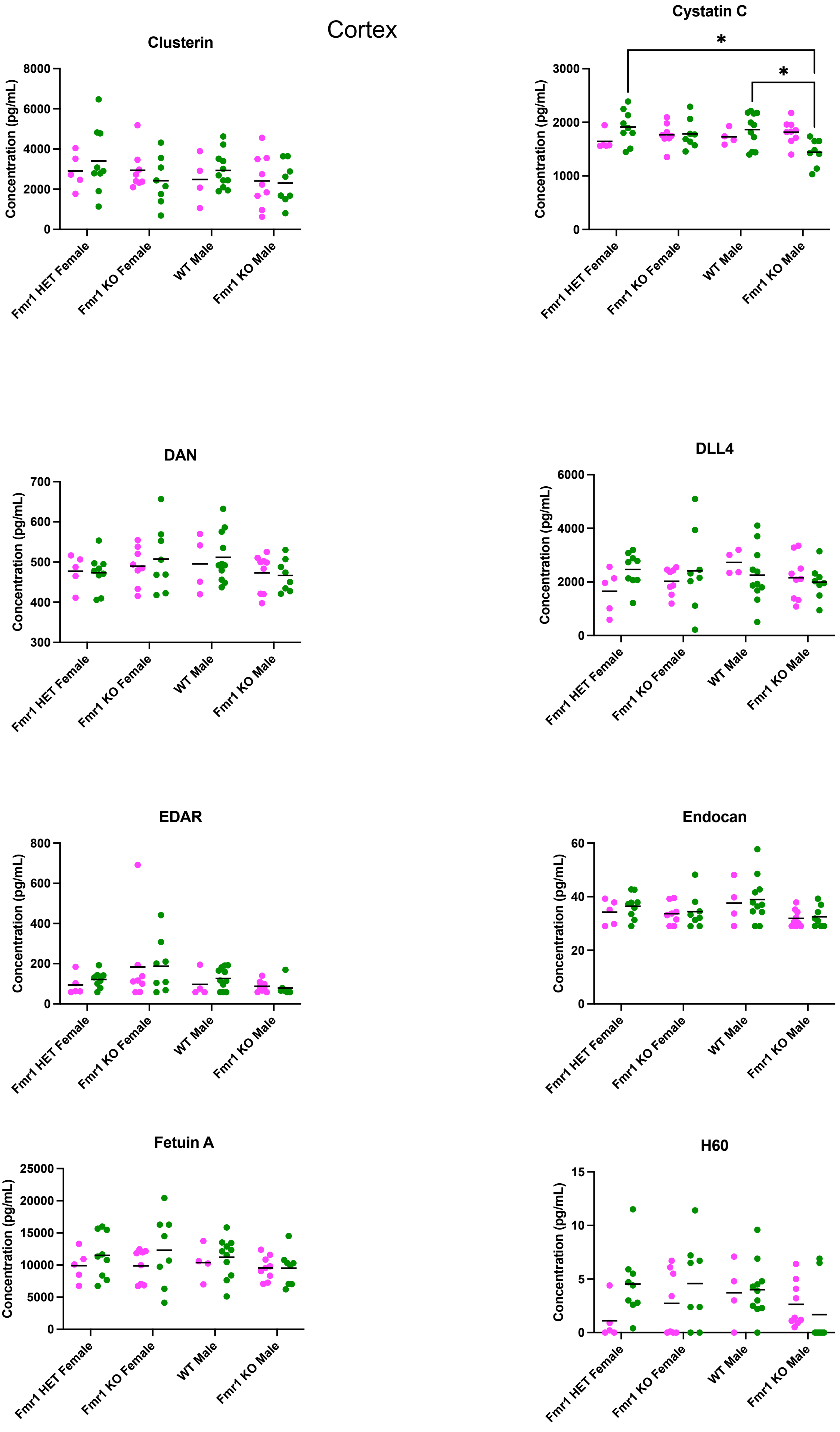
Cortex
Clusterin
Cystatin C
*
*
DAN
DLL4
EDAR
Endocan
Fetuin A
H60
Concentration (pg/mL)
8000
6000
4000
2000
0
3000
2000
1000
0
700
600
500
400
300
6000
4000
2000
0
800
600
400
200
0
60
40
20
0
25000
20000
15000
10000
5000
0
15
10
5
0
Fmr1 HET Female
Fmr1 KO Female
WT Male
Fmr1 KO Male

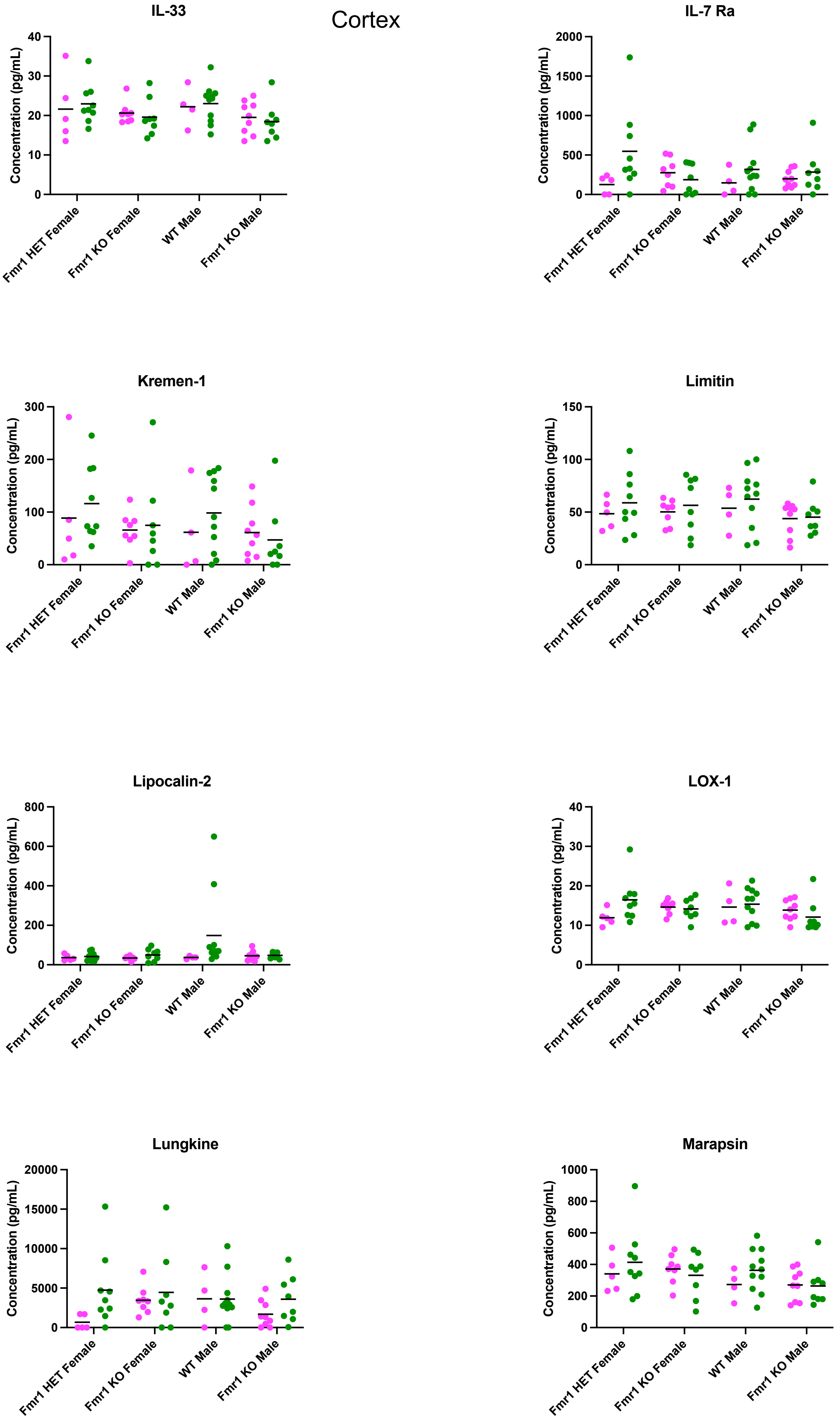

Cortex
IL-33
IL-7 Ra
Kremen-1
Limitin
Lipocalin-2
LOX-1
Lungkine
Marapsin
Concentration (pg/mL)
Fmr1 HET Female
Fmr1 KO Female
WT Male
Fmr1 KO Male

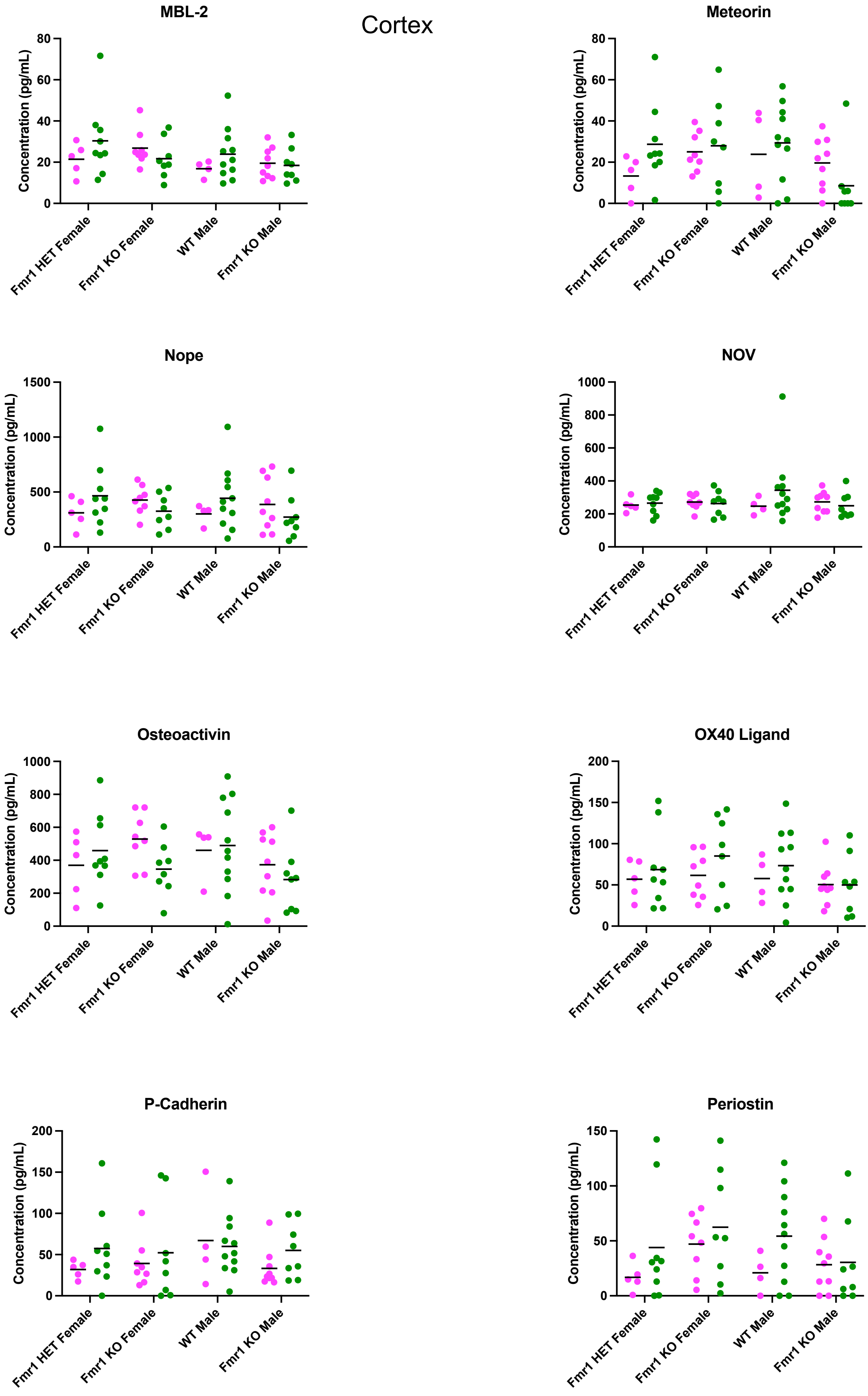
Cortex
MBL-2
Meteorin
Nope
NOV
Osteoactivin
OX40 Ligand
P-Cadherin
Periostin
Concentration (pg/mL)
Fmr1 HET Female
Fmr1 KO Female
WT Male
Fmr1 KO Male
0
20
40
60
80
500
1000
1500
200
400
600
800
1000
50
100
150
200

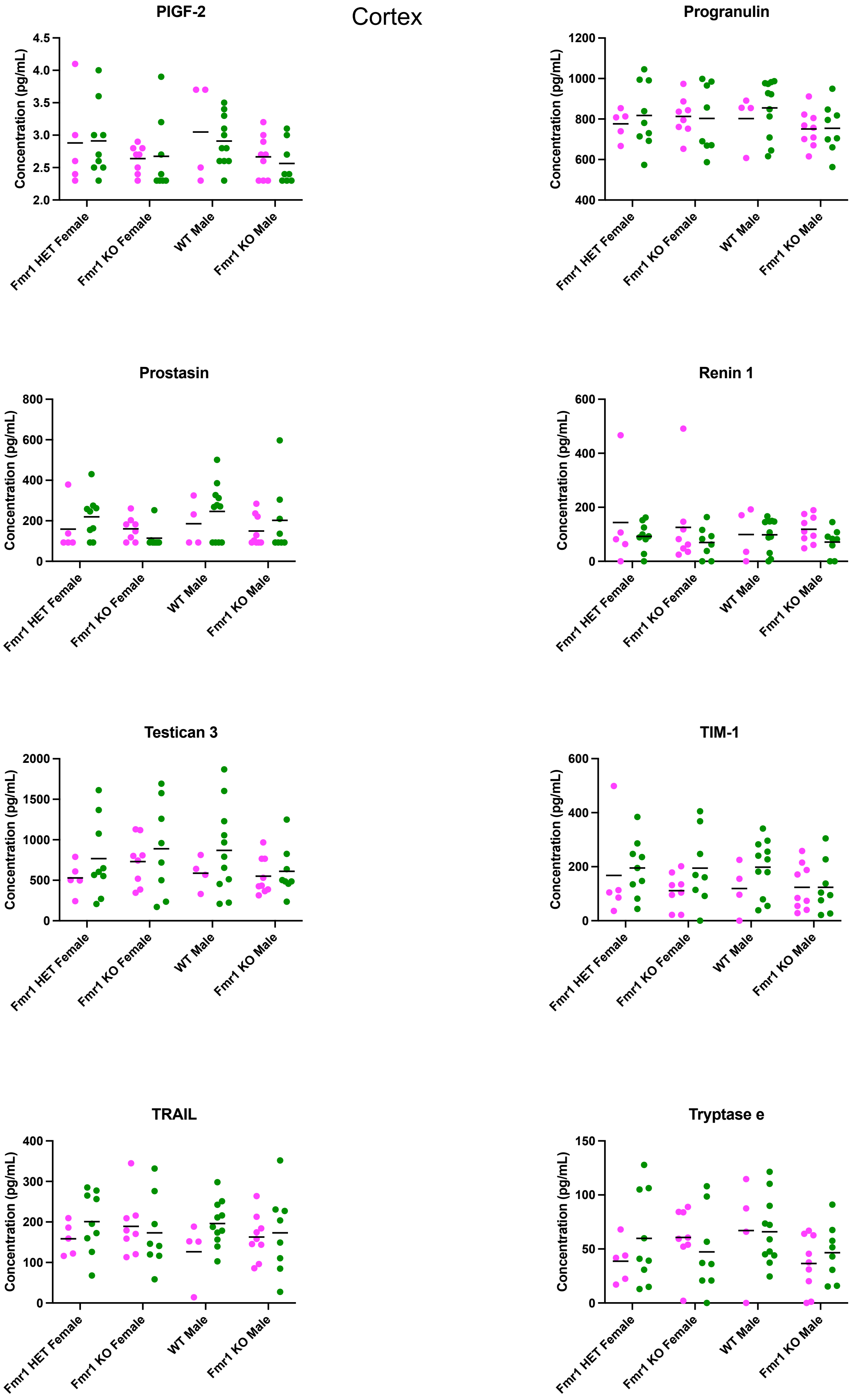
Cortex
PlGF-2
Progranulin
Prostasin
Renin 1
Testican 3
TIM-1
TRAIL
Tryptase e
Concentration (pg/mL)
Fmr1 HET Female
Fmr1 KO Female
WT Male
Fmr1 KO Male
2.0
2.5
3.0
3.5
4.0
4.5
400
600
800
1000
1200
0
200
400
600
800
0
200
400
600
0
500
1000
1500
2000
0
200
400
600
0
100
200
300
400
0
50
100
150

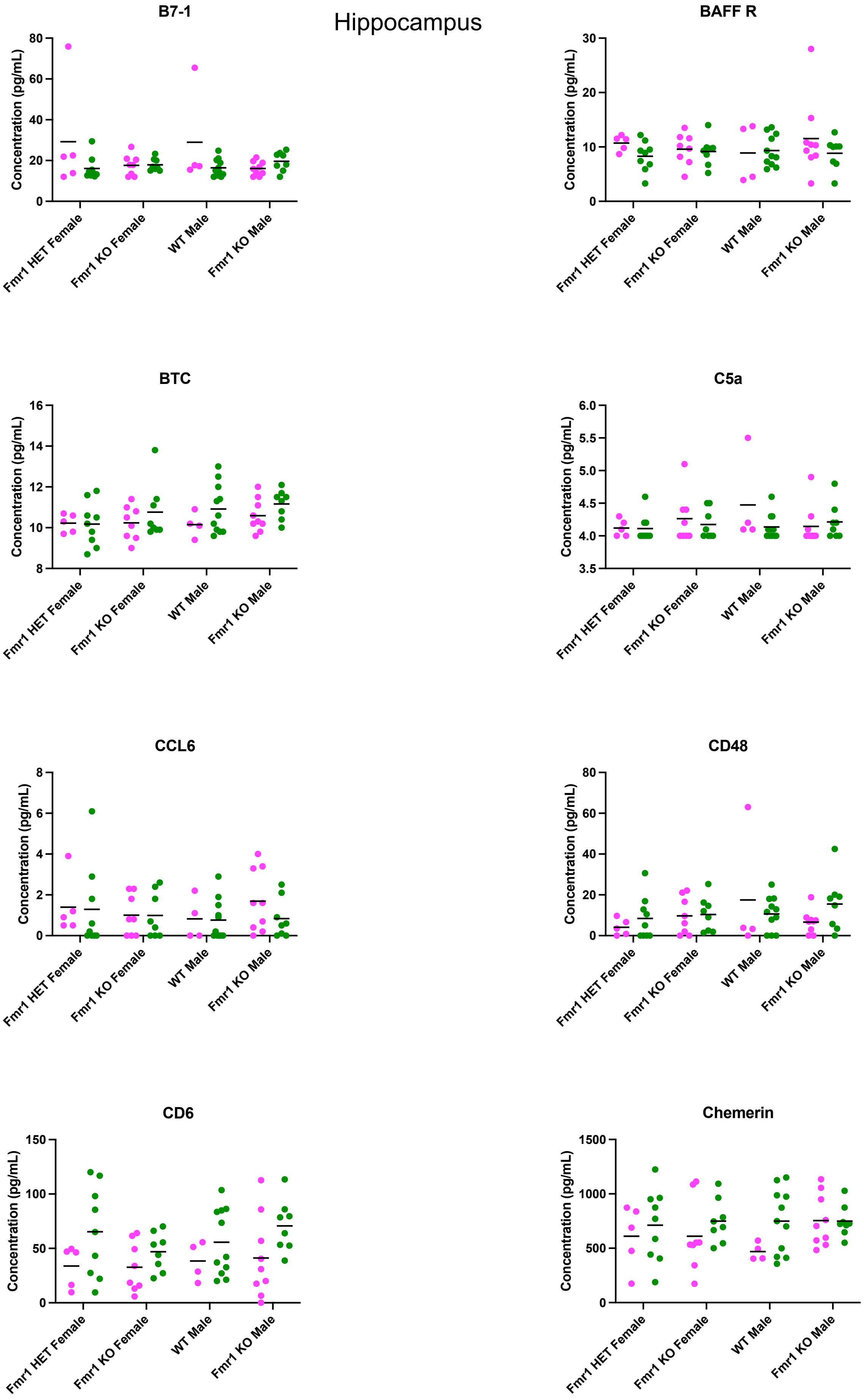
Hippocampus
B7-1
BAFF R
BTC
C5a
CCL6
CD48
CD6
Chemerin
Concentration (pg/mL)
0
20
40
60
80
10
30
8
12
14
16
3.5
4.0
4.5
5.0
5.5
6.0
2
4
6
50
100
150
500
1000
1500
Fmr1 HET Female
Fmr1 KO Female
WT Male
Fmr1 KO Male

Hippocampus

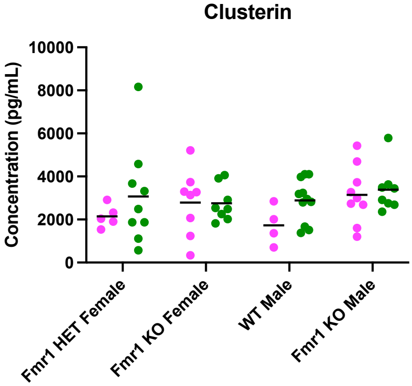

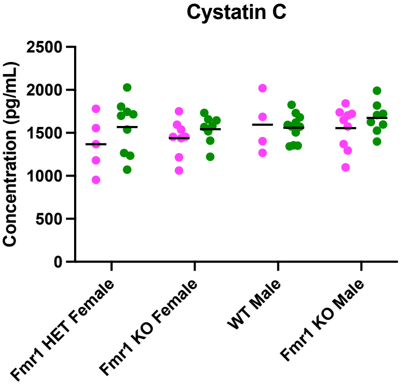

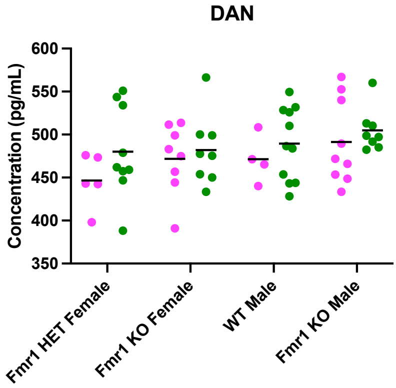

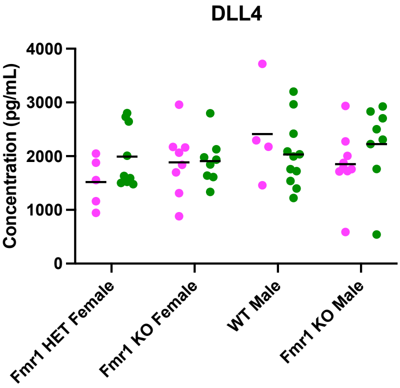

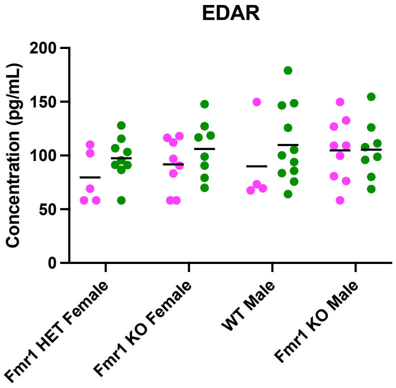

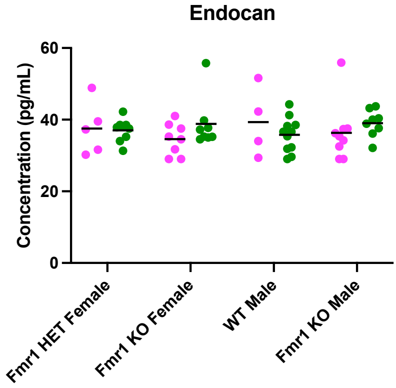

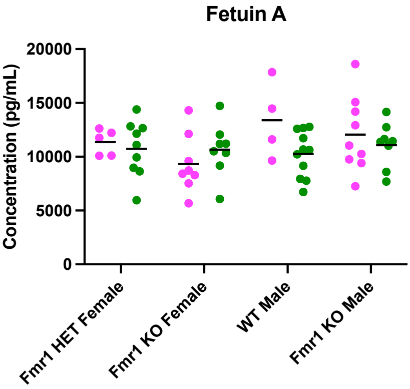

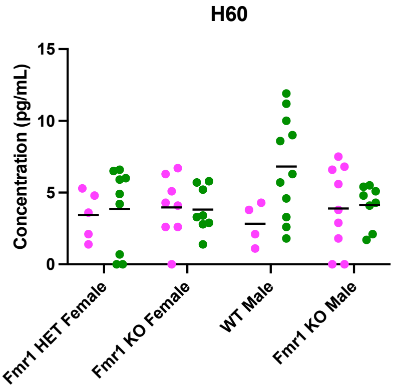

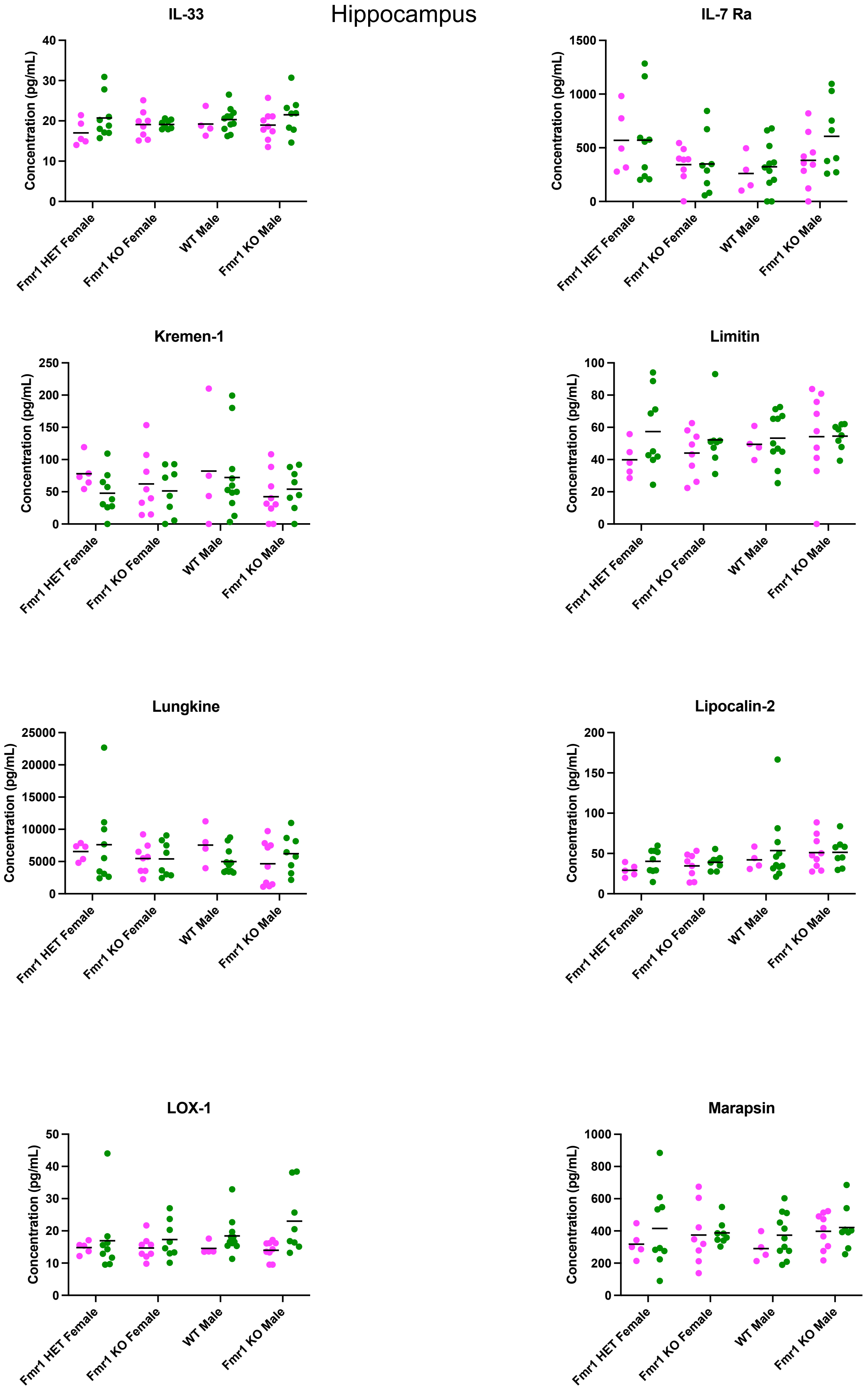
Hippocampus
IL-33
IL-7 Ra
Kremen-1
Limitin
Lungkine
Lipocalin-2
LOX-1
Marapsin
Concentration (pg/mL)
Fmr1 HET Female
Fmr1 KO Female
WT Male
Fmr1 KO Male

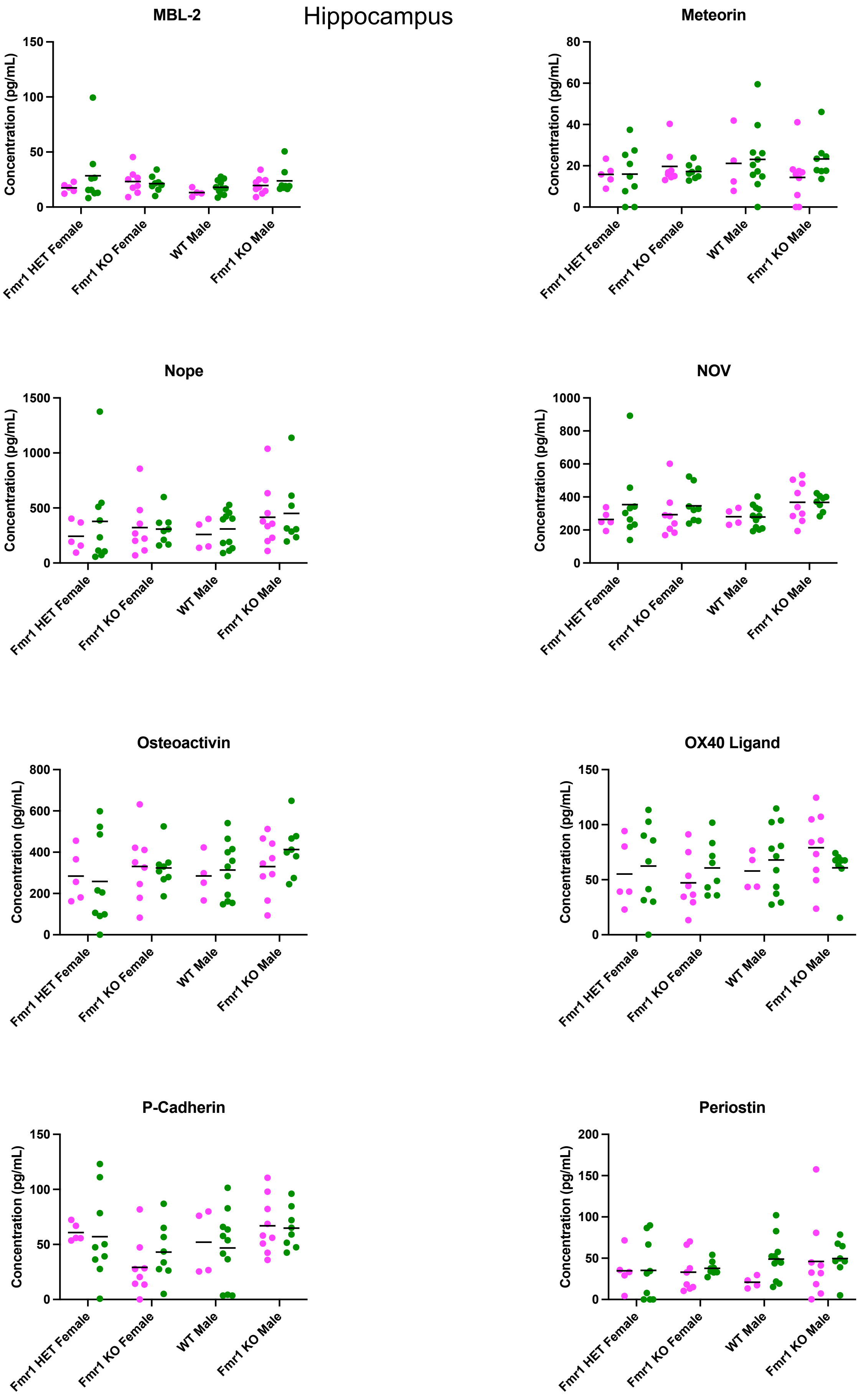
Hippocampus
MBL-2
Meteorin
Nope
NOV
Osteoactivin
OX40 Ligand
P-Cadherin
Periostin
Concentration (pg/mL)
Fmr1 HET Female
Fmr1 KO Female
WT Male
Fmr1 KO Male
0
50
100
150
20
40
60
80
500
1000
1500
200
400
600
800
1000

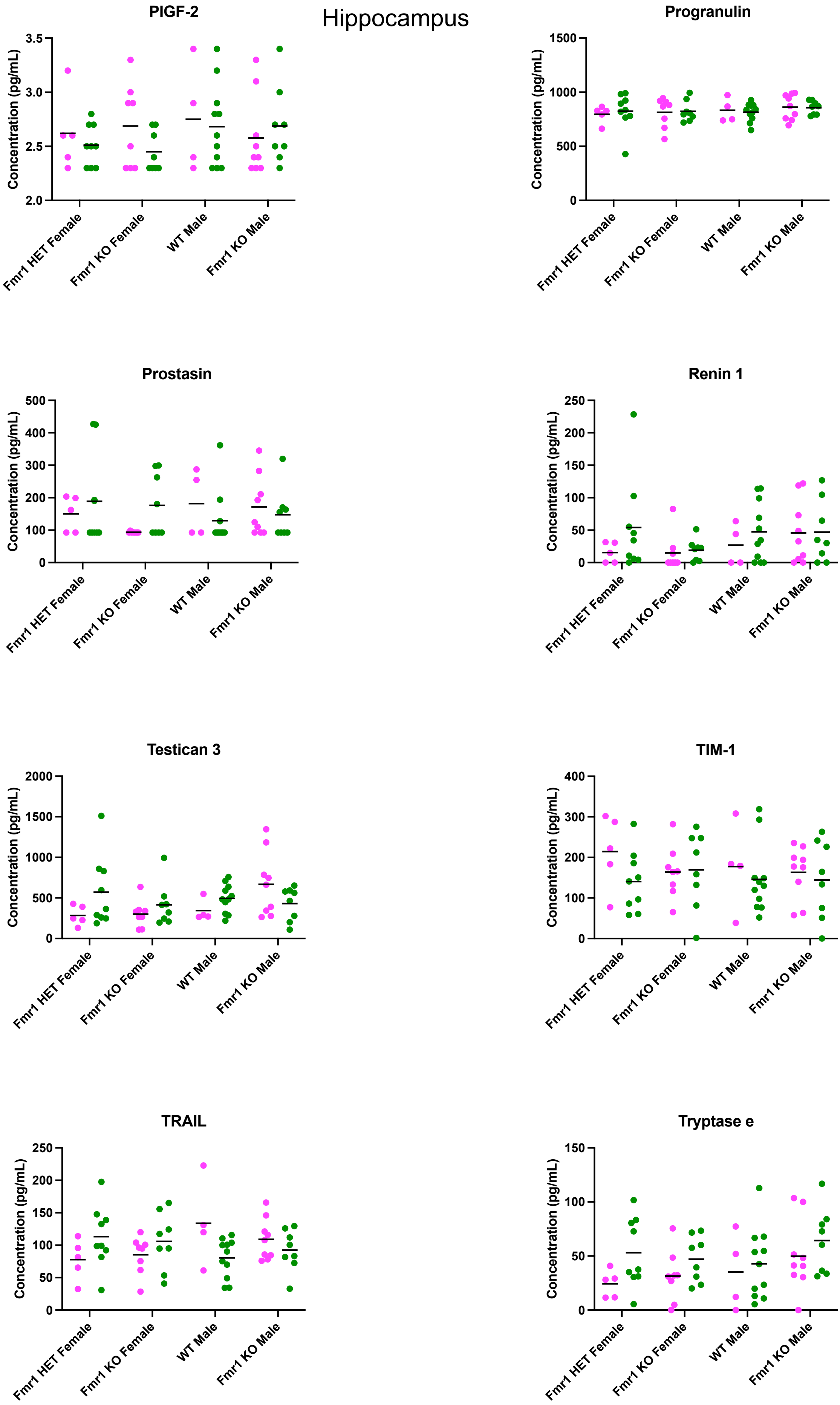
Hippocampus
PlGF-2
Progranulin
Prostasin
Renin 1
Testican 3
TIM-1
TRAIL
Tryptase e
Concentration (pg/mL)
Fmr1 HET Female
Fmr1 KO Female
WT Male
Fmr1 KO Male
2.0
2.5
3.0
3.5
0
500
1000
1500
100
200
300
400
50
150
250
2000

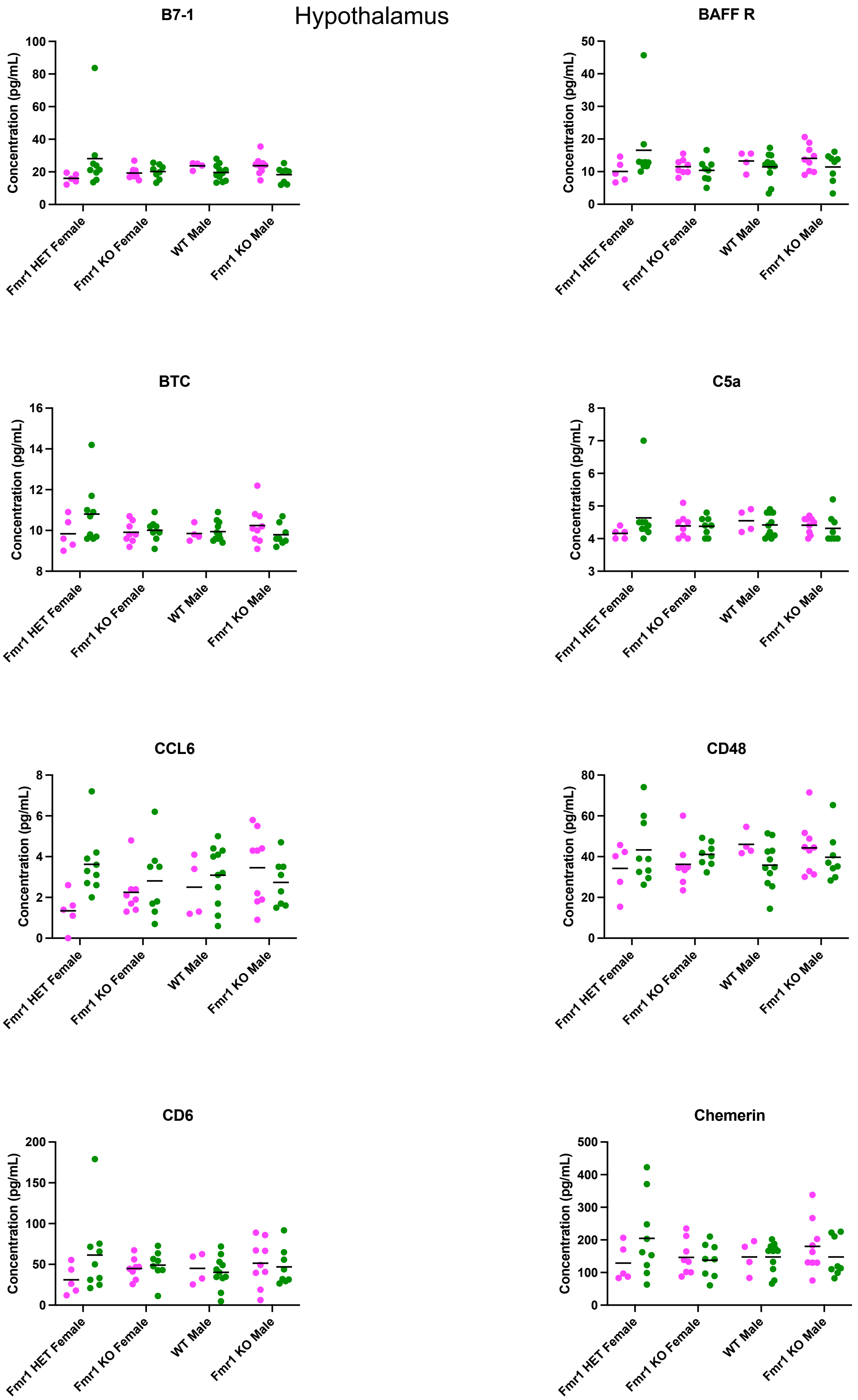
Hypothalamus
B7-1
BAFF R
BTC
C5a
CCL6
CD48
CD6
Chemerin
Concentration (pg/mL)
Fmr1 HET Female
Fmr1 KO Female
WT Male
Fmr1 KO Male

Hypothalamus

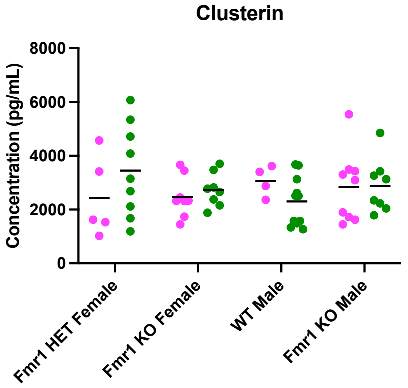

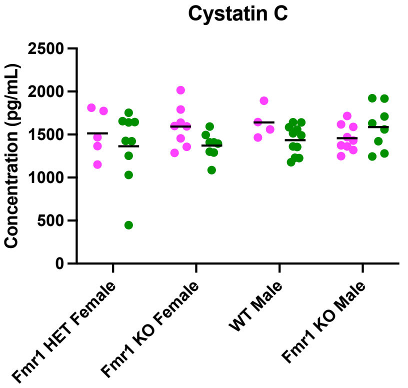

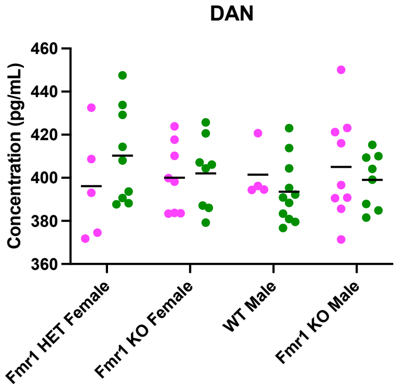

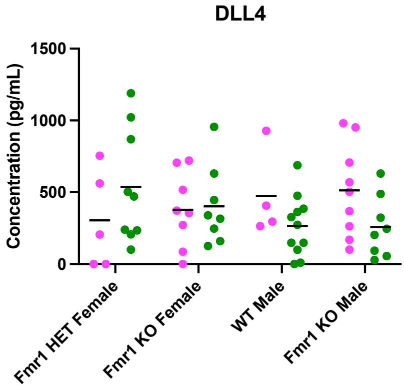

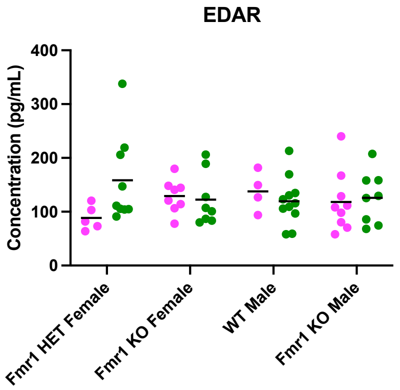

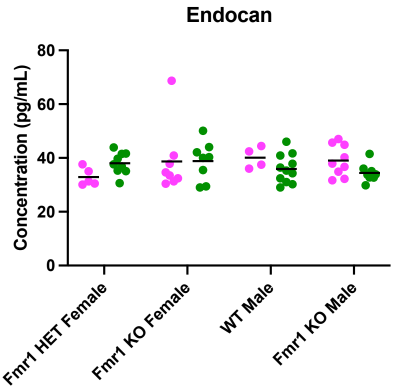

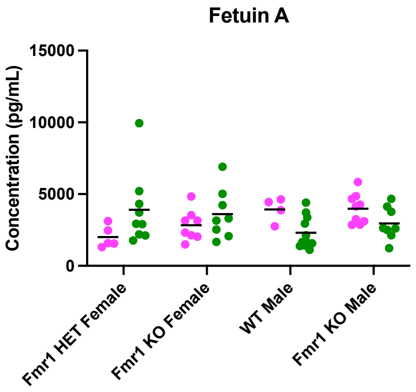

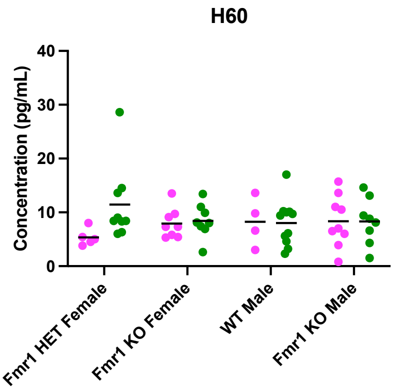

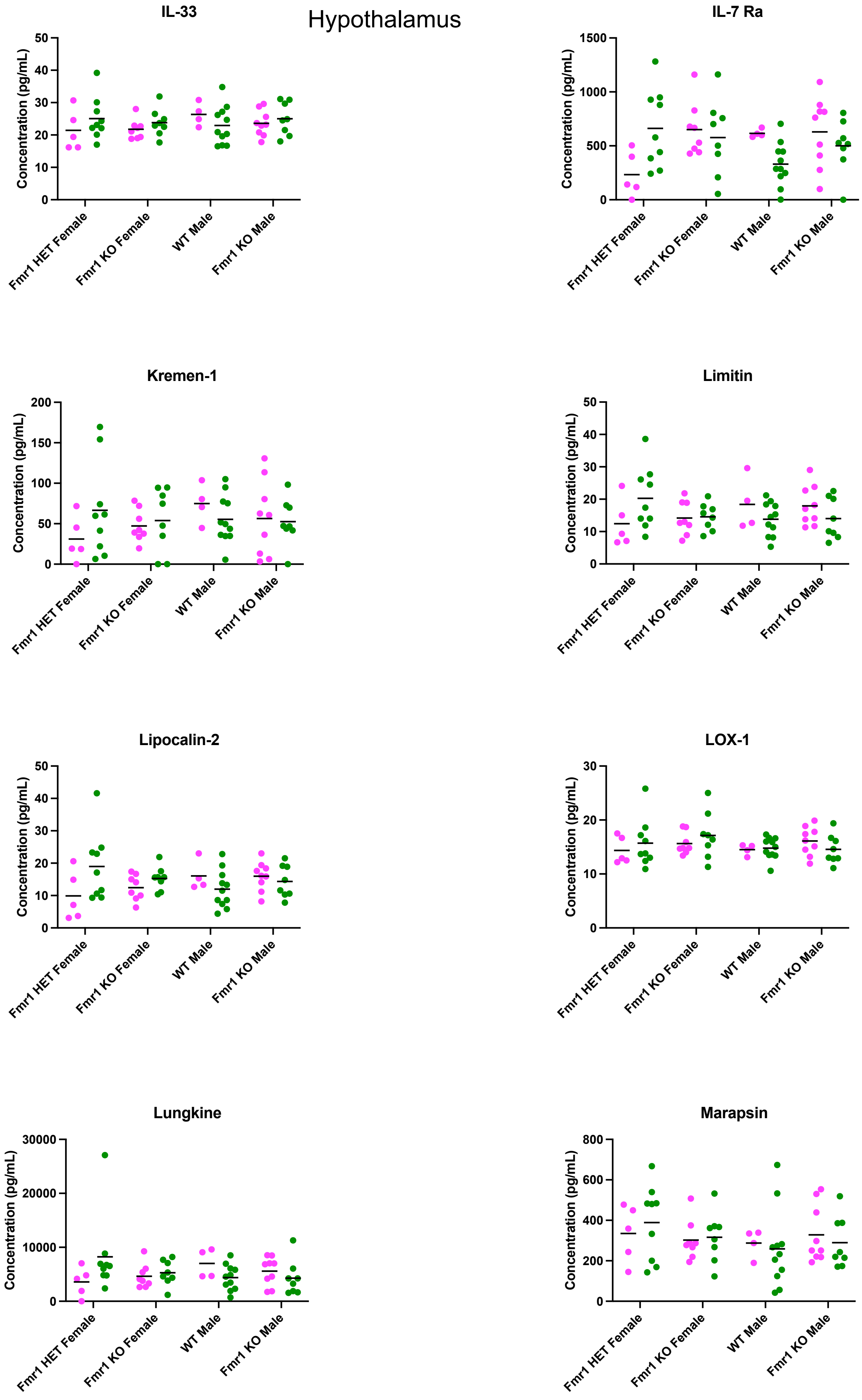
Hypothalamus
IL-33
IL-7 Ra
Kremen-1
Limitin
Lipocalin-2
LOX-1
Lungkine
Marapsin
Concentration (pg/mL)
Fmr1 HET Female
Fmr1 KO Female
WT Male
Fmr1 KO Male

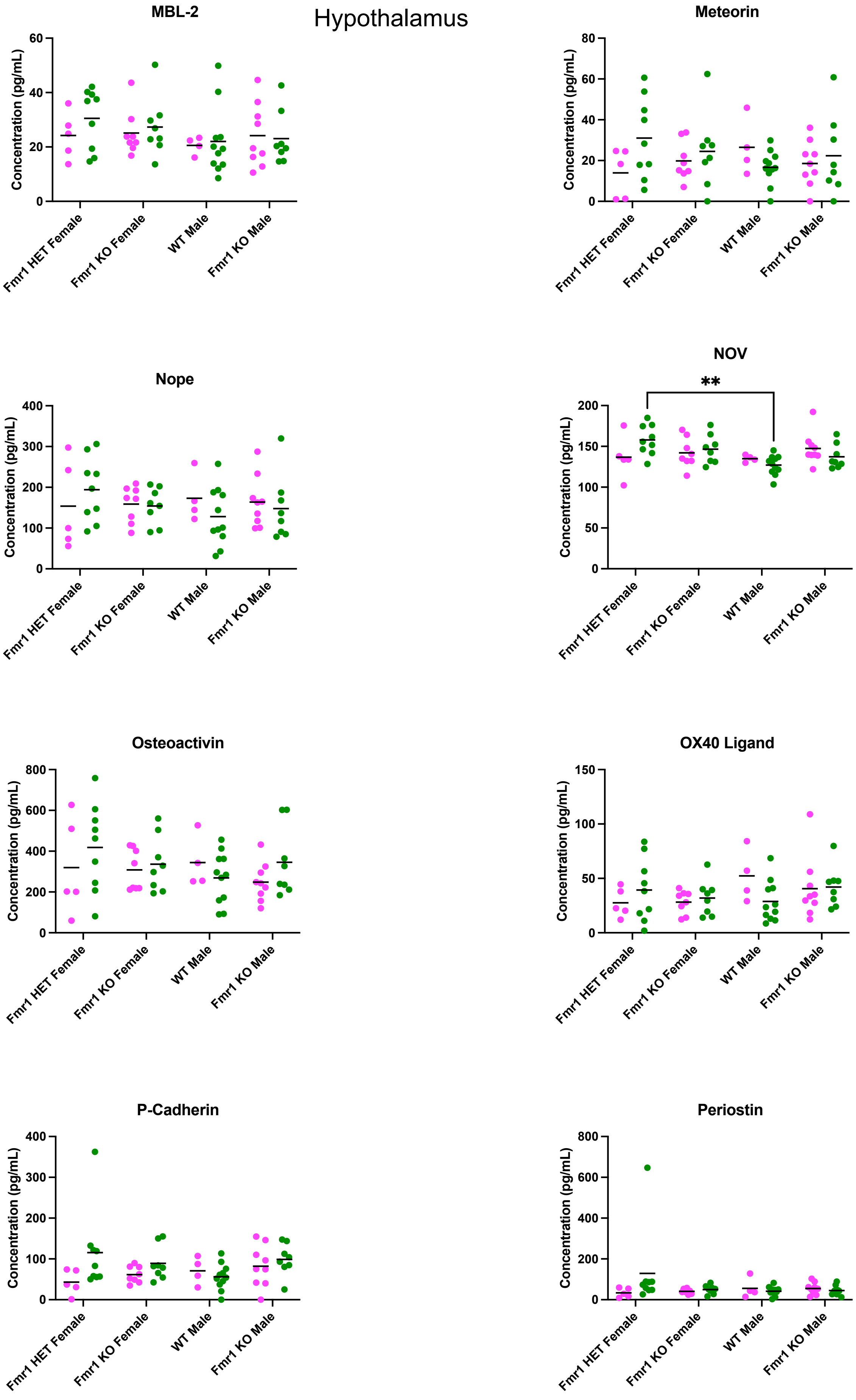
Hypothalamus
MBL-2
Meteorin
Nope
NOV
**
Osteoactivin
OX40 Ligand
P-Cadherin
Periostin
Concentration (pg/mL)
Fmr1 HET Female
Fmr1 KO Female
WT Male
Fmr1 KO Male

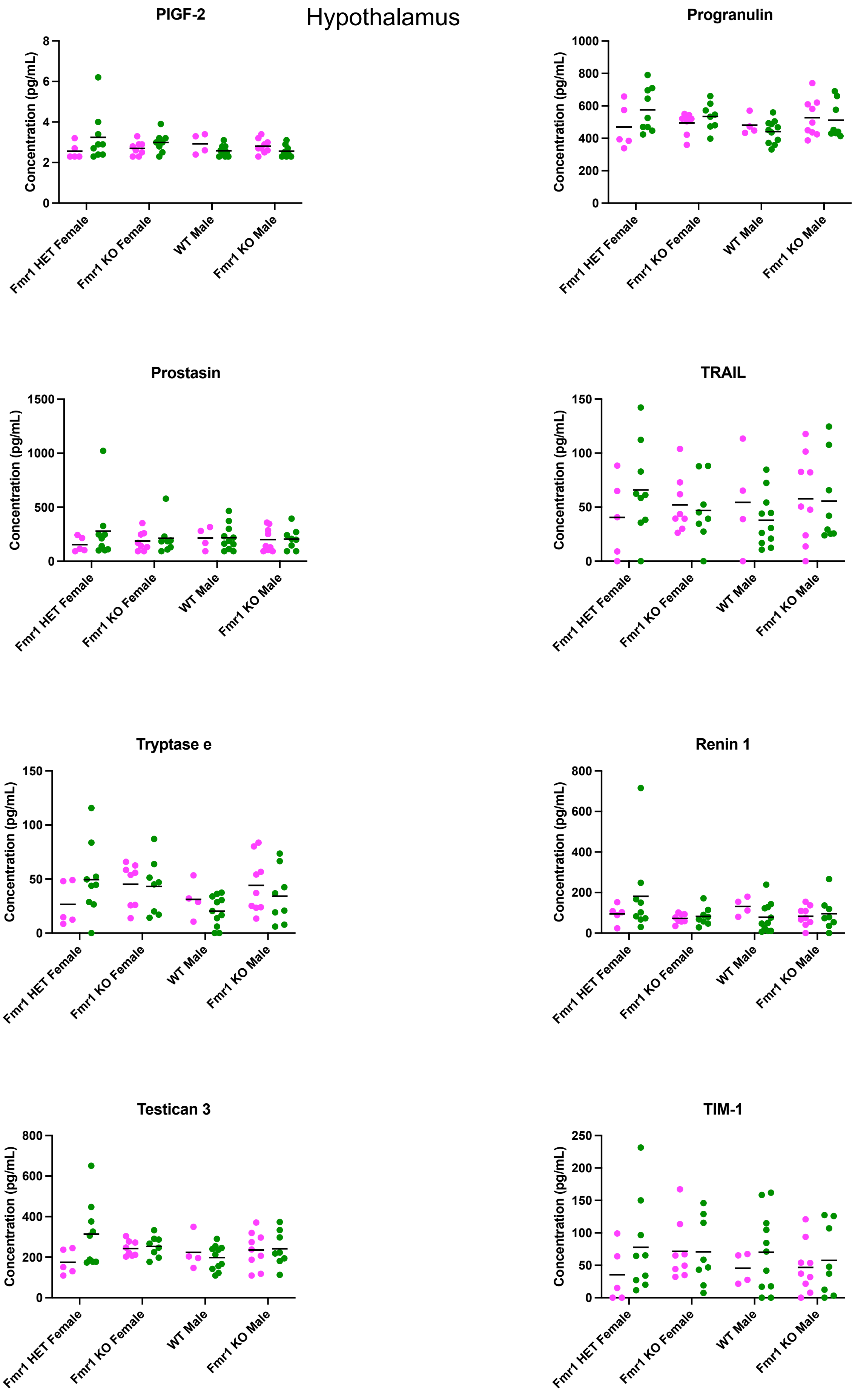
Hypothalamus
PIGF-2
Progranulin
Prostasin
TRAIL
Tryptase e
Renin 1
Testican 3
TIM-1
Concentration (pg/mL)
Fmr1 HET Female
Fmr1 KO Female
WT Male
Fmr1 KO Male

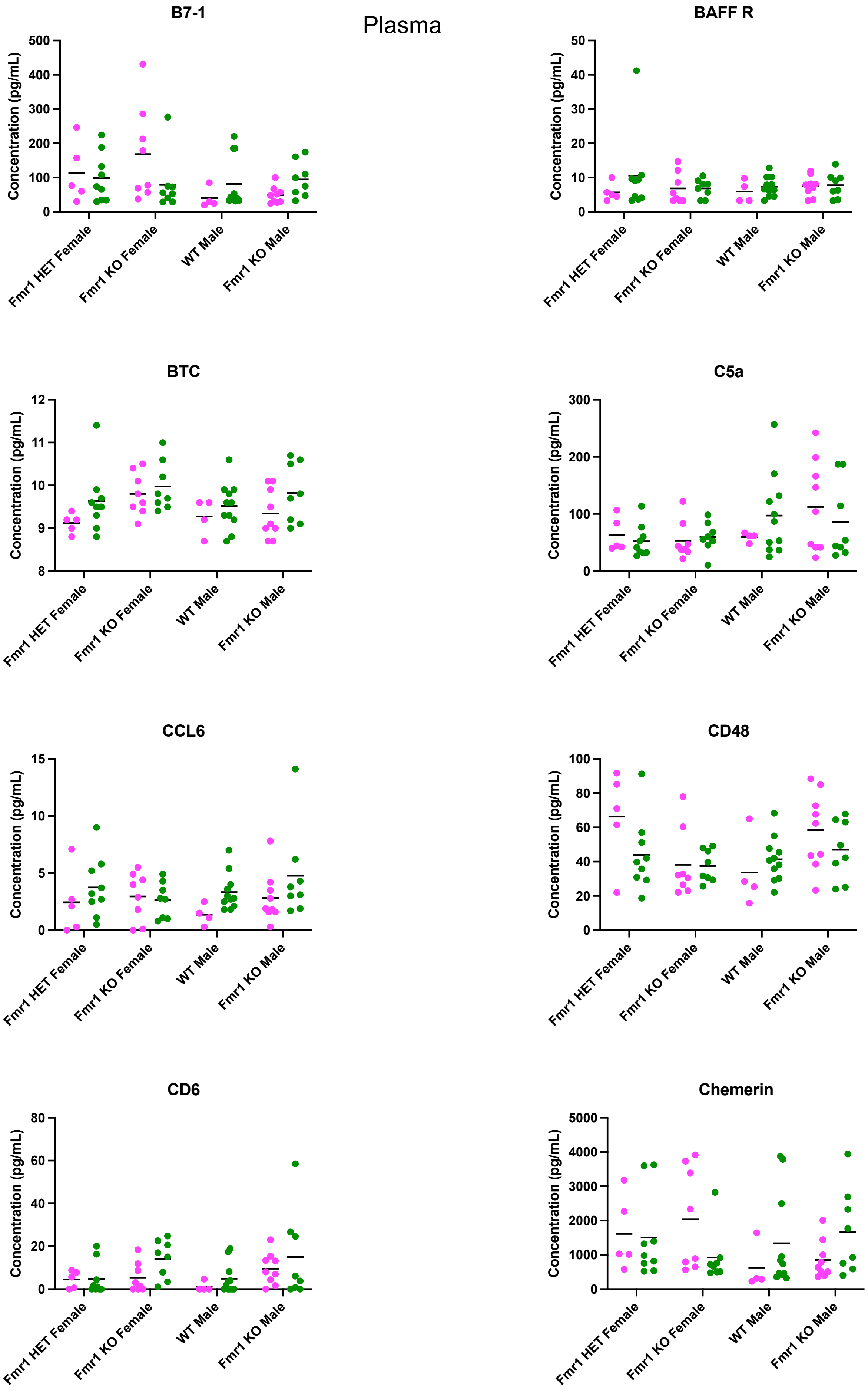
Plasma
B7-1
BAFF R
BTC
C5a
CCL6
CD48
CD6
Chemerin
Concentration (pg/mL)
Fmr1 HET Female
Fmr1 KO Female
WT Male
Fmr1 KO Male
0
100
200
300
400
500
10
20
30
40
50
8
9
10
11
12
5
15
60
80
1000
2000
3000
4000
5000

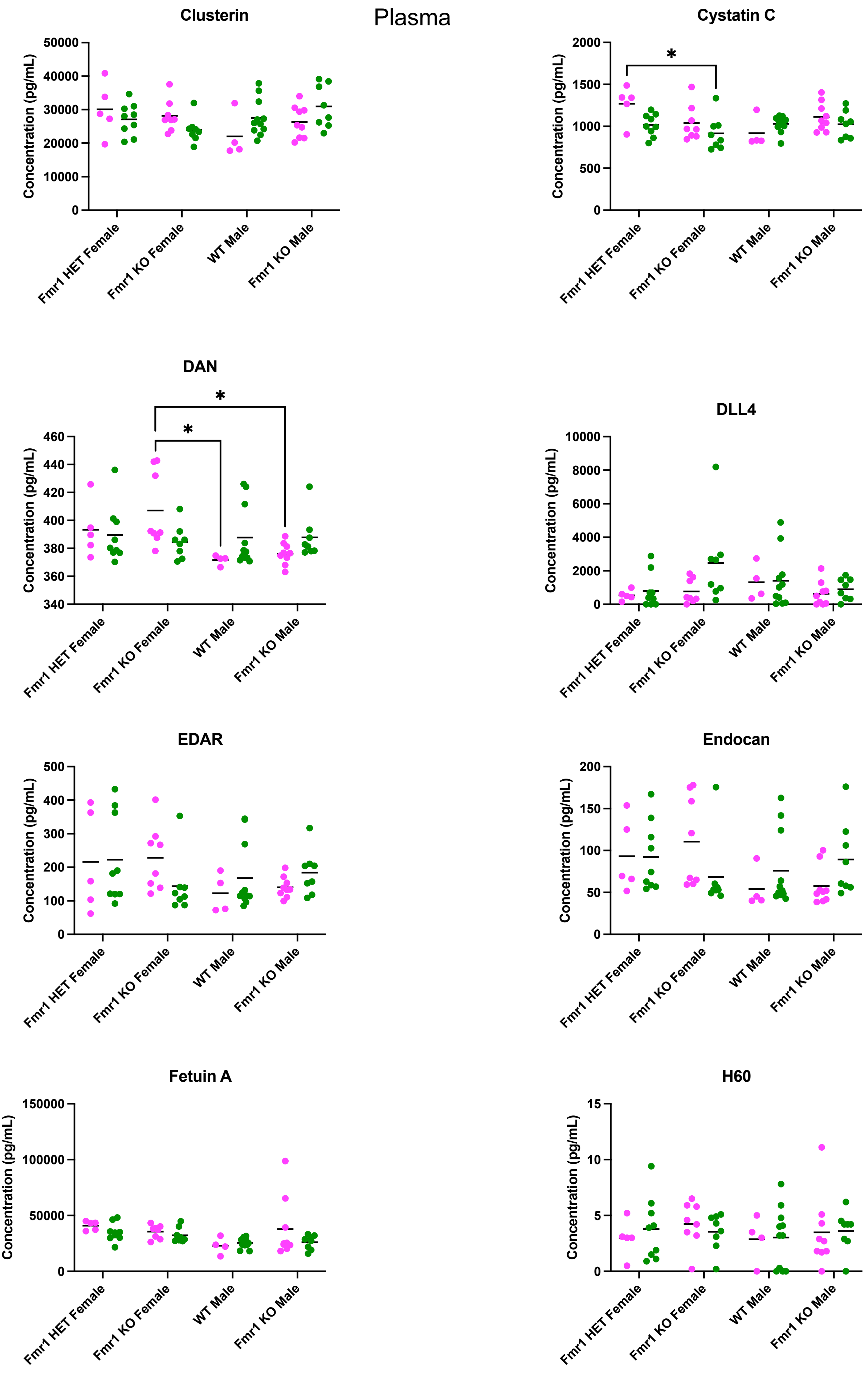

Plasma
Clusterin
Cystatin C
DAN
DLL4
EDAR
Endocan
Fetuin A
H60
Concentration (pg/mL)
Fmr1 HET Female
Fmr1 KO Female
WT Male
Fmr1 KO Male
*

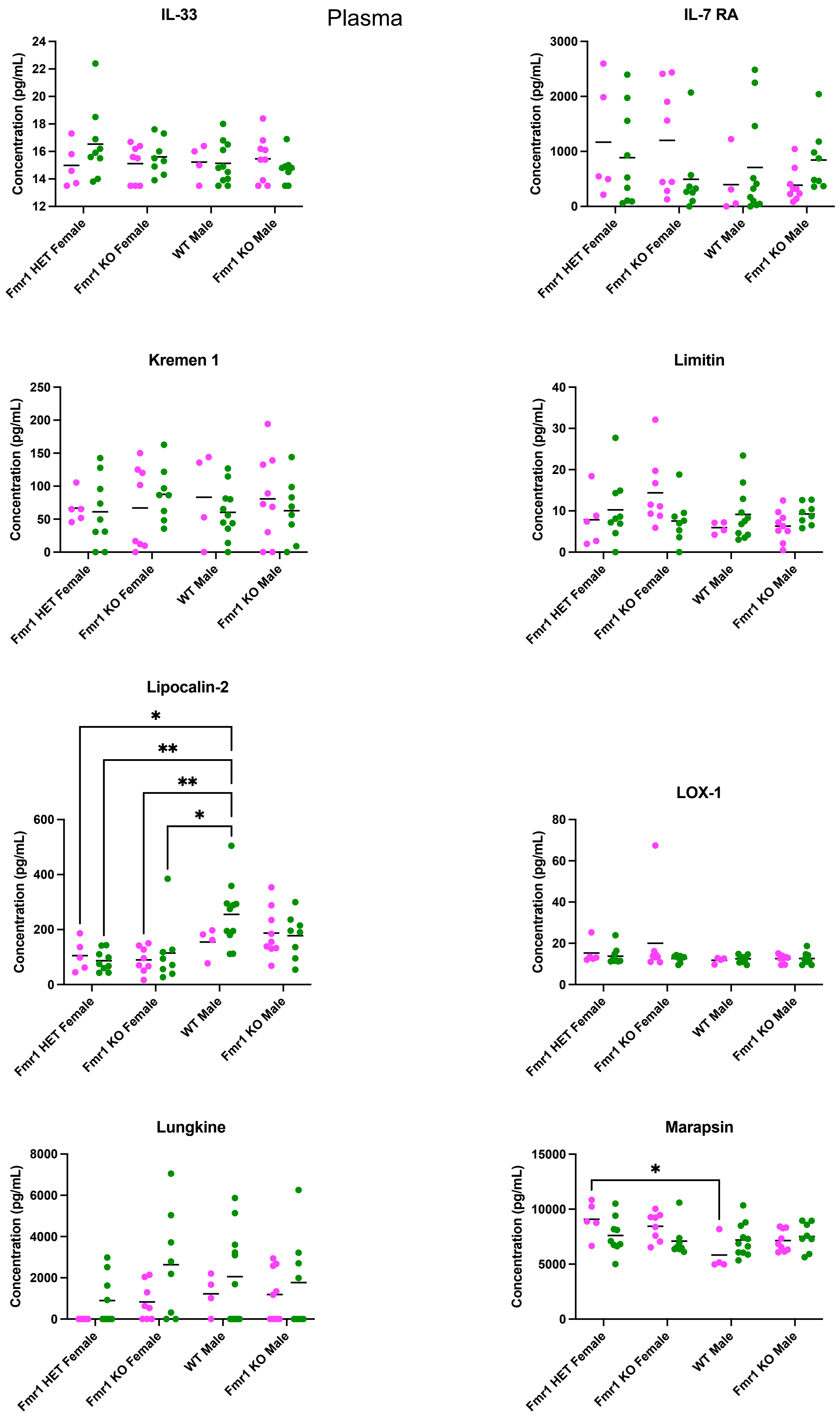
Plasma
IL-33
IL-7 RA
Kremen 1
Limitin
Lipocalin-2
LOX-1
Lungkine
Marapsin
Concentration (pg/mL)
*
**
**
*
*
Fmr1 HET Female
Fmr1 KO Female
WT Male
Fmr1 KO Male

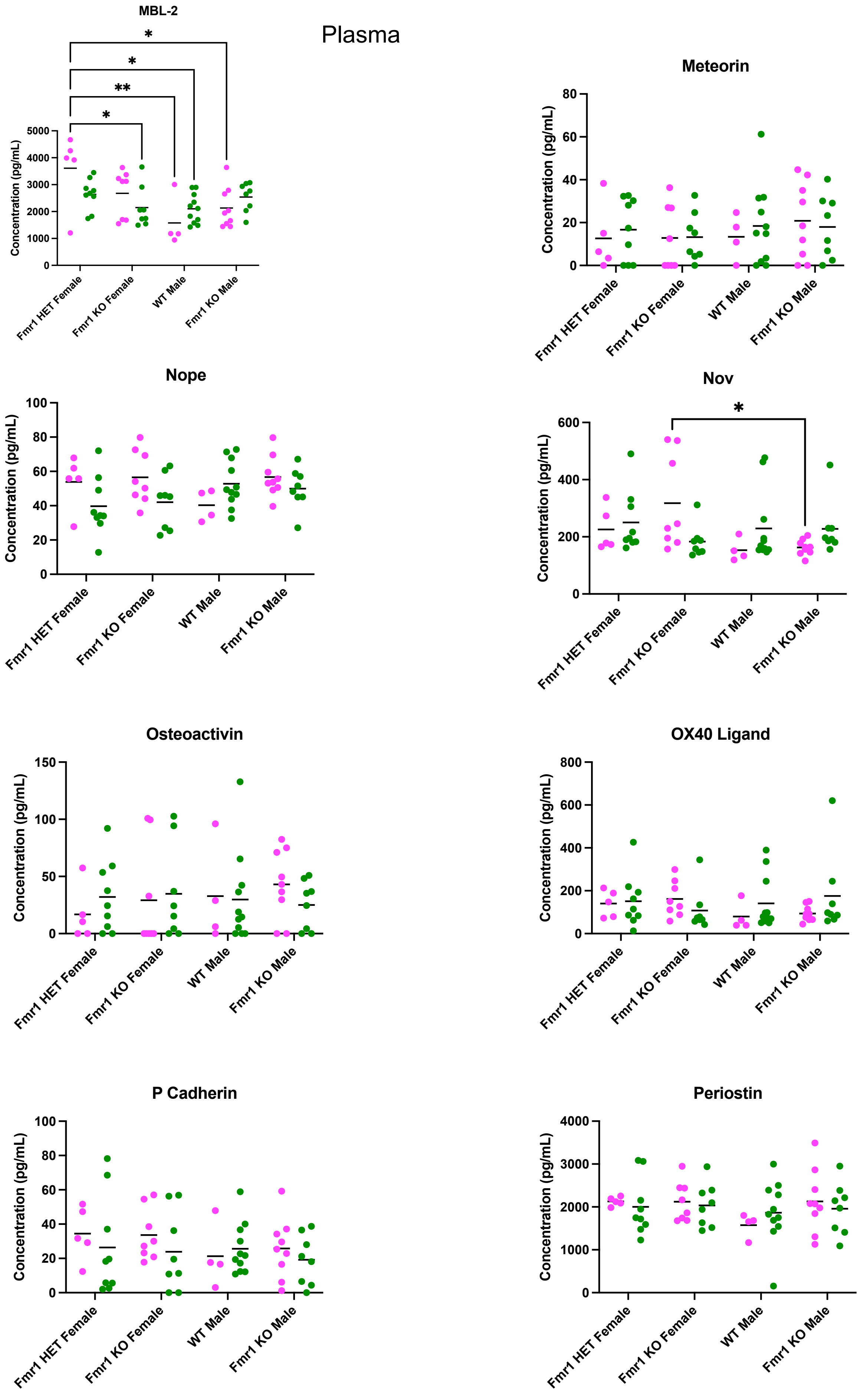

Plasma
MBL-2
Meteorin
Nope
Nov
Osteoactivin
OX40 Ligand
P Cadherin
Periostin
Concentration (pg/mL)
Fmr1 HET Female
Fmr1 KO Female
WT Male
Fmr1 KO Male
*
**

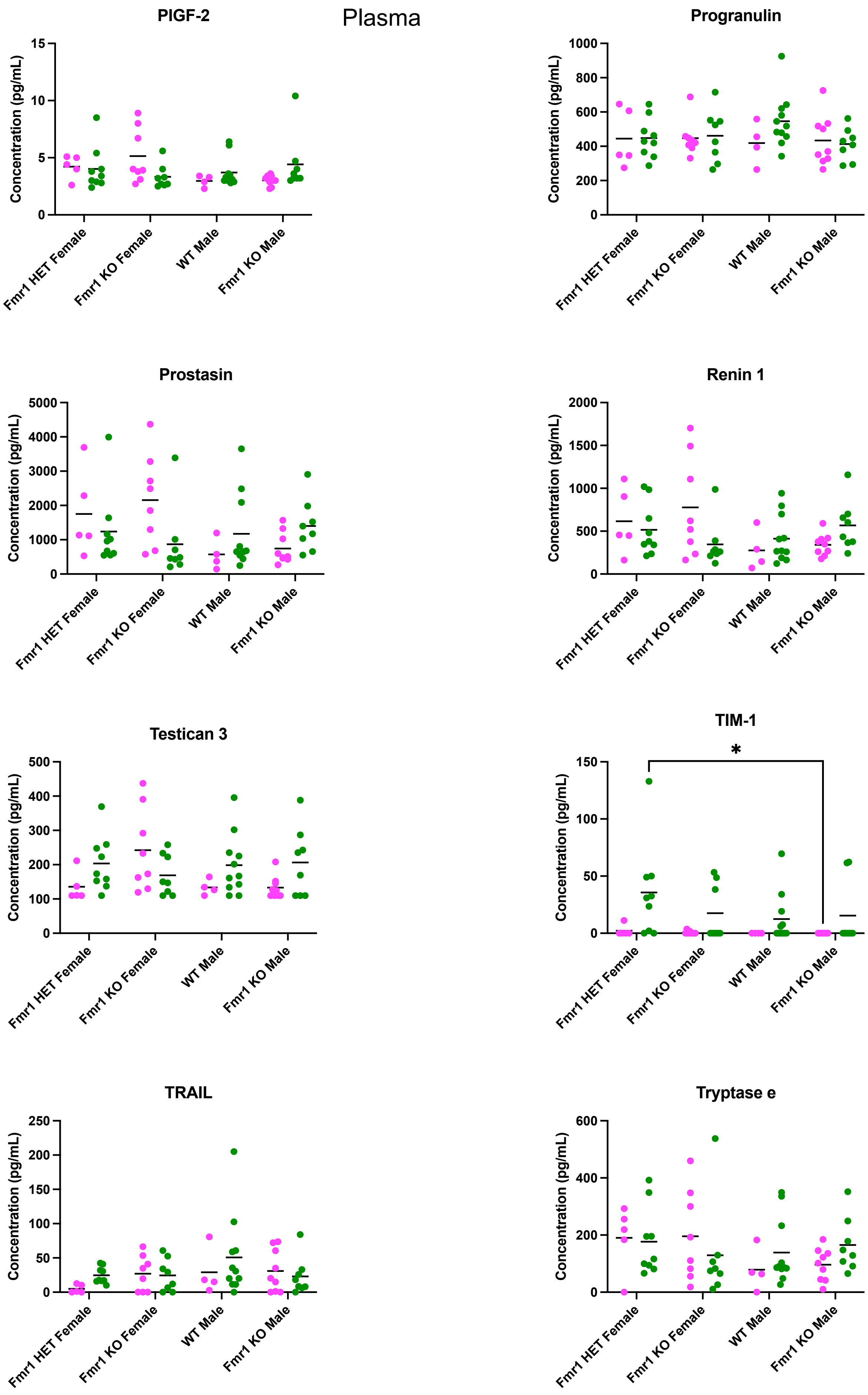

Plasma
PIGF-2
Progranulin
Prostasin
Renin 1
Testican 3
TIM-1
*
TRAIL
Tryptase e
Concentration (pg/mL)
Fmr1 HET Female
Fmr1 KO Female
WT Male
Fmr1 KO Male
