# Supplementary material for: Soy Protein Isolate Affects Blood and Brain Biomarker Expression in a Mouse Model of Fragile X
Source: Int J Mol Sci. 2025 Jun 26;26(13):6137. doi: 10.3390/ijms26136137 (PMC12250412; doi:10.3390/ijms26136137)
Supplement: Supplementary file 1 [file ijms-26-06137-s001.zip › Supplementary File S6b Array 8 Graphs.pdf]

**Supplementary File S6.** Protein expression of Array 8 targets as function of *Fmr1* genotype and AIN-93G diets. Mice on AIN-93G/cas (colored pink) included n=5 *Fmr1*$^{HET}$ female, n=8 *Fmr1*$^{KO}$ female, n=4 WT male and n=9 *Fmr1*$^{KO}$ male. Mice on AIN-93G/soy (colored green) included n=9 *Fmr1*$^{HET}$ female, n=8 *Fmr1*$^{KO}$ female, n=11 WT male and n=8 *Fmr1*$^{KO}$ male. The average concentration in cortex, hippocampus, hypothalamus and plasma in pg/mL was plotted versus genotype. Statistics were determined by 2-way ANOVA and Tukey's multiple comparison tests denoted by $p < 0.05$ (*), $p < 0.01$ (**), $p < 0.001$ (***) and $p < 0.0001$ (****).

cortex

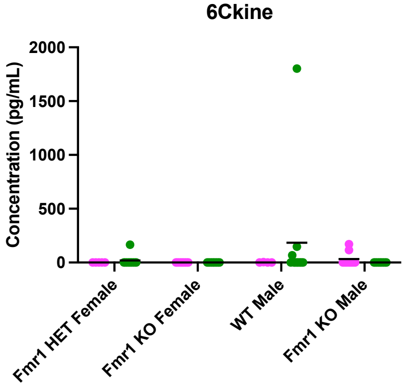
6Ckine
Concentration (pg/mL)
2000
1500
1000
500
0
Fmr1 HET Female
Fmr1 KO Female
WT Male
Fmr1 KO Male

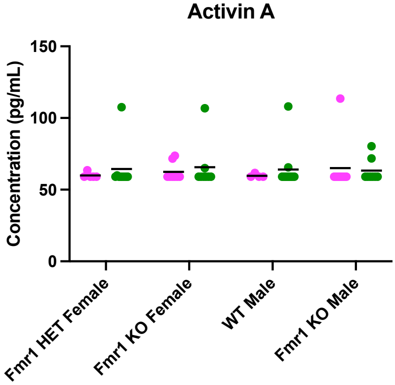
Activin A
Concentration (pg/mL)
150
100
50
0
Fmr1 HET Female
Fmr1 KO Female
WT Male
Fmr1 KO Male

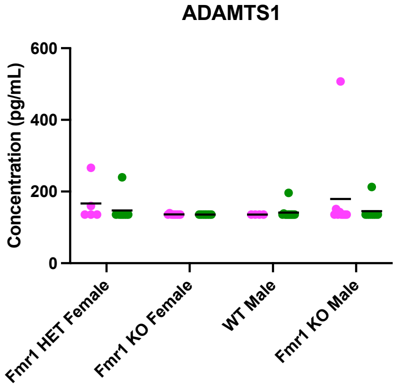
ADAMTS1
Concentration (pg/mL)
600
400
200
0
Fmr1 HET Female
Fmr1 KO Female
WT Male
Fmr1 KO Male

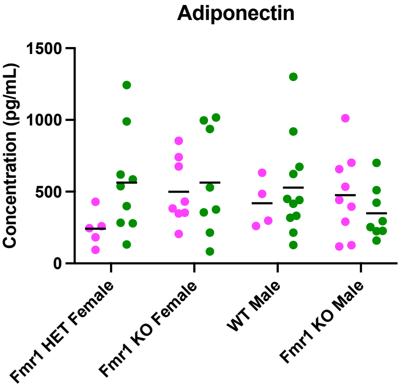
Adiponectin
Concentration (pg/mL)
1500
1000
500
0
Fmr1 HET Female
Fmr1 KO Female
WT Male
Fmr1 KO Male

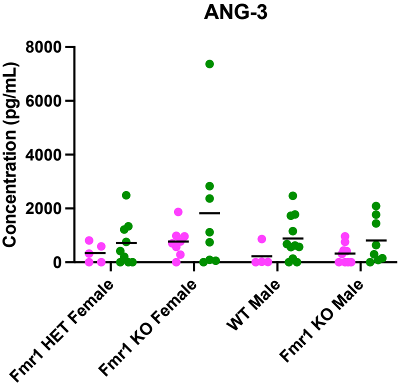
ANG-3
Concentration (pg/mL)
8000
6000
4000
2000
0
Fmr1 HET Female
Fmr1 KO Female
WT Male
Fmr1 KO Male

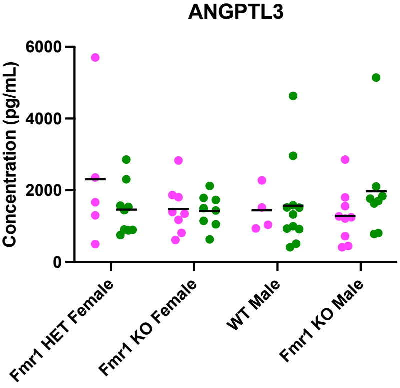
ANGPTL3
Concentration (pg/mL)
6000
4000
2000
0
Fmr1 HET Female
Fmr1 KO Female
WT Male
Fmr1 KO Male

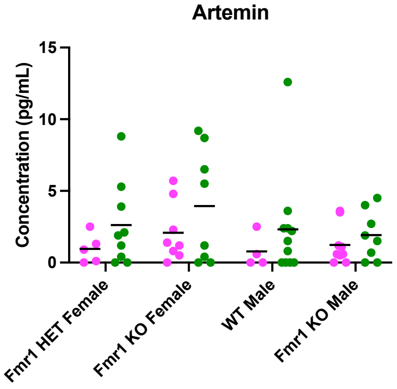
Artemin
Concentration (pg/mL)
15
10
5
0
Fmr1 HET Female
Fmr1 KO Female
WT Male
Fmr1 KO Male

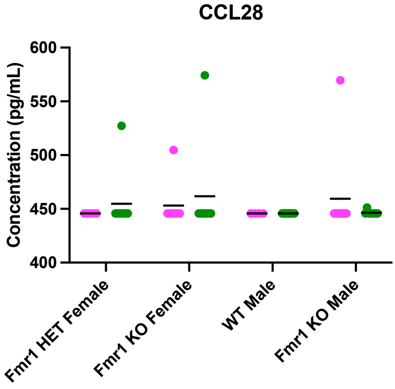
CCL28
Concentration (pg/mL)
600
550
500
450
400
Fmr1 HET Female
Fmr1 KO Female
WT Male
Fmr1 KO Male

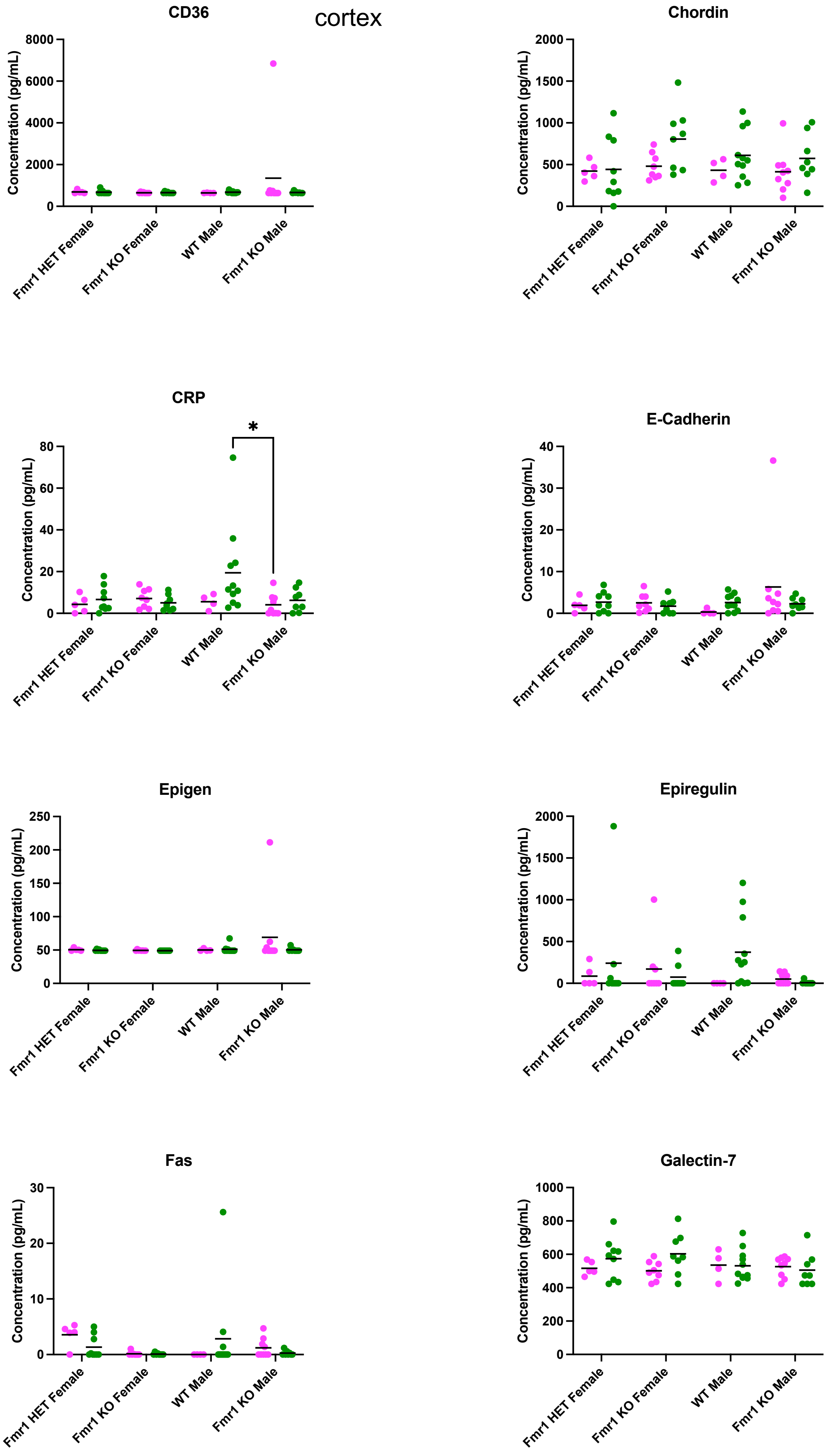
cortex
CD36
Chordin
CRP
E-Cadherin
Epigen
Epiregulin
Fas
Galectin-7
Concentration (pg/mL)
*
Fmr1 HET Female
Fmr1 KO Female
WT Male
Fmr1 KO Male
0
2000
4000
6000
8000
500
1000
1500
20
40
60
80
10
30
50
100
150
200
250
200
400
600
800

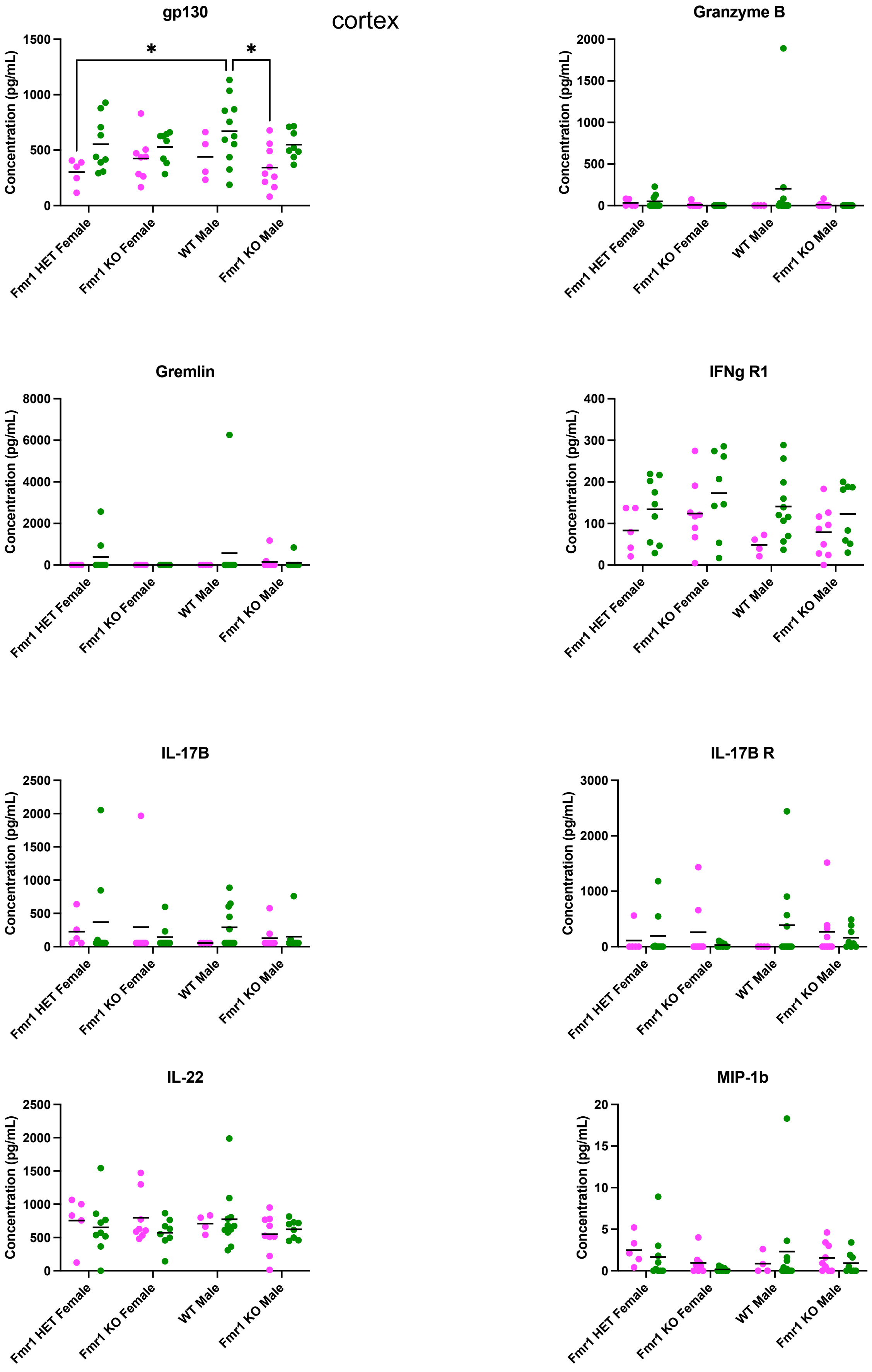
cortex
gp130
Granzyme B
Gremlin
IFNg R1
IL-17B
IL-17B R
IL-22
MIP-1b
Concentration (pg/mL)
*
*
Fmr1 HET Female
Fmr1 KO Female
WT Male
Fmr1 KO Male
0
500
1000
1500
2000
2500
3000
4000
6000
8000
100
200
300
400
5
10
15
20

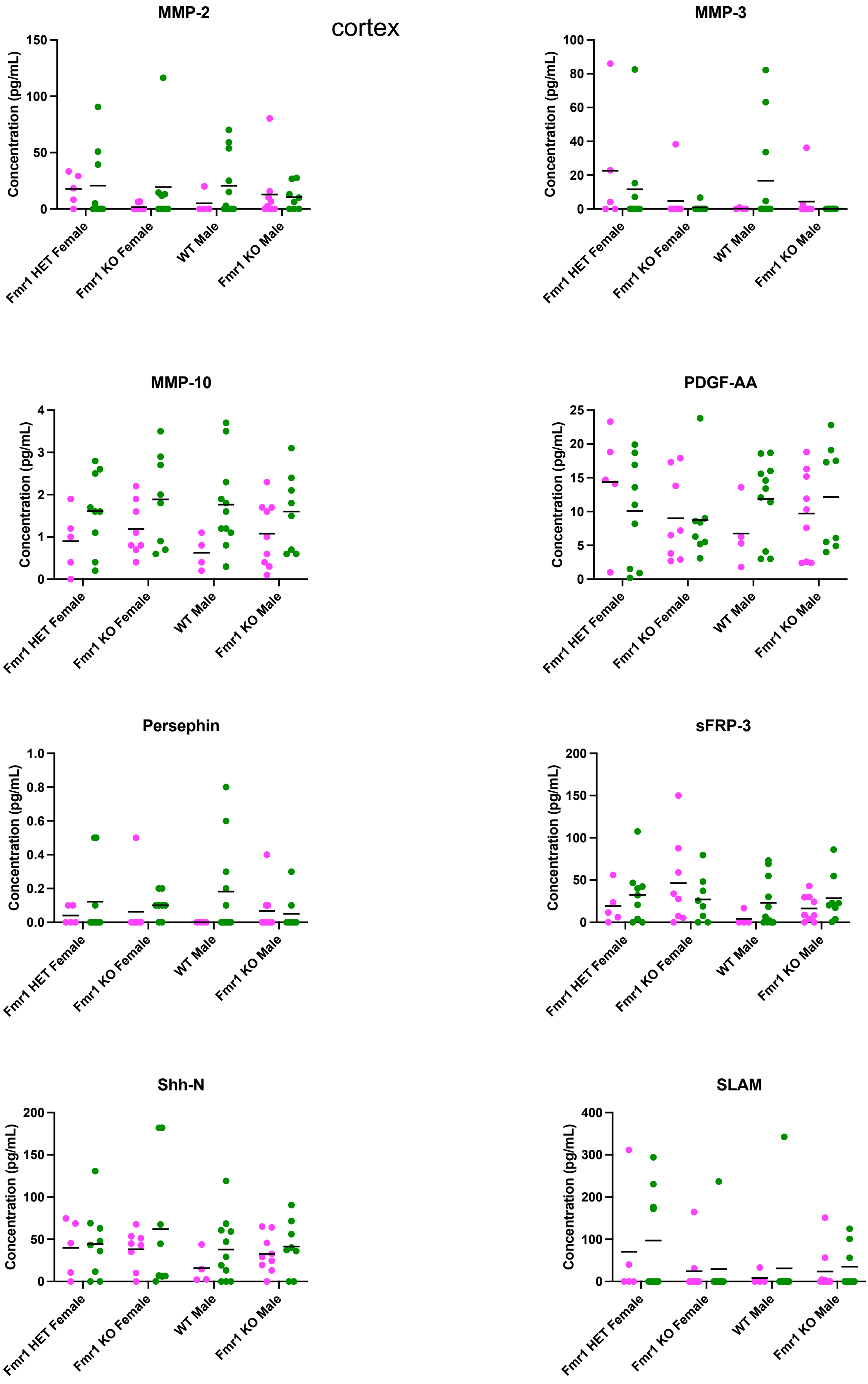
cortex
MMP-2
MMP-3
MMP-10
PDGF-AA
Persephin
sFRP-3
Shh-N
SLAM
Concentration (pg/mL)
Fmr1 HET Female
Fmr1 KO Female
WT Male
Fmr1 KO Male

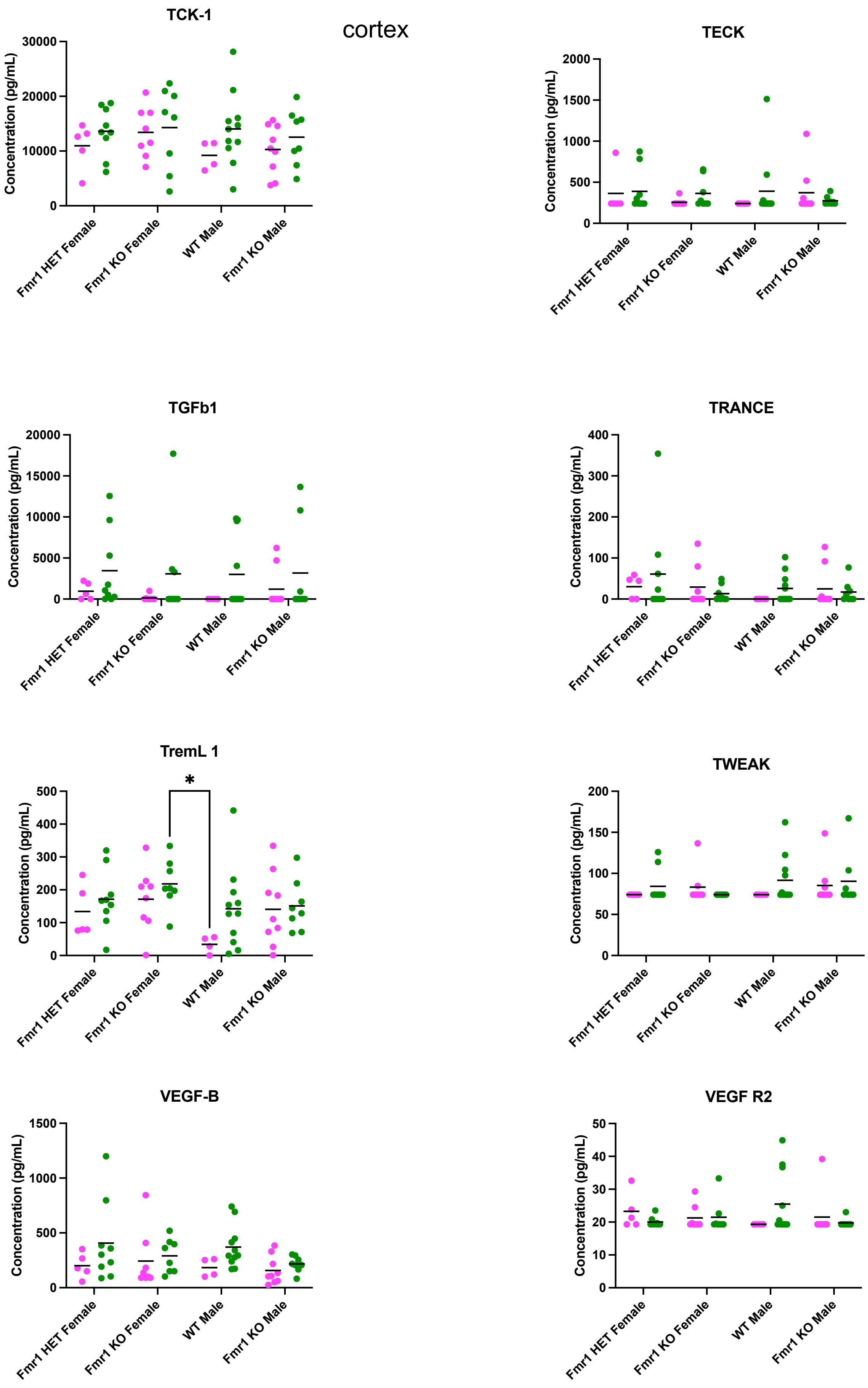

cortex
TCK-1
TECK
TGFb1
TRANCE
TremL 1
*
TWEAK
VEGF-B
VEGF R2
Concentration (pg/mL)
Fmr1 HET Female
Fmr1 KO Female
WT Male
Fmr1 KO Male

# Hippocampus

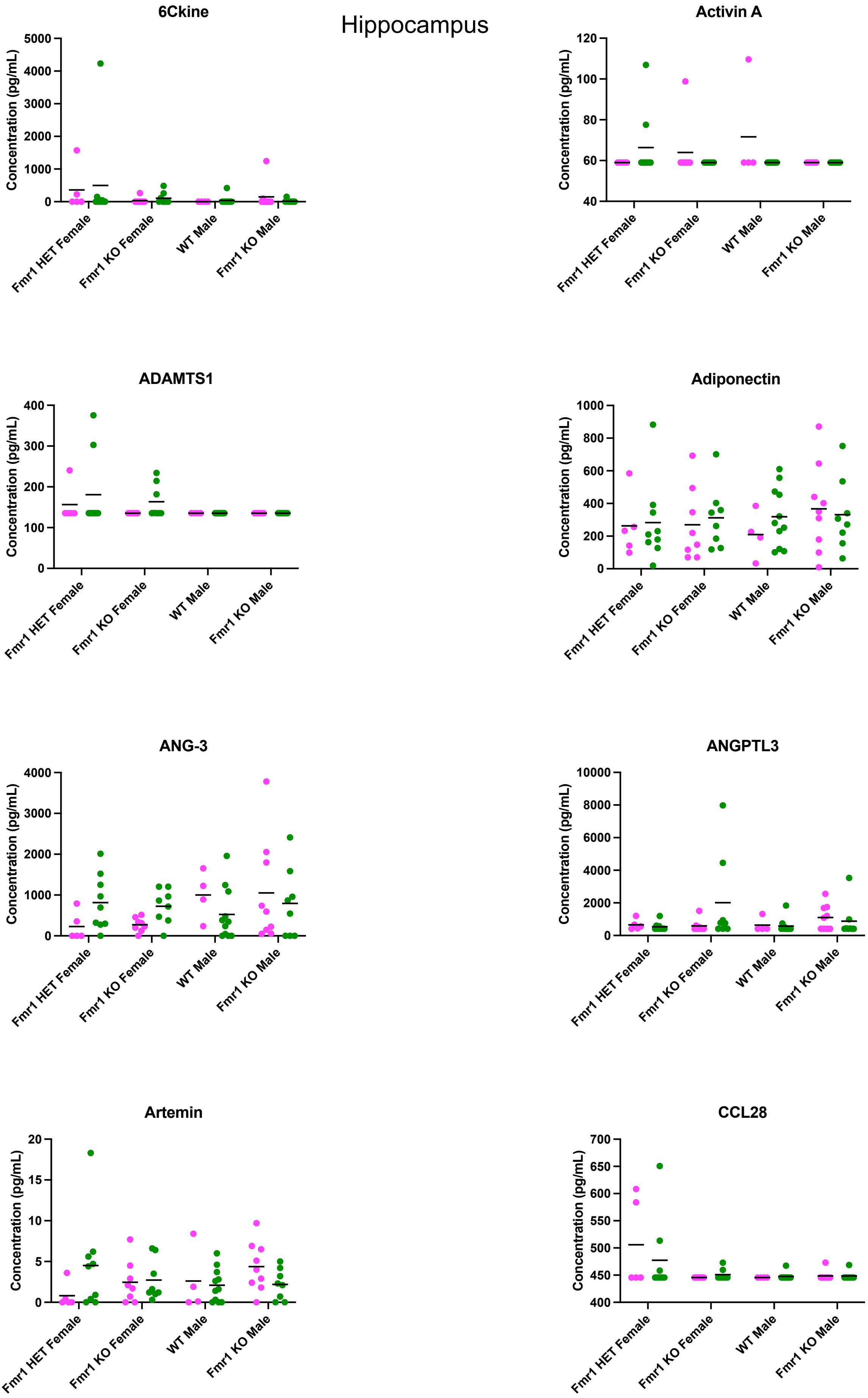

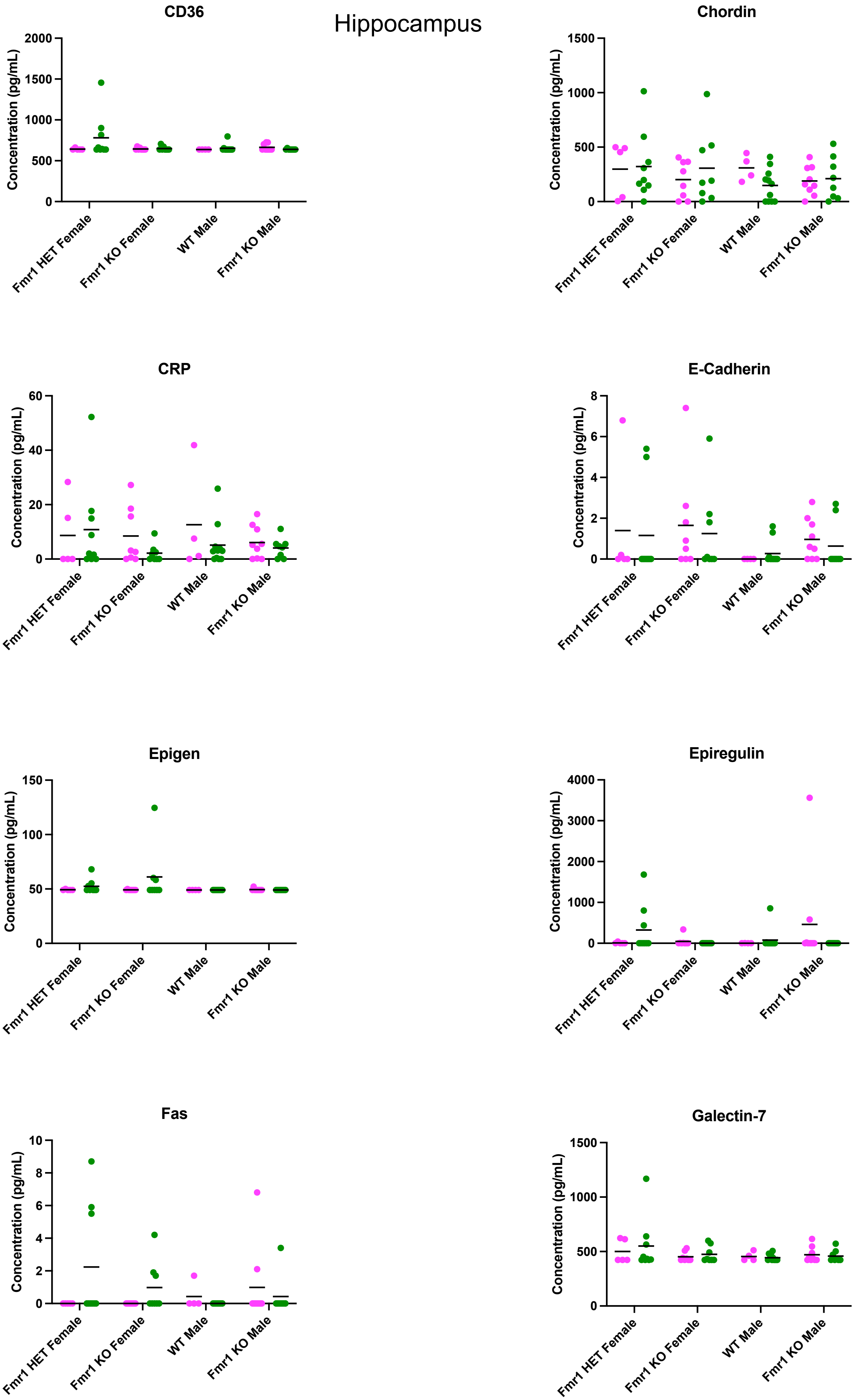
Hippocampus
CD36
Chordin
CRP
E-Cadherin
Epigen
Epiregulin
Fas
Galectin-7
Concentration (pg/mL)
Fmr1 HET Female
Fmr1 KO Female
WT Male
Fmr1 KO Male

# Hippocampus

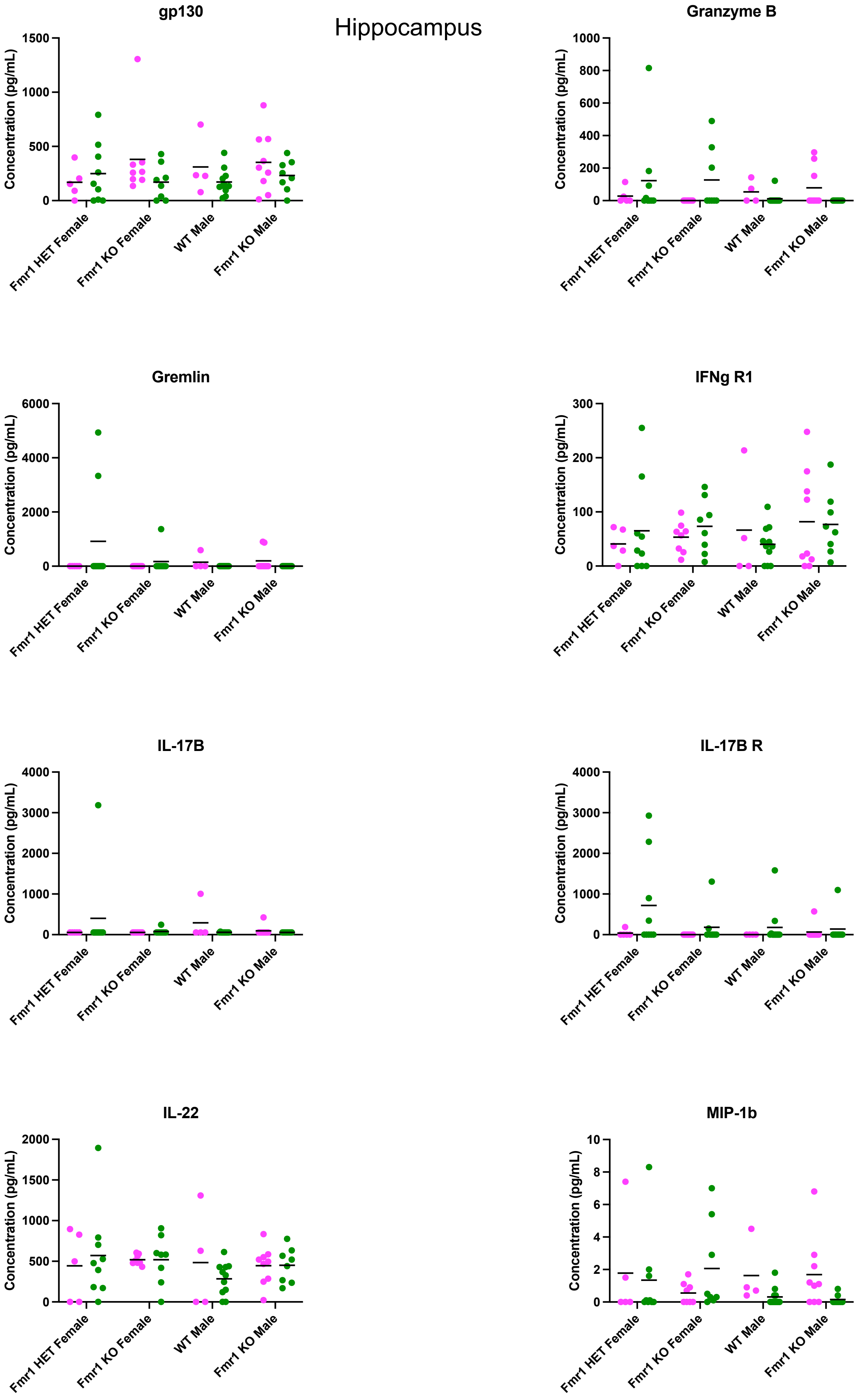

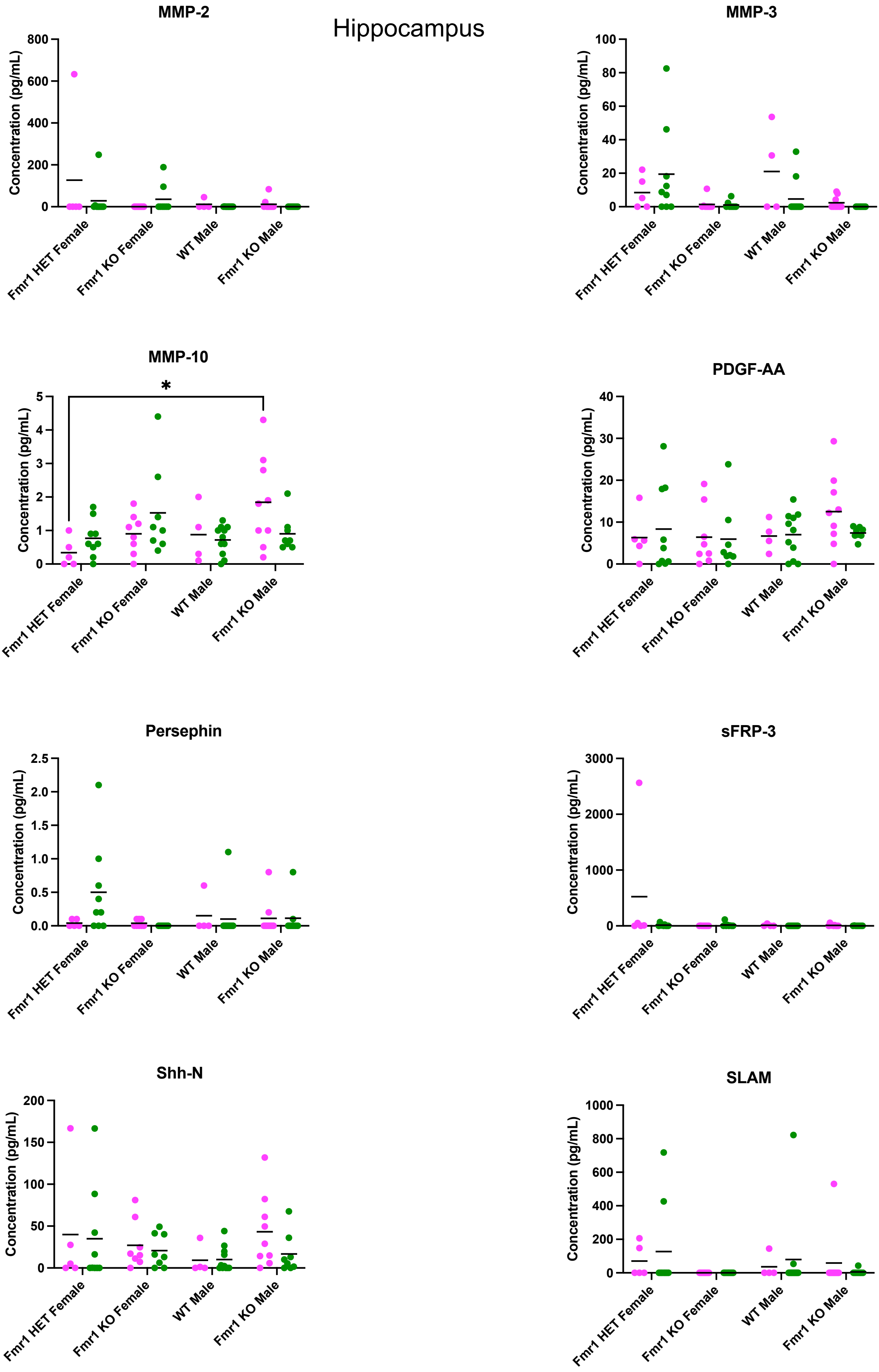
Hippocampus
MMP-2
MMP-3
MMP-10
*
PDGF-AA
Persephin
sFRP-3
Shh-N
SLAM
Concentration (pg/mL)
Fmr1 HET Female
Fmr1 KO Female
WT Male
Fmr1 KO Male

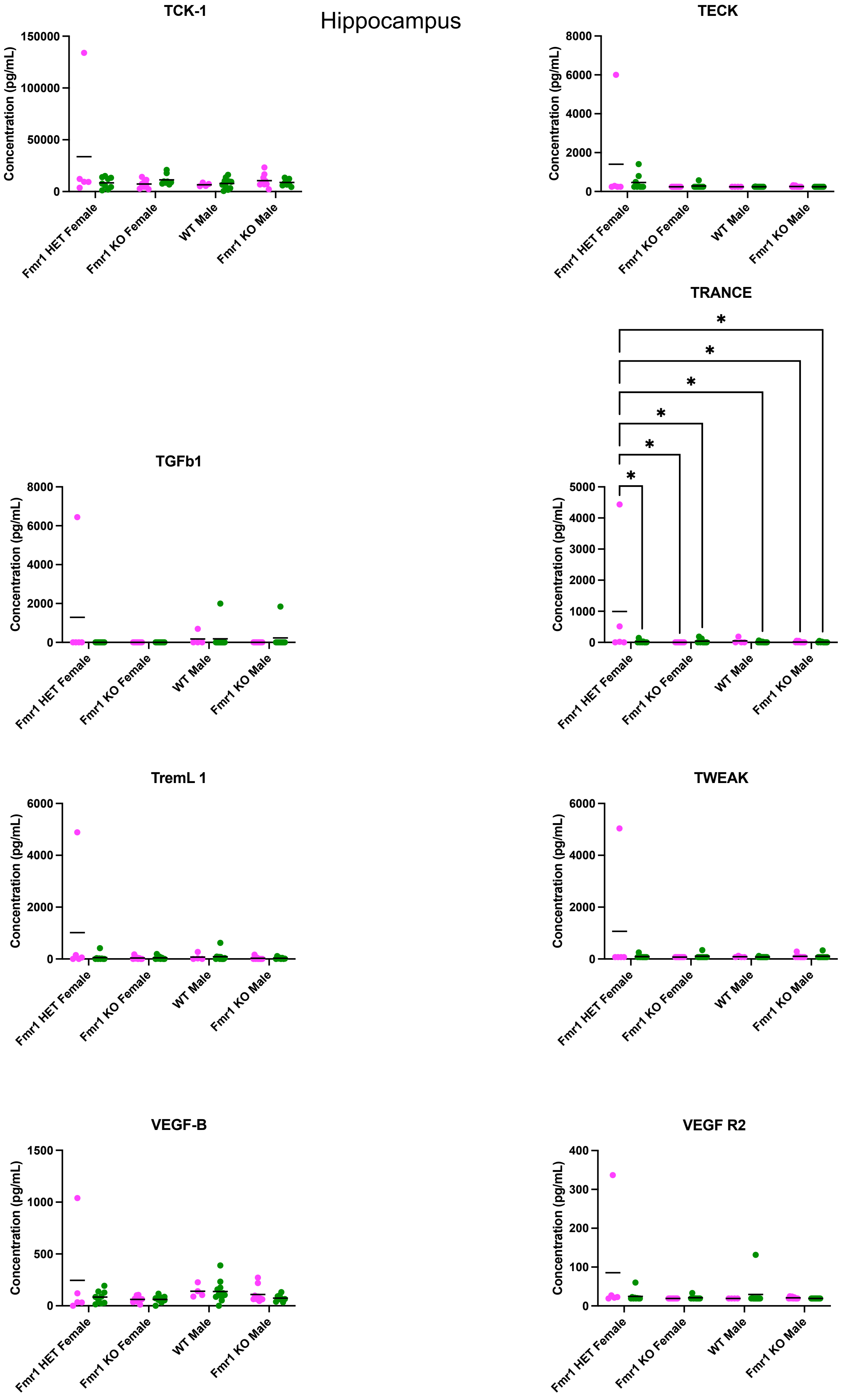
Hippocampus
TCK-1
TECK
TRANCE
TGFb1
TremL 1
TWEAK
VEGF-B
VEGF R2
Concentration (pg/mL)
0
50000
100000
150000
2000
4000
6000
8000
1000
3000
5000
500
1500
100
200
300
400
*
Fmr1 HET Female
Fmr1 KO Female
WT Male
Fmr1 KO Male

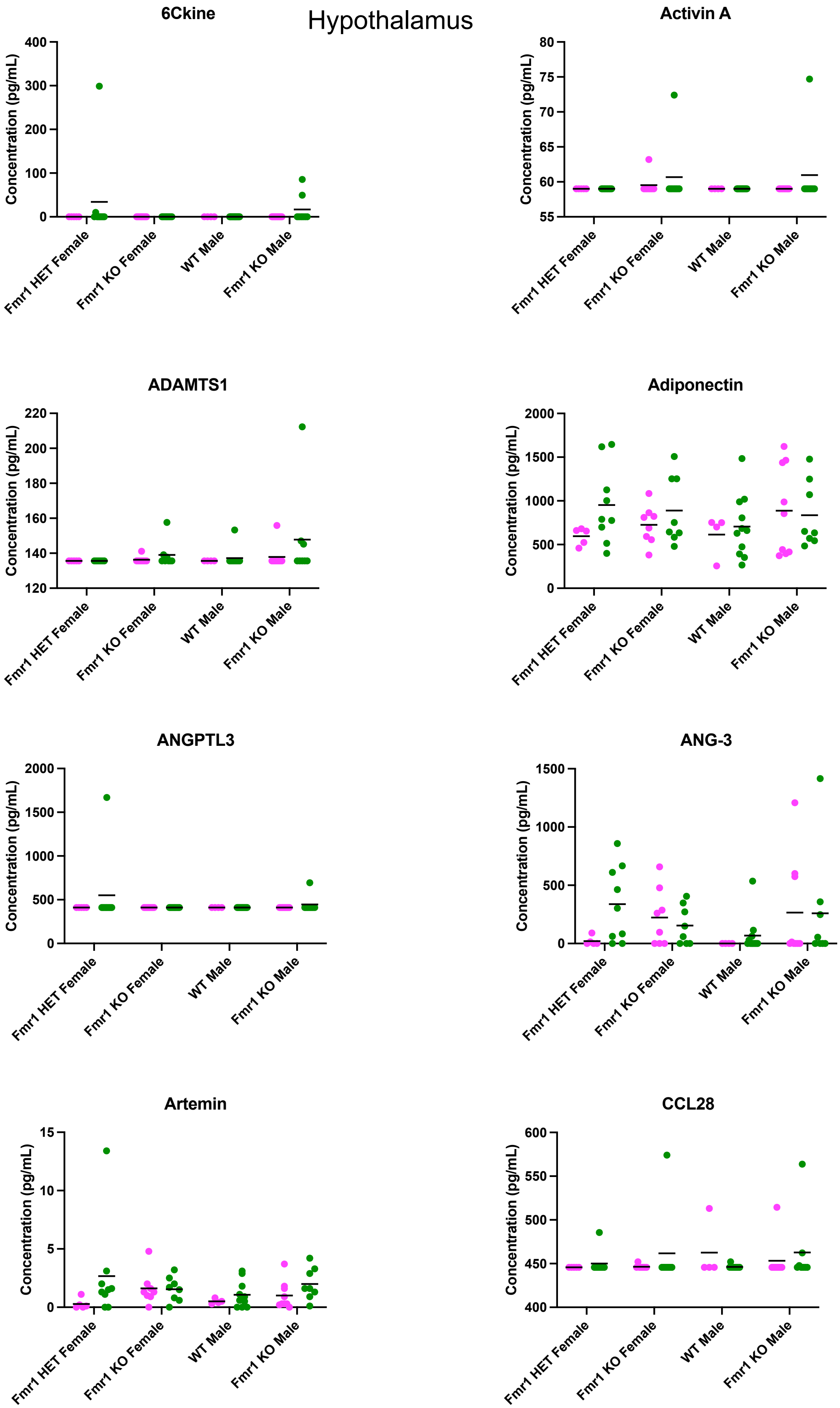

Hypothalamus
6Ckine
Activin A
ADAMTS1
Adiponectin
ANGPTL3
ANG-3
Artemin
CCL28
Concentration (pg/mL)
Fmr1 HET Female
Fmr1 KO Female
WT Male
Fmr1 KO Male

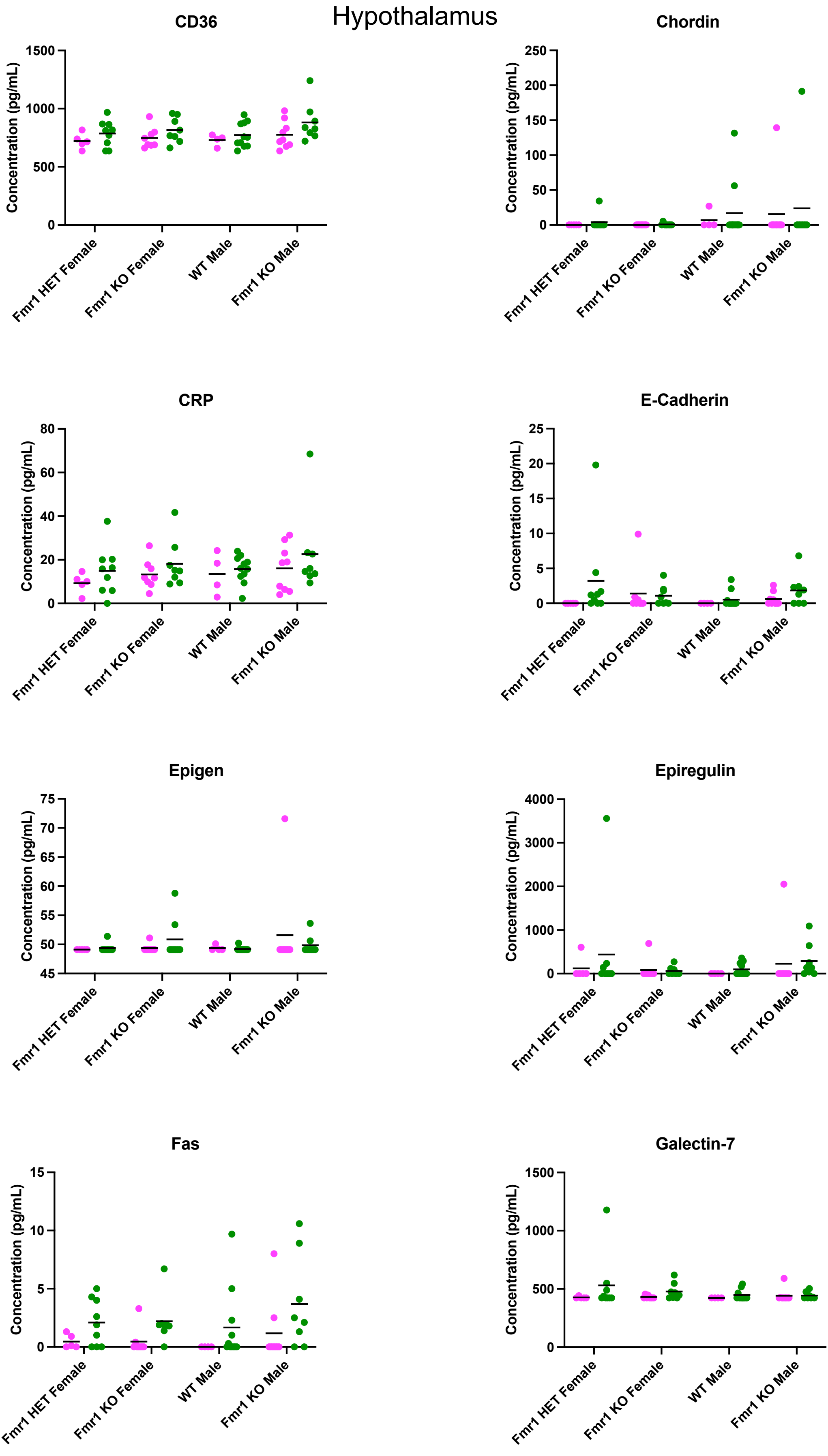
Hypothalamus
CD36
Chordin
CRP
E-Cadherin
Epigen
Epiregulin
Fas
Galectin-7
Concentration (pg/mL)
Fmr1 HET Female
Fmr1 KO Female
WT Male
Fmr1 KO Male

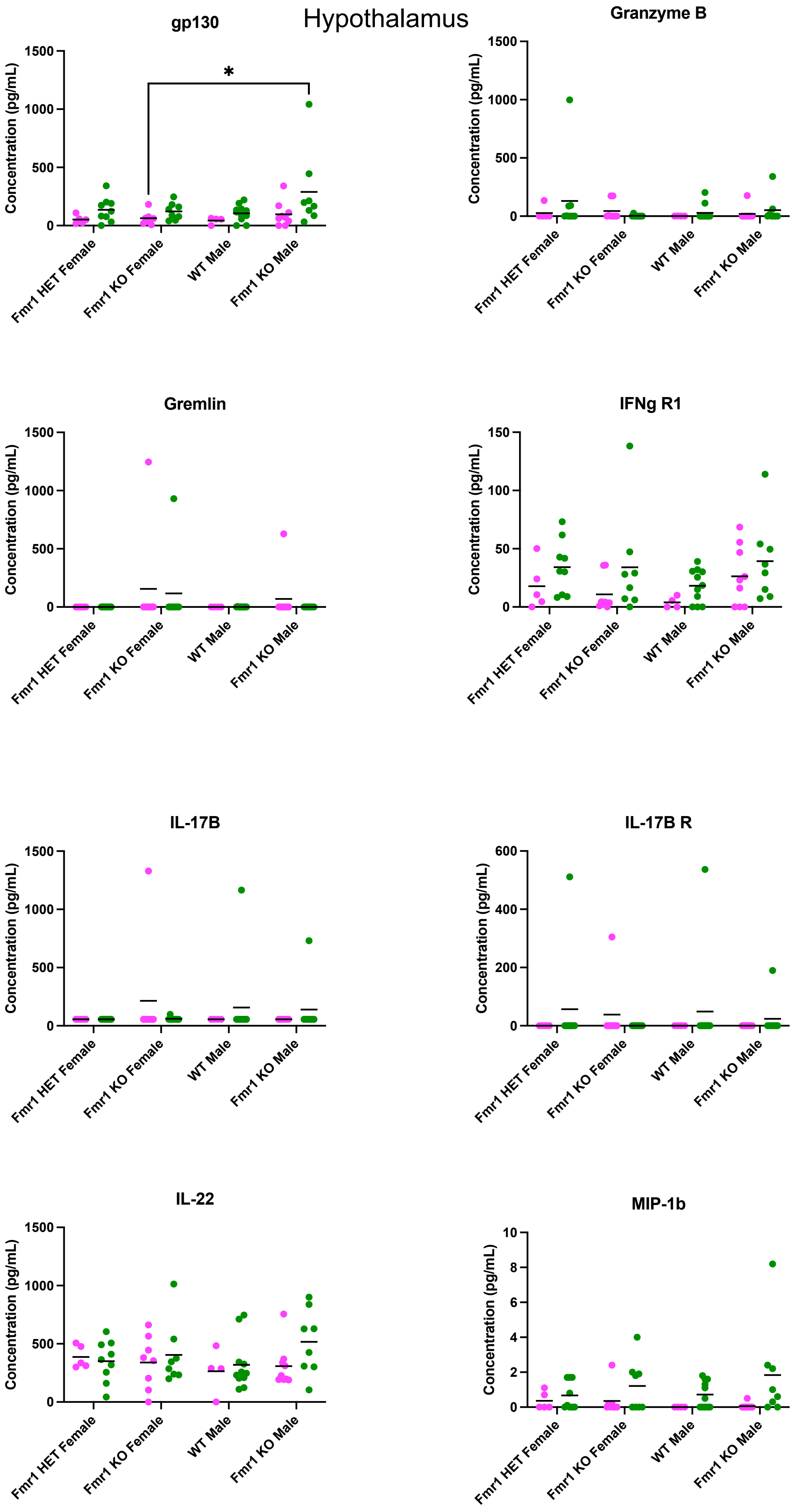

Hypothalamus
gp130
Granzyme B
Gremlin
IFNg R1
IL-17B
IL-17B R
IL-22
MIP-1b
Concentration (pg/mL)
*
0
500
1000
1500
50
100
150
200
400
600
2
4
6
8
10
Fmr1 HET Female
Fmr1 KO Female
WT Male
Fmr1 KO Male

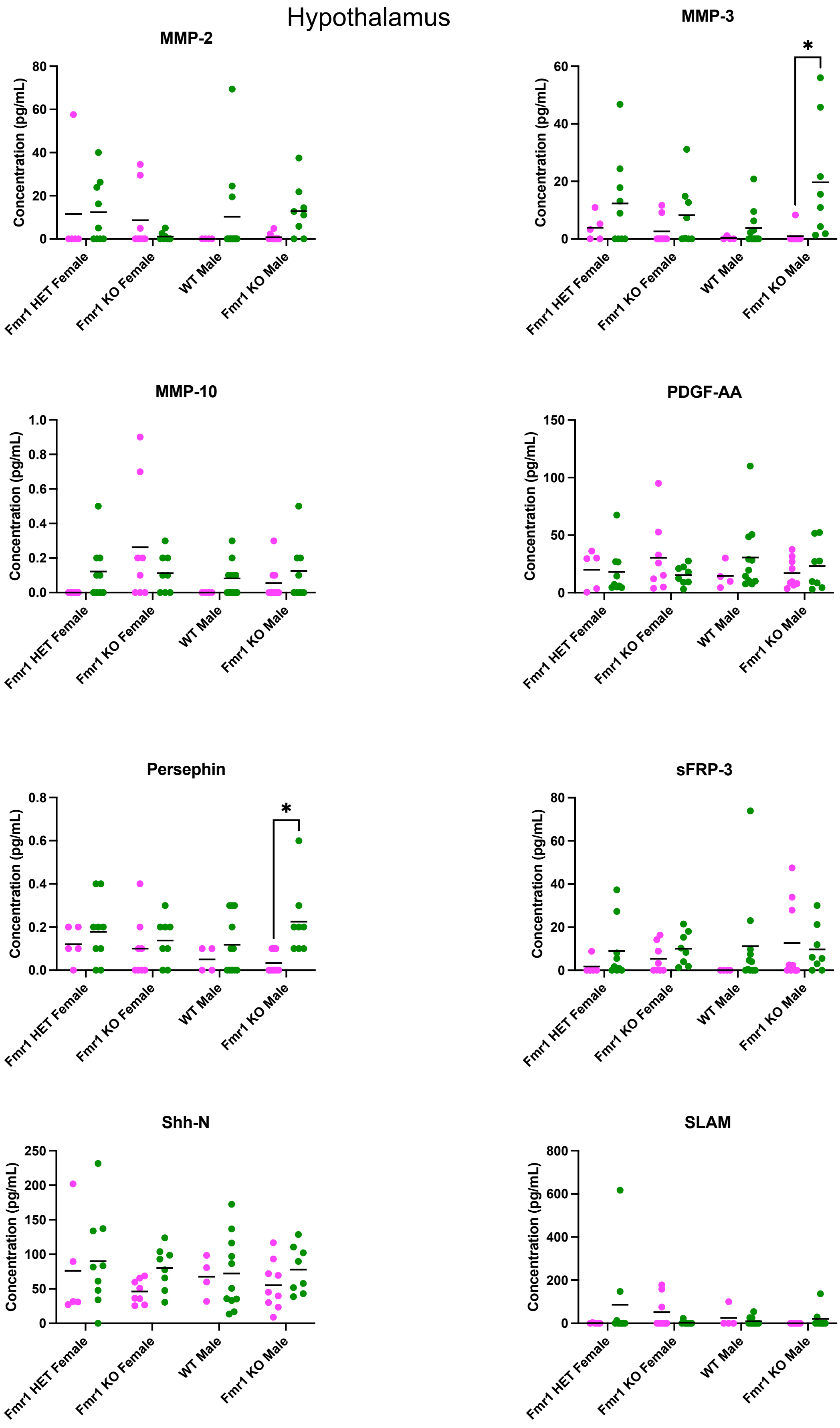
Hypothalamus
MMP-2
MMP-3
MMP-10
PDGF-AA
Persephin
sFRP-3
Shh-N
SLAM
Concentration (pg/mL)
Fmr1 HET Female
Fmr1 KO Female
WT Male
Fmr1 KO Male
*
*

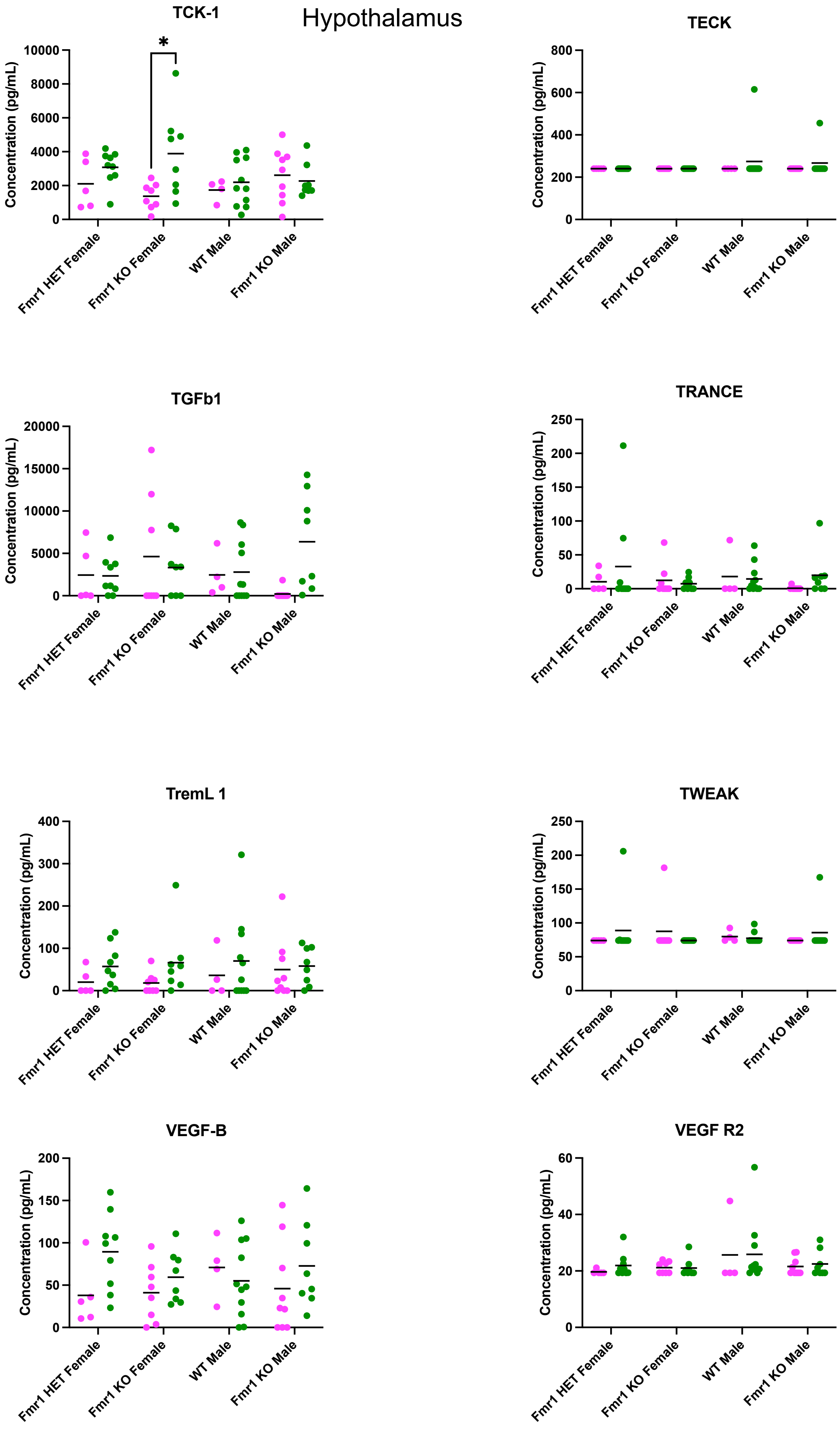
Hypothalamus
TCK-1
*
TECK
TGFb1
TRANCE
TremL 1
TWEAK
VEGF-B
VEGF R2
Concentration (pg/mL)
Fmr1 HET Female
Fmr1 KO Female
WT Male
Fmr1 KO Male
10000
8000
6000
4000
2000
0
800
600
400
200
20000
15000
10000
5000
250
200
150
100
50
400
300
60
40
20

# Plasma

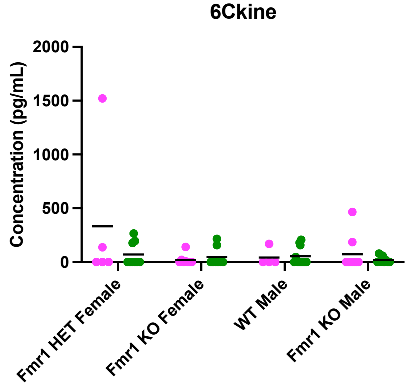
6Ckine
Concentration (pg/mL)
2000
1500
1000
500
0
Fmr1 HET Female
Fmr1 KO Female
WT Male
Fmr1 KO Male

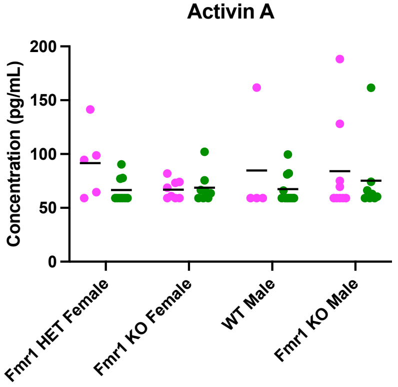
Activin A
Concentration (pg/mL)
200
150
100
50
0
Fmr1 HET Female
Fmr1 KO Female
WT Male
Fmr1 KO Male

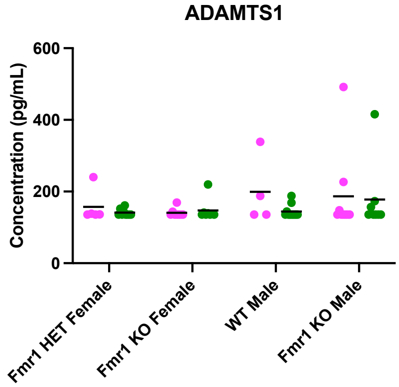
ADAMTS1
Concentration (pg/mL)
600
400
200
0
Fmr1 HET Female
Fmr1 KO Female
WT Male
Fmr1 KO Male

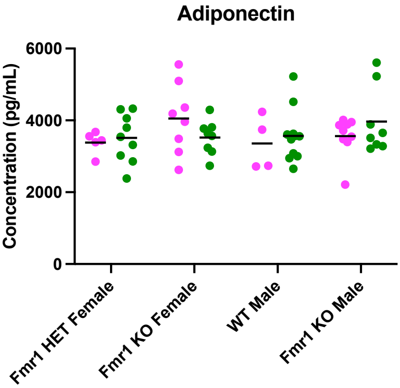
Adiponectin
Concentration (pg/mL)
6000
4000
2000
0
Fmr1 HET Female
Fmr1 KO Female
WT Male
Fmr1 KO Male

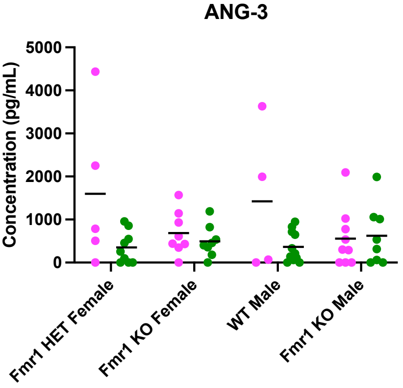
ANG-3
Concentration (pg/mL)
5000
4000
3000
2000
1000
0
Fmr1 HET Female
Fmr1 KO Female
WT Male
Fmr1 KO Male

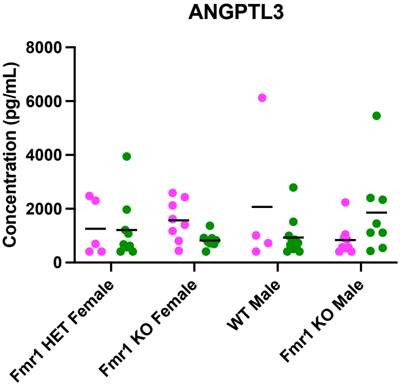
ANGPTL3
Concentration (pg/mL)
8000
6000
4000
2000
0
Fmr1 HET Female
Fmr1 KO Female
WT Male
Fmr1 KO Male

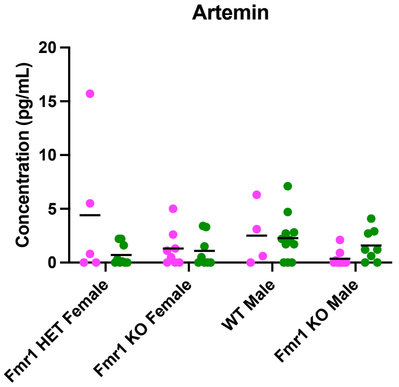
Artemin
Concentration (pg/mL)
20
15
10
5
0
Fmr1 HET Female
Fmr1 KO Female
WT Male
Fmr1 KO Male

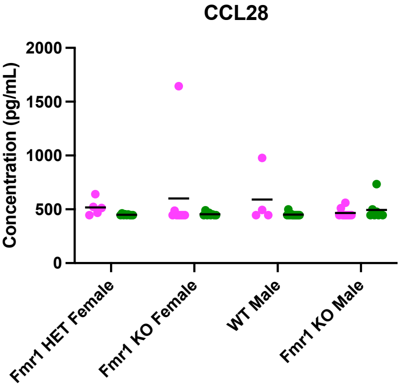
CCL28
Concentration (pg/mL)
2000
1500
1000
500
0
Fmr1 HET Female
Fmr1 KO Female
WT Male
Fmr1 KO Male

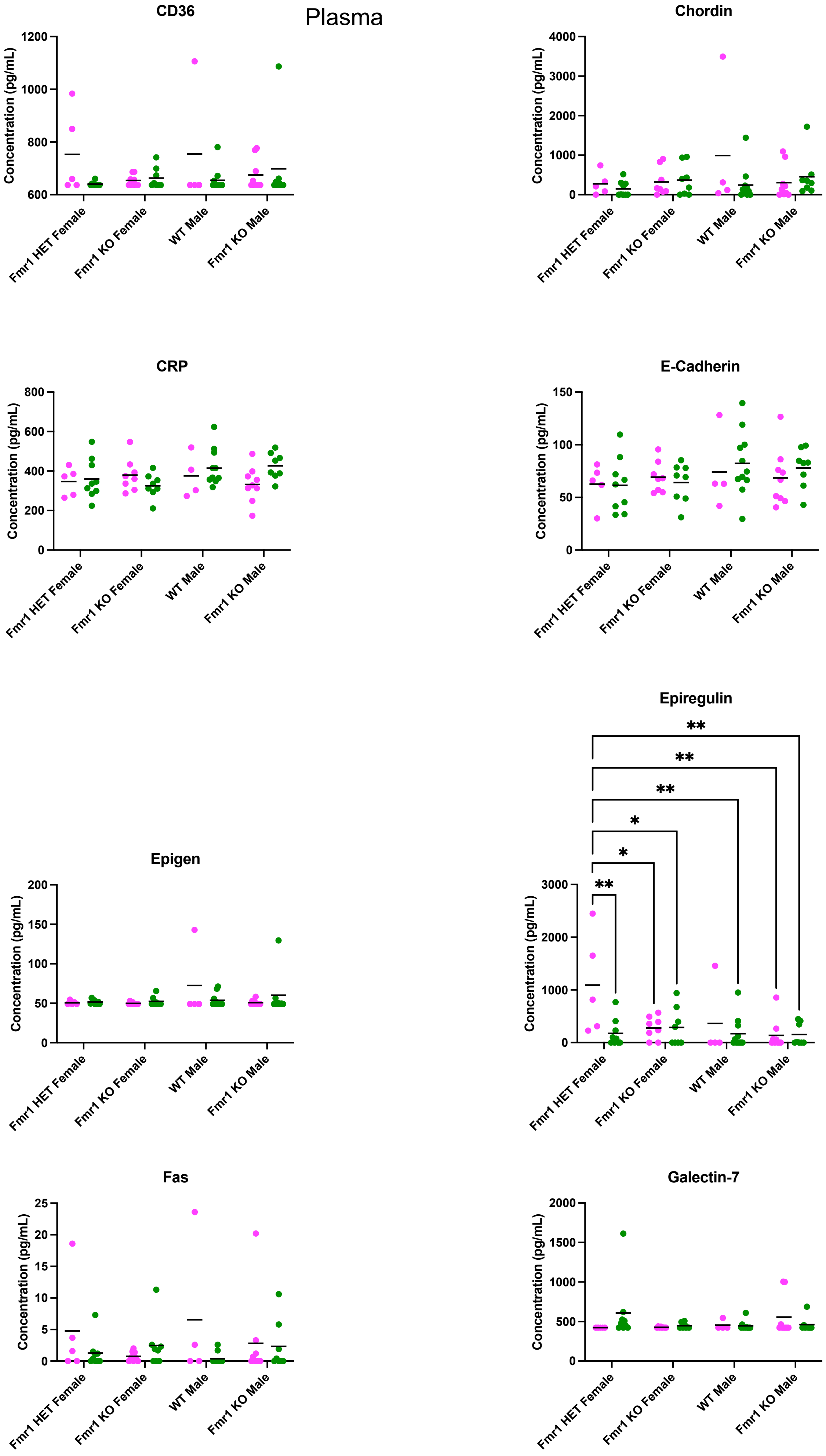
Plasma
CD36
Concentration (pg/mL)
1200
1000
800
600
Fmr1 HET Female
Fmr1 KO Female
WT Male
Fmr1 KO Male
Chordin
Concentration (pg/mL)
4000
3000
2000
1000
0
Fmr1 HET Female
Fmr1 KO Female
WT Male
Fmr1 KO Male
CRP
Concentration (pg/mL)
800
600
400
200
0
Fmr1 HET Female
Fmr1 KO Female
WT Male
Fmr1 KO Male
E-Cadherin
Concentration (pg/mL)
150
100
50
0
Fmr1 HET Female
Fmr1 KO Female
WT Male
Fmr1 KO Male
Epigen
Concentration (pg/mL)
200
150
100
50
0
Fmr1 HET Female
Fmr1 KO Female
WT Male
Fmr1 KO Male
Epiregulin
**
**
**
*
*
**
Concentration (pg/mL)
3000
2000
1000
0
Fmr1 HET Female
Fmr1 KO Female
WT Male
Fmr1 KO Male
Fas
Concentration (pg/mL)
25
20
15
10
5
0
Fmr1 HET Female
Fmr1 KO Female
WT Male
Fmr1 KO Male
Galectin-7
Concentration (pg/mL)
2000
1500
1000
500
0
Fmr1 HET Female
Fmr1 KO Female
WT Male
Fmr1 KO Male

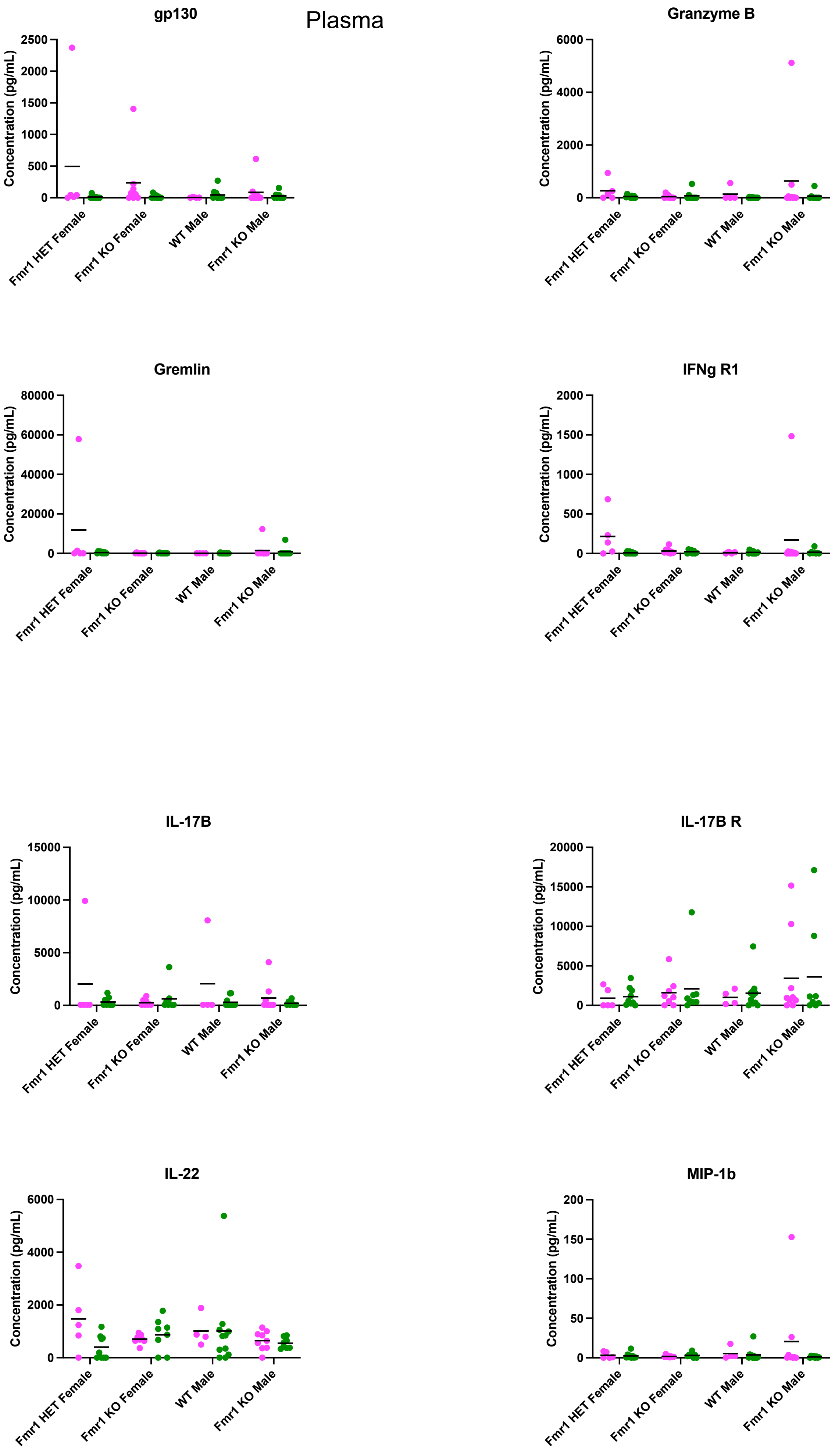
Plasma
gp130
Granzyme B
Gremlin
IFNg R1
IL-17B
IL-17B R
IL-22
MIP-1b
Concentration (pg/mL)
0
500
1000
1500
2000
2500
2000
4000
6000
20000
40000
60000
80000
5000
10000
15000
50
100
150
200
Fmr1 HET Female
Fmr1 KO Female
WT Male
Fmr1 KO Male

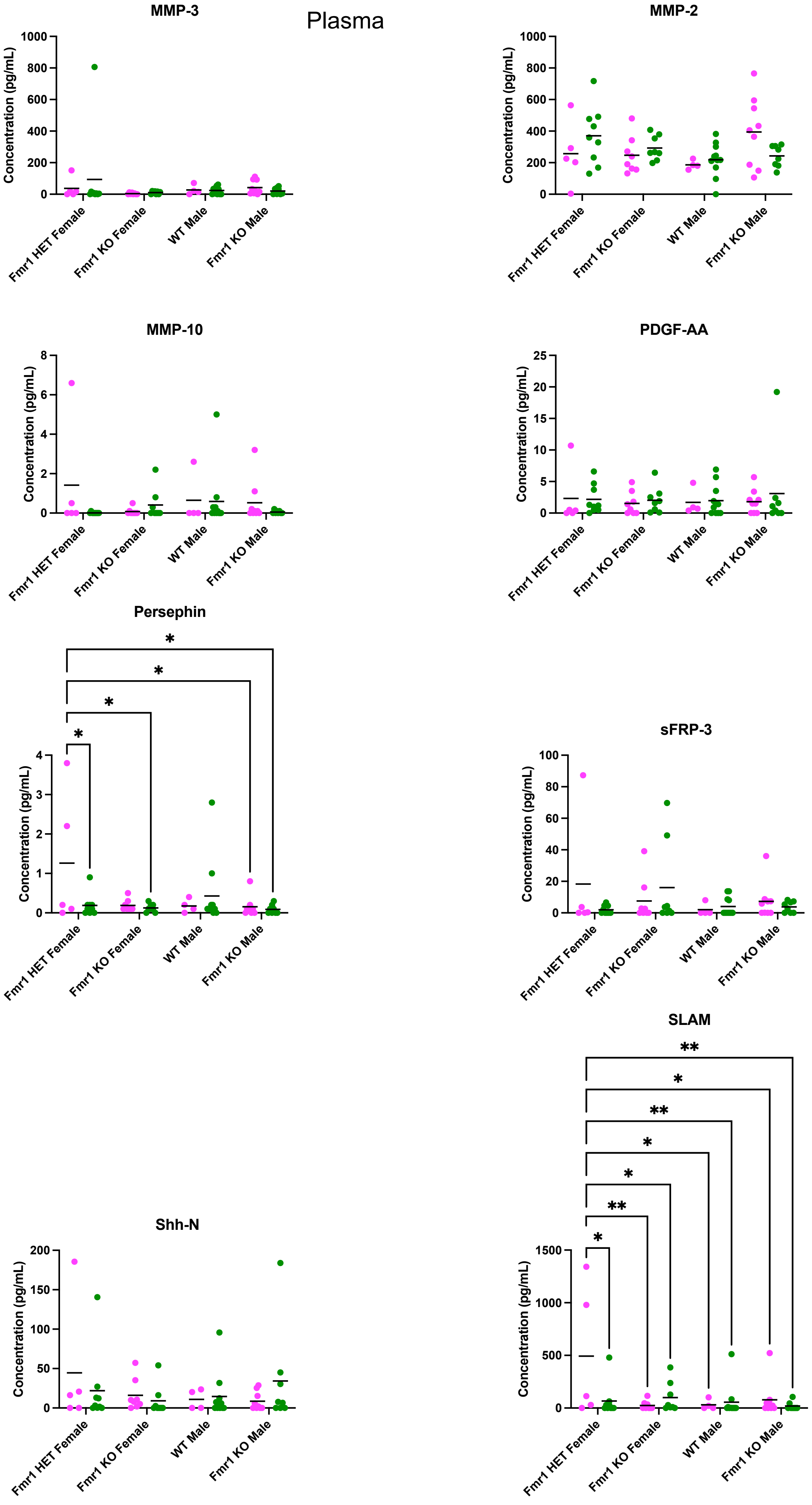
Plasma
MMP-3
MMP-2
MMP-10
PDGF-AA
Persephin
sFRP-3
SLAM
Shh-N
Concentration (pg/mL)
Fmr1 HET Female
Fmr1 KO Female
WT Male
Fmr1 KO Male
0
200
400
600
800
1000
2
4
6
8
5
10
15
20
25
1
3
20
40
60
80
100
50
150
200
500
1000
1500
*
**

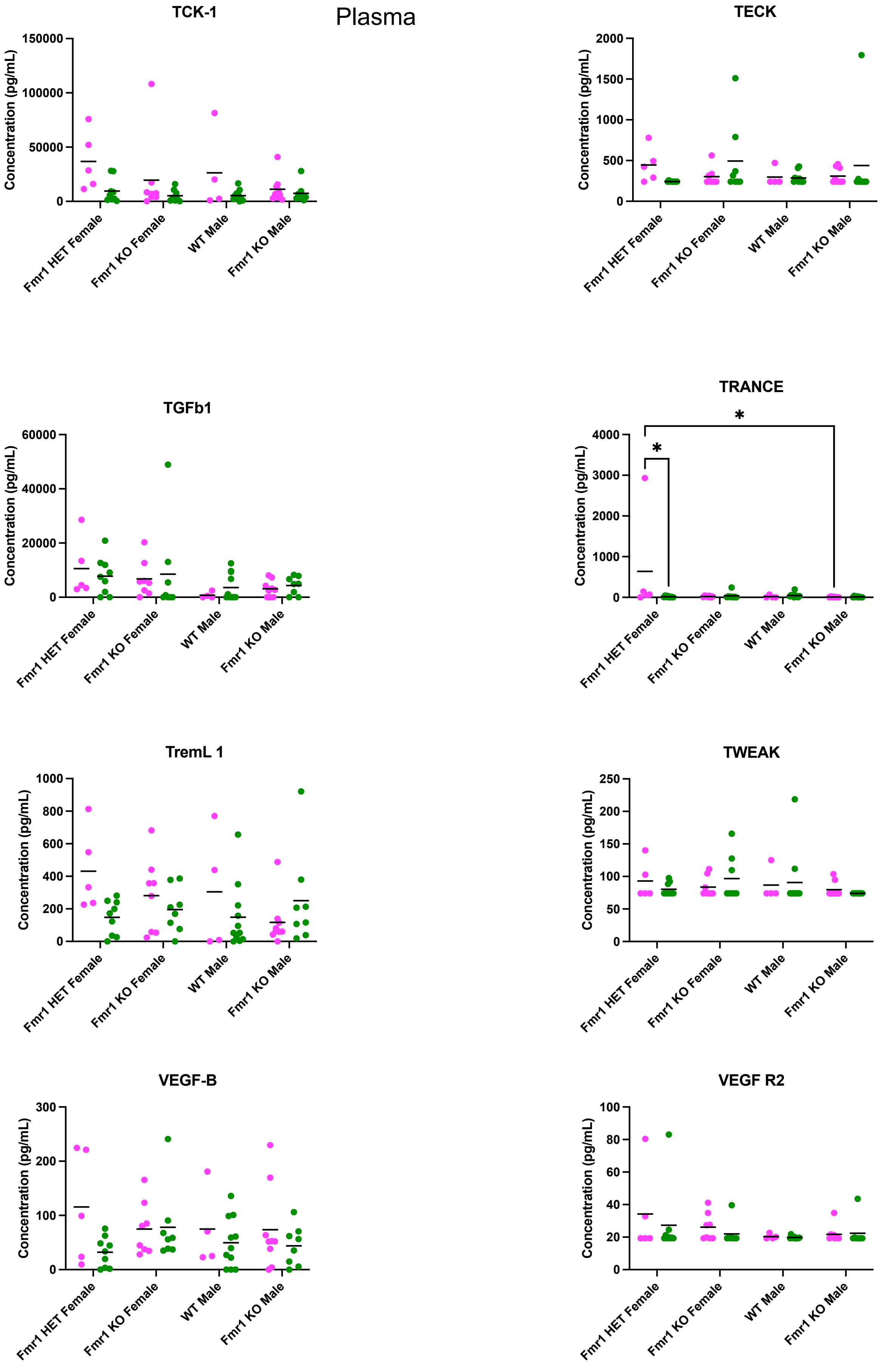
Plasma
TCK-1
TECK
TGFb1
TRANCE
*
*
TremL 1
TWEAK
VEGF-B
VEGF R2
Concentration (pg/mL)
0
50000
100000
150000
500
1000
1500
2000
20000
40000
60000
1000
2000
3000
4000
200
400
600
800
1000
50
100
150
200
250
100
200
300
20
40
60
80
100
Fmr1 HET Female
Fmr1 KO Female
WT Male
Fmr1 KO Male
