# Supplementary material for: Soy Protein Isolate Affects Blood and Brain Biomarker Expression in a Mouse Model of Fragile X
Source: Int J Mol Sci. 2025 Jun 26;26(13):6137. doi: 10.3390/ijms26136137 (PMC12250412; doi:10.3390/ijms26136137)
Supplement: Supplementary file 1 [file ijms-26-06137-s001.zip › Supplementary File S7b Array 9 Graphs.pdf]

Supplementary File S7. Protein expression of Array 9 targets as function of *Fmr1* genotype and AIN-93G diets. Mice on AIN-93G/cas (colored pink) included n=5 *Fmr1*^{HET} female, n=8 *Fmr1*^{KO} female, n=4 WT male and n=9 *Fmr1*^{KO} male. Mice on AIN-93G/soy (colored green) included n=9 *Fmr1*^{HET} female, n=8 *Fmr1*^{KO} female, n=11 WT male and n=8 *Fmr1*^{KO} male. The average concentration in cortex, hippocampus, hypothalamus and plasma in pg/mL was plotted versus genotype. Statistics were determined by 2-way ANOVA and Tukey's multiple comparison tests denoted by $p < 0.05$ (*), $p < 0.01$ (**), $p < 0.001$ (***) and $p < 0.0001$ (****).

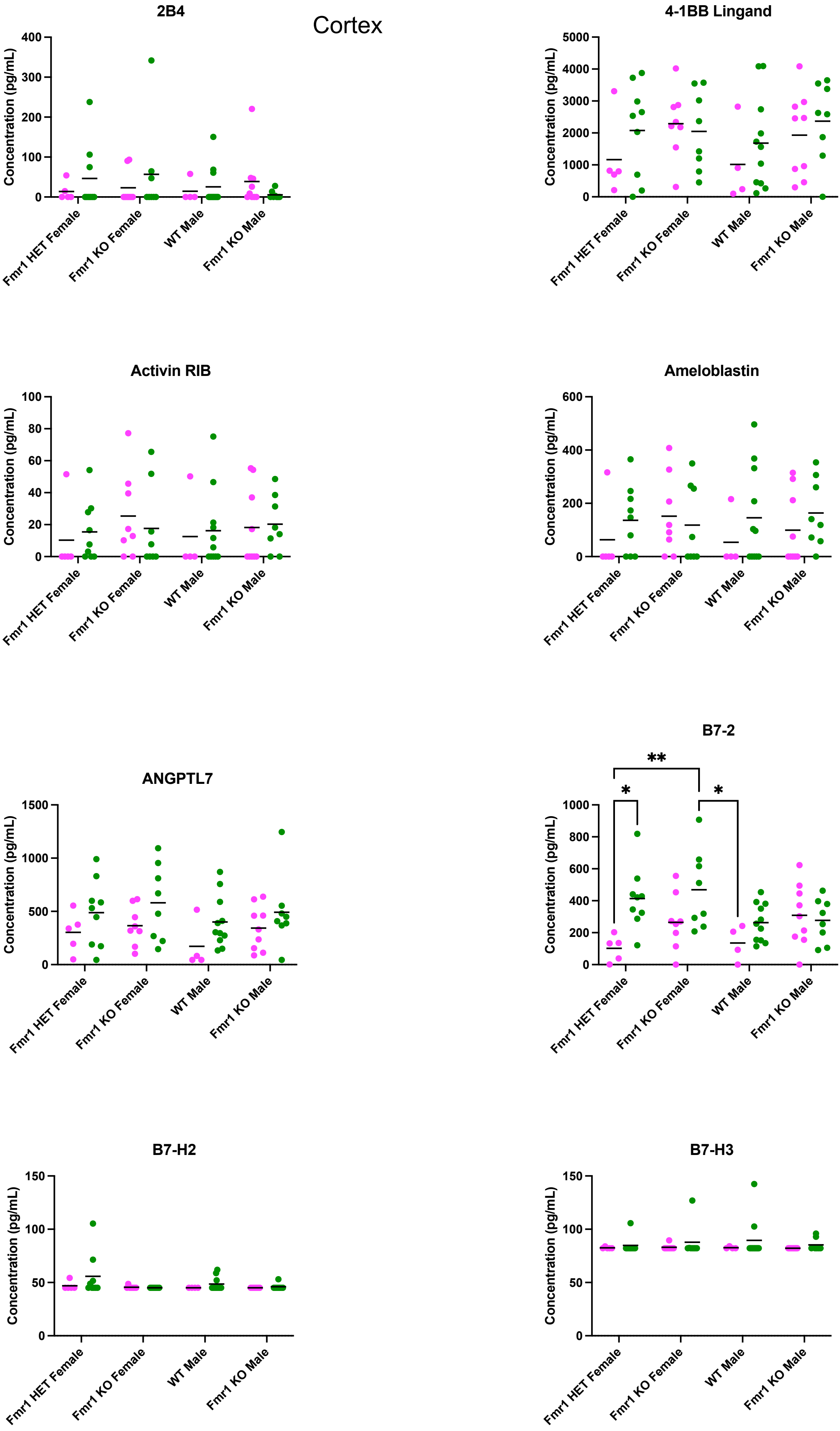
Cortex
2B4
4-1BB Lingand
Activin RIB
Ameloblastin
ANGPTL7
B7-2
B7-H2
B7-H3
Concentration (pg/mL)
Fmr1 HET Female
Fmr1 KO Female
WT Male
Fmr1 KO Male
**
*
*

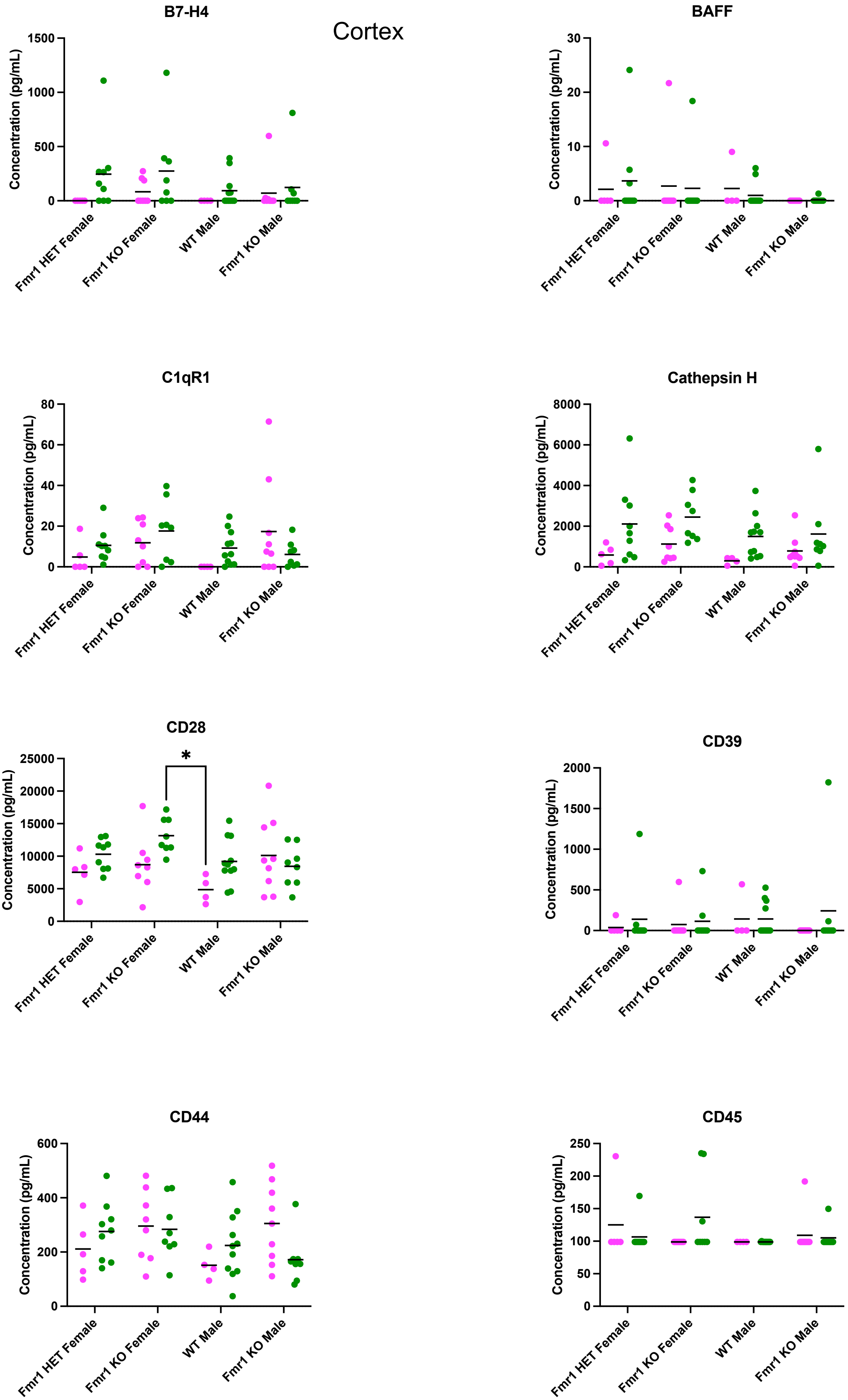
Cortex
B7-H4
BAFF
C1qR1
Cathepsin H
CD28
CD39
CD44
CD45
Concentration (pg/mL)
Fmr1 HET Female
Fmr1 KO Female
WT Male
Fmr1 KO Male
*
0
500
1000
1500
10
20
30
40
60
80
2000
4000
6000
8000
5000
10000
15000
20000
25000
200
400
600
50
100
150
200
250

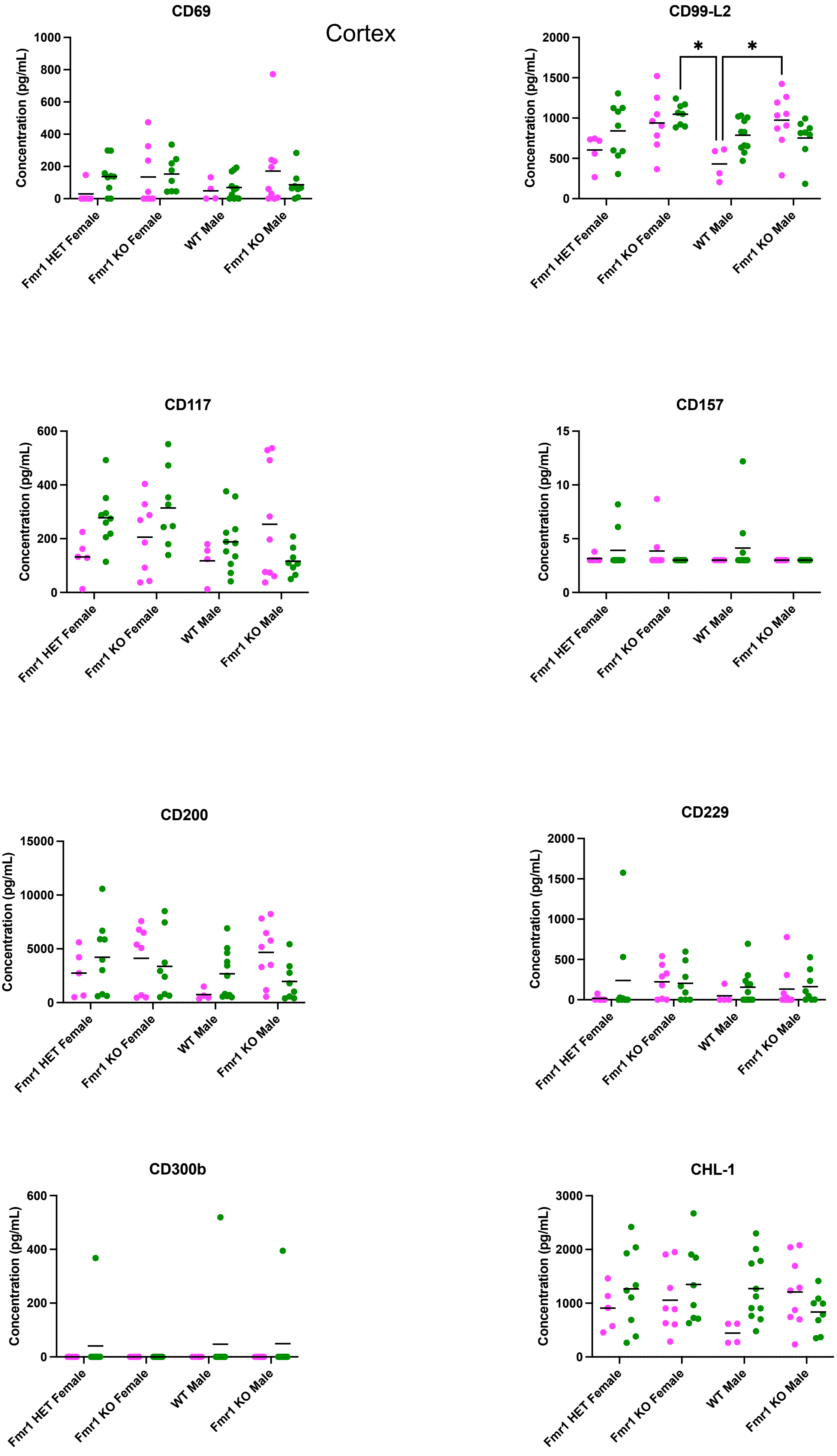
Cortex
CD69
CD99-L2
CD117
CD157
CD200
CD229
CD300b
CHL-1
Concentration (pg/mL)
Fmr1 HET Female
Fmr1 KO Female
WT Male
Fmr1 KO Male
*
*

Cortex

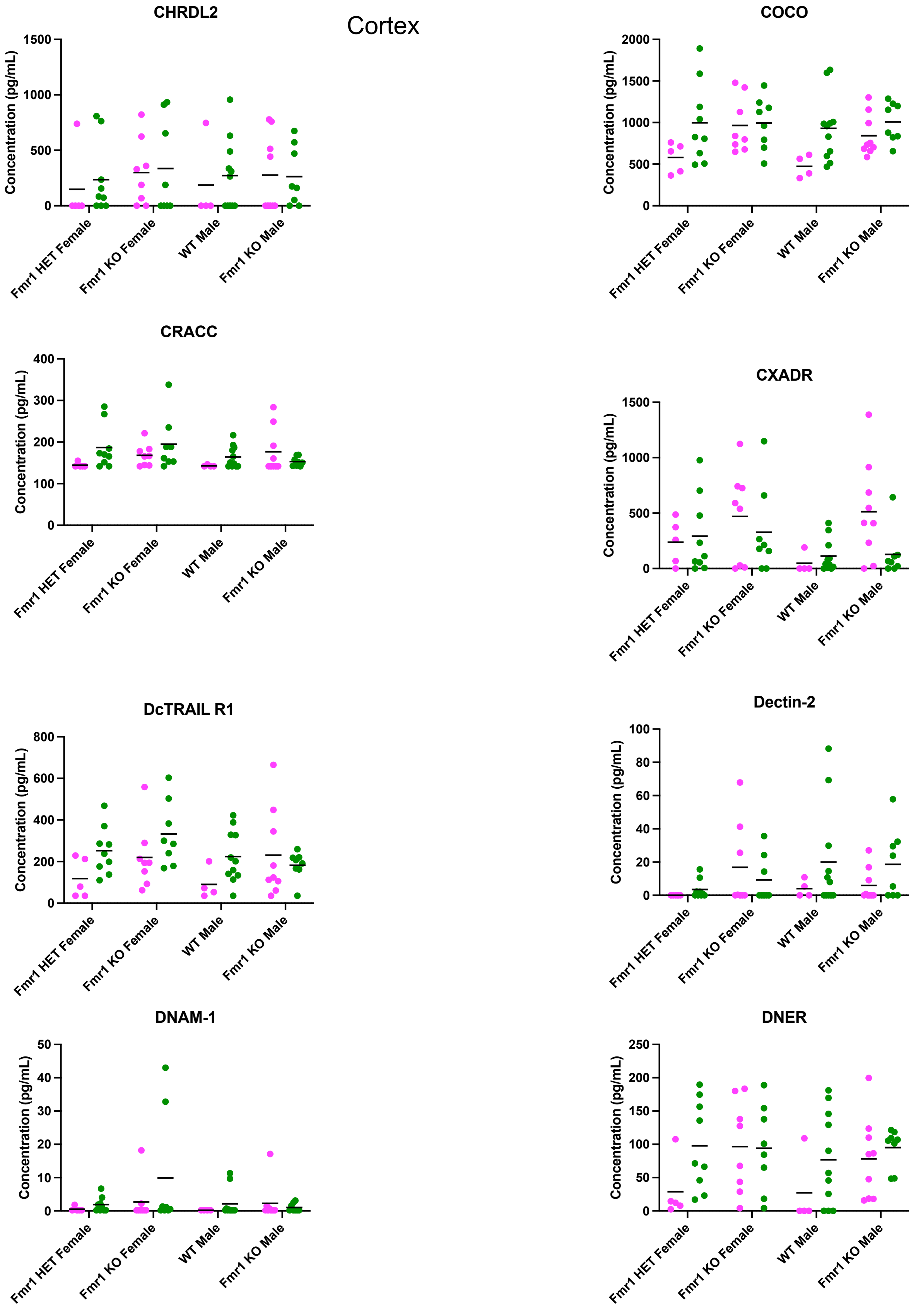

CHRDL2
COCO
CRACC
CXADR
DcTRAIL R1
Dectin-2
DNAM-1
DNER
Concentration (pg/mL)
Fmr1 HET Female
Fmr1 KO Female
WT Male
Fmr1 KO Male

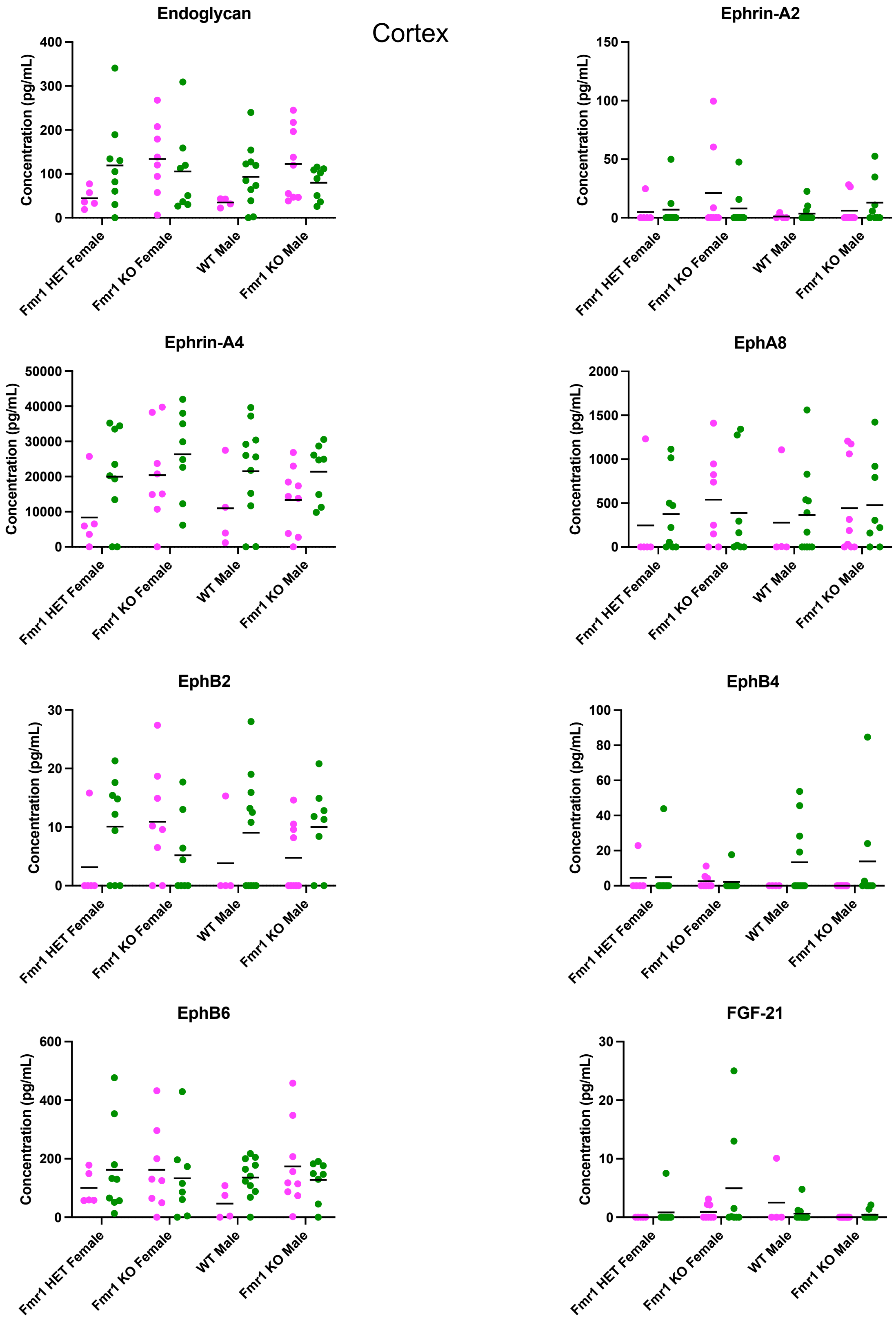
Cortex
Endoglycan
Ephrin-A2
Ephrin-A4
EphA8
EphB2
EphB4
EphB6
FGF-21
Concentration (pg/mL)
Fmr1 HET Female
Fmr1 KO Female
WT Male
Fmr1 KO Male

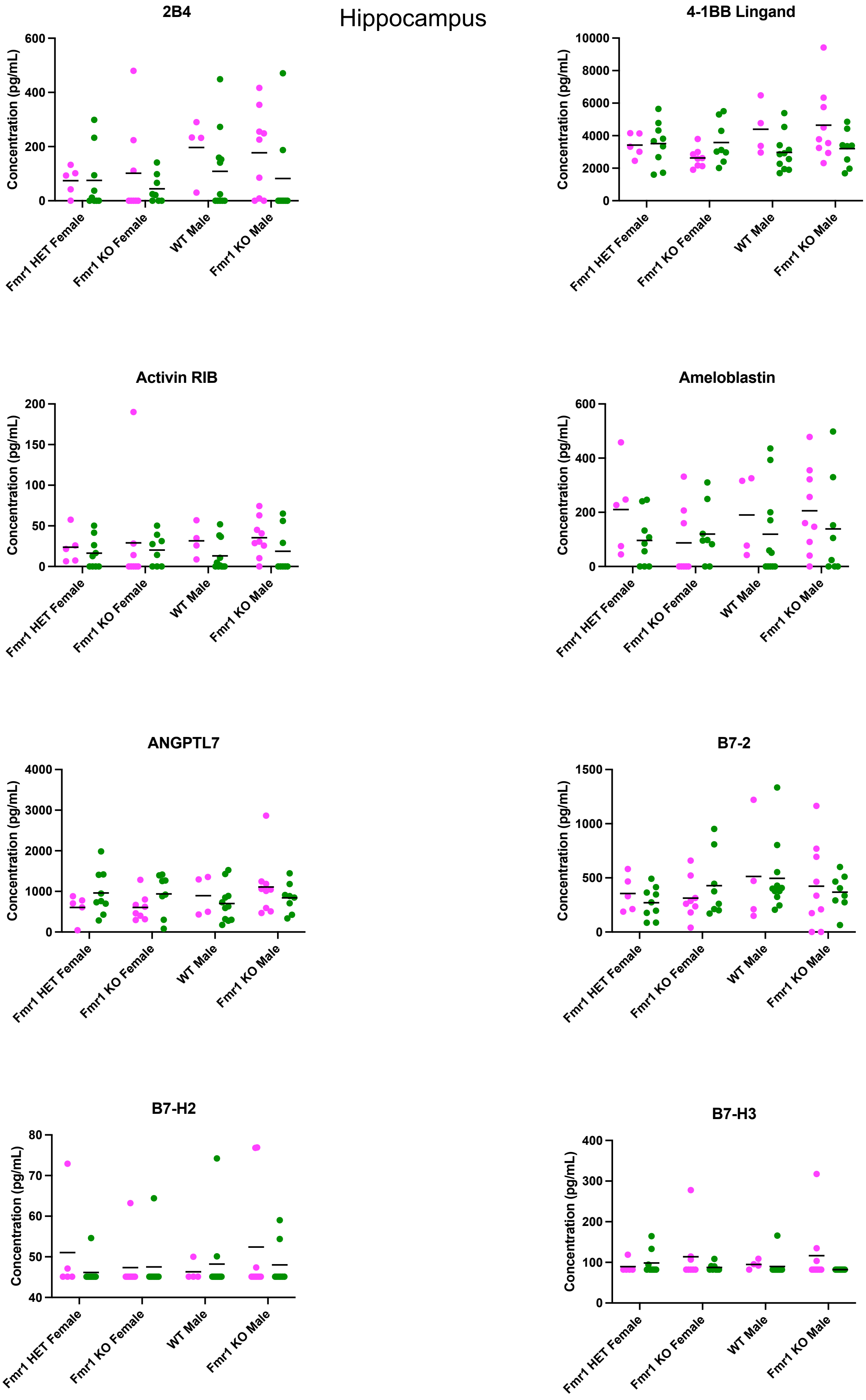
Hippocampus
2B4
4-1BB Lingand
Activin RIB
Ameloblastin
ANGPTL7
B7-2
B7-H2
B7-H3
Concentration (pg/mL)
Fmr1 HET Female
Fmr1 KO Female
WT Male
Fmr1 KO Male

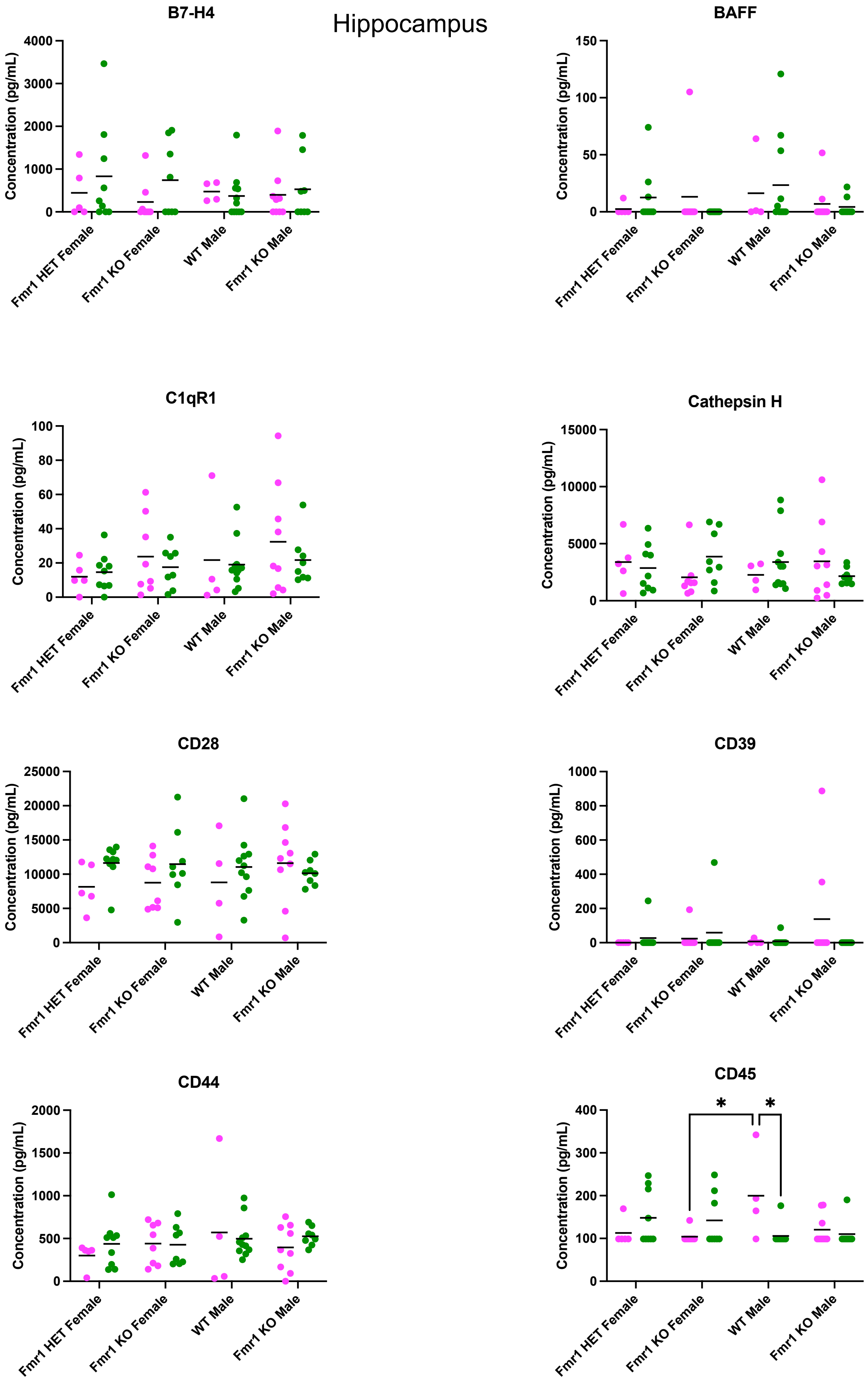
Hippocampus
B7-H4
BAFF
C1qR1
Cathepsin H
CD28
CD39
CD44
CD45
Concentration (pg/mL)
Fmr1 HET Female
Fmr1 KO Female
WT Male
Fmr1 KO Male
*
*

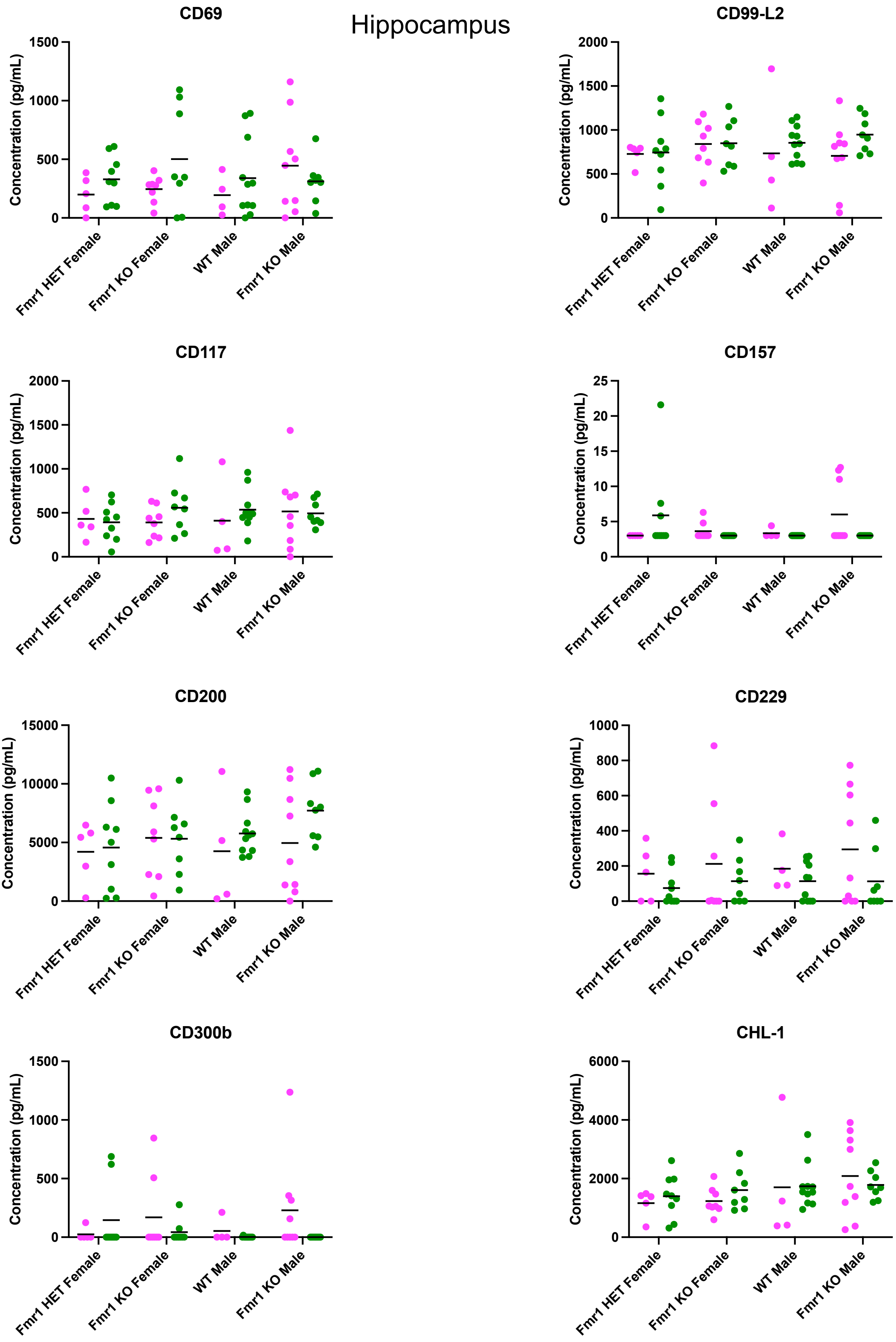
Hippocampus
CD69
CD99-L2
CD117
CD157
CD200
CD229
CD300b
CHL-1
Concentration (pg/mL)
0
500
1000
1500
2000
5
10
15
20
25
5000
10000
15000
200
400
600
800
2000
4000
6000
Fmr1 HET Female
Fmr1 KO Female
WT Male
Fmr1 KO Male

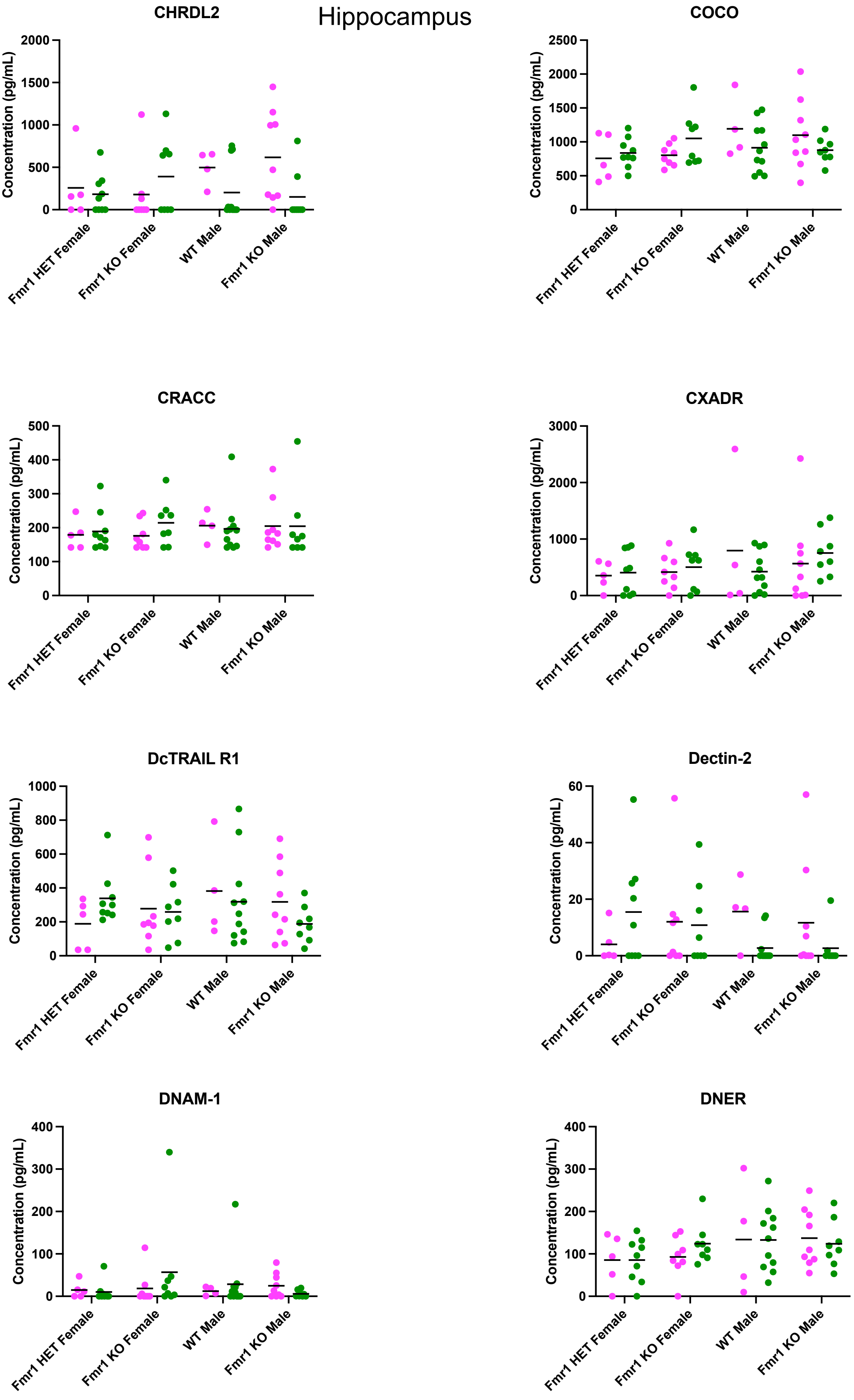
Hippocampus
CHRDL2
COCO
CRACC
CXADR
DcTRAIL R1
Dectin-2
DNAM-1
DNER
Concentration (pg/mL)
0
500
1000
1500
2000
2500
100
200
300
400
3000
600
800
20
40
60
Fmr1 HET Female
Fmr1 KO Female
WT Male
Fmr1 KO Male

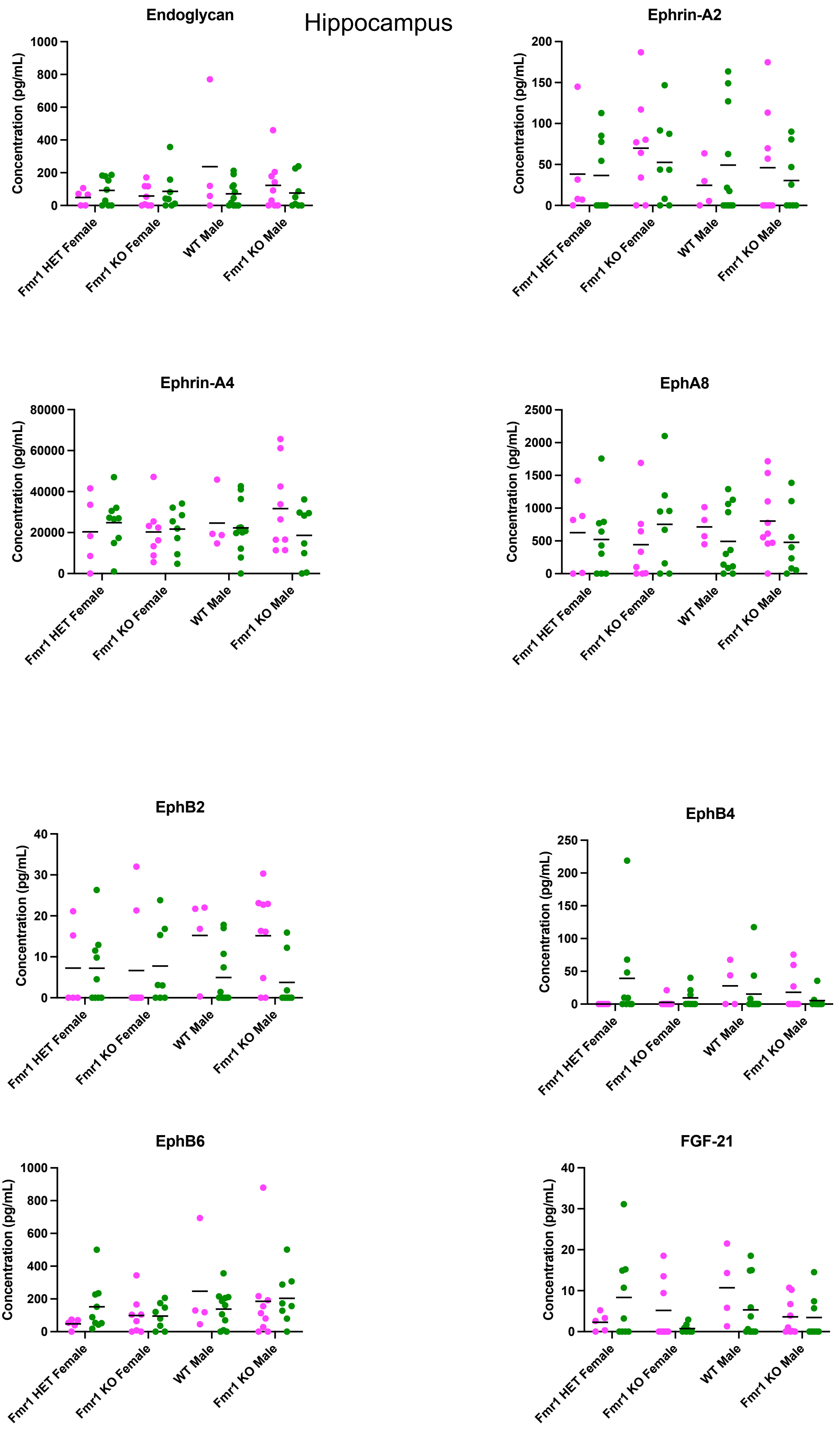

Hippocampus
Endoglycan
Ephrin-A2
Ephrin-A4
EphA8
EphB2
EphB4
EphB6
FGF-21
Concentration (pg/mL)
Fmr1 HET Female
Fmr1 KO Female
WT Male
Fmr1 KO Male

Plasma

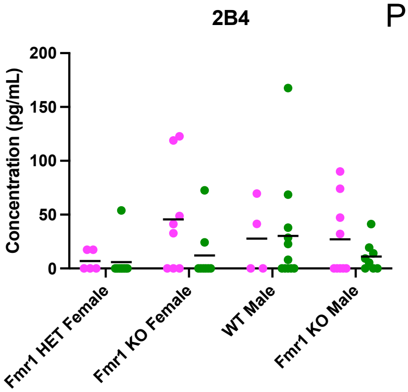
2B4
Concentration (pg/mL)
200
150
100
50
0
Fmr1 HET Female
Fmr1 KO Female
WT Male
Fmr1 KO Male

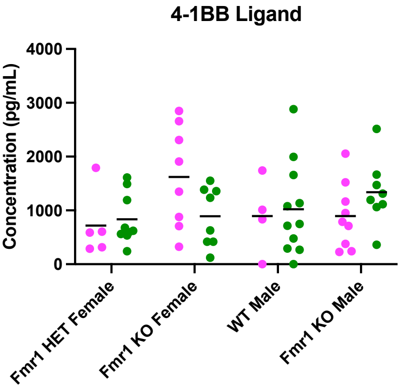
4-1BB Ligand
Concentration (pg/mL)
4000
3000
2000
1000
0
Fmr1 HET Female
Fmr1 KO Female
WT Male
Fmr1 KO Male

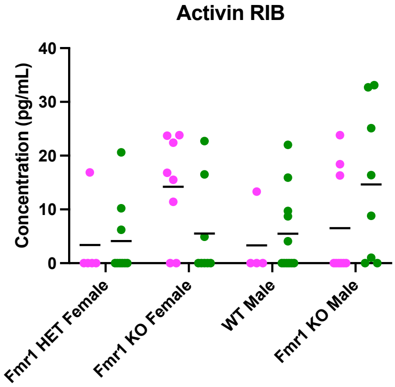
Activin RIB
Concentration (pg/mL)
40
30
20
10
0
Fmr1 HET Female
Fmr1 KO Female
WT Male
Fmr1 KO Male

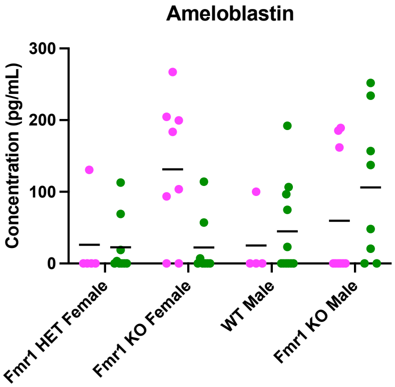
Ameloblastin
Concentration (pg/mL)
300
200
100
0
Fmr1 HET Female
Fmr1 KO Female
WT Male
Fmr1 KO Male

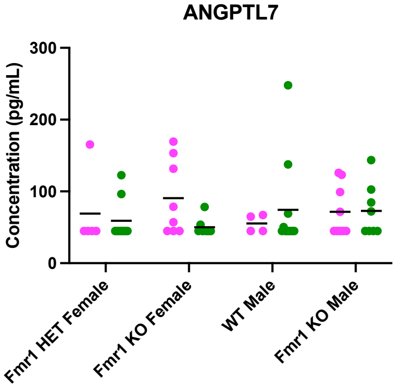
ANGPTL7
Concentration (pg/mL)
300
200
100
0
Fmr1 HET Female
Fmr1 KO Female
WT Male
Fmr1 KO Male

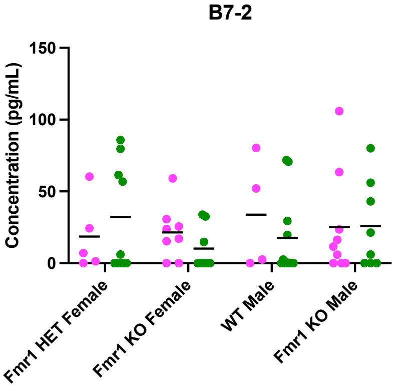
B7-2
Concentration (pg/mL)
150
100
50
0
Fmr1 HET Female
Fmr1 KO Female
WT Male
Fmr1 KO Male

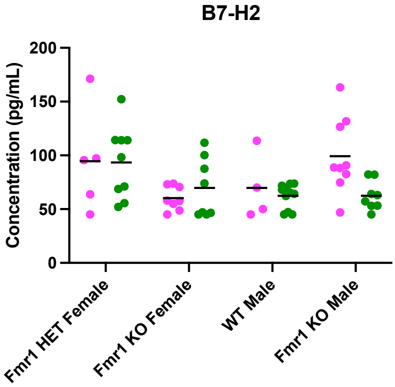
B7-H2
Concentration (pg/mL)
200
150
100
50
0
Fmr1 HET Female
Fmr1 KO Female
WT Male
Fmr1 KO Male

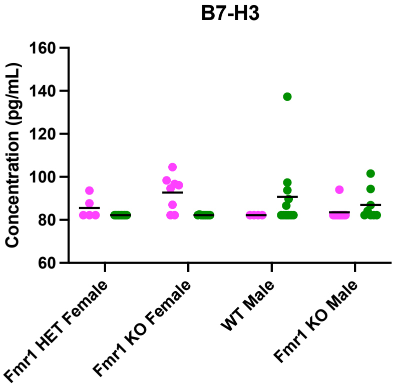
B7-H3
Concentration (pg/mL)
160
140
120
100
80
60
Fmr1 HET Female
Fmr1 KO Female
WT Male
Fmr1 KO Male

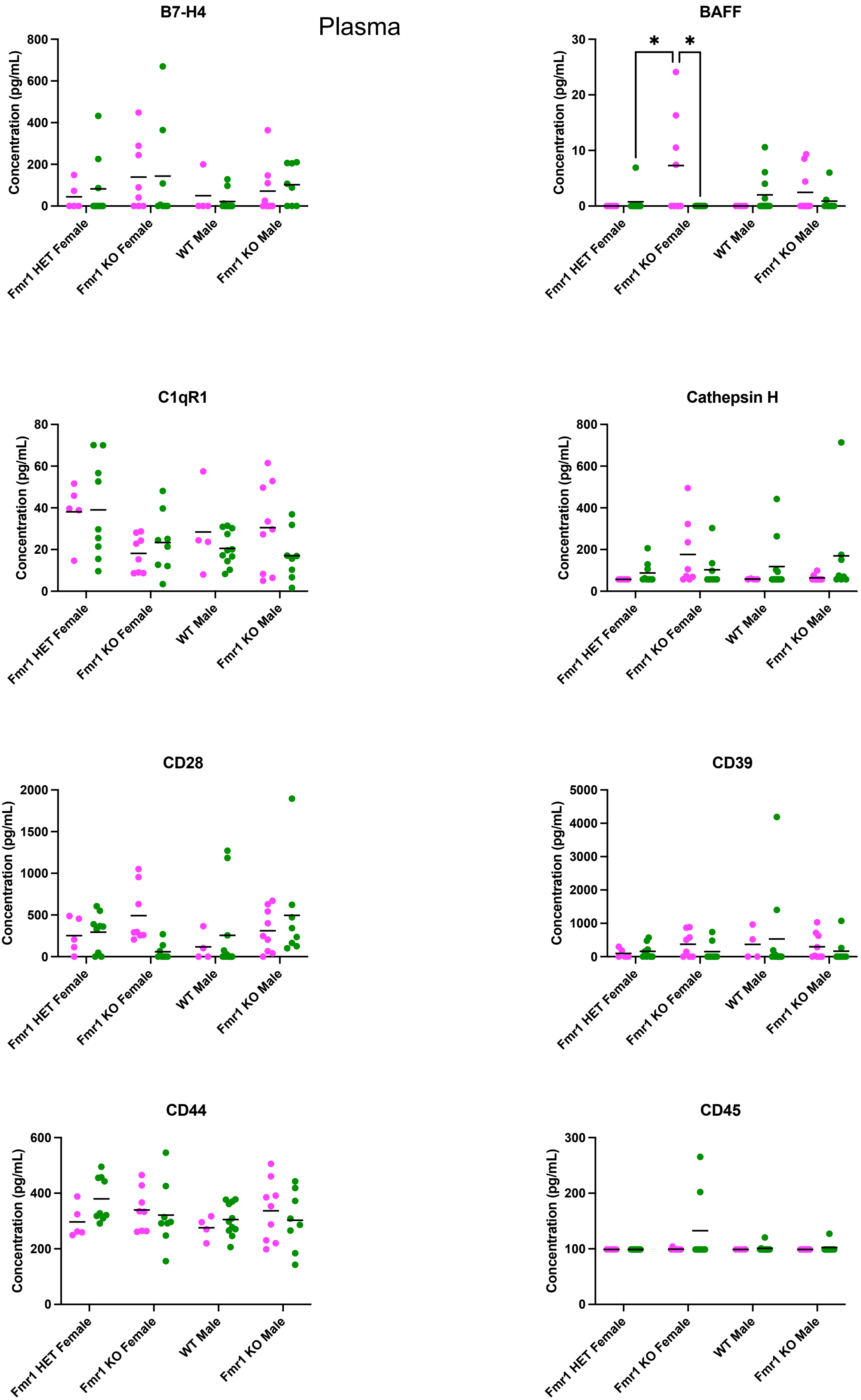
Plasma
B7-H4
BAFF
C1qR1
Cathepsin H
CD28
CD39
CD44
CD45
Concentration (pg/mL)
*
*
Fmr1 HET Female
Fmr1 KO Female
WT Male
Fmr1 KO Male

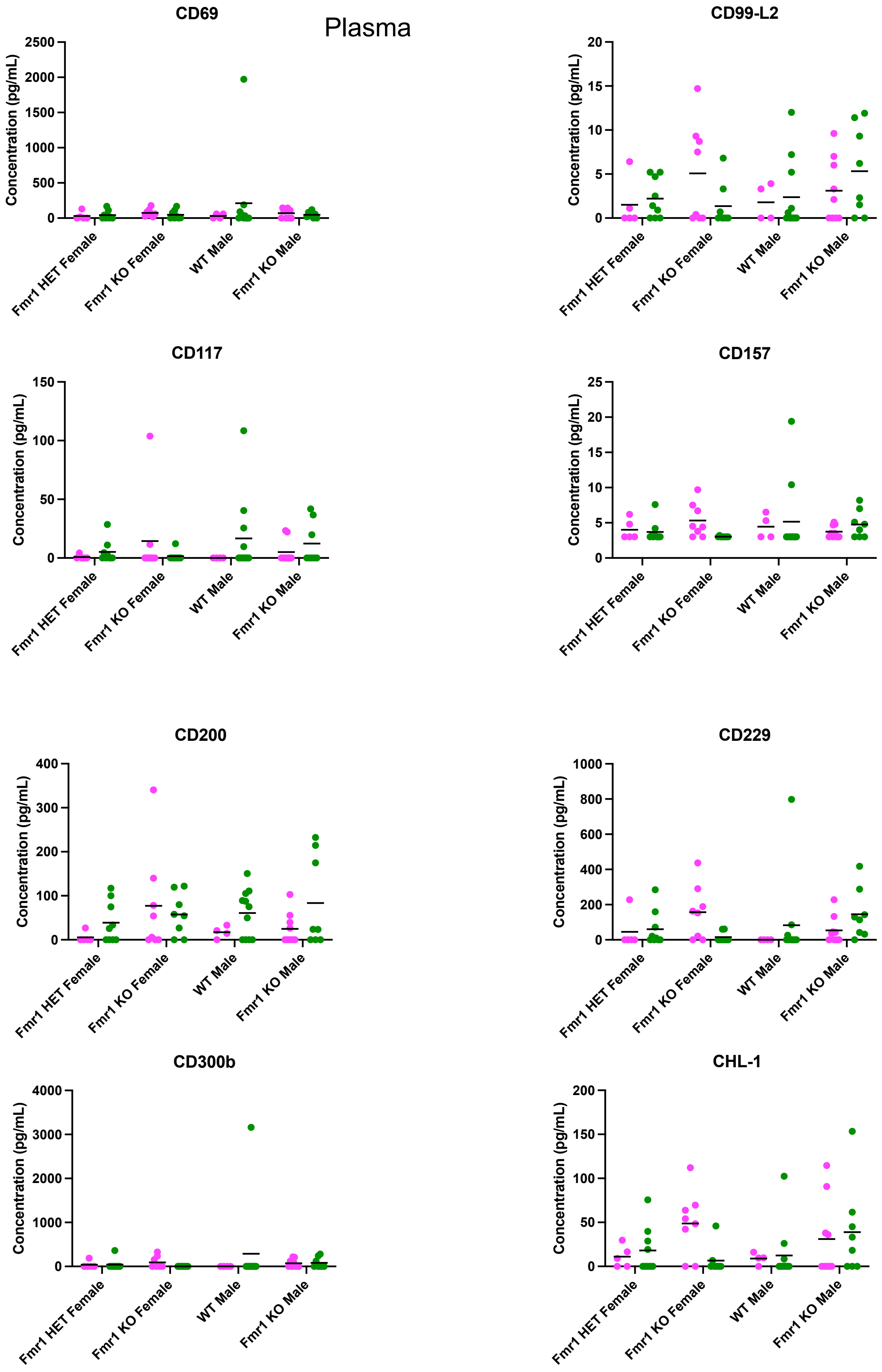
Plasma
CD69
CD99-L2
CD117
CD157
CD200
CD229
CD300b
CHL-1
Concentration (pg/mL)
Fmr1 HET Female
Fmr1 KO Female
WT Male
Fmr1 KO Male

Plasma

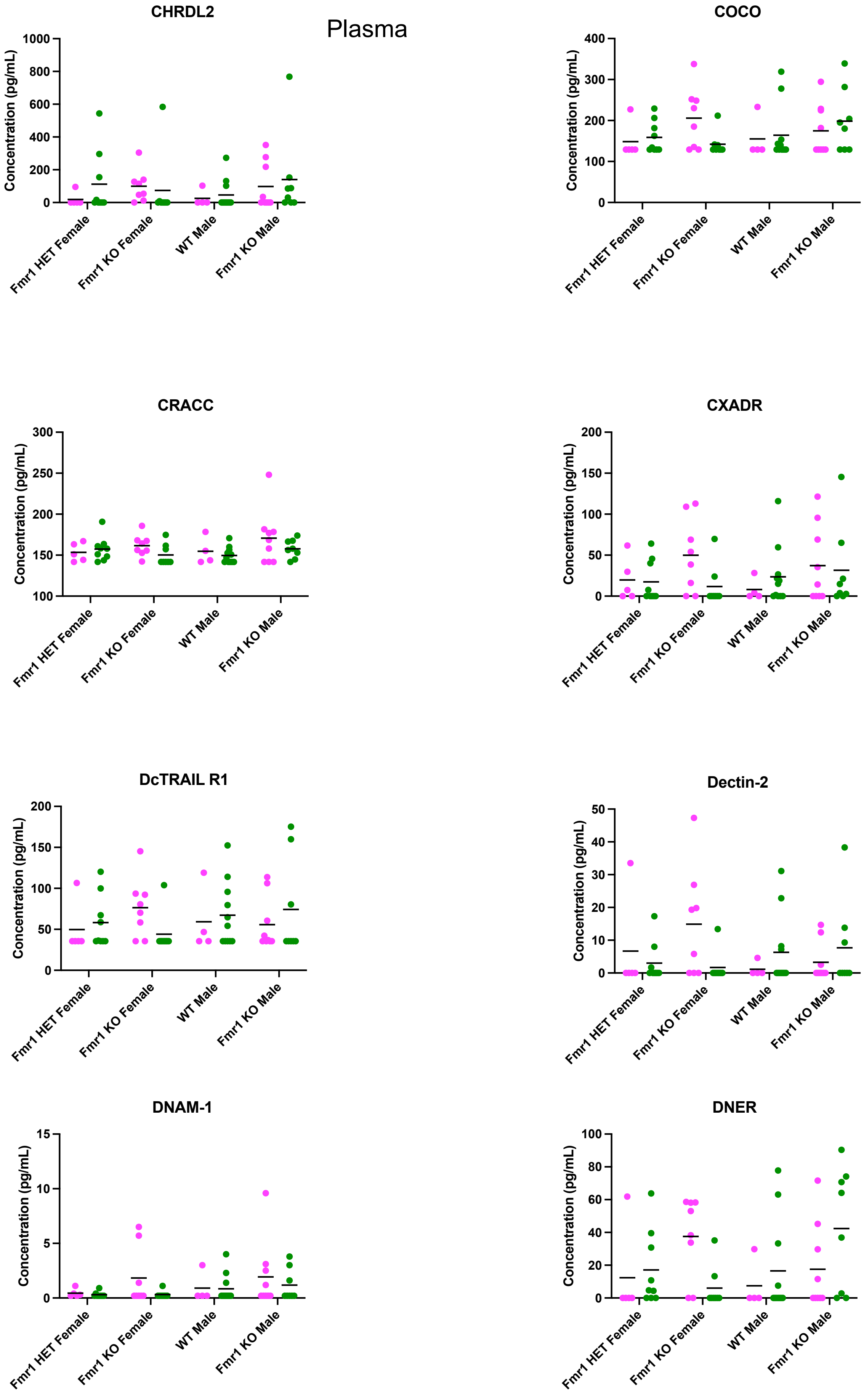
CHRDL2
COCO
CRACC
CXADR
DcTRAIL R1
Dectin-2
DNAM-1
DNER
Concentration (pg/mL)
0
200
400
600
800
1000
100
300
150
250
50
10
15
20
30
40
60
80
5
Fmr1 HET Female
Fmr1 KO Female
WT Male
Fmr1 KO Male

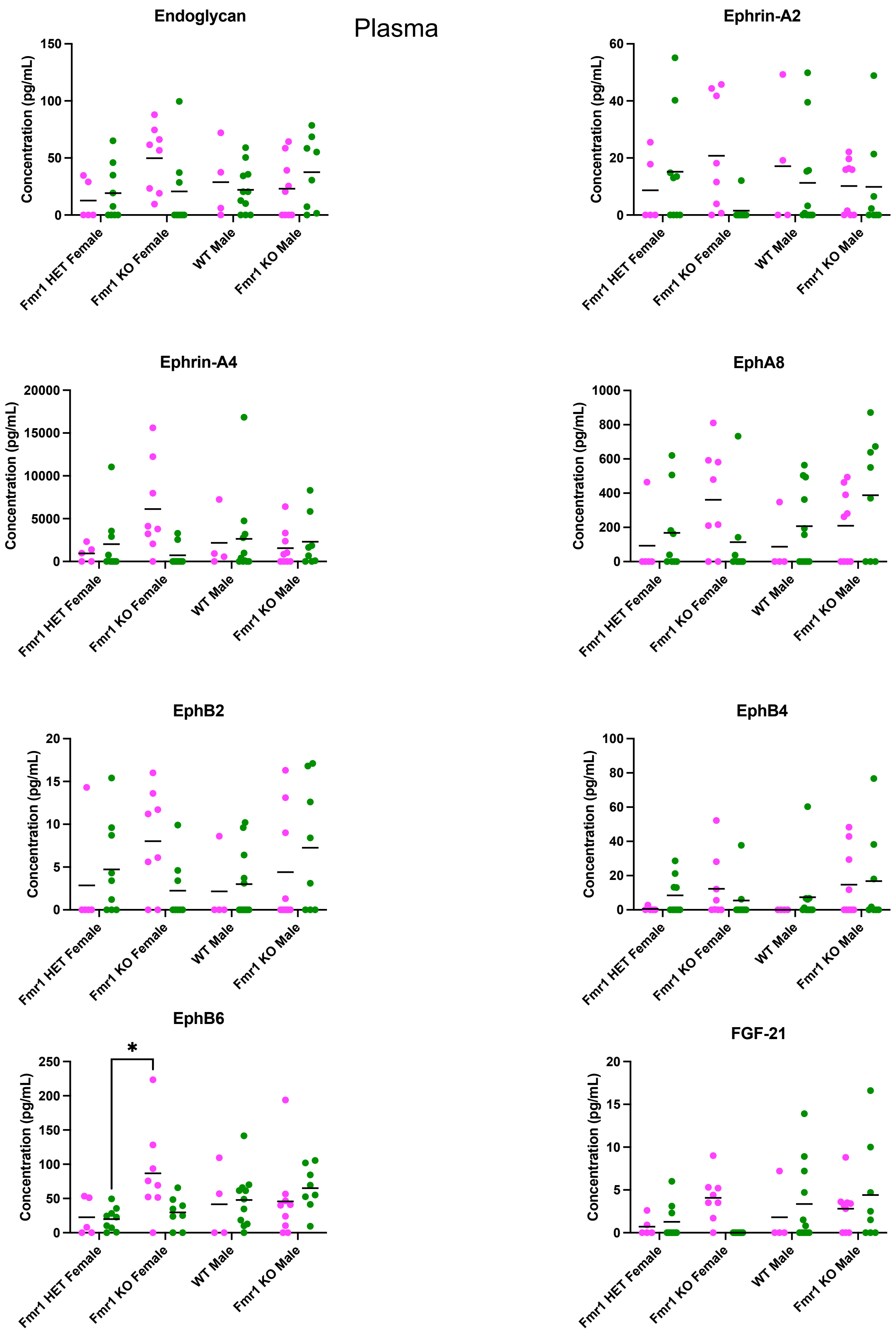
Plasma
Endoglycan
Ephrin-A2
Ephrin-A4
EphA8
EphB2
EphB4
EphB6
FGF-21
Concentration (pg/mL)
*
0
50
100
150
20
40
60
5000
10000
15000
20000
200
400
600
800
1000
5
10
15
80
250
Fmr1 HET Female
Fmr1 KO Female
WT Male
Fmr1 KO Male
