# Supplementary material for: Soy Protein Isolate Affects Blood and Brain Biomarker Expression in a Mouse Model of Fragile X
Source: Int J Mol Sci. 2025 Jun 26;26(13):6137. doi: 10.3390/ijms26136137 (PMC12250412; doi:10.3390/ijms26136137)
Supplement: Supplementary file 1 [file ijms-26-06137-s001.zip › Supplementary File S8b Array 10 Graphs.pdf]

Supplementary File S8. Protein expression of Array 10 targets as function of *Fmr1* genotype and AIN-93G diets. Mice on AIN-93G/cas (colored pink) included n=5 $Fmr1^{HET}$ female, n=8 $Fmr1^{KO}$ female, n=4 WT male and n=9 $Fmr1^{KO}$ male. Mice on AIN-93G/soy (colored green) included n=9 $Fmr1^{HET}$ female, n=8 $Fmr1^{KO}$ female, n=11 WT male and n=8 $Fmr1^{KO}$ male. The average concentration in cortex, hippocampus, hypothalamus and plasma in pg/mL was plotted versus genotype. Statistics were determined by 2-way ANOVA and Tukey's multiple comparison tests denoted by $p < 0.05$ (*), $p < 0.01$ (**), $p < 0.001$ (***) and $p < 0.0001$ (****).

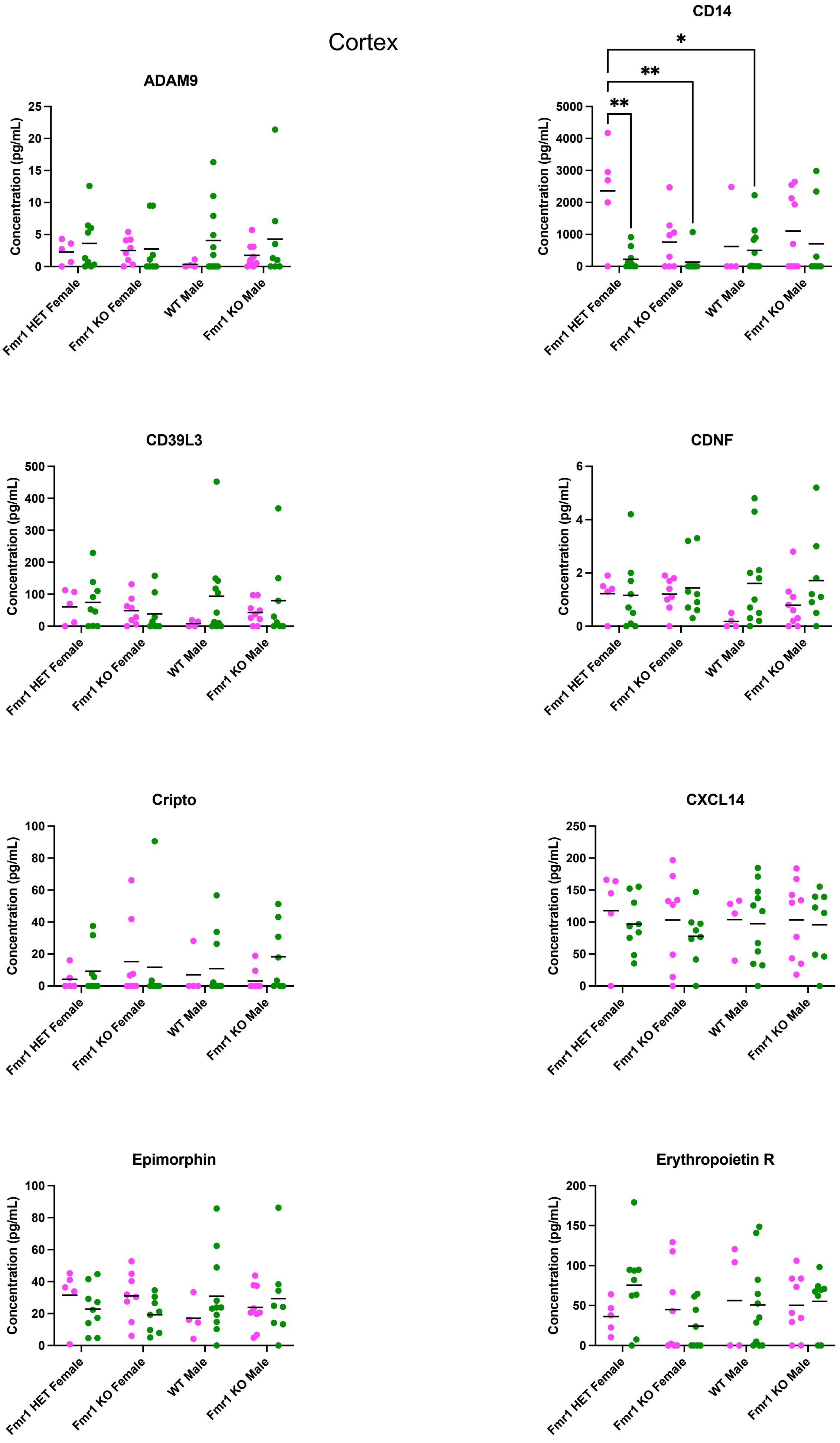
Cortex
ADAM9
CD14
*
**
**
CD39L3
CDNF
Cripto
CXCL14
Epimorphin
Erythropoietin R
Concentration (pg/mL)
Fmr1 HET Female
Fmr1 KO Female
WT Male
Fmr1 KO Male
0
5
10
15
20
25
1000
2000
3000
4000
5000
100
200
300
400
500
2
4
6
20
40
60
80
50
150
250

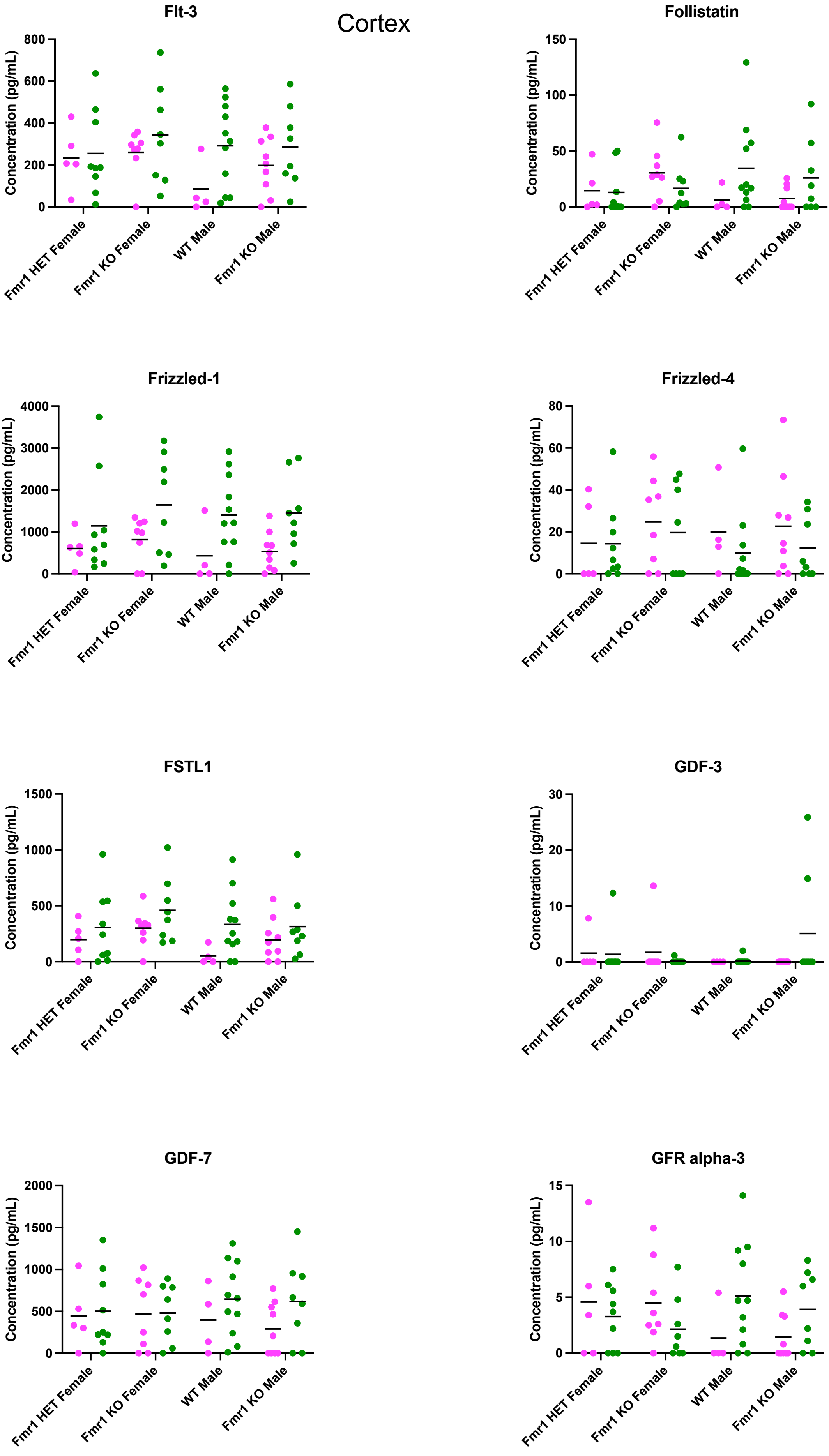
Cortex
Flt-3
Follistatin
Frizzled-1
Frizzled-4
FSTL1
GDF-3
GDF-7
GFR alpha-3
Concentration (pg/mL)
Fmr1 HET Female
Fmr1 KO Female
WT Male
Fmr1 KO Male
0
200
400
600
800
50
100
150
1000
2000
3000
4000
20
40
60
80
500
1500
10
30
5
15

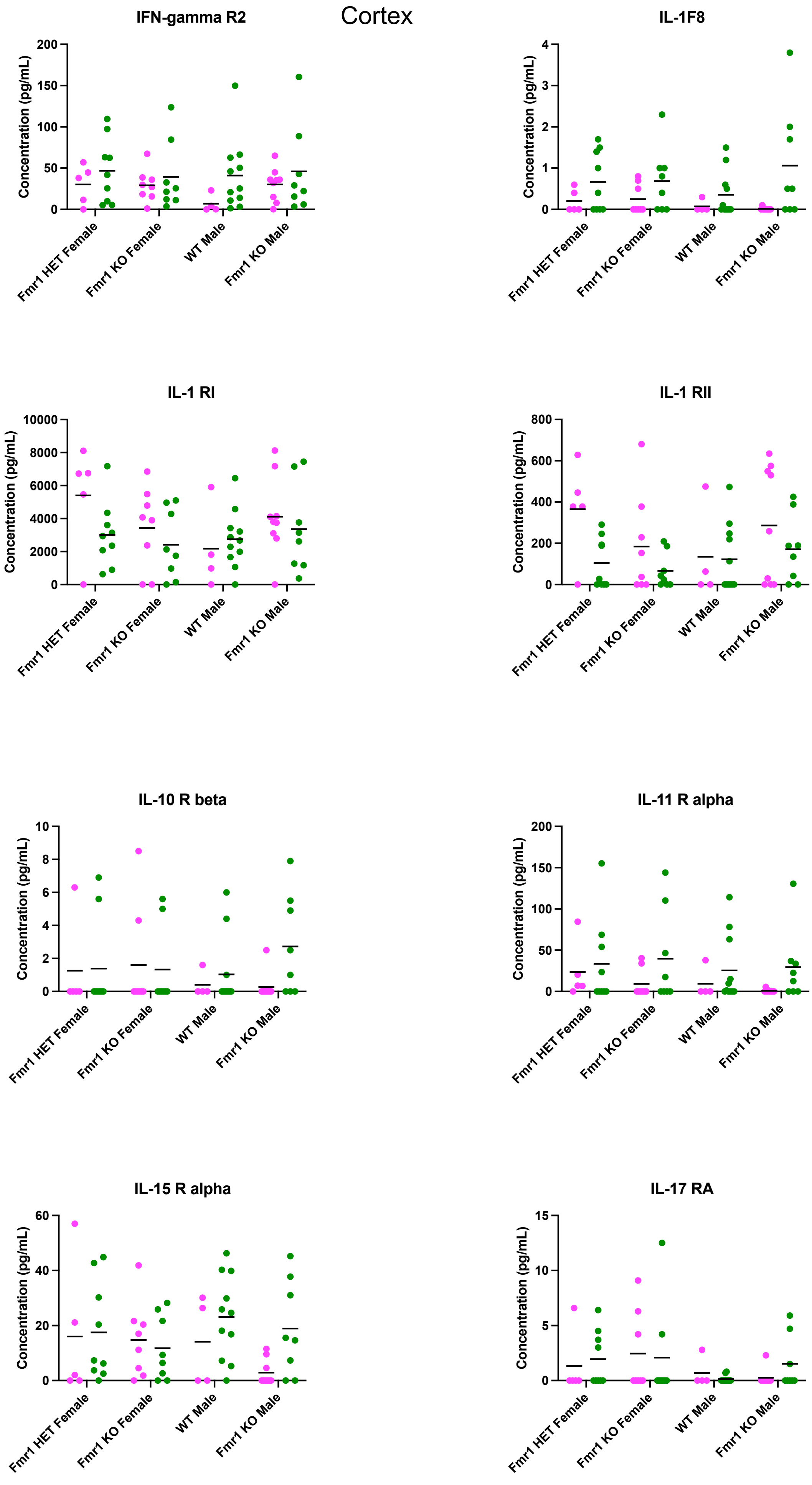
Cortex
IFN-gamma R2
IL-1F8
IL-1 RI
IL-1 RII
IL-10 R beta
IL-11 R alpha
IL-15 R alpha
IL-17 RA
Concentration (pg/mL)
Fmr1 HET Female
Fmr1 KO Female
WT Male
Fmr1 KO Male

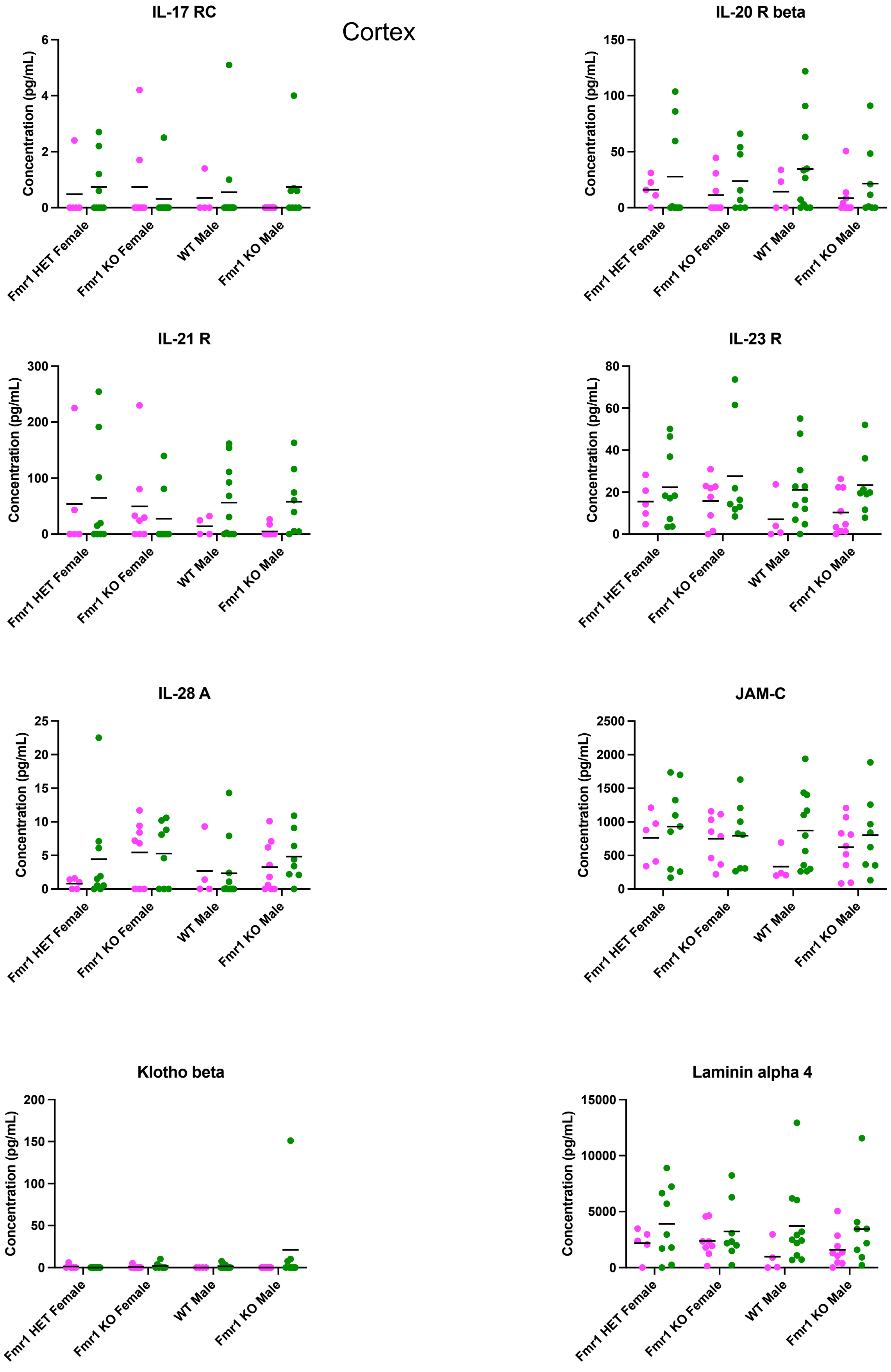
Cortex
IL-17 RC
IL-20 R beta
IL-21 R
IL-23 R
IL-28 A
JAM-C
Klotho beta
Laminin alpha 4
Concentration (pg/mL)
Fmr1 HET Female
Fmr1 KO Female
WT Male
Fmr1 KO Male
0
2
4
6
50
100
150
200
300
20
40
60
80
5
10
15
25
500
1000
1500
2000
2500
5000
10000
15000

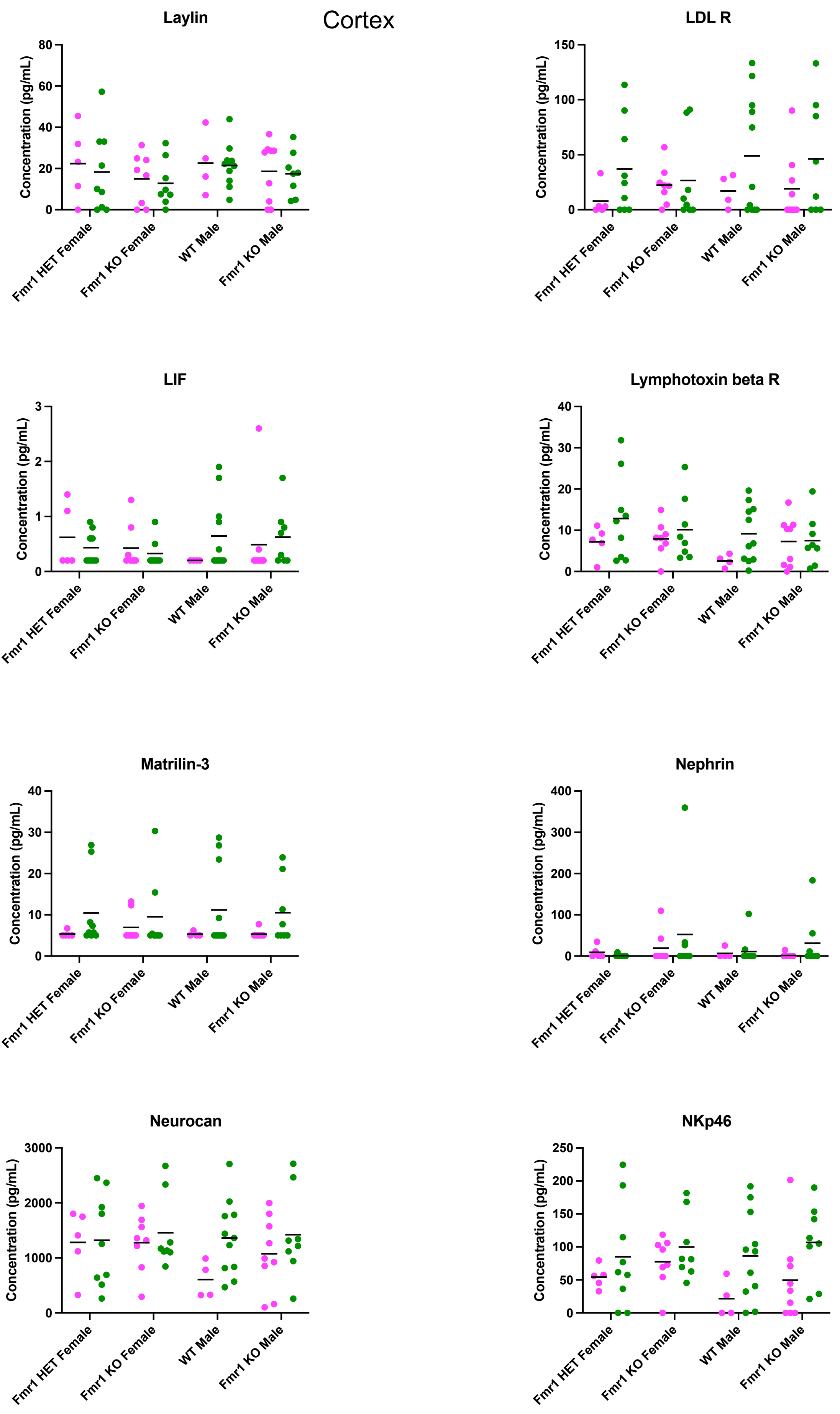

Cortex
Laylin
LDL R
LIF
Lymphotoxin beta R
Matrilin-3
Nephrin
Neurocan
NKp46
Concentration (pg/mL)
Fmr1 HET Female
Fmr1 KO Female
WT Male
Fmr1 KO Male

Hippocampus

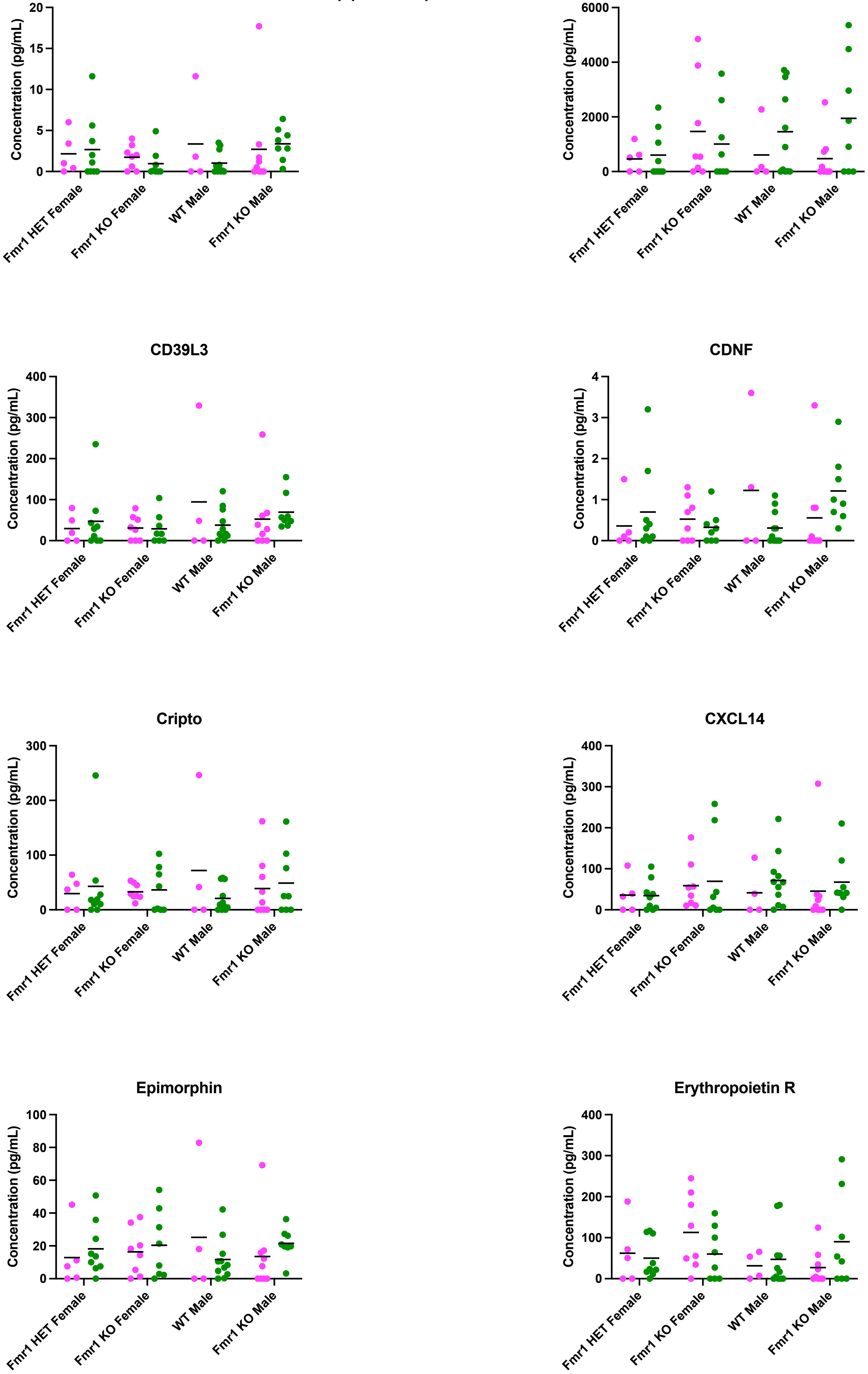

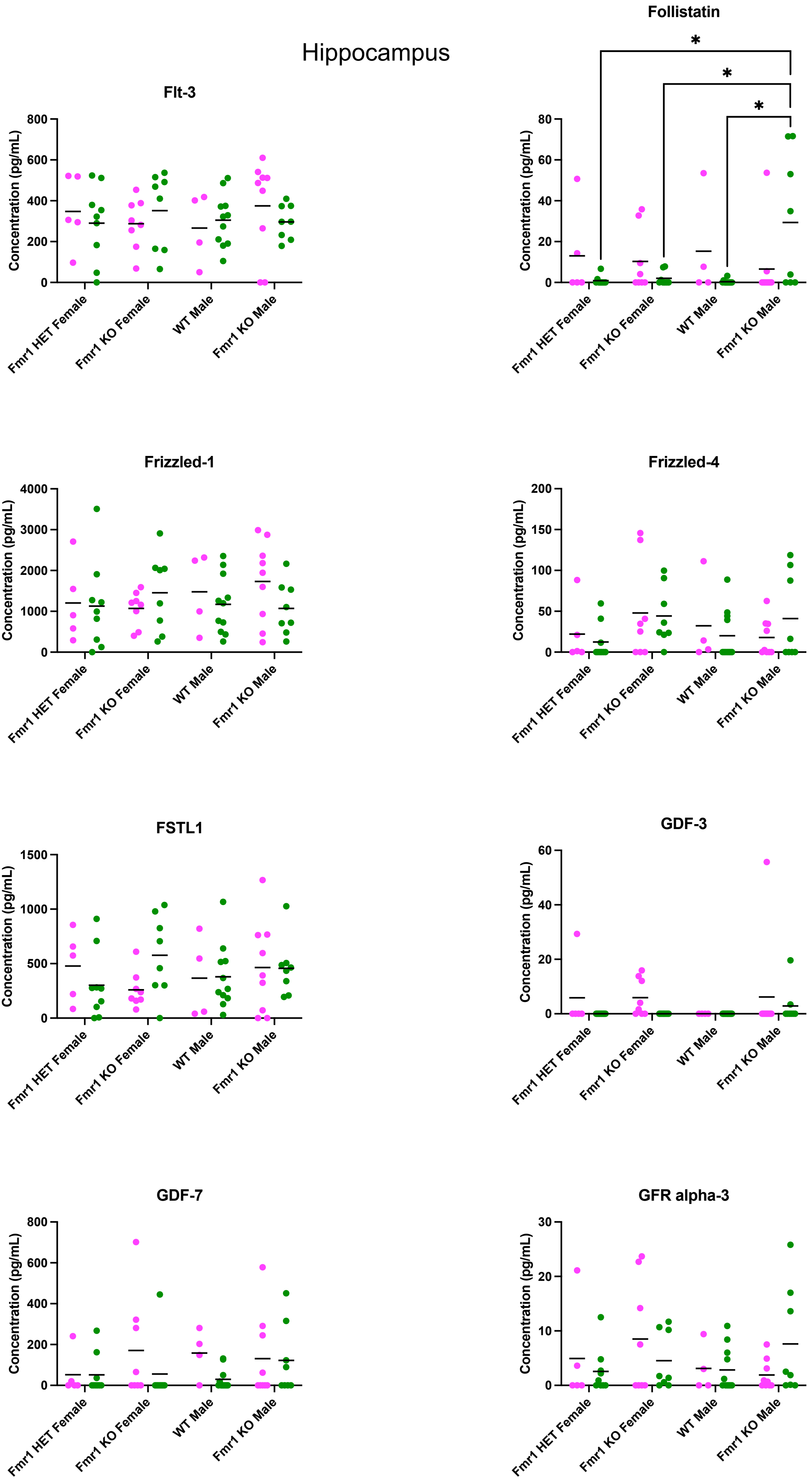
Hippocampus
Flt-3
Follistatin
Frizzled-1
Frizzled-4
FSTL1
GDF-3
GDF-7
GFR alpha-3
Concentration (pg/mL)
Fmr1 HET Female
Fmr1 KO Female
WT Male
Fmr1 KO Male
*
*
*

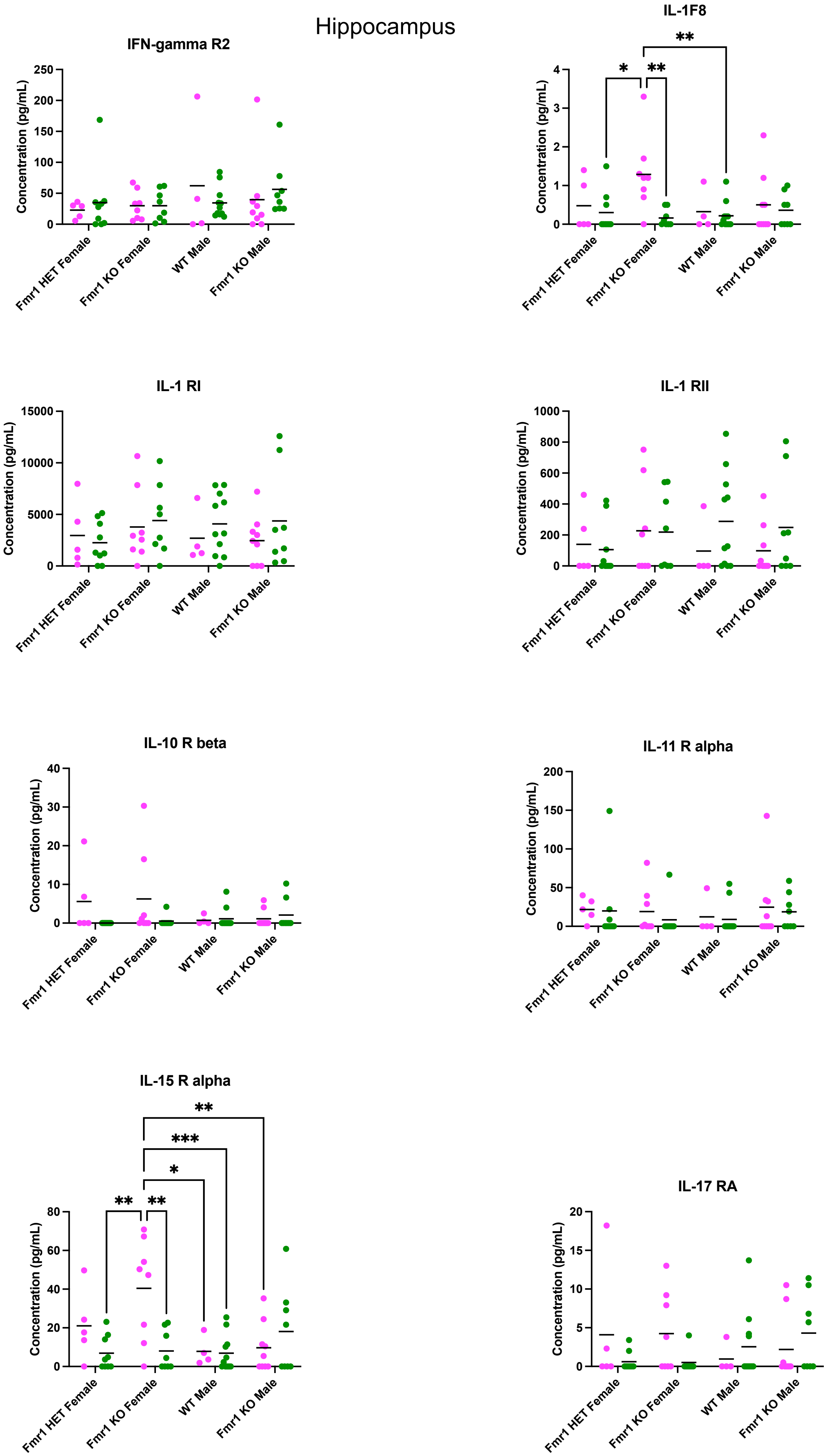
Hippocampus
IFN-gamma R2
IL-1F8
IL-1 RI
IL-1 RII
IL-10 R beta
IL-11 R alpha
IL-15 R alpha
IL-17 RA
Concentration (pg/mL)
Fmr1 HET Female
Fmr1 KO Female
WT Male
Fmr1 KO Male
*
**
***

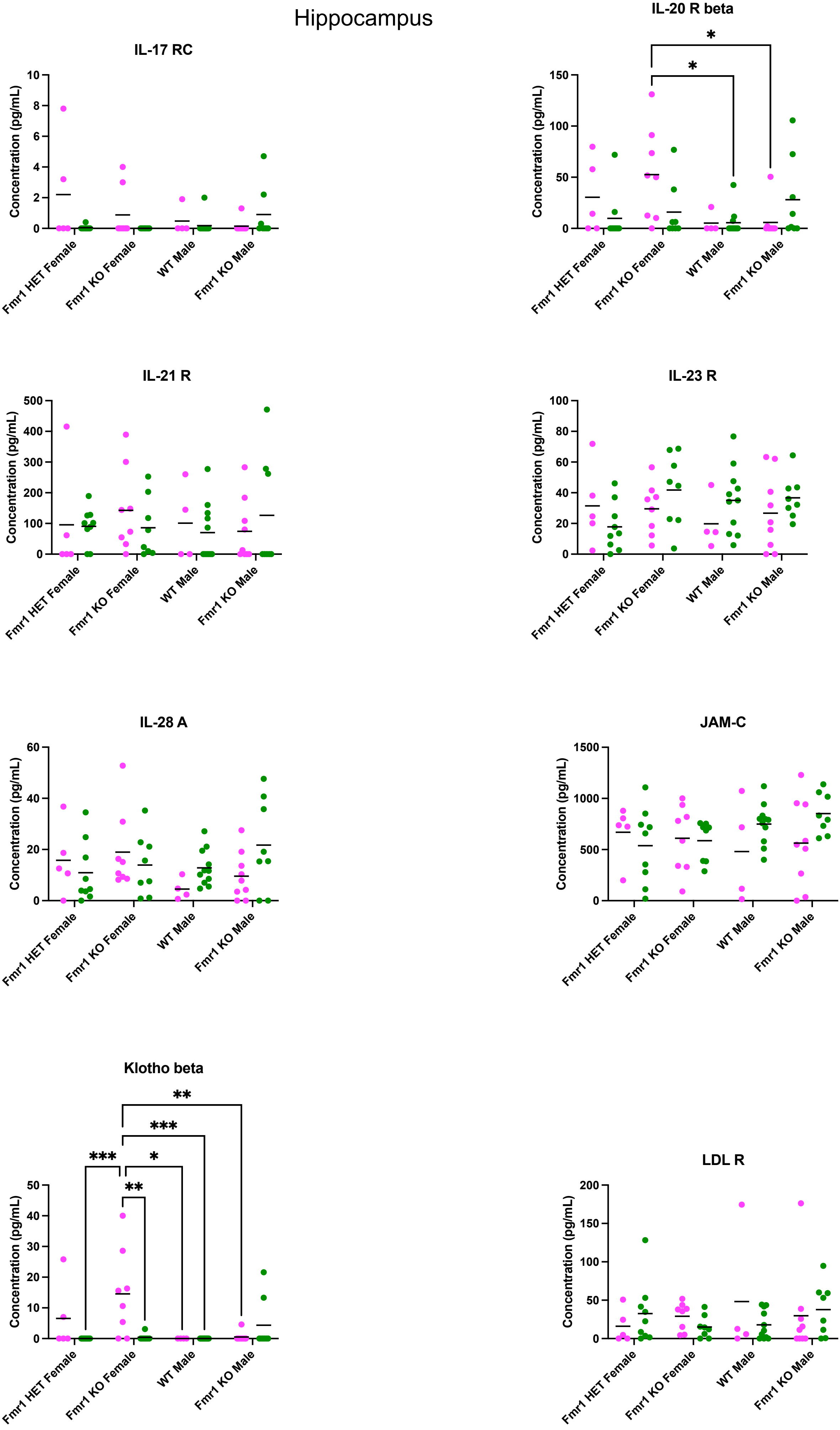
Hippocampus
IL-17 RC
IL-20 R beta
IL-21 R
IL-23 R
IL-28 A
JAM-C
Klotho beta
LDL R
Concentration (pg/mL)
Fmr1 HET Female
Fmr1 KO Female
WT Male
Fmr1 KO Male
*
**
***

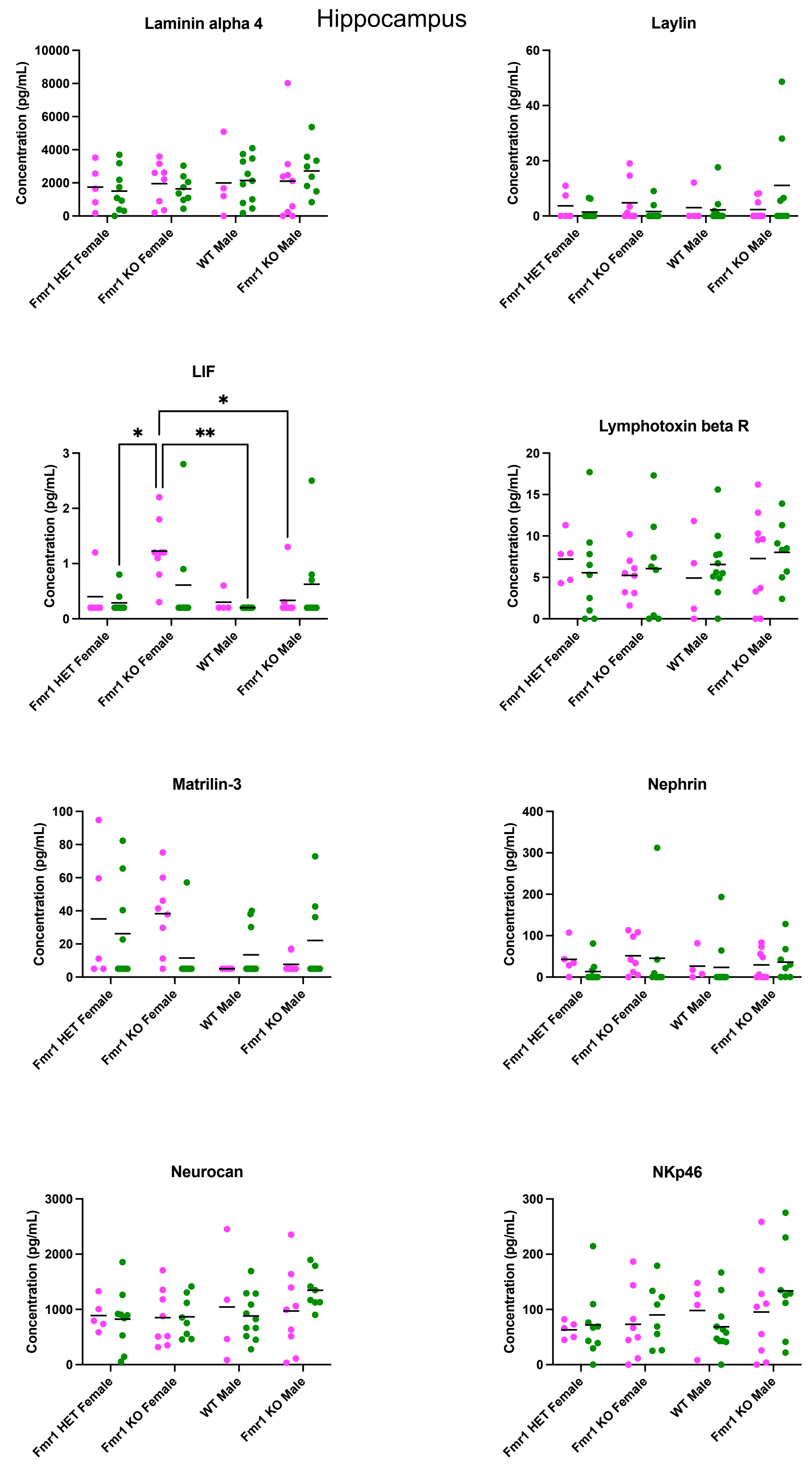
Hippocampus
Laminin alpha 4
Laylin
LIF
Lymphotoxin beta R
Matrilin-3
Nephrin
Neurocan
NKp46
Concentration (pg/mL)
0
2000
4000
6000
8000
10000
20
40
60
1
2
3
5
10
15
80
100
200
300
400
1000
3000
*
**
Fmr1 HET Female
Fmr1 KO Female
WT Male
Fmr1 KO Male

Plasma

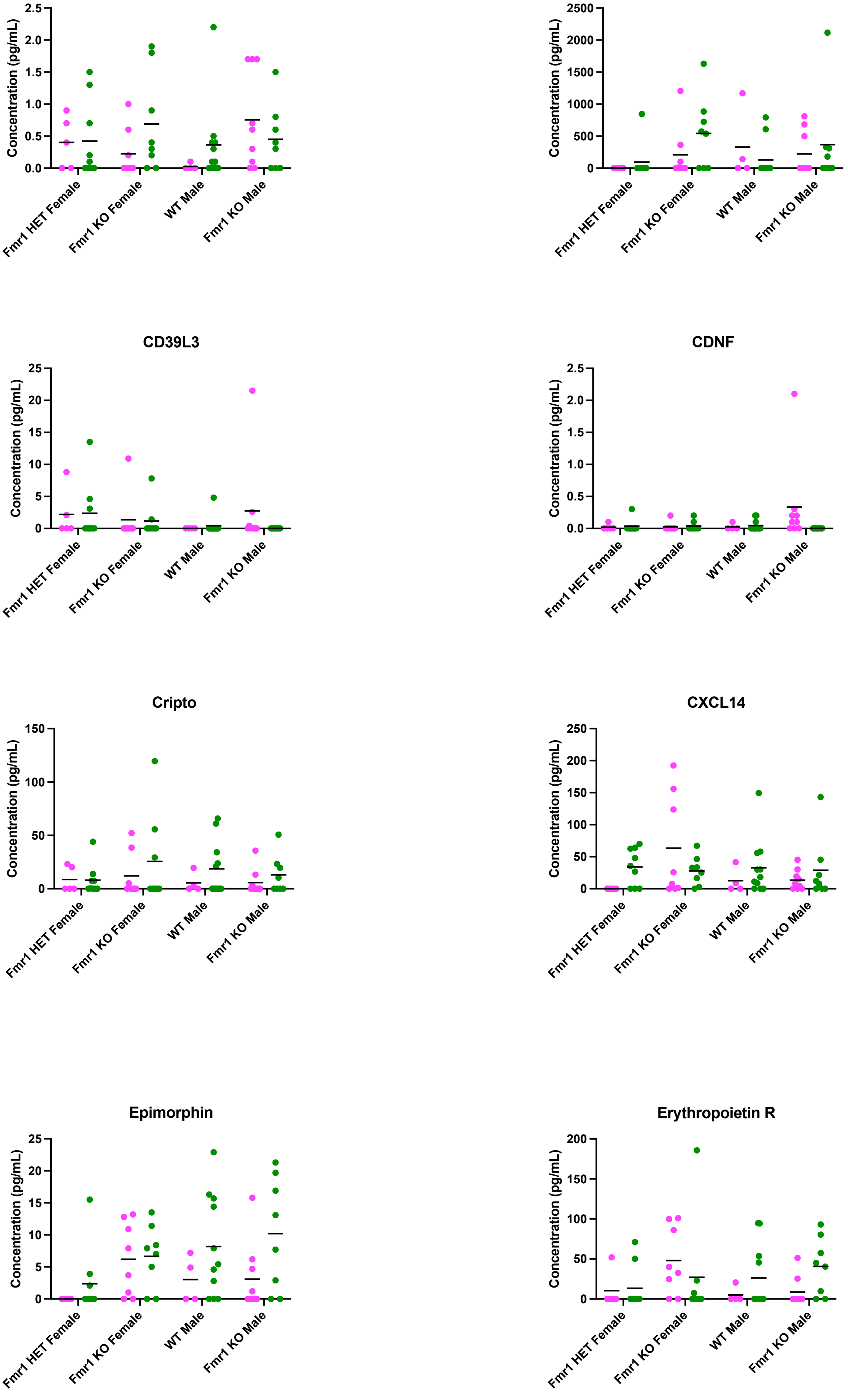
ADAM9
Concentration (pg/mL)
0.0
0.5
1.0
1.5
2.0
2.5
Fmr1 HET Female
Fmr1 KO Female
WT Male
Fmr1 KO Male
CD14
Concentration (pg/mL)
0
500
1000
1500
2000
2500
Fmr1 HET Female
Fmr1 KO Female
WT Male
Fmr1 KO Male
CD39L3
Concentration (pg/mL)
0
5
10
15
20
25
Fmr1 HET Female
Fmr1 KO Female
WT Male
Fmr1 KO Male
CDNF
Concentration (pg/mL)
0.0
0.5
1.0
1.5
2.0
2.5
Fmr1 HET Female
Fmr1 KO Female
WT Male
Fmr1 KO Male
Cripto
Concentration (pg/mL)
0
50
100
150
Fmr1 HET Female
Fmr1 KO Female
WT Male
Fmr1 KO Male
CXCL14
Concentration (pg/mL)
0
50
100
150
200
250
Fmr1 HET Female
Fmr1 KO Female
WT Male
Fmr1 KO Male
Epimorphin
Concentration (pg/mL)
0
5
10
15
20
25
Fmr1 HET Female
Fmr1 KO Female
WT Male
Fmr1 KO Male
Erythropoietin R
Concentration (pg/mL)
0
50
100
150
200
Fmr1 HET Female
Fmr1 KO Female
WT Male
Fmr1 KO Male

Plasma

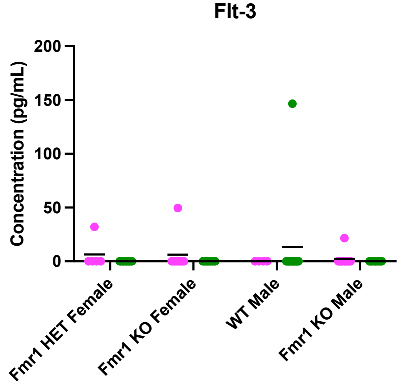
Flt-3
Concentration (pg/mL)
200
150
100
50
0
Fmr1 HET Female
Fmr1 KO Female
WT Male
Fmr1 KO Male

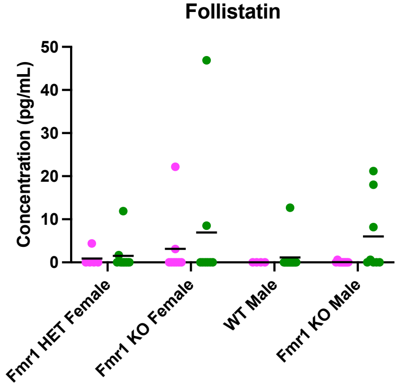
Follistatin
Concentration (pg/mL)
50
40
30
20
10
0
Fmr1 HET Female
Fmr1 KO Female
WT Male
Fmr1 KO Male

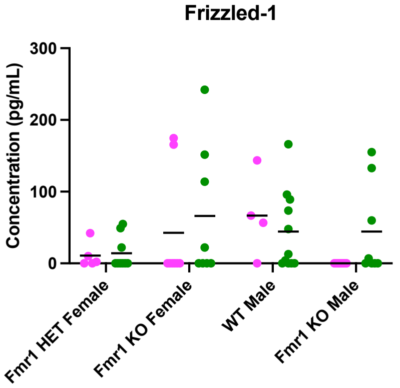
Frizzled-1
Concentration (pg/mL)
300
200
100
0
Fmr1 HET Female
Fmr1 KO Female
WT Male
Fmr1 KO Male

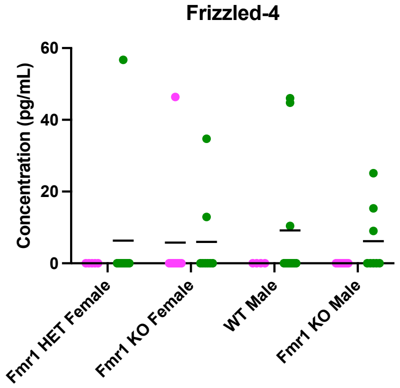
Frizzled-4
Concentration (pg/mL)
60
40
20
0
Fmr1 HET Female
Fmr1 KO Female
WT Male
Fmr1 KO Male

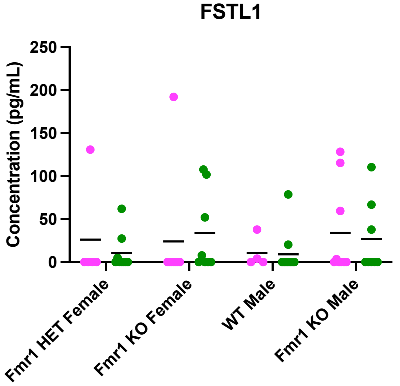
FSTL1
Concentration (pg/mL)
250
200
150
100
50
0
Fmr1 HET Female
Fmr1 KO Female
WT Male
Fmr1 KO Male

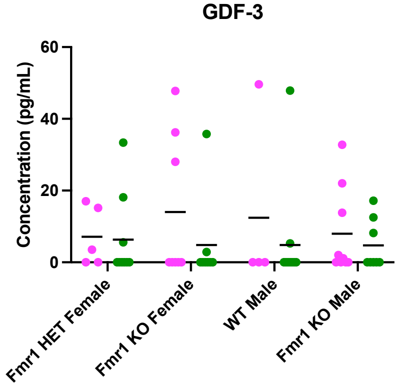
GDF-3
Concentration (pg/mL)
60
40
20
0
Fmr1 HET Female
Fmr1 KO Female
WT Male
Fmr1 KO Male

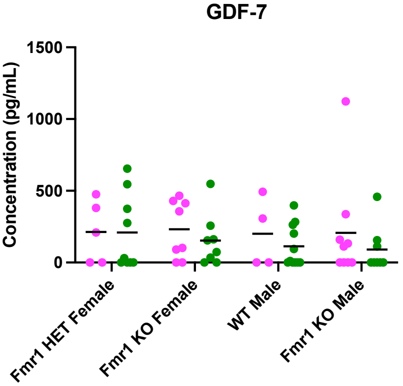
GDF-7
Concentration (pg/mL)
1500
1000
500
0
Fmr1 HET Female
Fmr1 KO Female
WT Male
Fmr1 KO Male

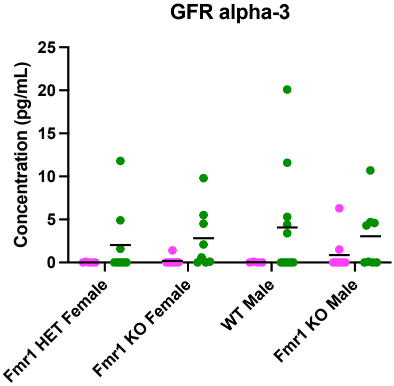
GFR alpha-3
Concentration (pg/mL)
25
20
15
10
5
0
Fmr1 HET Female
Fmr1 KO Female
WT Male
Fmr1 KO Male

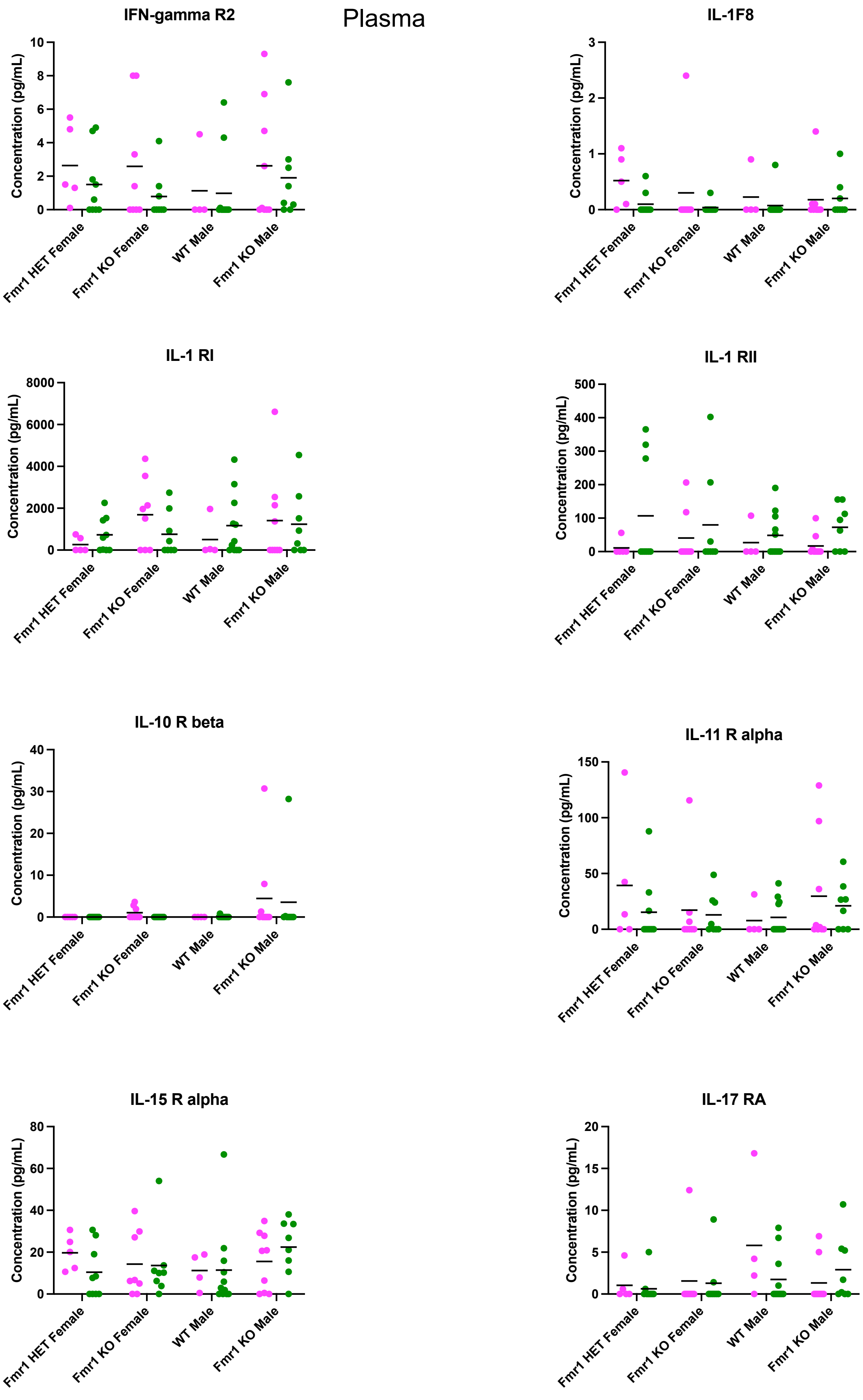
Plasma
IFN-gamma R2
IL-1F8
IL-1 RI
IL-1 RII
IL-10 R beta
IL-11 R alpha
IL-15 R alpha
IL-17 RA
Concentration (pg/mL)
Fmr1 HET Female
Fmr1 KO Female
WT Male
Fmr1 KO Male

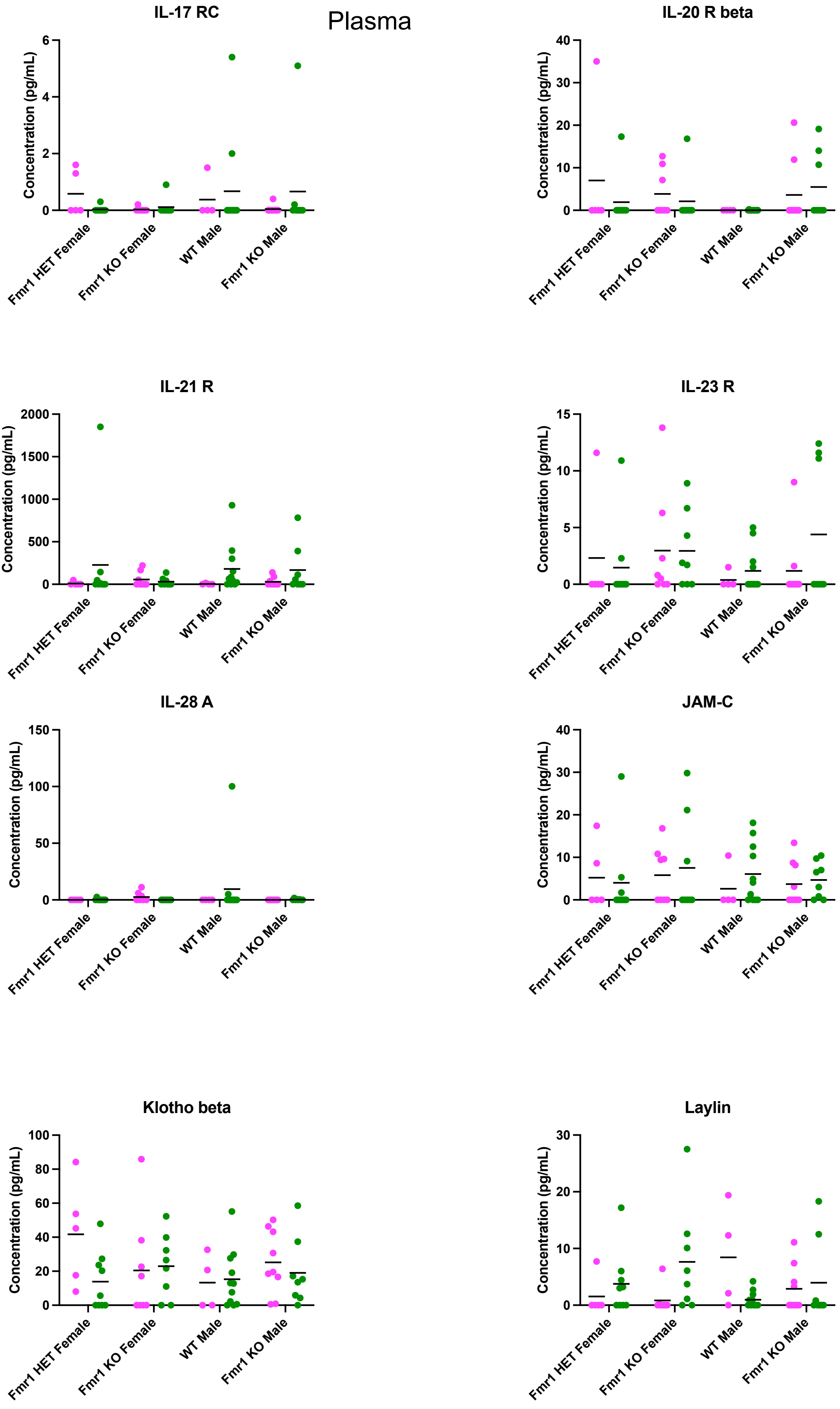
Plasma
IL-17 RC
IL-20 R beta
IL-21 R
IL-23 R
IL-28 A
JAM-C
Klotho beta
Laylin
Concentration (pg/mL)
Fmr1 HET Female
Fmr1 KO Female
WT Male
Fmr1 KO Male

Plasma

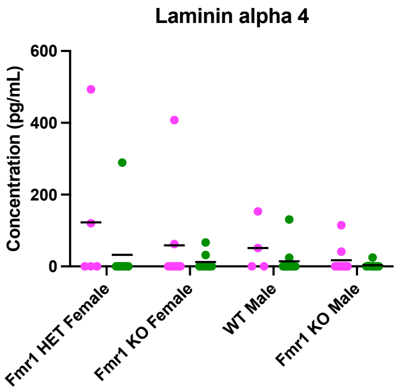
Laminin alpha 4
Concentration (pg/mL)
600
400
200
0
Fmr1 HET Female
Fmr1 KO Female
WT Male
Fmr1 KO Male

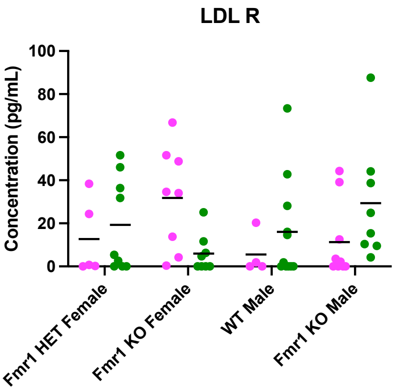
LDL R
Concentration (pg/mL)
100
80
60
40
20
0
Fmr1 HET Female
Fmr1 KO Female
WT Male
Fmr1 KO Male

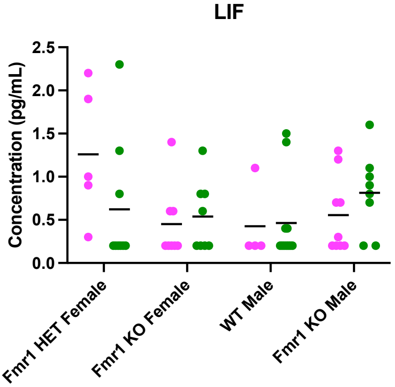
LIF
Concentration (pg/mL)
2.5
2.0
1.5
1.0
0.5
0.0
Fmr1 HET Female
Fmr1 KO Female
WT Male
Fmr1 KO Male

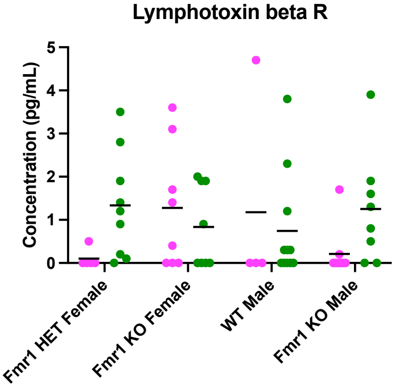
Lymphotoxin beta R
Concentration (pg/mL)
5
4
3
2
1
0
Fmr1 HET Female
Fmr1 KO Female
WT Male
Fmr1 KO Male

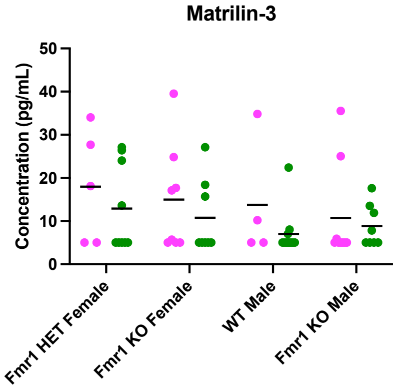
Matrilin-3
Concentration (pg/mL)
50
40
30
20
10
0
Fmr1 HET Female
Fmr1 KO Female
WT Male
Fmr1 KO Male

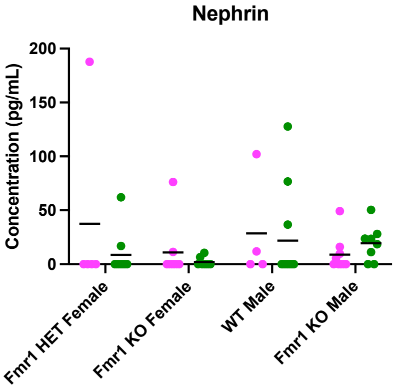
Nephrin
Concentration (pg/mL)
200
150
100
50
0
Fmr1 HET Female
Fmr1 KO Female
WT Male
Fmr1 KO Male

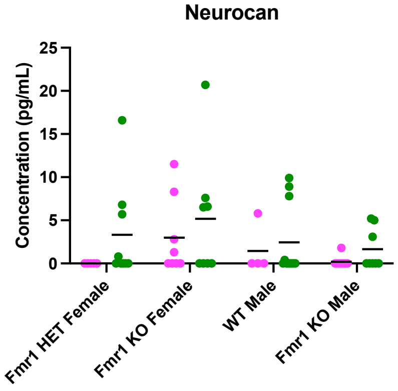
Neurocan
Concentration (pg/mL)
25
20
15
10
5
0
Fmr1 HET Female
Fmr1 KO Female
WT Male
Fmr1 KO Male

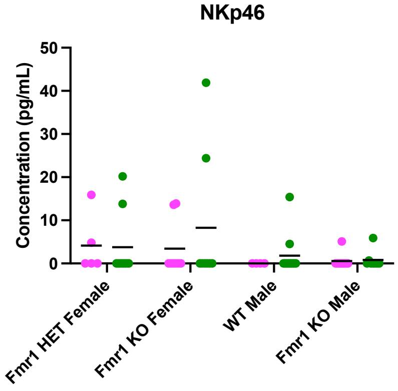
NKp46
Concentration (pg/mL)
50
40
30
20
10
0
Fmr1 HET Female
Fmr1 KO Female
WT Male
Fmr1 KO Male
