# Supplementary material for: Soy Protein Isolate Affects Blood and Brain Biomarker Expression in a Mouse Model of Fragile X
Source: Int J Mol Sci. 2025 Jun 26;26(13):6137. doi: 10.3390/ijms26136137 (PMC12250412; doi:10.3390/ijms26136137)
Supplement: Supplementary file 1 [file ijms-26-06137-s001.zip › Supplementary File S9b Array 11 Graphs.pdf]

Supplementary File S9. Protein expression of Array 11 targets as function of *Fmr1* genotype and AIN-93G diets. Mice on AIN-93G/cas (colored pink) included n=5 *Fmr1*^{HET} female, n=8 *Fmr1*^{KO} female, n=4 WT male and n=9 *Fmr1*^{KO} male. Mice on AIN-93G/soy (colored green) included n=9 *Fmr1*^{HET} female, n=8 *Fmr1*^{KO} female, n=11 WT male and n=8 *Fmr1*^{KO} male. The average concentration in cortex, hippocampus, hypothalamus and plasma in pg/mL was plotted versus genotype. Statistics were determined by 2-way ANOVA and Tukey's multiple comparison tests denoted by $p < 0.05$ (*), $p < 0.01$ (**), $p < 0.001$ (***) and $p < 0.0001$ (****).

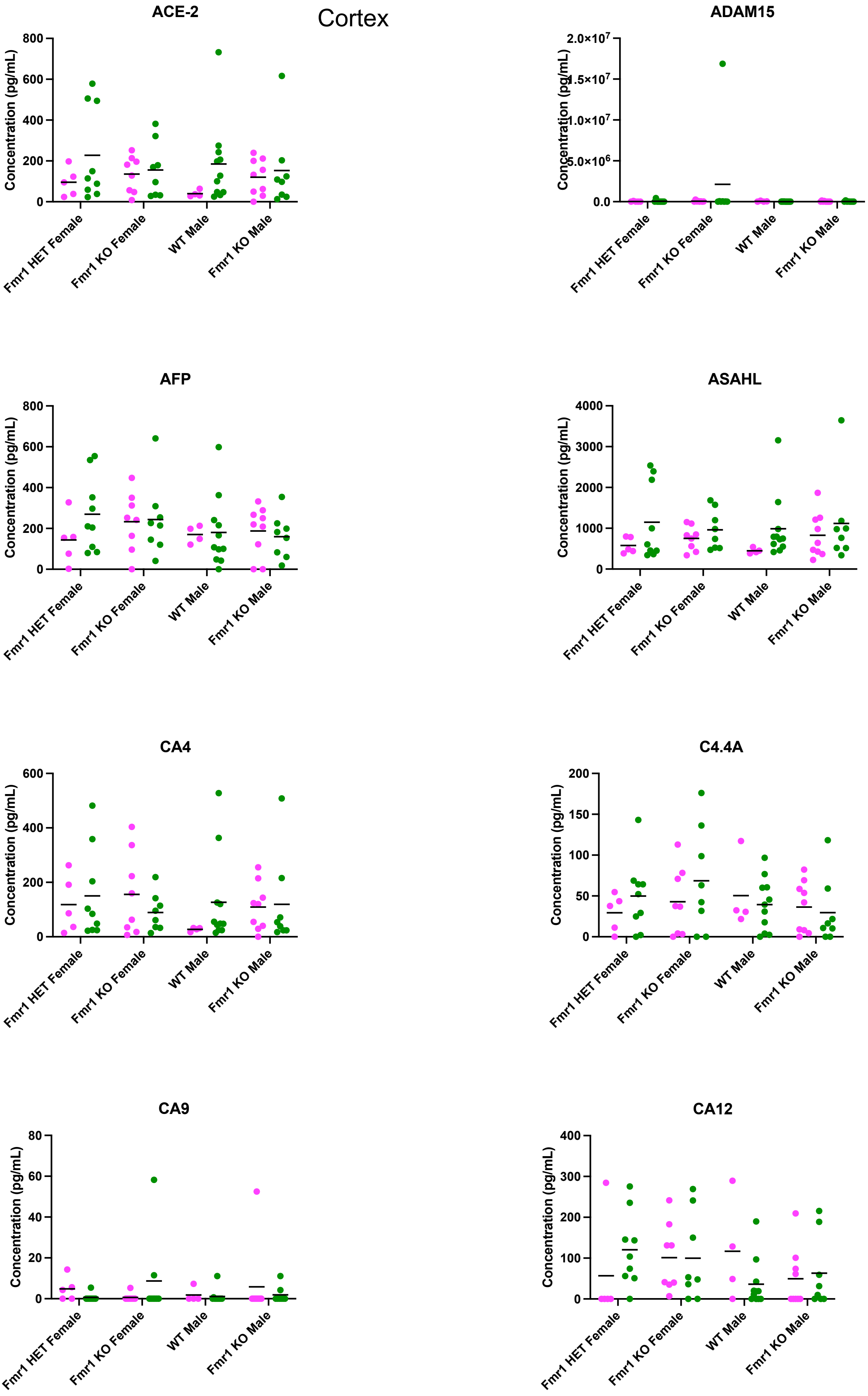
Cortex
ACE-2
ADAM15
AFP
ASAHL
CA4
C4.4A
CA9
CA12
Concentration (pg/mL)
Fmr1 HET Female
Fmr1 KO Female
WT Male
Fmr1 KO Male
0
200
400
600
800
0.0
5.0×10^6
1.0×10^7
1.5×10^7
2.0×10^7
1000
2000
3000
4000
50
100
150
20
40
60
80
300

Cortex

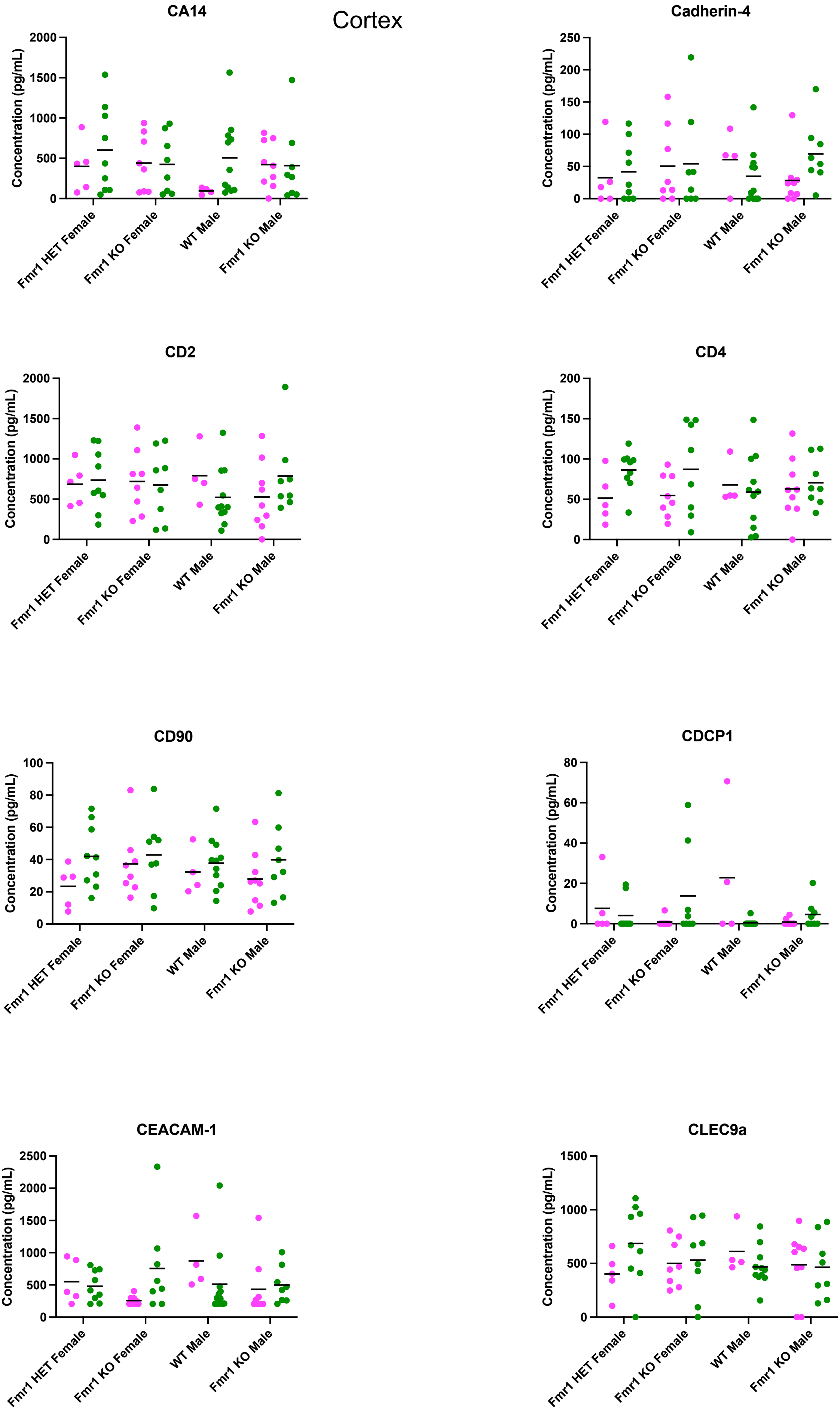
CA14
Cadherin-4
CD2
CD4
CD90
CDCP1
CEACAM-1
CLEC9a
Concentration (pg/mL)
Fmr1 HET Female
Fmr1 KO Female
WT Male
Fmr1 KO Male

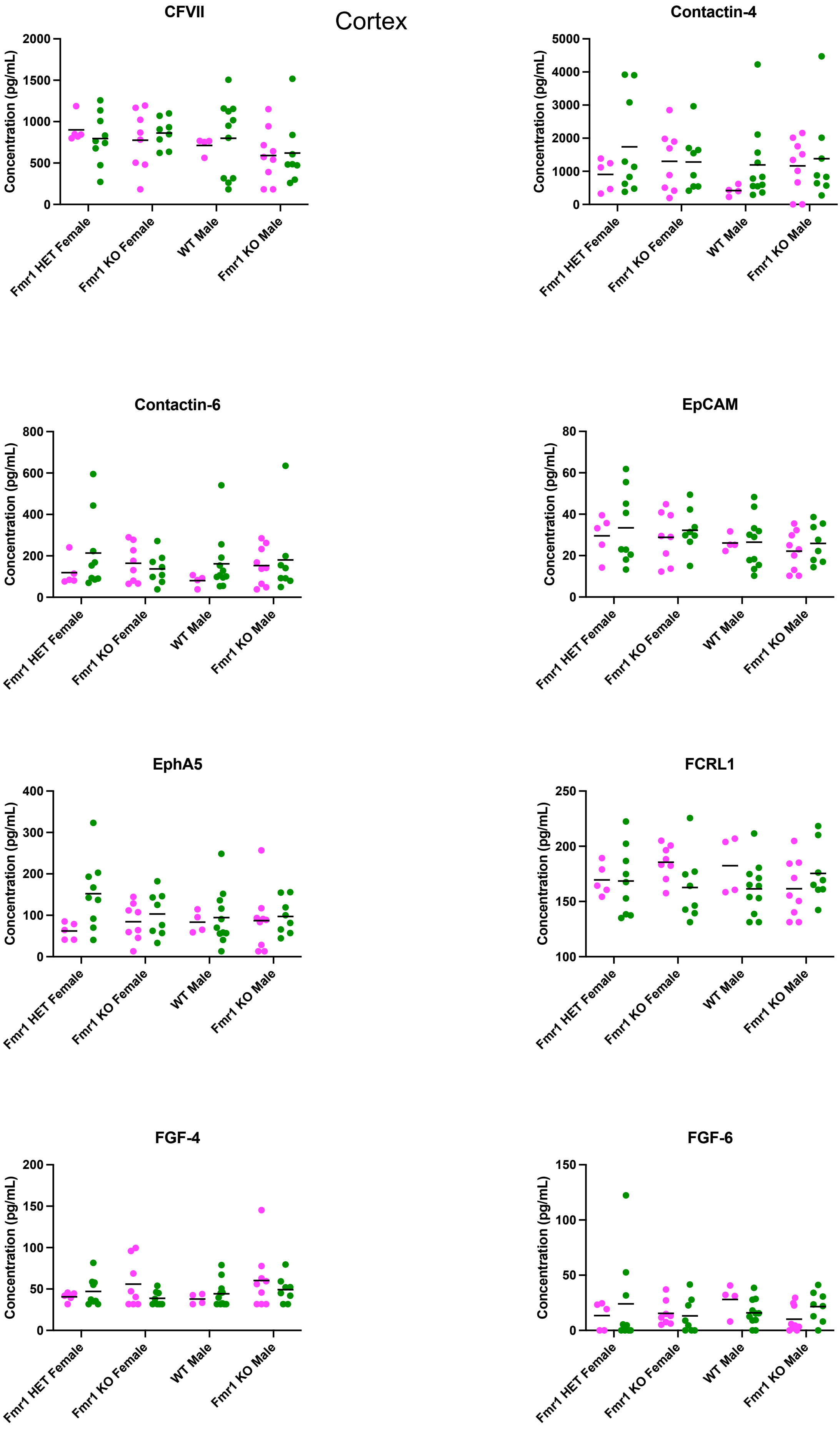
Cortex
CFVII
Contactin-4
Contactin-6
EpCAM
EphA5
FCRL1
FGF-4
FGF-6
Concentration (pg/mL)
Fmr1 HET Female
Fmr1 KO Female
WT Male
Fmr1 KO Male
0
500
1000
1500
2000
3000
4000
5000
200
400
600
800
20
40
60
80
100
300
150
250
50

Cortex

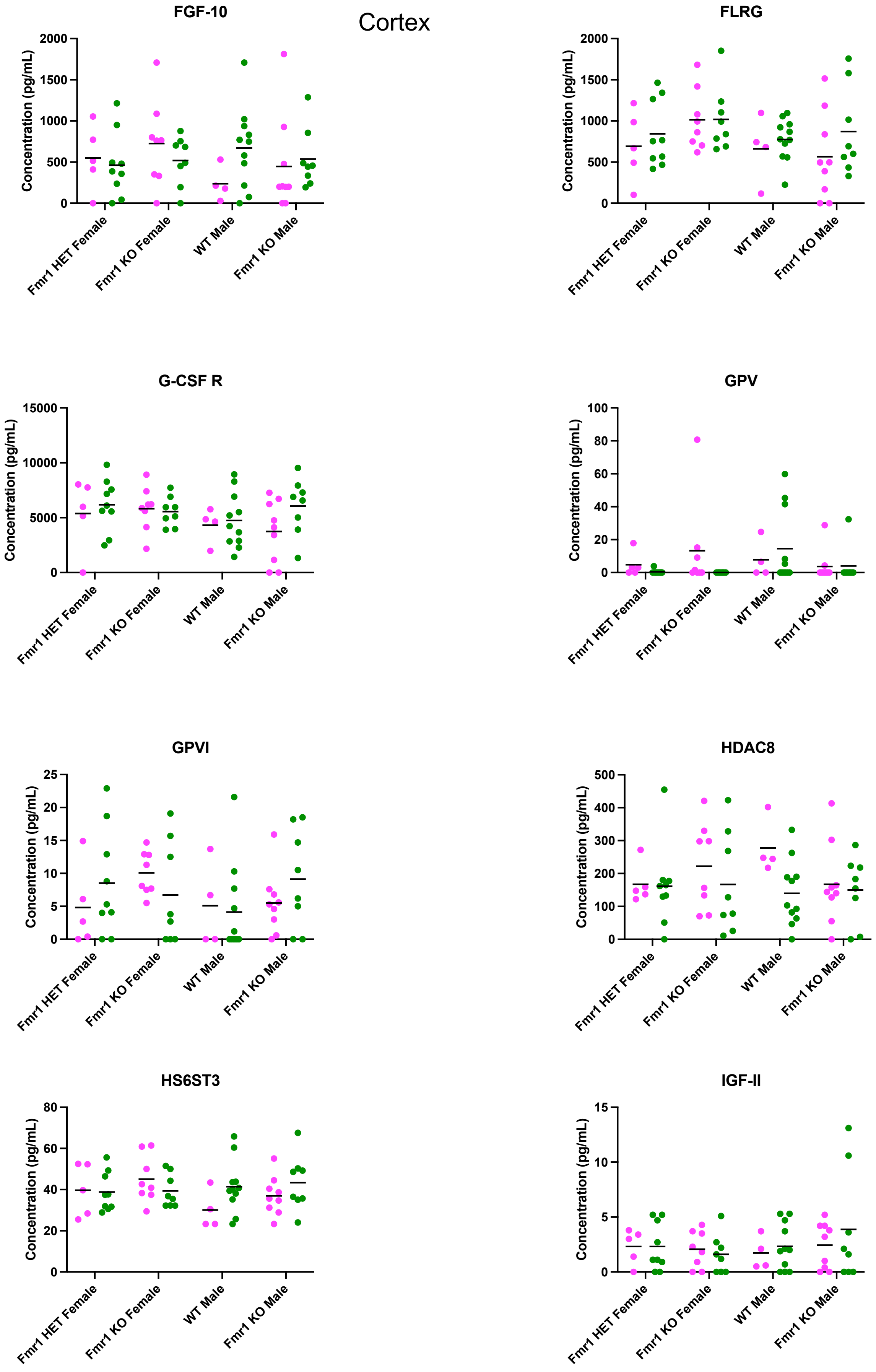
FGF-10
FLRG
G-CSF R
GPV
GPVI
HDAC8
HS6ST3
IGF-II
Concentration (pg/mL)
Fmr1 HET Female
Fmr1 KO Female
WT Male
Fmr1 KO Male

Cortex

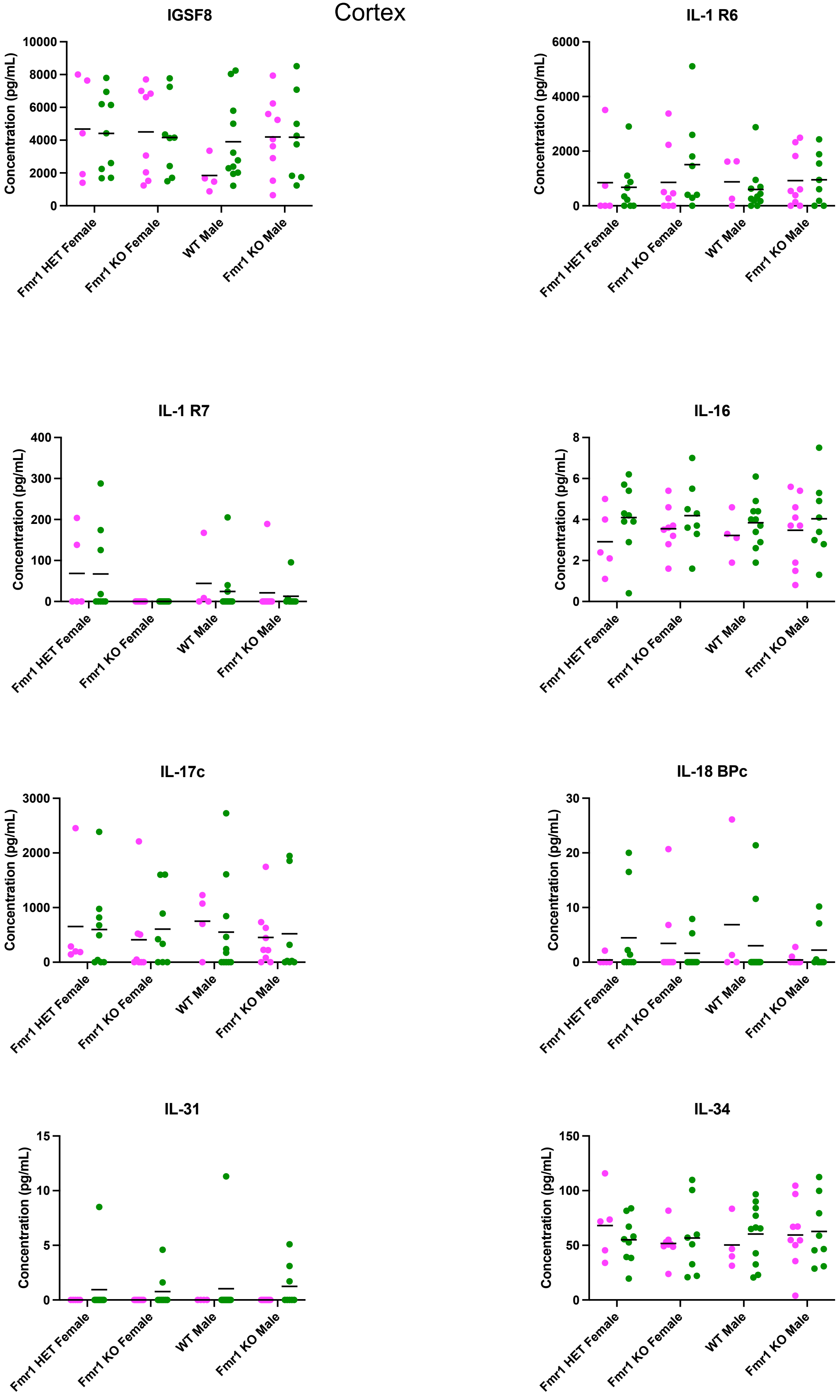
IGSF8
IL-1 R6
IL-1 R7
IL-16
IL-17c
IL-18 BPc
IL-31
IL-34
Concentration (pg/mL)
Fmr1 HET Female
Fmr1 KO Female
WT Male
Fmr1 KO Male

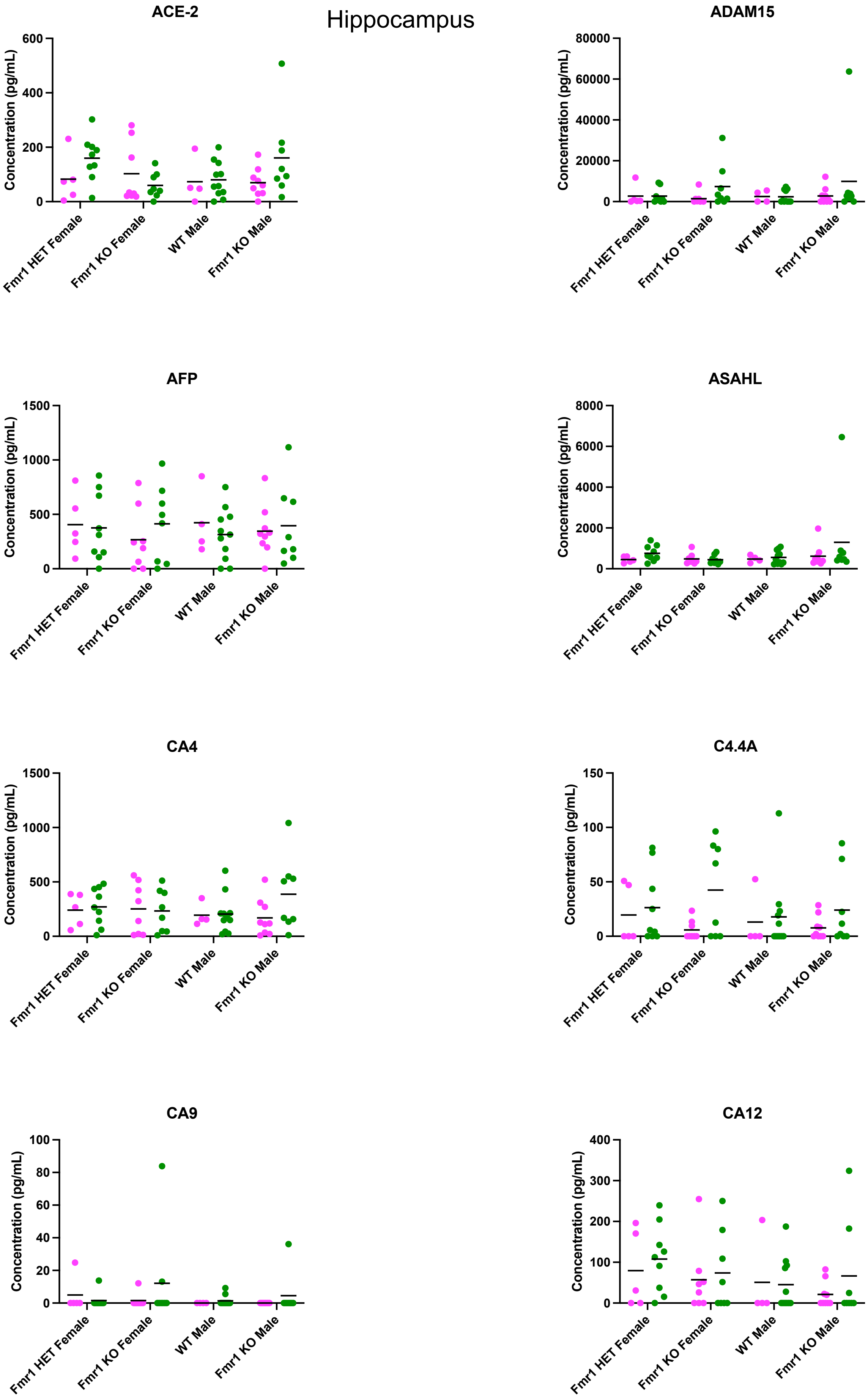
Hippocampus
ACE-2
ADAM15
AFP
ASAHL
CA4
C4.4A
CA9
CA12
Concentration (pg/mL)
Fmr1 HET Female
Fmr1 KO Female
WT Male
Fmr1 KO Male
0
200
400
600
20000
40000
60000
80000
500
1000
1500
2000
4000
6000
8000
50
100
150
20
40
60
80
300

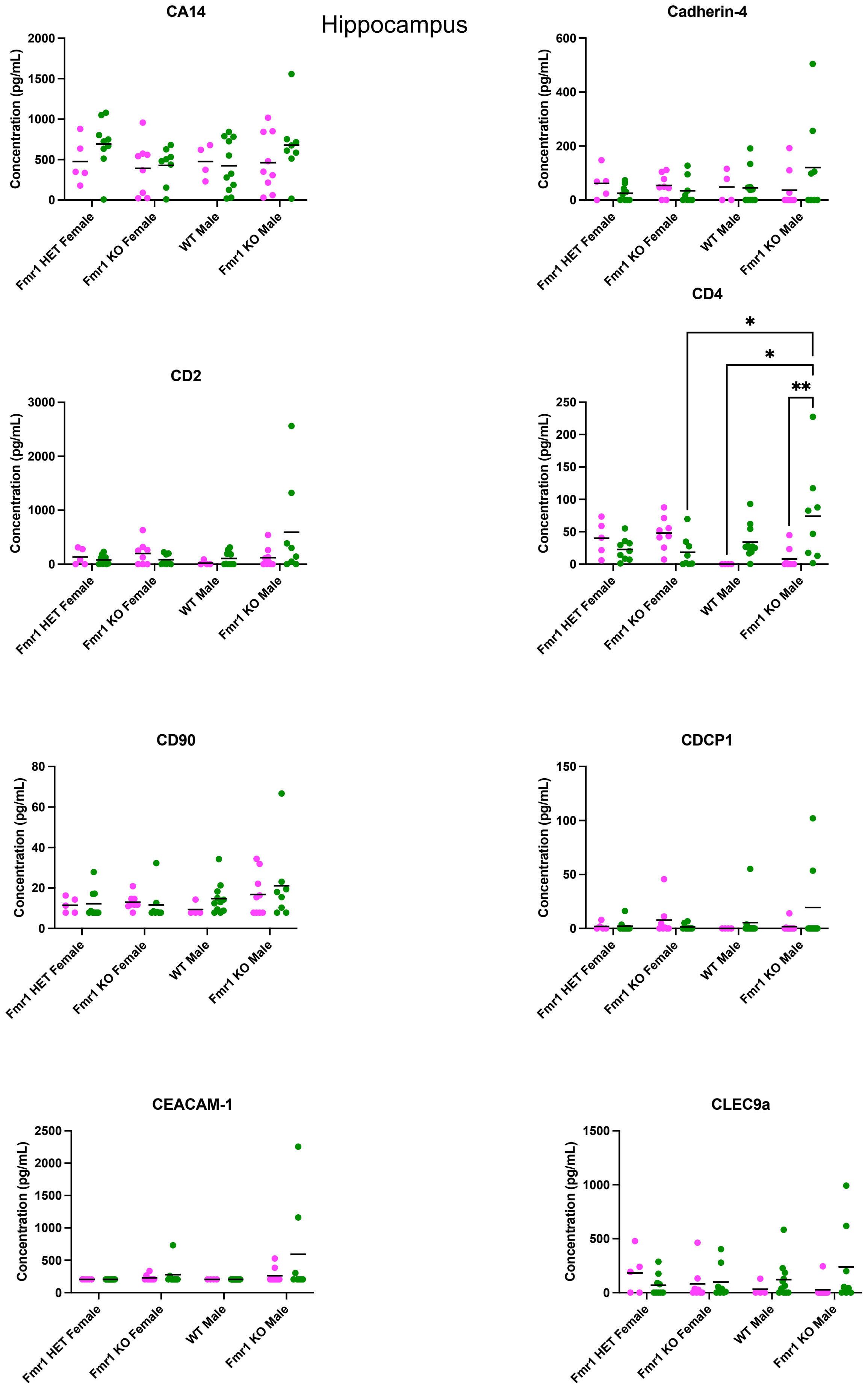
Hippocampus
CA14
Cadherin-4
CD2
CD4
CD90
CDCP1
CEACAM-1
CLEC9a
Concentration (pg/mL)
2000
1500
1000
500
0
600
400
200
3000
250
150
100
50
80
60
40
20
2500
*
*
**
Fmr1 HET Female
Fmr1 KO Female
WT Male
Fmr1 KO Male

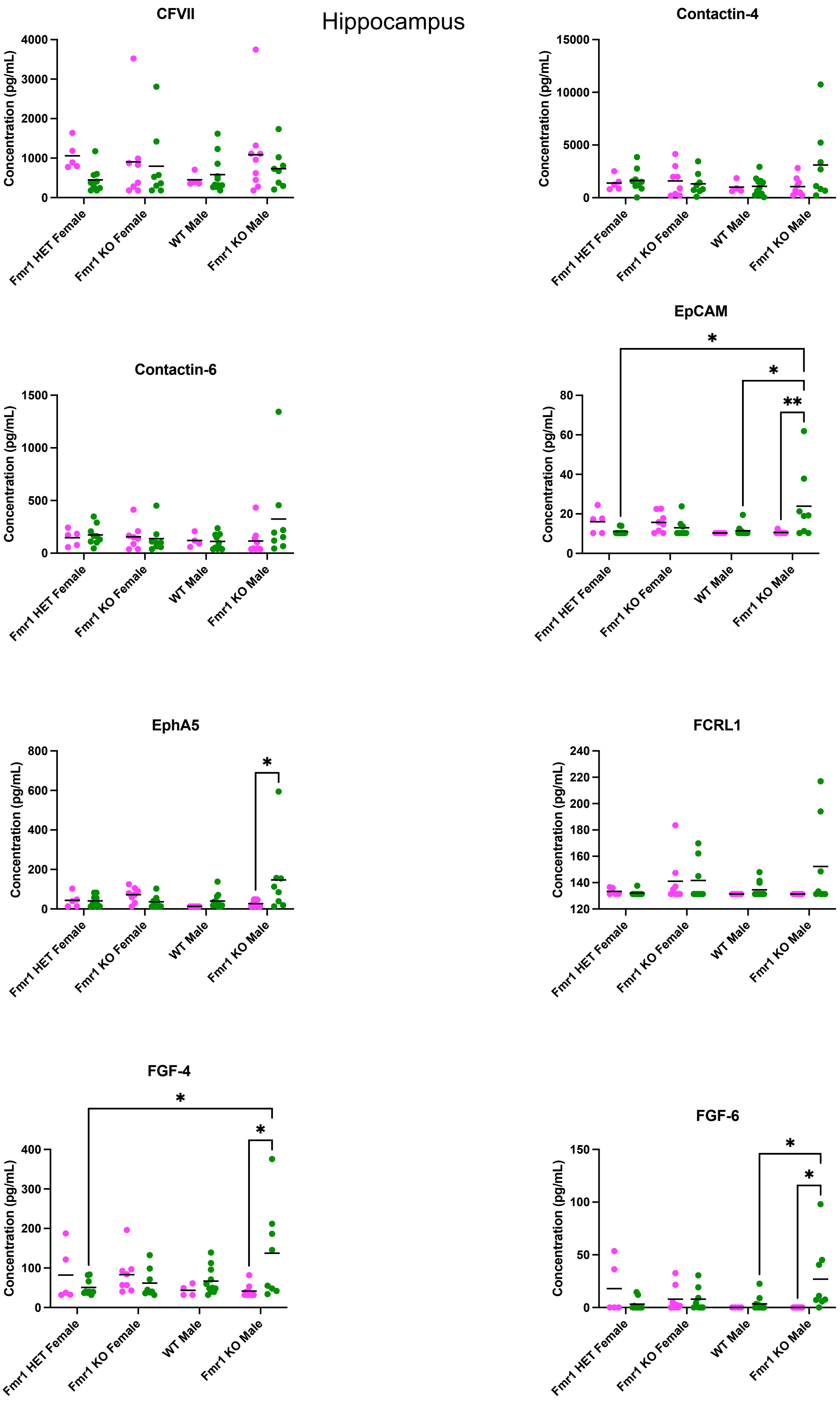
Hippocampus
CFVII
Concentration (pg/mL)
4000
3000
2000
1000
0
Fmr1 HET Female
Fmr1 KO Female
WT Male
Fmr1 KO Male
Contactin-4
15000
10000
5000
0
Contactin-6
1500
1000
500
0
EpCAM
*
*
**
80
60
40
20
0
EphA5
*
800
600
400
200
0
FCRL1
240
220
200
180
160
140
120
FGF-4
*
*
400
300
200
100
0
FGF-6
*
*
150
100
50
0

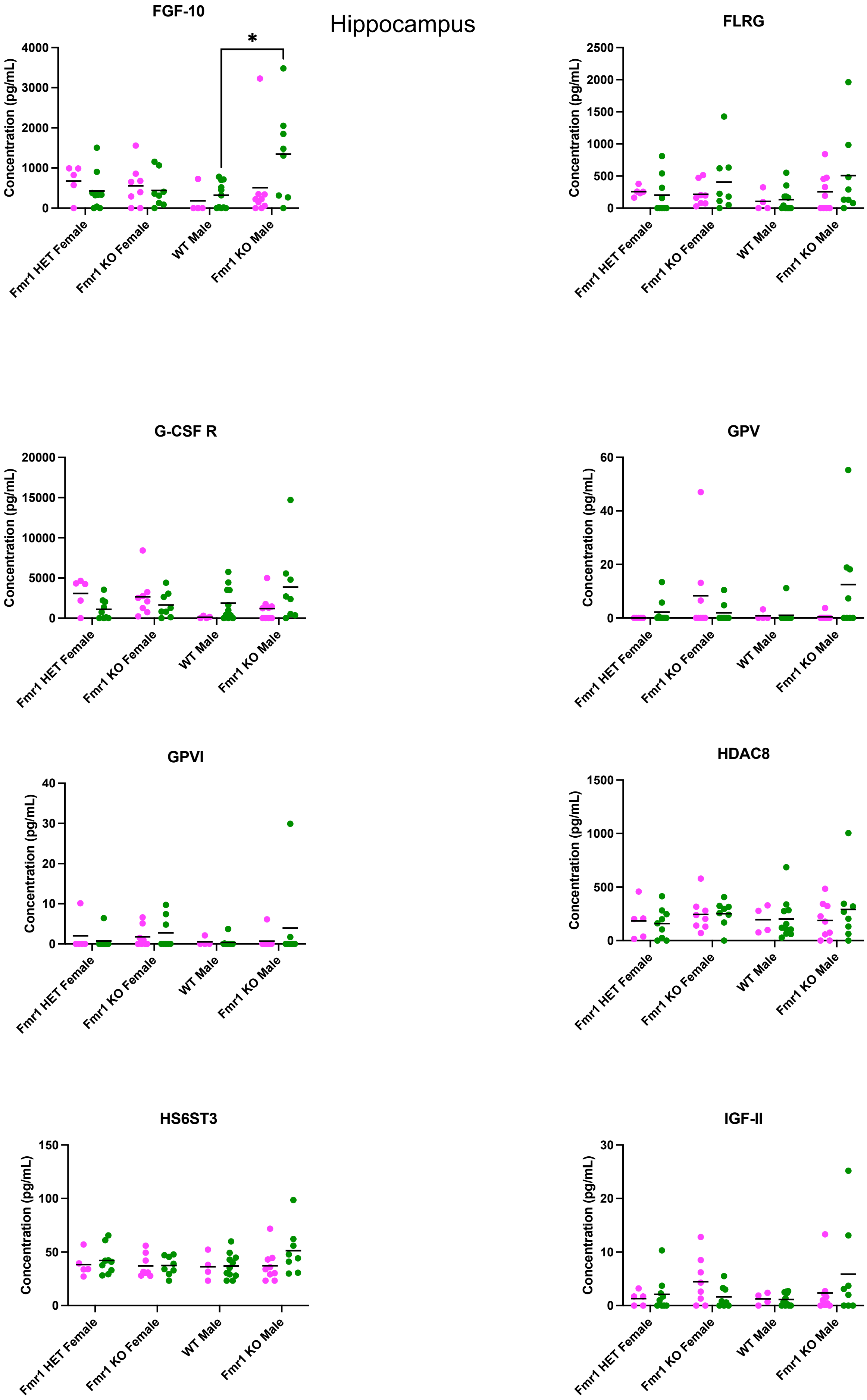
Hippocampus
FGF-10
FLRG
G-CSF R
GPV
GPVI
HDAC8
HS6ST3
IGF-II
Concentration (pg/mL)
Fmr1 HET Female
Fmr1 KO Female
WT Male
Fmr1 KO Male
*

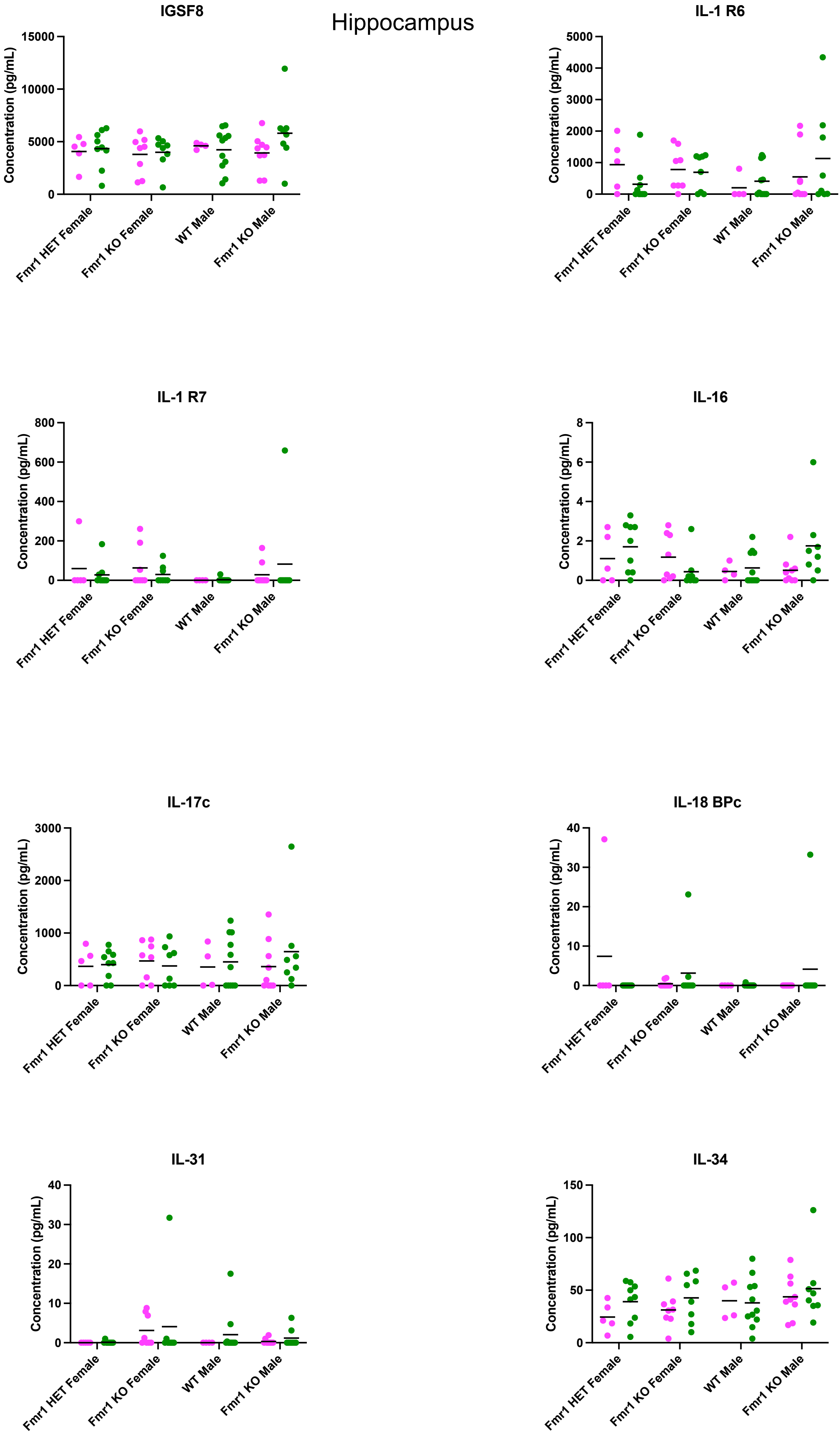
Hippocampus
IGSF8
IL-1 R6
IL-1 R7
IL-16
IL-17c
IL-18 BPc
IL-31
IL-34
Concentration (pg/mL)
Fmr1 HET Female
Fmr1 KO Female
WT Male
Fmr1 KO Male

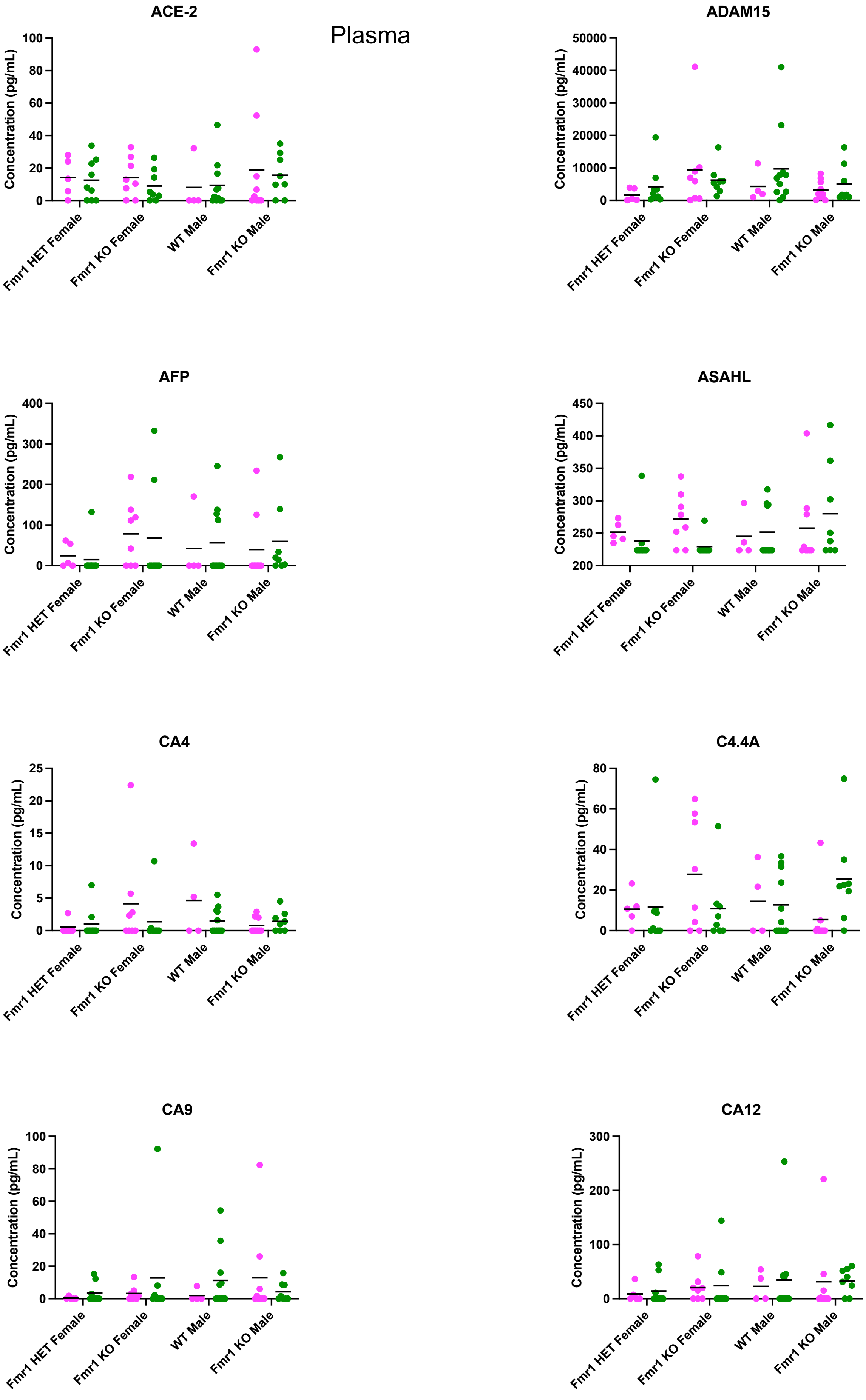
Plasma
ACE-2
Concentration (pg/mL)
0
20
40
60
80
100
Fmr1 HET Female
Fmr1 KO Female
WT Male
Fmr1 KO Male
ADAM15
Concentration (pg/mL)
0
10000
20000
30000
40000
50000
Fmr1 HET Female
Fmr1 KO Female
WT Male
Fmr1 KO Male
AFP
Concentration (pg/mL)
0
100
200
300
400
Fmr1 HET Female
Fmr1 KO Female
WT Male
Fmr1 KO Male
ASAHL
Concentration (pg/mL)
200
250
300
350
400
450
Fmr1 HET Female
Fmr1 KO Female
WT Male
Fmr1 KO Male
CA4
Concentration (pg/mL)
0
5
10
15
20
25
Fmr1 HET Female
Fmr1 KO Female
WT Male
Fmr1 KO Male
C4.4A
Concentration (pg/mL)
0
20
40
60
80
Fmr1 HET Female
Fmr1 KO Female
WT Male
Fmr1 KO Male
CA9
Concentration (pg/mL)
0
20
40
60
80
100
Fmr1 HET Female
Fmr1 KO Female
WT Male
Fmr1 KO Male
CA12
Concentration (pg/mL)
0
100
200
300
Fmr1 HET Female
Fmr1 KO Female
WT Male
Fmr1 KO Male

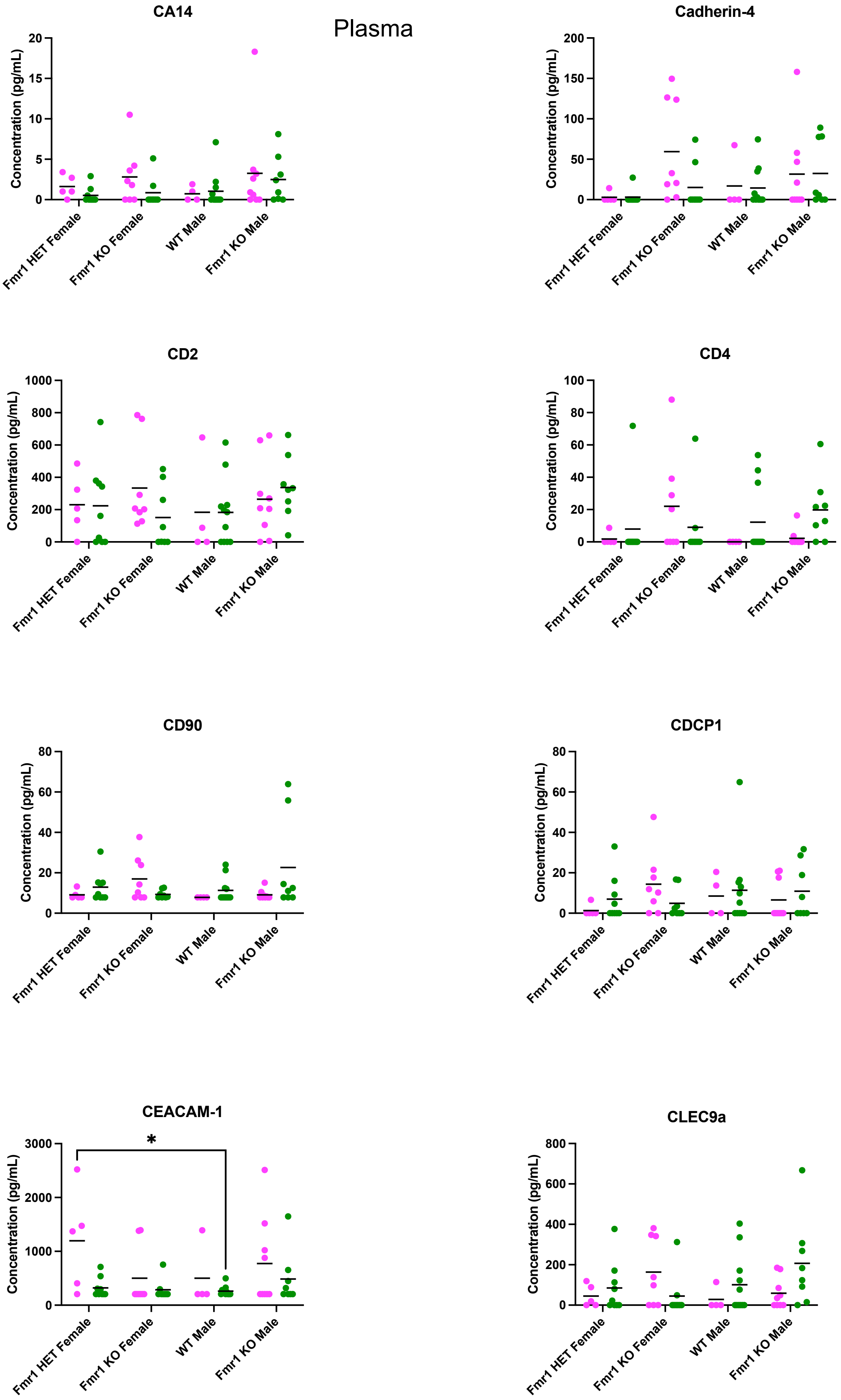
Plasma
CA14
Cadherin-4
CD2
CD4
CD90
CDCP1
CEACAM-1
CLEC9a
*
Concentration (pg/mL)
Fmr1 HET Female
Fmr1 KO Female
WT Male
Fmr1 KO Male
0
5
10
15
20
50
100
150
200
400
600
800
1000
40
60
80
1000
2000
3000

Plasma

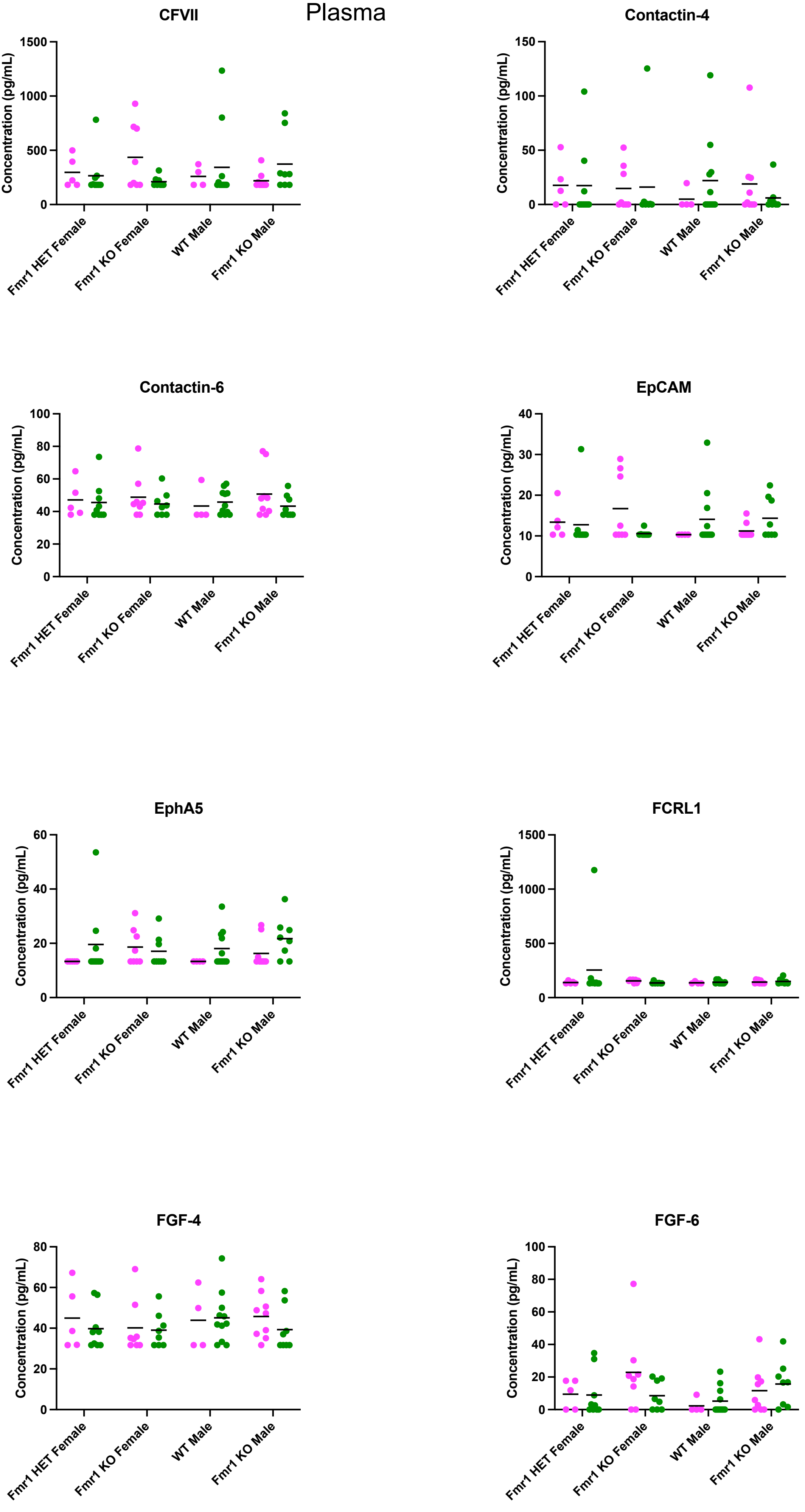
CFVII
Contactin-4
Contactin-6
EpCAM
EphA5
FCRL1
FGF-4
FGF-6
Concentration (pg/mL)
Fmr1 HET Female
Fmr1 KO Female
WT Male
Fmr1 KO Male

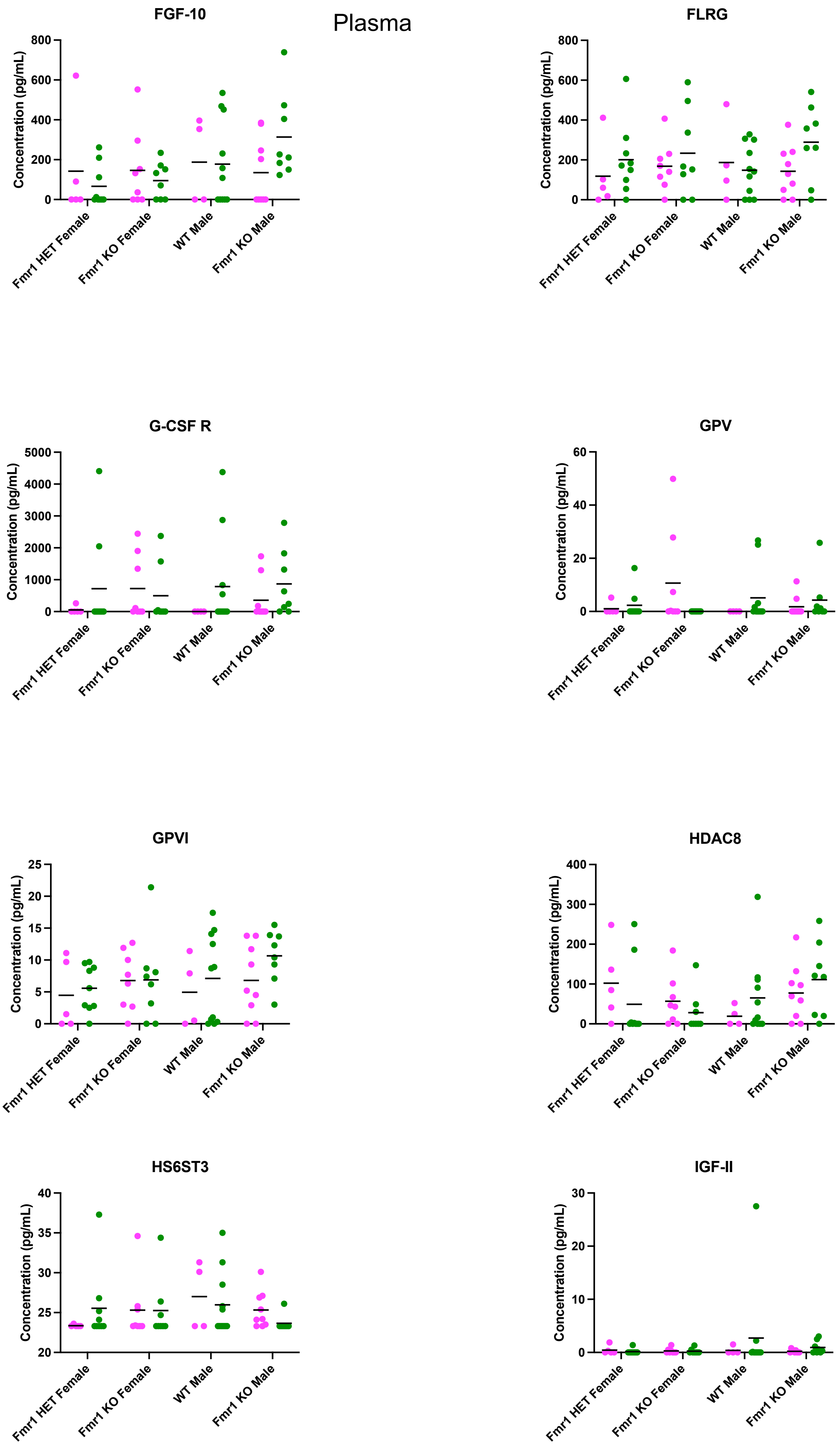
Plasma
FGF-10
FLRG
G-CSF R
GPV
GPVI
HDAC8
HS6ST3
IGF-II
Concentration (pg/mL)
Fmr1 HET Female
Fmr1 KO Female
WT Male
Fmr1 KO Male
0
200
400
600
800
1000
2000
3000
4000
5000
10
20
30
40
50
60
5
15
25
35
100
300

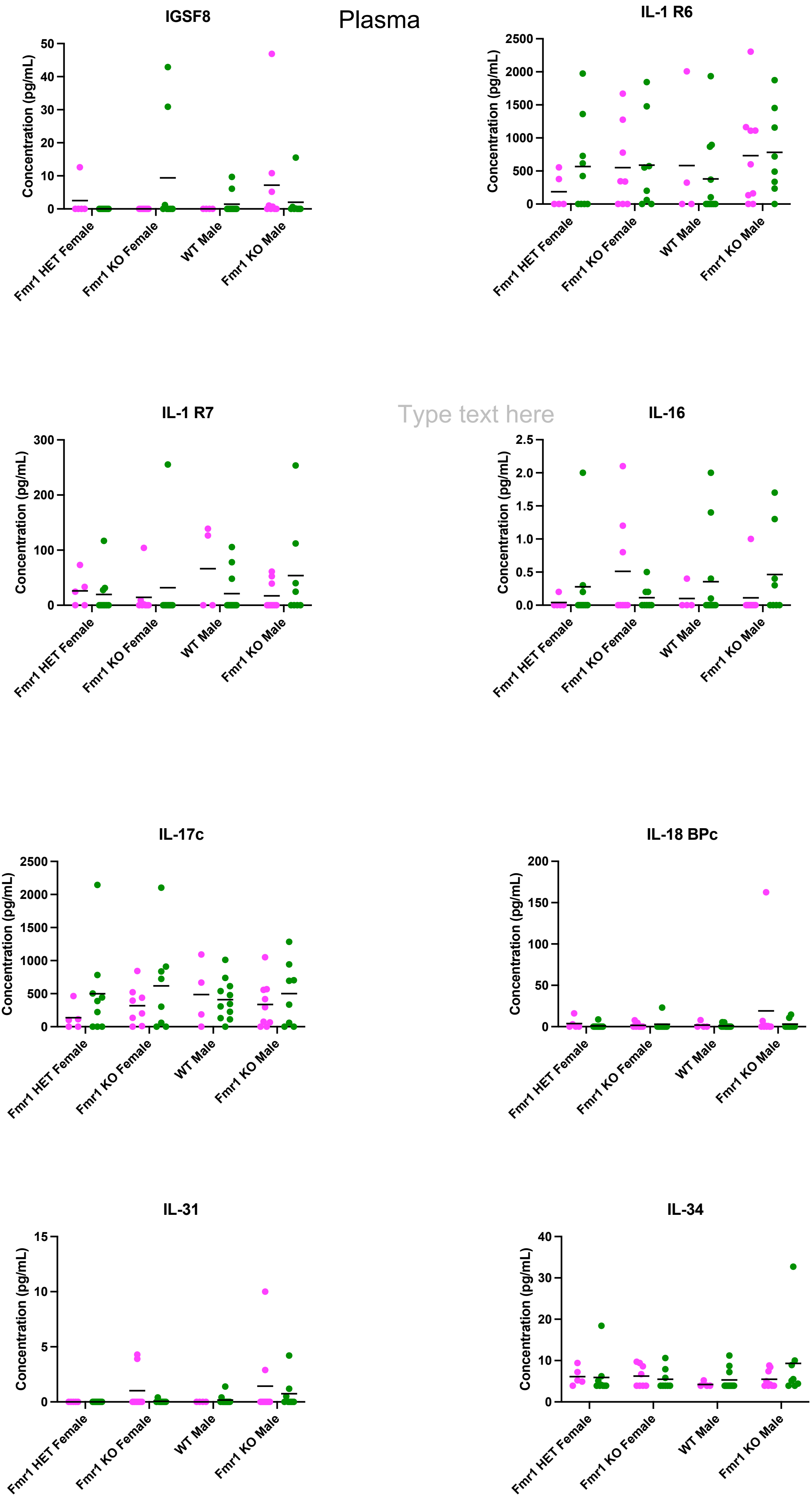
Plasma
Type text here
IGSF8
IL-1 R6
IL-1 R7
IL-16
IL-17c
IL-18 BPc
IL-31
IL-34
Concentration (pg/mL)
0
10
20
30
40
50
500
1000
1500
2000
2500
100
200
300
0.0
0.5
1.0
1.5
2.0
2.5
150
5
15
Fmr1 HET Female
Fmr1 KO Female
WT Male
Fmr1 KO Male
