# Supplementary material for: Soy Protein Isolate Affects Blood and Brain Biomarker Expression in a Mouse Model of Fragile X
Source: Int J Mol Sci. 2025 Jun 26;26(13):6137. doi: 10.3390/ijms26136137 (PMC12250412; doi:10.3390/ijms26136137)
Supplement: Supplementary file 1 [file ijms-26-06137-s001.zip › Supplementary Table S1.pdf]

Supplementary Table S1. Protein abbreviations and functions.

Protein	Function
ANG	Angiogenin (RNase5); formation of new blood vessels, cleaves tRNA to start a stress response
ASAM	Adipocyte-specific adhesion molecule; cell-cell adhesion
B7-1	CD80 (cluster of differentiation 80); crucial role in T cell activation by binding to CD28
B7-2	CD86 T-lymphocyte activation antigen; transmembrane protein that helps regulate the immune system
BAFF	B-cell activating factor; essential for B cell maturation and survival
BAMBI	BMP and activin membrane-bound inhibitor; transmembrane pseudoreceptor that inhibits TGF-beta signaling
BCHE	Butyrylcholinesterase; inactivates acetylcholine, breaks down toxic substances and drugs including cocaine, heroin and aspirin
βFGF	Fibroblast growth factor 2; regulates cell growth, tissue repair and wound healing
BID	BH3-interacting domain death agonist; key regulator of apoptosis and inflammation
BMP-6	Bone morphogenetic protein 6; regulates bone growth, iron homeostasis and lung function
BMP-7	Bone morphogenetic protein 7; growth factor with roles in bone and kidney development, liver regeneration and wound healing
C1QBP	Complement component 1Q subcomponent-binding protein; binds to C1Q protein, which is part of the complement system, and inhibits its activation
CA2	Carbonic anhydrase 2; catalyzes reversible hydration of CO_2 and bicarbonate, involved in acid-base balance and bone resorption
CA4	Carbonic anhydrase 4; catalyzes reversible hydration of CO_2 and plays crucial role in CO_2 and bicarbonate transport and acid-base balance
CADM3	Cell adhesion molecule; important role in axon guidance, myelination, and maintenance of the axonal architecture
CAMK4	Calcium/calmodulin-dependent protein kinase IV; serine/threonine kinase that regulates gene expression
CCL6	C-C motif chemokine ligand 6; cytokine that acts as a chemoattractant
CD34	Cluster of differentiation 34; transmembrane phosphoglycoprotein and cell surface antigen with crucial role in cell adhesion and differentiation
CD4	Cluster of differentiation 4; helps activate B cells to produce antibodies
CD14	Cluster of differentiation 14; cell surface protein that is part of innate immune system and recognizes pathogens and tissue damage
CD36	Cluster of differentiation 36; transmembrane glycoprotein with crucial role in lipid metabolism and signaling
CD45	Cluster of differentiation 45; transmembrane protein tyrosine phosphatase with crucial role in regulating T and B cell activation
CD47	Cluster of differentiation 47; cell surface protein that prevents phagocytosis by macrophages
CD74	Cluster of differentiation 74; cell surface receptor with crucial role in antigen presentation
CD99-L2	CD99-like 2; cell surface protein with role in leukocyte extravasation
CD160	Cluster of differentiation 160; glycophosphatidylinositiol (GPI)-anchored cell surface glycoprotein expressed on cytotoxic natural killer cells and T-cell subsets

CD164	Cluster of differentiation 164; glycoprotein that regulates cell adhesion, proliferation and migration, involved in development of skeletal muscle
CD200	Cluster of differentiation 200; membrane glycoprotein with role in immune regulation
CHL1	Cell adhesion molecule L1 like; cell adhesion molecular involved in nervous system development and synaptic plasticity
CHST4	Carbohydrate sulfotransferase 4; catalyzes transfer of sulfur group to the 6-position of non-reducing N-acetylglucosamine residues within mucin-type glycans
CLU	Clusterin; glycoprotein that functions as an extracellular chaperone and prevents protein aggregation and promotes cellular uptake of proteins for degradation
CMG-2	Capillary morphogenesis gene 2; transmembrane protein that regulates cell proliferation, tissue remodeling and angiogenesis
CNTN1	Contactin 1; cell adhesion molecule that helps bind neurons and glial cells together
CNTN2	Contactin 2; helps guides axons during brain development, forms cell-cell junctions between neurons and oligodendrocytes, and contributes to the formation of myelin sheaths
CRELD1	Cysteine-rich with EGF-like domains 1; crucial role in heart development
CST3	Cystatin C; cysteine protease inhibitor, biomarker for kidney function
CT-1	Cardiotrophin-1; protects heart cells from injury and stress
CXCL16	CXC chemokine ligand 16; type 1 membrane protein belonging the CXC chemokine family, acts as a chemoattractant
DAN	Differential screening-selected gene aberrative in neuroblastoma; secreted extracellular protein that acts as an antagonist to BMP signaling
EGF	Epidermal growth factor; stimulates growth and differentiation
EMMPRIN	Basigin aka CD147; transmembrane protein of the immunoglobulin superfamily that acts as inducer of matrix metalloproteinases
Eotaxin	Type of chemokine that acts as potent chemoattractant for eosinophils
EpCAM	Epithelial cell adhesion molecule; oncoprotein
EPHA1	Ephrin type-A receptor 1; receptor tyrosine kinase that regulates cell communication, differentiation and migration
EPHA4	Ephrin type-A receptor 4; crucial roles in developing and adult nervous system, particularly in synapse formation and axon guidance
EPHA5	Ephrin type-A receptor 5; involved in cell signaling and development, regulates axon guidance and synapse formation
EPHB1	Ephrin type-B receptor 1; receptor tyrosine kinase that regulates cell migration and adhesion and helps to form and maintain synapses
EPHB3	Ephrin type-B receptor 3, regulates cell death, migration and survival
EPI	Epiregulin; growth factor within the epidermal growth factor (EGF) family, stimulates proliferation by binding to the EGF receptor
E-selectin	CD62E; cell adhesion molecule on expressed on activated endothelial cells with crucial role in leukocyte recruitment to sites of inflammation
FAM3C	FAM3 metabolism regulating signaling molecule C; cytokine related to cell proliferation and tumor formation
FCRL5	Fc receptor-like protein 5; role in B cell activation and signaling
FDPS	Farnesyl diphosphate synthase; enzyme crucial in the mevalonate pathway, which is involved in the synthesis of cholesterol
FGF-4	Fibroblast growth factor 4; essential for development of limbs, heart and other organs during embryonic development

FGF-6	Fibroblast growth factor 6; crucial role in cell proliferation, differentiation, and muscle regeneration
FGF-10	Fibroblast growth factor 10; crucial for organ development particularly lung and limbs
FST	Follistatin; autocrine glycoprotein that binds and bioneutralizes members of the TGFβ superfamily particularly activin
FZD9	Frizzed-9; Wnt receptor involved in bone formation, lung cancer prevention and neural development
GAL-1	Galectin-1; a beta-galactoside-binding lectin with a crucial role in regulating immune cell homeostasis, inflammation and angiogenesis
GAL-3	Galectin-3; lectin involved in cell adhesion, inflammation and apoptosis
GAL-4	Galectin-4; lectin that functions in cell adhesion, intracellular signaling, stabilizing lipid rafts, tumor suppression and antimicrobial activity
GAPDH	Glyceraldehyde-3-phosphate dehydrogenase; multifunctional protein involved in glycolysis
GDF-9	Growth differentiation factor 9; key role in development of the ovaries and eggs, stimulates the growth of ovarian follicles
GHR	Growth hormone receptor; transmembrane protein that is a receptor for growth hormone
ICAM-1	Intracellular adhesion molecule-1, CD54; cell surface glycoprotein with crucial role in leukocyte recruitment to sites of inflammation
ICAM-5	Intracellular adhesion molecule-5, telencephalin; neuron-specific intracellular adhesion molecule expressed in the telencephalon (forebrain) of the mammalian brain
IGFBP-2	Insulin-like growth factor binding protein 2; regulates activity of IGFs
IGFBP-5	Insulin-like growth factor binding protein 5; regulates activity of IGFs
IGSF8	Immunoglobulin superfamily member 8; roles in immune function and cell adhesion
IL-1F8	Interleukin 1 family, member 8 a.k.a. IL-36 beta; cytokine that stimulates the production of inflammatory mediators and the secretion of epithelial defense proteins
IL-1b	Interleukin-1 beta; pro-inflammatory cytokine
IL-3 Rb	Interleukin-3 receptor beta; stimulates blood cell production
IL-12p40	Interleukin-12 beta subunit; protein subunit that forms part of IL-12 and IL-23
IL-15 Rα	Interleukin 15 receptor alpha; high affinity binding protein for IL-15, presents IL-15 to other immune cells to stimulate their proliferation and development
IL-17F	Interleukin 17F; pro-inflammatory cytokine that stimulates the production of other cytokines
IL-20	Interleukin 20; cytokine that regulates wound healing, tissue repair and inflammation
IL-20 Rβ	Interleukin-20 receptor beta; subunit of IL-20 and IL-22 receptors, involved in pro- and anti-inflammatory responses
IL-22 Rα1	Interleukin 22 receptor subunit alpha 1; primary receptor for IL-22, which is a key regulator of tissue homeostasis and response to injury/infection
IL-30	Interleukin-30 aka interleukin-27 p28; cytokine involved in immune regulation and inflammation
IP-10	Interferon gamma-induced protein 10; chemokine that helps attract activated T cells to sites of inflammation
JAM-A	Junction adhesion molecule-A; transmembrane protein that regulates barrier function, migration and inflammation

JAM-C	Junction adhesion molecule-C; transmembrane protein that plays a role in cell adhesion particularly in tight junctions between endothelial and epithelial cells
KC	Keratinocyte-derived chemokine, CXCL1; chemokine with crucial role in neutrophil recruitment and activation during inflammation and tissue repair
KLB	Klotho beta; regulate metabolism including glucose uptake, fatty acid metabolism and bile acid synthesis
LEP	Leptin; hormone produced by fat cells that regulates body weight and energy balance
LIF	Leukemia inhibitory factor; cytokine with crucial role in stem cell self-renewal, neuronal development, and embryonic implantation
LCN2	Lipocalin-2; secreted glycoprotein involved in innate immunity, iron homeostasis and inflammation
LRPAP	LDL receptor-related protein-associated protein; functions as a chaperone for LDL receptor family proteins
LTA4H	Leukotriene A4 hydrolase; enzyme that catalyzes the conversion of leukotriene A4 to leukotriene B4
MANF	Mesencephalic astrocyte-derived neurotrophic factor; neurotrophic factor localized to the endoplasmic reticulum
Marapsin	Serine protease 27, PRSS27; trypsin-like serine protease strongly expressed in pancreas
MFG-E8	Milk fat globule-epidermal growth factor-factor VIII; involved in phagocytosis of apoptotic cells, mammary gland morphogenesis, angiogenesis and tumor progression
MBL-2	Mannose-binding lectin 2; crucial component of innate immune system recognizing and binding to sugars on the surface of pathogens
MIG	Monokine induced by interferon-gamma a.k.a. CXCL9; chemoattractant that activates T cells and has anti-tumor properties
MMP-7	Matrix metalloproteinase-7; breaks down components of the extracellular matrix
MMP-10	Matrix metalloproteinase 10; tissue remodeling, wound healing, vascular remodeling
MOG	Myelin oligodendrocyte glycoprotein; expressed on the surface of myeline sheaths and oligodendrocyte membranes in the central nervous system with a role in myelin integrity and oligodendrocyte maturation
NAALADL-1	N-acetylated alpha-linked acidic dipeptidase like-1; ileum brush border membrane protein and aminopeptidase
NCAM-1	Neural cell adhesion molecule 1, CD56; cell adhesion molecule involved in cell-to-cell and cell-matrix interactions with crucial role in neuronal migration, neurite outgrowth, synaptogenesis, and synaptic plasticity and memory
NEP	Neprilysin; zinc-dependent membrane-bound enzyme that cleaves peptides
NOV	Nephroblastoma; cell adhesion, migration and proliferation
NTB-A	a.k.a. CD352; receptor that regulates immune cell activity
NTN4	Netrin-4; regulates cell growth, migration and survival
OLFM-1	Olfactomedian-1; glycoprotein that regulates axonal growth in the nervous system
OPG	Osteoprotegerin; glycoprotein that regulates bone remodeling
PODXL	Podocalyxin-like protein; cell surface adhesion molecule with role in maintaining glomerular filtration barrier in the kidneys and blood brain barrier

PPM1A	Protein phosphatase magnesium-dependent 1A; regulates physiological processes by dephosphorylating proteins including those involved in TGFβ signaling, NF-κB activation, and cell cycle control
PREP	Prolyl endopeptidase; serine protease that cleaves peptide bonds on the C-terminal side of prolyl residues and is involved in the maturation and degradation of peptide hormones and neuropeptides
PSMB6	Proteasome 20S subunit beta 6; involved in the proteolytic degradation of intracellular proteins
PSPN	Persephin; neurotrophic factor that helps maintain and develop the nervous system
PRL	Prolactin; hormone with role in lactation and breast development
Prss34	Mast cell protease-11 (MCP-11); trypsin-like protease with role in immune system
RSTN	Resistin; cysteine-rich peptide hormone linked to inflammation and metabolic disease
RGM-C	Repulsive guidance molecule C; glycoprotein involved in cell migration, differentiation, iron homeostasis and apoptosis
S100A9	S100 calcium-binding protein A9; role in inflammation and immune responses
SCF	Stem cell factor protein; cytokine that helps maintain and grow hematopoietic stem and progenitor cells
SEMA3C	Semaphorin 3C; plays a role in cancer, fibrosis and neuroinflammation
SPB10	Serpin B10; serine protease inhibitor
Siglec-3	Sialic acid-binding immunoglobulin-like lectin a.k.a. CD33; immune checkpoint receptor
SIRPA	Signal regulatory protein alpha; transmembrane protein involved in immune regulation, particularly in the interaction with CD47
SLAM	Signaling lymphocytic activation molecule; regulates immune cell development and function
SR-A1	Scavenger receptor A1; membrane glycoprotein with critical roles in innate immunity, cell apoptosis and proliferation
THOP1	Thimet oligopeptidase 1; metallopeptidase involved in processing peptides
Tie-2	Tyrosine kinase receptor; endothelial growth factor receptor
TIM-3	T-cell immunoglobulin and mucin domain 3; cell surface protein that regulates both innate and adaptive immune responses
TNF R1	Tumor necrosis factor receptor 1; membrane receptor that regulates inflammation, cell death and cell growth
TRANCE	Tumor necrosis factor-related activation-induced cytokine; regulates the immune system and bone development
TSC22D1	TSC22 domain family member 1; leucine zipper transcription factor stimulated by TGFβ and regulating the transcription of many genes
TWEAK R	Tumor necrosis factor-like weak inducer of apoptosis; cytokine in the TNF superfamily
UCHL1	Ubiquitin C-terminal hydrolase L1; thiol protease that hydrolyzes peptide bond at the C-terminal glycine of ubiquitin
uPAR	Urokinase-type plasminogen activator receptor; cell surface protein that binds to and activates urokinase
VLDL R	Very low density lipoprotein receptor; transports lipids
VNN-1	Vanin 1; enzyme that converts pantetheine into pantothenic acid cysteamine
